# A ligand-controlled regiodivergent three-component Heck-Tsuji reaction utilizing umpolung hydrazones enabled by palladium catalysis

**DOI:** 10.1038/s41467-026-76749-0

**Published:** 2026-08-20

**Authors:** Evan F. W. Chen, Ruofei Cheng, Faraz Alaghemand, Rustam Z. Khaliullin, Chao-Jun Li

**Affiliations:** 1 Department of Chemistry, and FRQNT Centre for Green Chemistry and Catalysis, McGill University, Montreal, QC Canada; 2 Department of Chemistry, McGill University, Montreal, QC Canada

**Keywords:** Homogeneous catalysis, Synthetic chemistry methodology

## Abstract

Developing sustainable, selective strategies for the multicomponent functionalization of allenes remains an ongoing challenge in synthetic chemistry. We report a ligand-controlled, regiodivergent palladium-catalyzed three-component Heck–Tsuji reaction of allenes, aryl iodides, and umpolung hydrazones. Leveraging the umpolung reactivity of hydrazones as sustainable carbanion surrogates enables the direct arylalkylation of allenes, forming two C–C bonds in a single step. The ligand environment governs reaction outcomes, switching selectivity between branched and linear adducts using simple, commercially available ligands. This method tolerates a wide range of aryl, heteroaryl, and alkyl substrates, operates under mild conditions, and is amenable to late-stage diversification of complex molecules. Computational studies suggest a plausible explanation for the mechanistic basis of the ligand-dependent regioselectivity, providing reasonable insights for design of divergent Pd-catalyzed multicomponent coupling reactions.

## Abstract (web summary)

The authors report a ligand-controlled, regiodivergent palladium-catalyzed three-component Heck–Tsuji reaction of allenes, aryl iodides, and umpolung hydrazones. Leveraging the umpolung reactivity of hydrazones as sustainable carbanion surrogates enables the direct arylalkylation of allenes, forming two C–C bonds in a single step.

## Introduction

Developing sustainable synthetic methods to access complex molecules from inexpensive and readily available hydrocarbons remains as a persistent challenge in synthetic chemistry. Among various intermediates, allenes have emerged as versatile synthetic building blocks due to their distinctive electronic and geometric characteristics. Defined by two consecutive C = C bonds, allenes exhibit differing reactivity and stability compared to their alkene and alkyne analogues. Over the past few decades, significant progress has been made in transition metal-catalyzed allene functionalization^1–5^. Through the exploitation of the intrinsic electrophilic nature of allenes, monofunctionalization has been widely developed^3,4,6–8^, whereas selective multifunctionalization through intermolecular multicomponent reactions remains an arduous task. Absolute control over chemo- and regioselectivity poses as major hurdles for multicomponent reactions, often leading to undesired side reactions or isomeric mixtures. Nevertheless, this strategy offers a powerful venue to introduce multiple functional groups simultaneously, enabling the efficient and rapid construction of molecular complexity and diversity in a single synthetic step.

**Fig. 1:**
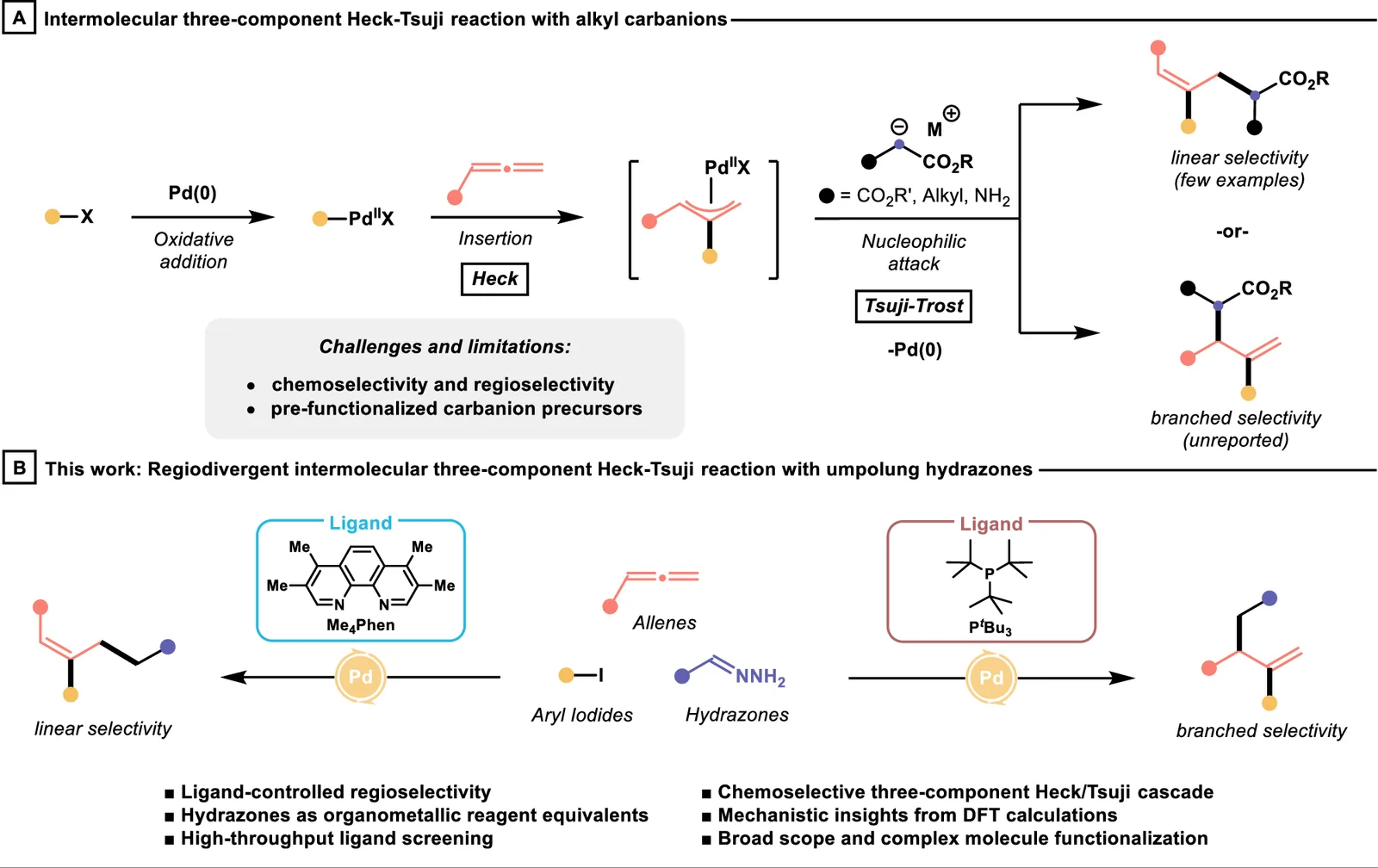
Heck-Tsuji reactions with carbanion nucleophiles. **A** Intermolecular three-component Heck-Tsuji reaction with alkyl carbanions. **B** This work: a ligand-controlled regiodivergent intermolecular three-component Heck-Tsuji reaction with umpolung hydrazones.

One approach to achieve allene multifunctionalization in an intermolecular multicomponent manner utilizes the Heck-Tsuji reaction. Catalyzed by palladium, this reaction has been widely applied to allene systems over the past few decades. In 1984, Tsuji and coworkers first reported the tandem Heck-Tsuji reaction of 1,2-dienes, in which treatment of an aryl halide, allene and secondary amine with a palladium catalyst afforded 2-aryl or alkenyl allylic amines^9^. Mechanistically, this reaction proceeds via oxidative addition of the aryl halide onto the Pd(0) catalyst, followed by a Heck-type insertion into the central sp carbon of the allene to generate a Pd-π-allyl intermediate. This intermediate subsequently undergoes nucleophilic addition by the amine in a Tsuji-Trost-type manner. Following its initial discovery, the Heck-Tsuji platform has been expanded to encompass not only allenes, but also various 1,*n*-diene systems, incorporating an array of heteroatom and carbon nucleophiles in both intra- and intermolecular variants^10–13^. Among these transformations, arylalkylation of allenes under Heck-Tsuji conditions represents a particularly appealing yet underdeveloped route to construct two distinct C–C bonds in a single operation, directly affording densely substituted alkenes of high synthetic value. Few existing examples predominantly utilize enolate derivatives as carbanions, though these strategies are often constrained by their heavy reliance on highly functionalized carbanion precursors or commercially unavailable additives. (Fig. 1A)^14–16^. Regioselectivity is typically dictated by sterics, as these strategies exclusively report alkylation of the least hindered terminus to afford the linear product. Due to higher steric demands, internal substitution yielding exclusively the branched product has not been achieved. Furthermore, the growing demand for chemical sustainability has intensified efforts to develop efficient catalytic methodologies for the direct valorization of renewable feedstocks into high-value chemical products, while minimizing resource consumption, costs, and waste generation.

Hydrazones, derived from readily accessible carbonyls and alcohols from renewable feedstocks, have previously been shown to exhibit umpolung reactivity to act as carbanions in transition metal-catalyzed reactions with various electrophiles. Over the past few years, our research group has been employing hydrazones as sustainable alternatives for organometallic reagents in nucleophilic addition or cross-coupling reactions^17,18^. While generating nitrogen gas and water as benign waste, hydrazones also exhibit tamed basicity/reactivity and air/moisture stability compared to organometallic reagents. Given the potential compatibility with Tsuji-Trost-type reactions, we speculated that hydrazones could be utilized as carbanion nucleophiles in the multicomponent Heck-Tsuji reaction with allenes and aryl iodides.

In this work, we report a three-component reaction with hydrazones as carbanion nucleophiles, where allenes are selectively difunctionalized to form two new C–C bonds (Fig. 1B). Notably, ligand-controlled regiodivergence was achieved, affording both exclusive linear and branched selectivity with simple, commercially available ligands. In contrast to the branched selectivity seen with other umpolung hydrazone reactions^19–22^, this linear selectivity also represents an alternative outcome for Tsuji-Trost-type reactions with umpolung hydrazones.

## Results and discussion

### Reaction optimization

**Fig. 2:**
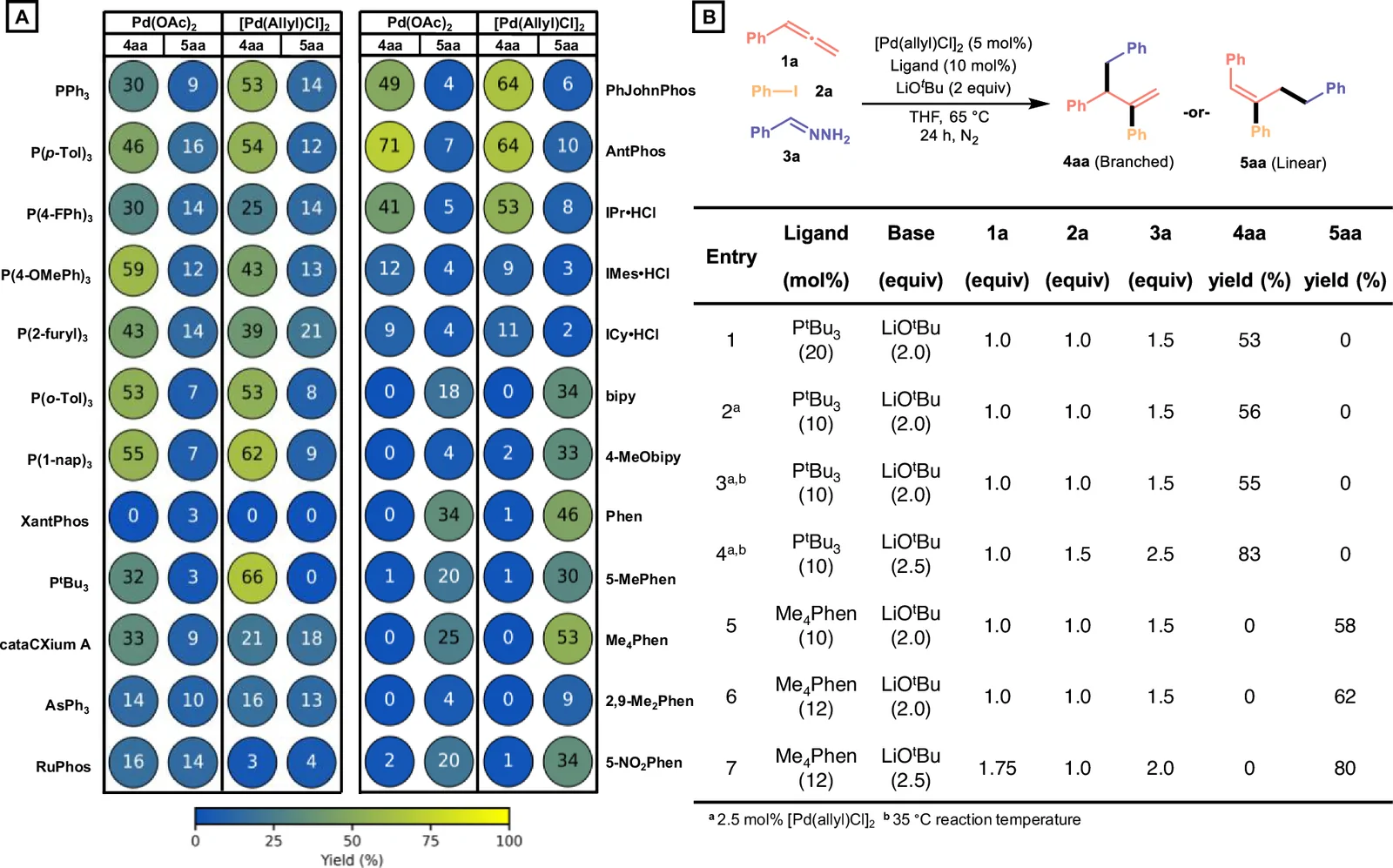
Reaction optimization. **A** Microscale high-throughput screening of palladium precatalysts and ligands. **B** Validation of HTE hits and further optimization of reaction conditions.

Our investigation began with the microscale high-throughput screening of palladium precatalysts and ligands using phenylallene (**1a**, 50 μmol), iodobenzene (**2a**, 1.0 equiv) and benzaldehyde hydrazone (**3a**, 1.5 equiv) as model substrates with lithium *tert*-butoxide (2.0 equiv) as the base at a reaction temperature of 65 °C (Fig. 2A). Initially, various substituted arylphosphine ligands were screened with both Pd(OAc)_2_ and [Pd(allyl)Cl]_2_, where the reaction afforded the branched-selective product **4aa** as the major product in 30–71% yield, a unique regioselectivity for multicomponent Heck-Tsuji reactions with allenes. In contrast, small amounts of linear product **5aa**, commonly observed in analogous systems, were detected with branched/linear (B/L) ratio ranging from 3:1 to 10:1. The general trend observed was that bulkier and strongly electron-donating ligands tend to favor the formation of **4aa** while suppressing **5aa**, leading to higher B/L ratios. Screening of bulky alkylphosphine ligands identified tri-*tert*-butylphosphine (P^*t*^Bu_3_), in combination with [Pd(allyl)Cl]_2_ as optimal, producing branched product **4aa** exclusively in a moderate yield of 66%. After establishing branched selectivity, we next sought conditions favoring the exclusive formation of the linear product **5aa**. Upon further inspection, *N*,*N*-biaryl bidentate ligands such as 2,2’-bipyridyl (bipy) or 1,10-phenanthroline (phen) derivatives proved to be ideal ligands in increasing linear selectivity. The combination of 3,4,7,8-tetramethyl-1,10-phenanthroline (Me_4_Phen) with [Pd(allyl)Cl]_2_ gave **5aa** in a moderate yield of 53% with exclusive linear selectivity. Encouraged by these high-throughput results, we scaled up our hits (P^*t*^Bu_3_ or Me_4_Phen with [Pd(allyl)Cl]_2_) from 50 μmol to 0.2 mmol to validate our reactions. We observed the identical selectivity in both cases, with **4aa** and **5aa** observed in 53% (entry 1) and 58% (entry 5) yield, respectively. Further optimization revealed that varying bases or solvents for the Pd/P^*t*^Bu_3_ system led to diminished or no product formation (see Supplementary Tables 2, 3). The reaction showed minimal temperature dependence, with **4aa** forming in 56% yield even at 35 °C. Furthermore, the reagent ratios were then screened, where the use of **1a** as the limiting reagent with **2a** (1.5 equiv), **3a** (2.5 equiv) and LiO^*t*^Bu (2.5 equiv) in excess improved the yield of **4aa** to 83% (entry 4). It was noted that with this catalytic system, minor *N*-arylation byproduct between **2a** and **3a** was observed, necessitating an excess of both. Similar conditions were applied to linear product **5aa**, where an increased Me_4_Phen loading of 12 mol% improved reproducibility (entry 6). The *N*-arylation byproduct was not observed with the Pd/Me_4_Phen catalyst system and therefore, iodobenzene **2a** was taken as the limiting reagent while **1a** (1.75 equiv), **3a (**2.0 equiv) and LiO^*t*^Bu (2.5 equiv) were employed in excess, producing **5aa** at 80% yield (entry 7). Control reactions were then conducted and confirmed that no product formation occurred in the absence of catalyst, ligand or base. Both conditions were also conducted under air, where diminished yields of **4aa** (52%) and **5aa** (40%) were observed (see Supplementary Tables 6, 10).

### Substrate scope

**Fig. 3:**
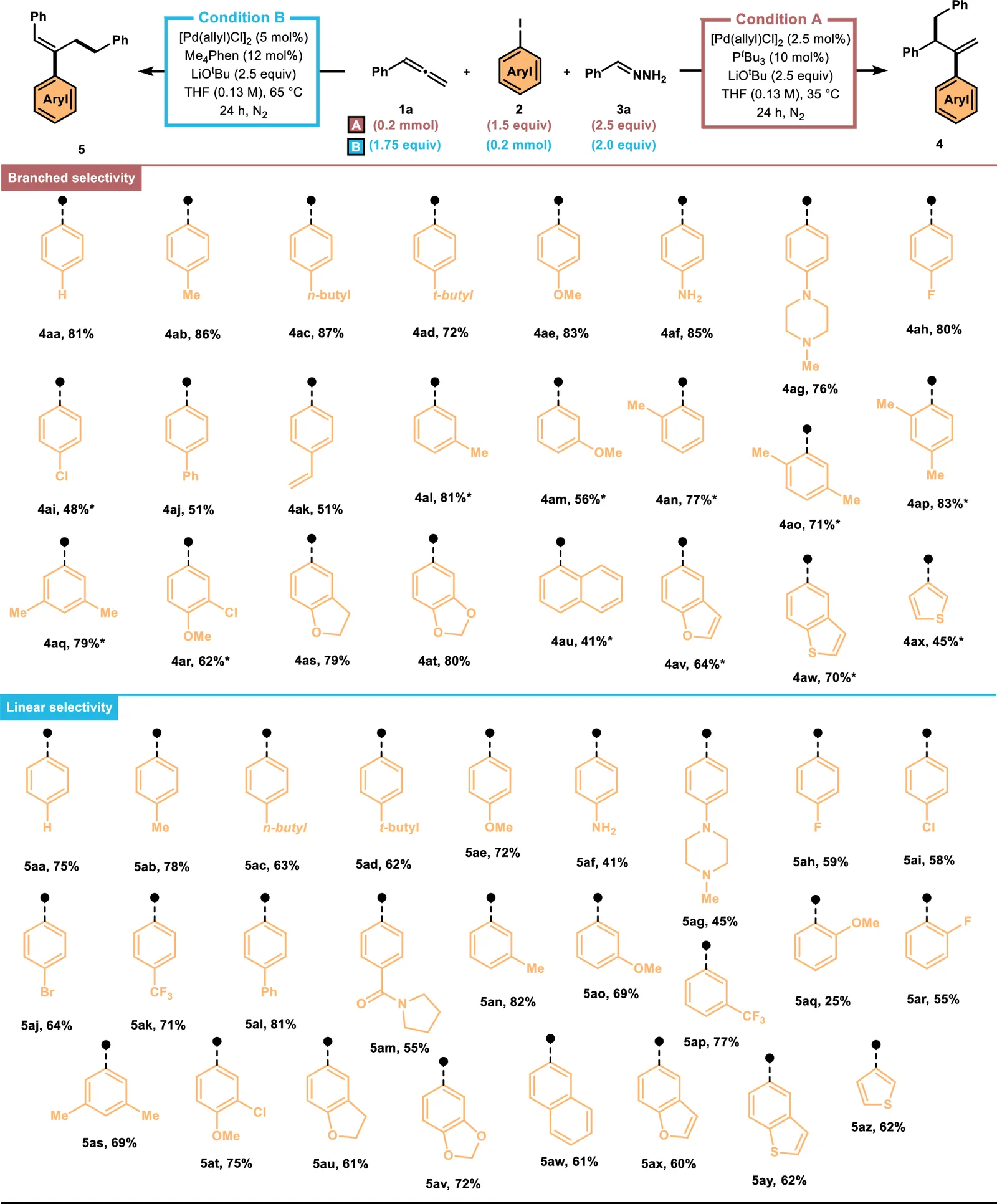
Aryl iodide substrate scope. Condition A: Phenylallene **1a** (0.2 mmol), aryl iodide **2** (0.3 mmol), benzaldehyde hydrazone **3a** (0.5 mmol), [Pd(allyl)Cl]_2_ (2.5 mol%), P^*t*^Bu_3_ (10 mol%), and LiO^*t*^Bu (0.5 mmol) in 1.5 mL THF at 35 °C for 24 h under N_2_. *Reactions were performed at 65 °C. Condition B: Phenylallene **1a** (0.35 mmol), aryl iodide **2** (0.2 mmol), benzaldehyde hydrazone **3a** (0.4 mmol), [Pd(allyl)Cl]_2_ (5 mol%), Me_4_Phen (12 mol%), and LiO^*t*^Bu (0.5 mmol) in 1.5 mL THF at 65 °C for 24 h under N_2_. Yields are reported as isolated yields.

We next investigated the substrate scope of this multicomponent Heck-Tsuji reaction under the optimized branched-selective (Condition A) and linear-selective (Condition B) conditions (Fig. 3). First, a broad range of aryl iodides proved to be compatible with our system. Under condition A, *para*-substituted phenyl iodides bearing electron-donating groups, such as methyl, *n*-butyl, *t*-butyl, methoxy, amino, and 4-methylpiperazin-1-yl, reacted smoothly to afford the branched products **4ab**–**4ag** in 72–87% yields. Electron-withdrawing or neutral substituents, including fluoro-, chloro-, phenyl- and vinyl-, were also tolerated, giving **4ai**–**4ak** in moderate yields (48–51%), except for **4ah** (80%). Steric effects had a pronounced impact on reactivity. For sterically demanding aryl iodides, elevated temperature (65 °C) was required to maintain efficiency. Several *meta*-, *ortho*-, and multi-substituted phenyl iodides furnished **4al**–**4ar** in good yields only at a higher temperature, while the bulkier naphthyl substrate afforded **4au** in a diminished 41% yield even at 65 °C. A variety of heteroaryl iodides were also evaluated. Dihydrobenzofuran- and benzodioxole-derived iodides delivered the branched products **4as** and **4at** in 79% and 80% yields, respectively. Likewise, benzofuran (**4av**, 64%) and thienyl derivatives (**4aw**, 70%; **4ax**, 45%) participated efficiently at 65 °C, although the five-membered thienyl iodide gave a lower yield. Under Condition B, complementary linear selectivity was observed, delivering **5aa**–**5az** in generally good yields across a similarly broad range of aryl iodides. In contrast to P^*t*^Bu₃, the less electron-rich Pd center generated by the Me₄Phen ligand enables efficient insertion of electron-deficient aryl iodides into the allene. Notably, halogen substrates (chloro- and bromo-) reacted effectively, highlighting the potential for orthogonal functionalization in downstream applications. *Meta*- and multiply substituted aryl iodides performed well in this reaction, giving **5an,** **5a** and **5as-5aw** in moderate to high yields (61–82%). Analogous to Condition A, sterically hindered aryl iodides such as *o-*methoxy and *o-*fluoro substrates showed lower conversion, affording products **4aq** and **4ar** in 25% and 55% yield. Heteroaryl iodides were also tested and gave the corresponding linear products **5ax**-**5az** in moderate yields (60–62%).

**Fig. 4:**
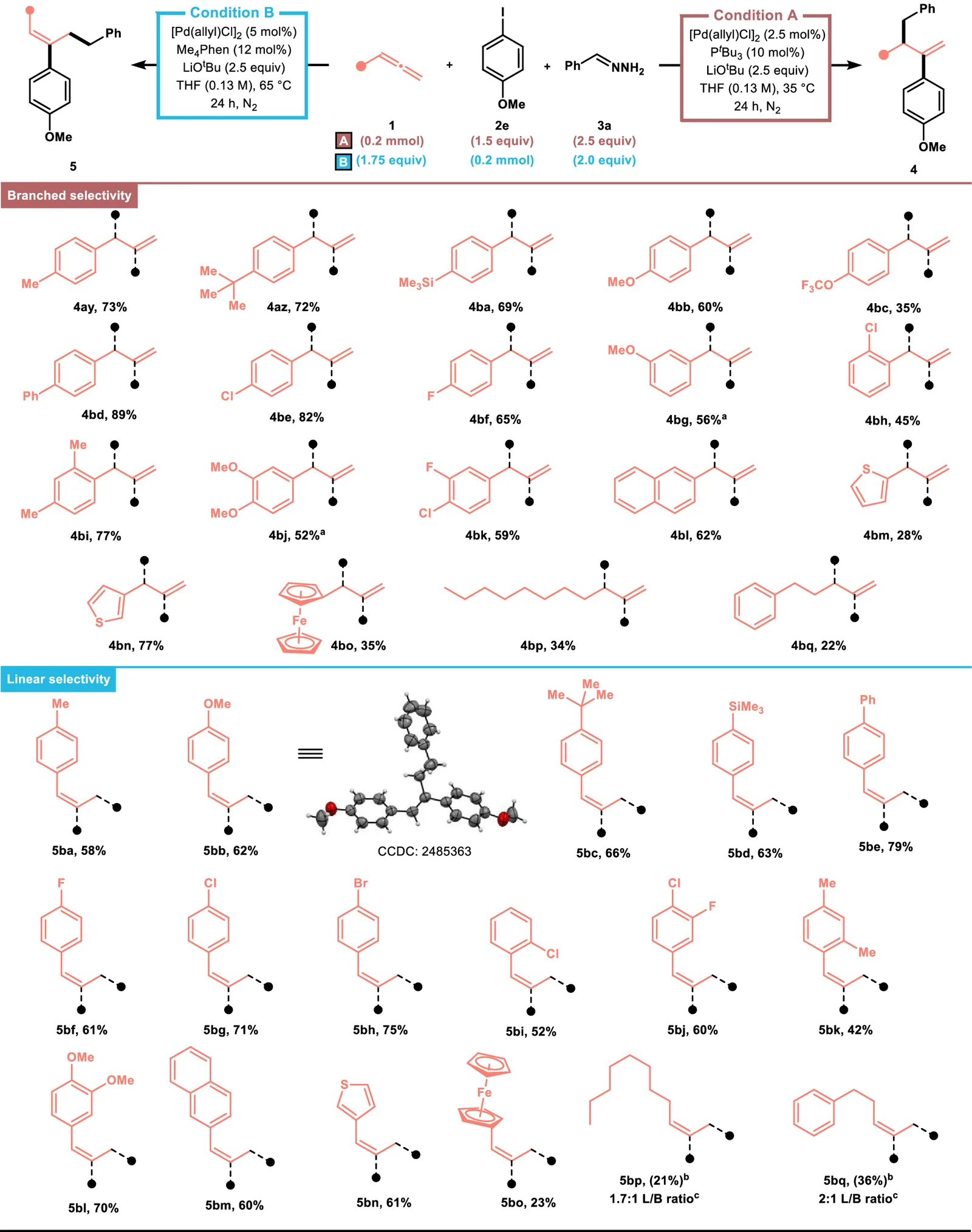
Allene substrate scope. Condition A: Allene **1** (0.2 mmol), 4-iodoanisole **2e** (0.3 mmol), benzaldehyde hydrazone **3a** (0.5 mmol), [Pd(allyl)Cl]_2_ (2.5 mol%), P^*t*^Bu_3_ (10 mol%), and LiO^*t*^Bu (0.5 mmol) in 1.5 mL THF at 35 °C for 24 h under N_2_. Condition B: Allene **1** (0.35 mmol), 4-iodoanisole **2e** (0.2 mmol), benzaldehyde hydrazone **3a** (0.4 mmol), [Pd(allyl)Cl]_2_ (5 mol%), Me_4_Phen (12 mol%), and LiO^*t*^Bu (0.5 mmol) in 1.5 mL THF at 65 °C for 24 h under N_2_. Yields are reported as isolated yields. ^a^Reactions were performed at 65 °C. ^b^Yields were determined by ^1^H NMR analysis, using CH_2_Br_2_ as an internal standard. ^c^Linear product **5** to branched product **4** ratio (L/B).

We then evaluated the scope of allene partners (Fig. 4). A variety of aryl- and alkyl-substituted terminal allenes were examined using 4-iodoanisole (**2e**) as coupling partners, and in most cases, the reaction proceeded smoothly to deliver the desired alkylation–arylation products with complementary regioselectivity. Under branched-selective conditions, *para*-substituted phenyl allenes bearing electron-donating (methyl, *t*-butyl, methoxy) or electron-withdrawing groups afforded the products (**4ay,** **4az,** **4bb,** **4bd**–**4bf**) in good to excellent yields (65–89%), while *para*-OCF_3_ (**4bc**), *meta*-OMe (**4bg**), and *ortho*-Cl substrates (**4bh**) gave lower yields. Also, sterically demanding polysubstituted phenylallenes and a 2-naphthyl allene furnished **4bi**–**4bl** in 52–77% yields. Ferrocene- and 2-thiophene-derived allenes were investigated and tolerated (**4bm** and **4bo**), but gave lower yields (28% and 35%). In addition to aryl allenes, alkyl-substituted allenes such as *n*-octyl and phenylethyl were compatible, affording **4aq** and **4ap** in 34% and 22% yields, respectively. By contrast, employing the linear-selective conditions furnished the corresponding linear adducts (**5ba**–**5bn**) in good yields (42–79%), across a similarly broad substrate range. Sterically hindered and heteroaryl allenes, including 3-thienylallene, reacted smoothly, while alkyl allenes gave the linear products in lower yields and poorer regioselectivities. Both conditions exhibited good functional group tolerance; notably, with silyl- (**4ba,** **5bd**) and bromo-substituted allenes (**5bh**) provided valuable handles for further derivatizations. The structure of **5bb** was further confirmed as the *E*-isomer by single-crystal X-ray analysis.

**Fig. 5:**
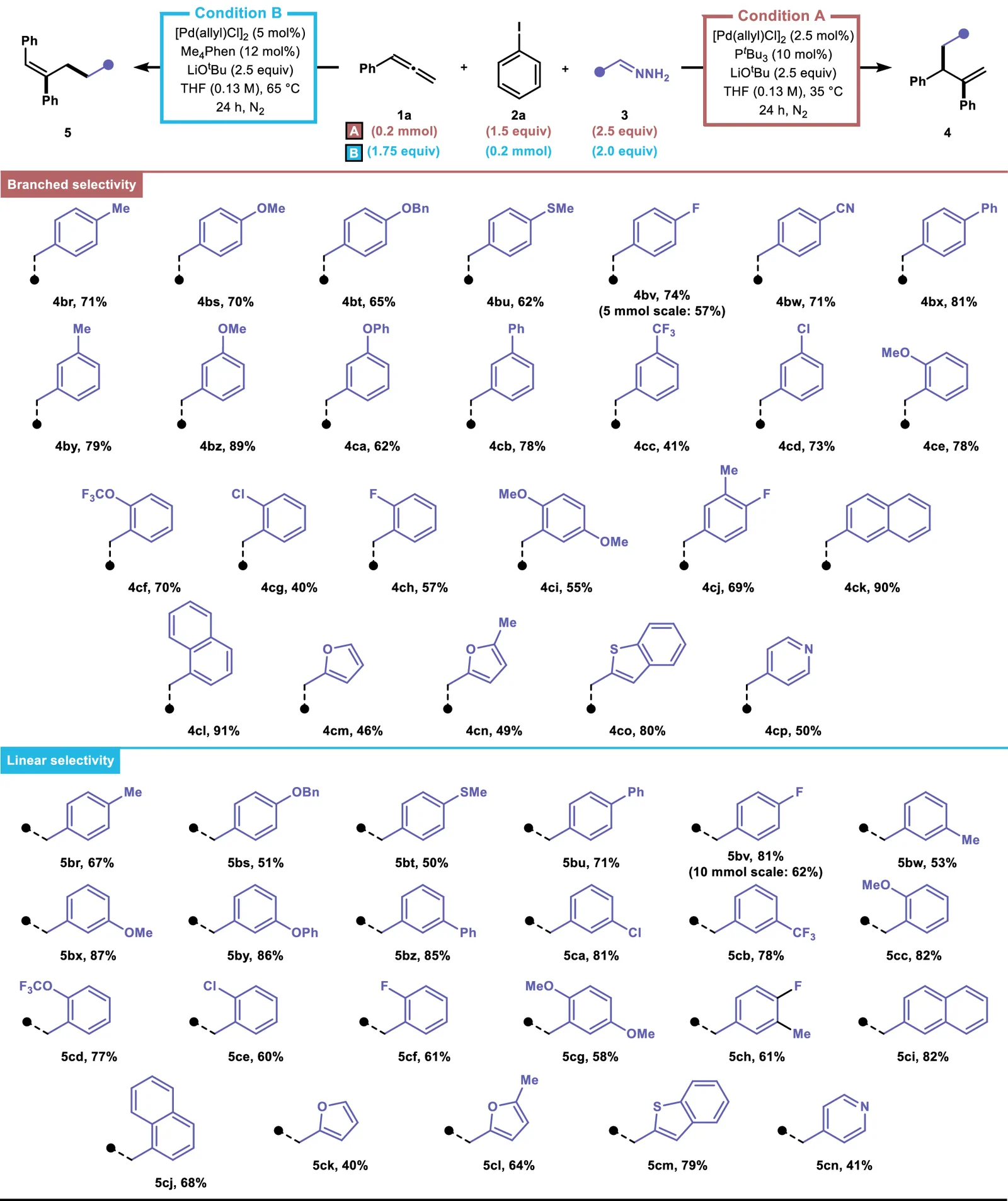
Hydrazone substrate scope. Condition A: Phenylallene **1a** (0.2 mmol), iodobenzene **2a** (0.3 mmol), hydrazone **3** (0.5 mmol), [Pd(allyl)Cl]_2_ (2.5 mol%), P^*t*^Bu_3_ (10 mol%), and LiO^*t*^Bu (0.5 mmol) in 1.5 mL THF at 35 °C for 24 h under N_2_. Condition B: Phenylallene **1a** (0.35 mmol), iodobenzene **2** (0.2 mmol), hydrazone **3a** (0.4 mmol), [Pd(allyl)Cl]_2_ (5 mol%), Me_4_Phen (12 mol%), and LiO^*t*^Bu (0.5 mmol) in 1.5 mL THF at 65 °C for 24 h under N_2_. Yields are reported as isolated yields.

**Fig. 6:**
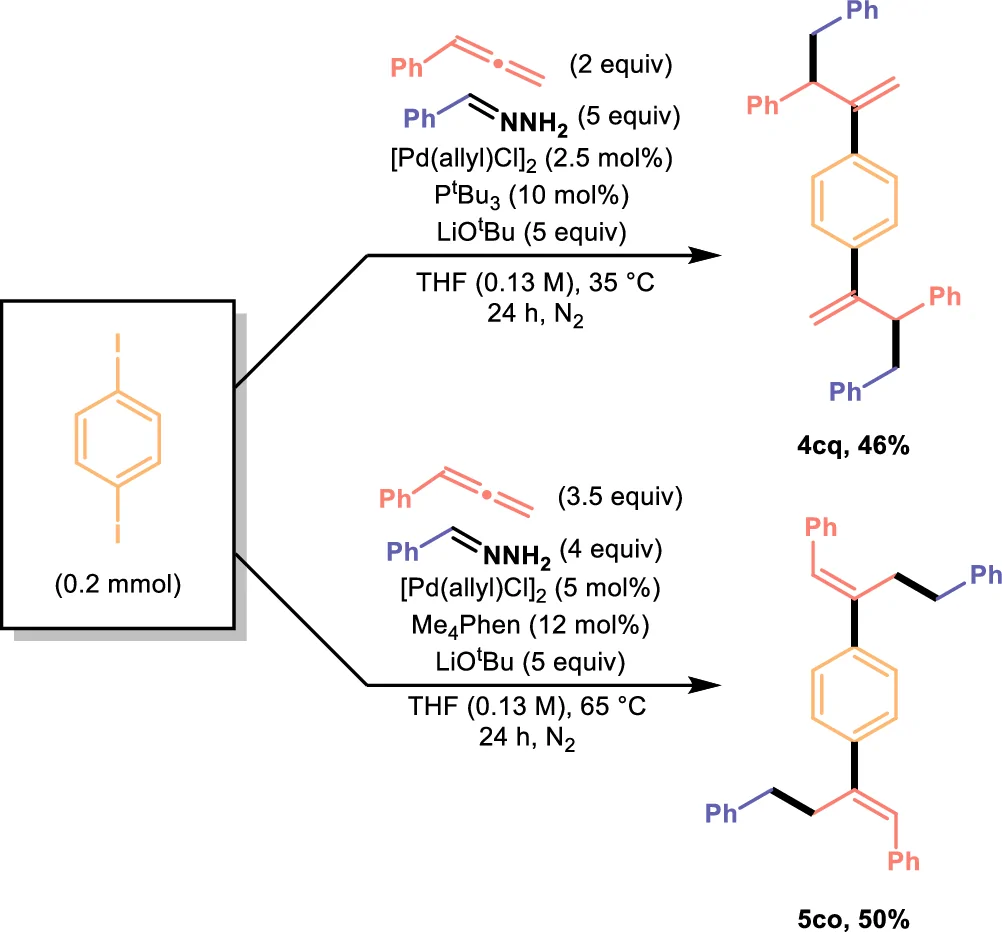
Difunctionalization of 1,4-diiodobenzene. Branched conditions: Phenylallene **1a** (0.4 mmol), 1,4-diiodobenzene (0.2 mmol), benzaldehyde hydrazone **3a** (1.0 mmol), [Pd(allyl)Cl]_2_ (2.5 mol%), P^*t*^Bu_3_ (10 mol%), and LiO^*t*^Bu (1.0 mmol) in 1.5 mL THF at 35 °C for 24 h under N_2_. Linear conditions: Phenylallene **1a** (0.7 mmol), 1,4-diiodobenzene (0.2 mmol), benzaldehyde hydrazone **3a** (0.8 mmol), [Pd(allyl)Cl]_2_ (5 mol%), Me_4_Phen (12 mol%), and LiO^*t*^Bu (1.0 mmol) in 1.5 mL THF at 65 °C for 24 h under N_2_. Yields are reported as isolated yields.

Finally, we examined the scope of hydrazone partners (Fig. 5). A broad range of aryl aldehyde-derived hydrazones were compatible under both branched- and linear-selective conditions. *Para*-, *meta*-, and *ortho*-substituted hydrazones bearing electron-donating (such as Me, OMe, OBn, SMe, and OPh) or electron-withdrawing groups (such as F, Cl, CN, Ph, and CF_3_) reacted smoothly to afford the corresponding products **4br**–**4ch** (40–89%) and **5br**–**5cf** (50–87%). Multi-substituted and naphthyl-derived hydrazones also performed well, delivering **4ci**–**4cl** and **5cg**–**5cj** in up to 91% yield. Heteroaromatic substrates (substituted with furyl, thienyl, and pyridyl groups) were likewise tolerated, furnishing **4cm**–**4cp** and **5ck**–**5cn** in 40–80% yields. The protocol also proved robust on gram scale, affording **4bv** (57%, 5 mmol scale) and **5bv** (62%, 10 mmol scale). Moreover, difunctionalization of 1,4-diiodobenzene delivered **4cq** and **5co** in moderate yields of 46% and 50% (Fig. 6).

**Fig. 7:**
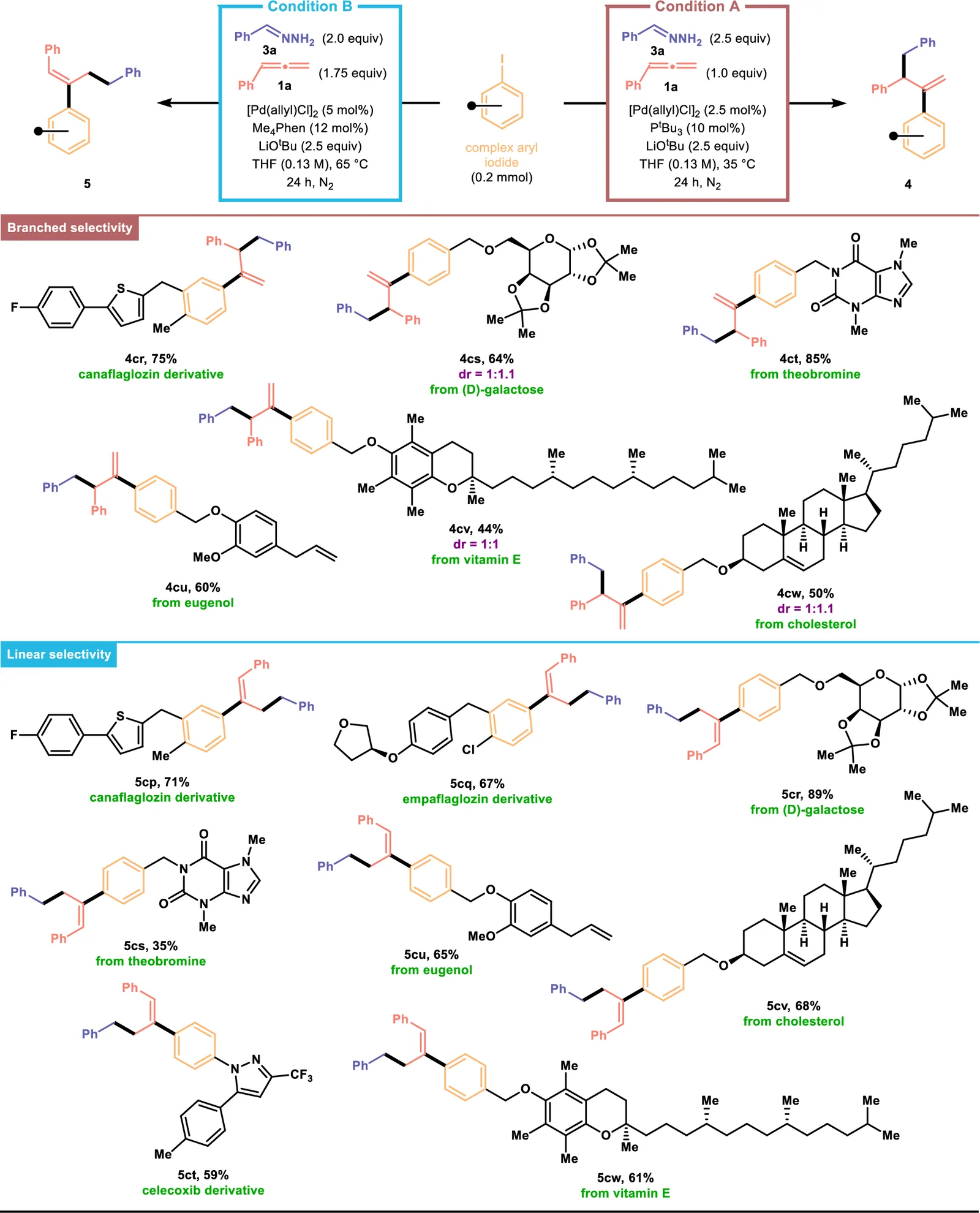
Complex molecule modifications. Condition A: Phenylallene **1a** (0.2 mmol), aryl iodide **2** (0.2 mmol), benzaldehyde hydrazone **3a** (0.5 mmol), [Pd(allyl)Cl]_2_ (2.5 mol%), P^*t*^Bu_3_ (10 mol%), and LiO^*t*^Bu (0.5 mmol) in 1.5 mL THF at 35 °C for 24 h under N_2_. Condition B for: Phenylallene **1a** (0.35 mmol), aryl iodide **2** (0.2 mmol), benzaldehyde hydrazone **3a** (0.4 mmol), [Pd(allyl)Cl]_2_ (5 mol%), Me_4_Phen (12 mol%), and LiO^*t*^Bu (0.5 mmol) in 1.5 mL THF at 65 °C for 24 h under N_2_. Yields are reported as isolated yields.

To demonstrate the practicality of this method, we applied it to late-stage modification of bioactive molecules and natural product-derived aryl iodides (Fig. 7). Under branched-selective conditions, substrates derived from canagliflozin, (D)-galactose, theobromine, eugenol, vitamin E, and cholesterol afforded adducts (**4cr**–**4cw**) in moderate to good yields (44–75%). Complementary linear-selective conditions delivered products (**5cp–5cw**) in 35–89% yield, which includes derivatives of canagliflozin, empagliflozin, (D)-galactose, theobromine, celecoxib, eugenol, cholesterol and vitamin E.

### Mechanistic Investigation

**Fig. 8:**
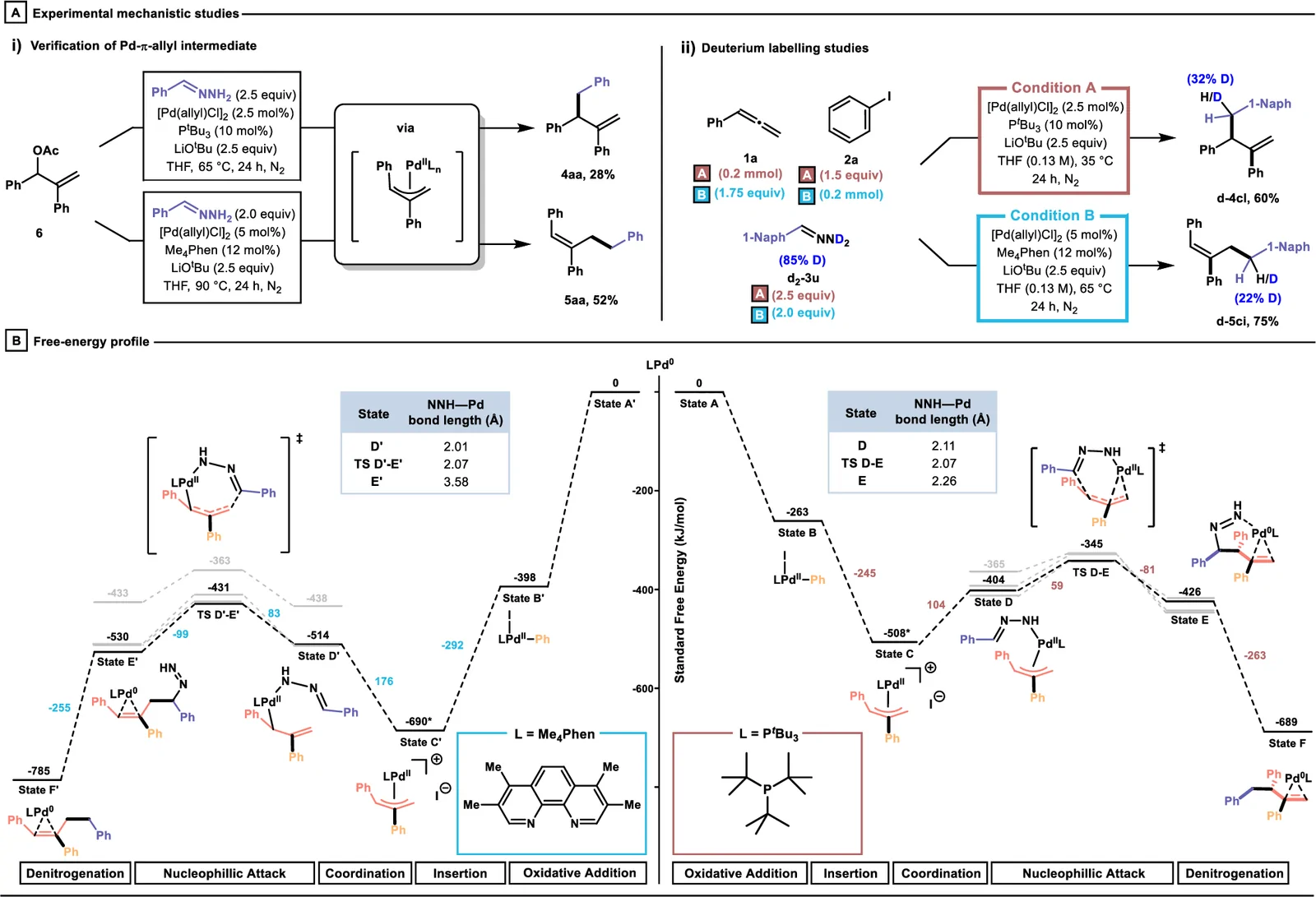
Mechanistic studies. **A** Experimental mechanistic studies - i) Verification of Pd-π-allyl intermediate and ii) deuterium labelling studies. **B** The ωB97XD/6-311 + + G(d,p)//ωB97XD/6-31 G(d,p) free energy profile of the reaction with the Pd-Me_4_Phen and Pd-P^*t*^Bu_3_ catalyst. The gray states represent different pathways for C-C bond formation, leading to *cis*/*trans* or linear/branched products. Major products (highlighted with the black line) are determined to be the main product based on kinetics calculations (see Supplementary Information). *States C and C’ are ion pairs; their free energy is considerably more sensitive to the treatment of solvation than those of the neutral species and is therefore less reliable.

To gain preliminary mechanistic insights into this Pd-catalyzed multicomponent of Heck–Tsuji reaction, we conducted a series of control and deuterium-labeling experiments. (Fig. 8A). We first validated the presence of a Pd-π-allyl intermediate, through the in-situ generation of this intermediate with the corresponding Pd-P^*t*^Bu_3_ or Pd-Me_4_Phen catalyst systems and utilizing 1,2-diphenylallyl acetate **6** as the precursor. Upon oxidative addition, the corresponding π-allylpalladium intermediate was converted to **4aa** (28% NMR yield) and **5aa** (52% NMR yield) at elevated temperatures (Figure 8Ai). Subsequently, deuterium labelling studies were performed with deuterated 1-naphthaldehyde hydrazone **3u**. Deuterated **4cl** (60% yield) and **5ci** (75% yield) were observed with deuteration at the benzylic position (32% and 22% deuteration, Figure 8Aii), indicating incorporation of deuterium from the hydrazone substrate. Although partial H/D scrambling is likely occurring under the reaction conditions.

To obtain a deeper understanding of the Heck–Tsuji reaction, density functional theory (DFT) calculations were performed to examine the reaction mechanism and to rationalize the distinct products formed under the two catalytic systems. 1-Phenylallene **1a**, iodobenzene **2a** and benzaldehyde hydrazone **3a** were selected as model substrates, with the catalyst coordinated to two different ligands. All calculations were carried out using the ωB97XD exchange–correlation functional as implemented in Gaussian 16 (Revision B.01). Geometry optimizations were performed using the LANL2DZ ECP basis set for Pd and I atoms, and the 6–31 G(d,p) basis set for all other atoms. Final energies of optimized structures were then calculated using the LANL2DZ and the larger 6–311 + + G(d,p) basis set with solvent effects described using the polarizable continuum model^23^ (see Supplementary Information for details).

Due to the favorability of the branched or linear products by specific metal ligand complexes, stable intermediate structures and key transition states were calculated for catalysts with both P^*t*^Bu_3_ and Me_4_Phen ligands. The free energy profiles obtained for the P^*t*^Bu_3_ and Me_4_Phen-based catalysts are shown in Fig. 8B. The computed energy profiles show that both catalytic systems proceed through predominantly exergonic steps with significant barriers in each pathway being 59 kJ/mol for P^*t*^Bu_3_ and 83 kJ/mol for Me_4_Phen. The higher barrier in the Me_4_Phen pathway is in agreement with elevated temperatures and higher catalyst loading observed in our experimental results.

As differing ligands produce varying regioselectivities, additional calculations were performed to investigate the origin of the unique selectivity of each catalyst, where four possibilities for the crucial second C–C bond formation step were considered for each ligand. These four possibilities correspond to distinct isomers of state D, each leading to a different product: branched, linear-*trans*, or linear-*cis*. Relative time-dependent concentrations of all four possible product states were computed for both ligands using DFT energies to determine the dominant products in the reaction.

The time-dependent concentrations (see Supplementary Fig. 5) show that the branched product is the major product formed with the Pd-P^*t*^Bu_3_ catalyst. This outcome is a result of the greater stability of the transition state TS D-E for the branched product (see Supplementary Fig. 5a). This stability arises from the coordination environment of Pd and the orientation of the phenyl rings. The transition states leading to the linear products are higher in energy, with the linear-*trans* pathway proceeding through a less favorable three-coordinated Pd ion (see Supplementary Table 12) and the linear-*cis* pathway further destabilized by steric effects. The kinetics calculations for the Pd-Me_4_Phen catalyst predict that the main linear-*trans* product—the one observed experimentally—is formed not only because of the stability of the initial D’ state but also because of the greater stability of the final E’ state and the lowest barrier leading to it. Although the initial states leading to the branched product are slightly more stable (as in the case of Pd-P^*t*^Bu_3_ catalyst) their transformation is endergonic and therefore reversible, which allows the most stable *trans*-linear product to form (see Supplementary Fig. 6). The stability of different Pd-Me_4_Phen isomers of state E’ is primarily determined by the proximity of the phenyl groups to each other, with linear*-trans* isomer being the most stable due to the optimal spatial distribution of the phenyl groups. In contrast, the least stable linear-*cis* isomer is the most crowded of all.

Thus, our kinetic model shows that the lower transition state barrier determines the experimentally observed branched product for the Pd-P^*t*^Bu_3_ catalyst, in a classical Curtin–Hammett type scenario. In contrast, the greater reversibility of the reaction in case of the Me_4_Phen catalyst allows for the accumulation of the more stable linear product E′.

To rationalize the different product distributions computed for the two catalytic systems, it is important to note that the energy ordering of the key intermediates D and E is similar for both ligands (see Supplementary Figs. 5a, 6a): intermediate D is slightly more stable for pathways leading to the branched product, whereas intermediate E is more stable for pathways leading to the linear product. With the similar relative energies of reactant and product states, the observed difference in product selectivity is explained primarily by the different relative energies of the D–E transition states. In both catalytic systems, the major product is formed via the lowest-energy transition state. Similar energetics of reaction endpoints and different energies of transition states are possible if the transition state is reactant-like (early TS) for the Pd-P^*t*^Bu_3_ catalyst, whereas the transition state is more product-like (later TS) for the Pd–Me_4_Phen catalyst. This conclusion is consistent with a more exothermic transformation for Pd-P^*t*^Bu_3_ and the less exothermic and even endothermic nature of this step for Pd–Me_4_Phen (Fig. 8B). This difference in exothermicity arises from the coordination of the NNH group of the reactant: in the monodentate P^*t*^Bu_3_ system, the NNH group remains coordinated to Pd throughout the C–C bond formation step (State E), stabilizing the product state, while in the bidentate Me_4_Phen system, the NNH group dissociates from Pd, reducing product stabilization (State E) (Supplementary Tables 12, 13). Thus, the monodentate versus bidentate nature of the ligand subtly tunes the reaction energetics, shifting control between early and late transition states and ultimately dictating regioselectivity.

In summary, we have developed a high-yielding, chemoselective and regio-divergent-selective Pd-catalyzed three-component intermolecular Heck-Tsuji reaction with aryl iodides, allenes and umpolung hydrazones. The exclusive regioselectivity of hydrazone nucleophilic attack is governed by the choice of P^*t*^Bu_3_ or Me_4_Phen as the ligand enabling access to either branched or linear products. DFT calculations offer mechanistic insight, elucidating a reasonable origin of the unique regioselectivity swap. Highlights of this methodology include the broad substrate scope of various substituted aryl aldehyde-derived hydrazone, aryl/alkyl allenes and aryl iodides; high functional group tolerance, and late-stage modification of complex, diverse molecules demonstrate its synthetic utility and functional group tolerance. Importantly, readily accessible hydrazones enable a sustainable and divergent arylalkylation of allenes, forming two new carbon–carbon bonds while releasing only innocuous nitrogen gas as a byproduct.

## Methods

### General procedure for the three-component Heck-Tsuji reaction with aryl halides, allenes and umpolung hydrazones

#### General procedure A (for branched selectivity)

To a 2–5 mL oven-dried microwave vial with a stir bar, allylpalladium chloride dimer (1.8 mg, 0.005 mmol, 0.025 equiv), tri-*tert*-butylphosphine (10 wt% solution in hexanes, 0.02 mmol, 0.1 equiv) were added inside a glovebox. Then, 0.5 mL of dry THF was then added and the mixture was stirred for 1 h. Lithium *tert*-butoxide (40 mg, 0.5 mmol, 2.5 equiv), iodoarene (0.3 mmol, 1.5 equiv), allene (0.2 mmol) and hydrazone (0.5 mmol, 2.5 equiv) were added sequentially and an additional 1 mL of dry THF was added. The microwave vial was capped with an aluminum cap equipped with a PTFE septum. Then, the vial was brought outside the glovebox and stirred for 24 h at 35 °C. Afterwards, the reaction mixture was filtered through a silica plug and washed with CH_2_Cl_2_. The solvent was evaporated under reduced pressure, and the remaining residue was purified with preparatory thin layer chromatography or flash chromatography.

#### General procedure B (for linear selectivity)

To a 2–5 mL oven-dried microwave vial with a stir bar, allylpalladium chloride dimer (3.6 mg, 0.01 mmol, 0.05 equiv), 3,4,7,8-tetramethyl-1,10-phenanthroline (5.7 mg, 0.024 mmol, 0.12 equiv) were added inside a glovebox. 0.5 mL of dry THF was then added and the mixture was stirred for 1 h. Then, lithium *tert*-butoxide (40 mg, 0.5 mmol, 2.5 equiv), iodoarene (0.2 mmol), allene (0.35 mmol, 1.75 equiv) and hydrazone (0.4 mmol, 2.0 equiv) were added sequentially and an additional 1 mL of dry THF was added. The microwave vial was capped with an aluminum cap with a PTFE septum. Then, the vial was brought outside the glovebox and stirred for 24 h at 65 °C. Afterwards, the reaction mixture was filtered through a silica plug and washed with CH_2_Cl_2_. The solvent was evaporated under reduced pressure, and the remaining residue was purified with preparatory thin layer chromatography or flash chromatography.

## Supplementary information

Supplementary Information

Transparent Peer Review file

## Source data

Source Data

## Data Availability

All experimental procedures, mechanistic investigations, computational data including all energies, free energies, crystallography data and characterization data of synthesized compounds are available in the Supplementary Information. Cartesian coordinates are in the source data provided with this paper. The supplemental crystallographic data for compound **5bb** reported in this study have been deposited at the Cambridge Crystallographic Data Centre (CCDC), under deposition number CCDC: 2485363. Data supporting the findings of this manuscript are also available from the corresponding author upon request. Source data are provided with this paper.
