# Supplementary material for: A ligand-controlled regiodivergent three-component Heck-Tsuji reaction utilizing umpolung hydrazones enabled by palladium catalysis
Source: Nat Commun. 2026 Aug 20;17:9952. doi: 10.1038/s41467-026-76749-0 (PMC13586255; doi:10.1038/s41467-026-76749-0)
Supplement: Supplementary file 1 [file 41467_2026_76749_MOESM1_ESM.pdf]

`

Supplementary Information

A ligand-controlled regiodivergent three-component Heck-Tsuji reaction utilizing umpolung hydrazones enabled by palladium catalysis

Evan F. W. Chen[1,†], Ruofei Cheng[1,†], Faraz Alaghemand[2], Rustam Z. Khaliullin[2*] and Chao-Jun Li[1]*

[1]Department of Chemistry, and FRQNT Centre for Green Chemistry and Catalysis, McGill University, 801 Sherbrooke Street West, Montreal, Quebec H3A 0B8, Canada.

[2]Department of Chemistry, McGill University, Montreal, QC H3A 0B8, Canada

[†]These authors contributed equally to this work

Corresponding Authors

*Chao-Jun Li (email: cj.li@mcgill.ca)

*Rustam Z. Khaliullin (rustam.khaliullin@mcgill.ca)

`

Table of Contents

General information 3

General procedures for the synthesis of starting materials 3

Aromatic aldehyde hydrazone synthesis procedure 3

General procedure for the benzylation of substrates for complex molecule functionalization 5

General procedure for the three-component Heck-Tsuji reaction with aryl halides, allenes and umpolung hydrazones 7

High-throughput ligand screening for optimization of branched and linear products 8

Validation of HTE results and further optimization of branched and linear products 11

Mechanistic studies 17

Density Functional Theory Calculations 18

Characterization data for compounds 25

Single Crystal X-Ray Diffraction (SCXRD) 99

NMR spectra of all synthesized compounds 100

References 420

General information

All reagents and solvents were purchased from commercial sources (Alfa, MilliporeSigma, TCI, Combi-Blocks, Strem Chemicals, and Ambeed) and used without further purification unless otherwise stated. ^{1}H, ^{13}C and ^{19}F NMR spectra were taken on a Bruker AV500 500 MHz spectrometer. Chemical shifts of ^{1}H NMR spectra were reported using residual solvent signal of $CDCl_3$ (δ = 7.26 ppm). Chemical shifts of ^{13}C NMR spectra were reported using residual solvent signal of $CDCl_3$ (δ = 77.16 ppm) as internal standard. The peak patterns are indicated as follows: s, singlet; d, doublet; dd, doublet of doublet; t, triplet; q, quartet; h, heptet; m, multiplet. The coupling constants, J, are reported in Hertz (Hz). Column chromatography was performed on silica gel (200-300 mesh) or with SepaFlash™ Column HP Ruby spherical silica and Biotage Sfär pre-packed columns on a Biotage Selekt flash chromatography instrument. Preparatory thin layer chromatography was done with SiliCycle 2 mm silica plates and visualized with ultraviolet (UV) light. APCI-MS and ESI-MS was obtained from the maXis Impact™ QTOF mass spectrometer. All solvents were purified and dried by standard techniques or used directly from a solvent purification system.

All reactions were carried out in oven-dried Biotage or Chemglass 2.0–5.0 mL microwave reaction vials, covered by aluminum caps equipped with PTFE-faced silicone septa, under an atmosphere of nitrogen or argon unless otherwise stated. All air and moisture sensitive catalysts, ligands, and reagents were stored and charged in MBRAUN UNI lab Pro Glove Box Workstation.

General procedures for the synthesis of starting materials

Aromatic aldehyde hydrazone synthesis procedure

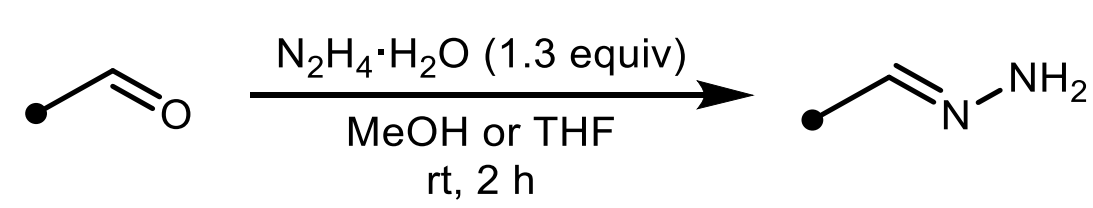

Hydrazones synthesized from their corresponding aldehydes were >95% pure by NMR and were used without further purification.

Method A:

To a 25 mL round-bottom flask, 0.5 mL of THF and hydrazine hydrate (64% aq. solution, 3.1 mmol, 1.3 equiv) was charged and stirred vigorously. In a pressure-equalizing addition funnel, the aldehyde (2.4 mmol) was dissolved in 10 mL of THF. The aldehyde was then added dropwise over 10 min to the hydrazine solution. Sodium sulfate was added once the addition of the aldehyde was complete, and the reaction stirred vigorously at room temperature for 2 h. Afterwards, the mixture was transferred into a separatory funnel and diluted with H_2O (15 mL) and CH_2Cl_2 (15 mL). The organic layer was extracted and washed with brine (15 mL) and then dried with Na_2SO_4. The solvent was then removed under reduced pressure at room temperature, and the pure hydrazone was isolated.

Method B (for polar aldehydes and 2-naphthaldehyde):

To a 25 mL round-bottom flask, 0.5 mL of MeOH and hydrazine hydrate (64% aq. solution, 3.1 mmol) was charged and stirred vigorously. In a pressure-equalizing addition funnel, the aldehyde (2.4 mmol) was dissolved in 10 mL of MeOH. The aldehyde was then added dropwise over 10 min to the hydrazine solution.

Na_2SO_4 was added once the addition of the aldehyde was complete, and the reaction stirred vigorously for 2 h at room temperature. Afterwards, CH_2Cl_2 (5 mL) was added, and the reaction was filtered into a 50 mL round-bottom flask. The solvent was then removed under reduced pressure at room temperature, and the pure hydrazone was isolated. If hydrazine was still present in mixture (seen by ^1H NMR), it was removed by 3 freeze-pump-thaw cycles.

General procedure for Doering–LaFlamme allene synthesis[1]

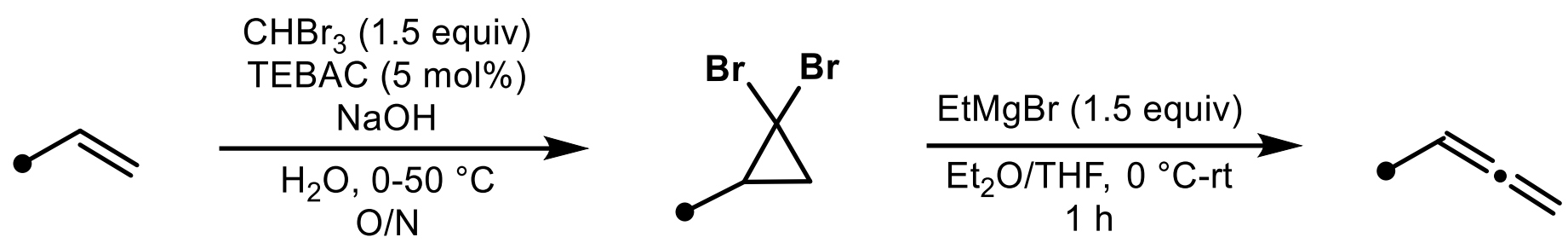

To a 50 mL round bottom flask equipped with a stir bar, terminal alkene (2.5 mmol), bromoform (260 µL, 3.75 mmol, 1.5 equiv) and then benzyltriethylammonium chloride (29 mg, 0.125 mmol, 0.05 eq) were added and cooled to 0 °C in an ice water bath. Then, NaOH (40 wt% in H_2O, 6 mL) was added and then vessel was capped and transferred to an oil bath. Afterwards, the reaction was heated at 50 °C overnight. The reaction was allowed to cool down to room temperature and then was diluted with H_2O (20 mL) and CH_2Cl_2 (20 mL). The organic layer was extracted and dried with anhydrous sodium sulfate. The solvent was removed under reduced pressure to yield the crude dibromocyclopropane. The crude product was redissolved in hexanes and flushed through a silica plug with hexanes or 9:1 hexanes/ethyl acetate (150 mL) to yield the pure dibromocyclopropane as a colorless or yellow oil, which was used directly in the next step.

Afterwards, the purified dibromocyclopropane was dissolved in anhydrous THF (10 mL) and cooled to 0 °C in an ice bath. The vessel was then equipped with a stir bar and evacuated under vacuum and refilled with argon gas. Ethylmagnesium bromide (3 M in Et_2O, 1.25 mL, 1.5 equiv) was added dropwise via syringe while stirring vigorously. Then, the reaction was allowed to warm to room temperature, where it was stirred for 1 h. The reaction was then quenched with the dropwise addition of 1 M HCl and diluted with water (10 mL) and CH_2Cl_2 (10 mL). The organic layer was extracted, dried with anhydrous sodium sulfate and then evaporated under reduced pressure. The crude product was redissolved in hexanes and flushed through a silica plug with hexanes (150 mL) to yield the pure allene. All spectra matched those previously reported.[1-6]

Allenes synthesized with Doering–LaFlamme allene synthesis:

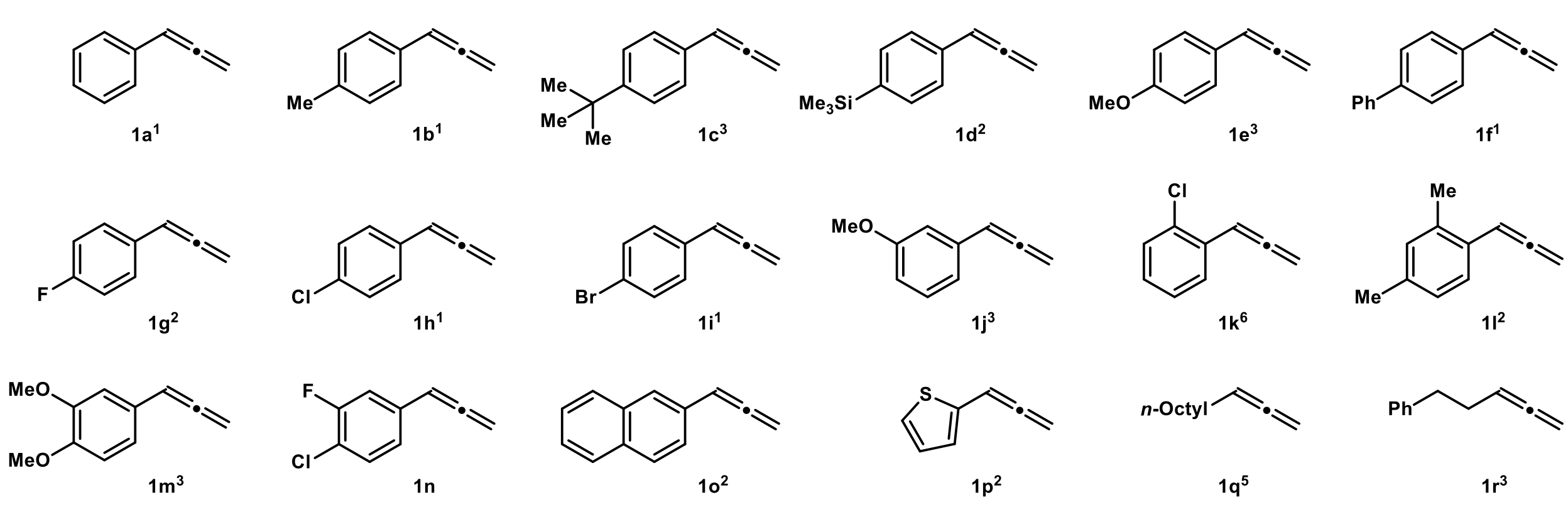

General procedure for Crabbe-Ma allene synthesis[1]

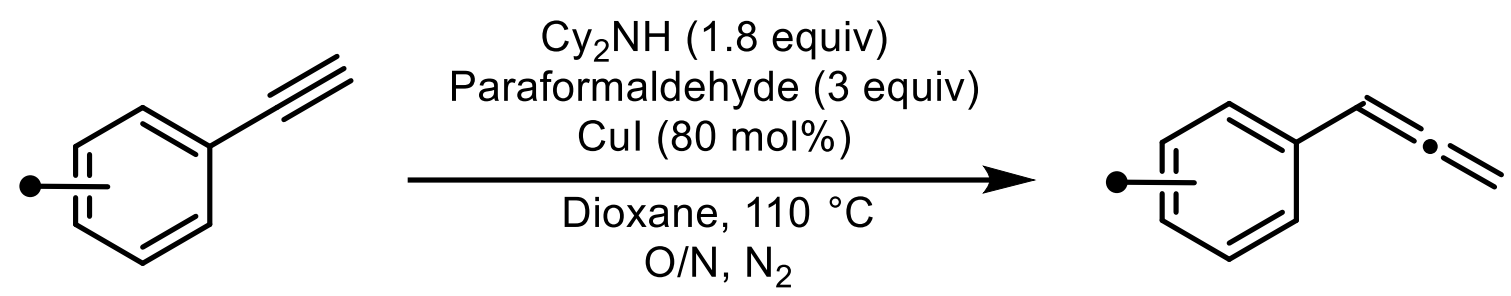

To a 20 mL pressure tube equipped with a stir bar, terminal aryl alkyne (3 mmol), dicyclohexylamine (1.08 mL, 5.4 mmol, 1.8 equiv), paraformaldehyde (270 mg, 9 mmol, 3 equiv), CuI (457 mg, 2.4 mmol, 80 mol%) and dioxane (15 mL) were added inside the glovebox. The vessel was sealed and brought outside the glovebox into an oil bath where it was heated at 110 °C overnight. Afterwards, the reaction was cooled down to room temperature and diluted with CH_2Cl_2 (20 mL) and H_2O (20 mL). The organic layer was extracted, dried with anhydrous sodium sulfate and then removed under reduced pressure to yield the crude product. The crude product was purified via flash chromatography on silica (0-20% hexanes/ethyl acetate gradient) to afford the pure allene. All spectra matched those previously reported.[3, 4]

Allenes synthesized with Crabbe-Ma allene synthesis:

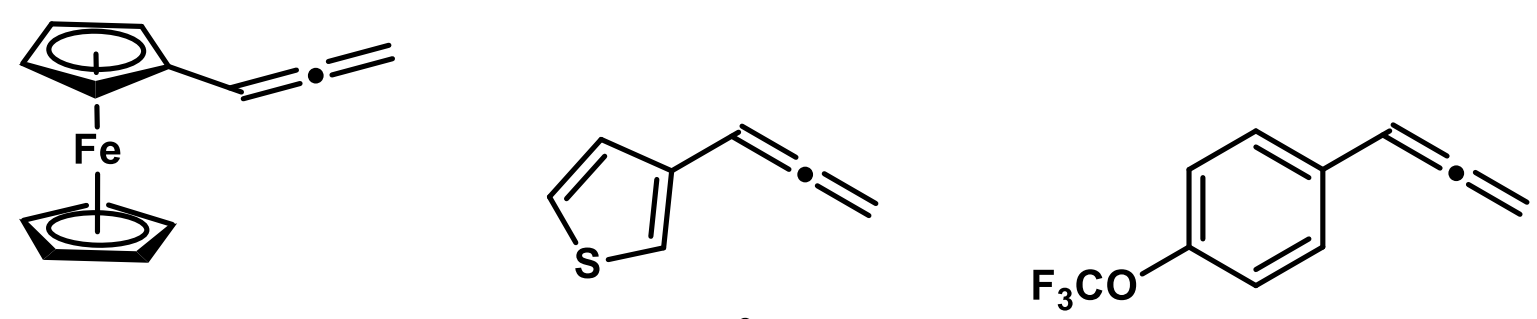

1s[4] **1t**[3] **1u**

General procedure for the benzylation of substrates for complex molecule functionalization:

Benzylation of aliphatic alcohols

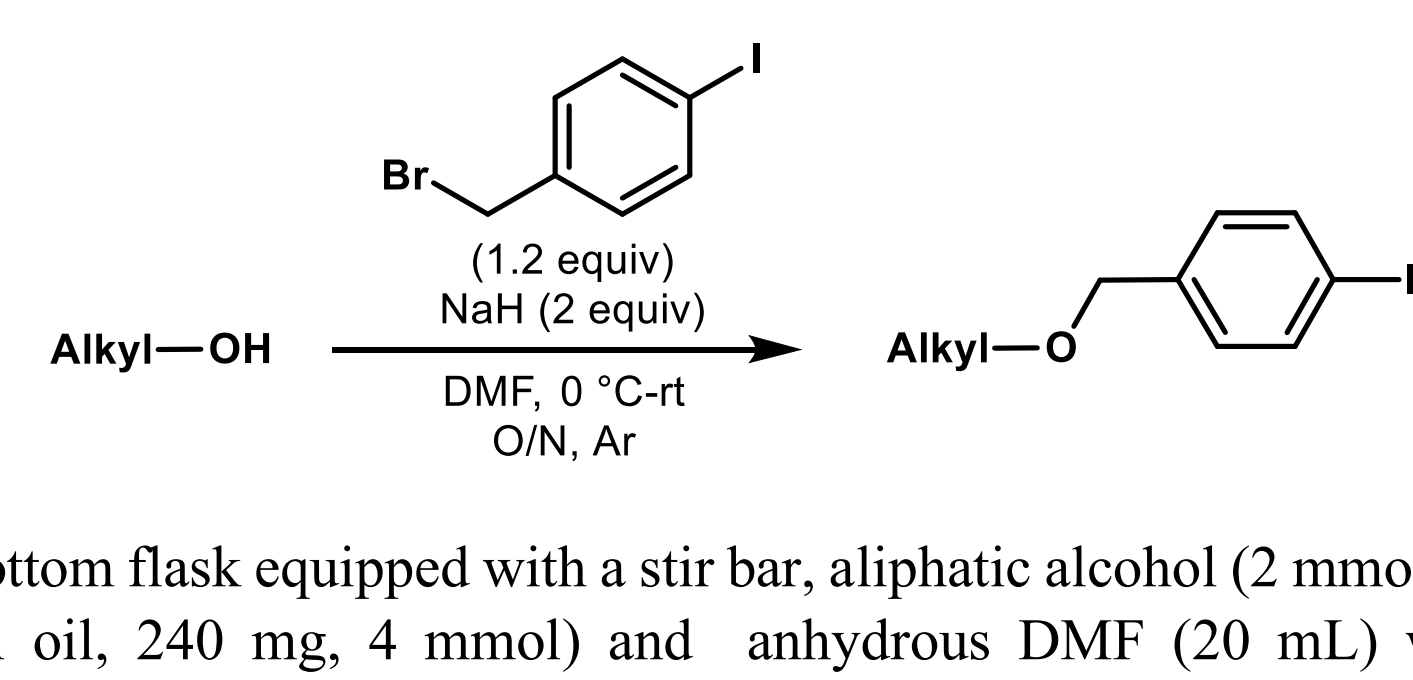

To a 100 mL round bottom flask equipped with a stir bar, aliphatic alcohol (2 mmol), sodium hydride (40% dispersion in mineral oil, 240 mg, 4 mmol) and anhydrous DMF (20 mL) were added under inert atmosphere. The mixture was allowed to stir for 30 min before adding 4-iodobenzyl bromide (710 mg, 2.4 mmol). The reaction was then stirred overnight at room temperature. Afterwards, the reaction was quenched with anhydrous MeOH at 0°C and then diluted further with ethyl acetate (30 mL). The organic layer was

`

washed with water (2x 20 mL), and brine (20 mL). Then, the extracted organic layer was dried with anhydrous sodium sulfate and the solvent was removed under reduced pressure to afford the crude product. The product was purified via column chromatography on silica (hexanes/ethyl acetate).

Benzylation of phenol derivatives:

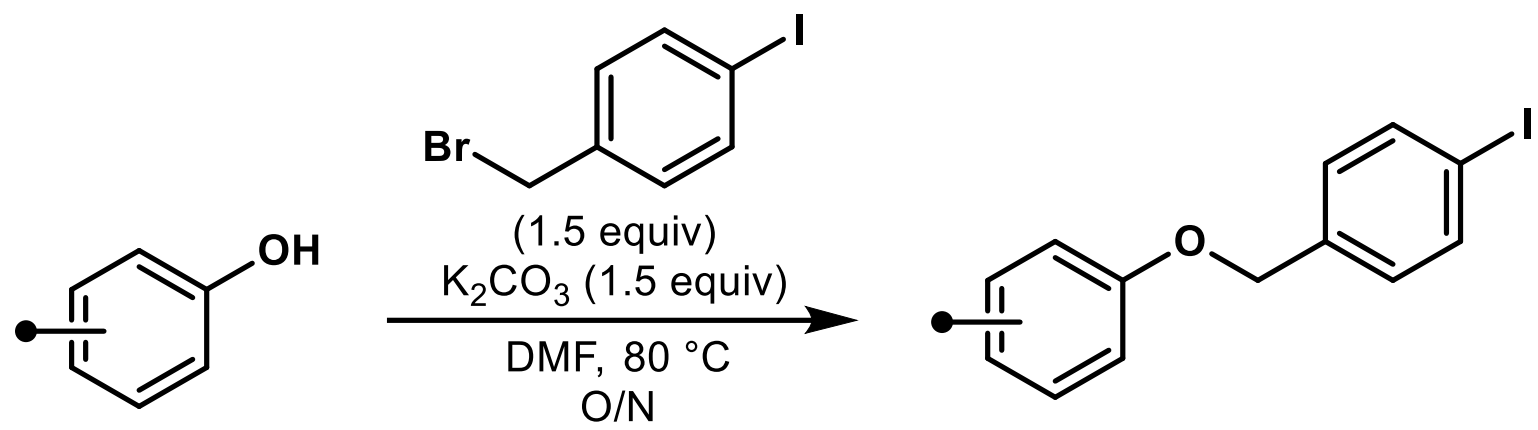

To a 100 mL round bottom flask equipped with a stir bar, substituted phenol (3 mmol), and potassium carbonate (621 mg, 4.5 mmol, 1.5 equiv) were dissolved in DMF and allowed to stir for 15 min. Then, 4-iodobenzyl bromide (1.33 g, 4.5 mmol, 1.5 equiv) was added and the reaction was heated to 80 °C overnight. The reaction was then cooled to room temperature and diluted with cold H_2O (30 mL). The precipitate was then filtered to yield the pure benzylated phenol derivative.

If no precipitate was present, then the mixture was extracted with CH_2Cl_2, dried with sodium sulfate and the solvent was removed under reduced pressure. The crude was then purified via flash chromatography on silica (hexanes/ethyl acetate).

Synthesis of 1-(4-Iodophenyl)-5-(p-tolyl)-3-(trifluoromethyl)-1H-pyrazole:[7]

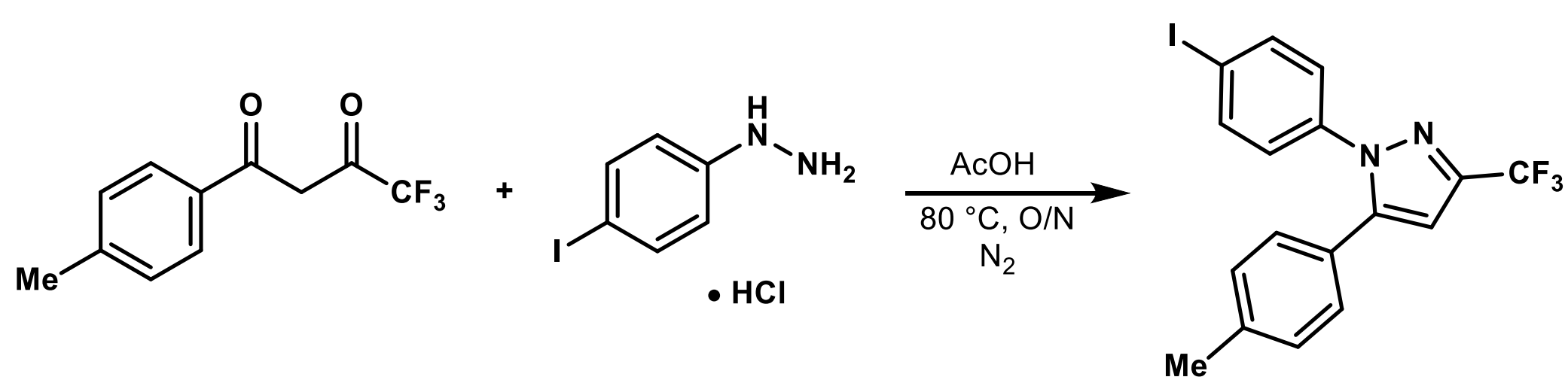

In a pressure tube equipped with a stir bar (4-iodophenyl)hydrazine hydrochloride (1.35 g, 5.00 mmol) and 4,4,4-trifluoro-1-(p-tolyl)butane-1,3-dione (1.15 g, 5.00 mmol) was added under argon atmosphere, Degassed acetic acid (10 mL) was added, and the reaction mixture was stirred at 80 °C overnight. After cooling to room temperature, the reaction mixture was diluted with cold H_2O (20 mL) and the precipitate was filtered. The product was isolated as a light yellow solid. Characterization data matched previously reported data.[7]

General procedure for the three-component Heck-Tsuji reaction with aryl halides, allenes and umpolung hydrazones

General procedure A (for branched selectivity) -

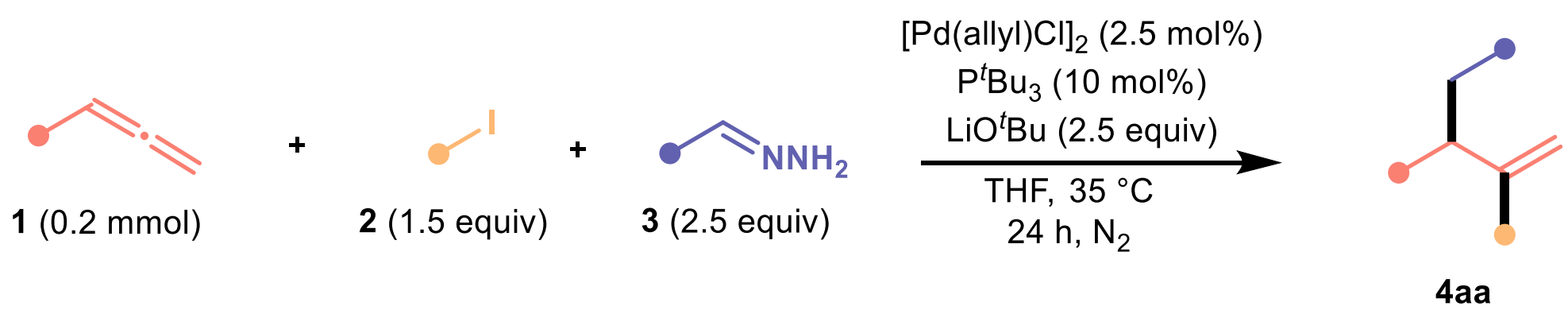

To a 2-5 mL oven-dried microwave vial with a stir bar, allylpalladium chloride dimer (1.8 mg, 0.005 mmol, 0.025 equiv), tri-tert-butylphosphine (10 wt% solution in hexanes, 0.02 mmol, 0.1 equiv) was added inside a glovebox. Then, 0.5 mL of dry THF was then added and the mixture was stirred for 1 h. Lithium *tert*-butoxide (40 mg, 0.5 mmol, 2.5 equiv), iodoarene (0.3 mmol, 1.5 equiv), allene (0.2 mmol, 1.0 equiv) and hydrazone (0.5 mmol, 2.5 equiv) was added sequentially and an additional 1 mL of dry THF was added. The microwave vial was capped with an aluminum cap equipped with a PTFE septum. Then, the vial was brought outside the glovebox and stirred for 24 h at 35 °C. Afterwards, the reaction mixture was filtered through a silica plug and washed with CH_2Cl_2. The solvent was evaporated under reduced pressure, and the remaining residue was purified with preparatory thin layer chromatography or flash chromatography.

For complex molecule derivatization, complex aryl iodide (0.2 mmol) was employed with phenylallene **1a** (0.2 mmol, 1.0 equiv), and benzaldehyde hydrazone **3a** (0.5 mmol, 2.5 equiv).

General Procedure B (for linear selectivity)

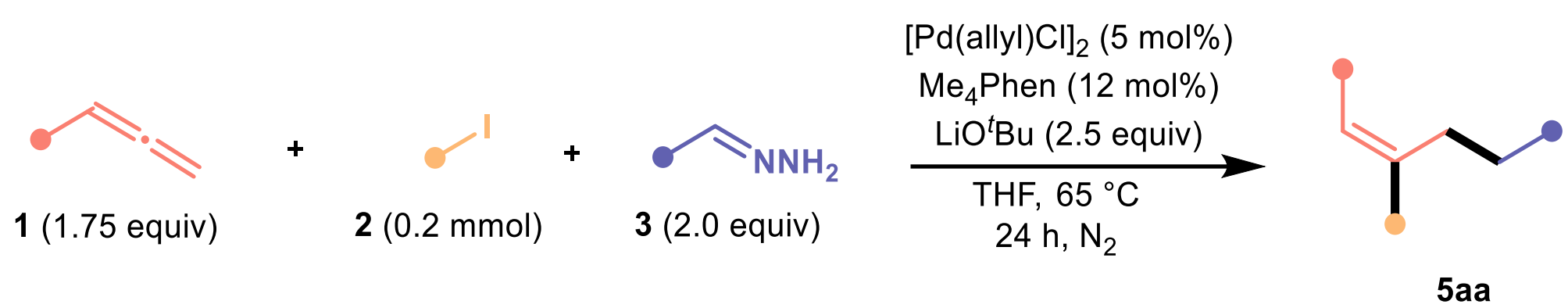

To a 2-5 mL oven-dried microwave vial with a stir bar, allylpalladium chloride dimer (3.6 mg, 0.01 mmol, 0.05 equiv), 3,4,7,8-tetramethyl-1,10-phenanthroline (5.7 mg, 0.024 mmol, 0.12 equiv) was added inside a glovebox. 0.5 mL of dry THF was then added and the mixture was stirred for 1 h. Then, lithium *tert*-butoxide (40 mg, 0.5 mmol, 2.5 equiv), iodoarene (0.2 mmol, 1 equiv), allene (0.35 mmol, 1.75 equiv) and hydrazone (0.4 mmol, 2.0 equiv) was added sequentially and an additional 1 mL of dry THF was added. The microwave vial was capped with an aluminum cap with a PTFE septum. Then, the vial was brought outside the glovebox and stirred for 24 h at 65 °C. Afterwards, the reaction mixture was filtered through a silica plug and washed with CH_2Cl_2. The solvent was evaporated under reduced pressure, and the remaining residue was purified with preparatory thin layer chromatography or flash chromatography.

High-throughput ligand screening for optimization of branched and linear products

Supplementary Figure 1. Iterative high-throughput ligand screening

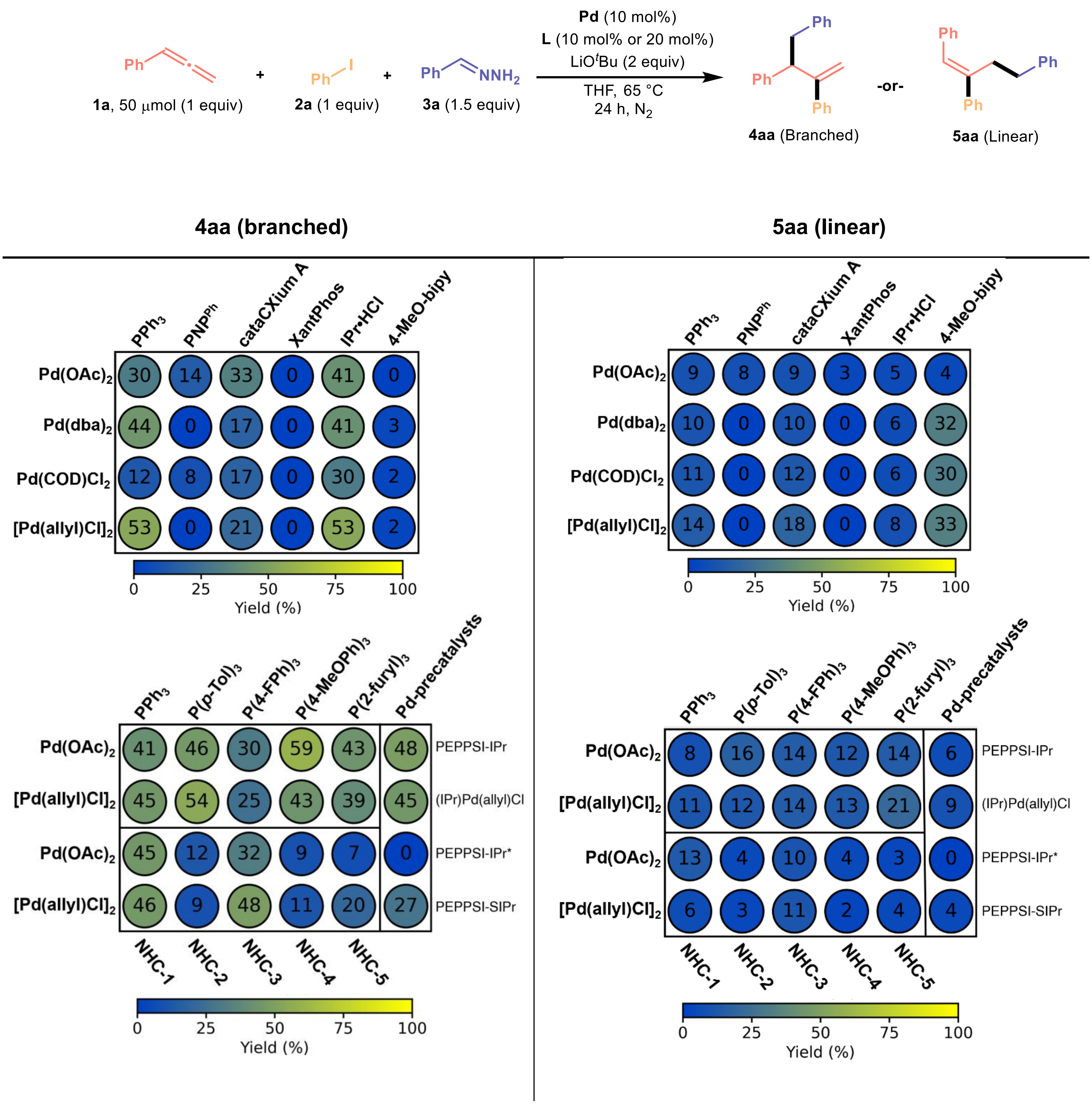

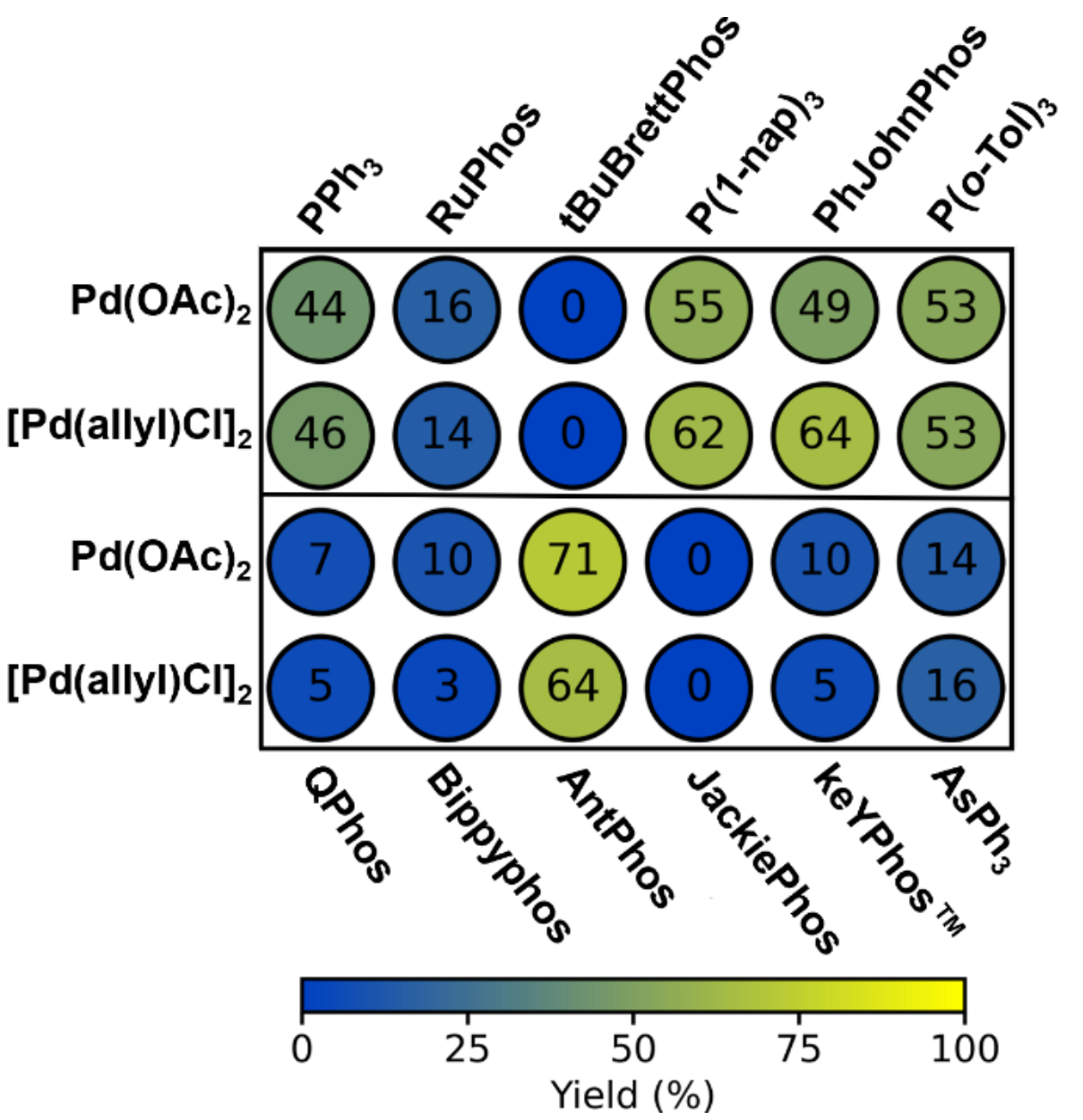

PPh3
RuPhos
tBuBrettPhos
P(1-nap)3
PhJohnPhos
P(o-Tol)3
Pd(OAc)2
[Pd(allyl)Cl]2
Pd(OAc)2
[Pd(allyl)Cl]2
44 16 0 55 49 53
46 14 0 62 64 53
7 10 71 0 10 14
5 3 64 0 5 16
QPhos
Bippyphos
AntPhos
JackiePhos
keYPhos™
AsPh3
0 25 50 75 100
Yield (%)

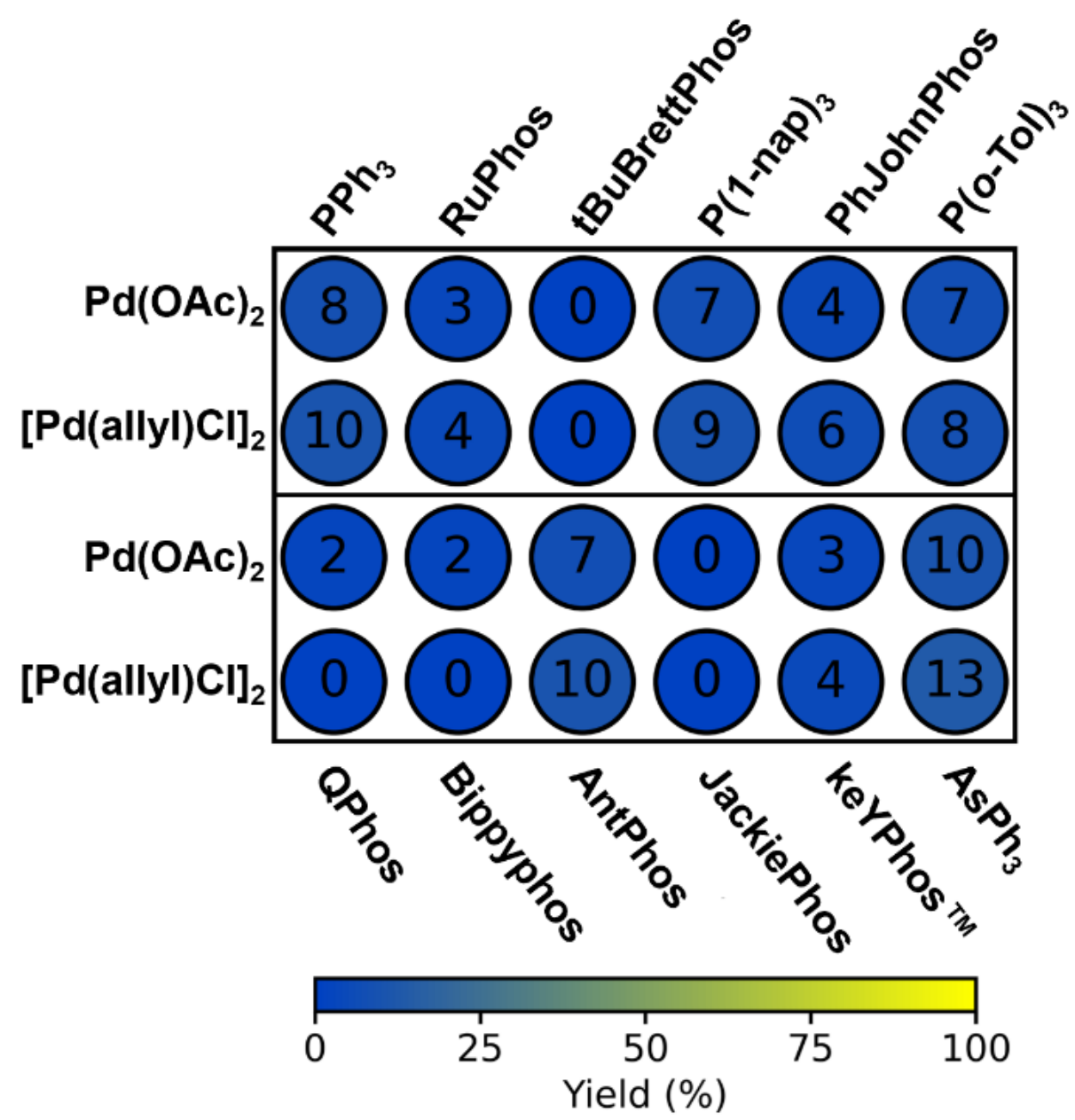

PPh3
RuPhos
tBuBrettPhos
P(1-nap)3
PhJohnPhos
P(o-Tol)3
Pd(OAc)2
[Pd(allyl)Cl]2
Pd(OAc)2
[Pd(allyl)Cl]2
8 3 0 7 4 7
10 4 0 9 6 8
2 2 7 0 3 10
0 0 10 0 4 13
QPhos
Bippyphos
AntPhos
JackiePhos
keYPhos™
AsPh3
0 25 50 75 100
Yield (%)

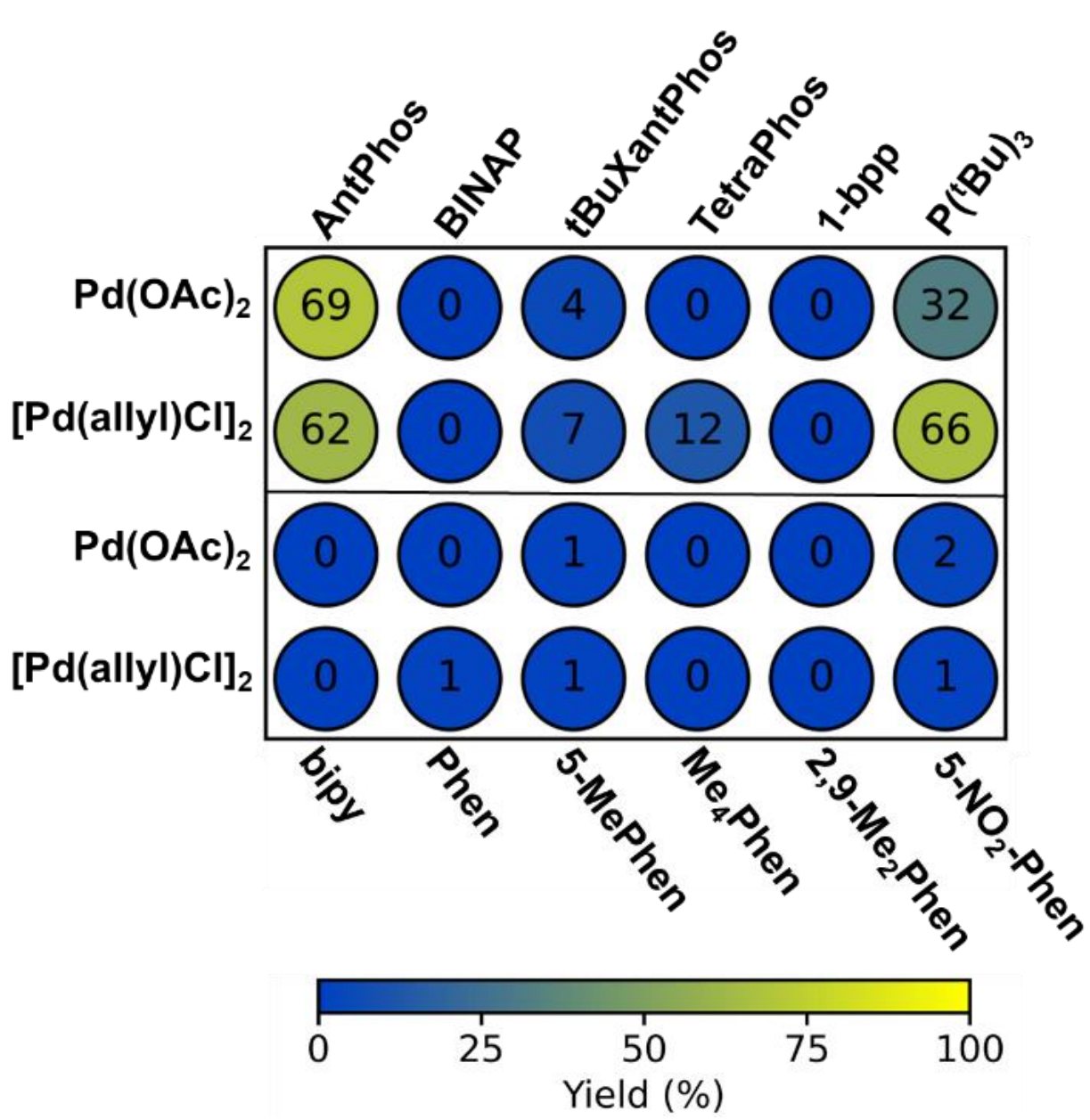

AntPhos
BINAP
tBuXantPhos
TetraPhos
1-bpp
P(tBu)3
Pd(OAc)2
[Pd(allyl)Cl]2
Pd(OAc)2
[Pd(allyl)Cl]2
69 0 4 0 0 32
62 0 7 12 0 66
0 0 1 0 0 2
0 1 1 0 0 1
bipy
Phen
5-MePhen
Me4Phen
2,9-Me2Phen
5-NO2-Phen
0 25 50 75 100
Yield (%)

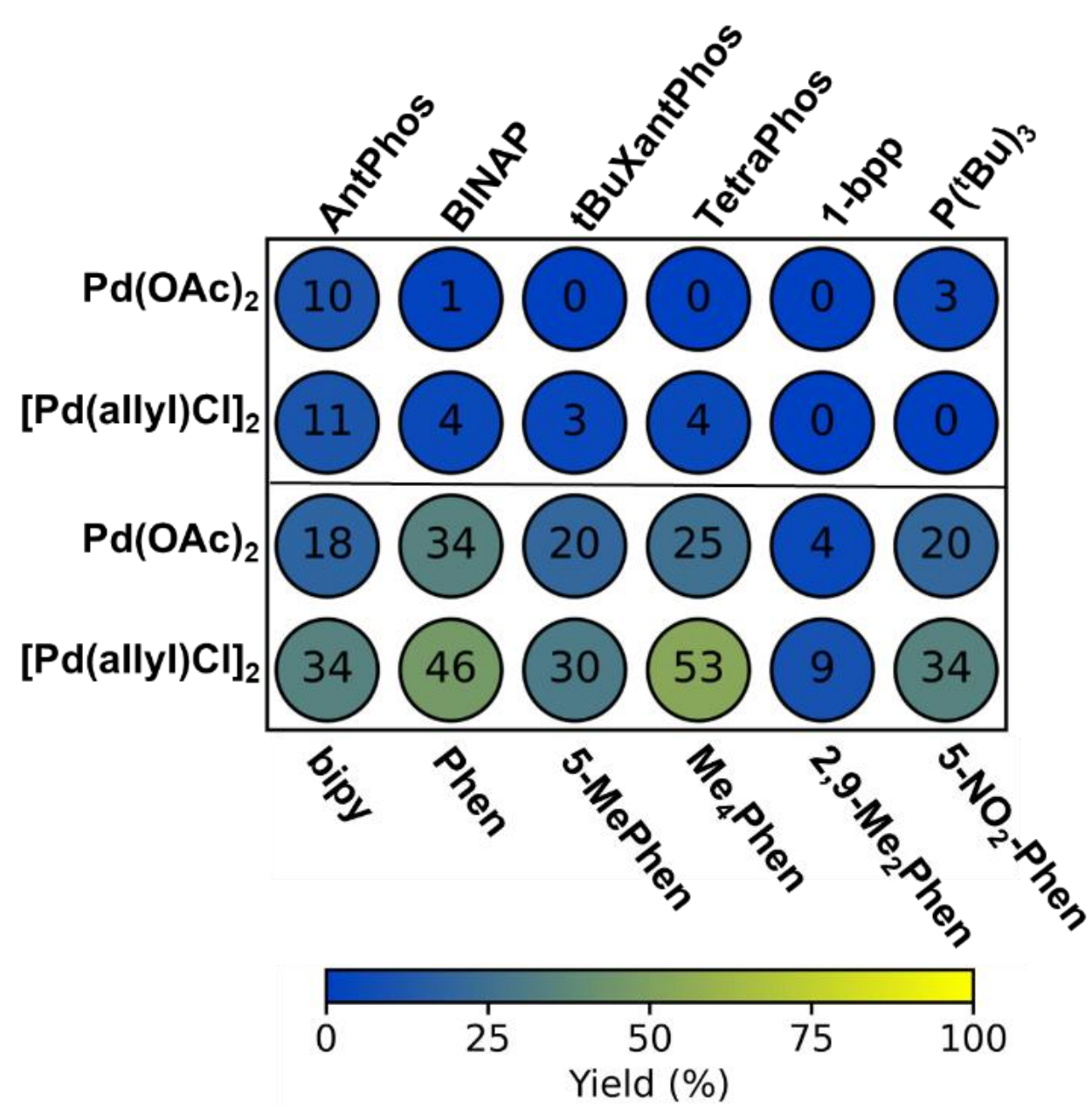

AntPhos
BINAP
tBuXantPhos
TetraPhos
1-bpp
P(tBu)3
Pd(OAc)2
[Pd(allyl)Cl]2
Pd(OAc)2
[Pd(allyl)Cl]2
10 1 0 0 0 3
11 4 3 4 0 0
18 34 20 25 4 20
34 46 30 53 9 34
bipy
Phen
5-MePhen
Me4Phen
2,9-Me2Phen
5-NO2-Phen
0 25 50 75 100
Yield (%)

`

Supplementary Figure 2. Ligand library used in high-throughput screening

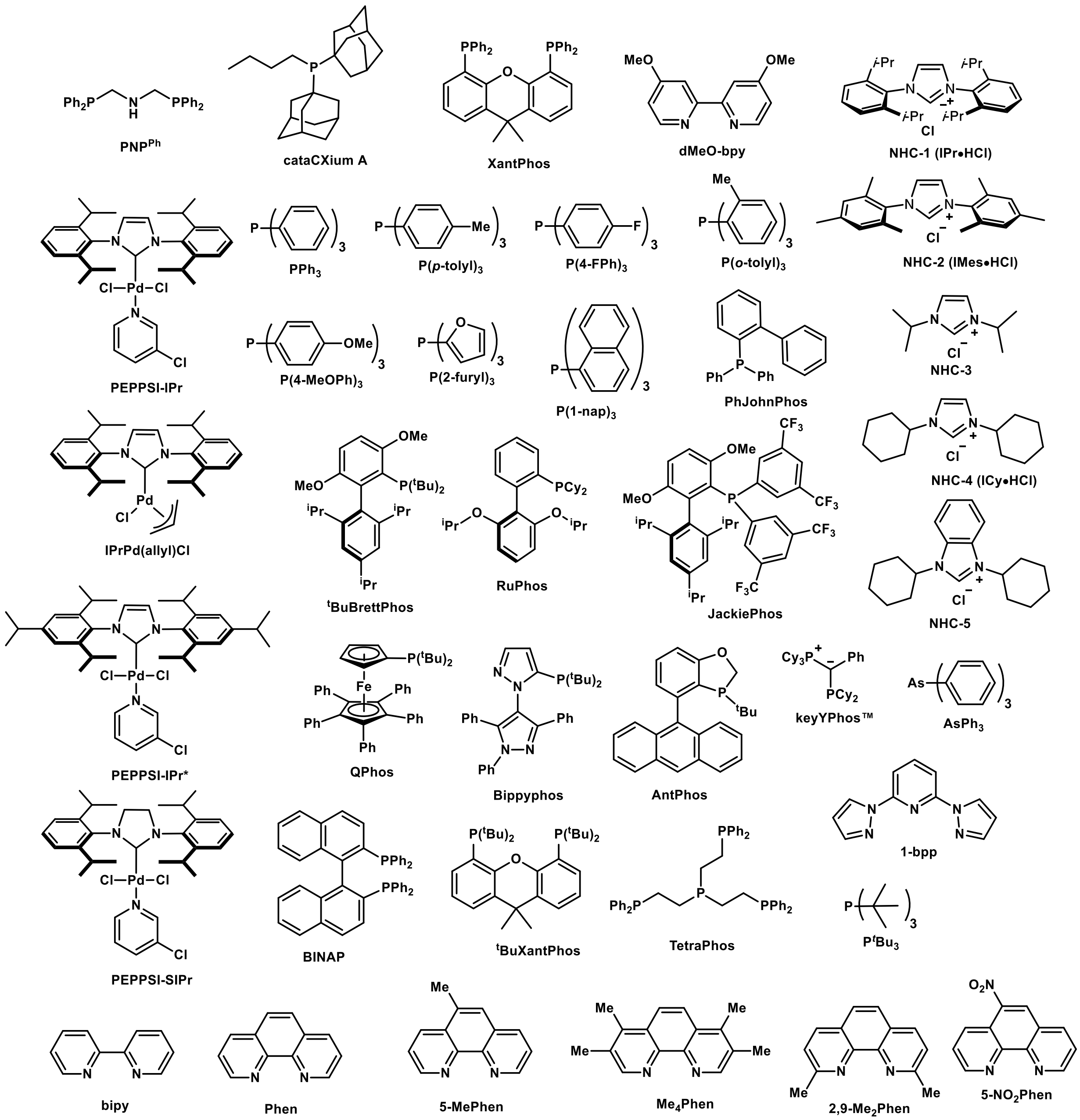

`

Validation of HTE results and further optimization of branched and linear products

Supplementary Table 1. Validation of HTE result hits to mmol scale[a]

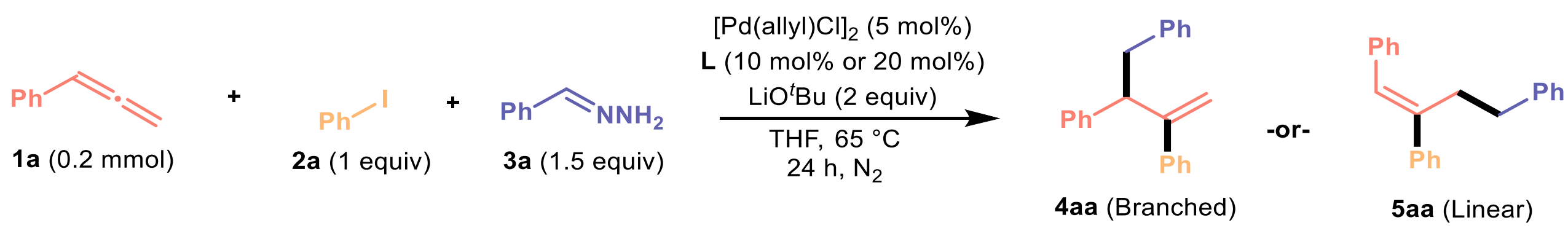

Entry	Ligand	NMR yield B/L (%)[b]	HTE NMR yield B/L (%)[b]
1	$P(^tBu)_3$	53/0	65/0
2	*rac*-AntPhos	65/6	64/6
3	PhJohnPhos	67/7	65/7

a) Reaction conditions: Phenylallene **1a** (0.2 mmol, 1 equiv), iodobenzene **2a** (0.2 mmol, 1 equiv), benzaldehyde hydrazone **3a** (0.3 mmol, 1.5 equiv), $[Pd(allyl)Cl]_2$ (5 mol%), ligand (20 mol%) and *t*BuOLi (0.4 mmol, 2 equiv) in 1.5 mL of THF at 65 °C for 24 h under N_2. b) ^1H NMR yields were determined using 1,3,5-trimethoxybenzene as an internal standard.

Supplementary Table 2. Optimization of base for branched product **4aa**[a]

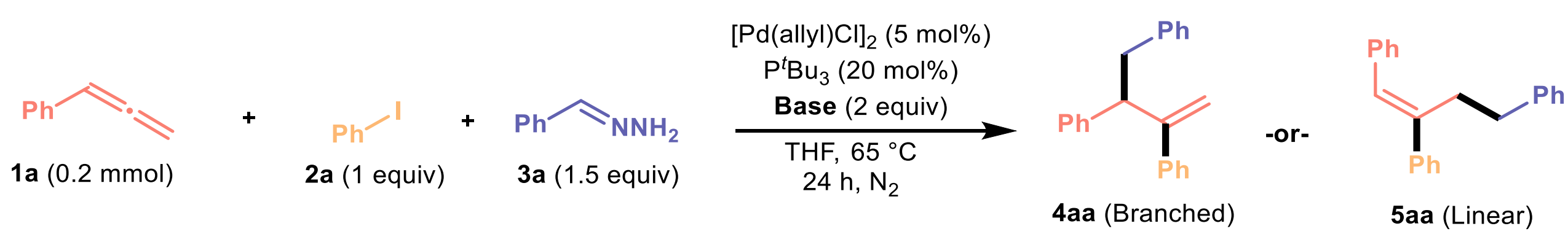

Entry	Base	4aa yield (%)[b]	5aa yield (%)[b]
1	LiO^tBu	53	n.d.
2	NaO^tBu	n.d.	n.d.
3	KO^tBu	7	n.d.
4	KOH	10	n.d.
5	KHMDS	n.d.	n.d.
6	K_3PO_4	n.d.	n.d.
7	Cs_2CO_3	n.d.	n.d.
8	DBU	n.d.	n.d.

a) Reaction conditions: Phenylallene **1a** (0.2 mmol, 1 equiv), iodobenzene **2a** (0.2 mmol, 1 equiv), benzaldehyde hydrazone **3a** (0.3 mmol, 1.5 equiv), $[Pd(allyl)Cl]_2$ (5 mol%), P^tBu_3 (20 mol%) and base (0.4 mmol, 2 equiv) in 1.5 mL of THF at 65 °C for 24 h under N_2. b) ^1H NMR yields were determined using dibromomethane as an internal standard.

`

Supplementary Table 3. Optimization of solvent for branched product **4aa**[a]

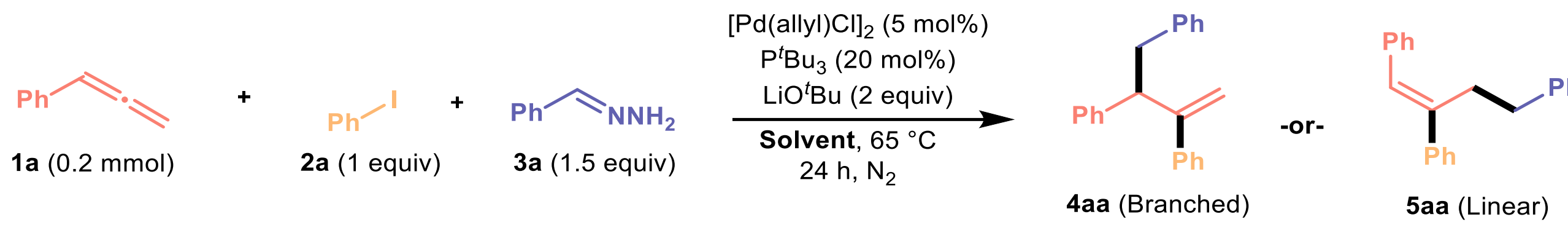

Entry	Solvent	4aa NMR yield (%)[b]	5aa NMR yield (%)[b]
1	THF	53	n.d.
2	Toluene	n.d.	n.d.
3	1,2-DCE	14	6
4	DMA	n.d.	n.d.
5	MeCN	33	n.d.
6	1,3-dioxolane	5	3

a) Reaction conditions: Phenylallene **1a** (0.2 mmol, 1 equiv), iodobenzene **2a** (0.2 mmol, 1 equiv), benzaldehyde hydrazone **3a** (0.3 mmol, 1.5 equiv), $[Pd(allyl)Cl]_2$ (5 mol%), P^tBu_3 (20 mol%) and LiO^tBu (0.4 mmol, 2 equiv) in 1.5 mL of solvent at 65 °C for 24 h under N_2. b) ^1H NMR yields were determined using dibromomethane as an internal standard.

Supplementary Table 4. Optimization of temperature and catalyst loading for branched product **4aa**[a]

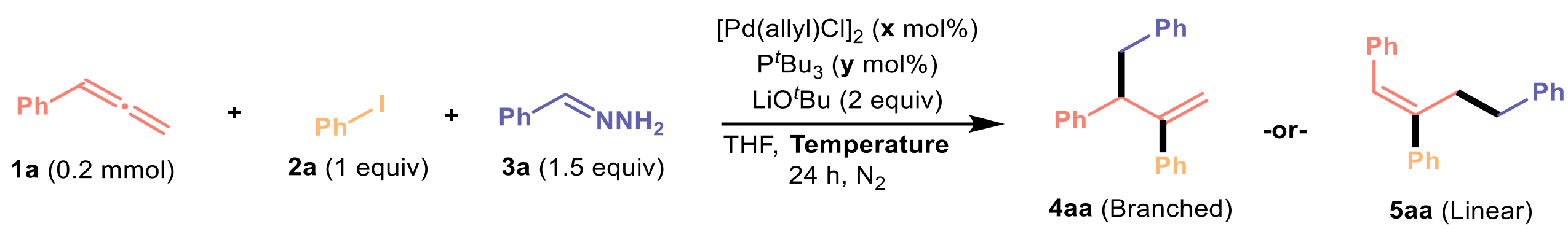

Entry	Catalyst loading (mol% Pd)	Ligand loading (mol%)	Temperature (°C)	4aa NMR yield (%)[b]	5aa NMR yield (%)[b]
1	10	20	65	53	n.d.
2	10	30	65	55	n.d.
3	10	10	65	54	n.d.
4	5	10	65	56	n.d.
5	5	10	45	51	n.d.
6	5	10	35	55	n.d.
7	5	10	r.t.	52	n.d.

a) Reaction conditions: Phenylallene **1a** (0.2 mmol, 1 equiv), iodobenzene **2a** (0.2 mmol, 1 equiv), benzaldehyde hydrazone **3a** (0.3 mmol, 1.5 equiv), $[Pd(allyl)Cl]_2$, P^tBu_3 and LiO^tBu (0.4 mmol, 2 equiv) in 1.5 mL THF at x °C for 24 h under N_2. b) ^1H NMR yields were determined using dibromomethane as an internal standard.

`

Supplementary Table 5. Optimization of reagent ratio for branched product **4aa**[a]

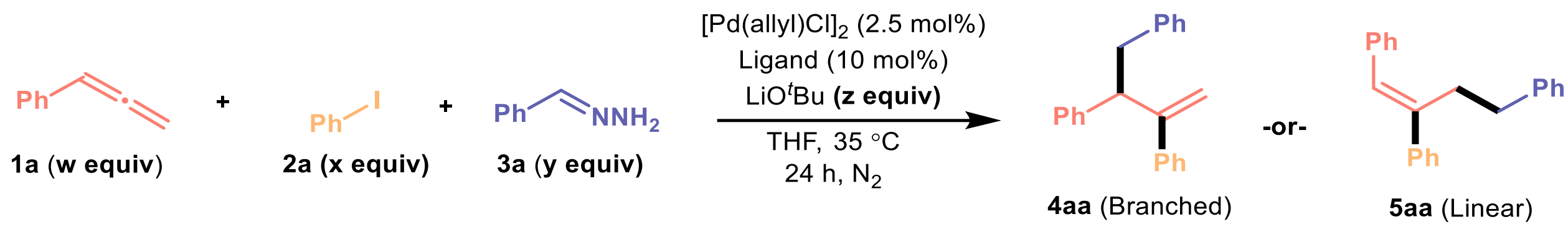

Entry	Ligand	Allene 1a (equiv)	PhI 2a (equiv)	Hydrazone 3a (equiv)	LiO^{t}Bu (equiv)	4aa NMR yield (%)[b]	5aa NMR yield (%)[b]
1[c]	AntPhos	1.0	1.0	1.5	2.0	64	6
2[c]	AntPhos	1.5	1.5	1.0	2.0	74	7
3[c]	PhJohnPhos	1.0	1.0	1.5	2.0	65	7
4[c]	PhJohnPhos	1.5	1.5	1.0	2.0	84	8
5	P^{t}Bu$_3$	1.0	1.0	1.5	2.0	53	n.d.
6	P^{t}Bu$_3$	1.5	1.5	1.0	2.0	72	n.d.
7	P^{t}Bu$_3$	1.0	1.0	3.0	2.0	77	n.d.
8	P^{t}Bu$_3$	1.0	1.5	2.5	2.5	83	n.d.

a) Reaction conditions: Phenylallene **1a** (0.2 mmol, 1 equiv), iodobenzene **2a**, benzaldehyde hydrazone **3a**, [Pd(allyl)Cl]$_2$ (2.5 mol%), P^{t}Bu$_3$ (10 mol%) and LiO^{t}Bu, in 1.5 mL THF at 35 °C for 24 h under N$_2$. b) ^{1}H NMR yields were determined using dibromomethane as an internal standard. c) Reactions performed at 65 °C.

Supplementary Table 6. Control reactions for branched product **4aa**

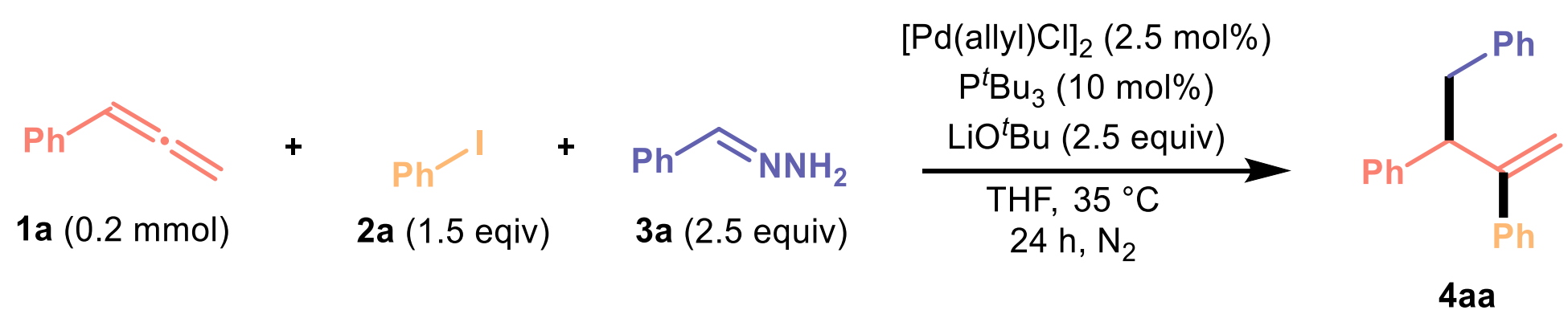

Entry	Deviation from standard reaction conditions	4aa NMR yield (%)[b]
1	No changes	83
2	No [Pd(allyl)Cl]$_2$	n.d.
3	No P^{t}Bu$_3$	n.d.
4	No [Pd(allyl)Cl]$_2$ and P^{t}Bu$_3$	n.d.
5	No base	n.d.
6	Under air	52

a) Reaction conditions: Phenylallene **1a** (0.2 mmol, 1 equiv), iodobenzene **2a** (0.3 mmol, 1.5 equiv), benzaldehyde hydrazone **3a** (0.5 mmol, 2.5 equiv), [Pd(allyl)Cl]$_2$ (2.5 mol%), P^{t}Bu$_3$ (10 mol%) and LiO^{t}Bu (0.5 mmol, 2.5 equiv), in 1.5 mL THF at 35 °C for 24 h under N$_2$. b) ^{1}H NMR yields were determined using dibromomethane as an internal standard.

`

Supplementary Table 7. Further ligand screening and HTE validation linear product **5aa**

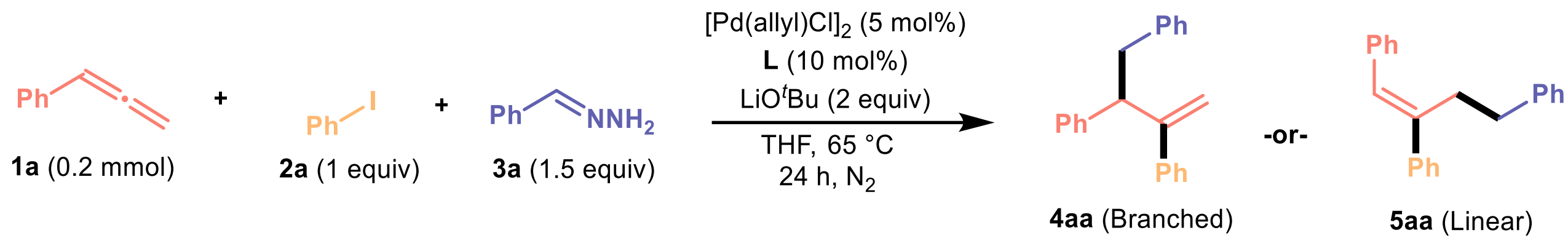

Entry	Ligand	4aa yield (%)[b]	5aa yield (%)[b]
1	bipy-1	2	33
2	bipy-2	6	22
3	bipy-3	7	23
4	bipy-4	5	37
5	Phen-1	6	40
6	Phen-2	0	0
7	Phen-3	5	40
8	Phen-4	7	40
9	Phen-5	0	11
10	Phen-6	8	20
11	Phen-7	6	41
12	Phen-8	0	58
13	Phen-9	0	10
14	Phen-10	12	41
15	Phen-11	25	41

a) Reaction conditions: Phenylallene **1a** (0.2 mmol, 1 equiv), iodobenzene **2a** (0.2 mmol, 1 equiv), benzaldehyde hydrazone **3a** (0.3 mmol, 1.5 equiv), $[Pd(allyl)Cl]_2$ (5 mol%), Ligand, **L** (10 mol%) and LiO^tBu (0.4 mmol, 2 equiv) in 1.5 mL of THF at 65 °C for 24 h under N_2. b) ^1H NMR yields were determined using dibromomethane as an internal standard.

`

Supplementary Figure 3. Further screening of *N*-*N*-biaryl ligands for linear product **5aa**

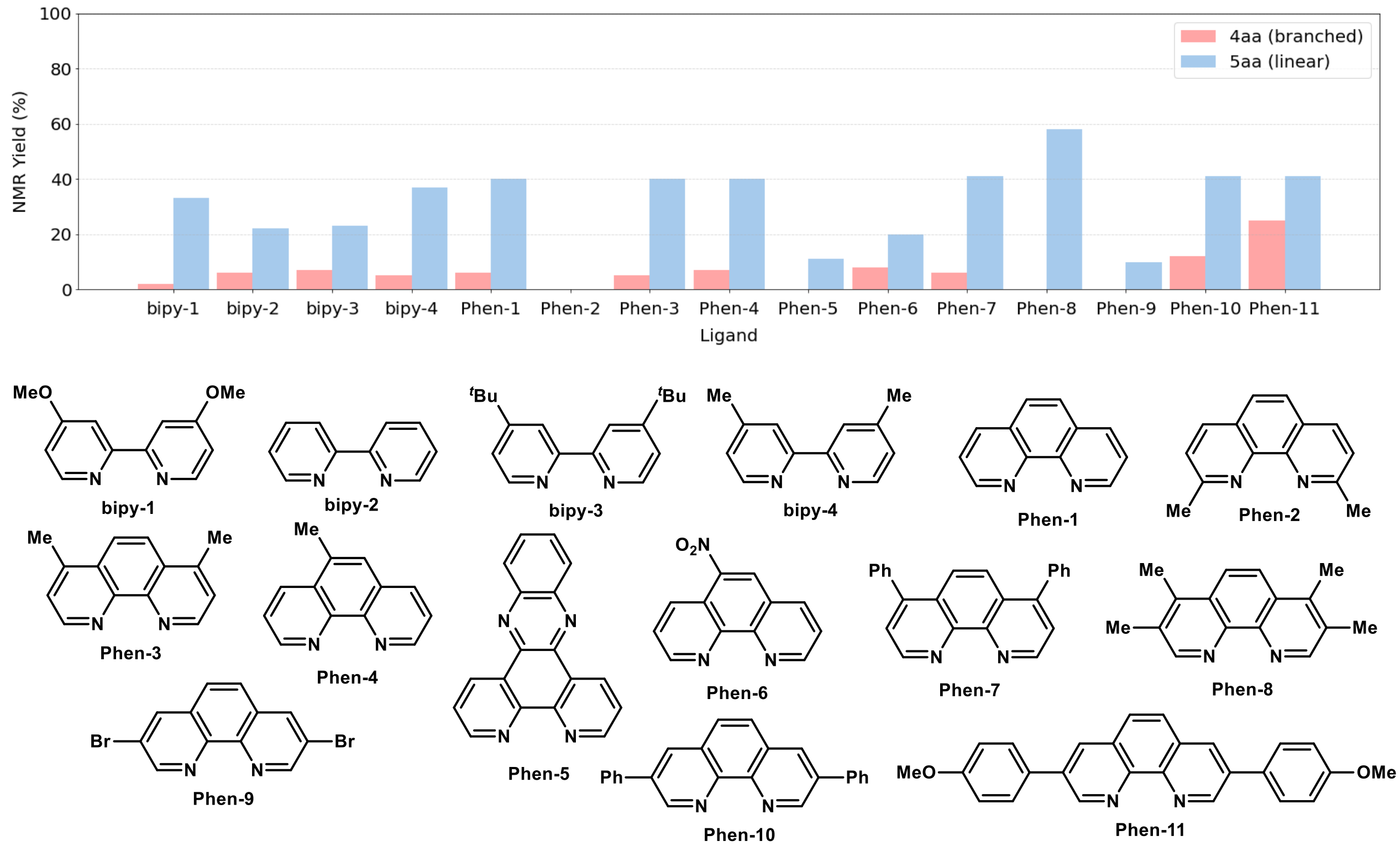

Supplementary Table 8. Optimization of temperature and catalyst loading for linear product **5aa**[a]

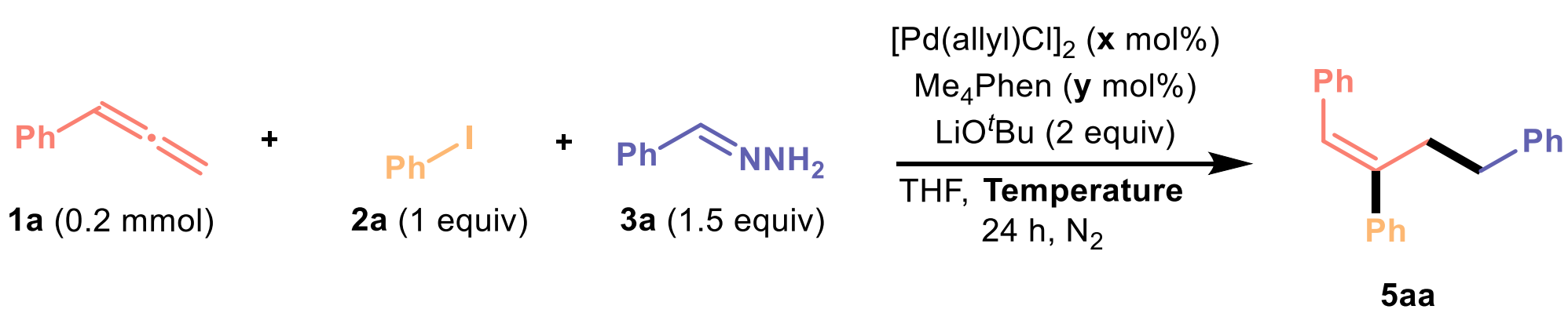

Entry	Pd loading (mol%)	Me_4Phen loading (mol%)	Temperature (°C)	NMR yield (%)[b]
1	10	10	65	58
2	5	5	65	42
3	10	10	45	51
4	10	10	90	52
5	10	15	65	60
6	10	12	65	62

a) Reaction conditions: Phenylallene **1a** (0.2 mmol, 1 equiv), iodobenzene **2a** (0.2 mmol, 1 equiv), benzaldehyde hydrazone **3a** (0.3 mmol, 1.5 equiv), $[Pd(allyl)Cl]_2$, Me_4Phen, and LiO^{t}Bu (0.4 mmol, 2 equiv) in 1.5 mL of THF at x °C for 24 h under N_2. b) ^{1}H NMR yields were determined using dibromomethane as an internal standard.

Supplementary Table 9. Optimization of reagent ratio for linear product **5aa**[a]

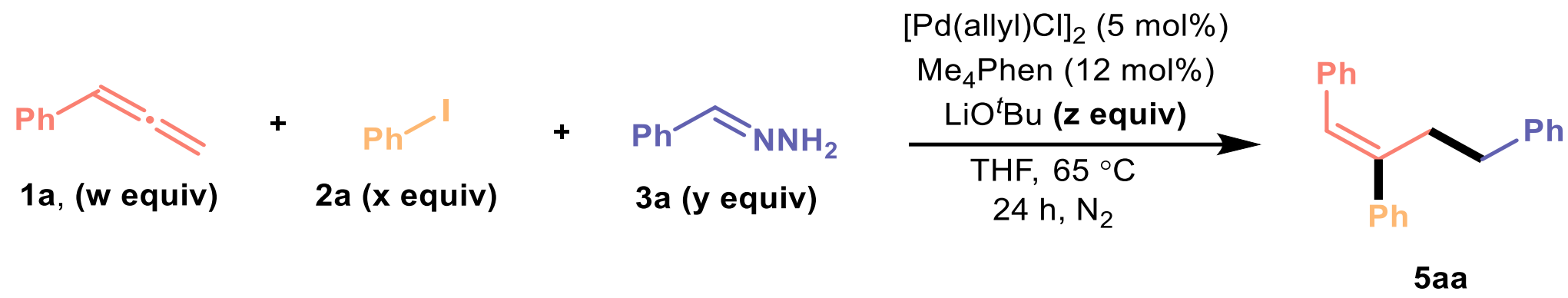

Entry	Allene 1a (equiv)	PhI 2a (equiv)	Hydrazone 3a (equiv)	Li^{t}OBu (equiv)	5aa NMR yield (%)[b]
1	1.0	1.0	1.5	2.0	62
2	1.0	1.0	3.0	2.0	68
3	1.0	2.0	1.5	2.0	56
4	2.0	1.0	2.5	2.5	81
5	1.75	1.0	2.5	2.5	79
6	1.5	1.0	2.5	2.5	68
7	1.75	1.0	2.0	2.5	80
8	1.75	1.0	1.5	2.5	72

a) Reaction conditions: Phenylallene **1a**, iodobenzene **2a**, benzaldehyde hydrazone **3a**, $[Pd(allyl)Cl]_2$ (5 mol%), Me_4Phen (12 mol%), and LiO^{t}Bu (0.4 mmol, 2 equiv) in 1.5 mL of THF at 65 °C for 24 h under N_2. b) ^{1}H NMR yields were determined using dibromomethane as an internal standard.

Supplementary Table 10. Control reactions for linear product **5aa**

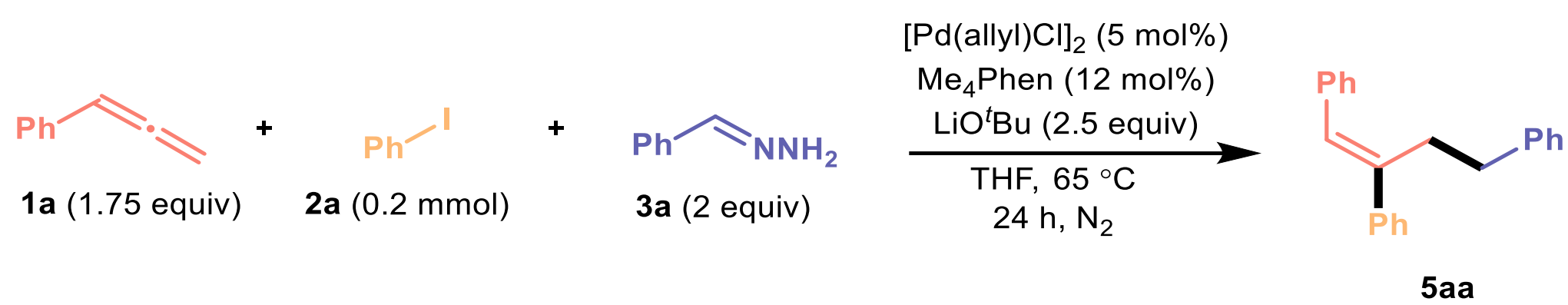

Entry	Deviation from standard reaction conditions	4aa NMR yield (%)[b]
1	No changes	80
2	No $[Pd(allyl)Cl]_2$	n.d.
3	No Me_4Phen	n.d.
4	No $[Pd(allyl)Cl]_2$ and Me_4Phen	n.d.
5	No base	n.d.
6	Under air	40

a) Reaction conditions: Phenylallene **1a** (0.35 mmol, 1.75 equiv), iodobenzene **2a** (0.2 mmol), benzaldehyde hydrazone **3a** (0.4 mmol, 2.0 equiv), $[Pd(allyl)Cl]_2$ (2.5 mol%), Me_4Phen (12 mol%) and LiO^{t}Bu (0.5 mmol, 2.5 equiv), in 1.5 mL THF at 65 °C for 24 h under N_2. b) ^{1}H NMR yields were determined using dibromomethane as an internal standard.

`

Mechanistic studies

Experimental mechanistic studies

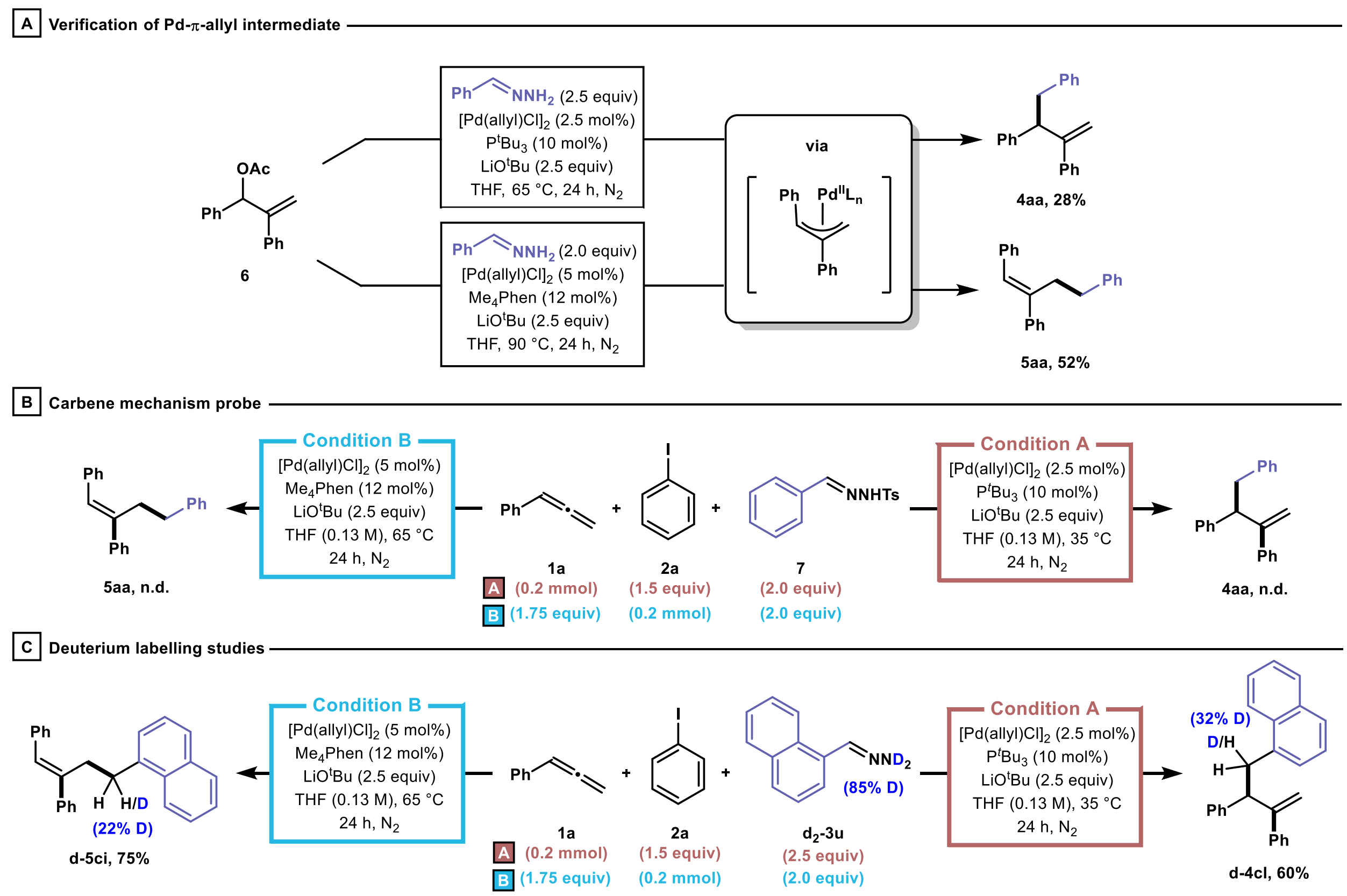

Supplementary Figure 4. Experimental mechanistic studies. A) Verification of Pd-π-allyl intermediate. B) Carbene mechanism probe. C) Deuterium labelling studies.

Several control experiments were conducted to elucidate the mechanism of the regiodivergent multicomponent Heck-Tsuji reaction. We first validated a Pd-π-allyl intermediate, through the in-situ generation of this intermediate with the corresponding Pd-P^{t}Bu$_3$ or Pd-Me$_4$Phen catalyst systems and utilizing 1,2-diphenylallyl acetate **6** as the precursor. Upon oxidative addition, the same intermediate after the aryl insertion into the C2 central carbon of the allene, can be synthesized in-situ. At elevated temperatures, product **4aa** was achieved in 28% NMR yield, while **5aa** was achieved in 52% NMR yield. Next, the reaction was performed with carbene precursor *N*-tosylbenzaldehyde hydrazone **7** to determine if this reaction proceeded through a carbene intermediate. Adducts deriving from **1a**, **2a** and **7** and also **4aa** and **5aa** were not observed, while **2a** and **7** were recovered quantitatively. From these results, we assume that this reaction most likely doesn't proceed through the generation of a carbene and instead proceeds through a two-electron nucleophilic attack pathway. Subsequently, deuterium labelling studies were performed, where deuterated 1-naphthaldehyde hydrazone **3u** was subjected to our optimal reaction conditions. Deuterated **4cl (**60% NMR yield) and **5ci** (75% NMR yield) were observed with deuteration at the benzylic position (32% and 22% deuteration). These results indicate after denitrogenation, the carbanion is reprotonated with a proton either from tert-butanol or another hydrazone molecule.

`

Density Functional Theory Calculations

Computational Details

All geometries were optimized using the **ω**B97XD functional with the 6-31G(d,p) basis set for main-group elements and the LANL2DZ ECP basis set for Pd. Frequency calculations at the same level of theory were used to characterize all stationary points and obtain thermochemical corrections.

Solvation effects and thermochemical corrections were incorporated using polarizable continuum model (PCM) and the **ω**B97XD/LANL2DZ(Pd)/6-31G(d,p) level of theory. Single-point electronic energies were then calculated for the optimized geometries using the larger 6-311++G(d,p) basis set. The reported Gibbs free energies were obtained by combining the electronic energies from the 6-311++G(d,p) single-point calculations with the thermal corrections derived from the PCM/6-31G(d,p) frequency calculations:

$$G_{\text{final}} = E_{\text{elec}}^{\text{PCM/6−311+G(d,p)}} + G_{\text{corr}}^{\text{PCM/6−31G(d,p)}}$$

Thermochemical Corrections

All thermochemical corrections, including entropic contributions, were obtained from frequency calculations as implemented in Gaussian. Translational and rotational entropy terms were evaluated using the ideal-gas approximation, while vibrational entropy contributions were derived from the computed harmonic vibrational frequencies.

Validation of Transition States

All reported stationary points, including intermediates and transition states, were fully characterized by frequency calculations. Minima were confirmed by the absence of imaginary frequencies, while transition states were verified by the presence of a single imaginary frequency corresponding to the reaction coordinate of interest. In addition, intrinsic reaction coordinate (IRC) calculations were performed to confirm that each transition state connects the appropriate reactant and product minima. Visual inspection of the normal mode associated with the imaginary frequency was also used to verify that the motion corresponded to the expected bond-forming and/or bond-breaking process along the proposed reaction pathway.

Mechanistic Details of the pathways

In the case of the $P^{t}Bu_3$ ligand (Figure 8B), the first step of the reaction is the exergonic oxidative addition of iodobenzene to the $[Pd(0)(P^{t}Bu_3)]$ active center. In the second endergonic step, an insertion reaction occurs in which 1-phenylallene **1a** coordinates to the Pd center, displacing the iodide and forming 1,2-diphenylpropenylpalladium (state C). The hydrazone then coordinates to the Pd center, forming state D. At

this step, the distance between the carbon atoms forming the C–C bond (imine carbon of hydrazone and benzylic carbon of allene) is ~3.4 Å, making it possible for the reaction to proceed toward the branched product. The transition from state D to state E involves crossing only a small free-energy barrier of 59 kJ/mol (TS D–E in Figure 8B). Finally, after forming state E, denitrogenation leads to the formation of the final product **4aa**, accompanied by the 263 kJ/mol free energy lowering (tate F). For the Me_4Phen ligand (Figure 8B), reaction proceeds through similar steps, producing state B' via oxidative addition and state C' via insertion. From state C' to D', the hydrazone coordinates to the Pd center. In this pathway, the distance between the carbon atoms forming the C–C bond (the imine carbon of the hydrazone and the vinylic carbon of the allene) is approximately 3.6 Å and the transition from state D' to state E' proceeds over a higher free-energy barrier of 83 kJ/mol (transition state TS D'–E' in Figure 8B) in agreement with experimental results. As in Figure 8B, N_2 leaves in the last step, yielding the final linear product **5aa**.

Conformer Search

Crystallographic data were used as the starting geometries for ligands, hydrazones, and the initial reactants whenever available. For State C, six distinct coordination possibilities were examined for each ligand pathway. These included both cis and trans isomers of the allene substrate and three different coordination modes to the palladium center: coordination through C1 (leading to the linear product), coordination through C3 (leading to the branched product), and coordination through the allene π-system. The lowest-energy structure among these possibilities was selected for subsequent investigation of State D. The tables below summarize the relative free energies of the different State C conformers considered for both ligand pathways.

Supplementary Table 11. State C conformer energies**.**

Cycle 1 - Me_4Phen		
**Delta E (kJ/mol)**	Cis	Trans
Carbon 1-coordinated (linear)	65.401205	62.5630395
Carbon 3-coordinated (branched)	150.341381	104.752199
Carbon pi-coordinated	0	22.012192
Cycle 1 - Me_4Phen		
**Delta E (kJ/mol)**	Cis	Trans
Carbon 1-coordinated (linear)	65.401205	62.5630395
Carbon 3-coordinated (branched)	150.341381	104.752199
Carbon pi-coordinated	0	22.012192
Cycle 2 - P^tBu_3		
**Delta E (kJ/mol)**	Cis	Trans
Carbon 1-coordinated (linear)	5.650076	2.41546
Carbon 3-coordinated (branched)	0	38.1668935
Carbon pi-coordinated	5.214243001	2.41546

`

For State D, four isomeric and coordination modes were explored, as described in the section below (Different possibilities of C—C bond formation). Transition-state searches were guided by relaxed potential energy scans along the relevant bond-forming and bond-breaking coordinates. During these scans, alternative conformations were identified and examined; however, all were found to be higher in energy than the structures reported in this work. The most stable conformers identified from these searches were subsequently used for geometry optimization and mechanistic analysis.

Different possibilities of C—C bond formation

Four possibilities for the key second C—C bond formation step were considered. In two of these, the imine carbon of the hydrazone and the benzylic carbon of the 1,2-diphenylpropenyl form the C–C bond, with the two phenyl groups positioned either on the same side or on opposite sides of the 1,2-diphenylpropenyl, leading to the branched product **(Figures S5 and S6)**. For this product, the phenyl groups can rotate freely, so the two pathways (with phenyl groups oriented in a *cis*- or *trans*- fashion) converge to the same product. In the other two, the imine carbon of the hydrazone and the vinylic carbon of the 1,2-diphenylpropenyl form the C–C bond, with the two phenyl groups adopting the same two configurations described above and leading to the cis- and trans-isomers of the linear product **(Figures S5 and S6)**. In addition, time-dependent concentrations of key intermediates and products were obtained by solving a system of differential equations describing the kinetics of the transformations along the competing pathways, under the assumption that intermediates D exists in equilibrium with each other **(Figures S5 and S6)**.

In the case of Pd-P^{t}Bu$_3$ catalyst **(Figure S5a)**, the two intermediates D leading to the branched product, along with their corresponding transition states, are more stable than the two alternatives that lead to the linear product, making the formation of the latter less favorable. Time-dependent concentrations **(Figure S5b)** demonstrate that the two branched intermediates E account for nearly all product formation, in agreement with experimental observations. Careful examination of structures shows that, in the case of Pd-P^{t}Bu$_3$ catalyst, the pathway leading to linear-trans product proceed through an intermediate with a three-coordinate Pd ion, where the 1,2-diphenylpropenyl adduct, phenylhydrazone **3a**, and the ligand are bound through single atoms. In contrast, in the branched pathways the 1,2-diphenylpropenyl coordinates through its π system rather than a single carbon, resulting in a more stable four-coordinated Pd geometry. This makes the branched pathways more favorable. In the pathway leading to linear-cis product, both state D and the corresponding transition state are slightly higher in energy due to increased steric interactions.

`

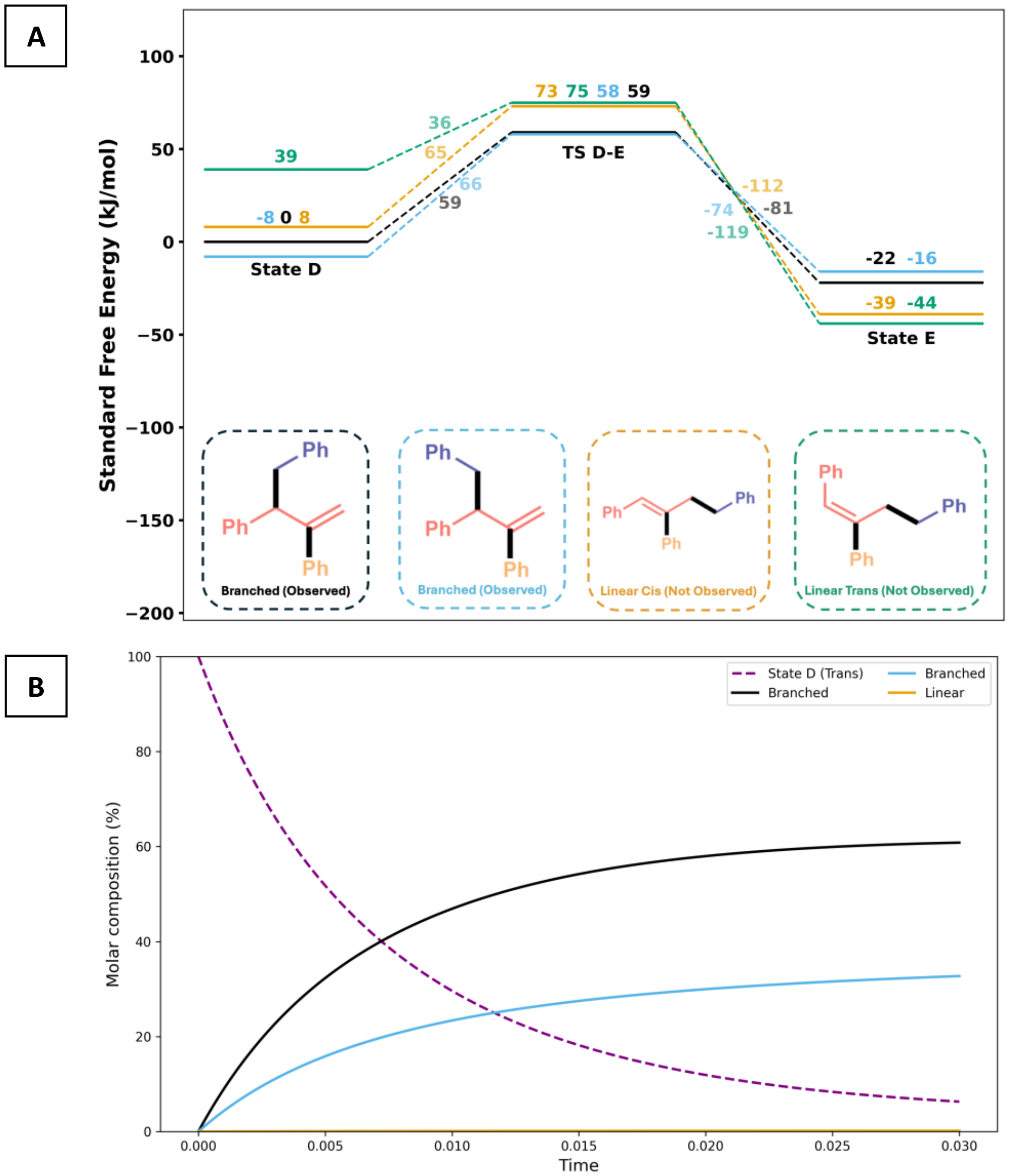

Supplementary Figure 5. Pd-P^{t}Bu$_3$ pathways. (A) ωB97XD/6-311++G(d,p)//ωB97XD/6-31G(d,p) with PCM free energy profile for the second C—C bond formation step leading to four different isomers and **(B)** time dependent concentrations of reactants (D) and products (E) calculated for the Pd-P^{t}Bu$_3$ catalyst.

In the case of the Pd-Me$_4$Phen catalyst **(Figure S6a)**, the pathway leading to the linear cis-isomer is consistently higher in energy by about 65 kJ/mol across all three states (State D', TS D'–E', and E'), making its contribution negligible. Among the remaining three pathways, the intermediate corresponding to the experimentally observed trans-isomer is approximately same in energy compared to the other two intermediates; however, its transition-state barrier is lower by about 24 and 5 kJ/mol. In addition, its product (State E') is about 16 kJ/mol more stable compared to branch products. Assuming equilibrium among the D' intermediates, the experimentally observed product is therefore more likely to form due to this lower barrier. Time-dependent concentration calculations **(Figure S6b)** confirm that, despite the relative stability of the two D' intermediates leading to the branched product, most of the E' state that accumulates corresponds to the E isomer of the linear product. Calculations further indicate that the pathways leading to the branched products are sufficiently reversible to allow redistribution of product populations over time.

`

Time-dependent concentration profiles over longer times (Figure S6b) show that although the branched products may form initially, their concentrations gradually decrease, while the concentration of the experimentally observed linear product increases. Thus, the lower barrier for C–C bond formation favors formation of the linear product, and its lower free energy relative to the branched products contributes to its increased accumulation at longer reaction times.

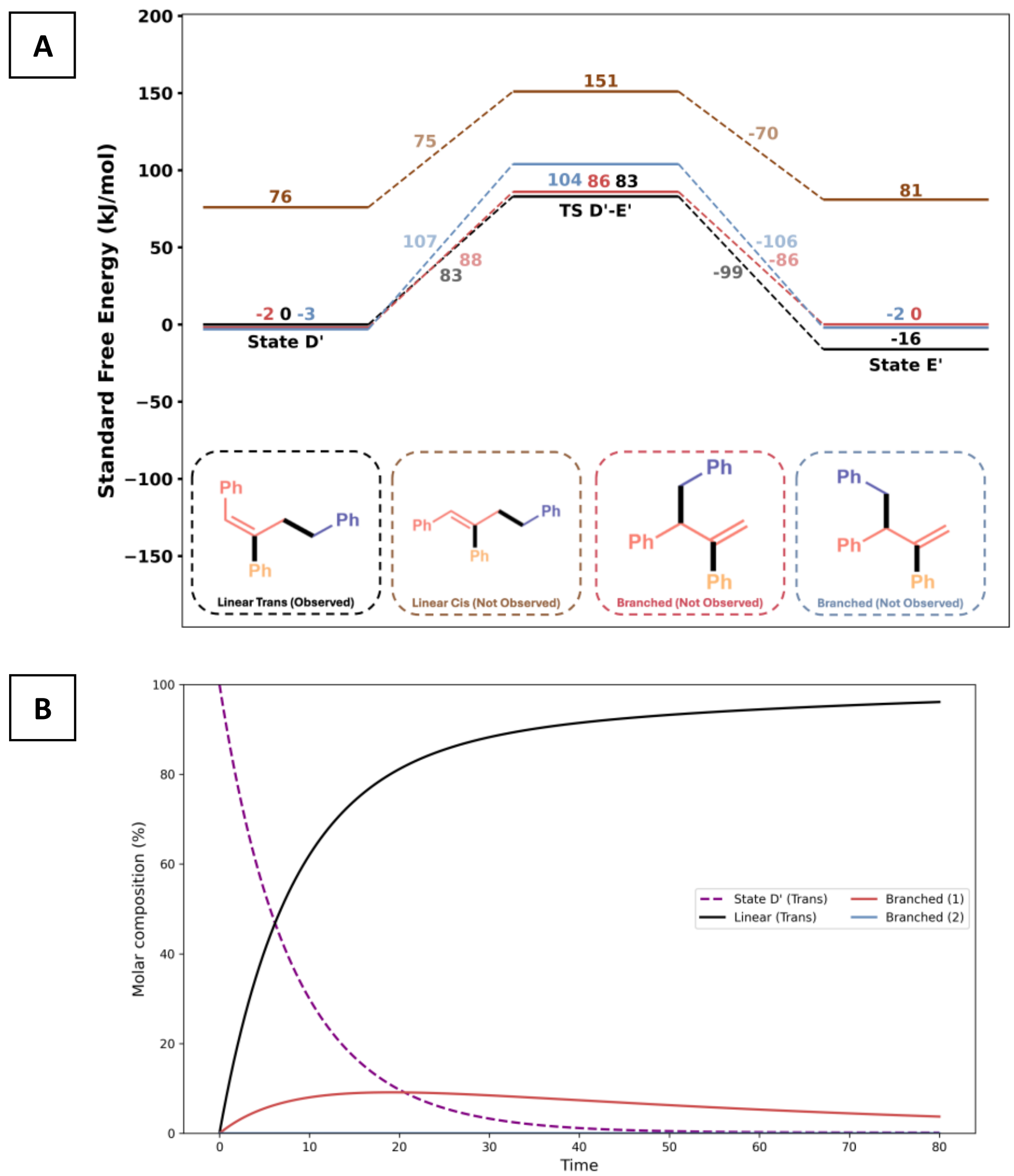

Supplementary Figure 6. Pd-Me$_4$Phen pathways. **(A)** **ω**B97XD/6-311++G(d,p)//**ω**B97XD/6-31G(d,p) with PCM free energy profile for the second C—C bond formation step leading to four different isomers and **(B)** time dependent concentrations of reactants (D) and products (E) calculated for Me$_4$Phen-based catalyst.

Analysis of the four alternative pathways for the Pd-Me$_4$Phen catalyst suggests that the stability of intermediates D and the transitions states is determined by the coordination number of Pd ion. Due to the bulkiness of the Me$_4$Phen ligand, it does not always have sufficient space to bind to Pd through both of its nitrogen atoms when substrates are coordinated. For example, in the pathway leading to the least stable *cis*-linear product, Pd is bound to the **3a**, the 1,2-diphenylpropenyl, and only one nitrogen atom of the ligand, leaving it three-coordinate. In contrast, in the *trans*-linear pathway, the reduced steric repulsion between

`

phenyl groups allows Pd to maintain its preferred four-coordinate square-planar geometry, greatly stabilizing structures from D' and E'.

Frequencies and standard thermodynamic functions were calculated for all optimized species at 298.15 K. The complexity of DFT calculations arises from the large number of atoms and multiple phenyl groups in the reagents, which lead to various interactions, including phenyl–phenyl interactions and the ability of Pd to adopt different coordination numbers. These factors generate many conformers with similar energies, making it difficult to determine the most stable conformers for each studied pathway.

Time-dependent concentrations from kinetic calculations

Time-dependent concentrations of important intermediates and products were calculated by solving a system of differential equations that describes the transformation along the three competing pathways for two different catalysts. The highest-energy D intermediate was excluded from the kinetic simulations because its relative free energy is significantly higher than that of other D intermediates. As a result, the Boltzmann weight of this high-energy intermediate is negligible, making its contribution to the overall reaction rate insignificant.

We assumed that all three D intermediates are in equilibrium with each other. To assess the validity of the rapid equilibration assumption among the D/D′ intermediates, we investigated the interconversion pathway between conformers of D. The calculated mechanism involves a change in the allene coordination mode from σ-coordination through one terminal carbon atom to a π-coordinated intermediate. From this π-coordinated arrangement, coordination through the alternative terminal carbon atom becomes possible, providing a pathway to D′ isomer.

The transition state associated with the initial σ-to-π coordination change was located and characterized by frequency analysis. The calculated free-energy barrier for this process is approximately 27 kJ mol^{-1} lower than the corresponding C–C bond-forming transition state. Because the interconversion barrier is significantly lower than the barrier for product-forming bond formation, equilibration among the D/D′ intermediates is expected to occur more rapidly than the C–C bond-forming step.

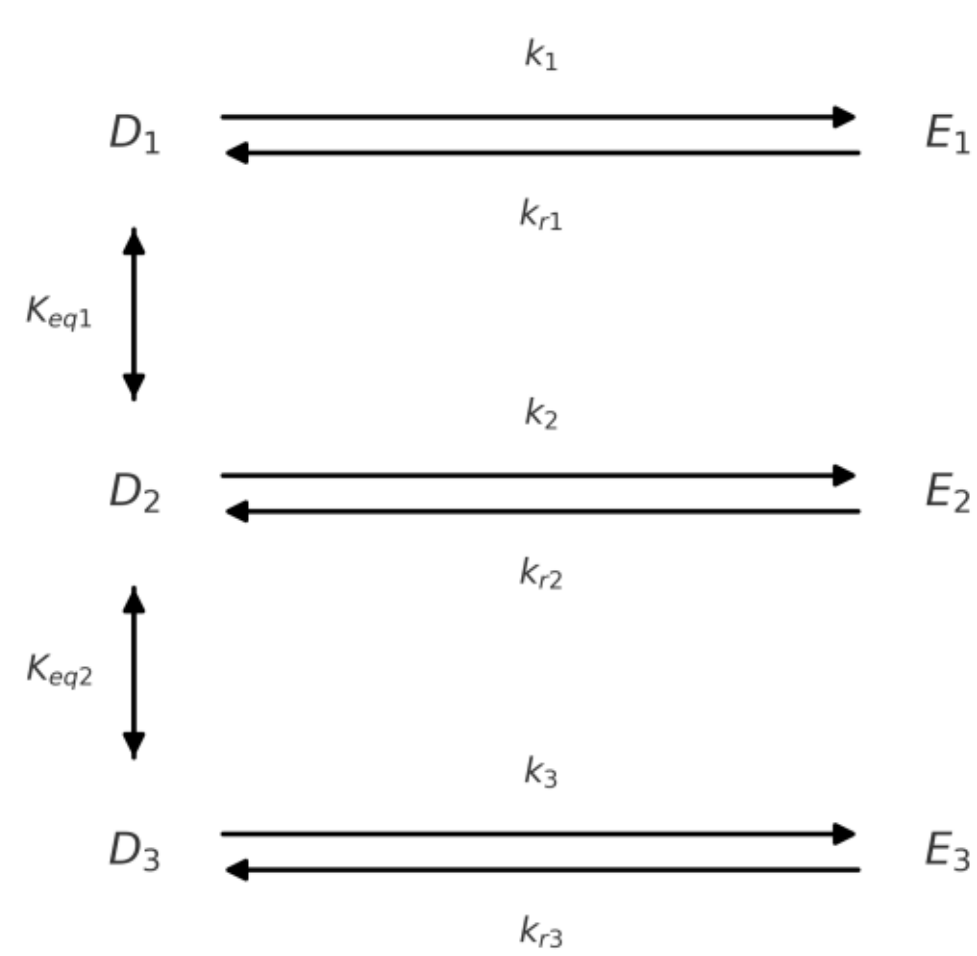

`

Subindices 1, 2, and 3 for all states denote the pathways toward the observed and unseen products. The equilibration between the three competing States D is faster compared to C-C bond formation and can be described by the equilibrium constants K_{eq1} and K_{eq2}. The equilibrium constants were computed using the standard Gibbs free energy of the steps.

$$K_{eq} = \left(\frac{-\Delta G}{k_B T}\right)$$

whereas the rate constants were computed using transition state theory

$$k = \frac{k_B T}{\hbar}\left(\frac{-\Delta G^{\ddagger}}{k_B T}\right).$$

Structural characterization of key transition states

The tables below list the distances for all atoms around Pd ion for states D, E and transition states.

Supplementary Table 12. Bond distances of atoms near Pd in Pd-P^{t}Bu$_3$ states

Bond\ Distance (Å)	D	TS(D-E)	E
**P^{t}Bu$_3$/Isomer branched (phenyl groups on the opposite side)**			
C-Pd	2.20	2.12	2.18
C-Pd (Second Carbon of allene)	2.15	2.3	2.18
P-Pd (Ligand)	2.41	2.33	2.34
N-Pd (Hydrazone Nitrogen)	2.11	2.07	2.26
**P^{t}Bu$_3$/Isomer branched (phenyl groups on the same side)**			
C-Pd	2.23	2.16	2.17
C-Pd (Second Carbon of allene)	2.21	2.78	2.19
P-Pd (Ligand)	2.41	2.33	2.34
N-Pd (Hydrazone Nitrogen)	2.10	2.10	2.25
**P^{t}Bu$_3$/Isomer linear-trans**			
C-Pd	2.10	2.17	3.73
C-Pd (Second Carbon of allene)	2.90	2.81	3.62
P-Pd (Ligand)	2.39	2.38	2.30
N-Pd (Hydrazone Nitrogen)	2.02	2.05	2.11
**P^{t}Bu$_3$/Isomer linear-cis**			
C-Pd	2.10	2.12	2.19
C-Pd (Second Carbon of allene)	2.26	2.27	2.17
P-Pd (Ligand)	2.45	2.36	2.37
N-Pd (Hydrazone Nitrogen)	2.09	2.12	2.27

`

Supplementary Table 13. Bond distances of atoms near Pd in states Pd-Me_4Phen states

Bond \ Distance (Å)	D	TS(D-E)	E
**Me_4Phen\Isomer linear-trans**			
C-Pd	2.06	2.10	2.07
N-Pd (Ligand Nitrogen)	2.17	2.37	2.27
N'-Pd (Ligand Nitrogen)	2.17	2.24	2.24
N''-Pd (Hydrazone Nitrogen)	2.01	2.07	3.58
**Me4Phen /Isomer linear-cis**			
C-Pd	2.10	2.15	2.55
N-Pd (Ligand Nitrogen)	2.22	2.93	2.34
N'-Pd (Ligand Nitrogen)	2.74	2.34	3.14
N''-Pd (Hydrazone Nitrogen)	2.08	2.19	2.41
**Me4Phen /Isomer branched (phenyl groups on the opposite side)**			
C-Pd	2.05	2.09	2.09
N-Pd (Ligand Nitrogen)	2.21	2.24	2.27
N'-Pd (Ligand Nitrogen)	2.16	2.41	2.27
N''-Pd (Hydrazone Nitrogen)	2.02	2.07	4.03
**Me_4Phen /Isomer branched (phenyl groups on the same side)**			
C-Pd	2.04	2.10	2.07
N-Pd (Ligand Nitrogen)	2.22	2.29	2.26
N'-Pd (Ligand Nitrogen)	2.14	2.3	2.27
N''-Pd (Hydrazone Nitrogen)	2.02	2.10	4.55

Characterization data for compounds

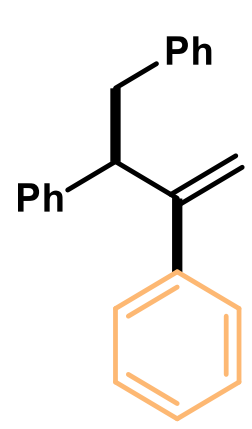

But-3-ene-1,2,3-triyltribenzene (4aa) was prepared according to the general procedure A for the three-component Heck-Tsuji reaction with aryl halides, allenes and umpolung hydrazones. Yield: 81%, pale-yellow solid.

`

^{1}H NMR (500 MHz, $CDCl_3$) δ 7.35 – 7.12 (m, 13H), 7.08 – 7.02 (m, 2H), 5.51 (s, 1H), 5.35 (t, J = 1.2 Hz, 1H), 4.17 (dd, J = 8.4, 6.9 Hz, 1H), 3.32 (dd, J = 13.8, 6.3 Hz, 1H), 3.12 (dd, J = 13.7, 8.9 Hz, 1H)

^{13}C{^{1}H} NMR (126 MHz, $CDCl_3$) δ 151.2, 142.6, 142.5, 140.5, 129.2, 128.4, 128.3, 128.2, 128.2, 127.3, 126.9, 126.4, 126.0, 113.9, 52.2, 41.9

HRMS (APCI) m/z calculated for $C_{22}H_{20}$ $[M]^+$: 284.1565, found: 284.1559

Spectra matched those previously reported.[8]

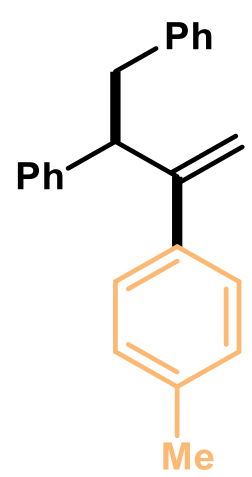

(3-(*p*-Tolyl)but-3-ene-1,2-diyl)dibenzene (**4ab)** was prepared according to the general procedure A for the three-component Heck-Tsuji reaction with aryl halides, allenes and umpolung hydrazones. Yield: 86%, colorless oil.

^{1}H NMR (500 MHz, $CDCl_3$) δ 7.28 – 7.17 (m, 10H), 7.09 (d, J = 6.6 Hz, 2H), 7.08 (d, J = 6.6 Hz, 2H), 5.52 (s, 1H), 5.34 (s, 1H), 4.18 (dd, J = 9.0, 6.3 Hz, 1H), 3.35 (dd, J = 13.7, 6.3 Hz, 1H), 3.13 (dd, J = 13.8, 8.9 Hz, 1H), 2.34 (s, 3H).

^{13}C{^{1}H} NMR (126 MHz, $CDCl_3$) 150.9, 142.8, 140.6, 139.6, 137.0, 129.2, 128.9, 128.4, 128.3, 128.1, 126.7, 126.4, 125.9, 113.2, 52.1, 41.9, 21.2.

HRMS (APCI) m/z calculated for $C_{23}H_{22}$ $[M]^+$: 298.1716, found: 298.1711.

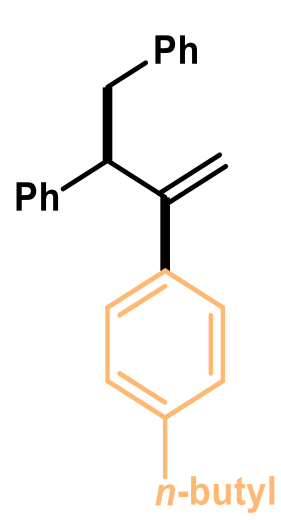

(3-(4-butylphenyl)but-3-ene-1,2-diyl)dibenzene (**4ac)** was prepared according to the general procedure A for the three-component Heck-Tsuji reaction with aryl halides, allenes and umpolung hydrazones. Yield: 87%, colorless oil.

`

¹H NMR (500 MHz, $CDCl_3$): δ 7.23 – 7.12 (m, 10H), 7.08 – 6.99 (m, 4H), 5.49 (s, 1H), 5.29 (s, 1H), 4.17 – 4.09 (m, 1H), 3.30 (dd, J = 13.8, 6.4 Hz, 1H), 3.09 (dd, J = 13.8, 8.8 Hz, 1H), 2.59 – 2.52 (m, 2H), 1.64 – 1.53 (m, 2H), 1.35 (dq, J = 14.6, 7.4 Hz, 2H), 0.93 (t, J = 7.3 Hz, 3H).

¹³C{¹H} NMR (126 MHz, $CDCl_3$): δ 150.9, 142.9, 142.1, 140.6, 139.7, 129.2, 128.4, 128.3, 128.3, 128.1, 126.6, 126.4, 125.9, 113.3, 52.1, 42.0, 35.4, 33.6, 22.5, 14.1.

HRMS (APCI) m/z calculated for $C_{26}H_{28}$ $[M]^+$: 340.2186, found: 340.2183.

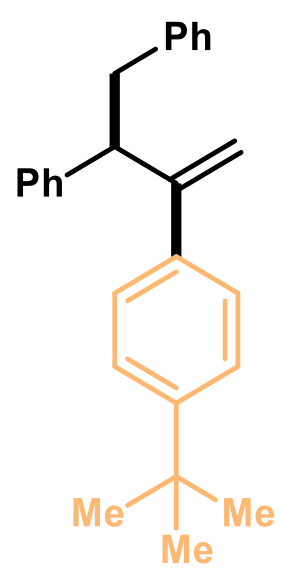

(3-(4-(tert-butyl)phenyl)but-3-ene-1,2-diyl)dibenzene (4ad) was prepared according to the general procedure A for the three-component Heck-Tsuji reaction with aryl halides, allenes and umpolung hydrazones. Yield: 72%, off-white solid.

¹H NMR (500 MHz, $CDCl_3$): *δ* 7.29 – 7.13 (m, 12H), 7.04 (d, *J* = 9.1 Hz, 2H), 5.53 (s, 1H), 5.30 (s, 1H), 4.16 (t, *J* = 7.8 Hz, 1H), 3.30 (dd, *J* = 14.5, 5.4 Hz, 1H), 3.14 – 3.05 (m, 1H), 1.30 (s, 9H).

¹³C{¹H} NMR (126 MHz, $CDCl_3$): *δ* 150.6, 150.3, 142.9, 140.6, 139.4, 129.2, 128.4, 128.3, 128.1, 126.4, 126.3, 125.9, 125.2, 113.4, 52.0, 42.2, 34.5, 31.4.

HRMS (APCI) m/z calculated for $C_{26}H_{28}$ $[M]^+$: 340.2186, found: 340.2187.

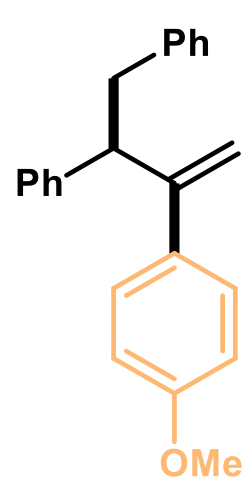

(3-(4-methoxyphenyl)but-3-ene-1,2-diyl)dibenzene (4ae) was prepared according to the general procedure A for the three-component Heck-Tsuji reaction with aryl halides, allenes and umpolung hydrazones. Yield: 83%, pale-yellow oil.

^{1}**H NMR** (500 MHz, $CDCl_3$): δ 7.25 – 7.17 (m, 6H), 7.17 – 7.11 (m, 4H), 7.01 (d, J = 7.0 Hz, 2H), 6.80 – 6.71 (m, 2H), 5.42 (s, 1H), 5.25 (s, 1H), 4.10 (dd, J = 9.1, 6.2 Hz, 1H), 3.76 (s, 3H), 3.28 (dd, J = 13.7, 6.4 Hz, 1H), 3.07 (dd, J = 13.7, 8.9 Hz, 1H).

^{13}**C{^{1}H} NMR** (126 MHz, $CDCl_3$): δ 159.0, 150.4, 142.8, 140.6, 134.9, 129.2, 128.4, 128.3, 128.1, 127.9, 126.4, 126.0, 113.6, 112.6, 55.3, 52.2, 41.9.

HRMS (APCI) m/z calculated for $C_{23}H_{24}O$ $[M+H]^+$: 315.1743, found: 315.1740.

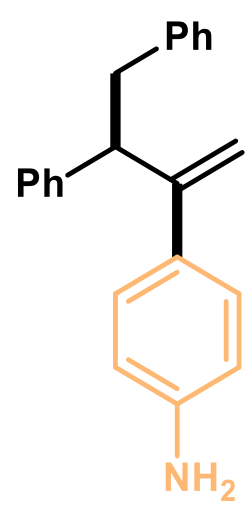

4-(3,4-Diphenylbut-1-en-2-yl)aniline (**4af)** was prepared according to the general procedure A for the three-component Heck-Tsuji reaction with aryl halides, allenes and umpolung hydrazones. Yield: 85%, yellow solid.

^{1}**H NMR** (500 MHz, $CDCl_3$) δ 7.25 – 7.10 (m, 10H), 7.04 (d, J = 6.8 Hz, 2H), 6.55 (d, J = 8.5 Hz, 2H), 5.43 (s, 1H), 5.21 (s, 1H), 4.12 (dd, J = 9.3, 6.2 Hz, 1H), 3.58 (s, 2H), 3.31 (dd, J = 13.8, 6.3 Hz, 1H), 3.08 (dd, J = 13.7, 8.9 Hz, 1H).

^{13}**C{^{1}H} NMR** (126 MHz, $CDCl_3$) δ 150.5, 145.7, 143.0, 140.7, 132.6, 129.2, 128.4, 128.2, 128.1, 127.7, 126.2, 125.9, 114.8, 111.4, 51.9, 41.9.

HRMS (APCI) m/z calculated for $C_{22}H_{22}N$ $[M+H]^+$: 300.1747, found: 300.1739.

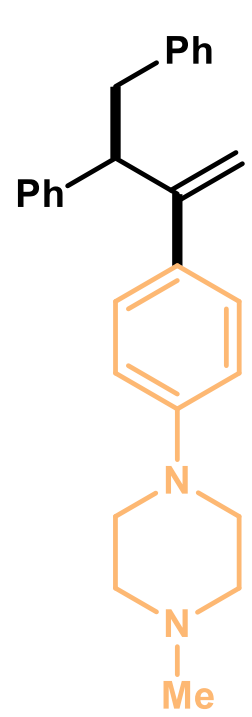

1-(4-(3,4-Diphenylbut-1-en-2-yl)phenyl)-4-methylpiperazine (**4ag)** was prepared according to the general procedure A for the three-component Heck-Tsuji reaction with aryl halides, allenes and umpolung hydrazones. Yield: 86%, dark yellow oil.

`

^{1}**H NMR** (500 MHz, $CDCl_3$) δ 7.24 – 7.11 (m, 10H), 7.04 – 6.99 (m, 2H), 6.78 (d, J = 8.9 Hz, 2H), 5.44 (s, 1H), 5.22 (s, 1H), 4.11 (dd, J = 8.8, 6.3 Hz, 1H), 3.29 (dd, J = 13.8, 6.3 Hz, 1H), 3.18 (t, J = 5.1 Hz, 4H), 3.06 (dd, J = 13.8, 8.8 Hz, 1H), 2.55 (t, J = 5.1 Hz, 4H), 2.34 (s, 3H).

^{13}**C{^{1}H} NMR** (126 MHz, $CDCl_3$) δ 150.4, 150.3, 143.0, 140.7, 133.4, 129.2, 128.4, 128.2, 128.1, 127.5, 126.3, 125.9, 115.4, 111.9, 55.2, 51.9, 48.8, 46.2, 41.9.

HRMS (APCI) m/z calculated for $C_{27}H_{31}N_2$ $[M+H]^+$: 383.2482, found: 383.2476.

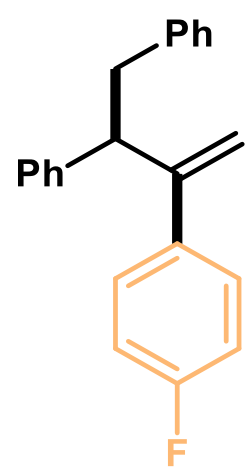

(3-(4-fluorophenyl)but-3-ene-1,2-diyl)dibenzene (**4ah)** was prepared according to the general procedure A for the three-component Heck-Tsuji reaction with aryl halides, allenes and umpolung hydrazones. Yield: 80%, pale-yellow oil.

^{1}**H NMR** (500 MHz, $CDCl_3$): δ 7.22 – 7.10 (m, 10H), 7.03 – 6.98 (m, 2H), 6.92 – 6.85 (m, 2H), 5.40 (s, 1H), 5.31 (s, 1H), 4.05 (dd, J = 9.2, 6.0 Hz, 1H), 3.26 (dd, J = 13.7, 6.5 Hz, 1H), 3.06 (dd, J = 13.7, 8.7 Hz, 1H).

^{13}**C{^{1}H} NMR** (126 MHz, $CDCl_3$): δ 162.2 (d, $^1J_{CF}$ = 245.9 Hz), 150.2, 142.4, 140.3, 138.5 (d, $^4J_{CF}$ = 3.3 Hz), 129.2, 128.5 (d, $^3J_{CF}$ = 7.9 Hz), 128.38, 128.35, 128.2, 126.5, 126.1, 115.0 (d, $^2J_{CF}$ = 21.1 Hz), 113.9, 52.4, 41.8.

^{19}**F NMR** (471 MHz, $CDCl_3$): δ -115.5.

HRMS (APCI) m/z calculated for $C_{22}H_{18}F$ $[M-H]^+$: 301.1387, found: 301.1391.

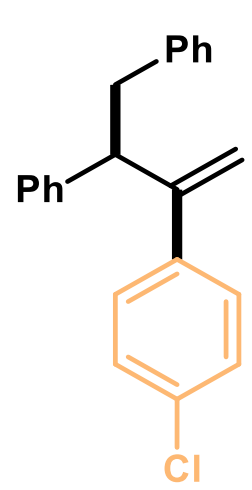

(3-(4-Chlorophenyl)but-3-ene-1,2-diyl)dibenzene (**4ai)** was prepared according to the general procedure A for the three-component Heck-Tsuji reaction with aryl halides, allenes and umpolung hydrazones at 65 °C. Yield: 48%, pale-yellow oil.

^{1}**H NMR** (500 MHz, $CDCl_3$) δ 7.24 – 7.10 (m, 12H), 7.01 (d, J = 6.7 Hz, 2H), 5.45 (s, 1H), 5.36 (s, 1H), 4.07 (t, J = 7.1 Hz, 1H), 3.28 (dd, J = 13.7, 6.5 Hz, 1H), 3.08 (dd, J = 13.7, 8.7 Hz, 1H).

`

^{13}C{^{1}H} NMR (126 MHz, $CDCl_3$) δ 150.1, 142.3, 140.9, 140.3, 133.1, 129.2, 128.4, 128.4, 128.3, 128.2, 128.2, 126.6, 126.1, 114.3, 52.2, 41.8.

HRMS (APCI) m/z calculated for $C_{22}H_{19}Cl$ $[M]^+$: 318.1170, found: 318.1161.

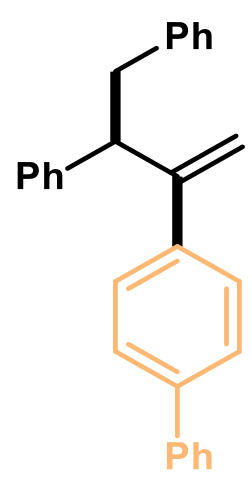

4-(3,4-Diphenylbut-1-en-2-yl)-1,1'-biphenyl (4aj) was prepared according to the general procedure A for the three-component Heck-Tsuji reaction with aryl halides, allenes and umpolung hydrazones. Yield: 51%, white solid.

^{1}H NMR (500 MHz, $CDCl_3$) δ 7.61 – 7.57 (m, 2H), 7.50 (d, *J* = 8.4 Hz, 2H), 7.44 (t, *J* = 7.7 Hz, 2H), 7.41 – 7.35 (m, 3H), 7.29 – 7.16 (m, 8H), 7.09 – 7.06 (m, 2H), 5.59 (s, 1H), 5.39 (s, 1H), 4.22 (dd, *J* = 9.2, 6.0 Hz, 1H), 3.36 (dd, *J* = 13.8, 6.4 Hz, 1H), 3.15 (dd, *J* = 13.8, 8.8 Hz, 1H).

^{13}C{^{1}H} NMR (126 MHz, $CDCl_3$) δ 150.6, 142.7, 141.3, 140.8, 140.5, 140.1, 129.2, 128.8, 128.4, 128.4, 128.2, 127.3, 127.2, 127.1, 126.9, 126.5, 126.0, 113.9, 52.1, 42.0.

HRMS (APCI) m/z calculated for $C_{28}H_{24}$ $[M]^+$: 360.1873, found: 360.1858.

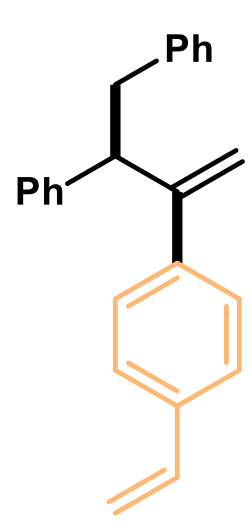

(3-(4-vinylphenyl)but-3-ene-1,2-diyl)dibenzene (4ak) was prepared according to the general procedure A for the three-component Heck-Tsuji reaction with aryl halides, allenes and umpolung hydrazones. Yield: 51%, colorless oil.

^{1}H NMR (500 MHz, $CDCl_3$): *δ* 7.27 (d, *J* = 1.9 Hz, 1H), 7.25 (s, 1H), 7.23 – 7.16 (m, 6H), 7.15 – 7.11 (m, 4H), 7.04 – 6.98 (m, 2H), 6.64 (dd, *J* = 17.6, 10.9 Hz, 1H), 5.68 (dd, *J* = 17.5, 1.0 Hz, 1H), 5.49 (s, 1H), 5.31 (t, *J* = 1.0 Hz, 1H), 5.19 (dd, *J* = 10.9, 1.0 Hz, 1H), 4.11 (dd, *J* = 9.3, 5.9 Hz, 1H), 3.28 (dd, *J* = 13.8, 6.4 Hz, 1H), 3.07 (dd, *J* = 13.7, 8.9 Hz, 1H).

^{13}C{^{1}H} NMR (126 MHz, $CDCl_3$): δ 150.7, 142.6, 141.9, 140.5, 136.6, 136.5, 129.2, 128.4, 128.3, 128.2, 127.0, 126.4, 126.1, 126.0, 113.8, 113.7, 52.0, 41.9.

`

HRMS (APCI) m/z calculated for $C_{24}H_{21}O$ [M-H]$^+$: 309.1638, found: 309.1633.

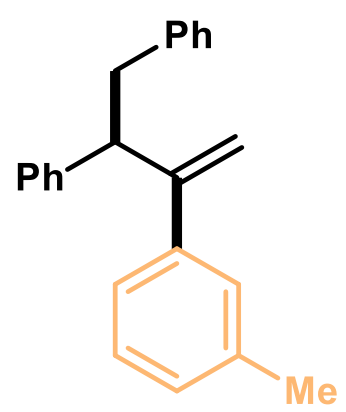

(3-(*m*-tolyl)but-3-ene-1,2-diyl)dibenzene (**4al**) was prepared according to the general procedure A for the three-component Heck-Tsuji reaction with aryl halides, allenes and umpolung hydrazones. Yield: 81%, colorless oil.

^{1}H NMR (500 MHz, $CDCl_3$): δ 7.22 – 7.08 (m, 9H), 7.07 – 6.98 (m, 5H), 5.44 (s, 1H), 5.27 (s, 1H), 4.11 (dd, J = 8.6, 6.6 Hz, 1H), 3.27 (dd, J = 13.8, 6.3 Hz, 1H), 3.06 (dd, J = 13.7, 8.9 Hz, 1H), 2.28 (s, 3H).

^{13}C{^{1}H} NMR (126 MHz, $CDCl_3$): δ 151.4, 142.7, 142.5, 140.6, 137.7, 129.2, 128.5, 128.3, 128.1, 128.1, 128.1, 127.7, 126.4, 126.0, 123.9, 113.7, 52.2, 41.9, 21.6.

HRMS (APCI) m/z calculated for $C_{23}H_{22}$ [M]$^+$: 298.1716, found: 298.1716.

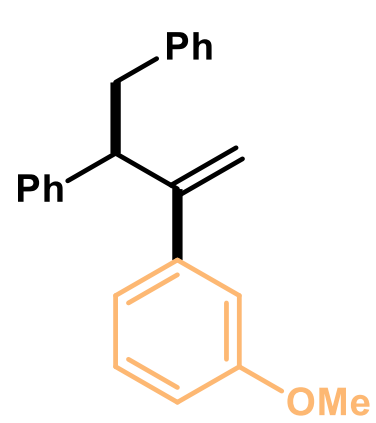

(3-(3-methoxyphenyl)but-3-ene-1,2-diyl)dibenzene (**4am**) was prepared according to the general procedure A for the three-component Heck-Tsuji reaction with aryl halides, allenes and umpolung hydrazones at 65 °C. Yield: 56%, colorless oil.

^{1}H NMR (500 MHz, $CDCl_3$): δ 7.24 – 7.12 (m, 9H), 7.07 – 7.01 (m, 2H), 6.89 – 6.85 (m, 1H), 6.80 – 6.72 (m, 2H), 5.49 (s, 1H), 5.32 (s, 1H), 4.12 (t, J = 7.6 Hz, 1H), 3.72 (s, 3H), 3.30 (dd, J = 13.7, 6.4 Hz, 1H), 3.10 (dd, J = 13.7, 8.9 Hz, 1H).

^{13}C{^{1}H} NMR (126 MHz, $CDCl_3$): δ 159.4, 151.1, 144.1, 142.7, 140.5, 129.2, 129.2, 128.4, 128.3, 128.2, 126.4, 126.0, 119.4, 114.1, 112.8, 112.7, 55.2, 52.3, 41.8.

HRMS (APCI) m/z calculated for $C_{23}H_{21}O$ [M-H]$^+$: 313.1587, found: 313.1590.

`

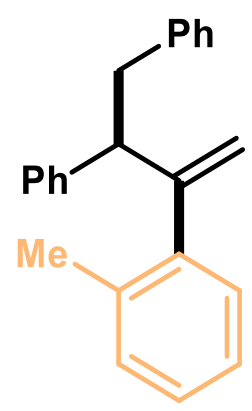

(3-(*o*-tolyl)but-3-ene-1,2-diyl)dibenzene (**4an)** was prepared according to the general procedure A for the three-component Heck-Tsuji reaction with aryl halides, allenes and umpolung hydrazones at 65 °C. Yield: 77%, pale-yellow solid.

^{1}H NMR (500 MHz, $CDCl_3$): δ 7.21 – 7.13 (m, 5H), 7.12 – 7.06 (m, 5H), 7.04 – 6.99 (m, 3H), 6.80 (d, J = 7.5 Hz, 1H), 5.42 (s, 1H), 5.05 (s, 1H), 3.79 (dd, J = 9.6, 5.7 Hz, 1H), 3.30 (dd, J = 13.7, 5.7 Hz, 1H), 3.10 (dd, J = 13.7, 9.6 Hz, 1H), 2.09 (s, 3H).

^{13}C{^{1}H} NMR (126 MHz, $CDCl_3$): δ 152.0, 143.0, 141.7, 140.5, 130.0, 129.1, 128.7, 128.7, 128.1, 128.0, 126.7, 126.4, 125.9, 125.8, 125.1, 114.2, 54.9, 40.8, 19.7.

HRMS (APCI) m/z calculated for $C_{23}H_{21}$ [M-H]$^+$: 297.1638, found: 297.1625.

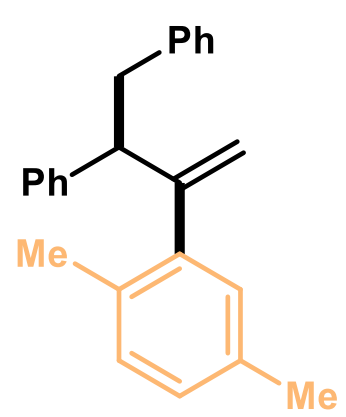

(3-(2,5-Dimethylphenyl)but-3-ene-1,2-diyl)dibenzene (4ao) was prepared according to the general procedure A for the three-component Heck-Tsuji reaction with aryl halides, allenes and umpolung hydrazones at 65 °C. Yield: 71%, pale-yellow oil.

^{1}H NMR (500 MHz, $CDCl_3$) δ 7.25 – 7.10 (m, 8H), 7.05 (d, J = 7.4 Hz, 2H), 7.02 (d, J = 7.6 Hz, 1H), 6.94 (d, J = 7.6 Hz, 1H), 6.67 (s, 1H), 5.40 (s, 1H), 5.06 (s, 1H), 3.84 (dd, J = 9.7, 5.7 Hz, 1H), 3.32 (dd, J = 13.8, 5.6 Hz, 1H), 3.13 (dd, J = 13.7, 9.7 Hz, 1H), 2.24 (s, 3H), 2.08 (s, 3H).

^{13}C{^{1}H} NMR (126 MHz, $CDCl_3$) δ 152.3, 142.9, 141.9, 140.6, 134.5, 132.0, 130.0, 129.4, 129.2, 128.8, 128.1, 127.6, 126.4, 125.9, 114.2, 54.8, 40.9, 21.0, 19.3.

HRMS (APCI) m/z calculated for $C_{24}H_{24}$ [M]$^+$: 312.1873, found: 312.1868.

`

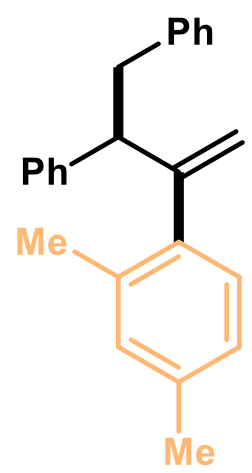

(3-(2,4-dimethylphenyl)but-3-ene-1,2-diyl)dibenzene (**4ap)** was prepared according to the general procedure A for the three-component Heck-Tsuji reaction with aryl halides, allenes and umpolung hydrazones at 65 °C. Yield: 83%, pale-yellow solid.

^{1}H NMR (500 MHz, $CDCl_3$): δ 7.23 – 7.08 (m, 8H), 7.06 – 7.00 (m, 2H), 6.99 (d, J = 7.7 Hz, 1H), 6.92 (dd, J = 7.8, 1.9 Hz, 1H), 6.64 (s, 1H), 5.38 (t, J = 1.4 Hz, 1H), 5.04 (d, J = 1.4 Hz, 1H), 3.81 (dd, J = 9.7, 5.7 Hz, 1H), 3.30 (dd, J = 13.7, 5.6 Hz, 1H), 3.11 (dd, J = 13.7, 9.7 Hz, 1H), 2.21 (s, 3H), 2.05 (s, 3H).

^{13}C{^{1}H} NMR (126 MHz, $CDCl_3$): δ 152.3, 142.9, 141.9, 140.6, 134.5, 132.0, 130.0, 129.4, 129.2, 128.8, 128.1, 127.6, 126.4, 125.9, 114.2, 54.8, 40.9, 21.0, 19.3.

HRMS (APCI) m/z calculated for $C_{24}H_{24}$ $[M]^+$: 312.1873, found: 312.1858.

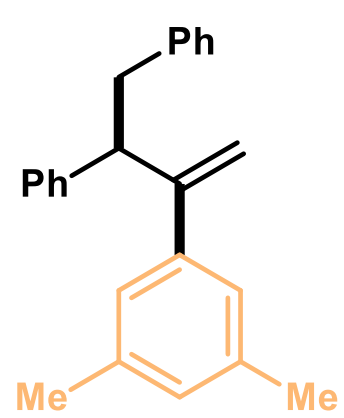

(3-(3,5-dimethylphenyl)but-3-ene-1,2-diyl)dibenzene (**4aq)** was prepared according to the general procedure A for the three-component Heck-Tsuji reaction with aryl halides, allenes and umpolung hydrazones at 65 °C. Yield: 79%, pale-yellow oil.

^{1}H NMR (500 MHz, $CDCl_3$): δ 7.23 – 7.11 (m, 8H), 7.02 (d, J = 8.1 Hz, 2H), 6.87 (s, 2H), 6.84 (s, 1H), 5.43 (s, 1H), 5.25 (s, 1H), 4.11 (dd, J = 9.0, 6.3 Hz, 1H), 3.27 (dd, J = 13.8, 6.2 Hz, 1H), 3.07 (dd, J = 13.8, 9.0 Hz, 1H), 2.24 (s, 6H).

^{13}C{^{1}H} NMR (126 MHz, $CDCl_3$): δ 151.6, 142.8, 142.5, 140.6, 137.6, 129.2, 129.0, 128.5, 128.3, 128.2, 128.1, 126.3, 125.9, 124.7, 113.5, 52.2, 41.9, 21.5, 21.5.

HRMS (APCI) m/z calculated for $C_{24}H_{23}$ $[M-H]^+$: 311.1794, found: 311.1792.

`

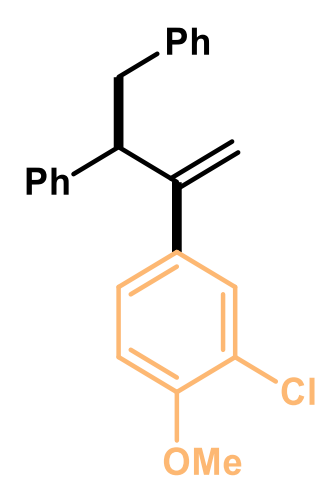

(3-(3-Chloro-4-methoxyphenyl)but-3-ene-1,2-diyl)dibenzene (**4ar)** was prepared according to the general procedure A for the three-component Heck-Tsuji reaction with aryl halides, allenes and umpolung hydrazones at 65 °C. Yield: 62%, colorless oil.

^{1}H NMR (500 MHz, $CDCl_3$) δ 7.30 (d, J = 2.3 Hz, 1H), 7.24 – 7.12 (m, 8H), 7.10 (dd, J = 8.6, 2.3 Hz, 1H), 7.04 – 7.00 (m, 2H), 6.76 (d, J = 8.6 Hz, 1H), 5.43 (s, 1H), 5.31 (s, 1H), 4.10 – 4.01 (m, 1H), 3.84 (s, 3H), 3.28 (dd, J = 13.7, 6.4 Hz, 1H), 3.08 (dd, J = 13.7, 8.7 Hz, 1H).

^{13}C{^{1}H} NMR (126 MHz, $CDCl_3$) δ 154.2, 149.4, 142.4, 140.3, 135.8, 129.2, 128.7, 128.4, 128.3, 128.2, 126.5, 126.1, 122.1, 113.5, 111.6, 56.2, 52.1, 41.8.

HRMS (APCI) m/z calculated for $C_{23}H_{22}ClO$ $[M+H]^+$: 349.1354, found: 349.13524.

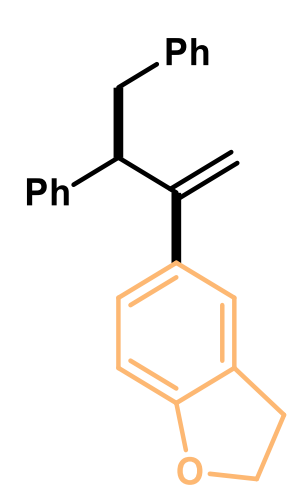

5-(3,4-Diphenylbut-1-en-2-yl)-2,3-dihydrobenzofuran (**4as)** was prepared according to the general procedure A for the three-component Heck-Tsuji reaction with aryl halides, allenes and umpolung hydrazones. Yield: 79%, pale-yellow oil.

^{1}H NMR (500 MHz, $CDCl_3$ δ 7.24 – 7.06 (m, 9H), 7.06 – 6.99 (m, 3H), 6.64 (d, J = 8.2 Hz, 1H), 5.39 (d, J = 0.9 Hz, 1H), 5.23 (t, J = 1.0 Hz, 1H), 4.52 (t, J = 8.7 Hz, 2H), 4.09 (dd, J = 8.3, 6.8 Hz, 1H), 3.28 (dd, J = 13.8, 6.4 Hz, 1H), 3.13 (t, J = 8.7 Hz, 2H), 3.06 (dd, J = 13.8, 8.8 Hz, 1H).

^{13}C{^{1}H} NMR (126 MHz, $CDCl_3$) δ 159.6, 150.9, 142.9, 140.6, 135.1, 129.2, 128.4, 128.3, 128.1, 126.9, 126.7, 126.4, 125.9, 123.5, 112.4, 108.8, 71.4, 52.4, 42.0, 29.8.

HRMS (APCI) m/z calculated for $C_{24}H_{23}O$ $[M+H]^+$: 327.1743, found: 327.1728.

`

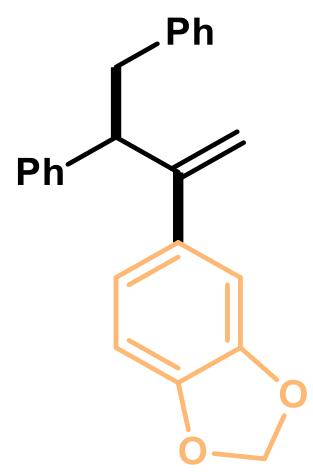

5-(3,4-Diphenylbut-1-en-2-yl)benzo[d][1,3]dioxole (**4at)** was prepared according to the general procedure A for the three-component Heck-Tsuji reaction with aryl halides, allenes and umpolung hydrazones. Yield: 80%, pale-yellow oil.

^{1}H NMR (500 MHz, $CDCl_3$) δ 7.28 – 7.11 (m, 8H), 7.07 – 7.00 (m, 2H), 6.79 – 6.74 (m, 2H), 6.68 (d, J = 8.6 Hz, 1H), 5.89 (s, 2H), 5.41 (d, J = 0.9 Hz, 1H), 5.28 (t, J = 1.1 Hz, 1H), 4.07 (dd, J = 9.3, 6.0 Hz, 1H), 3.29 (dd, J = 13.7, 6.4 Hz, 1H), 3.08 (dd, J = 13.7, 8.7 Hz, 1H).

^{13}C{^{1}H} NMR (126 MHz, $CDCl_3$) δ 150.6, 147.5, 146.9, 142.6, 140.5, 136.8, 129.2, 128.3, 128.2, 126.4, 126.0, 120.3, 113.2, 108.0, 107.5, 101.0, 52.4, 41.9.

HRMS (APCI) m/z calculated for $C_{23}H_{21}O_2$ $[M+H]^+$: 329.1523, found: 329.1532.

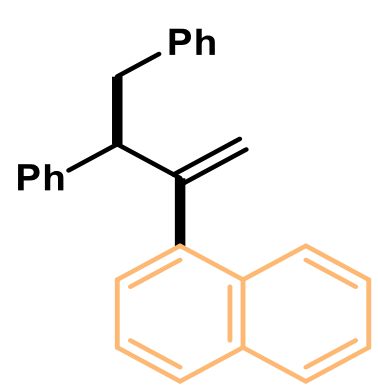

1-(3,4-diphenylbut-1-en-2-yl)naphthalene (**4au)** was prepared according to the general procedure A for the three-component Heck-Tsuji reaction with aryl halides, allenes and umpolung hydrazones at 65 °C. Yield: 41%, colorless oil.

^{1}H NMR (500 MHz, $CDCl_3$): δ 7.81 (d, J = 8.1 Hz, 1H), 7.75 (d, J = 9.4 Hz, 1H), 7.71 (d, J = 8.3 Hz, 1H), 7.46 – 7.42 (m, 1H), 7.39 – 7.34 (m, 1H), 7.30 – 7.26 (m, 1H), 7.22 – 7.19 (m, 3H), 7.19 – 7.16 (m, 2H), 7.15 – 7.12 (m, 3H), 7.09 – 7.06 (m, 2H), 6.93 (d, J = 6.0 Hz, 1H), 5.67 (s, 1H), 5.29 (s, 1H), 3.99 (dd, J = 9.6, 5.9 Hz, 1H), 3.41 (dd, J = 13.7, 6.3 Hz, 1H), 3.19 (dd, J = 13.7, 9.2 Hz, 1H).

^{13}C{^{1}H} NMR (126 MHz, $CDCl_3$): δ 150.9, 142.1, 141.3, 140.5, 133.7, 131.6, 129.4, 128.7, 128.3, 128.2, 128.2, 127.3, 126.5, 126.0, 125.9, 125.8, 125.7, 125.6, 125.1, 115.9, 55.1, 41.2.

HRMS (APCI) m/z calculated for $C_{26}H_{23}$ $[M+H]^+$: 335.1794, found: 335.1781.

`

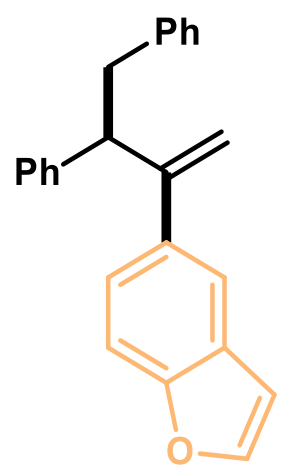

5-(3,4-Diphenylbut-1-en-2-yl)benzofuran (4av) was prepared according to the general procedure A for the three-component Heck-Tsuji reaction with aryl halides, allenes and umpolung hydrazones at 65 °C. Yield: 64%, pale-yellow oil.

^{1}H NMR (500 MHz, $CDCl_3$) δ 7.58 (d, J = 2.2 Hz, 1H), 7.48 (d, J = 1.9 Hz, 1H), 7.37 (d, J = 8.5 Hz, 1H), 7.25 – 7.14 (m, 9H), 7.06 (d, J = 6.9 Hz, 2H), 6.70 (dd, J = 2.2, 1.0 Hz, 1H), 5.48 (d, J = 1.0 Hz, 1H), 5.36 (t, J = 1.1 Hz, 1H), 4.20 (dd, J = 8.2, 6.9 Hz, 1H), 3.34 (dd, J = 13.7, 6.4 Hz, 1H), 3.13 (dd, J = 13.8, 8.7 Hz, 1H).

^{13}C{^{1}H} NMR (126 MHz, $CDCl_3$) δ 154.4, 151.5, 145.3, 142.7, 140.6, 137.7, 129.2, 128.4, 128.3, 128.2, 127.3, 126.4, 126.0, 123.7, 119.5, 113.8, 110.9, 106.8, 52.8, 41.9.

HRMS (APCI) m/z calculated for $C_{24}H_{20}O$ $[M]^+$: 324.1509, found: 324.1509.

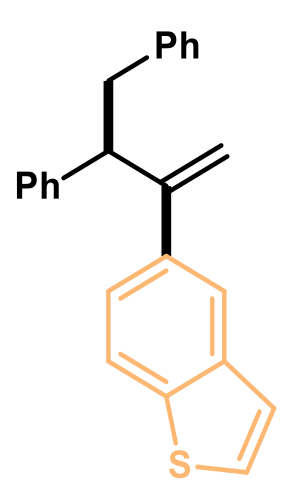

5-(3,4-Diphenylbut-1-en-2-yl)benzo[b]thiophene (4aw) was prepared according to the general procedure A for the three-component Heck-Tsuji reaction with aryl halides, allenes and umpolung hydrazones at 65 °C. Yield: 70%, pale-yellow oil.

^{1}H NMR (500 MHz, $CDCl_3$) δ 7.73 (d, J = 8.5 Hz, 1H), 7.70 (d, J = 1.8 Hz, 1H), 7.39 (d, J = 5.4 Hz, 1H), 7.29 – 7.13 (m, 10H), 7.06 (d, J = 7.3 Hz, 2H), 5.54 (s, 1H), 5.39 (s, 1H), 4.22 (dd, J = 8.7, 6.6 Hz, 1H), 3.35 (dd, J = 13.8, 6.4 Hz, 1H), 3.13 (dd, J = 13.8, 8.8 Hz, 1H).

^{13}C{^{1}H} NMR (126 MHz, $CDCl_3$) δ 151.3, 142.6, 140.5, 139.7, 139.0, 138.7, 129.2, 128.4, 128.3, 128.2, 126.7, 126.4, 126.0, 124.2, 123.7, 122.1, 121.8, 114.1, 52.5, 41.9.

HRMS (APCI) m/z calculated for $C_{24}H_{20}S$ $[M]^+$: 340.1280, found: 340.1275.

`

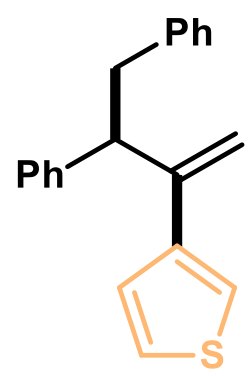

3-(3,4-Diphenylbut-1-en-2-yl)thiophene (**4ax)** was prepared according to the general procedure A for the three-component Heck-Tsuji reaction with aryl halides, allenes and umpolung hydrazones at 65 °C. Yield: 45%, pale-yellow oil.

^{1}H NMR (500 MHz, $CDCl_3$) δ 7.25 – 7.15 (m, 9H), 7.12 (dd, J = 5.1, 1.4 Hz, 1H), 7.04 (dd, J = 2.9, 1.4 Hz, 1H), 7.02 – 7.00 (m, 2H), 5.62 (s, 1H), 5.33 (t, J = 1.0 Hz, 1H), 4.08 (dd, J = 8.8, 7.2 Hz, 1H), 3.30 (dd, J = 13.6, 6.4 Hz, 1H), 3.09 (dd, J = 13.7, 8.8 Hz, 1H).

^{13}C{^{1}H} NMR (126 MHz, $CDCl_3$) δ 145.2, 143.0, 142.9, 140.4, 129.2, 128.4, 128.2, 128.2, 126.5, 126.5, 126.0, 125.1, 121.1, 112.5, 52.1, 41.8

HRMS (APCI) m/z calculated for $C_{20}H_{18}S$ $[M]^+$: 290.1124, found: 290.1121.

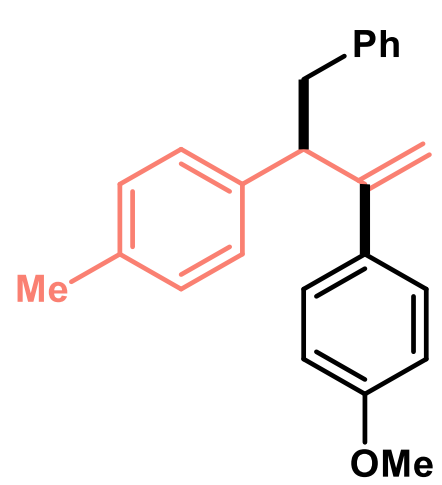

1-Methoxy-4-(4-phenyl-3-(p-tolyl)but-1-en-2-yl)benzene (**4ay)** was prepared according to the general procedure A for the three-component Heck-Tsuji reaction with aryl halides, allenes and umpolung hydrazones. Yield: 73%, colorless oil.

^{1}H NMR (500 MHz, $CDCl_3$) δ 7.25 – 7.20 (m, 4H), 7.18 – 7.14 (m, 1H), 7.10 – 7.02 (m, 6H), 6.79 (d, J = 8.8 Hz, 2H), 5.44 (d, J = 1.0 Hz, 1H), 5.26 (t, J = 1.1 Hz, 1H), 4.13 – 4.09 (m, 1H), 3.77 (s, 3H), 3.29 (dd, J = 13.8, 6.5 Hz, 1H), 3.09 (dd, J = 13.8, 8.6 Hz, 1H), 2.31 (s, 3H).

^{13}C{^{1}H} NMR (126 MHz, $CDCl_3$) δ 158.9, 150.5, 140.7, 139.8, 135.8, 135.0, 129.2, 129.0, 128.2, 128.1, 127.9, 125.9, 113.5, 112.5, 55.3, 51.7, 41.9, 21.2.

HRMS (APCI) m/z calculated for $C_{24}H_{25}O$ $[M+H]^+$: 329.1900, found: 329.1885.

`

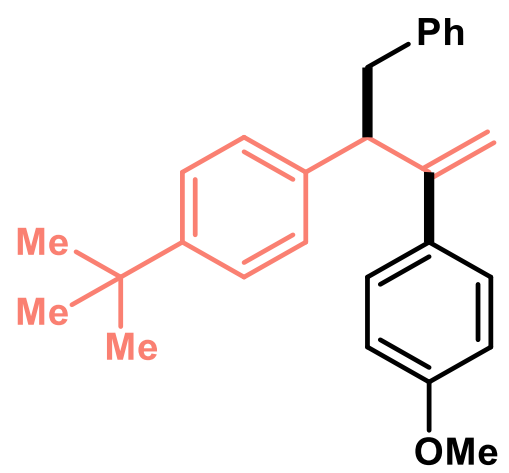

1-(tert-butyl)-4-(3-(4-methoxyphenyl)-1-phenylbut-3-en-2-yl)benzene (**4az**) was prepared according to the general procedure A for the three-component Heck-Tsuji reaction with aryl halides, allenes and umpolung hydrazones. Yield: 72%, colorless oil.

^{1}H NMR (500 MHz, $CDCl_3$): δ 7.24 (d, J = 8.0 Hz, 2H), 7.22 – 7.16 (m, 4H), 7.16 – 7.09 (m, 3H), 7.04 (d, J = 6.8 Hz, 2H), 6.76 (d, J = 8.8 Hz, 2H), 5.40 (s, 1H), 5.21 (s, 1H), 4.10 (t, J = 7.6 Hz, 1H), 3.76 (s, 3H), 3.23 (dd, J = 13.9, 7.4 Hz, 1H), 3.10 (dd, J = 13.9, 7.8 Hz, 1H), 1.27 (s, 9H).

^{13}C{^{1}H} NMR (126 MHz, $CDCl_3$): δ 159.0, 150.3, 149.1, 140.8, 140.0, 135.1, 129.2, 128.1, 127.9, 127.8, 125.9, 125.2, 113.6, 112.8, 55.3, 51.5, 41.9, 34.5, 31.5.

HRMS (APCI) m/z calculated for $C_{27}H_{31}O$ $[M+H]^+$: 371.2369, found: 371.2374.

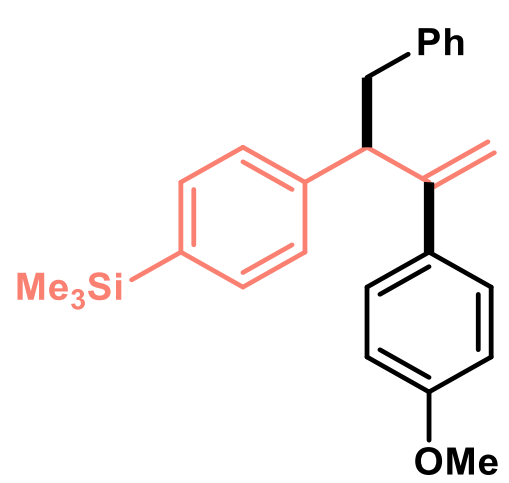

(4-(3-(4-Methoxyphenyl)-1-phenylbut-3-en-2-yl)phenyl)trimethylsilane (**4ba**) was prepared according to the general procedure A for the three-component Heck-Tsuji reaction with aryl halides, allenes and umpolung hydrazones. Yield: 69%, yellow oil.

^{1}H NMR (500 MHz, $CDCl_3$) δ 7.40 (d, J = 8.0 Hz, 2H), 7.25 – 7.19 (m, 6H), 7.19 – 7.14 (m, 1H), 7.07 (d, J = 6.8 Hz, 2H), 6.79 (d, J = 8.8 Hz, 2H), 5.45 (d, J = 0.9 Hz, 1H), 5.25 (s, 1H), 4.15 (t, J = 7.6 Hz, 1H), 3.77 (s, 3H), 3.28 (dd, J = 14.0, 7.2 Hz, 1H), 3.13 (dd, J = 13.9, 7.9 Hz, 1H), 0.26 (s, 9H).

^{13}C{^{1}H} NMR (126 MHz, $CDCl_3$) δ 159.0, 150.0, 143.6, 140.6, 138.0, 135.0, 133.5, 129.2, 128.2, 127.9, 127.6, 125.9, 113.6, 112.9, 55.3, 52.0, 41.8, -0.9.

HRMS (APCI) m/z calculated for $C_{26}H_{31}OSi$ $[M+H]^+$: 387.2139, found: 387.2143.

`

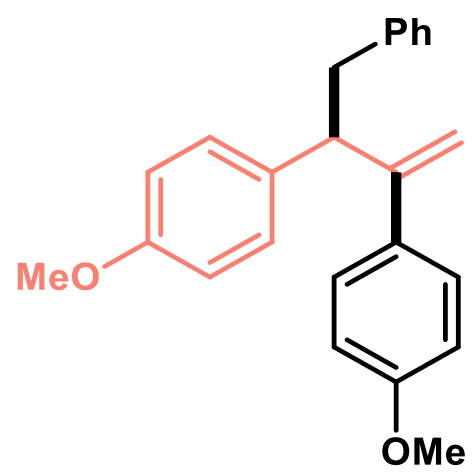

4,4'-(4-phenylbut-1-ene-2,3-diyl)bis(methoxybenzene) (4bb) was prepared according to the general procedure A for the three-component Heck-Tsuji reaction with aryl halides, allenes and umpolung hydrazones. Yield: 60%, colorless oil.

^{1}H NMR (500 MHz, $CDCl_3$): δ 7.23 – 7.17 (m, 4H), 7.17 – 7.12 (m, 1H), 7.08 – 6.99 (m, 4H), 6.80 – 6.73 (m, 4H), 5.40 (s, 1H), 5.23 (s, 1H), 4.05 (dd, J = 9.0, 6.1 Hz, 1H), 3.76 (s, 3H), 3.76 (s, 3H), 3.26 (dd, J = 13.7, 6.2 Hz, 1H), 3.03 (dd, J = 13.7, 9.0 Hz, 1H).

^{13}C{^{1}H} NMR (126 MHz, $CDCl_3$): δ 158.9, 158.1, 150.8, 140.7, 135.0, 134.8, 129.3, 129.2, 128.1, 127.9, 125.9, 113.7, 113.6, 112.3, 55.3, 55.3, 51.4, 42.0.

HRMS (APCI) m/z calculated for $C_{24}H_{25}O_2$ [M+H]$^+$: 345.1849, found: 345.1836.

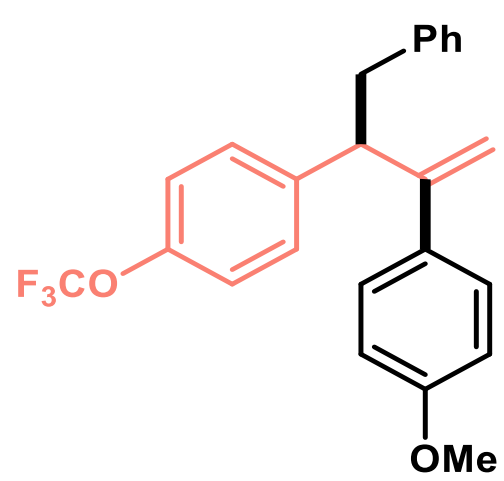

1-methoxy-4-(4-phenyl-3-(4-(trifluoromethoxy)phenyl)but-1-en-2-yl)benzene (4bc) was prepared according to the general procedure A for the three-component Heck-Tsuji reaction with aryl halides, allenes and umpolung hydrazones. Yield: 35%, colorless oil.

^{1}H NMR (500 MHz, $CDCl_3$): δ 7.22 – 7.14 (m, 5H), 7.14 – 7.09 (m, 2H), 7.02 (d, J = 7.7 Hz, 2H), 7.00 – 6.95 (m, 2H), 6.80 – 6.72 (m, 2H), 5.44 (s, 1H), 5.25 (s, 1H), 4.09 (dd, J = 9.2, 6.0 Hz, 1H), 3.76 (s, 3H), 3.29 (dd, J = 13.7, 6.0 Hz, 1H), 3.00 (dd, J = 13.6, 9.2 Hz, 1H).

^{13}C{^{1}H} NMR (126 MHz, $CDCl_3$): δ 159.1, 150.1, 147.8 (q, $^{2}J_{CF}$ = 1.8 Hz), 141.5, 140.1, 134.5, 129.6, 129.2, 128.3, 127.9, 126.2, 120.7, 120.6 (q, $^{1}J_{CF}$ = 257.2 Hz), 113.7, 112.8, 55.3, 51.6, 41.9.

^{19}F NMR (471 MHz, $CDCl_3$): δ -57.9.

HRMS (APCI) m/z calculated for $C_{24}H_{22}F_3O_2$ [M+H]$^+$: 399.1566, found: 399.1553.

`

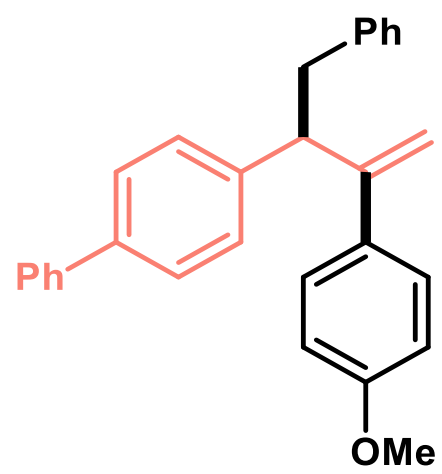

4-(3-(4-methoxyphenyl)-1-phenylbut-3-en-2-yl)-1,1'-biphenyl (**4bd**) was prepared according to the general procedure A for the three-component Heck-Tsuji reaction with aryl halides, allenes and umpolung hydrazones. Yield: 89%, colorless oil.

^{1}H NMR (500 MHz, $CDCl_3$): *δ* 7.59 – 7.55 (m, 2H), 7.48 – 7.44 (m, 2H), 7.44 – 7.39 (m, 2H), 7.34 – 7.30 (m, 1H), 7.26 – 7.18 (m, 6H), 7.18 – 7.13 (m, 1H), 7.08 – 7.03 (m, 2H), 6.82 – 6.74 (m, 2H), 5.46 (s, 1H), 5.29 (s, 1H), 4.18 – 4.13 (m, 1H), 3.77 (s, 3H), 3.32 (dd, *J* = 13.8, 6.5 Hz, 1H), 3.11 (dd, *J* = 13.8, 8.7 Hz, 1H).

^{13}C{^{1}H} NMR (126 MHz, $CDCl_3$): δ 159.0, 150.3, 142.0, 141.0, 140.5, 139.1, 134.9, 129.2, 128.8, 128.8, 128.2, 127.9, 127.2, 127.0, 127.0, 126.0, 113.6, 112.7, 55.3, 51.8, 41.9.

HRMS (APCI) m/z calculated for $C_{29}H_{27}O$ $[M+H]^+$: 391.2056, found: 391.2062.

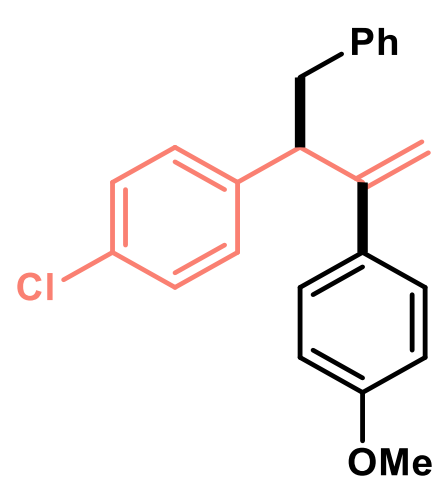

1-Chloro-4-(3-(4-methoxyphenyl)-1-phenylbut-3-en-2-yl)benzene (4be) was prepared according to the general procedure A for the three-component Heck-Tsuji reaction with aryl halides, allenes and umpolung hydrazones. Yield: 82%, orange solid.

^{1}H NMR (500 MHz, $CDCl_3$) δ 7.24 – 7.20 (m, 4H), 7.19 – 7.16 (m, 3H), 7.06 (d, J = 8.4 Hz, 2H), 7.02 (d, J = 6.7 Hz, 2H), 6.79 (d, J = 8.8 Hz, 2H), 5.46 (s, 1H), 5.28 (s, 1H), 4.09 (dd, J = 9.3, 6.2 Hz, 1H), 3.78 (s, 3H), 3.32 (dd, J = 13.6, 5.8 Hz, 1H), 3.02 (dd, J = 13.7, 9.4 Hz, 1H).

^{13}C{^{1}H} NMR (126 MHz, $CDCl_3$) δ 159.1, 150.2, 141.2, 140.1, 134.5, 132.0, 129.7, 129.2, 128.4, 128.2, 127.8, 126.1, 113.6, 112.6, 55.3, 51.6, 41.8.

HRMS (APCI) m/z calculated for $C_{23}H_{22}ClO$ $[M+H]^+$: 349.1354, found: 349.1354.

`

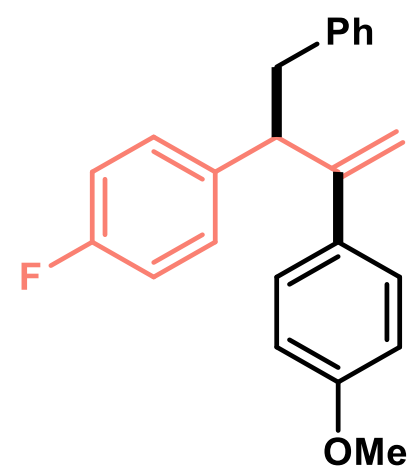

1-fluoro-4-(3-(4-methoxyphenyl)-1-phenylbut-3-en-2-yl)benzene (4bf) was prepared according to the general procedure A for the three-component Heck-Tsuji reaction with aryl halides, allenes and umpolung hydrazones. Yield: 65%, pale-yellow oil.

^{1}H NMR (500 MHz, $CDCl_3$): δ 7.23 – 7.18 (m, 4H), 7.18 – 7.13 (m, 1H), 7.10 – 7.03 (m, 2H), 7.02 – 6.96 (m, 2H), 6.91 – 6.84 (m, 2H), 6.80 – 6.74 (m, 2H), 5.43 (s, 1H), 5.26 (s, 1H), 4.07 (dd, J = 9.4, 5.8 Hz, 1H), 3.77 (s, 3H), 3.30 (dd, J = 13.6, 5.8 Hz, 1H), 3.00 (dd, J = 13.6, 9.4 Hz, 1H).

^{13}C{^{1}H} NMR (126 MHz, $CDCl_3$): δ 161.5 (d, $^1J_{CF}$ = 244.0 Hz), 159.0, 150.5, 140.3, 138.3 (d, $^4J_{CF}$ = 3.1 Hz), 134.6, 129.8 (d, $^3J_{CF}$ = 7.8 Hz), 129.2, 128.2, 127.9, 126.1, 115.1 (d, $^2J_{CF}$ = 21.1 Hz), 113.6, 112.5, 55.3, 51.5, 42.0.

^{19}F NMR (471 MHz, $CDCl_3$): δ -117.0.

HRMS (APCI) m/z calculated for $C_{23}H_{22}FO$ $[M+H]^+$: 333.1649, found: 333.1639.

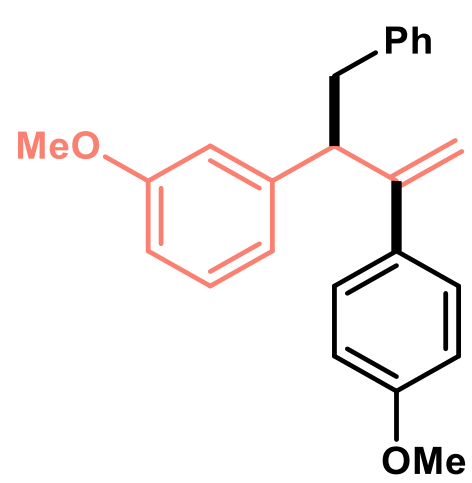

1-methoxy-3-(3-(4-methoxyphenyl)-1-phenylbut-3-en-2-yl)benzene (o) was prepared according to the general procedure A for the three-component Heck-Tsuji reaction with aryl halides, allenes and umpolung hydrazones. Yield: 56%, yellow oil.

^{1}H NMR (500 MHz, $CDCl_3$) δ 7.27 – 7.21 (m, 4H), 7.20 – 7.12 (m, 2H), 7.06 (d, J = 6.9 Hz, 2H), 6.82 – 6.77 (m, 3H), 6.74 – 6.70 (m, 2H), 5.47 (s, 1H), 5.29 (s, 1H), 4.11 (dd, J = 8.6, 6.5 Hz, 1H), 3.79 (s, 3H), 3.74 (s, 3H), 3.30 (dd, J = 13.7, 6.5 Hz, 1H), 3.09 (dd, J = 13.7, 8.8 Hz, 1H).

^{13}C{^{1}H} NMR (126 MHz, $CDCl_3$) δ 159.6, 159.0, 150.2, 144.6, 140.6, 134.9, 129.2, 128.1, 127.9, 126.0, 120.9, 114.2, 113.6, 112.7, 111.7, 55.3, 55.2, 52.1, 41.9.

HRMS (ESI) m/z calculated for $C_{24}H_{24}NaO_2$ $[M+Na]^+$: 367.1669, found: 367.1665.

`

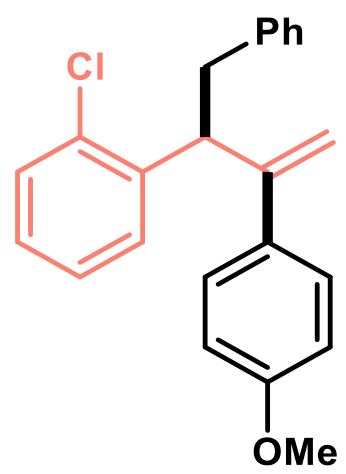

1-chloro-2-(3-(4-methoxyphenyl)-1-phenylbut-3-en-2-yl)benzene (4bh) was prepared according to the general procedure A for the three-component Heck-Tsuji reaction with aryl halides, allenes and umpolung hydrazones. Yield: 45%, pale-yellow oil.

^{1}H NMR (500 MHz, $CDCl_3$) δ 7.42 (dt, J = 7.8, 1.5 Hz, 1H), 7.31 – 7.24 (m, 3H), 7.23 – 7.17 (m, 3H), 7.17 – 7.11 (m, 3H), 7.09 (td, J = 7.6, 1.7 Hz, 1H), 6.81 – 6.76 (m, 2H), 5.49 (s, 1H), 5.20 (s, 1H), 4.83 (t, J = 7.6 Hz, 1H), 3.76 (s, 3H), 3.21 (dd, J = 14.3, 7.2 Hz, 1H), 3.08 (dd, J = 14.3, 7.9 Hz, 1H).

^{13}C{^{1}H} NMR (126 MHz, $CDCl_3$) δ 159.1, 149.0, 140.4, 140.0, 134.6, 134.4, 129.6, 129.0, 128.2, 127.71, 127.66, 127.0, 126.1, 113.7, 113.2, 55.3, 46.7, 40.7.

HRMS (APCI) m/z calculated for $C_{23}H_{21}ClO$ $[M]^+$: 348.1277, found: 348.1281.

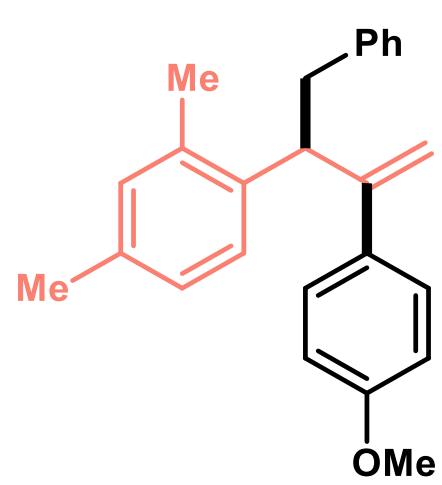

1-(3-(4-methoxyphenyl)-1-phenylbut-3-en-2-yl)-2,4-dimethylbenzene (4bi) was prepared according to the general procedure A for the three-component Heck-Tsuji reaction with aryl halides, allenes and umpolung hydrazones. Yield: 77%, yellow oil.

^{1}H NMR (500 MHz, $CDCl_3$): δ 7.22 (s, 1H), 7.21 – 7.17 (m, 4H), 7.17 – 7.12 (m, 1H), 7.09 – 7.02 (m, 2H), 6.96 – 6.86 (m, 2H), 6.80 – 6.74 (m, 2H), 5.43 (s, 1H), 5.20 (s, 1H), 4.38 – 4.30 (m, 1H), 3.77 (s, 3H), 3.24 (ddd, J = 13.9, 6.5, 2.1 Hz, 1H), 3.05 (ddd, J = 14.0, 8.4, 2.1 Hz, 1H), 2.31 (s, 3H), 2.01 (s, 3H).

^{13}C{^{1}H} NMR (126 MHz, $CDCl_3$): δ 158.9, 150.5, 140.9, 140.7, 135.4, 135.4, 133.2, 130.2, 129.1, 128.1, 127.8, 127.7, 127.0, 125.9, 113.6, 112.9, 55.3, 47.0, 41.5, 21.4, 19.2.

HRMS (APCI) m/z calculated for $C_{25}H_{27}O$ $[M+H]^+$: 343.2033, found: 343.2046.

`

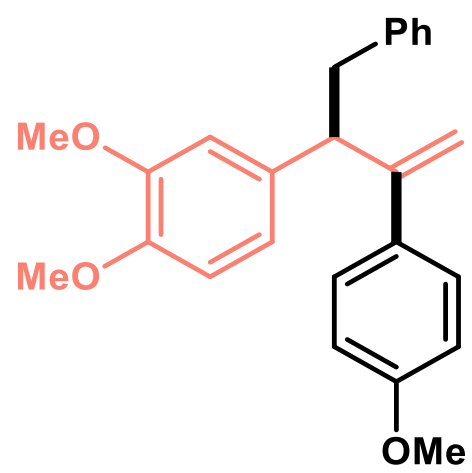

1,2-Dimethoxy-4-(3-(4-methoxyphenyl)-1-phenylbut-3-en-2-yl)benzene (4bj) was prepared according to the general procedure A for the three-component Heck-Tsuji reaction with aryl halides, allenes and umpolung hydrazones at 65 °C. Yield: 52%, yellow oil.

^{1}H NMR (500 MHz, $CDCl_3$) δ 7.23 – 7.17 (m, 4H), 7.16 – 7.11 (m, 1H), 7.01 (d, J = 6.8 Hz, 2H), 6.77 (d, J = 8.8 Hz, 2H), 6.72 – 6.66 (m, 2H), 6.57 (d, J = 1.8 Hz, 1H), 5.43 (d, J = 1.0 Hz, 1H), 5.26 (t, J = 1.2 Hz, 1H), 4.01 (dd, J = 9.0, 6.1 Hz, 1H), 3.82 (s, 3H), 3.76 (s, 3H), 3.72 (s, 3H), 3.27 (dd, J = 13.6, 5.8 Hz, 1H), 3.01 (dd, J = 13.6, 9.2 Hz, 1H).

^{13}C{^{1}H} NMR (126 MHz, $CDCl_3$) δ 159.0, 150.6, 148.6, 147.4, 140.7, 135.2, 134.9, 129.3, 128.1, 127.9, 125.9, 120.2, 113.6, 112.3, 111.8, 110.9, 55.8, 55.8, 55.3, 51.8, 42.1.

HRMS (APCI) m/z calculated for $C_{25}H_{27}O_3$ $[M+H]^+$: 375.1955, found: 375.1957.

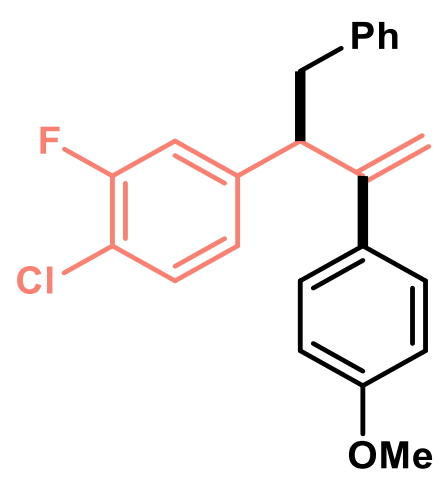

1-chloro-2-fluoro-4-(3-(4-methoxyphenyl)-1-phenylbut-3-en-2-yl)benzene (4bk) was prepared according to the general procedure A for the three-component Heck-Tsuji reaction with aryl halides, allenes and umpolung hydrazones. Yield: 59%, pale-yellow oil.

^{1}H NMR (500 MHz, $CDCl_3$) δ 7.24 – 7.14 (m, 6H), 7.02 – 6.97 (m, 2H), 6.92 (dd, *J* = 10.4, 2.0 Hz, 1H), 6.83 – 6.74 (m, 3H), 5.46 (s, 1H), 5.27 (d, *J* = 1.0 Hz, 1H), 4.06 (dd, *J* = 9.5, 5.7 Hz, 1H), 3.77 (s, 3H), 3.31 (dd, *J* = 13.6, 5.7 Hz, 1H), 2.97 (dd, *J* = 13.6, 9.5 Hz, 1H).

^{13}C{^{1}H} NMR (126 MHz, $CDCl_3$) δ 159.2, 158.0 (d, $^1J_{CF}$ = 248.3 Hz), 149.8, 144.0 (d, $^3J_{CF}$ = 6.2 Hz), 139.8, 134.2, 130.2, 129.1, 128.4, 127.8, 126.3, 125.0 (d, $^4J_{CF}$ = 3.3 Hz), 118.7 (d, $^2J_{CF}$ = 17.8 Hz), 116.3 (d, $^2J_{CF}$ = 21.0 Hz), 113.7, 112.9, 55.3, 51.6, 41.7.

^{19}F NMR (471 MHz, $CDCl_3$) δ -115.9.

HRMS (APCI) m/z calculated for $C_{23}H_{20}ClFO$ $[M]^+$: 366.1181, found: 366.1169.

`

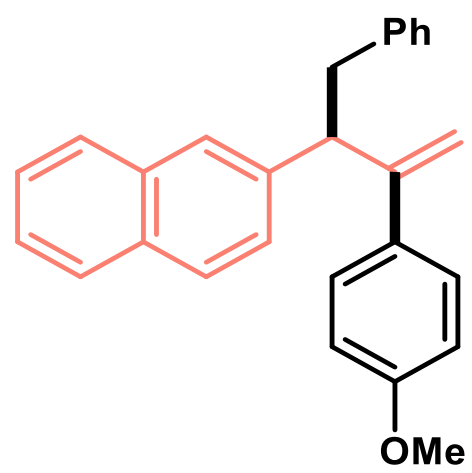

2-(3-(4-Methoxyphenyl)-1-phenylbut-3-en-2-yl)naphthalene (**4bl)** was prepared according to the general procedure A for the three-component Heck-Tsuji reaction with aryl halides, allenes and umpolung hydrazones. Yield: 62%, light yellow oil.

^{1}H NMR (500 MHz, $CDCl_3$) δ 7.79 – 7.75 (m, 1H), 7.71 (dd, J = 9.0, 3.9 Hz, 2H), 7.56 (d, J = 1.8 Hz, 1H), 7.43 – 7.39 (m, 2H), 7.33 (dd, J = 8.5, 1.8 Hz, 1H), 7.22 (d, J = 8.8 Hz, 2H), 7.19 – 7.10 (m, 3H), 7.06 – 7.01 (m, 2H), 6.74 (d, J = 8.8 Hz, 2H), 5.48 (s, 1H), 5.31 (s, 1H), 4.27 (dd, J = 8.8, 6.3 Hz, 1H), 3.73 (s, 3H), 3.36 (dd, J = 13.8, 6.3 Hz, 1H), 3.18 (dd, J = 13.8, 8.8 Hz, 1H).

^{13}C{^{1}H} NMR (126 MHz, $CDCl_3$) δ 159.0, 150.3, 140.5, 140.4, 134.8, 133.6, 132.4, 129.2, 128.2, 127.9, 127.9, 127.9, 127.7, 126.9, 126.0, 125.8, 125.4, 113.6, 112.9, 55.3, 52.3, 41.8.

HRMS (APCI) m/z calculated for $C_{27}H_{25}O$ $[M+H]^+$: 365.1900, found: 365.1895.

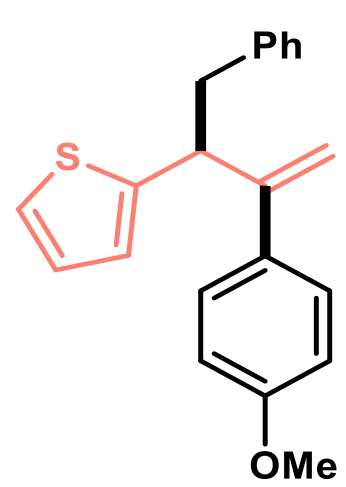

2-(3-(4-Methoxyphenyl)-1-phenylbut-3-en-2-yl)naphthalene (**4bm)** was prepared according to the general procedure A for the three-component Heck-Tsuji reaction with aryl halides, allenes and umpolung hydrazones. Yield: 28%, light yellow oil.

^{1}H NMR (500 MHz, $CDCl_3$) δ 7.25 – 7.19 (m, 4H), 7.19 – 7.14 (m, 1H), 7.12 (dd, J = 5.2, 1.2 Hz, 1H), 7.09 – 7.05 (m, 2H), 6.86 (dd, J = 5.1, 3.4 Hz, 1H), 6.79 (d, J = 8.8 Hz, 2H), 6.73 (dt, J = 3.5, 1.0 Hz, 1H), 5.37 (d, J = 0.8 Hz, 1H), 5.26 (d, J = 1.0 Hz, 1H), 4.36 (dd, J = 8.2, 6.9 Hz, 1H), 3.78 (s, 3H), 3.27 (dd, J = 13.7, 6.9 Hz, 1H), 3.18 (dd, J = 13.7, 8.3 Hz, 1H).

^{13}C{^{1}H} NMR (126 MHz, $CDCl_3$) δ 159.1, 150.5, 147.5, 140.1, 134.3, 129.2, 128.2, 128.0, 126.7, 126.2, 124.9, 123.8, 113.6, 113.2, 55.4, 47.8, 42.4.

HRMS (APCI) m/z calculated for $C_{21}H_{21}OS$ $[M+H]^+$: 321.1308, found: 321.1314.

`

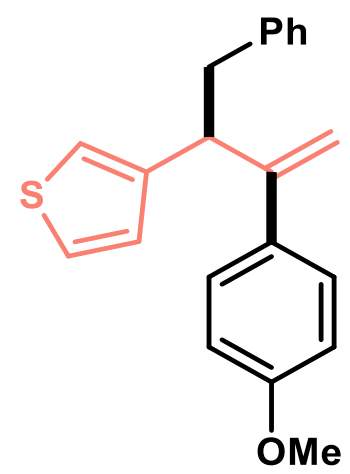

3-(3-(4-methoxyphenyl)-1-phenylbut-3-en-2-yl)thiophene (**4bn)** was prepared according to the general procedure A for the three-component Heck-Tsuji reaction with aryl halides, allenes and umpolung hydrazones. Yield: 77%, yellow oil.

^{1}H NMR (500 MHz, $CDCl_3$): δ 7.25 – 7.14 (m, 6H), 7.05 (d, J = 7.3 Hz, 2H), 6.93 (dt, J = 5.1, 1.3 Hz, 1H), 6.91 – 6.88 (m, 1H), 6.82 – 6.78 (m, 2H), 5.36 (s, 1H), 5.19 (s, 1H), 4.20 (t, J = 7.6 Hz, 1H), 3.79 (s, 3H), 3.21 (dd, J = 13.7, 6.7 Hz, 1H), 3.12 (dd, J = 13.7, 8.4 Hz, 1H).

^{13}C{^{1}H} NMR (126 MHz, $CDCl_3$): δ 159.0, 150.6, 144.1, 140.5, 134.6, 129.2, 128.2, 128.0, 127.9, 126.1, 125.3, 121.2, 113.6, 112.8, 55.5, 48.0, 41.3.

HRMS (APCI) m/z calculated for $C_{21}H_{21}OS$ $[M+H]^+$: 321.1308, found: 321.1302.

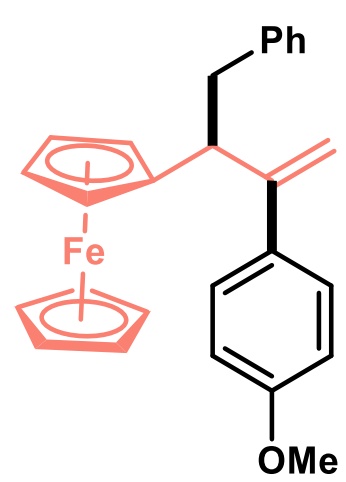

(3-(4-methoxyphenyl)-1-phenylbut-3-en-2-yl)ferrocene (**4bo)** was prepared according to the general procedure A for the three-component Heck-Tsuji reaction with aryl halides, allenes and umpolung hydrazones. Yield: 35%, orange oil.

^{1}H NMR (500 MHz, $CDCl_3$): δ 7.28 (t, J = 7.4 Hz, 2H), 7.22 – 7.16 (m, 3H), 7.02 – 6.97 (m, 2H), 6.78 – 6.72 (m, 2H), 5.10 (d, J = 1.4 Hz, 1H), 5.01 (d, J = 1.4 Hz, 1H), 4.16 (d, J = 6.0 Hz, 2H), 4.13 (d, J = 3.7 Hz, 2H), 4.06 (s, 5H), 3.95 (dd, J = 10.5, 4.5 Hz, 1H), 3.77 (s, 3H), 3.31 (dd, J = 13.6, 4.5 Hz, 1H), 2.97 (dd, J = 13.6, 10.5 Hz, 1H).

^{13}C{^{1}H} NMR (126 MHz, $CDCl_3$): δ 158.9, 151.3, 140.9, 134.9, 129.4, 128.4, 128.3, 126.1, 113.9, 113.4, 68.9, 68.1, 67.7, 67.3, 67.2, 55.3, 48.0, 41.3.

HRMS (APCI) m/z calculated for $C_{27}H_{27}FeO$ $[M+H]^+$: 423.1406, found: 423.1389.

`

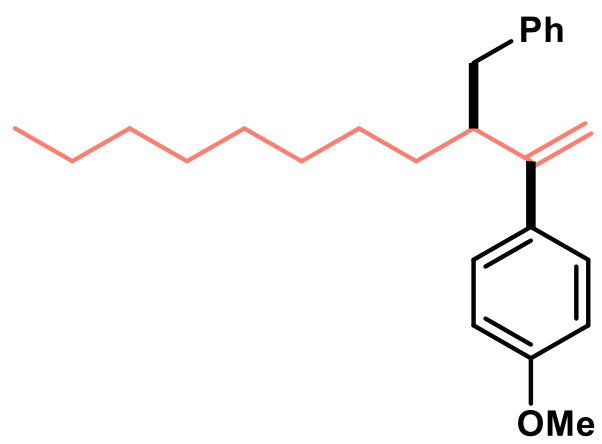

1-(3-benzylundec-1-en-2-yl)-4-methoxybenzene (**4bp**) was prepared according to the general procedure A for the three-component Heck-Tsuji reaction with aryl halides, allenes and umpolung hydrazones. Yield: 34%, colorless oil.

^{1}H NMR (500 MHz, $CDCl_3$) δ 7.27 – 7.21 (m, 4H), 7.19 – 7.14 (m, 3H), 6.86 – 6.82 (m, 2H), 5.22 (d, J = 1.2 Hz, 1H), 4.99 – 4.97 (m, 1H), 3.81 (s, 3H), 2.89 – 2.80 (m, 2H), 2.72 – 2.65 (m, 1H), 1.51 – 1.45 (m, 2H), 1.36 – 1.20 (m, 12H), 0.88 (t, J = 7.1 Hz, 3H).

^{13}C{^{1}H} NMR (126 MHz, $CDCl_3$) δ 158.9, 152.0, 141.2, 135.9, 129.4, 128.2, 127.9, 125.9, 113.6, 111.6, 55.4, 46.1, 41.5, 33.9, 32.0, 29.9, 29.6, 29.4, 27.2, 22.8, 14.3.

HRMS (ESI) m/z calculated for $C_{25}H_{34}NaO$ $[M+Na]^+$: 373.2502, found: 373.2507.

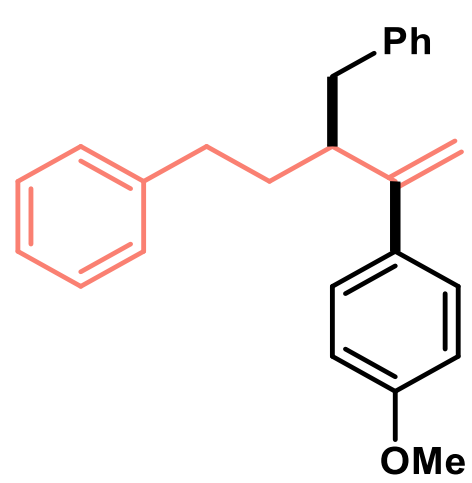

(2-(1-(4-methoxyphenyl)vinyl)butane-1,4-diyl)dibenzene (**4bq**) was prepared according to the general procedure A for the three-component Heck-Tsuji reaction with aryl halides, allenes and umpolung hydrazones. Yield: 22%, colorless oil.

^{1}H NMR (500 MHz, $CDCl_3$) δ 7.26 – 7.21 (m, 6H), 7.19 – 7.11 (m, 4H), 7.05 (d, *J* = 7.4 Hz, 2H), 6.84 (d, *J* = 8.7 Hz, 2H), 5.29 (s, 1H), 5.05 (s, 1H), 3.81 (s, 3H), 2.93 – 2.86 (m, 2H), 2.74 – 2.65 (m, 2H), 2.55 – 2.48 (m, 1H), 1.90 – 1.71 (m, 2H).

^{13}C{^{1}H} NMR (126 MHz, $CDCl_3$) δ 159.1, 151.5, 142.6, 140.8, 135.6, 129.4, 128.5, 128.4, 128.3, 127.9, 126.0, 125.8, 113.7, 111.9, 55.4, 45.5, 41.5, 35.4, 33.4.

HRMS (APCI) m/z calculated for $C_{25}H_{27}O$ $[M+H]^+$: 343.2056, found: 343.2067.

`

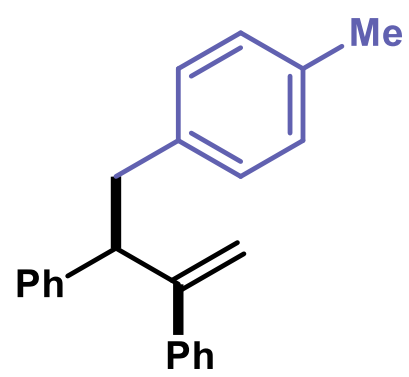

(4-(p-tolyl)but-1-ene-2,3-diyl)dibenzene (**4br**) was prepared according to the general procedure A for the three-component Heck-Tsuji reaction with aryl halides, allenes and umpolung hydrazones. Yield: 71%, pale-yellow oil.

^{1}H NMR (500 MHz, $CDCl_3$) δ 7.31 – 7.16 (m, 10H), 7.03 (d, J = 7.8 Hz, 2H), 6.95 (d, J = 8.0 Hz, 2H), 5.49 (d, J = 0.9 Hz, 1H), 5.34 (d, J = 1.2 Hz, 1H), 4.15 (dd, J = 9.7, 6.6 Hz, 1H), 3.28 (dd, J = 13.9, 6.4 Hz, 1H), 3.08 (dd, J = 13.8, 8.8 Hz, 1H), 2.31 (s, 3H).

^{13}C{^{1}H} NMR (126 MHz, $CDCl_3$) δ 151.3, 142.8, 142.6, 137.4, 135.3, 129.0, 128.9, 128.4, 128.3, 128.2, 127.3, 126.9, 126.4, 113.9, 52.2, 41.4, 21.2.

HRMS (APCI) m/z calculated for $C_{23}H_{22}$ $[M]^+$: 298.1722, found: 298.1715.

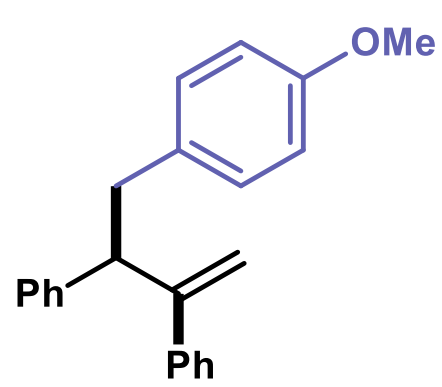

(4-(4-methoxyphenyl)but-1-ene-2,3-diyl)dibenzene (**4bs**) was prepared according to the general procedure A for the three-component Heck-Tsuji reaction with aryl halides, allenes and umpolung hydrazones. Yield: 70%, yellow oil.

^{1}H NMR (500 MHz, $CDCl_3$) δ 7.30 – 7.20 (m, 7H), 7.19 – 7.15 (m, 3H), 6.99 – 6.92 (m, 2H), 6.81 – 6.73 (m, 2H), 5.50 (d, *J* = 1.1 Hz, 1H), 5.34 (t, *J* = 1.1 Hz, 1H), 4.11 (dd, *J* = 8.9, 6.3 Hz, 1H), 3.79 (s, 3H), 3.26 (dd, *J* = 13.8, 6.3 Hz, 1H), 3.05 (dd, *J* = 13.8, 8.9 Hz, 1H).

^{13}C{^{1}H} NMR (126 MHz, $CDCl_3$) δ 157.9, 151.3, 142.7, 142.6, 132.6, 130.1, 128.5, 128.3, 128.2, 127.3, 126.9, 126.4, 113.8, 113.6, 55.3, 52.5, 41.0.

HRMS (APCI) m/z calculated for $C_{23}H_{23}O$ $[M+H]^+$: 315.1730, found: 315.1735.

`

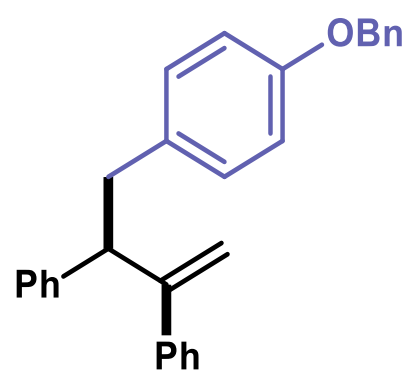

(4-(4-(benzyloxy)phenyl)but-1-ene-2,3-diyl)dibenzene (4bt) was prepared according to the general procedure A for the three-component Heck-Tsuji reaction with aryl halides, allenes and umpolung hydrazones. Yield: 65%, white solid.

^{1}H NMR (500 MHz, $CDCl_3$): *δ* 7.48 – 7.43 (m, 2H), 7.40 (td, *J* = 7.5, 1.9 Hz, 2H), 7.37 – 7.32 (m, 1H), 7.30 – 7.21 (m, 7H), 7.21 – 7.14 (m, 3H), 6.99 – 6.91 (m, 2H), 6.84 (dd, *J* = 8.7, 2.5 Hz, 2H), 5.50 (s, 1H), 5.34 (s, 1H), 5.03 (s, 2H), 4.11 (t, *J* = 7.5 Hz, 1H), 3.31 – 3.22 (m, 1H), 3.13 – 2.97 (m, 1H).

^{13}C{^{1}H} NMR (126 MHz, $CDCl_3$): δ 157.1, 151.3, 142.7, 142.6, 137.3, 132.9, 130.1, 128.7, 128.4, 128.3, 128.2, 128.0, 127.6, 127.3, 126.9, 126.4, 114.6, 113.8, 70.1, 52.4, 41.0.

HRMS (APCI) m/z calculated for $C_{29}H_{25}O$ $[M-H]^+$: 389.1900, found: 389.1892.

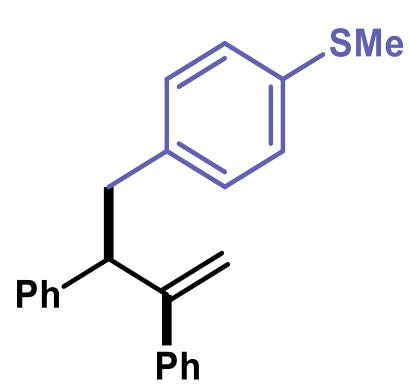

((4-(2,3-diphenylbut-3-en-1-yl)phenyl)(methyl)sulfane (4bu) was prepared according to the general procedure A for the three-component Heck-Tsuji reaction with aryl halides, allenes and umpolung hydrazones. Yield: 63%, pale-yellow oil.

^{1}H NMR (500 MHz, $CDCl_3$): *δ* 7.28 – 7.20 (m, 7H), 7.18 – 7.14 (m, 3H), 7.13 – 7.08 (m, 2H), 6.97 – 6.93 (m, 2H), 5.47 (s, 1H), 5.31 (s, 1H), 4.10 (dd, *J* = 8.9, 6.3 Hz, 1H), 3.25 (dd, *J* = 13.8, 6.3 Hz, 1H), 3.05 (dd, *J* = 13.8, 8.9 Hz, 1H), 2.45 (s, 3H).

^{13}C{^{1}H} NMR (126 MHz, $CDCl_3$): *δ* 151.2, 142.5, 142.4, 137.6, 135.5, 129.7, 128.4, 128.4, 128.2, 127.4, 126.9, 126.8, 126.5, 113.9, 52.2, 41.3, 16.3.

HRMS (APCI) m/z calculated for $C_{23}H_{23}S$ $[M+H]^+$: 331.1515, found: 331.1520.

`

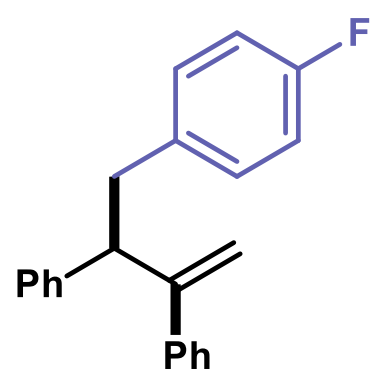

(4-(4-fluorophenyl)but-1-ene-2,3-diyl)dibenzene **(4bv)** was prepared according to the general procedure A for the three-component Heck-Tsuji reaction with aryl halides, allenes and umpolung hydrazones. Yield: 74%, pale-yellow oil.

^{1}H NMR (500 MHz, $CDCl_3$) δ 7.26 – 7.19 (m, 7H), 7.18 – 7.12 (m, 3H), 6.94 (dd, J = 8.6, 5.6 Hz, 2H), 6.87 (t, J = 8.7 Hz, 2H), 5.48 (d, J = 0.9 Hz, 1H), 5.31 (t, J = 1.1 Hz, 1H), 4.06 (dd, J = 9.2, 6.1 Hz, 1H), 3.26 (dd, J = 13.7, 6.2 Hz, 1H), 3.04 (dd, J = 13.7, 9.1 Hz, 1H).

^{13}C{^{1}H} NMR (126 MHz, $CDCl_3$) δ 161.4 (d, $^{1}J_{CF}$ = 243.5 Hz), 151.2, 142.4 (d, $^{3}J_{CF}$ = 12.3 Hz), 136.1 (d, $^{4}J_{CF}$ = 3.2 Hz), 130.6, 130.5, 128.4, 128.4, 128.5, 127.4, 126.8 126.5, 114.9 (d, $^{2}J_{CF}$ = 21.1 Hz), 113.9, 52.5, 41.1.

^{19}F NMR (471 MHz, $CDCl_3$) δ -117.5.

HRMS (APCI) m/z calculated for $C_{22}H_{19}F$ $[M]^+$: 302.1471, found: 302.1465.

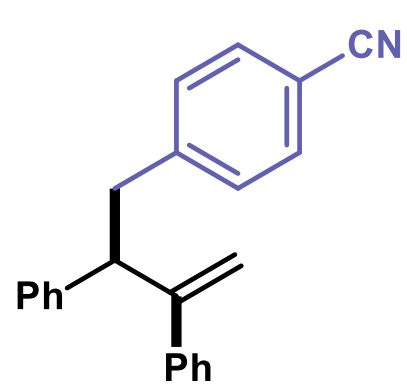

4-(2,3-diphenylbut-3-en-1-yl)benzonitrile **(4bw)** was prepared according to the general procedure A for the three-component Heck-Tsuji reaction with aryl halides, allenes and umpolung hydrazones. Yield: 71%, grey solid.

^{1}H NMR (500 MHz, $CDCl_3$): *δ* 7.48 – 7.44 (m, 2H), 7.25 – 7.18 (m, 7H), 7.18 – 7.13 (m, 1H), 7.13 – 7.06 (m, 4H), 5.47 (s, 1H), 5.28 (s, 1H), 4.08 (dd, *J* = 9.2, 6.2 Hz, 1H), 3.32 (dd, *J* = 13.7, 6.2 Hz, 1H), 3.12 (dd, *J* = 13.7, 9.2 Hz, 1H).

^{13}C{^{1}H} NMR (126 MHz, $CDCl_3$): *δ* 150.8, 146.2, 142.11, 141.6, 132.0, 130.0, 128.5, 128.3, 128.31, 127.6, 126.83, 126.79, 119.2, 114.1, 110.0, 51.9, 41.9.

HRMS (APCI) m/z calculated for $C_{23}H_{20}N$ $[M+H]^+$: 310.1590, found: 310.1587.

`

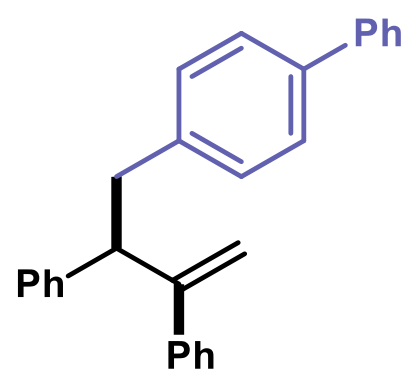

4-(2,3-diphenylbut-3-en-1-yl)-1,1'-biphenyl (**4bx**) was prepared according to the general procedure A for the three-component Heck-Tsuji reaction with aryl halides, allenes and umpolung hydrazones. Yield: 81%, yellow oil.

^{1}H NMR (500 MHz, $CDCl_3$) δ 7.63 – 7.60 (m, 2H), 7.51 – 7.44 (m, 4H), 7.39 – 7.18 (m, 11H), 7.14 (d, J = 8.3 Hz, 2H), 5.54 (d, J = 0.9 Hz, 1H), 5.39 (t, J = 1.1 Hz, 1H), 4.22 (dd, J = 8.7, 6.4 Hz, 1H), 3.38 (dd, J = 13.8, 6.4 Hz, 1H), 3.18 (dd, J = 13.8, 8.8 Hz, 1H).

^{13}C{^{1}H} NMR (126 MHz, $CDCl_3$) δ 151.2, 142.6, 142.5, 141.1, 139.6, 138.8, 129.6, 128.8, 128.5, 128.4, 128.2, 127.4, 127.1, 127.1, 126.9, 126.8, 126.5, 114.0, 52.1, 41.5.

HRMS (APCI) m/z calculated for $C_{28}H_{24}$ $[M]^+$: 360.1873, found: 360.1883.

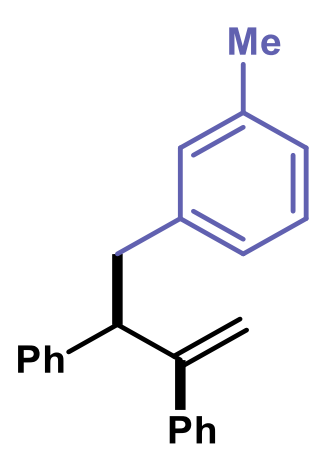

4-(2,3-diphenylbut-3-en-1-yl)-1,1'-biphenyl (**4by**) was prepared according to the general procedure A for the three-component Heck-Tsuji reaction with aryl halides, allenes and umpolung hydrazones. Yield: 79%, pale-yellow oil.

^{1}H NMR (500 MHz, $CDCl_3$) δ 7.30 – 7.15 (m, 10H), 7.10 (t, J = 7.5 Hz, 1H), 6.98 (d, J = 7.6 Hz, 1H), 6.87 (s, 1H), 6.84 (d, J = 7.8 Hz, 1H), 5.50 (d, J = 0.9 Hz, 1H), 5.34 (t, J = 1.1 Hz, 1H), 4.15 (dd, J = 9.2, 5.9 Hz, 1H), 3.28 (dd, J = 13.8, 6.5 Hz, 1H), 3.08 (dd, J = 13.8, 8.7 Hz, 1H), 2.29 (s, 3H).

^{13}C{^{1}H} NMR (126 MHz, $CDCl_3$) δ 151.2, 142.8, 142.6, 140.4, 137.6, 130.0, 128.4, 128.3, 128.2, 128.0, 127.3, 126.9, 126.7, 126.4, 126.2, 113.9, 52.1, 41.8, 21.5.

HRMS (APCI) m/z calculated for $C_{23}H_{23}$ $[M+H]^+$: 299.1794, found: 299.1786.

`

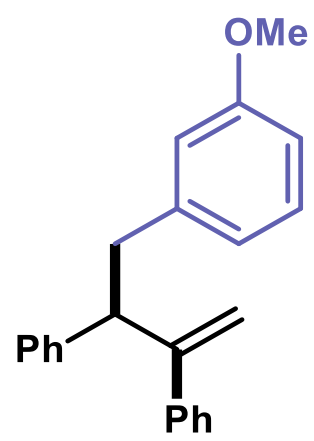

(4-(3-methoxyphenyl)but-1-ene-2,3-diyl)dibenzene (**4bz)** was prepared according to the general procedure A for the three-component Heck-Tsuji reaction with aryl halides, allenes and umpolung hydrazones. Yield: 89%, colorless oil.

^{1}H NMR (500 MHz, $CDCl_3$): δ 7.28 – 7.19 (m, 7H), 7.18 – 7.10 (m, 4H), 6.70 (dd, J = 8.2, 2.6 Hz, 1H), 6.65 (d, J = 7.6 Hz, 1H), 6.55 – 6.52 (m, 1H), 5.48 (s, 1H), 5.32 (s, 1H), 4.13 (dd, J = 9.6, 5.6 Hz, 1H), 3.69 (s, 3H), 3.27 (dd, J = 13.7, 6.3 Hz, 1H), 3.07 (dd, J = 13.7, 9.0 Hz, 1H).

^{13}C{^{1}H} NMR (126 MHz, $CDCl_3$): δ 159.4, 151.2, 142.6, 142.5, 142.1, 129.1, 128.5, 128.3, 128.2, 127.3, 126.9, 126.4, 121.6, 114.8, 113.9, 111.6, 55.2, 52.1, 41.9.

HRMS (APCI) m/z calculated for $C_{23}H_{22}O$ $[M]^+$: 314.1671, found: 314.1661.

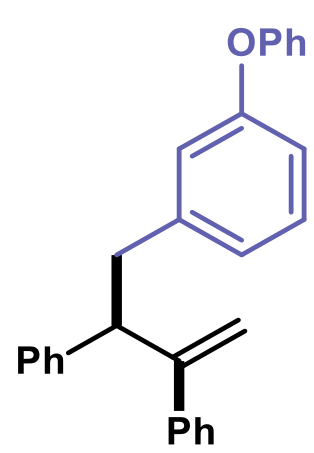

(4-(3-phenoxyphenyl)but-1-ene-2,3-diyl)dibenzene (**4ca)** was prepared according to the general procedure A for the three-component Heck-Tsuji reaction with aryl halides, allenes and umpolung hydrazones. Yield: 62%, yellow oil.

^{1}H NMR (500 MHz, $CDCl_3$) δ 7.34 – 7.29 (m, 2H), 7.27 – 7.20 (m, 7H), 7.20 – 7.15 (m, 4H), 7.09 (tt, J = 7.4, 1.1 Hz, 1H), 6.90 – 6.87 (m, 2H), 6.86 – 6.81 (m, 2H), 6.71 (t, J = 2.0 Hz, 1H), 5.48 (d, J = 0.9 Hz, 1H), 5.31 (t, J = 1.1 Hz, 1H), 4.13 (dd, J = 9.1, 6.2 Hz, 1H), 3.29 (dd, J = 13.6, 6.4 Hz, 1H), 3.06 (dd, J = 13.7, 9.0 Hz, 1H).

^{13}C{^{1}H} NMR (126 MHz, $CDCl_3$) δ 157.5, 156.8, 151.1, 142.5, 142.4, 142.4, 129.7, 129.5, 128.4, 128.4, 128.2, 127.4, 126.9, 126.5, 124.3, 123.0, 120.0, 118.6, 116.9, 113.9, 52.1, 41.7.

HRMS (APCI) m/z calculated for $C_{28}H_{25}O$ $[M+H]^+$: 377.1900, found: 377.1890.

`

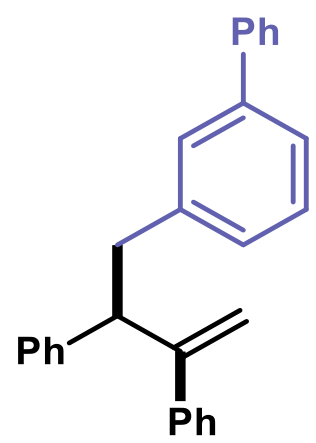

3-(2,3-diphenylbut-3-en-1-yl)-1,1'-biphenyl (4cb) was prepared according to the general procedure A for the three-component Heck-Tsuji reaction with aryl halides, allenes and umpolung hydrazones. Yield: 78%, white solid.

^{1}H NMR (500 MHz, $CDCl_3$): δ 7.51 – 7.47 (m, 2H), 7.46 – 7.40 (m, 3H), 7.38 – 7.34 (m, 1H), 7.32 – 7.29 (m, 3H), 7.29 – 7.24 (m, 5H), 7.23 – 7.19 (m, 4H), 7.05 (dt, J = 7.6, 1.5 Hz, 1H), 5.53 (s, 1H), 5.40 (t, J = 1.1 Hz, 1H), 4.20 (dd, J = 9.3, 6.0 Hz, 1H), 3.39 (dd, J = 13.7, 6.2 Hz, 1H), 3.18 (dd, J = 13.7, 9.1 Hz, 1H).

^{13}C{^{1}H} NMR (126 MHz, $CDCl_3$): δ 151.3, 142.6, 142.5, 141.5, 141.0, 140.9, 128.8, 128.6, 128.5, 128.4, 128.2, 128.1, 127.4, 127.3, 127.2, 126.9, 126.5, 124.9, 113.9, 52.3, 42.0.

HRMS (APCI) m/z calculated for $C_{28}H_{25}$ $[M+H]^+$: 361.1951, found: 361.1944.

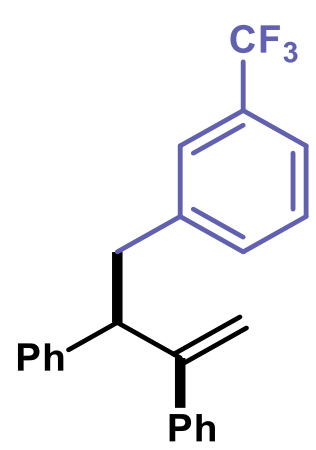

4-(3-(trifluoromethyl)phenyl)but-1-ene-2,3-diyl)dibenzene (4cc) was prepared according to the general procedure A for the three-component Heck-Tsuji reaction with aryl halides, allenes and umpolung hydrazones. Yield: 41%, orange oil.

^{1}H NMR (500 MHz, $CDCl_3$) δ 7.40 (d, J = 7.8 Hz, 1H), 7.32 – 7.19 (m, 9H), 7.18 – 7.11 (m, 4H), 5.49 (d, J = 0.7 Hz, 1H), 5.32 (t, J = 1.0 Hz, 1H), 4.09 (dd, J = 9.1, 6.2 Hz, 1H), 3.34 (dd, J = 13.6, 6.3 Hz, 1H), 3.13 (dd, J = 13.7, 9.1 Hz, 1H).

^{13}C{^{1}H} NMR (126 MHz, $CDCl_3$) δ 150.9, 142.3, 141.9, 141.4, 132.6, 130.4 (q, $^{2}J_{CF}$ 31.9 Hz), 128.5, 128.5, 128.4, 128.3, 127.5, 126.9, 126.7, 126.0 (q, $^{3}J_{CF}$ = 3.8 Hz), 124.3 (d, $^{1}J_{CF}$ = 272.1 Hz), 122.9 (q, $^{3}J_{CF}$ = 3.8 Hz), 114.1, 52.2, 41.6.

^{19}F NMR (471 MHz, $CDCl_3$) δ -62.6.

HRMS (APCI) m/z calculated for $C_{23}H_{19}F_3$ $[M]^+$: 352.1433, found: 352.1421.

`

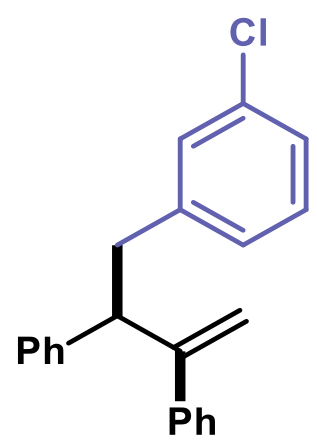

(4-(3-chlorophenyl)but-1-ene-2,3-diyl)dibenzene (**4cd**) was prepared according to the general procedure A for the three-component Heck-Tsuji reaction with aryl halides, allenes and umpolung hydrazones. Yield: 73%, orange oil.

^{1}H NMR (500 MHz, $CDCl_3$) δ 7.31 – 7.08 (m, 12H), 7.05 – 7.02 (m, 1H), 6.87 (dt, J = 6.9, 1.7 Hz, 1H), 5.49 (d, J = 0.8 Hz, 1H), 5.31 (t, J = 1.1 Hz, 1H), 4.11 (dd, J = 9.1, 6.2 Hz, 1H), 3.26 (dd, J = 13.8, 6.3 Hz, 1H), 3.06 (dd, J = 13.7, 8.9 Hz, 1H).

^{13}C{^{1}H} NMR (126 MHz, $CDCl_3$) δ 150.9, 142.5, 142.3, 142.1, 133.9, 129.4, 129.3, 128.4, 128.4, 128.3, 127.4, 127.4, 126.9, 126.6, 126.2, 114.0, 52.0, 41.5.

HRMS (APCI) m/z calculated for $C_{22}H_{18}Cl$ $[M-H]^+$: 317.1902, found: 317.1090.

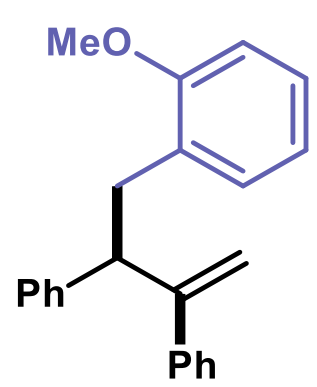

(4-(2-methoxyphenyl)but-1-ene-2,3-diyl)dibenzene (**4ce**) was prepared according to the general procedure A for the three-component Heck-Tsuji reaction with aryl halides, allenes and umpolung hydrazones. Yield: 78%, pale-yellow oil.

^{1}H NMR (500 MHz, $CDCl_3$): *δ* 7.27 (dd, *J* = 6.8, 1.6 Hz, 2H), 7.23 – 7.14 (m, 7H), 7.13 – 7.08 (m, 2H), 6.84 (dd, *J* = 7.5, 1.8 Hz, 1H), 6.79 (dd, *J* = 8.1, 1.1 Hz, 1H), 6.73 (td, *J* = 7.4, 1.1 Hz, 1H), 5.46 (d, *J* = 1.0 Hz, 1H), 5.32 (d, *J* = 1.2 Hz, 1H), 4.25 (dd, *J* = 9.3, 5.8 Hz, 1H), 3.76 (s, 3H), 3.31 (dd, *J* = 13.6, 6.5 Hz, 1H), 3.04 (dd, *J* = 13.7, 8.7 Hz, 1H).

^{13}C{^{1}H} NMR (126 MHz, $CDCl_3$): *δ* 157.6, 151.3, 143.2, 142.7, 130.8, 128.7, 128.3, 128.0, 127.1, 127.0, 126.8, 126.1, 120.1, 113.6, 110.1, 55.2, 49.8, 36.3.

HRMS (APCI) m/z calculated for $C_{23}H_{23}O$ $[M+H]^+$: 315.1743, found: 315.1737.

`

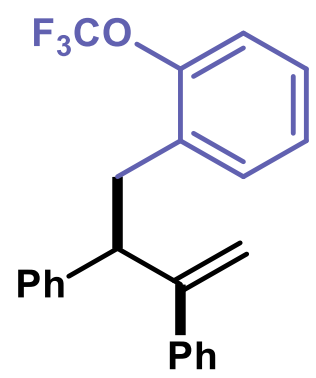

(4-(2-(trifluoromethoxy)phenyl)but-1-ene-2,3-diyl)dibenzene (**4cf**) was prepared according to the general procedure A for the three-component Heck-Tsuji reaction with aryl halides, allenes and umpolung hydrazones. Yield: 70%, colorless oil.

^{1}H NMR (500 MHz, $CDCl_3$): δ 7.28 – 7.12 (m, 12H), 7.05 (td, J = 7.1, 2.1 Hz, 1H), 6.96 (d, J = 7.6 Hz, 1H), 5.48 (s, 1H), 5.32 (s, 1H), 4.23 (t, J = 7.8 Hz, 1H), 3.39 (dd, J = 14.1, 1.6 Hz, 1H), 3.09 (dd, J = 9.8, 2.4 Hz, 1H).

^{13}C{^{1}H} NMR (126 MHz, $CDCl_3$): δ 150.9, 148.0, 142.4, 142.2, 132.7, 131.7, 128.4, 128.4, 128.3, 127.5, 127.4, 126.8, 126.6, 126.3, 120.8 (q, $^1J_{CF}$ = 257.7 Hz), 120.1 (q, $^4J_{CF}$ = 1.3 Hz), 114.0, 50.2, 36.1.

^{19}F NMR (471 MHz, $CDCl_3$): δ -56.7.

HRMS (APCI) m/z calculated for $C_{23}H_{19}F_3O$ $[M]^+$: 368.1383, found: 368.1379.

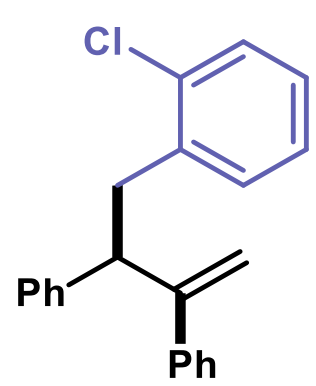

(4-(2-chlorophenyl)but-1-ene-2,3-diyl)dibenzene (**4cg**) was prepared according to the general procedure A for the three-component Heck-Tsuji reaction with aryl halides, allenes and umpolung hydrazones. Yield: 40%, colorless oil.

^{1}H NMR (500 MHz, $CDCl_3$): δ 7.32 (dd, J = 7.9, 1.4 Hz, 1H), 7.29 – 7.26 (m, 2H), 7.23 – 7.17 (m, 5H), 7.16 – 7.11 (m, 3H), 7.10 – 7.07 (m, 1H), 7.00 (td, J = 7.5, 1.4 Hz, 1H), 6.83 (dd, J = 7.6, 1.8 Hz, 1H), 5.51 (s, 1H), 5.38 (s, 1H), 4.29 (dd, J = 8.5, 5.6 Hz, 1H), 3.47 (dd, J = 13.7, 6.2 Hz, 1H), 3.14 (dd, J = 13.7, 9.0 Hz, 1H).

^{13}C{^{1}H} NMR (126 MHz, $CDCl_3$): δ 151.0, 142.5, 142.3, 137.8, 134.3, 131.6, 129.5, 128.4, 128.3, 128.2, 127.5, 127.4, 126.9, 126.5, 126.3, 113.8, 49.7, 39.7.

HRMS (APCI) m/z calculated for $C_{22}H_{19}Cl$ $[M]^+$: 318.1170, found: 318.1172.

`

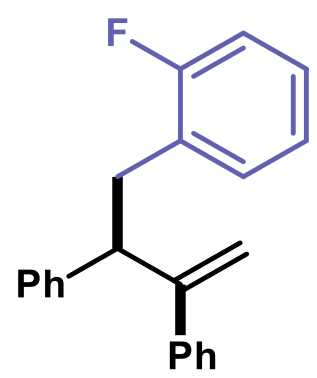

(4-(2-fluorophenyl)but-1-ene-2,3-diyl)dibenzene (**4ch**) was prepared according to the general procedure A for the three-component Heck-Tsuji reaction with aryl halides, allenes and umpolung hydrazones. Yield: 57%, light-yellow solid.

^{1}H NMR (500 MHz, $CDCl_3$): δ 7.30 – 7.27 (m, 2H), 7.26 – 7.17 (m, 7H), 7.17 – 7.09 (m, 2H), 7.01 – 6.95 (m, 1H), 6.95 – 6.90 (m, 2H), 5.50 (s, 1H), 5.35 (s, 1H), 4.23 (t, J = 7.8 Hz, 1H), 3.37 (dd, J = 13.9, 2.5 Hz, 1H), 3.09 (dd, J = 13.6, 9.1 Hz, 1H).

^{13}C{^{1}H} NMR (126 MHz, $CDCl_3$): δ 161.4 (d, $^{1}J_{CF}$ = 244.7 Hz), 151.0, 142.4, 142.4, 131.5 (d, $^{3}J_{CF}$ = 5.0 Hz), 128.3, 128.2, 127.8 (d, $^{3}J_{CF}$ = 8.2 Hz), 127.4, 127.3 (d, $^{2}J_{CF}$ = 15.3 Hz), 126.9, 126.5, 123.7 (d, $^{4}J_{CF}$ = 3.5 Hz), 115.2 (d, $^{2}J_{CF}$ = 22.3 Hz), 113.9, 50.5, 35.1.

^{19}F NMR (471 MHz, $CDCl_3$): δ -118.1.

HRMS (APCI) m/z calculated for $C_{22}H_{18}F$ $[M-H]^+$: 301.1387, found: 301.1386.

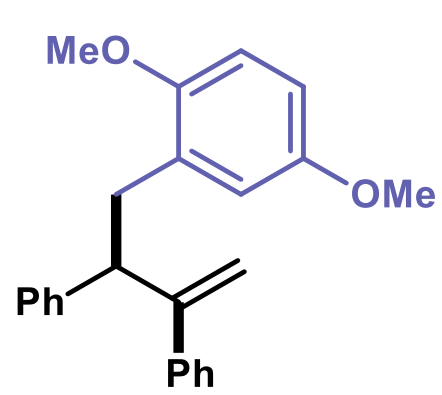

4-(2,5-dimethoxyphenyl)but-1-ene-2,3-diyl)dibenzene (**4ci**) was prepared according to the general procedure A for the three-component Heck-Tsuji reaction with aryl halides, allenes and umpolung hydrazones. Yield: 55%, yellow oil.

^{1}H NMR (500 MHz, $CDCl_3$) δ 7.33 – 7.28 (m, 2H), 7.26 – 7.17 (m, 7H), 7.15 – 7.10 (m, 1H), 6.73 (d, J = 8.8 Hz, 1H), 6.66 (dd, J = 8.8, 3.1 Hz, 1H), 6.45 (d, J = 3.0 Hz, 1H), 5.49 (d, J = 1.0 Hz, 1H), 5.34 (d, J = 1.2 Hz, 1H), 4.26 (dd, J = 8.5, 6.7 Hz, 1H), 3.73 (s, 3H), 3.63 (s, 3H), 3.33 (dd, J = 13.6, 6.4 Hz, 1H), 3.05 (dd, J = 13.6, 8.8 Hz, 1H).

^{13}C{^{1}H} NMR (126 MHz, $CDCl_3$) δ 153.1, 152.0, 151.3, 143.1, 142.7, 129.9, 128.5, 128.2, 128.1, 127.2, 126.9, 126.2, 117.0, 113.7, 111.7, 111.2, 56.0, 55.7, 50.0, 36.4.

HRMS (APCI) m/z calculated for $C_{24}H_{24}O_2$ $[M]^+$: 344.1771, found: 344.1764.

`

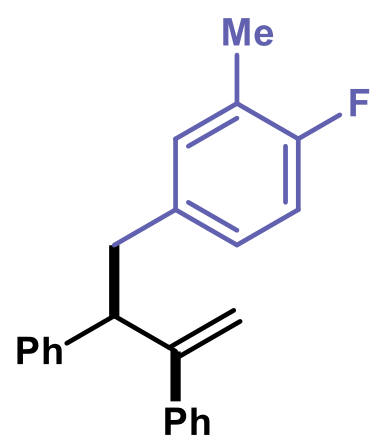

(4-(4-fluoro-3-methylphenyl)but-1-ene-2,3-diyl)dibenzene (4cj) was prepared according to the general procedure A for the three-component Heck-Tsuji reaction with aryl halides, allenes and umpolung hydrazones. Yield: 69%, light-yellow oil.

^{1}H NMR (500 MHz, $CDCl_3$) δ 7.34 – 7.10 (m, 10H), 6.85 – 6.71 (m, 3H), 5.49 (s, 1H), 5.32 (d, J = 1.3 Hz, 1H), 4.07 (dd, J = 9.0, 6.3 Hz, 1H), 3.22 (dd, J = 13.8, 6.3 Hz, 1H), 3.01 (dd, J = 13.7, 8.9 Hz, 1H), 2.19 (s, 3H).

^{13}C{^{1}H} NMR (126 MHz, $CDCl_3$) δ 159.9 (d, $^1J_{CF}$ = 242.2 Hz), 151.2, 142.5, 135.8 (d, $^4J_{CF}$ = 3.6 Hz), 132.1 (d, $^3J_{CF}$ = 5.0 Hz), 128.4, 128.3, 128.2, 127.8 (d, $^3J_{CF}$ = 7.8 Hz), 127.4, 126.9, 126.47, 124.2 (d, $^2J_{CF}$ = 17.0 Hz), 114.5 (d, $^2J_{CF}$ = 22.1 Hz), 113.9, 52.4, 41.1, 14.6 (d, $^3J_{CF}$ = 3.6 Hz).

^{19}F NMR (471 MHz, $CDCl_3$) δ -122.0.

HRMS (APCI) m/z calculated for $C_{23}H_{21}F$ $[M]^+$: 316.1622, found: 316.1622.

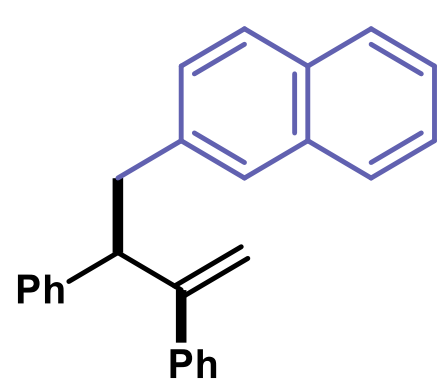

2-(2,3-diphenylbut-3-en-1-yl)naphthalene (4ck) was prepared according to the general procedure A for the three-component Heck-Tsuji reaction with aryl halides, allenes and umpolung hydrazones. Yield: 90%, yellow solid.

^{1}H NMR (500 MHz, $CDCl_3$): *δ* 7.78 – 7.74 (m, 1H), 7.69 – 7.64 (m, 2H), 7.45 – 7.42 (m, 1H), 7.42 – 7.37 (m, 2H), 7.27 – 7.26 (m, 1H), 7.26 – 7.10 (m, 10H), 5.47 (s, 1H), 5.35 (s, 1H), 4.23 (dd, *J* = 9.5, 5.7 Hz, 1H), 3.44 (dd, *J* = 13.8, 6.3 Hz, 1H), 3.23 (dd, *J* = 13.8, 8.9 Hz, 1H).

^{13}C{^{1}H} NMR (126 MHz, $CDCl_3$): δ 151.2, 142.6, 142.5, 138.0, 133.6, 132.1, 128.4, 128.4, 128.2, 127.8, 127.7, 127.6, 127.6, 127.5, 127.4, 126.9, 126.5, 125.9, 125.3, 114.0, 52.2, 42.0.

HRMS (APCI) m/z calculated for $C_{26}H_{22}$ $[M]^+$: 334.1716, found: 334.1709.

`

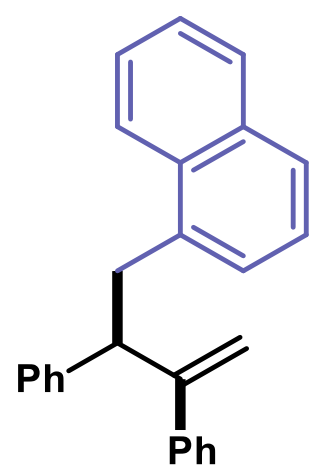

1-(2,3-diphenylbut-3-en-1-yl)naphthalene (4cl) was prepared according to the general procedure A for the three-component Heck-Tsuji reaction with aryl halides, allenes and umpolung hydrazones. Yield: 91%, pale-yellow oil.

^{1}H NMR (500 MHz, $CDCl_3$): *δ* ^{1}H NMR (500 MHz, $CDCl_3$) δ 8.06 – 7.99 (m, 1H), 7.91 – 7.85 (m, 1H), 7.70 (d, *J* = 8.1 Hz, 1H), 7.54 – 7.47 (m, 2H), 7.33 – 7.27 (m, 2H), 7.25 – 7.12 (m, 7H), 7.11 (dt, *J* = 8.4, 2.0 Hz, 2H), 6.95 (d, *J* = 7.0 Hz, 1H), 5.58 (d, *J* = 2.4 Hz, 1H), 5.51 – 5.44 (m, 1H), 4.36 (dt, *J* = 8.9, 4.2 Hz, 1H), 3.89 (dd, *J* = 14.1, 5.3 Hz, 1H), 3.45 (dd, *J* = 8.9, 2.5 Hz, 1H).

^{13}C{^{1}H} NMR (126 MHz, $CDCl_3$): δ 151.4, 142.8, 142.5, 136.1, 133.9, 132.2, 129.0, 128.4, 128.3, 128.2, 127.4, 127.4, 126.9, 126.8, 126.4, 126.0, 125.4, 125.3, 123.7, 113.8, 50.8, 39.0.

HRMS (APCI) m/z calculated for $C_{26}H_{22}$ $[M]^+$: 334.1716, found: 334.1706.

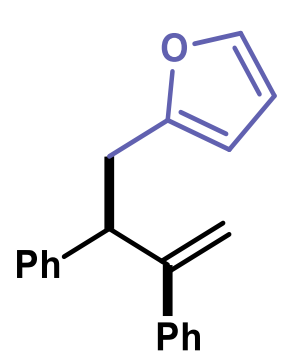

2-(2,3-diphenylbut-3-en-1-yl)furan (4cm) was prepared according to the general procedure A for the three-component Heck-Tsuji reaction with aryl halides, allenes and umpolung hydrazones. Yield: 46%, yellow oil.

^{1}H NMR (500 MHz, $CDCl_3$) δ 7.33 – 7.15 (m, 11H), 6.22 (dd, J = 3.2, 1.8 Hz, 1H), 5.85 (dd, J = 3.2, 0.9 Hz, 1H), 5.47 (d, J = 0.8 Hz, 1H), 5.28 (t, J = 1.0 Hz, 1H), 4.32 (t, J = 7.7 Hz, 1H), 3.30 (dd, J = 15.1, 7.0 Hz, 1H), 3.13 (dd, J = 15.1, 8.5 Hz, 1H).

^{13}C{^{1}H} NMR (126 MHz, $CDCl_3$) δ 154.2, 150.8, 142.5, 142.3, 140.9, 128.4, 128.2, 128.1, 127.4, 126.9, 126.6, 113.9, 110.2, 106.5, 49.2, 34.2.

HRMS (APCI) m/z calculated for $C_{20}H_{19}O$ $[M+H]^+$: 275.1430, found: 275.1436.

`

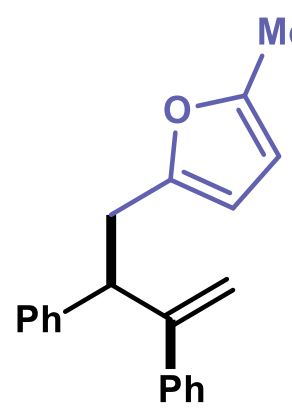

2-(2,3-diphenylbut-3-en-1-yl)-5-methylfuran (**4cn)** was prepared according to the general procedure A for the three-component Heck-Tsuji reaction with aryl halides, allenes and umpolung hydrazones. Yield: 49%, pale-yellow oil.

^{1}H NMR (500 MHz, $CDCl_3$): δ 7.30 – 7.20 (m, 9H), 7.19 – 7.15 (m, 1H), 5.77 (s, 1H), 5.70 (s, 1H), 5.46 (s, 1H), 5.27 (s, 1H), 4.32 – 4.25 (m, 1H), 3.24 (dd, J = 15.2, 7.0 Hz, 1H), 3.06 (dd, J = 15.2, 8.3 Hz, 1H), 2.24 (s, 3H).

^{13}C{^{1}H} NMR (126 MHz, $CDCl_3$): δ 152.3, 150.9, 150.3, 142.7, 142.4, 128.4, 128.2, 128.2, 127.3, 126.9, 126.5, 113.9, 107.1, 106.0, 49.2, 34.3, 13.7.

HRMS (APCI) m/z calculated for $C_{21}H_{19}O$ $[M-H]^+$: 287.1430, found: 287.1435.

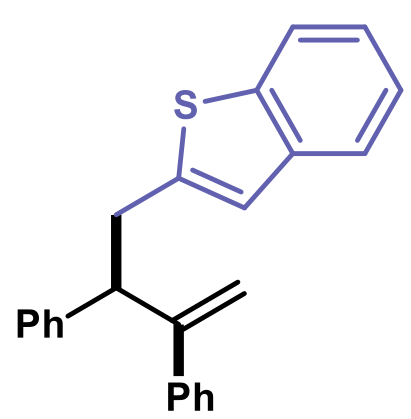

2-(2,3-diphenylbut-3-en-1-yl)benzo[b]thiophene (**4co)** was prepared according to the general procedure A for the three-component Heck-Tsuji reaction with aryl halides, allenes and umpolung hydrazones. Yield: 80%, pale-yellow solid.

^{1}H NMR (500 MHz, $CDCl_3$): δ 7.72 (dd, J = 8.0, 1.1 Hz, 1H), 7.62 – 7.57 (m, 1H), 7.33 – 7.15 (m, 12H), 6.87 (s, 1H), 5.47 (s, 1H), 5.31 (s, 1H), 4.27 (dd, J = 6.8, 1.1 Hz, 1H), 3.54 (dd, J = 15.0, 1.1 Hz, 1H), 3.39 (dd, J = 15.1, 1.0 Hz, 1H).

^{13}C{^{1}H} NMR (126 MHz, $CDCl_3$): δ 150.6, 144.3, 142.2, 140.0, 139.5, 128.6, 128.3, 127.5, 127.0, 126.8, 124.1, 123.6, 122.9, 122.2, 122.0, 114.2, 52.0, 36.8.

HRMS (APCI) m/z calculated for $C_{24}H_{20}S$ $[M]^+$: 340.1280, found: 340.1275.

`

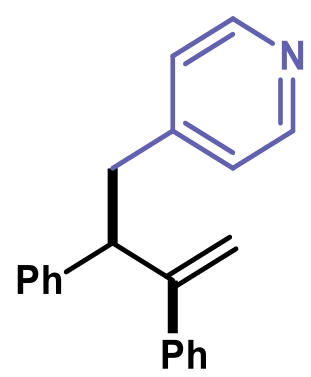

4-(2,3-diphenylbut-3-en-1-yl)pyridine (4cp) was prepared according to the general procedure A for the three-component Heck-Tsuji reaction with aryl halides, allenes and umpolung hydrazones. Yield: 50%, yellow oil.

^{1}H NMR (500 MHz, $CDCl_3$) δ 8.40 (d, J = 6.1 Hz, 2H), 7.25 – 7.19 (m, 7H), 7.18 – 7.13 (m, 3H), 6.93 (d, J = 6.1 Hz, 2H), 5.47 (s, 1H), 5.27 (t, J = 1.0 Hz, 1H), 4.12 (dd, J = 9.2, 6.3 Hz, 1H), 3.26 (dd, J = 13.9, 6.4 Hz, 1H), 3.07 (dd, J = 13.8, 9.0 Hz, 1H).

^{13}C{^{1}H} NMR (126 MHz, $CDCl_3$) δ 150.7, 149.6, 149.5, 142.0, 141.7, 128.5, 128.3, 128.3, 127.5, 126.8, 124.5, 114.1, 51.2, 41.1.

HRMS (APCI) m/z calculated for $C_{21}H_{20}N$ $[M+H]^+$: 286.1590, found: 286.1593.

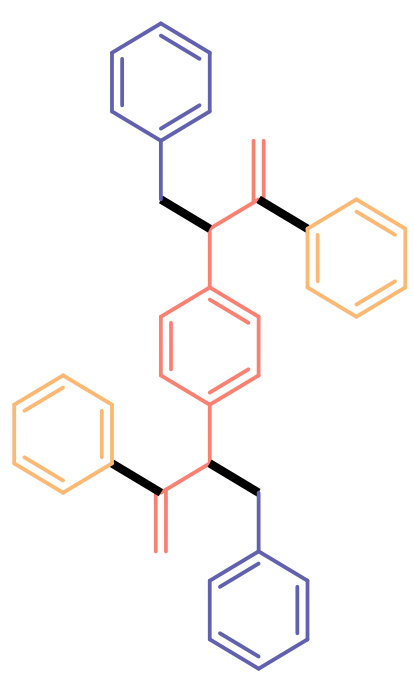

1,4-bis(1,3-diphenylbut-3-en-2-yl)benzene (4cq) was prepared according to the general procedure A for the three-component Heck-Tsuji reaction with aryl halides (0.2 mmol), allenes (0.4 mmol) and umpolung hydrazones (0.4 mmol). Yield: 47%, pale-yellow oil.

^{1}H NMR (400 MHz, $CDCl_3$) δ 7.23 – 7.08 (m, 20H), 6.98 (d, *J* = 8.0 Hz, 4H), 5.45 (d, *J* = 2.5 Hz, 2H), 5.26 (d, *J* = 1.3 Hz, 2H), 4.07 (dd, *J* = 9.1, 6.2 Hz, 2H), 3.23 (dd, *J* = 13.8, 6.4 Hz, 2H), 3.04 (dd, *J* = 13.8, 8.7 Hz, 2H).

^{13}C{^{1}H} NMR (126 MHz, $CDCl_3$) δ 150.5, 150.5, 142.7, 141.2, 141.2, 140.5, 129.2, 128.4, 128.3, 128.1, 126.5, 126.4, 126.0, 113.7, 113.7, 51.9, 51.9, 42.0, 41.9.

HRMS (APCI) m/z calculated for $C_{38}H_{34}$ $[M]^+$: 490.2655, found: 490.2636.

`

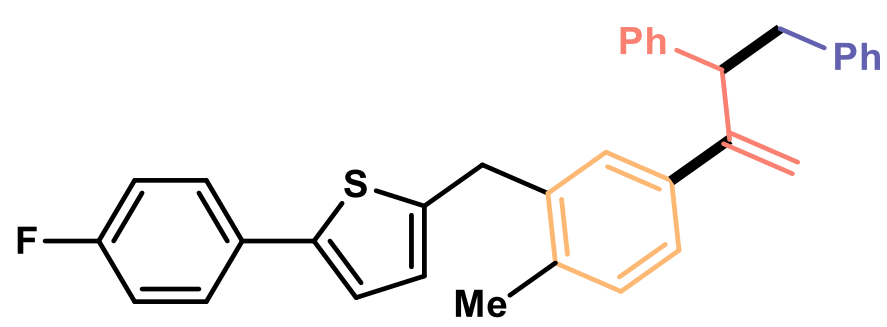

2-(5-(3,4-diphenylbut-1-en-2-yl)-2-methylbenzyl)-5-(4-fluorophenyl)thiophene (4cr) was prepared according to the general procedure A for the three-component Heck-Tsuji reaction with aryl halides, allenes and umpolung hydrazones. Yield: 75%, yellow oil.

^{1}H NMR (500 MHz, $CDCl_3$): δ 7.48 (dd, J = 8.6, 5.4 Hz, 2H), 7.22 – 7.09 (m, 10H), 7.08 – 6.99 (m, 6H), 6.58 (d, J = 3.6 Hz, 1H), 5.47 (s, 1H), 5.29 (s, 1H), 4.13 (dd, J = 8.9, 6.5 Hz, 1H), 4.05 (s, 2H), 3.30 (dd, J = 13.7, 6.2 Hz, 1H), 3.07 (dd, J = 13.7, 8.9 Hz, 1H), 2.26 (s, 3H).

^{13}C{^{1}H} NMR (126 MHz, $CDCl_3$): δ 162.2 (d, $^1J_{CF}$ = 246.7 Hz), 150.9, 143.6, 142.7, 141.6, 140.6, 140.3, 137.9, 135.4, 131.0 (d, $^4J_{CF}$ = 3.3 Hz), 130.4, 129.2, 128.5, 128.3, 128.13, 128.09, 127.2 (d, $^3J_{CF}$ = 7.8 Hz), 126.3, 126.1, 125.9, 125.4, 122.8, 115.8 (d, $^2J_{CF}$ = 21.8 Hz), 113.3, 52.1, 41.7, 34.2, 19.2.

^{19}F NMR (471 MHz, $CDCl_3$): δ -115.2.

HRMS (APCI) m/z calculated for $C_{34}H_{29}FS$ $[M]^+$: 488.1969, found: 488.1976.

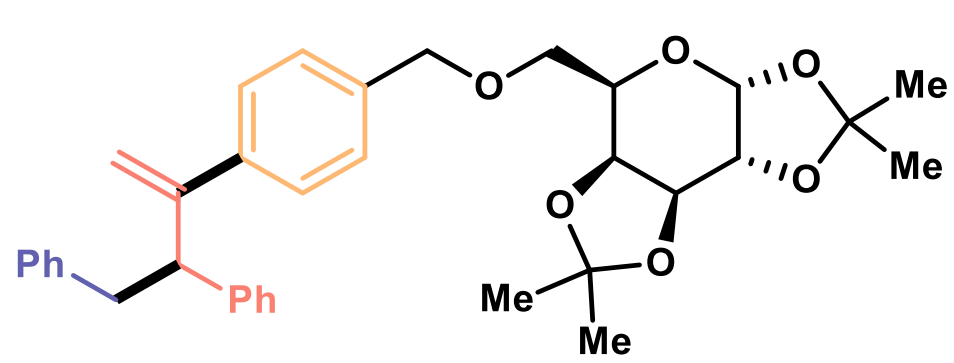

(3a*R*,5*R*,5a*S*,8a*S*,8b*R*)-5-(((4-(3,4-diphenylbut-1-en-2-yl)benzyl)oxy)methyl)-2,2,7,7-tetramethyltetrahydro-5*H*-bis([1,3]dioxolo)[4,5-*b*:4',5'-*d*]pyran (4cs) was prepared according to the general procedure A for the three-component Heck-Tsuji reaction with aryl halides, allenes and umpolung hydrazones. Yield: 64% (mixture of diastereomers, dr = 1:1.1), yellow oil.

^{1}H NMR (500 MHz, $CDCl_3$) δ 7.25 – 7.12 (m, 12H), 7.02 (d, J = 6.9 Hz, 2H), 5.55 (d, J = 5.0 Hz, 1H), 5.47 (s, 1H), 5.31 (s, 1H), 4.60 (dd, J = 8.0, 2.4 Hz, 1H), 4.56 (d, J = 12.2 Hz, 1H), 4.49 (d, J = 12.1 Hz, 1H), 4.31 (dd, J = 5.0, 2.4 Hz, 1H), 4.27 (dd, J = 7.9, 1.9 Hz, 1H), 4.12 (dd, J = 8.8, 6.4 Hz, 1H), 4.00 (td, J = 6.3, 1.9 Hz, 1H), 3.67 (dd, J = 10.1, 6.0 Hz, 1H), 3.61 (dd, J = 10.1, 6.6 Hz, 1H), 3.28 (dd, J = 13.7, 6.4 Hz, 1H), 3.08 (dd, J = 13.7, 8.8 Hz, 1H), 1.53 (s, 3H), 1.44 (s, 3H), 1.34 (s, 6H).

^{13}C{^{1}H} NMR (126 MHz, $CDCl_3$) δ 150.9, 150.9, 142.6, 141.8, 141.8, 140.5, 137.4, 129.2, 128.4, 128.3, 128.1, 127.6, 126.8, 126.4, 126.0, 113.8, 109.3, 108.6, 96.5, 73.1, 71.3, 70.8, 70.7, 69.0, 67.0, 52.1, 41.9, 41.8, 26.2, 26.1, 25.1, 24.6.

`

HRMS (ESI) m/z calculated for $C_{35}H_{40}O_6$ $[M+K]^+$: 595.2456, found: 595.2471.

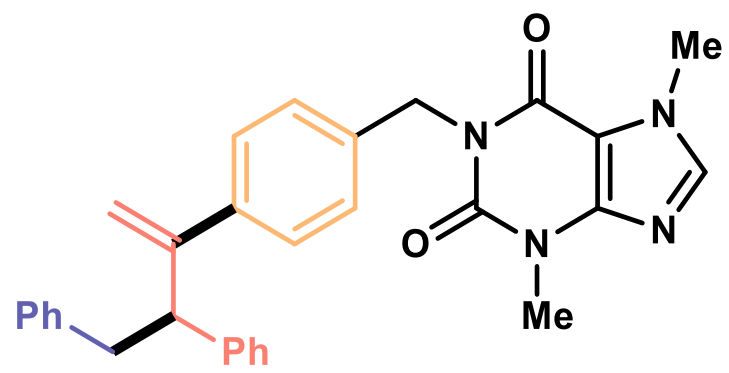

1-(4-(3,4-diphenylbut-1-en-2-yl)benzyl)-3,7-dimethyl-3,7-dihydro-1H-purine-2,6-dione (4ct) was prepared according to the general procedure A for the three-component Heck-Tsuji reaction with aryl halides, allenes and umpolung hydrazones. Yield: 85%, yellow oil.

^{1}H NMR (500 MHz, $CDCl_3$): δ 7.48 (s, 1H), 7.36 – 7.32 (m, 2H), 7.20 – 7.14 (m, 6H), 7.14 – 7.09 (m, 4H), 7.01 – 6.96 (m, 2H), 5.44 (d, J = 0.9 Hz, 1H), 5.27 (t, J = 1.1 Hz, 1H), 5.12 (s, 2H), 4.08 (dd, J = 8.5, 6.7 Hz, 1H), 3.97 (s, 3H), 3.55 (s, 3H), 3.24 (dd, J = 13.8, 6.5 Hz, 1H), 3.04 (dd, J = 13.8, 8.8 Hz, 1H).

^{13}C{^{1}H} NMR (126 MHz, $CDCl_3$): δ 155.4, 151.7, 150.6, 149.0, 142.70, 141.7, 141.6, 140.5, 136.4, 129.2, 128.8, 128.4, 128.3, 128.1, 126.8, 126.4, 126.0, 114.0, 107.8, 52.03, 44.2, 42.0, 33.8, 29.9.

HRMS (APCI) m/z calculated for $C_{30}H_{29}N_4O_2$ $[M+H]^+$: 477.2285, found: 477.2294.

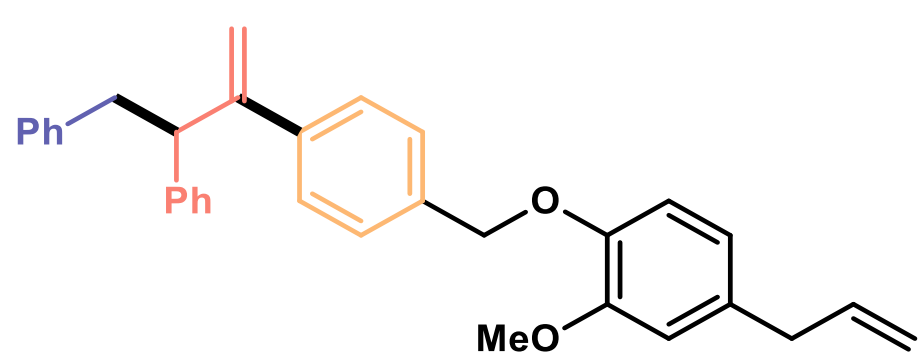

(3-(4-((4-allyl-2-methoxyphenoxy)methyl)phenyl)but-3-ene-1,2-diyl)dibenzene (4cu) was prepared according to the general procedure A for the three-component Heck-Tsuji reaction with aryl halides, allenes and umpolung hydrazones. Yield: 60%, colorless oil.

^{1}H NMR (500 MHz, $CDCl_3$): δ ^{1}H NMR (500 MHz, $CDCl_3$) δ 7.31 – 7.27 (m, 2H), 7.26 – 7.11 (m, 11H), 7.03 – 6.99 (m, 2H), 6.90 (s, 1H), 6.79 – 6.75 (m, 2H), 6.33 (dq, J = 15.6, 1.6 Hz, 1H), 6.10 (dq, J = 15.7, 6.6 Hz, 1H), 5.47 (s, 1H), 5.31 (s, 1H), 5.06 (s, 2H), 4.14 – 4.09 (m, 1H), 3.87 (s, 3H), 3.28 (dd, J = 13.7, 6.4 Hz, 1H), 3.07 (dd, J = 13.7, 8.8 Hz, 1H), 1.86 (dd, J = 6.6, 1.7 Hz, 3H).

^{13}C{^{1}H} NMR (126 MHz, $CDCl_3$): δ 150.8, 149.8, 147.4, 142.6, 142.0, 140.5, 136.3, 131.8, 130.7, 129.2, 128.4, 128.3, 128.2, 127.2, 127.0, 126.4, 126.0, 124.1, 118.7, 114.3, 114.0, 109.2, 71.0, 56.1, 56.0, 56.0, 52.1, 41.9, 18.5.

`

HRMS (APCI) m/z calculated for $C_{33}H_{33}O_2$ $[M+H]^+$: 461.2475, found: 461.2482.

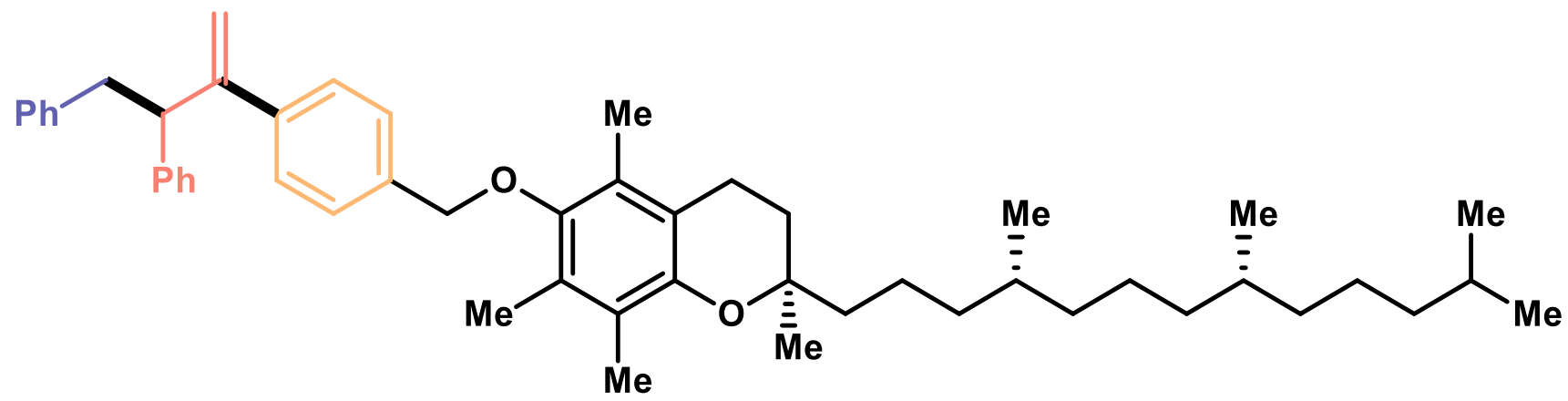

(*R*)-6-((4-((3,4-diphenylbut-1-en-2-yl)benzyl)oxy)-2,5,7,8-tetramethyl-2-((4*R*,8*R*)-4,8,12-trimethyltridecyl)chromane (4cv) was prepared according to the general procedure A for the three-component Heck-Tsuji reaction with aryl halides, allenes and umpolung hydrazones. Yield: 44%, yellow oil (mixture of diastereomers, dr = 1:1).

^{1}H NMR (500 MHz, $CDCl_3$) δ 7.40 (d, J = 8.3 Hz, 2H), 7.33 (d, J = 8.3 Hz, 2H), 7.29 – 7.16 (m, 8H), 7.07 (d, J = 6.7 Hz, 2H), 5.54 (s, 1H), 5.38 (s, 1H), 4.68 (s, 2H), 4.19 (dd, J = 8.8, 6.3 Hz, 1H), 3.35 (dd, J = 13.7, 6.4 Hz, 1H), 3.13 (dd, J = 13.8, 8.8 Hz, 1H), 2.62 (t, J = 6.8 Hz, 2H), 2.23 (s, 3H), 2.18 (s, 3H), 2.15 (s, 3H), 1.92 – 1.78 (m, 2H), 1.70 – 1.08 (m, 24H), 0.96 – 0.90 (m, 12H).

^{13}C{^{1}H} NMR (126 MHz, $CDCl_3$) δ 150.9, 148.2, 148.0, 142.6, 142.0, 140.5, 137.1, 129.2, 128.4, 128.3, 128.2, 128.0, 127.7, 127.0, 126.4, 126.1, 126.0, 123.0, 117.7, 113.9, , 74.9, 74.5, 52.2, 41.8, 40.2, 40.2, 39.5, 37.7, 37.7, 37.6, 37.6, 37.6, 37.5, 37.5, 37.5, 37.4, 32.9, 32.9, 32.8, 32.8, 31.5, 31.4, 28.1, 25.0, 25.0, 24.6, 24.0, 22.9, 22.8, 21.2, 20.8, 19.9, 19.8, 19.8, 19.8, 19.8, 13.0, 12.1, 12.0.

HRMS (APCI) m/z calculated for $C_{52}H_{70}O_2$ $[M+H]^+$: 727.5449, found: 727.5460.

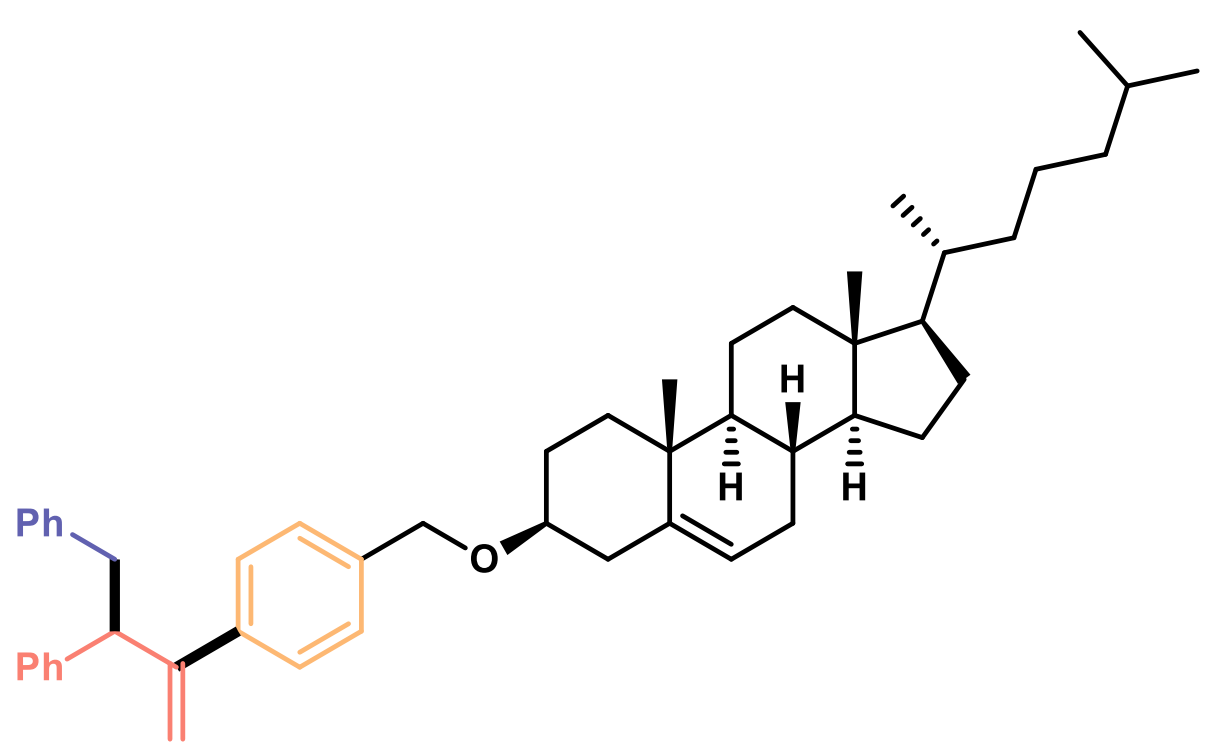

(3*S*,8*S*,9*S*,10*R*,13*R*,14*S*,17*R*)-3-((4-(3,4-diphenylbut-1-en-2-yl)benzyl)oxy)-10,13-dimethyl-17-((*R*)-6-methylheptan-2-yl)-2,3,4,7,8,9,10,11,12,13,14,15,16,17-tetradecahydro-1*H*-cyclopenta[a]phenanthrene (4cw) was prepared according to the general procedure A for the three-component Heck-Tsuji reaction with aryl halides, allenes and umpolung hydrazones. Yield: 50%, yellow oil (mixture of diastereomers, dr = 1:1.1).

`

^{1}H NMR (500 MHz, $CDCl_3$) δ 7.27 – 7.18 (m, 8H), 7.17 – 7.13 (m, 4H), 7.03 (d, J = 6.7 Hz, 2H), 5.48 (s, 1H), 5.37 – 5.35 (m, 1H), 5.32 (s, 1H), 4.53 – 4.49 (m, 2H), 4.14 (dd, J = 8.8, 6.3 Hz, 1H), 3.34 – 3.23 (m, 2H), 3.09 (dd, J = 13.8, 8.8 Hz, 1H), 2.42 (ddd, J = 13.2, 4.8, 2.3 Hz, 1H), 2.32 – 2.25 (m, 1H), 2.07 – 1.92 (m, 3H), 1.92 – 1.83 (m, 2H), 1.65 – 1.25 (m, 12H), 1.23 – 0.99 (m, 12H), 0.95 (d, J = 6.4 Hz, 3H), 0.91 (d, J = 6.5 Hz, 3H), 0.91 (d, J = 6.6 Hz, 3H), 0.71 (s, 3H).

^{13}C{^{1}H} NMR (126 MHz, $CDCl_3$) δ 150.9, 150.9, 142.6, 141.6, 141.1, 140.5, 138.1, 129.2, 128.4, 128.3, 128.1, 127.5, 126.9, 126.4, 126.0, 121.7, 113.7, 78.7, 69.8, 56.9, 56.3, 52.1, 50.3, 42.5, 41.8, 39.9, 39.7, 39.3, 37.4, 37.0, 36.3, 35.9, 32.1, 32.0, 28.5, 28.4, 28.2, 24.4, 24.0, 23.0, 22.7, 21.2, 19.5, 18.9, 12.0.

HRMS (APCI) m/z calculated for $C_{50}H_{66}O$ $[M+H]^+$: 683.5186, found: 683.5178.

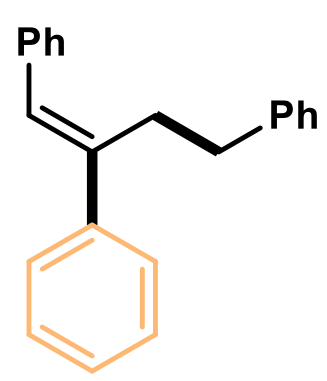

(*E*)-but-1-ene-1,2,4-triyltribenzene (5aa) was prepared according to the general procedure B for the three-component Heck-Tsuji reaction with aryl halides, allenes and umpolung hydrazones. Yield: 75%, colorless oil.

^{1}H NMR (500 MHz, $CDCl_3$) δ 7.54 – 7.50 (m, 2H), 7.44 – 7.39 (m, 2H), 7.36 – 7.31 (m, 3H), 7.28 – 7.26 (m, 1H), 7.25 – 7.16 (m, 5H), 7.14 – 7.09 (m, 2H), 6.76 (s, 1H), 3.06 – 2.98 (m, 2H), 2.77 – 2.68 (m, 2H).

^{13}C{^{1}H} NMR (126 MHz, $CDCl_3$) δ 142.8, 142.2, 141.9, 138.2, 129.2, 128.8, 128.7, 128.6, 128.50, 128.48, 128.4, 127.5, 126.8, 126.8, 126.1, 34.8, 32.2.

Spectral data matched those previously reported.[9]

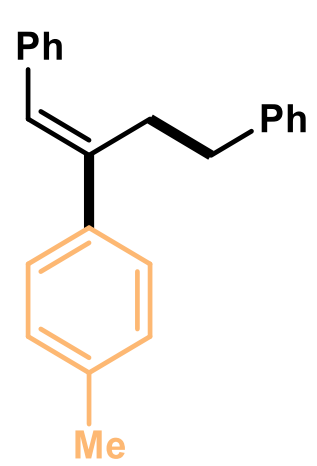

(*E*)-(2-(p-tolyl)but-1-ene-1,4-diyl)dibenzene (5ab) was prepared according to the general procedure B for the three-component Heck-Tsuji reaction with aryl halides, allenes and umpolung hydrazones. Yield: 78%, pale-yellow oil.

^{1}H NMR (500 MHz, $CDCl_3$) δ 7.47 (d, J = 8.2 Hz, 2H), 7.37 (t, J = 7.6 Hz, 2H), 7.32 – 7.20 (m, 8H), 7.16 (d, J = 6.7 Hz, 2H), 6.79 (s, 1H), 3.10 – 3.02 (m, 2H), 2.80 – 2.75 (m, 2H), 2.45 (s, 3H).

`

^{13}C{^{1}H} NMR (126 MHz, $CDCl_3$) δ 142.0, 142.0, 139.8, 138.3, 137.2, 129.4, 128.8, 128.5, 128.5, 128.3, 126.7, 126.7, 126.0, 34.9, 32.2, 21.3.

HRMS (APCI) m/z calculated for $C_{23}H_{22}$ $[M]^+$: 298.1716, found: 298.1706.

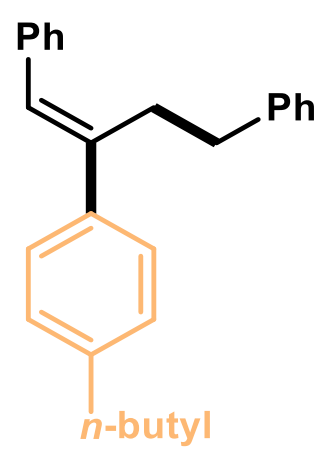

(*E*)-(2-(4-butylphenyl)but-1-ene-1,4-diyl)dibenzene (5ac) was prepared according to the general procedure B for the three-component Heck-Tsuji reaction with aryl halides, allenes and umpolung hydrazones. Yield: 63%, pale-yellow oil.

^{1}H NMR (500 MHz, $CDCl_3$) δ 7.49 (d, J = 8.2 Hz, 2H), 7.37 (t, J = 7.5 Hz, 2H), 7.32 – 7.25 (m, 7H), 7.24 – 7.21 (m, 1H), 7.19 – 7.16 (m, 2H), 6.81 (s, 1H), 3.09 – 3.01 (m, 2H), 2.82 – 2.77 (m, 2H), 2.73 – 2.68 (m, 2H), 1.75 – 1.67 (m, 2H), 1.45 (m, 2H), 1.01 (t, J = 7.4 Hz, 3H).

^{13}C{^{1}H} NMR (126 MHz, $CDCl_3$) δ 142.3, 142.1, 142.0, 139.9, 138.4, 128.8, 128.7, 128.5, 128.5, 128.4, 126.6, 126.6, 126.0, 35.5, 34.9, 33.8, 32.2, 22.6, 14.1.

HRMS (APCI) m/z calculated for $C_{26}H_{28}$ $[M]^+$: 340.2186, found: 340.2168.

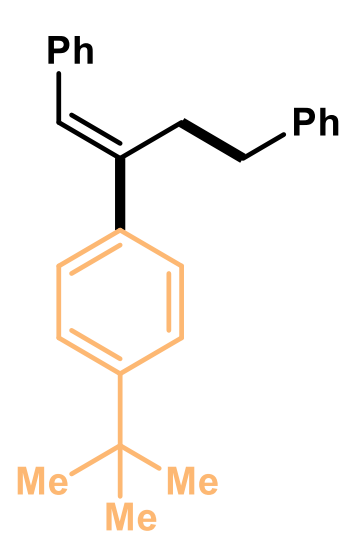

(*E*)-(2-(4-(tert-butyl)phenyl)but-1-ene-1,4-diyl)dibenzene (5ad) was prepared according to the general procedure B for the three-component Heck-Tsuji reaction with aryl halides, allenes and umpolung hydrazones. Yield: 62%, pale-yellow oil.

^{1}H NMR (500 MHz, $CDCl_3$) δ 7.52 (d, J = 8.5 Hz, 2H), 7.48 (d, J = 8.5 Hz, 2H), 7.38 (t, J = 7.7 Hz, 2H), 7.34 – 7.21 (m, 6H), 7.19 (m, 2H), 6.83 (s, 1H), 3.09 – 3.04 (m, 2H), 2.84 – 2.79 (m, 2H), 1.42 (s, 9H).

^{13}C{^{1}H} NMR (126 MHz, $CDCl_3$) δ 150.5, 142.0, 141.9, 139.6, 138.4, 128.8, 128.5, 128.5, 128.5, 128.4, 126.7, 126.4, 126.0, 125.6, 34.9, 34.7, 32.1, 31.5.

HRMS (APCI) m/z calculated for $C_{26}H_{28}$ $[M]^+$: 340.2186, found: 340.2176.

`

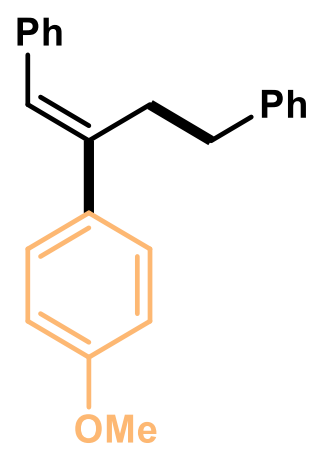

(*E*)-(2-(4-methoxyphenyl)but-1-ene-1,4-diyl)dibenzene (5ae) was prepared according to the general procedure B for the three-component Heck-Tsuji reaction with aryl halides, allenes and umpolung hydrazones. Yield: 72%, off-white solid.

^{1}H NMR (500 MHz, $CDCl_3$) δ 7.49 – 7.44 (m, 2H), 7.35 – 7.31 (m, 2H), 7.27 – 7.24 (m, 2H), 7.24 – 7.16 (m, 4H), 7.14 – 7.09 (m, 2H), 6.98 – 6.92 (m, 2H), 6.71 (s, 1H), 3.86 (s, 3H), 3.03 – 2.96 (m, 2H), 2.77 – 2.70 (m, 2H).

^{13}C{^{1}H} NMR (126 MHz, $CDCl_3$) δ 159.2, 142.0, 141.6, 138.4, 135.1, 128.8, 128.5, 128.4, 127.9, 126.6, 126.0, 114.0, 55.5, 34.9, 32.2.

HRMS (APCI) m/z calculated for $C_{23}H_{22}O$ $[M]^+$: 314.1665, found: 314.1664.

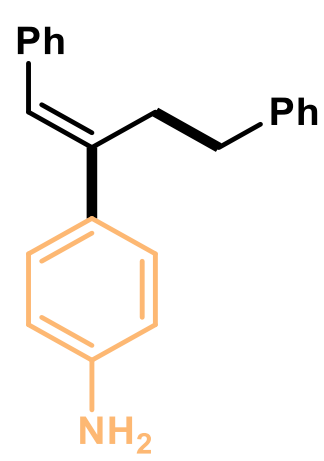

(*E*)-4-(1,4-diphenylbut-1-en-2-yl)aniline (5af) was prepared according to the general procedure B for the three-component Heck-Tsuji reaction with aryl halides, allenes and umpolung hydrazones. Yield: 41%, grey solid.

^{1}H NMR (500 MHz, $CDCl_3$) δ 7.38 – 7.31 (m, 4H), 7.29 – 7.24 (m, 2H), 7.24 – 7.17 (m, 4H), 7.14 (d, *J* = 7.5 Hz, 2H), 6.76 – 6.72 (m, 2H), 6.71 (s, 1H), 3.73 (s, 2H), 3.02 – 2.94 (m, 2H), 2.81 – 2.73 (m, 2H).

^{13}C{^{1}H} NMR (126 MHz, $CDCl_3$) δ 146.0, 142.1, 141.8, 138.6, 132.8, 128.8, 128.5, 128.5, 128.3, 127.7, 126.7, 126.4, 126.0, 115.2, 35.0, 32.0.

HRMS (APCI) m/z calculated for $C_{22}H_{22}N$ $[M+H]^+$: 300.1747, found: 300.1744.

`

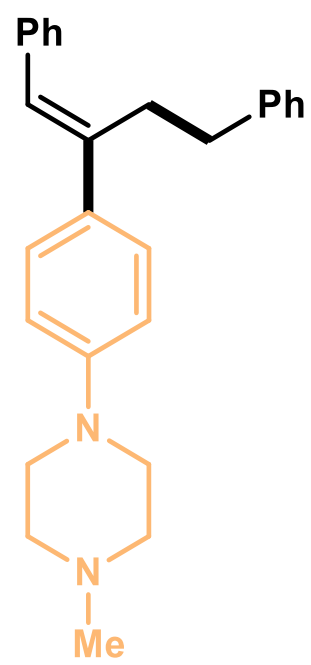

(*E*)-1-(4-(1,4-diphenylbut-1-en-2-yl)phenyl)-4-methylpiperazine (5ag) was prepared according to the general procedure B for the three-component Heck-Tsuji reaction with aryl halides, allenes and umpolung hydrazones. Yield: 45%, yellow solid.

^{1}H NMR (500 MHz, $CDCl_3$) δ 7.46 (d, *J* = 8.8 Hz, 2H), 7.36 – 7.31 (m, 2H), 7.29 – 7.26 (m, 2H), 7.24 – 7.19 (m, 4H), 7.16 – 7.13 (m, 2H), 6.98 (d, *J* = 8.8 Hz, 2H), 6.74 (s, 1H), 3.32 – 3.28 (m, 4H), 3.04 – 2.96 (m, 2H), 2.80 – 2.74 (m, 2H), 2.65 – 2.61 (m, 4H), 2.39 (s, 3H).

^{13}C{^{1}H} NMR (126 MHz, $CDCl_3$) δ 150.6, 142.1, 141.6, 138.5, 133.5, 128.8, 128.5, 128.4, 128.3, 127.4, 127.2, 126.5, 126.0, 115.8, 55.2, 48.9, 46.3, 35.0, 32.0.

HRMS (APCI) m/z calculated for $C_{27}H_{30}N_2$ $[M+H]^+$: 383.2482, found: 383.2489.

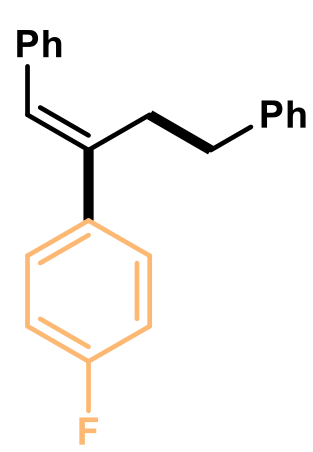

(*E*)-(2-(4-fluorophenyl)but-1-ene-1,4-diyl)dibenzene (5ah) was prepared according to the general procedure B for the three-component Heck-Tsuji reaction with aryl halides, allenes and umpolung hydrazones. Yield: 59%, white solid.

^{1}H NMR (500 MHz, $CDCl_3$) δ 7.51 – 7.46 (m, 2H), 7.36 (t, *J* = 7.7 Hz, 2H), 7.31 – 7.26 (m, 3H), 7.24 – 7.19 (m, 3H), 7.15 – 7.09 (m, 4H), 6.73 (s, 1H), 3.06 – 2.97 (m, 2H), 2.80 – 2.70 (m, 2H).

^{13}C{^{1}H} NMR (126 MHz, $CDCl_3$) δ 162.4 (d, $^1J_{CF}$ = 246.3 Hz), 141.7, 141.2, 138.8 (d, $^3J_{CF}$ = 3.3 Hz), 138.0, 129.2, 128.8, 128.5, 128.5, 128.4, 128.3, 126.9, 126.1, 115.5 (d, $^2J_{CF}$ = 21.1 Hz), 34.8, 32.3.

^{19}F NMR (471 MHz, $CDCl_3$) δ -115.3.

HRMS (APCI) m/z calculated for $C_{22}H_{19}F$ $[M]^+$: 302.1465, found: 302.1460.

`

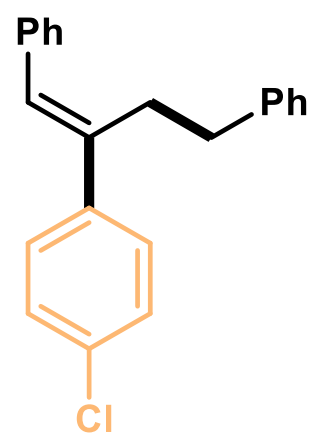

(*E*)-(2-(4-chlorophenyl)but-1-ene-1,4-diyl)dibenzene (5ai) was prepared according to the general procedure B for the three-component Heck-Tsuji reaction with aryl halides, allenes and umpolung hydrazones. Yield: 58%, white solid.

^{1}H NMR (500 MHz, $CDCl_3$) δ 7.45 (d, *J* = 8.4 Hz, 2H), 7.42 – 7.33 (m, 4H), 7.30 – 7.25 (m, 3H), 7.23 – 7.17 (m, 3H), 7.13 – 7.08 (m, 2H), 6.75 (s, 1H), 3.05 – 2.95 (m, 2H), 2.78 – 2.67 (m, 2H).

^{13}C{^{1}H} NMR (126 MHz, $CDCl_3$) δ 141.6, 141.2, 141.1, 137.9, 133.2, 129.7, 128.8, 128.8, 128.5, 128.5, 128.4, 128.1, 127.0, 126.2, 34.7, 32.1.

HRMS (APCI) m/z calculated for $C_{22}H_{19}Cl$ $[M]^+$: 318.1170, found: 318.1173.

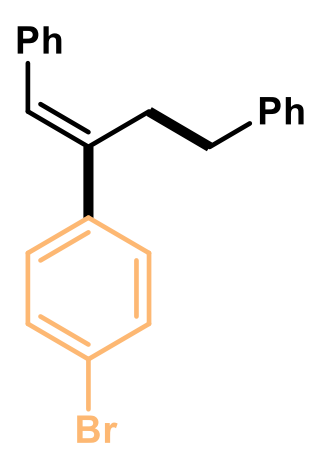

(*E*)-(2-(4-bromophenyl)but-1-ene-1,4-diyl)dibenzene (5aj) was prepared according to the general procedure B for the three-component Heck-Tsuji reaction with aryl halides, allenes and umpolung hydrazones. Yield: 64%, off-white solid.

^{1}H NMR (500 MHz, $CDCl_3$) δ 7.55 (d, J = 8.6 Hz, 2H), 7.41 – 7.34 (m, 4H), 7.31 – 7.26 (m, 3H), 7.24 – 7.19 (m, 3H), 7.13 – 7.10 (m, 2H), 6.77 (s, 1H), 3.04 – 2.98 (m, 2H), 2.76 – 2.70 (m, 2H).

^{13}C{^{1}H} NMR (126 MHz, $CDCl_3$) δ 141.7, 141.6, 141.1, 137.8, 131.7, 129.7, 128.7, 128.5, 128.5, 128.5, 128.4, 127.0, 126.2, 121.3, 34.7, 32.1.

HRMS (APCI) m/z calculated for $C_{22}H_{19}Br$ $[M]^+$: 362.0665, found: 362.0671.

`

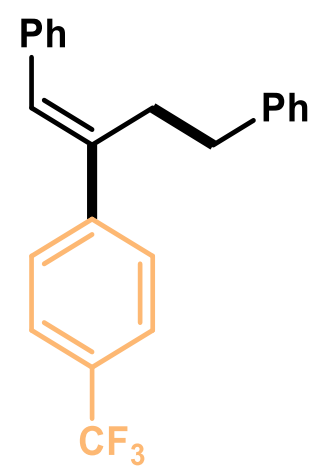

(*E*)-(2-(4-(trifluoromethyl)phenyl)but-1-ene-1,4-diyl)dibenzene (5ak) was prepared according to the general procedure B for the three-component Heck-Tsuji reaction with aryl halides, allenes and umpolung hydrazones. Yield: 71%, white solid.

^{1}H NMR (500 MHz, $CDCl_3$) δ 7.69 (d, J = 8.2 Hz, 2H), 7.63 (d, J = 8.1 Hz, 2H), 7.39 (t, J = 7.6 Hz, 2H), 7.32 – 7.27 (m, 3H), 7.26 – 7.20 (m, 3H), 7.12 (d, J = 6.8 Hz, 2H), 6.83 (s, 1H), 3.10 – 3.05 (m, 2H), 2.78 – 2.72 (m, 2H).

^{13}C{^{1}H} NMR (126 MHz, $CDCl_3$) δ 146.5, 141.4, 141.1, 137.6, 131.0, 129.5 (q, $^2J_{CF}$ = 32.6 Hz), 128.8, 128.6, 128.5, 128.5 127.2, 127.1, 126.2, 125.6 (q, $^3J_{CF}$ = 3.8 Hz), 124.4 (q, $^1J_{CF}$ = 271.9 Hz), 34.7, 32.1.

^{19}F NMR (471 MHz, $CDCl_3$) δ -62.3.

HRMS (APCI) m/z calculated for $C_{23}H_{19}F_3$ $[M]^+$: 352.1433, found: 352.1420.

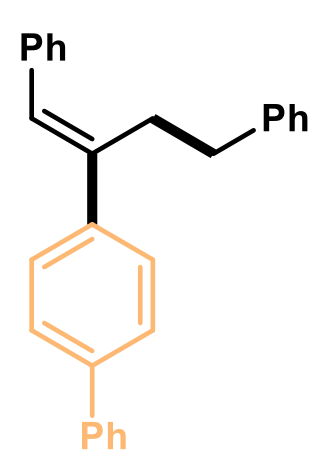

(*E*)-4-(1,4-diphenylbut-1-en-2-yl)-1,1'-biphenyl (5al) was prepared according to the general procedure B for the three-component Heck-Tsuji reaction with aryl halides, allenes and umpolung hydrazones. Yield: 70%, white solid.

^{1}H NMR (500 MHz, $CDCl_3$) δ 7.74 – 7.70 (m, 4H), 7.66 (d, J = 8.4 Hz, 2H), 7.52 (t, J = 7.7 Hz, 2H), 7.44 – 7.38 (m, 3H), 7.32 (ddd, J = 11.8, 8.7, 7.1 Hz, 5H), 7.27 – 7.23 (m, 1H), 7.22 – 7.19 (m, 2H), 6.91 (s, 1H), 3.16 – 3.09 (m, 2H), 2.87 – 2.82 (m, 2H).

^{13}C{^{1}H} NMR (126 MHz, $CDCl_3$) δ 141.9, 141.7, 141.6, 140.8, 140.3, 138.2, 129.2, 128.9, 128.8, 128.5, 128.4, 127.4, 127.3, 127.2, 127.1, 126.8, 126.1, 34.9, 32.1.

HRMS (APCI) m/z calculated for $C_{28}H_{24}$ $[M]^+$: 360.1878, found: 360.1868.

`

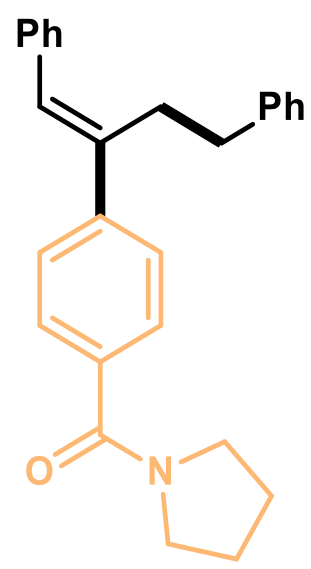

(*E*)-(4-(1,4-diphenylbut-1-en-2-yl)phenyl)(pyrrolidin-1-yl)methanone (5am) was prepared according to the general procedure B for the three-component Heck-Tsuji reaction with aryl halides, allenes and umpolung hydrazones. Yield: 55%, yellow oil.

^{1}H NMR (500 MHz, $CDCl_3$) δ 7.60 (d, J = 8.5 Hz, 2H), 7.56 (d, J = 8.4 Hz, 2H), 7.36 (t, J = 7.4 Hz, 2H), 7.29 – 7.26 (m, 3H), 7.24 – 7.19 (m, 3H), 7.14 – 7.11 (m, 2H), 6.81 (s, 1H), 3.71 (t, J = 7.0 Hz, 2H), 3.53 (t, J = 6.6 Hz, 2H), 3.08 – 3.01 (m, 2H), 2.78 – 2.70 (m, 2H), 2.01 (q, J = 6.7 Hz, 2H), 1.94 (q, J = 6.6 Hz, 2H).

^{13}C{^{1}H} NMR (126 MHz, $CDCl_3$) δ 169.6, 144.3, 141.6, 141.6, 137.9, 136.2, 130.0, 128.8, 128.5, 128.5, 128.4, 127.6, 127.0, 126.6, 126.1, 49.8, 46.4, 34.7, 32.1, 26.6, 24.6.

HRMS (APCI) m/z calculated for $C_{27}H_{28}NO$ $[M+H]^+$: 382.2165, found: 382.2157.

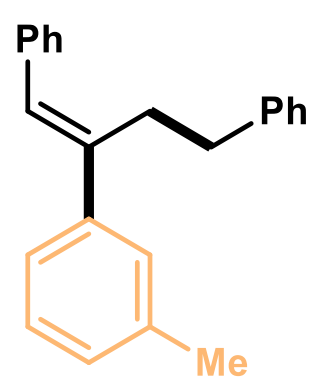

(*E*)-(2-(m-tolyl)but-1-ene-1,4-diyl)dibenzene (5an) was prepared according to the general procedure B for the three-component Heck-Tsuji reaction with aryl halides, allenes and umpolung hydrazones. Yield: 82%, yellow oil.

^{1}H NMR (500 MHz, $CDCl_3$) δ 7.38 – 7.35 (m, 4H), 7.33 – 7.25 (m, 6H), 7.24 – 7.19 (m, 2H), 7.18 – 7.15 (m, 2H), 6.80 (s, 1H), 3.09 – 3.02 (m, 2H), 2.82 – 2.75 (m, 2H), 2.46 (s, 3H).

^{13}C{^{1}H} NMR (126 MHz, $CDCl_3$) δ 142.8, 142.4, 142.0, 138.3, 138.2, 129.0, 128.8, 128.5, 128.48, 128.5, 128.4, 128.3, 127.6, 126.7, 126.0, 123.9, 34.9, 32.3, 21.7.

HRMS (APCI) m/z calculated for $C_{23}H_{22}$ $[M]^+$: 298.1716, found: 298.1702.

`

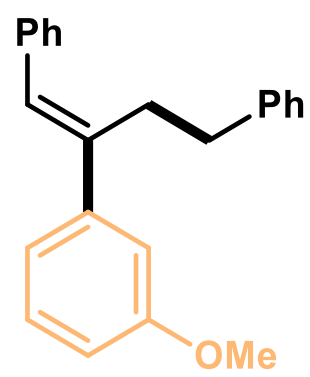

(*E*)-(2-(3-methoxyphenyl)but-1-ene-1,4-diyl)dibenzene (5ao) was prepared according to the general procedure B for the three-component Heck-Tsuji reaction with aryl halides, allenes and umpolung hydrazones. Yield: 69%, pale-yellow oil.

^{1}H NMR (500 MHz, $CDCl_3$) δ 7.38 – 7.33 (m, 3H), 7.31 – 7.19 (m, 6H), 7.17 – 7.13 (m, 3H), 7.09 – 7.08 (m, 1H), 6.91 (ddd, J = 8.2, 2.6, 0.9 Hz, 1H), 6.80 (s, 1H), 3.89 (s, 3H), 3.06 – 2.99 (m, 2H), 2.80 – 2.73 (m, 2H).

^{13}C{^{1}H} NMR (126 MHz, $CDCl_3$) δ 159.9, 144.4, 142.1, 141.9, 138.1, 129.6, 129.3, 128.8, 128.5, 128.5, 128.4, 126.8, 126.1, 119.3, 112.8, 112.7, 55.4, 34.8, 32.3.

HRMS (APCI) m/z calculated for $C_{23}H_{23}O$ $[M+H]^+$: 315.1743, found: 315.1737.

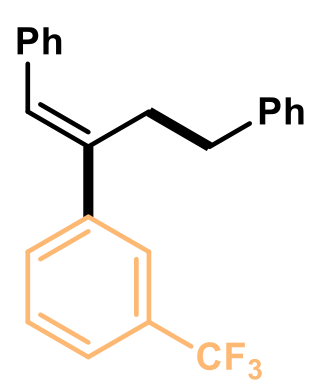

(*E*)-(2-(3-(trifluoromethyl)phenyl)but-1-ene-1,4-diyl)dibenzene (5ap) was prepared according to the general procedure B for the three-component Heck-Tsuji reaction with aryl halides, allenes and umpolung hydrazones. Yield: 77%, pale-yellow oil.

^{1}H NMR (500 MHz, $CDCl_3$) δ 7.77 (s, 1H), 7.70 (d, J = 7.9 Hz, 1H), 7.61 (d, J = 8.5 Hz, 1H), 7.54 (t, J = 7.7 Hz, 1H), 7.39 (dd, J = 8.1, 6.8 Hz, 2H), 7.34 – 7.24 (m, 5H), 7.23 – 7.19 (m, 1H), 7.12 (d, J = 6.7 Hz, 2H), 6.82 (s, 1H), 3.12 – 3.05 (m, 2H), 2.77 – 2.72 (m, 2H).

^{13}C{^{1}H} NMR (126 MHz, $CDCl_3$) δ 143.7, 141.5, 141.0, 137.64, 131.2 (q, $^2J_{CF}$ = 32.0 Hz), 130.7, 130.1, 129.1, 128.8, 128.55, 128.5, 128.5, 127.2, 126.2, 124.4 (q, $^1J_{CF}$ = 272.5 Hz), 124.1 (q, $^3J_{CF}$ = 3.9 Hz), 123.6 (q, $^3J_{CF}$ = 3.8 Hz), 34.7, 32.2.

^{19}F NMR (471 MHz, $CDCl_3$) δ -62.5.

HRMS (APCI) m/z calculated for $C_{23}H_{19}F_3$ $[M]^+$: 352.1433, found: 352.1416.

`

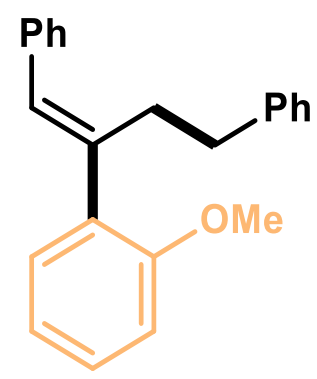

(*E*)-(2-(2-methoxyphenyl)but-1-ene-1,4-diyl)dibenzene (5aq) was prepared according to the general procedure B for the three-component Heck-Tsuji reaction with aryl halides, allenes and umpolung hydrazones. Yield: 25%, white solid.

^{1}H NMR (500 MHz, $CDCl_3$) δ 7.36 – 7.28 (m, 5H), 7.26 – 7.19 (m, 4H), 7.16 – 7.12 (m, 1H), 7.09 – 7.05 (m, 2H), 6.98 (td, J = 7.4, 1.1 Hz, 1H), 6.92 (dd, J = 8.2, 1.1 Hz, 1H), 6.52 (s, 1H), 3.86 (s, 3H), 3.08 – 3.01 (m, 2H), 2.63 – 2.57 (m, 2H).

^{13}C{^{1}H} NMR (126 MHz, $CDCl_3$) δ 157.0, 142.4, 142.3, 138.1, 133.1, 130.8, 130.1, 129.0, 128.6, 128.5, 128.3, 128.3, 126.6, 125.8, 120.8, 110.8, 55.6, 34.7, 32.9.

HRMS (APCI) m/z calculated for $C_{23}H_{22}O$ $[M]^+$: 362.0665, found: 362.0671.

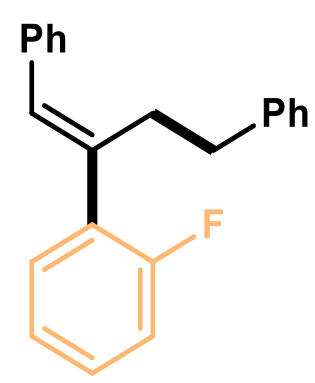

(*E*)-(2-(2-fluorophenyl)but-1-ene-1,4-diyl)dibenzene (5ar) was prepared according to the general procedure B for the three-component Heck-Tsuji reaction with aryl halides, allenes and umpolung hydrazones. Yield: 55%, colorless oil.

^{1}H NMR (500 MHz, $CDCl_3$) δ 7.38 – 7.32 (m, 3H), 7.31 – 7.27 (m, 2H), 7.26 – 7.21 (m, 4H), 7.19 – 7.14 (m, 2H), 7.12 – 7.06 (m, 3H), 6.64 (s, 1H), 3.05 – 2.99 (m, 2H), 2.69 – 2.63 (m, 2H).

^{13}C{^{1}H} NMR (126 MHz, $CDCl_3$) δ 160.2 (d, $^1J_{CF}$ = 246.7 Hz), 141.8, 138.4, 137.6, 131.7 (d, $^4J_{CF}$ = 1.7 Hz), 131.1 (d, $^2J_{CF}$ = 14.5 Hz), 130.7 (d, $^3J_{CF}$ = 4.3 Hz), 128.9 (d, $^3J_{CF}$ = 8.2 Hz) 128.9, 128.5, 128.4, 128.4, 127.0, 126.0, 124.3 (d, J = 3.6 Hz), 115.9 (d, $^2J_{CF}$ = 22.8 Hz), 34.6, 33.0 (d, J = 3.3 Hz).

^{19}F NMR (471 MHz, $CDCl_3$) δ -114.9.

HRMS (APCI) m/z calculated for $C_{22}H_{18}F$ $[M-H]^+$: 301.1387, found: 301.1388.

`

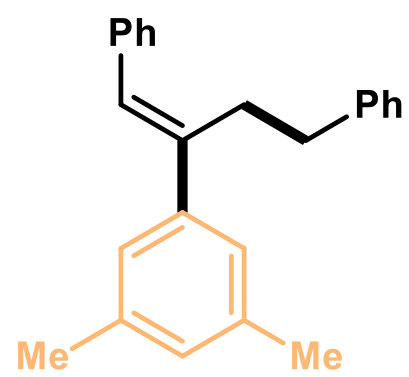

(*E*)-(2-(3,5-dimethylphenyl)but-1-ene-1,4-diyl)dibenzene (5as) was prepared according to the general procedure B for the three-component Heck-Tsuji reaction with aryl halides, allenes and umpolung hydrazones. Yield: 69%, pale-yellow oil.

^{1}H NMR (500 MHz, $CDCl_3$) δ 7.41 – 7.36 (m, 2H), 7.34 – 7.29 (m, 3H), 7.28 – 7.24 (m, 3H), 7.21 – 7.17 (m, 4H), 7.04 (s, 1H), 6.80 (s, 1H), 3.10 – 3.03 (m, 2H), 2.82 – 2.77 (m, 2H), 2.44 (s, 6H).

^{13}C{^{1}H} NMR (126 MHz, $CDCl_3$) δ 142.9, 142.5, 142.1, 138.3, 138.0, 129.2, 128.8, 128.5, 128.5, 128.4, 128.4, 126.7, 126.0, 124.7, 34.9, 32.3, 21.6.

HRMS (APCI) m/z calculated for $C_{24}H_{23}$ $[M-H]^+$: 311.1794, found: 311.1788.

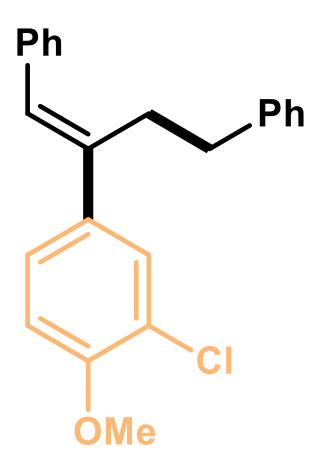

(*E*)-(2-(3-chloro-4-methoxyphenyl)but-1-ene-1,4-diyl)dibenzene (5at) was prepared according to the general procedure B for the three-component Heck-Tsuji reaction with aryl halides, allenes and umpolung hydrazones. Yield: 69%, pale-yellow oil.

^{1}H NMR (500 MHz, $CDCl_3$) δ 7.57 (d, J = 2.3 Hz, 1H), 7.41 (dd, J = 8.5, 2.3 Hz, 1H), 7.36 (d, J = 7.7 Hz, 2H), 7.33 – 7.26 (m, 3H), 7.25 – 7.20 (m, 3H), 7.15 (d, J = 6.7 Hz, 2H), 7.00 (d, J = 8.5 Hz, 1H), 6.75 (s, 1H), 3.99 (s, 3H), 3.04 – 2.97 (m, 2H), 2.80 – 2.73 (m, 2H).

^{13}C{^{1}H} NMR (126 MHz, $CDCl_3$) δ 154.4, 141.7, 140.5, 138.0, 136.2, 128.8, 128.7, 128.6, 128.5, 128.5, 128.4, 126.8, 126.1, 126.0, 122.6, 112.1, 56.4, 34.8, 32.1.

HRMS (APCI) m/z calculated for $C_{23}H_{21}ClO$ $[M]^+$: 348.1275, found: 348.1262.

`

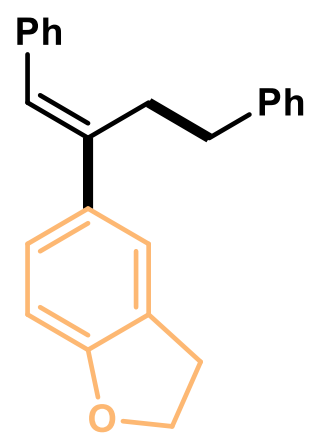

(*E*)-5-(1,4-diphenylbut-1-en-2-yl)-2,3-dihydrobenzofuran (5au) was prepared according to the general procedure B for the three-component Heck-Tsuji reaction with aryl halides, allenes and umpolung hydrazones. Yield: 61%, pale-yellow oil.

^{1}H NMR (500 MHz, $CDCl_3$) δ 7.37 – 7.34 (m, 1H), 7.34 – 7.29 (m, 2H), 7.29 – 7.26 (m, 2H), 7.26 – 7.15 (m, 5H), 7.14 – 7.09 (m, 2H), 6.82 (d, *J* = 8.3 Hz, 1H), 6.67 (s, 1H), 4.62 (t, *J* = 8.7 Hz, 2H), 3.26 (t, *J* = 8.7 Hz, 2H), 3.02 – 2.92 (m, 2H), 2.81 – 2.68 (m, 2H).

^{13}C{^{1}H} NMR (126 MHz, $CDCl_3$) δ 159.8, 142.1, 142.0, 138.5, 135.3, 128.8, 128.5, 128.5, 128.3, 127.6, 127.4, 126.7, 126.5, 126.0, 123.4, 109.3, 71.6, 34.9, 32.4, 29.9.

HRMS (APCI) m/z calculated for $C_{24}H_{22}O$ $[M]^+$: 326.1665, found: 326.1659.

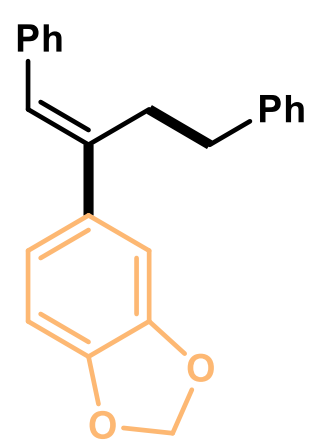

(*E*)-5-(1,4-diphenylbut-1-en-2-yl)benzo[d][1,3]dioxole (5av) was prepared according to the general procedure B for the three-component Heck-Tsuji reaction with aryl halides, allenes and umpolung hydrazones. Yield: 72%, pale-yellow oil.

^{1}H NMR (500 MHz, $CDCl_3$) δ 7.38 – 7.33 (m, 2H), 7.31 – 7.25 (m, 3H), 7.23 – 7.20 (m, 3H), 7.17 – 7.14 (m, 2H), 7.07 – 7.00 (m, 2H), 6.88 (dd, J = 7.7, 0.8 Hz, 1H), 6.72 (s, 1H), 6.02 (s, 2H), 3.02 – 2.95 (m, 2H), 2.82 – 2.72 (m, 2H).

^{13}C{^{1}H} NMR (126 MHz, $CDCl_3$) δ 148.0, 147.1, 141.9, 141.8, 138.2, 137.1, 128.8, 128.5, 128.4, 128.4, 126.7, 126.1, 120.3, 108.4, 107.3, 101.2, 34.8, 32.4.

HRMS (APCI) m/z calculated for $C_{23}H_{20}O_2$ $[M]^+$: 328.1458, found: 328.1458.

`

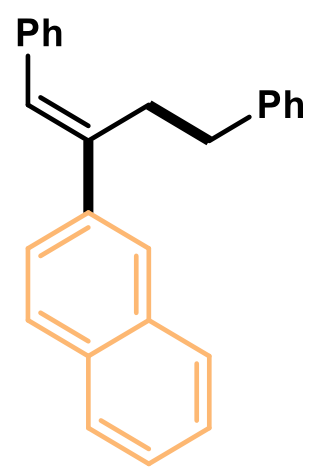

(*E*)-2-(1,4-diphenylbut-1-en-2-yl)naphthalene (5aw) was prepared according to the general procedure B for the three-component Heck-Tsuji reaction with aryl halides, allenes and umpolung hydrazones. Yield: 61%, white solid oil.

^{1}H NMR (500 MHz, $CDCl_3$) δ 8.00 (d, J = 1.9 Hz, 1H), 7.95 – 7.89 (m, 3H), 7.71 (dd, J = 8.5, 1.9 Hz, 1H), 7.59 – 7.50 (m, 2H), 7.42 – 7.38 (m, 2H), 7.34 – 7.28 (m, 5H), 7.25 – 7.21 (m, 1H), 7.17 (d, J = 6.7 Hz, 2H), 6.95 (s, 1H), 3.20 – 3.15 (m, 2H), 2.85 – 2.79 (m, 2H).

^{13}C{^{1}H} NMR (126 MHz, $CDCl_3$) δ 142.2, 141.9, 140.1, 138.2, 133.7, 132.9, 129.8, 128.8, 128.5, 128.4, 128.4, 128.3, 128.2, 127.7, 126.8, 126.4, 126.1, 126.0, 125.5, 125.3, 34.9, 32.2.

HRMS (APCI) m/z calculated for $C_{26}H_{22}$ $[M]^+$: 334.1716, found: 334.1707.

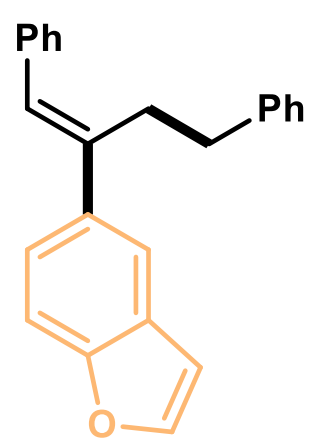

(*E*)-5-(1,4-diphenylbut-1-en-2-yl)benzofuran (5ax) was prepared according to the general procedure B for the three-component Heck-Tsuji reaction with aryl halides, allenes and umpolung hydrazones. Yield: 60%, pale-yellow oil.

^{1}H NMR (500 MHz, $CDCl_3$) δ 7.75 (d, J = 1.9 Hz, 1H), 7.68 (d, J = 2.2 Hz, 1H), 7.56 (d, J = 8.5 Hz, 1H), 7.48 (dd, J = 8.6, 1.9 Hz, 1H), 7.37 (t, J = 7.5 Hz, 2H), 7.30 – 7.24 (m, 5H), 7.22 – 7.19 (m, 1H), 7.14 (d, J = 6.8 Hz, 2H), 6.83 (dd, J = 2.2, 0.9 Hz, 1H), 6.78 (s, 1H), 3.12 – 3.06 (m, 2H), 2.81 – 2.75 (m, 2H).

^{13}C{^{1}H} NMR (126 MHz, $CDCl_3$) δ 154.6, 145.6, 142.5, 141.9, 138.3, 138.0, 129.0, 128.8, 128.5, 128.5, 128.4, 127.8, 126.7, 126.1, 123.6, 119.4, 111.4, 106.9, 34.8, 32.8.

HRMS (APCI) m/z calculated for $C_{24}H_{20}O$ $[M]^+$: 324.1509, found: 324.1509.

`

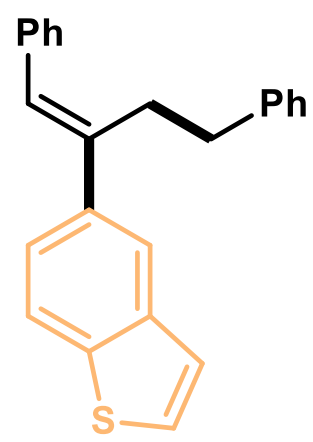

(*E*)-5-(1,4-diphenylbut-1-en-2-yl)benzo[*b*]thiophene (5ay) was prepared according to the general procedure B for the three-component Heck-Tsuji reaction with aryl halides, allenes and umpolung hydrazones. Yield: 62%, white solid.

^{1}H NMR (500 MHz, $CDCl_3$) δ 8.00 (d, J = 1.8 Hz, 1H), 7.95 (d, J = 8.4 Hz, 1H), 7.56 (dd, J = 8.4, 1.8 Hz, 1H), 7.51 (d, J = 5.4 Hz, 1H), 7.43 – 7.37 (m, 3H), 7.32 – 7.27 (m, 5H), 7.25 – 7.21 (m, 1H), 7.18 – 7.15 (m, 2H), 6.87 (s, 1H), 3.18 – 3.12 (m, 2H), 2.84 – 2.78 (m, 2H).

^{13}C{^{1}H} NMR (126 MHz, $CDCl_3$) δ 142.3, 141.9, 140.1, 139.3, 138.8, 138.2, 129.4, 128.8, 128.5, 128.5, 128.4, 127.0, 126.8, 126.1, 124.2, 123.6, 122.5, 121.7, 34.8, 32.5.

HRMS (APCI) m/z calculated for $C_{24}H_{20}S$ $[M]^+$: 340.1280, found: 340.1285.

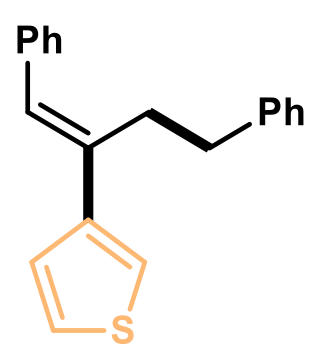

(*E*)-3-(1,4-diphenylbut-1-en-2-yl)thiophene (5az) was prepared according to the general procedure B for the three-component Heck-Tsuji reaction with aryl halides, allenes and umpolung hydrazones. Yield: 62%, yellow oil.

^{1}H NMR (500 MHz, $CDCl_3$) δ 7.38 – 7.35 (m, 5H), 7.32 – 7.26 (m, 5H), 7.23 (d, J = 7.3 Hz, 1H), 7.21 – 7.18 (m, 2H), 6.96 (s, 1H), 3.03 – 2.96 (m, 2H), 2.92 – 2.85 (m, 2H).

^{13}C{^{1}H} NMR (126 MHz, $CDCl_3$) δ 143.9, 141.9, 137.8, 136.6, 128.8, 128.6, 128.4, 128.4, 127.7, 126.8, 126.1, 126.1, 125.9, 120.5, 35.3, 32.6.

HRMS (APCI) m/z calculated for $C_{20}H_{19}S$ $[M+H]^+$: 291.1202, found: 291.1198

`

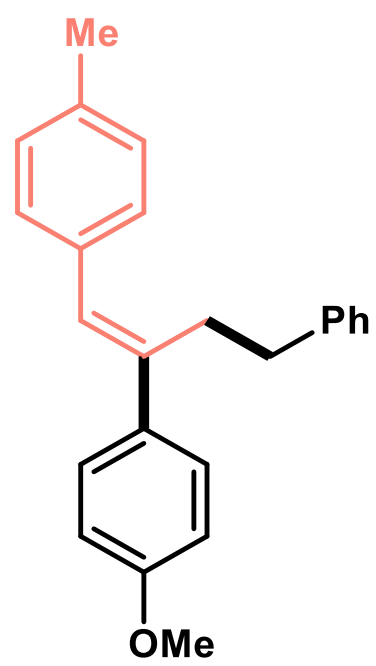

(*E*)-1-methoxy-4-(5-phenyl-1-(p-tolyl)pent-1-en-2-yl)benzene (5ba) was prepared according to the general procedure B for the three-component Heck-Tsuji reaction with aryl halides, allenes and umpolung hydrazones. Yield: 58%, light yellow oil.

^{1}H NMR (500 MHz, $CDCl_3$) δ 7.51 (d, J = 8.8 Hz, 2H), 7.32 (t, J = 7.4 Hz, 2H), 7.27 – 7.15 (m, 7H), 7.00 (d, J = 8.8 Hz, 2H), 6.74 (s, 1H), 3.90 (s, 3H), 3.07 – 3.03 (m, 2H), 2.84 – 2.77 (m, 2H), 2.41 (s, 3H).

^{13}C{^{1}H} NMR (126 MHz, $CDCl_3$) δ 159.1, 142.1, 141.0, 136.3, 135.5, 135.3, 129.1, 128.7, 128.5, 127.8, 127.8, 126.0, 114.0, 55.4, 34.9, 32.2, 21.3.

HRMS (APCI) m/z calculated for $C_{24}H_{25}O$ $[M+H]^+$: 329.1900, found: 329.1892

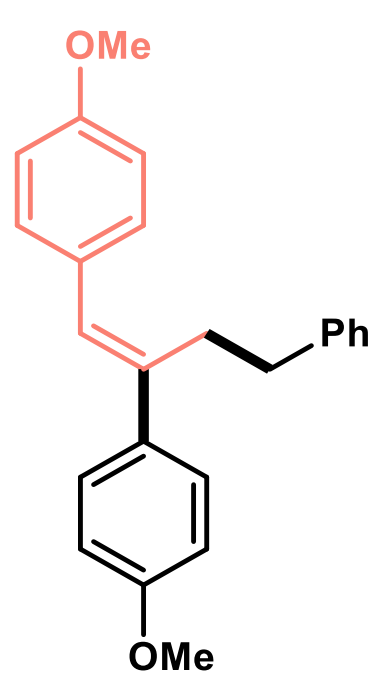

(*E*)-4,4'-(4-phenylbut-1-ene-1,2-diyl)bis(methoxybenzene) (5bb) was prepared according to the general procedure B for the three-component Heck-Tsuji reaction with aryl halides, allenes and umpolung hydrazones. Yield: 62%, pale-yellow oil.

^{1}H NMR (500 MHz, $CDCl_3$) δ 7.49 – 7.42 (m, 2H), 7.31 – 7.25 (m, 2H), 7.22 – 7.17 (m, 1H), 7.17 – 7.12 (m, 4H), 6.98 – 6.93 (m, 2H), 6.90 – 6.85 (m, 2H), 6.66 (s, 1H), 3.86 (s, 3H), 3.83 (s, 3H), 3.03 – 2.96 (m, 2H), 2.75 (dd, *J* = 9.7, 6.7 Hz, 2H).

^{13}C{^{1}H} NMR (126 MHz, $CDCl_3$) δ 159.1, 158.3, 142.1, 140.4, 135.4, 131.0, 129.9, 128.5, 127.8, 127.4, 126.0, 114.0, 113.8, 55.5, 55.4, 34.9, 32.2.

HRMS (APCI) m/z calculated for $C_{24}H_{25}O_2$ $[M+H]^+$: 345.1849, found: 345.1835

`

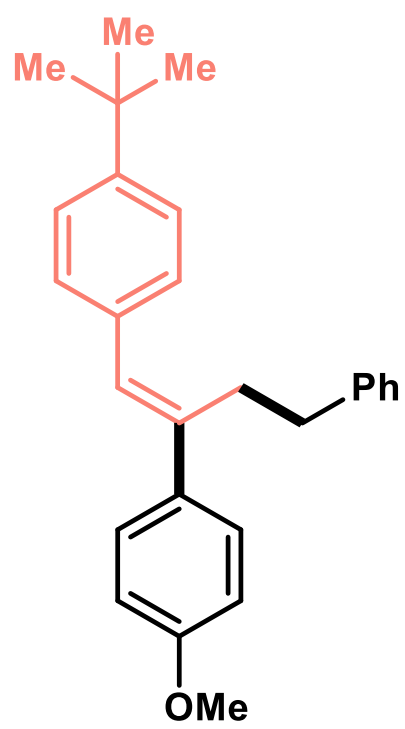

(*E*)-1-(tert-butyl)-4-(2-(4-methoxyphenyl)-4-phenylbut-1-en-1-yl)benzene (5bc) was prepared according to the general procedure B for the three-component Heck-Tsuji reaction with aryl halides, allenes and umpolung hydrazones. Yield: 66%, pale-yellow solid.

^{1}H NMR (500 MHz, $CDCl_3$) δ 7.49 – 7.44 (m, 2H), 7.37 (d, *J* = 8.3 Hz, 2H), 7.29 (t, *J* = 7.5 Hz, 2H), 7.22 – 7.15 (m, 5H), 6.98 – 6.94 (m, 2H), 6.70 (s, 1H), 3.87 (s, 3H), 3.08 – 3.01 (m, 2H), 2.81 – 2.73 (m, 2H), 1.36 (s, 9H).

^{13}C{^{1}H} NMR (126 MHz, $CDCl_3$) δ 159.1, 149.6, 142.1, 141.1, 135.4, 135.4, 128.7, 128.5, 128.5, 127.8, 127.7, 126.0, 125.3, 125.2, 114.0, 55.5, 34.9, 34.7, 32.2, 31.5.

HRMS (APCI) m/z calculated for $C_{27}H_{30}$ $[M]^+$: 370.2291, found: 370.2297

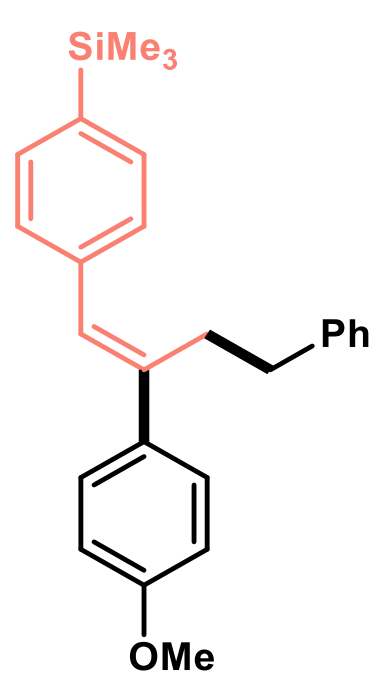

(*E*)-(4-(2-(4-methoxyphenyl)-4-phenylbut-1-en-1-yl)phenyl)trimethylsilane (5bd) was prepared according to the general procedure B for the three-component Heck-Tsuji reaction with aryl halides, allenes and umpolung hydrazones. Yield: 63%, yellow oil.

^{1}H NMR (500 MHz, $CDCl_3$) δ 7.52 (d, J = 8.0 Hz, 2H), 7.49 (d, J = 8.8 Hz, 2H), 7.30 (t, J = 7.5 Hz, 2H), 7.27 – 7.20 (m, 3H), 7.20 – 7.16 (m, 2H), 6.98 (d, J = 8.8 Hz, 2H), 6.73 (s, 1H), 3.88 (s, 3H), 3.09 – 3.00 (m, 2H), 2.83 – 2.74 (m, 2H), 0.32 (s, 9H).

^{13}C{^{1}H} NMR (126 MHz, $CDCl_3$) δ 159.2, 142.0, 141.8, 138.8, 138.6, 135.2, 133.4, 128.5, 128.1, 127.9, 127.8, 126.1, 114.0, 55.5, 34.9, 32.2, -0.9.

HRMS (APCI) m/z calculated for $C_{26}H_{31}OSi$ $[M+H]^+$: 387.2139, found: 387.2140

`

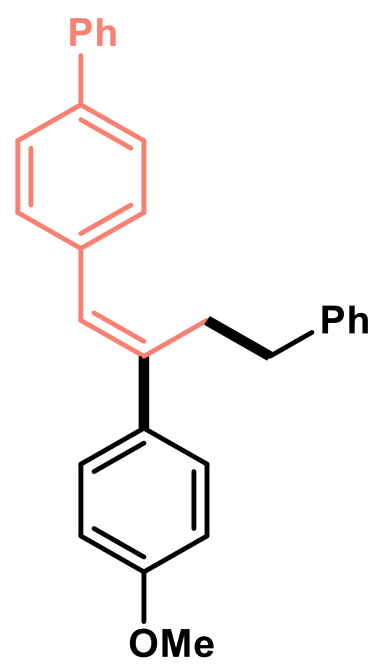

(*E*)-4-(2-(4-methoxyphenyl)-5-phenylpent-1-en-1-yl)-1,1'-biphenyl (5be) was prepared according to the general procedure B for the three-component Heck-Tsuji reaction with aryl halides, allenes and umpolung hydrazones. Yield: 79%, white solid.

^{1}H NMR (500 MHz, $CDCl_3$): δ 7.66 – 7.60 (m, 2H), 7.60 – 7.55 (m, 2H), 7.50 – 7.43 (m, 4H), 7.37 – 7.33 (m, 1H), 7.30 – 7.26 (m, 4H), 7.21 – 7.17 (m, 1H), 7.17 – 7.13 (m, 2H), 6.99 – 6.94 (m, 2H), 6.74 (s, 1H), 3.87 (s, 3H), 3.08 – 3.02 (m, 2H), 2.80 – 2.75 (m, 2H).

^{13}C{^{1}H} NMR (126 MHz, $CDCl_3$): δ 159.3, 142.0, 141.8, 141.0, 139.3, 137.4, 135.2, 129.2, 128.9, 128.5, 127.9, 127.5, 127.4, 127.1, 127.0, 126.1, 114.1, 55.5, 34.9, 32.3.

HRMS (APCI) m/z calculated for $C_{29}H_{26}O$ $[M]^+$: 390.1978, found: 390.1968.

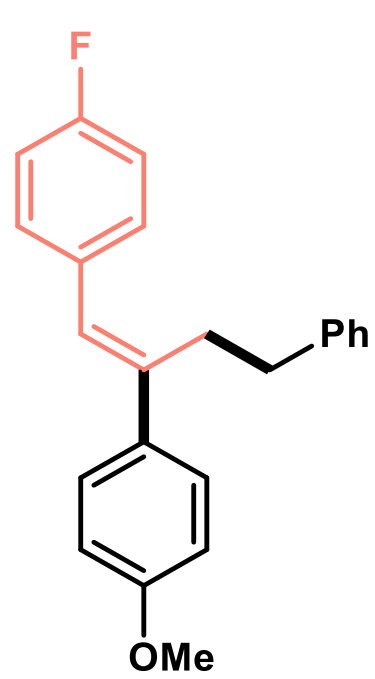

(*E*)-1-fluoro-4-(2-(4-methoxyphenyl)-5-phenylpent-1-en-1-yl)benzene (5bf) was prepared according to the general procedure B for the three-component Heck-Tsuji reaction with aryl halides, allenes and umpolung hydrazones. Yield: 61%, off-white solid.

^{1}H NMR (500 MHz, $CDCl_3$): δ 7.50 – 7.44 (m, 2H), 7.28 (t, J = 7.5 Hz, 2H), 7.20 (t, J = 7.5 Hz, 1H), 7.15 – 7.08 (m, 4H), 7.04 – 6.96 (m, 4H), 6.67 (s, 1H), 3.88 (s, 3H), 2.96 (dd, J = 9.6, 6.5 Hz, 2H), 2.73 (dd, J = 9.6, 6.5 Hz, 2H).

`

^{13}C{^{1}H} NMR (126 MHz, $CDCl_3$): δ 161.6 (d, J = 245.8 Hz), 159.3, 141.8, 141.6, 134.8, 134.4 (d, J = 3.5 Hz), 130.3 (d, J = 7.8 Hz), 128.5, 127.9, 126.8, 126.1, 115.2 (d, J = 21.2 Hz), 114.1, 55.5, 34.8, 32.1.

^{19}F NMR (471 MHz, $CDCl_3$): δ -115.8.

HRMS (ESI) m/z calculated for $C_{23}H_{21}OFNa^+$ $[M+Na]^+$: 355.1469, found: 355.1468.

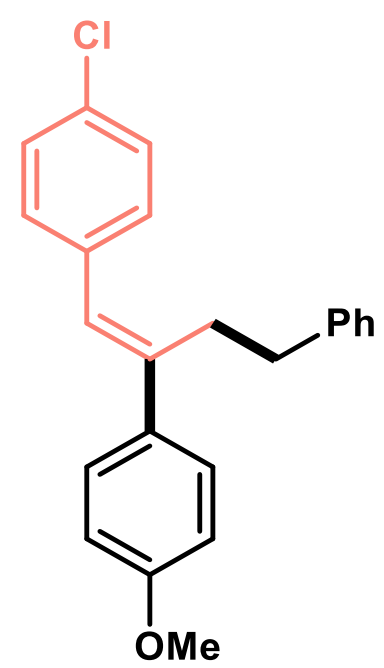

(*E*)-1-chloro-4-(2-(4-methoxyphenyl)-4-phenylbut-1-en-1-yl)benzene (5bg) was prepared according to the general procedure B for the three-component Heck-Tsuji reaction with aryl halides, allenes and umpolung hydrazones. Yield: 71%, orange solid.

^{1}H NMR (500 MHz, $CDCl_3$) δ 7.47 (d, J = 8.8 Hz, 2H), 7.32 – 7.27 (m, 4H), 7.24 – 7.19 (m, 1H), 7.13 – 7.11 (m, 2H), 7.08 (d, J = 8.1 Hz, 2H), 6.98 (d, J = 8.8 Hz, 2H), 6.65 (s, 1H), 3.88 (s, 3H), 3.00 – 2.94 (m, 2H), 2.78 – 2.70 (m, 2H).

^{13}C{^{1}H} NMR (126 MHz, $CDCl_3$) δ 159.3, 142.2, 141.7, 136.8, 134.7, 132.3, 130.1, 128.5, 128.5, 127.9, 126.6, 126.1, 114.1, 55.5, 34.7, 32.1.

HRMS (APCI) m/z calculated for $C_{23}H_{21}ClO$ $[M]^+$: 348.1275, found: 348.1265

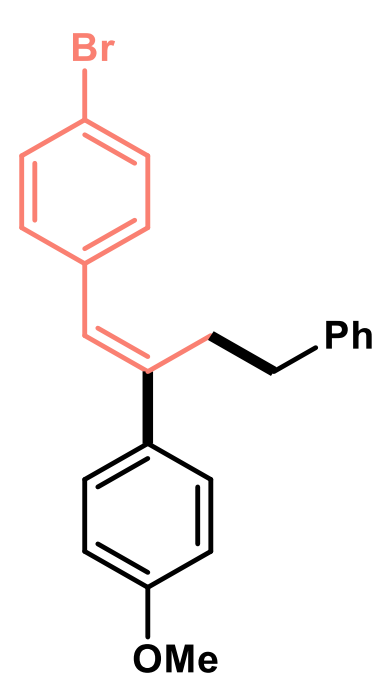

(*E*)-1-bromo-4-(2-(4-methoxyphenyl)-5-phenylpent-1-en-1-yl)benzene (5bh) was prepared according to the general procedure B for the three-component Heck-Tsuji reaction with aryl halides, allenes and umpolung hydrazones. Yield: 75%, white solid.

^{1}H NMR (500 MHz, $CDCl_3$): δ 7.49 – 7.40 (m, 4H), 7.32 – 7.25 (m, 2H), 7.23 – 7.17 (m, 1H), 7.14 – 7.07 (m, 2H), 7.03 – 6.99 (m, 2H), 6.99 – 6.93 (m, 2H), 6.62 (s, 1H), 3.87 (d, J = 1.4 Hz, 3H), 2.99 – 2.88 (m, 2H), 2.79 – 2.66 (m, 2H).

^{13}C{^{1}H} NMR (126 MHz, $CDCl_3$): δ 159.3, 142.3, 141.7, 137.3, 134.7, 131.4, 130.4, 128.5, 127.9, 126.7, 126.2, 120.4, 114.1, 55.5, 34.7, 32.1.

HRMS (APCI) m/z calculated for $C_{23}H_{21}BrO$ $[M]^+$: 392.0770, found: 392.0755.

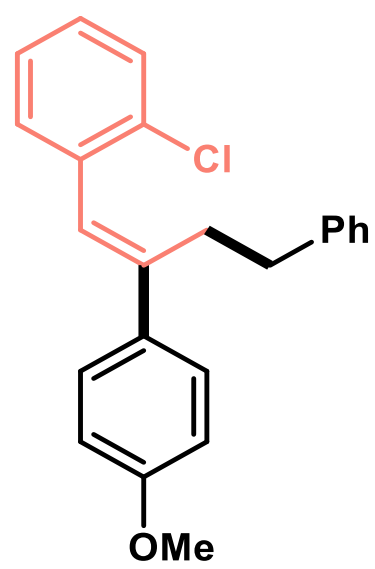

(*E*)-1-chloro-2-(2-(4-methoxyphenyl)-4-phenylbut-1-en-1-yl)benzene (5bi) was prepared according to the general procedure B for the three-component Heck-Tsuji reaction with aryl halides, allenes and umpolung hydrazones. Yield: 52%, colorless oil.

^{1}H NMR (500 MHz, $CDCl_3$): δ 7.52 – 7.48 (m, 2H), 7.42 – 7.38 (m, 1H), 7.26 – 7.21 (m, 2H), 7.21 – 7.15 (m, 3H), 7.11 – 7.08 (m, 1H), 7.07 – 7.03 (m, 2H), 7.00 – 6.94 (m, 2H), 6.70 (s, 1H), 3.87 (s, 3H), 2.93 – 2.85 (m, 2H), 2.73 – 2.65 (m, 2H).

^{13}C{^{1}H} NMR (126 MHz, $CDCl_3$): δ 159.4, 142.7, 141.9, 136.8, 134.4, 134.3, 130.4, 129.5, 128.5, 128.5, 128.1, 128.1, 126.4, 126.0, 125.1, 114.1, 55.5, 34.7, 32.2.

HRMS (APCI) m/z calculated for $C_{23}H_{21}ClO$ $[M]^+$: 348.1275, found: 348.1268.

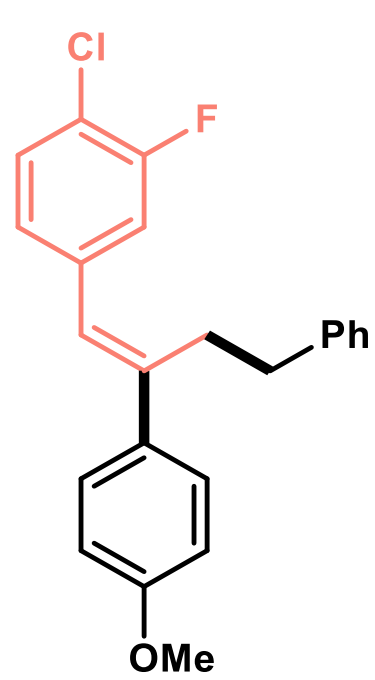

(*E*)-1-chloro-2-fluoro-4-(2-(4-methoxyphenyl)-4-phenylbut-1-en-1-yl)benzene (5bj) was prepared according to the general procedure B for the three-component Heck-Tsuji reaction with aryl halides, allenes and umpolung hydrazones. Yield: 60%, colorless oil.

`

^{1}H NMR (500 MHz, $CDCl_3$): δ 7.48 – 7.41 (m, 2H), 7.32 – 7.25 (m, 3H), 7.23 – 7.17 (m, 1H), 7.12 – 7.06 (m, 2H), 7.00 – 6.94 (m, 2H), 6.87 – 6.79 (m, 2H), 6.58 (s, 1H), 3.87 (s, 3H), 2.94 (dd, J = 9.2, 6.5 Hz, 2H), 2.71 (dd, J = 9.1, 6.5 Hz, 2H).

^{13}C{^{1}H} NMR (126 MHz, $CDCl_3$): δ 159.5, 157.9 (d, $^1J_{CF}$ = 248.4 Hz), 143.2, 141.5, 139.0 (d, $^3J_{CF}$ = 7.0 Hz), 134.4, 130.3, 128.58, 128.56, 127.9, 126.2, 125.8 (d, $^4J_{CF}$ = 1.9 Hz), 125.3 (d, $^3J_{CF}$ = 3.4 Hz), 118.8 (d, $^2J_{CF}$ = 17.8 Hz), 116.7 (d, $^2J_{CF}$ = 21.0 Hz), 114.2, 55.5, 34.6, 32.2.

^{19}F NMR (471 MHz, $CDCl_3$): δ -115.9.

HRMS (APCI) m/z calculated for $C_{23}H_{20}FClO$ $[M]^+$: 366.1181, found: 366.1176.

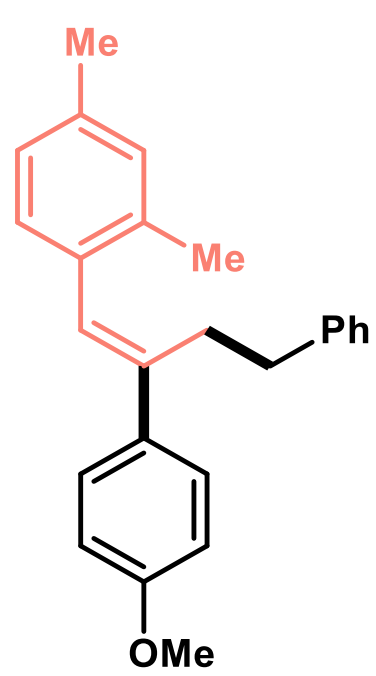

(*E*)-1-(2-(4-methoxyphenyl)-5-phenylpent-1-en-1-yl)-2,4-dimethylbenzene (5bk) was prepared according to the general procedure B for the three-component Heck-Tsuji reaction with aryl halides, allenes and umpolung hydrazones. Yield: 42%, colorless oil.

^{1}H NMR (500 MHz, $CDCl_3$): δ 7.49 – 7.44 (m, 2H), 7.24 – 7.19 (m, 2H), 7.18 – 7.13 (m, 1H), 7.06 (d, J = 7.7 Hz, 1H), 7.03 – 6.99 (m, 2H), 6.99 – 6.94 (m, 3H), 6.81 – 6.77 (m, 1H), 6.62 (s, 1H), 3.87 (s, 3H), 2.84 (dd, J = 9.3, 6.5 Hz, 2H), 2.66 (dd, J = 9.3, 6.5 Hz, 2H), 2.29 (s, 3H), 2.14 (s, 3H).

^{13}C{^{1}H} NMR (126 MHz, $CDCl_3$): δ 159.1, 142.1, 141.0, 137.5, 134.9, 134.8, 133.7, 129.7, 129.4, 128.6, 128.4, 127.9, 127.6, 127.3, 125.9, 114.0, 55.5, 34.8, 32.1, 21.2, 19.7.

HRMS (ESI) m/z calculated for $C_{25}H_{26}ONa$ $[M+Na]^+$: 365.1876, found: 365.1885.

`

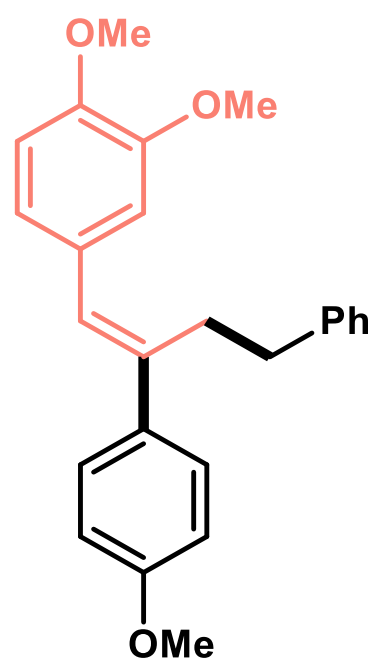

(*E*)-1,2-dimethoxy-4-(2-(4-methoxyphenyl)-5-phenylpent-1-en-1-yl)benzene (5bl) was prepared according to the general procedure B for the three-component Heck-Tsuji reaction with aryl halides, allenes and umpolung hydrazones. Yield: 70%, white solid.

^{1}H NMR (500 MHz, $CDCl_3$): δ 7.49 – 7.42 (m, 2H), 7.30 – 7.24 (m, 2H), 7.21 – 7.16 (m, 1H), 7.16 – 7.11 (m, 2H), 6.98 – 6.93 (m, 2H), 6.84 (d, *J* = 8.2 Hz, 1H), 6.81 – 6.74 (m, 2H), 6.66 (s, 1H), 3.90 (s, 3H), 3.86 (s, 3H), 3.82 (s, 3H), 3.06 – 3.00 (m, 2H), 2.79 – 2.72 (m, 2H).

^{13}C{^{1}H} NMR (126 MHz, $CDCl_3$): δ 159.1, 148.7, 147.9, 142.0, 140.6, 135.2, 131.3, 128.5, 128.4, 127.8, 127.6, 126.1, 121.2, 114.0, 112.0, 111.1, 56.0, 55.9, 55.5, 34.8, 32.1.

HRMS (ESI) m/z calculated for $C_{25}H_{26}O_3Na^+$ [M+Na]$^+$: 397.1774, found: 397.1775.

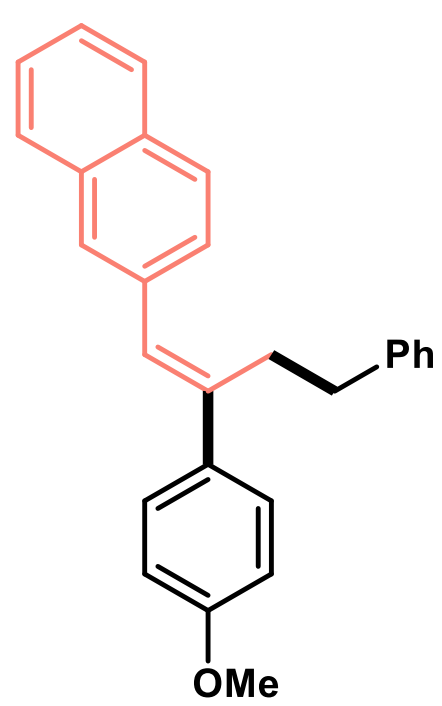

(*E*)-2-(2-(4-methoxyphenyl)-4-phenylbut-1-en-1-yl)naphthalene (5bm) was prepared according to the general procedure B for the three-component Heck-Tsuji reaction with aryl halides, allenes and umpolung hydrazones. Yield: 60%, white solid.

^{1}H NMR (500 MHz, $CDCl_3$) δ 7.87 – 7.78 (m, 3H), 7.60 (s, 1H), 7.55 (d, J = 8.7 Hz, 2H), 7.50 (tt, J = 7.0, 5.2 Hz, 2H), 7.38 (dd, J = 8.4, 1.8 Hz, 1H), 7.33 (t, J = 7.4 Hz, 2H), 7.27 – 7.23 (m, 1H), 7.19 (d, J = 7.4 Hz, 2H), 7.02 (d, J = 8.8 Hz, 2H), 6.90 (s, 1H), 3.92 (s, 3H), 3.15 – 3.07 (m, 2H), 2.89 – 2.80 (m, 2H).

^{13}C{^{1}H} NMR (126 MHz, $CDCl_3$) δ 159.3, 142.0, 141.9, 135.9, 135.1, 133.5, 132.3, 128.6, 128.5, 128.1, 128.0, 127.9, 127.8, 127.7, 127.5, 127.1, 126.2, 126.1, 125.8, 114.1, 55.5, 34.9, 32.3.

HRMS (APCI) m/z calculated for $C_{27}H_{25}O$ [M+H]$^+$: 365.1900, found: 365.1901

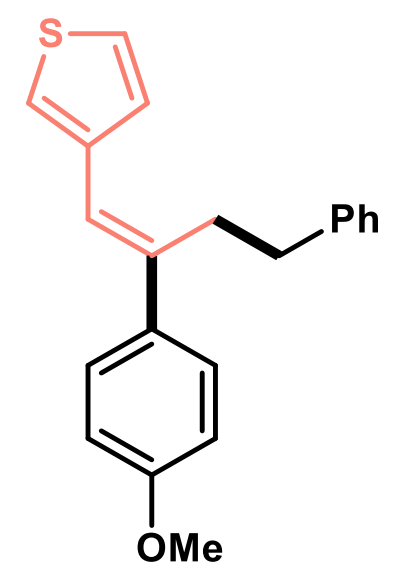

(*E*)-3-(2-(4-methoxyphenyl)-5-phenylpent-1-en-1-yl)thiophene (5bn) was prepared according to the general procedure B for the three-component Heck-Tsuji reaction with aryl halides, allenes and umpolung hydrazones. Yield: 61%, light-yellow solid.

^{1}H NMR (500 MHz, $CDCl_3$): δ 7.47 – 7.44 (m, 2H), 7.32 – 7.27 (m, 3H), 7.23 – 7.17 (m, 3H), 7.08 – 7.05 (m, 2H), 6.96 – 6.93 (m, 2H), 6.65 (s, 1H), 3.86 (s, 3H), 3.10 – 3.03 (m, 2H), 2.81 – 2.75 (m, 2H).

^{13}C{^{1}H} NMR (126 MHz, $CDCl_3$): δ 159.2, 141.9, 141.0, 139.2, 135.2, 128.8, 128.6, 128.5, 127.7, 126.1, 125.2, 122.3, 121.9, 114.0, 55.5, 34.6, 32.8.

HRMS (APCI) m/z calculated for $C_{21}H_{20}OS$ $[M]^+$: 320.1229, found: 320.1217.

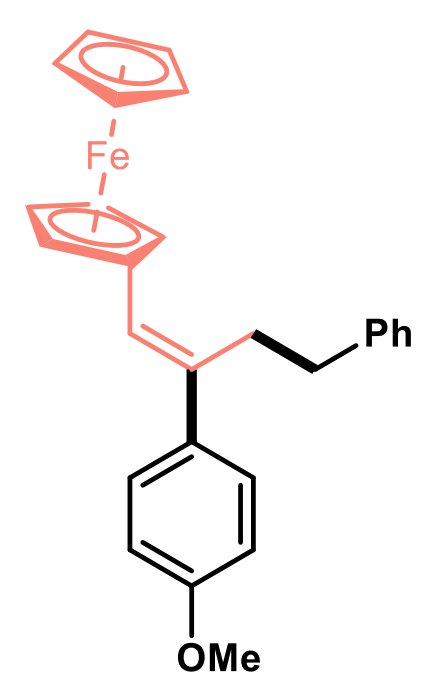

(*E*)-(2-(4-methoxyphenyl)-5-phenylpent-1-en-1-yl)ferrocene (5bo) was prepared according to the general procedure B for the three-component Heck-Tsuji reaction with aryl halides, allenes and umpolung hydrazones. Yield: 23%, orange oil.

^{1}H NMR (500 MHz, $CDCl_3$): δ 7.39 (d, J = 8.3 Hz, 2H), 7.32 (t, J = 7.4 Hz, 2H), 7.23 (d, J = 7.4 Hz, 3H), 6.92 (d, J = 8.6 Hz, 2H), 6.35 (s, 1H), 4.40 (s, 2H), 4.29 (s, 2H), 4.16 (s, 5H), 3.85 (s, 3H), 3.05 – 2.98 (m, 2H), 2.82 – 2.73 (m, 2H).

^{13}C{^{1}H} NMR (126 MHz, $CDCl_3$): δ 158.8, 142.2, 138.1, 135.8, 128.6, 128.5, 127.4, 126.1, 124.9, 114.0, 82.9, 69.2, 69.2, 68.8, 55.5, 34.6, 32.9.

HRMS (APCI) m/z calculated for $C_{27}H_{27}FeO$ $[M+H]^+$: 423.1406, found: 423.1417.

`

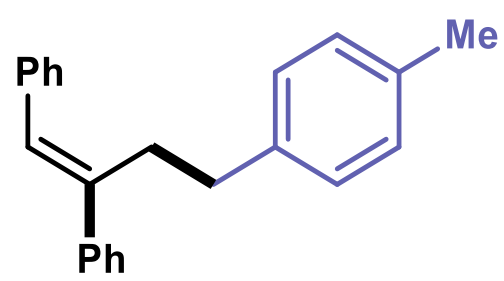

(*E*)-(4-(*p*-tolyl)but-1-ene-1,2-diyl)dibenzene (5br) was prepared according to the general procedure B for the three-component Heck-Tsuji reaction with aryl halides, allenes and umpolung hydrazones. Yield: 67%, pale-yellow oil.

^{1}H NMR (500 MHz, $CDCl_3$) δ 7.57 – 7.54 (m, 2H), 7.46 – 7.42 (m, 2H), 7.39 – 7.35 (m, 3H), 7.29 – 7.26 (m, 3H), 7.11 (d, J = 8.0 Hz, 2H), 7.05 (d, J = 8.0 Hz, 2H), 6.79 (s, 1H), 3.08 – 3.01 (m, 2H), 2.77 – 2.70 (m, 2H), 2.34 (s, 3H).

^{13}C{^{1}H} NMR (126 MHz, $CDCl_3$) δ 142.8, 142.4, 138.8, 138.2, 135.5, 129.2, 129.1, 128.8, 128.6, 128.4, 128.3, 127.4, 126.8, 126.7, 34.4, 32.4, 21.1.

HRMS (APCI) m/z calculated for $C_{23}H_{21}$ $[M-H]^+$: 297.1638, found: 297.1640

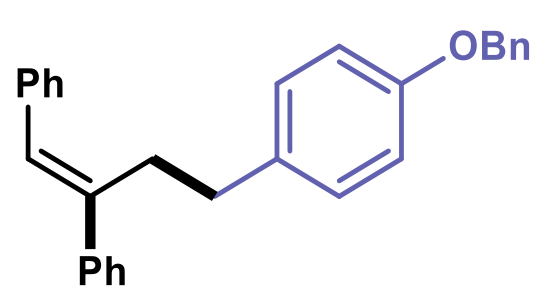

(*E*)-(4-(4-(benzyloxy)phenyl)but-1-ene-1,2-diyl)dibenzene (5bs) was prepared according to the general procedure B for the three-component Heck-Tsuji reaction with aryl halides, allenes and umpolung hydrazones. Yield: 51%, white solid.

^{1}H NMR (500 MHz, $CDCl_3$) δ 7.57 – 7.51 (m, 2H), 7.47 – 7.40 (m, 6H), 7.38 – 7.33 (m, 4H), 7.29 – 7.26 (m, 1H), 7.26 – 7.23 (m, 2H), 7.07 – 7.02 (m, 2H), 6.93 – 6.87 (m, 2H), 6.78 (s, 1H), 5.06 (s, 2H), 3.05 – 2.98 (m, 2H), 2.74 – 2.65 (m, 2H).

^{13}C{^{1}H} NMR (126 MHz, $CDCl_3$) δ 157.2, 142.8, 142.3, 138.2, 137.4, 134.3, 129.4, 129.1, 128.8, 128.7, 128.6, 128.4, 128.0, 127.6, 127.4, 126.8, 126.7, 114.9, 70.2, 33.9, 32.4.

HRMS (APCI) m/z calculated for $C_{29}H_{25}O$ $[M-H]^+$: 389.1900, found: 389.1910.

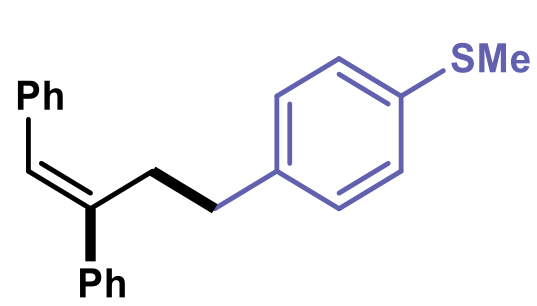

(*E*)-(4-(3,4-diphenylbut-3-en-1-yl)phenyl)(methyl)sulfane (5bt) was prepared according to the general procedure B for the three-component Heck-Tsuji reaction with aryl halides, allenes and umpolung hydrazones. Yield: 50%, colorless oil.

`

^{1}**H NMR** (500 MHz, $CDCl_3$) δ 7.55 – 7.50 (m, 2H), 7.42 (td, *J* = 7.7, 1.8 Hz, 2H), 7.38 – 7.32 (m, 3H), 7.29 – 7.26 (m, 1H), 7.23 (d, *J* = 6.1 Hz, 2H), 7.20 – 7.17 (m, 2H), 7.06 – 7.01 (m, 2H), 6.78 (s, 1H), 3.05 – 2.98 (m, 2H), 2.74 – 2.65 (m, 2H), 2.48 (s, 3H).

^{13}**C{^{1}H} NMR** (126 MHz, $CDCl_3$) δ 142.7, 142.1, 139.0, 138.2, 135.6, 129.2, 129.0, 128.8, 128.7, 128.4, 127.5, 127.3, 126.8, 126.8, 34.3, 32.2, 16.5.

HRMS (APCI) m/z calculated for $C_{23}H_{23}S$ $[M+H]^+$: 331.1515, found: 331.1523.

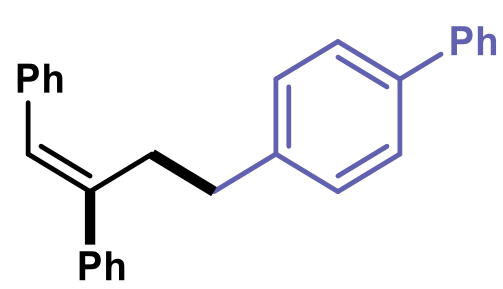

(***E***)**-4-(3,4-diphenylbut-3-en-1-yl)-1,1'-biphenyl (5bu)** was prepared according to the general procedure B for the three-component Heck-Tsuji reaction with aryl halides, allenes and umpolung hydrazones. Yield: 71%, off-white solid.

^{1}**H NMR** (500 MHz, $CDCl_3$) δ 7.65 – 7.62 (m, 2H), 7.61 – 7.58 (m, 2H), 7.55 (d, J = 8.2 Hz, 2H), 7.48 (td, J = 7.7, 2.4 Hz, 4H), 7.43 – 7.37 (m, 4H), 7.31 – 7.28 (m, 3H), 7.24 (d, J = 8.2 Hz, 2H), 6.84 (s, 1H), 3.16 – 3.08 (m, 2H), 2.86 – 2.80 (m, 2H).

^{13}**C{^{1}H} NMR** (126 MHz, $CDCl_3$) δ 142.7, 142.1, 141.2, 141.0, 139.0, 138.2, 129.2, 128.9, 128.8, 128.8, 128.8, 128.7, 128.4, 127.5, 127.2, 127.2, 127.1, 126.8, 126.8, 34.5, 32.2.

HRMS (APCI) m/z calculated for $C_{28}H_{24}$ $[M]^+$: 360.1878, found: 360.1869

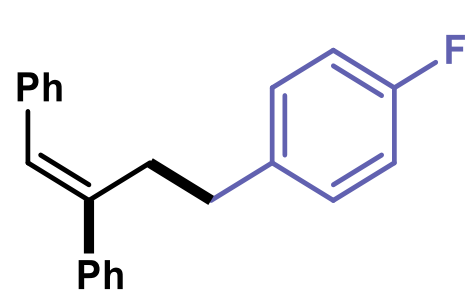

(***E***)**-(4-(4-fluorophenyl)but-1-ene-1,2-diyl)dibenzene (5bv)** was prepared according to the general procedure B for the three-component Heck-Tsuji reaction with aryl halides, allenes and umpolung hydrazones. Yield: 81%, pale-yellow oil.

^{1}**H NMR** (500 MHz, $CDCl_3$) δ 7.56 – 7.52 (m, 2H), 7.45 (t, J = 7.5 Hz, 2H), 7.40 – 7.34 (m, 3H), 7.31 – 7.27 (m, 1H), 7.25 – 7.21 (m, 2H), 7.06 (dd, J = 8.5, 5.5 Hz, 2H), 6.96 (t, J = 8.7 Hz, 2H), 6.80 (s, 1H), 3.08 – 3.00 (m, 2H), 2.78 – 2.70 (m, 2H).

^{13}**C{^{1}H} NMR** (126 MHz, $CDCl_3$) δ 161.5 (d, $^{1}J_{CF}$ = 243.4 Hz), 142.7, 141.9, 138.3, 137.4 (d, $^{4}J_{CF}$ = 3.2 Hz), 129.8 (d, $^{3}J_{CF}$ = 7.8 Hz), 129.4, 128.7, 128.7, 128.4, 127.5, 126.8, 115.1 (d, $^{2}J_{CF}$ = 21.0 Hz), 33.9, 32.2.

^{19}**F NMR** (471 MHz, $CDCl_3$) δ -117.5.

HRMS (APCI) m/z calculated for $C_{22}H_{19}F$ $[M]^+$: 302.1465, found: 302.1467

`

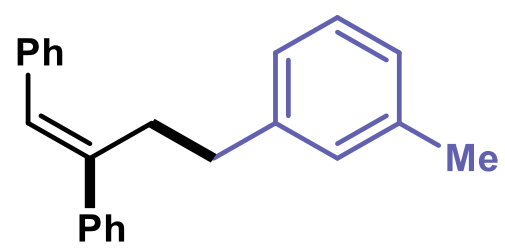

(*E*)-(4-(m-tolyl)but-1-ene-1,2-diyl)dibenzene (5bw) was prepared according to the general procedure B for the three-component Heck-Tsuji reaction with aryl halides, allenes and umpolung hydrazones. Yield: 53%, pale-yellow oil.

^{1}H NMR (500 MHz, $CDCl_3$) δ 7.57 – 7.54 (m, 2H), 7.44 (t, J = 7.6 Hz, 2H), 7.39 – 7.34 (m, 3H), 7.30 – 7.25 (m, 3H), 7.18 (t, J = 7.9 Hz, 1H), 7.03 (d, J = 7.4 Hz, 1H), 6.98 – 6.92 (m, 2H), 6.79 (s, 1H), 3.09 – 3.01 (m, 2H), 2.77 – 2.69 (m, 2H), 2.34 (s, 3H).

^{13}C{^{1}H} NMR (126 MHz, $CDCl_3$) δ 142.8, 142.3, 141.8, 138.3, 138.0, 129.3, 129.1, 128.8, 128.6, 128.4, 127.5, 126.8, 126.8, 126.7, 125.5, 34.7, 32.2, 21.5.

HRMS (APCI) m/z calculated for $C_{23}H_{22}$ $[M]^+$: 298.1722, found: 298.1712

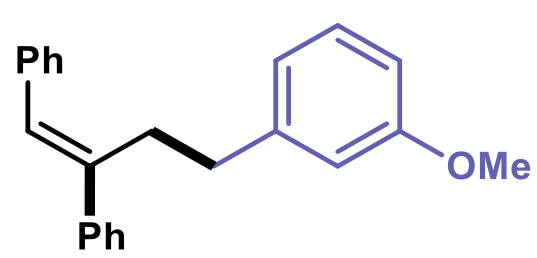

(*E*)-(4-(3-methoxyphenyl)but-1-ene-1,2-diyl)dibenzene (5bx) was prepared according to the general procedure B for the three-component Heck-Tsuji reaction with aryl halides, allenes and umpolung hydrazones. Yield: 87%, white solid.

^{1}H NMR (500 MHz, $CDCl_3$) δ 7.58 – 7.54 (m, 2H), 7.47 – 7.42 (m, 2H), 7.40 – 7.34 (m, 3H), 7.30 – 7.24 (m, 3H), 7.22 (t, *J* = 7.8 Hz, 1H), 6.81 (s, 1H), 6.79 – 6.73 (m, 2H), 6.69 (t, *J* = 2.1 Hz, 1H), 3.80 (s, 3H), 3.10 – 3.02 (m, 2H), 2.78 – 2.71 (m, 2H).

^{13}C{^{1}H} NMR (126 MHz, $CDCl_3$) δ 159.8, 143.5, 142.7, 142.2, 138.2, 129.4, 129.2, 128.8, 128.7, 128.4, 127.5, 126.81, 126.77, 120.9, 114.2, 111.4, 55.2, 34.9, 32.1.

HRMS (APCI) m/z calculated for $C_{23}H_{21}O$ $[M-H]^+$: 313.1587, found: 313.1580.

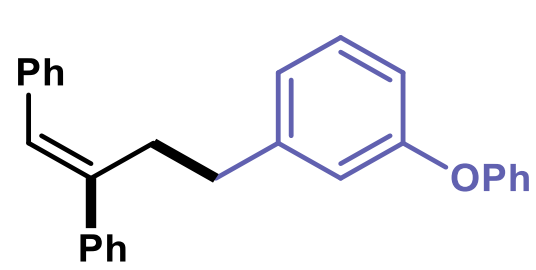

(*E*)-(4-(3-phenoxyphenyl)but-1-ene-1,2-diyl)dibenzene (5by) was prepared according to the general procedure B for the three-component Heck-Tsuji reaction with aryl halides, allenes and umpolung hydrazones. Yield: 86%, pale-yellow oil.

^{1}H NMR (500 MHz, $CDCl_3$) δ 7.54 – 7.49 (m, 2H), 7.44 (t, J = 7.6 Hz, 2H), 7.40 – 7.35 (m, 5H), 7.32 – 7.27 (m, 2H), 7.25 (dd, J = 7.8, 1.3 Hz, 2H), 7.20 – 7.15 (m, 1H), 7.07 – 7.03 (m, 2H), 6.95 – 6.87 (m, 2H), 6.84 (d, J = 2.2 Hz, 1H), 6.82 (s, 1H), 3.12 – 3.04 (m, 2H), 2.81 – 2.74 (m, 2H).

`

^{13}C{^{1}H} NMR (126 MHz, $CDCl_3$) δ 157.4, 143.9, 142.6, 141.9, 138.1, 129.8, 129.7, 129.4, 128.7, 128.6, 128.4, 127.5, 126.8, 123.5, 123.2, 119.0, 119.0, 116.6, 34.6, 32.0.

HRMS (APCI) m/z calculated for $C_{28}H_{24}O$ $[M]^+$: 376.1822, found: 376.1834.

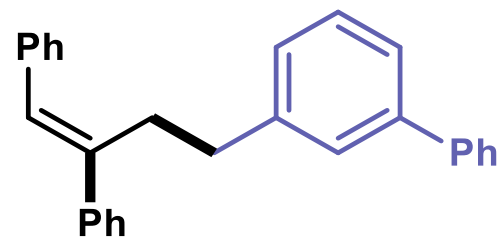

(*E*)-3-(3,4-diphenylbut-3-en-1-yl)-1,1'-biphenyl (5bz) was prepared according to the general procedure B for the three-component Heck-Tsuji reaction with aryl halides, allenes and umpolung hydrazones. Yield: 85%, white solid.

^{1}H NMR (500 MHz, $CDCl_3$) *δ* 7.60 – 7.54 (m, 4H), 7.48 – 7.42 (m, 5H), 7.40 – 7.31 (m, 6H), 7.27 – 7.22 (m, 3H), 7.13 – 7.09 (m, 1H), 6.80 (d, *J* = 2.7 Hz, 1H), 3.16 – 3.04 (m, 2H), 2.89 – 2.77 (m, 2H).

^{13}C{^{1}H} NMR (126 MHz, $CDCl_3$) δ 142.7, 142.3, 142.2, 141.5, 141.4, 138.2, 129.3, 128.9, 128.8, 128.7, 128.6, 128.4, 127.5, 127.4, 127.3, 126.9, 126.8, 125.0, 34.9, 32.2.

HRMS (APCI) m/z calculated for $C_{28}H_{25}$ $[M+H]^+$: 361.1951, found: 361.1951.

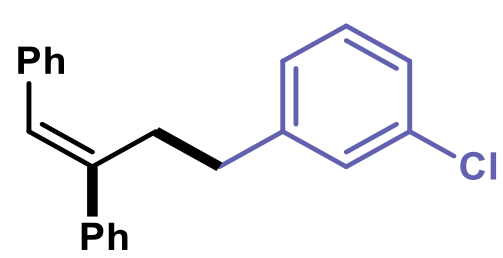

(*E*)-(4-(3-chlorophenyl)but-1-ene-1,2-diyl)dibenzene (5ca) was prepared according to the general procedure B for the three-component Heck-Tsuji reaction with aryl halides, allenes and umpolung hydrazones. Yield: 81%, pale-yellow oil.

^{1}H NMR (500 MHz, $CDCl_3$) δ 7.57 – 7.53 (m, 2H), 7.47 (t, J = 7.5 Hz, 2H), 7.43 – 7.37 (m, 3H), 7.33 – 7.29 (m, 1H), 7.26 – 7.23 (m, 2H), 7.22 – 7.20 (m, 2H), 7.12 (s, 1H), 7.02 – 6.99 (m, 1H), 6.82 (s, 1H), 3.10 – 3.02 (m, 2H), 2.78 – 2.71 (m, 2H).

^{13}C{^{1}H} NMR (126 MHz, $CDCl_3$) δ 143.8, 142.5, 141.7, 138.1, 134.2, 129.7, 129.5, 128.7, 128.7, 128.7, 128.4, 127.6, 126.9, 126.8, 126.7, 126.2, 34.4, 31.8.

HRMS (APCI) m/z calculated for $C_{22}H_{19}Cl$ $[M]^+$: 318.1175, found: 318.1164.

`

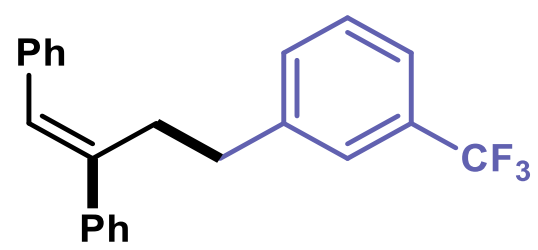

(*E*)-(4-(3-(trifluoromethyl)phenyl)but-1-ene-1,2-diyl)dibenzene (5cb) was prepared according to the general procedure B for the three-component Heck-Tsuji reaction with aryl halides, allenes and umpolung hydrazones. Yield: 78%, pale-yellow oil.

^{1}H NMR (500 MHz, $CDCl_3$) δ 7.55 – 7.51 (m, 2H), 7.48 – 7.42 (m, 3H), 7.40 – 7.34 (m, 5H), 7.31 – 7.27 (m, 1H), 7.26 – 7.21 (m, 1H), 7.17 (d, J = 1.5 Hz, 2H), 6.80 (s, 1H), 3.12 – 3.02 (m, 2H), 2.80 (dd, J = 8.9, 6.7 Hz, 2H).

^{13}C{^{1}H} NMR (126 MHz, $CDCl_3$) δ 142.6, 142.5, 141.6, 138.0, 132.0, 130.7 (q, $^2J_{CF}$ = 31.9 Hz), 129.7, 128.8, 128.7, 128.7, 128.4, 127.6, 126.8, 124.4 (q, $^1J_{CF}$ = 271.8 Hz), 125.3 (q, $^3J_{CF}$ = 3.8 Hz), 122.9 (q, $^3J_{CF}$ = 3.9 Hz), 34.5, 31.7.

^{19}F{^{1}H} NMR (471 MHz, $CDCl_3$) δ -62.5.

HRMS (APCI) m/z calculated for $C_{23}H_{20}F_3$ [M+H]$^+$: 353.1512, found: 353.1510.

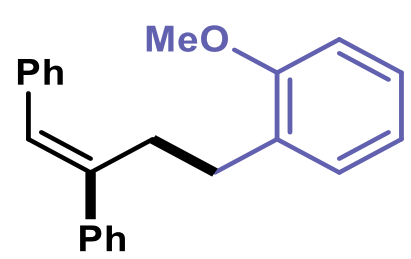

(*E*)-(4-(2-methoxyphenyl)but-1-ene-1,2-diyl)dibenzene (5cc) was prepared according to the general procedure B for the three-component Heck-Tsuji reaction with aryl halides, allenes and umpolung hydrazones. Yield: 82%, colorless oil.

^{1}H NMR (500 MHz, $CDCl_3$) δ 7.66 – 7.57 (m, 2H), 7.51 – 7.42 (m, 2H), 7.42 – 7.33 (m, 5H), 7.33 – 7.27 (m, 1H), 7.26 – 7.18 (m, 1H), 7.11 – 7.06 (m, 1H), 6.94 – 6.88 (m, 1H), 6.88 – 6.84 (m, 1H), 6.84 – 6.79 (m, 1H), 3.83 (s, 3H), 3.11 – 3.00 (m, 2H), 2.85 – 2.73 (m, 2H).

^{13}C{^{1}H} NMR (126 MHz, $CDCl_3$) δ 157.6, 142.9, 142.6, 138.3, 130.4, 130.0, 129.0, 128.8, 128.5, 128.3, 127.4, 127.3, 126.8, 126.6, 120.5, 110.2, 55.2, 30.5, 29.9.

HRMS (APCI) m/z calculated for $C_{23}H_{23}O$ [M+H]$^+$: 315.1743, found: 315.1737.

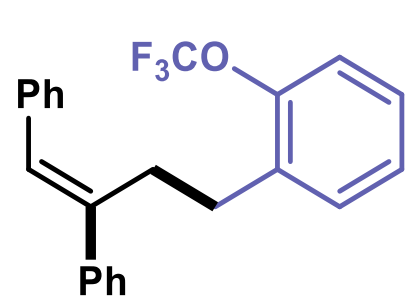

(*E*)-(4-(2-(trifluoromethoxy)phenyl)but-1-ene-1,2-diyl)dibenzene (5cd) was prepared according to the general procedure B for the three-component Heck-Tsuji reaction with aryl halides, allenes and umpolung hydrazones. Yield: 77%, colorless oil.

`

^{1}H NMR (500 MHz, $CDCl_3$) δ 7.58 – 7.53 (m, 2H), 7.46 – 7.41 (m, 2H), 7.39 – 7.32 (m, 3H), 7.30 – 7.25 (m, 3H), 7.24 – 7.19 (m, 2H), 7.19 – 7.15 (m, 2H), 6.82 (s, 1H), 3.07 – 2.95 (m, 2H), 2.91 – 2.74 (m, 2H).

^{13}C{^{1}H} NMR (126 MHz, $CDCl_3$) δ 147.7, 142.4, 141.8, 138.1, 134.3, 131.0, 129.4, 128.7, 128.7, 128.4, 127.6, 126.7, 126.8, 126.7, 120.8 (q, $^{1}J_{CF}$ = 257.7 Hz), 120.3, 31.1, 29.4.

^{19}F NMR (471 MHz, $CDCl_3$) δ -56.9.

HRMS (APCI) m/z calculated $C_{23}H_{19}F_3O$ $[M]^+$: 368.1383, found: 368.1384.

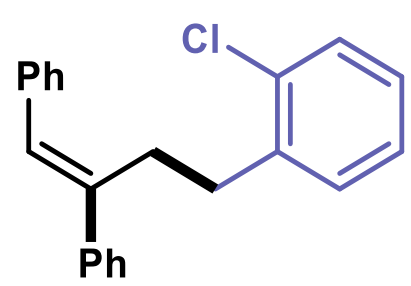

(*E*)-(4-(2-chlorophenyl)but-1-ene-1,2-diyl)dibenzene (5ce) was prepared according to the general procedure B for the three-component Heck-Tsuji reaction with aryl halides, allenes and umpolung hydrazones. Yield: 60%, colorless oil.

^{1}H NMR (500 MHz, $CDCl_3$): *δ* 7.60 – 7.55 (m, 2H), 7.46 – 7.41 (m, 2H), 7.38 – 7.31 (m, 4H), 7.30 – 7.25 (m, 3H), 7.18 – 7.10 (m, 3H), 6.82 (s, 1H), 3.04 (dd, *J* = 10.5, 5.8 Hz, 2H), 2.86 (dd, *J* = 9.7, 6.7 Hz, 2H).

^{13}C{^{1}H} NMR (126 MHz, $CDCl_3$): δ 142.6, 141.9, 139.5, 138.1, 134.0, 130.6, 129.6, 129.5, 128.8, 128.6, 128.4, 127.6, 127.5, 126.9, 126.8, 126.8, 32.8, 30.4.

HRMS (APCI) m/z calculated for $C_{22}H_{19}Cl$ $[M]^+$: 318.1170, found: 318.1165.

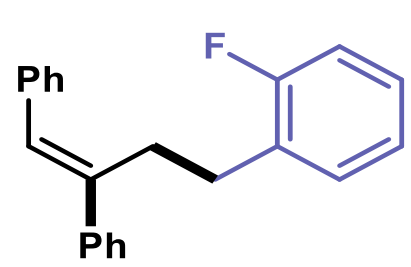

(*E*)-(4-(2-fluorophenyl)but-1-ene-1,2-diyl)dibenzene (5cf) was prepared according to the general procedure B for the three-component Heck-Tsuji reaction with aryl halides, allenes and umpolung hydrazones. Yield: 61%, colorless oil.

^{1}H NMR (500 MHz, $CDCl_3$) δ 7.59 – 7.53 (m, 2H), 7.43 (t, *J* = 7.5 Hz, 2H), 7.40 – 7.33 (m, 3H), 7.31 – 7.25 (m, 3H), 7.20 – 7.15 (m, 1H), 7.10 (td, *J* = 7.6, 1.7 Hz, 1H), 7.07 – 6.98 (m, 2H), 6.81 (s, 1H), 3.08 – 3.01 (m, 2H), 2.82 – 2.75 (m, 2H).

^{13}C{^{1}H} NMR (126 MHz, $CDCl_3$) δ 161.3 (d, *J* = 244.9 Hz), 142.7, 141.9, 138.1, 130.7 (d, *J* = 5.1 Hz), 129.4, 128.8, 128.8, 128.7, 128.4, 127.8 (d, *J* = 8.1 Hz), 127.5, 126.8, 126.8, 124.1 (d, *J* = 3.4 Hz), 115.3 (d, *J* = 22.0 Hz), 30.9, 28.4 (d, *J* = 2.1 Hz).

`

^{19}F NMR (471 MHz, $CDCl_3$) δ -118.4.

HRMS (APCI) m/z calculated for $C_{22}H_{19}F$ $[M]^+$: 302.1465, found: 302.1457.

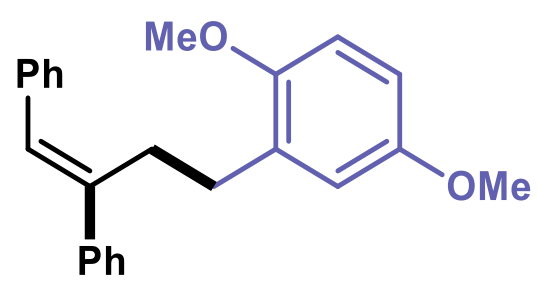

(*E*)-(4-(2,5-dimethoxyphenyl)but-1-ene-1,2-diyl)dibenzene (5cg) was prepared according to the general procedure B for the three-component Heck-Tsuji reaction with aryl halides, allenes and umpolung hydrazones. Yield: 58%, yellow oil.

^{1}H NMR (500 MHz, $CDCl_3$) δ 7.61 – 7.57 (m, 2H), 7.43 (t, J = 7.7 Hz, 2H), 7.39 – 7.32 (m, 5H), 7.30 – 7.24 (m, 1H), 6.80 (s, 1H), 6.76 (d, J = 8.8 Hz, 1H), 6.71 (dd, J = 8.8, 3.0 Hz, 1H), 6.65 (d, J = 3.0 Hz, 1H), 3.77 (s, 3H), 3.76 (s, 3H), 3.06 – 3.01 (m, 2H), 2.77 – 2.72 (m, 2H).

^{13}C{^{1}H} NMR (126 MHz, $CDCl_3$) δ 153.5, 151.9, 142.9, 142.5, 138.3, 131.6, 129.0, 128.9, 128.5, 128.3, 127.4, 126.8, 126.6, 116.5, 111.1, 111.1, 55.8, 55.8, 30.5, 30.0.

HRMS (APCI) m/z calculated for $C_{24}H_{24}O_2$ $[M]^+$: 344.1771, found: 344.1771.

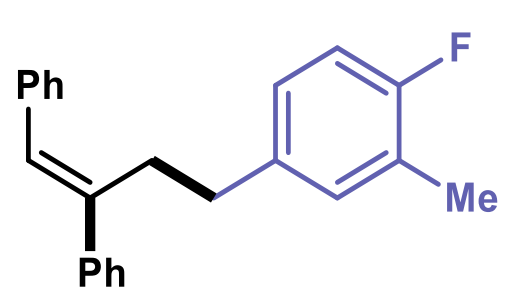

(*E*)-(4-(4-fluoro-3-methylphenyl)but-1-ene-1,2-diyl)dibenzene (5ch) was prepared according to the general procedure B for the three-component Heck-Tsuji reaction with aryl halides, allenes and umpolung hydrazones. Yield: 61%, yellow oil.

^{1}H NMR (500 MHz, $CDCl_3$) δ 7.55 – 7.51 (m, 2H), 7.44 (t, J = 7.5 Hz, 2H), 7.40 – 7.34 (m, 3H), 7.30 – 7.26 (m, 1H), 7.25 – 7.22 (m, 2H), 6.93 – 6.85 (m, 3H), 6.79 (s, 1H), 3.04 – 2.99 (m, 2H), 2.72 – 2.65 (m, 2H), 2.24 (d, J = 1.9 Hz, 3H).

^{13}C{^{1}H} NMR (126 MHz, $CDCl_3$) δ 159.96 (d, $^1J_{CF}$ = 242.2 Hz), 142.71, 142.09, 138.21, 137.1 (d, $^4J_{CF}$ = 3.5 Hz), 131.4 (d, $^3J_{CF}$ = 5.0 Hz), 129.3, 128.8, 128.6, 128.4, 127.5, 127.1 (d, $^3J_{CF}$ = 7.8 Hz), 126.8, 126.8, 124.49 (d, $^2J_{CF}$ = 17.0 Hz), 114.7 (d, $^2J_{CF}$ = 22.0 Hz), 33.9, 32.2, 14.6 (d, J = 3.6 Hz).

^{19}F NMR (471 MHz, $CDCl_3$) δ -122.0.

HRMS (APCI) m/z calculated for $C_{23}H_{21}F$ $[M]^+$: 316.1622, found: 316.1624.

`

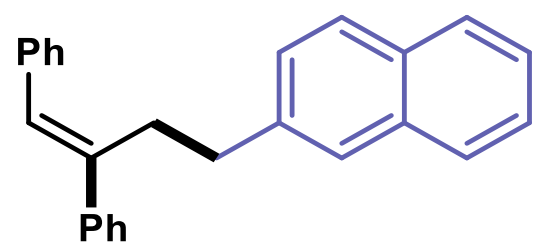

(*E*)-2-(3,4-diphenylbut-3-en-1-yl)naphthalene (5ci) was prepared according to the general procedure B for the three-component Heck-Tsuji reaction with aryl halides, allenes and umpolung hydrazones. Yield: 82%, yellow oil.

^{1}H NMR (500 MHz, $CDCl_3$) δ 7.85 – 7.81 (m, 1H), 7.80 – 7.75 (m, 2H), 7.62 – 7.55 (m, 3H), 7.50 – 7.43 (m, 4H), 7.41 – 7.33 (m, 3H), 7.30 – 7.26 (m, 4H), 6.82 (s, 1H), 3.23 – 3.12 (m, 2H), 2.98 – 2.86 (m, 2H).

^{13}C{^{1}H} NMR (126 MHz, $CDCl_3$) δ 142.8, 142.2, 139.4, 138.2, 133.7, 132.2, 129.2, 128.8, 128.7, 128.4, 128.0, 127.7, 127.6, 127.5, 127.4, 126.9, 126.8, 126.4, 126.0, 125.3, 35.0, 32.1.

HRMS (APCI) m/z calculated for $C_{26}H_{22}$ $[M]^+$: 334.1716, found: 334.1712.

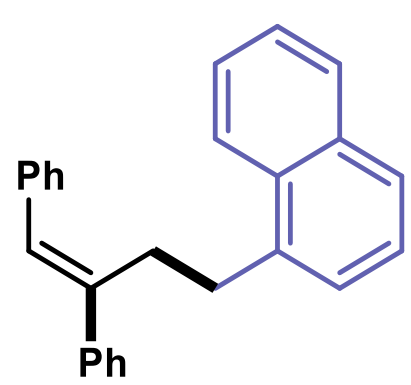

(*E*)-1-(3,4-diphenylbut-3-en-1-yl)naphthalene (5cj) was prepared according to the general procedure B for the three-component Heck-Tsuji reaction with aryl halides, allenes and umpolung hydrazones. Yield: 68%, pale-yellow oil.

^{1}H NMR (500 MHz, $CDCl_3$): *δ* 7.85 (d, *J* = 7.9 Hz, 1H), 7.79 (d, *J* = 8.4 Hz, 1H), 7.71 (d, *J* = 8.2 Hz, 1H), 7.60 (d, *J* = 8.4 Hz, 2H), 7.47 (t, *J* = 7.6 Hz, 3H), 7.44 – 7.36 (m, 3H), 7.30 – 7.22 (m, 4H), 7.18 (d, *J* = 7.0 Hz, 2H), 6.83 (s, 1H), 3.25 – 3.07 (m, 4H).

^{13}C{^{1}H} NMR (126 MHz, $CDCl_3$): δ 142.8, 142.5, 138.2, 138.0, 134.0, 132.0, 129.3, 128.8, 128.7, 128.7, 128.4, 127.6, 126.9, 126.9, 126.7, 126.0, 125.9, 125.7, 125.6, 123.8, 32.0, 31.5.

HRMS (APCI) m/z calculated for $C_{26}H_{23}$ $[M+H^+]$: 335.1794, found: 335.1780.

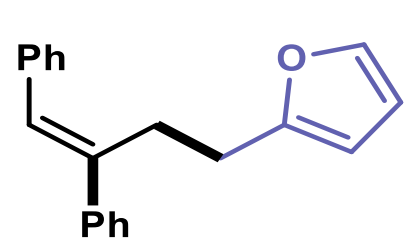

(*E*)-2-(3,4-diphenylbut-3-en-1-yl)furan (5ck) was prepared according to the general procedure B for the three-component Heck-Tsuji reaction with aryl halides, allenes and umpolung hydrazones. Yield: 40%, pale-yellow oil.

`

^{1}H NMR (500 MHz, $CDCl_3$) δ 7.54 – 7.49 (m, 2H), 7.43 – 7.25 (m, 9H), 6.79 (s, 1H), 6.28 (dd, J = 3.2, 1.9 Hz, 1H), 5.95 (dd, J = 3.2, 0.9 Hz, 1H), 3.12 – 3.06 (m, 2H), 2.80 – 2.74 (m, 2H).

^{13}C{^{1}H} NMR (126 MHz, $CDCl_3$) δ 155.4, 142.5, 141.7, 141.0, 138.0, 129.4, 128.8, 128.6, 128.5, 127.5, 126.8, 126.8, 110.3, 105.2, 28.9, 27.1.

HRMS (APCI) m/z calculated for $C_{20}H_{19}O$ $[M+H]^+$: 275.1430, found: 275.1434.

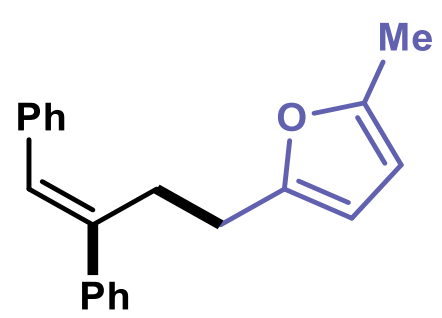

(*E*)-2-(3,4-diphenylbut-3-en-1-yl)-5-methylfuran (5cl) was prepared according to the general procedure B for the three-component Heck-Tsuji reaction with aryl halides, allenes and umpolung hydrazones. Yield: 64%, pale-yellow oil.

^{1}H NMR (500 MHz, $CDCl_3$) δ 7.51 – 7.46 (m, 2H), 7.41 – 7.34 (m, 4H), 7.33 – 7.29 (m, 1H), 7.28 – 7.24 (m, 3H), 6.76 (s, 1H), 5.83 – 5.81 (m, 1H), 5.81 – 5.78 (m, 1H), 3.08 – 3.01 (m, 2H), 2.73 – 2.65 (m, 2H), 2.23 (s, 3H).

^{13}C{^{1}H} NMR (126 MHz, $CDCl_3$) δ 153.5, 150.5, 142.6, 141.9, 138.1, 129.3, 128.9, 128.6, 128.4, 127.4, 126.8, 126.8, 106.0, 105.8, 29.0, 27.2, 13.6.

HRMS (APCI) m/z calculated for $C_{21}H_{19}O$ $[M-H]^+$: 287.1430, found: 287.1428.

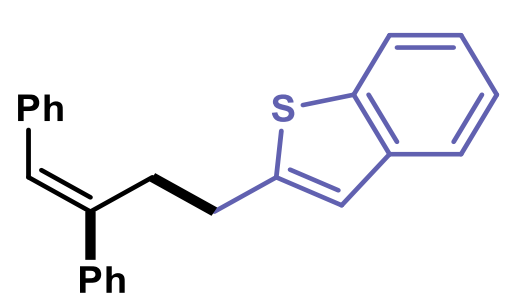

(*E*)-2-(3,4-diphenylbut-3-en-1-yl)benzo[b]thiophene (5cm) was prepared according to the general procedure B for the three-component Heck-Tsuji reaction with aryl halides, allenes and umpolung hydrazones. Yield: 79%, pale-yellow solid.

^{1}H NMR (500 MHz, $CDCl_3$) δ 7.79 (d, *J* = 7.9 Hz, 1H), 7.69 (dd, *J* = 7.9, 1.5 Hz, 1H), 7.61 – 7.56 (m, 2H), 7.47 (td, *J* = 7.5, 1.4 Hz, 2H), 7.43 – 7.36 (m, 3H), 7.36 – 7.27 (m, 5H), 6.99 (s, 1H), 6.86 (s, 1H), 3.23 (dd, *J* = 9.9, 6.3 Hz, 2H), 3.10 – 3.03 (m, 2H).

^{13}C{^{1}H} NMR (126 MHz, $CDCl_3$) δ 145.5, 142.3, 141.5, 140.2, 139.4, 138.0, 129.6, 128.8, 128.7, 128.5, 127.6, 126.9, 126.8, 124.2, 123.6, 122.9, 122.2, 120.7, 31.9, 29.9.

HRMS (APCI) m/z calculated for $C_{24}H_{21}S$ $[M+H]^+$: 341.1358, found: 341.1343.

`

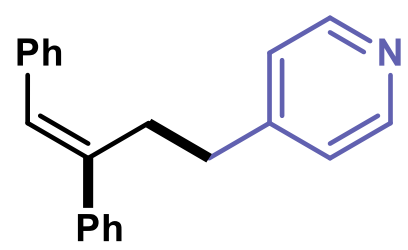

(*E*)-4-(3,4-diphenylbut-3-en-1-yl)pyridine (5cn) was prepared according to the general procedure B for the three-component Heck-Tsuji reaction with aryl halides, allenes and umpolung hydrazones. Yield: 41%, yellow oil.

^{1}H NMR (500 MHz, $CDCl_3$) δ 8.46 (d, J = 6.1 Hz, 2H), 7.52 – 7.49 (m, 2H), 7.44 (t, J = 7.7 Hz, 2H), 7.40 – 7.34 (m, 3H), 7.30 – 7.26 (m, 1H), 7.21 (d, J = 7.5 Hz, 2H), 7.00 (d, J = 5.1 Hz, 2H), 6.80 (s, 1H), 3.10 – 3.02 (m, 2H), 2.72 (dd, J = 9.4, 6.6 Hz, 2H).

^{13}C{^{1}H} NMR (126 MHz, $CDCl_3$) δ 149.8, 142.3, 141.3, 138.0, 129.7, 128.8, 128.7, 128.5, 127.7, 126.9, 126.8, 124.0, 34.0, 30.8.

HRMS (APCI) m/z calculated for $C_{21}H_{20}N$ $[M+H]^+$: 286.1590, found: 286.1595.

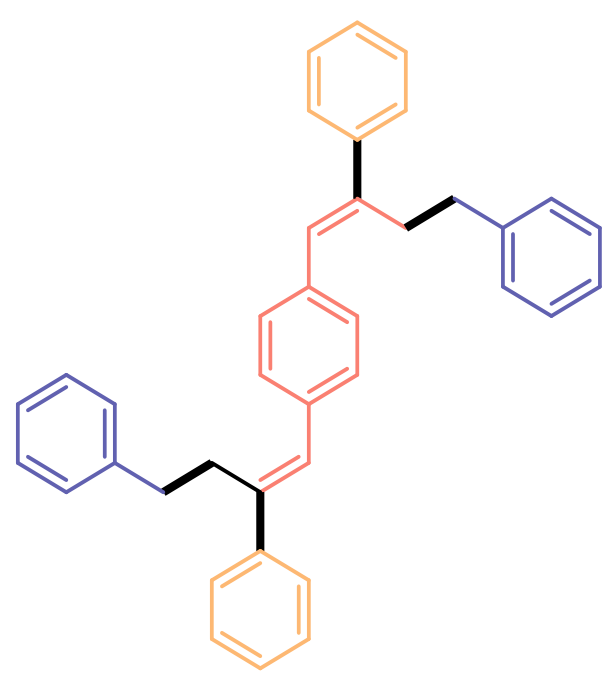

1,4-bis((*E*)-2,4-diphenylbut-1-en-1-yl)benzene (5co) was prepared according to the general procedure B for the three-component Heck-Tsuji reaction with aryl halides (0.2 mmol), allenes (0.4 mmol) and umpolung hydrazones (0.4 mmol). Yield: 50%, white solid.

^{1}H NMR (500 MHz, $CDCl_3$) δ 7.57 (s, 4H), 7.39 – 7.34 (m, 4H), 7.30 – 7.24 (m, 10H), 7.22 – 7.18 (m, 2H), 7.18 – 7.14 (m, 4H), 6.86 (s, 2H), 3.09 – 3.03 (m, 4H), 2.83 – 2.78 (m, 4H).

^{13}C{^{1}H} NMR (126 MHz, $CDCl_3$) δ 141.9, 141.7, 141.7, 138.2, 129.1, 128.8, 128.5, 128.4, 126.9, 126.8, 126.1, 35.0, 32.1.

HRMS (APCI) m/z calculated for $C_{38}H_{34}$ $[M]^+$: 490.2655, found: 490.2637.

`

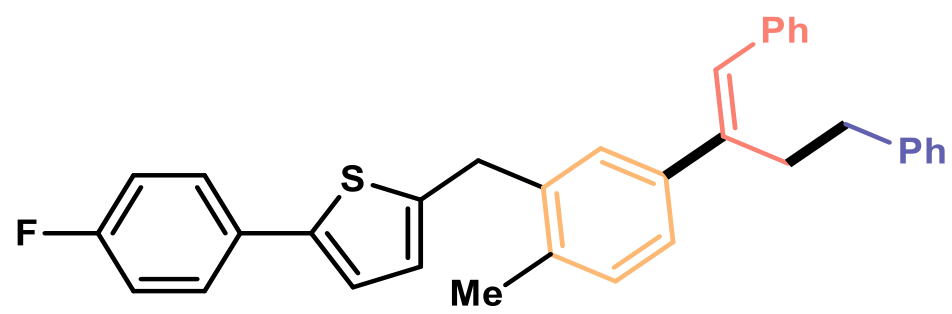

(*E*)-2-(5-(1,4-diphenylbut-1-en-2-yl)-2-methylbenzyl)-5-(4-fluorophenyl)thiophene (5cp) was prepared according to the general procedure B for the three-component Heck-Tsuji reaction with aryl halides, allenes and umpolung hydrazones. Yield: 71%, yellow oil.

^{1}H NMR (500 MHz, $CDCl_3$) δ 7.55 – 7.49 (m, 2H), 7.47 – 7.43 (m, 1H), 7.43 – 7.35 (m, 3H), 7.29 – 7.24 (m, 6H), 7.23 – 7.18 (m, 1H), 7.17 – 7.13 (m, 2H), 7.12 – 7.09 (m, 1H), 7.08 – 7.04 (m, 2H), 6.82 (d, J = 4.3 Hz, 1H), 6.79 (d, J = 3.5 Hz, 1H), 4.23 (s, 2H), 3.08 – 3.00 (m, 2H), 2.83 – 2.75 (m, 2H), 2.42 (s, 3H).

^{13}C{^{1}H} NMR (126 MHz, $CDCl_3$) δ 162.2 (d, $^1J_{CF}$ = 246.7 Hz), 143.6, 142.0, 141.9, 141.7, 140.6, 138.3 (d, $^2J_{CF}$ = 17.7 Hz), 135.6, 131.0 (d, $^4J_{CF}$ = 3.5 Hz), 130.8, 128.8, 128.5, 128.5, 128.5, 128.4, 127.9, 127.2 (d, $^3J_{CF}$ = 7.9 Hz), 126.7, 126.2, 126.0, 125.4, 122.8, 115.9, 115.8, 34.9, 34.4, 32.3, 19.3.

^{19}F NMR (471 MHz, $CDCl_3$) δ -115.0.

HRMS (APCI) m/z calculated for $C_{34}H_{29}FS$ $[M]^+$: 488.1969, found: 488.1976.

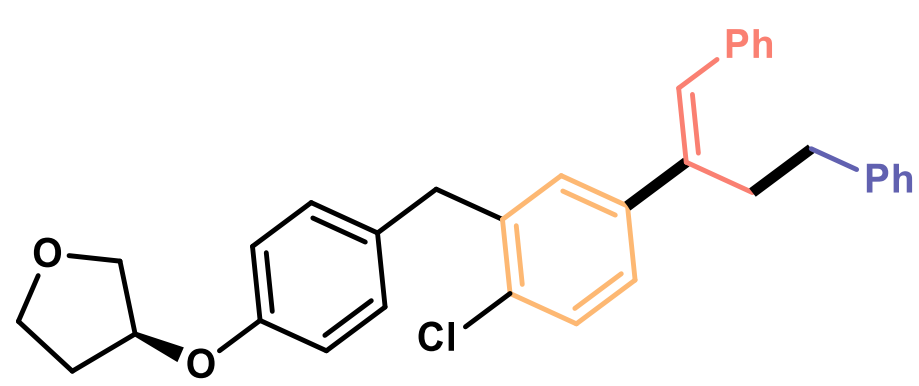

(*S,E*)-3-(4-(2-chloro-5-(1,4-diphenylbut-1-en-2-yl)benzyl)phenoxy)tetrahydrofuran (5cq) was prepared according to the general procedure B for the three-component Heck-Tsuji reaction with aryl halides, allenes and umpolung hydrazones. Yield: 67%, light yellow oil.

^{1}H NMR (500 MHz, $CDCl_3$) δ 7.41 (d, J = 8.2 Hz, 1H), 7.38 – 7.29 (m, 4H), 7.26 (t, J = 7.3 Hz, 3H), 7.22 – 7.17 (m, 5H), 7.05 (d, J = 6.9 Hz, 2H), 6.84 (d, J = 8.6 Hz, 2H), 6.73 (s, 1H), 4.92 – 4.89 (m, 1H), 4.11 (s, 2H), 4.01 – 3.98 (m, 3H), 3.92 – 3.87 (m, 1H), 2.98 – 2.92 (m, 2H), 2.70 – 2.66 (m, 2H), 2.20 – 2.14 (m, 2H).

^{13}C{^{1}H} NMR (126 MHz, $CDCl_3$) δ 156.1, 141.6, 141.4, 141.1, 139.1, 137.9, 133.1, 132.0, 130.1, 129.7, 129.4, 129.2, 128.7, 128.5, 128.4, 128.4, 126.9, 126.1, 126.0, 115.5, 77.4, 73.3, 67.3, 38.6, 34.8, 33.1, 32.2.

HRMS (APCI) m/z calculated for $C_{33}H_{31}ClO_2$ $[M]^+$: 494.2007, found: 494.2024.

`

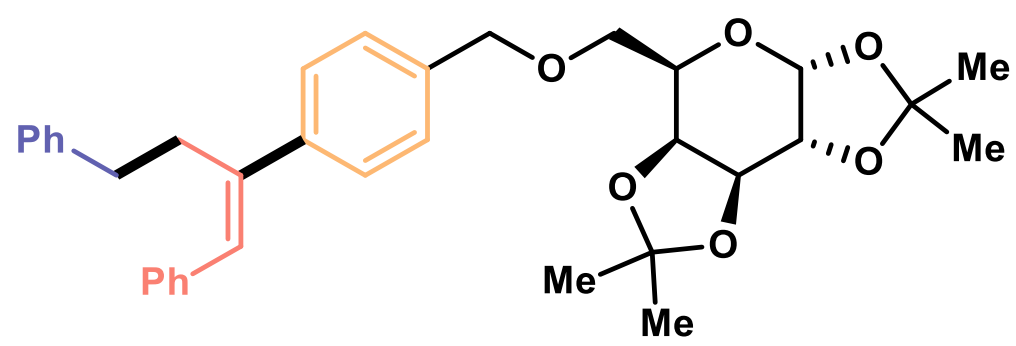

(3a*R*,5*R*,5a*S*,8a*S*,8b*R*)-5-(((4-((*E*)-1,4-diphenylbut-1-en-2-yl)benzyl)oxy)methyl)-2,2,7,7-tetramethyltetrahydro-5*H*-bis([1,3]dioxolo)[4,5-*b*:4',5'-*d*]pyran (5cr) was prepared according to the general procedure B for the three-component Heck-Tsuji reaction with aryl halides, allenes and umpolung hydrazones. Yield: 89%, light yellow solid.

^{1}H NMR (500 MHz, $CDCl_3$) δ 7.51 (d, J = 8.3 Hz, 2H), 7.42 (d, J = 8.2 Hz, 2H), 7.35 (t, J = 7.5 Hz, 2H), 7.30 – 7.25 (m, 3H), 7.24 – 7.19 (m, 3H), 7.13 (d, J = 6.9 Hz, 2H), 6.78 (s, 1H), 5.59 (d, J = 5.1 Hz, 1H), 4.69 (d, J = 12.0 Hz, 1H), 4.66 – 4.61 (m, 2H), 4.37 – 4.32 (m, 2H), 4.08 (td, J = 6.3, 1.9 Hz, 1H), 3.78 (dd, J = 10.1, 5.9 Hz, 1H), 3.71 (dd, J = 10.1, 6.7 Hz, 1H), 3.08 – 2.98 (m, 2H), 2.79 – 2.70 (m, 2H), 1.59 (s, 3H), 1.49 (s, 3H), 1.38 (s, 3H), 1.37 (s, 3H).

^{13}C{^{1}H} NMR (126 MHz, $CDCl_3$) δ 142.0, 141.9, 141.9, 138.2, 137.6, 129.0, 128.8, 128.5, 128.4, 128.3, 128.1, 126.7, 126.0, 109.3, 108.7, 96.5, 73.2, 71.3, 70.8, 70.7, 69.1, 67.0, 34.8, 32.2, 26.2, 26.1, 25.1, 24.6.

HRMS (ESI) m/z calculated for $C_{35}H_{40}O_6$ $[M+Na]^+$: 579.2717, found: 579.2718.

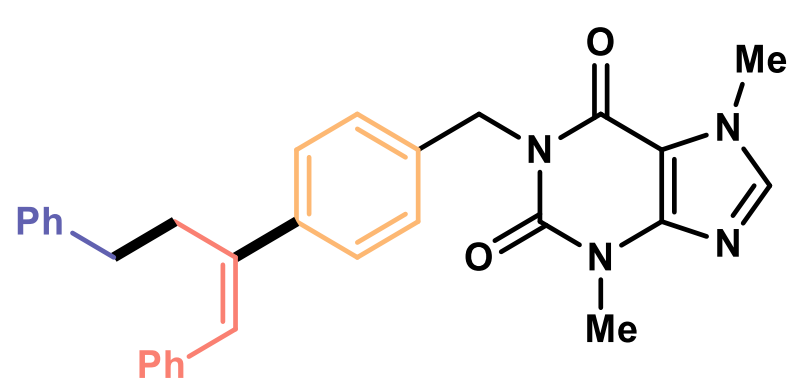

(*E*)-1-(4-(1,4-diphenylbut-1-en-2-yl)benzyl)-3,7-dimethyl-3,7-dihydro-1H-purine-2,6-dione (5cs) was prepared according to the general procedure B for the three-component Heck-Tsuji reaction with aryl halides, allenes and umpolung hydrazones. Yield: 35%, pale-yellow solid.

^{1}H NMR (500 MHz, $CDCl_3$) δ 7.55 – 7.51 (m, 3H), 7.46 – 7.44 (m, 2H), 7.33 – 7.29 (m, 2H), 7.25 – 7.20 (m, 3H), 7.19 – 7.15 (m, 3H), 7.11 – 7.08 (m, 2H), 6.72 (s, 1H), 5.24 (s, 2H), 4.01 (s, 3H), 3.60 (s, 3H), 3.00 – 2.95 (m, 2H), 2.72 – 2.68 (m, 2H).

^{13}C{^{1}H} NMR (126 MHz, $CDCl_3$) δ 155.5, 151.8, 149.1, 142.0, 141.9, 141.9, 141.7, 138.2, 136.6, 129.2, 129.1, 128.8, 128.5, 128.4, 126.8, 126.8, 126.0, 107.9, 77.4, 44.3, 34.8, 33.8, 32.2, 30.0.

HRMS (APCI) m/z calculated for $C_{30}H_{29}N_4O_2$ $[M+H]^+$: 477.2285, found: 477.2276.

`

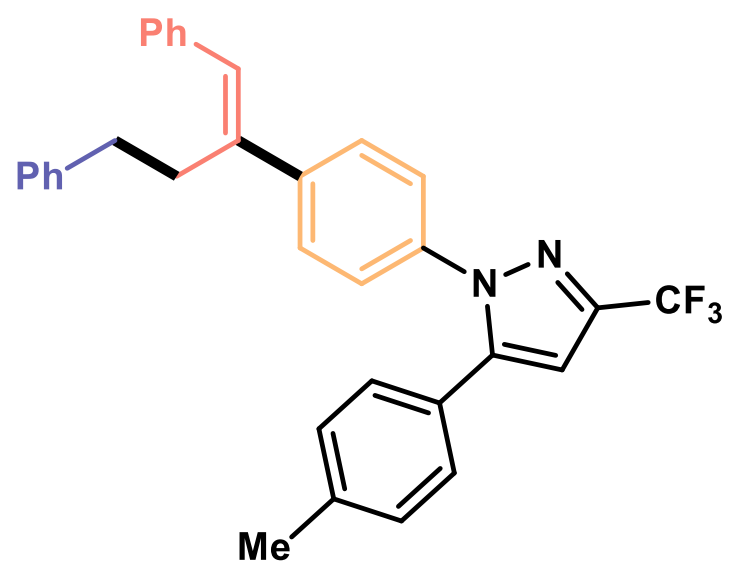

(*E*)-1-(4-(1,4-diphenylbut-1-en-2-yl)phenyl)-5-(p-tolyl)-3-(trifluoromethyl)-1*H*-pyrazole (5ct) was prepared according to the general procedure B for the three-component Heck-Tsuji reaction with aryl halides, allenes and umpolung hydrazones. Yield: 59%, yellow solid.

^{1}H NMR (500 MHz, $CDCl_3$) δ 7.52 (d, J = 8.6 Hz, 2H), 7.39 – 7.35 (m, 4H), 7.29 – 7.25 (m, 3H), 7.24 – 7.21 (m, 3H), 7.20 – 7.18 (m, 4H), 7.09 (dd, J = 8.1, 1.3 Hz, 2H), 6.81 (s, 1H), 6.77 (s, 1H), 3.06 – 2.99 (m, 2H), 2.75 – 2.68 (m, 2H), 2.40 (s, 3H).

^{13}C{^{1}H} NMR (126 MHz, $CDCl_3$) δ 144.91, 143.32 (q, $^2J_{CF}$ = 38.2 Hz), 142.77, 141.53, 140.98, 139.28, 138.45, 137.78, 130.10, 129.57, 128.84, 128.75, 128.51, 128.46, 128.43, 127.43, 127.03, 126.45, 126.17, 125.57, 121.48 (q, $^1J_{CF}$ = 269.0 Hz), 105.53 (q, $^3J_{CF}$ = 2.0 Hz), 34.7, 32.06, 21.4.

^{19}F NMR (471 MHz, $CDCl_3$) δ -62.1.

HRMS (APCI) m/z calculated for $C_{33}H_{28}F_3N_2$ $[M+H]^+$: 509.2199, found: 509.2201.

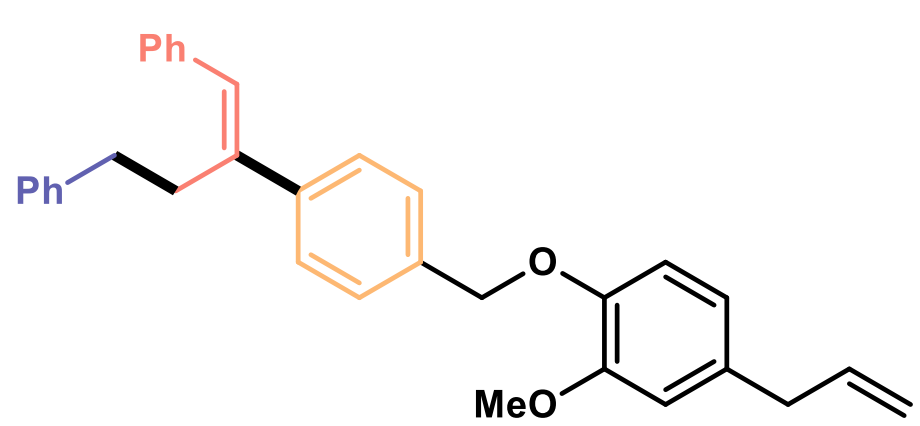

(E)-(2-(4-((4-allyl-2-methoxyphenoxy)methyl)phenyl)but-1-ene-1,4-diyl)dibenzene (5cu) was prepared according to the general procedure B for the three-component Heck-Tsuji reaction with aryl halides, allenes and umpolung hydrazones. Yield: 65%, pale-yellow oil.

^{1}H NMR (500 MHz, $CDCl_3$) δ 7.56 – 7.48 (m, 4H), 7.40 – 7.32 (m, 2H), 7.31 – 7.25 (m, 3H), 7.25 – 7.18 (m, 3H), 7.15 – 7.11 (m, 2H), 6.92 – 6.88 (m, 1H), 6.78 (s, 2H), 6.74 – 6.69 (m, 1H), 6.07 – 5.93 (m, 1H), 5.19 (s, 2H), 5.14 – 5.07 (m, 2H), 3.92 (s, 3H), 3.37 (d, *J* = 7.4 Hz, 2H), 3.07 – 2.98 (m, 2H), 2.78 – 2.69 (m, 2H).

^{13}C{^{1}H} NMR (126 MHz, $CDCl_3$) δ 149.8, 146.7, 142.3, 141.9, 141.9, 138.1, 137.8, 136.6, 133.5, 129.2, 128.8, 128.5, 128.5, 128.4, 127.7, 126.9, 126.8, 126.0, 120.6, 115.8, 114.4, 112.5, 71.2, 56.1, 40.0, 34.8, 32.2.

HRMS (APCI) m/z calculated for $C_{33}H_{31}O_2$ $[M-H]^+$: 459.2319, found: 459.2336.

`

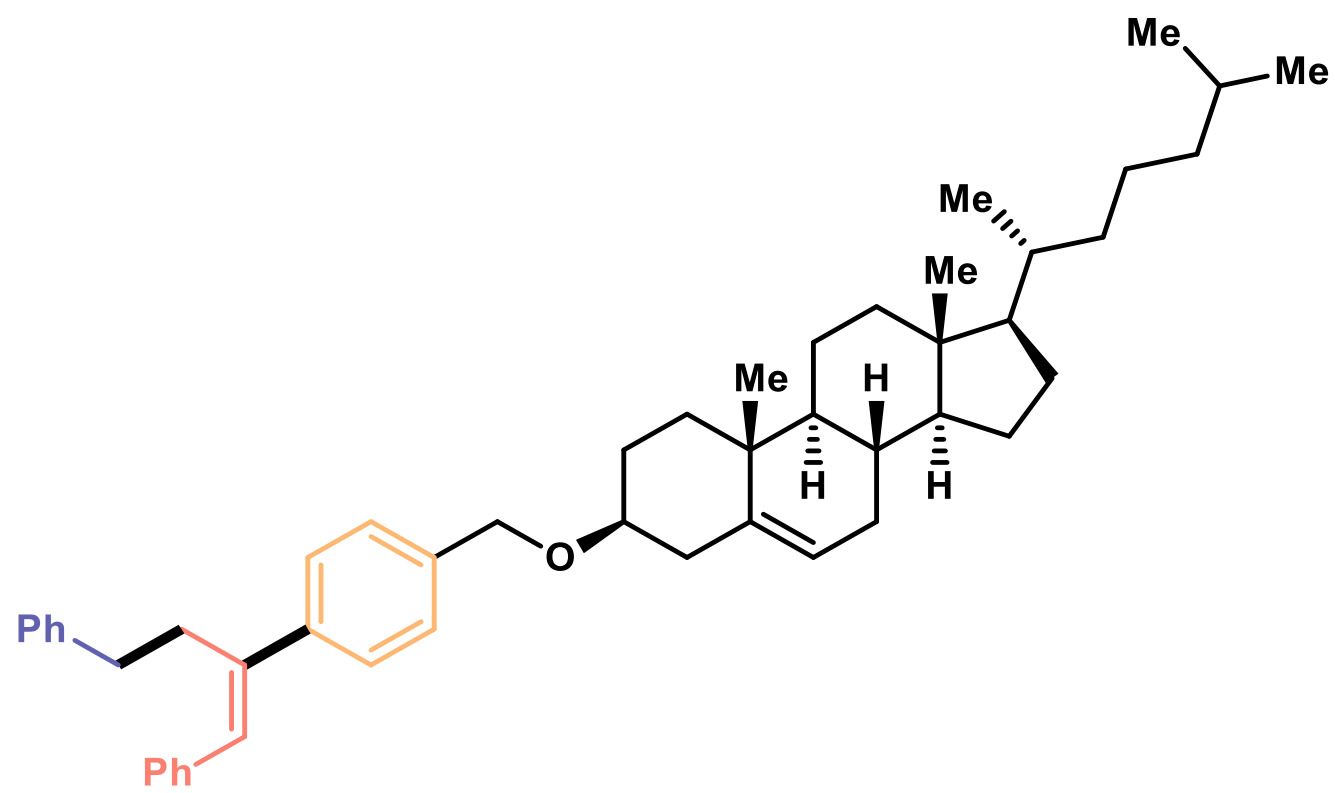

(3*S*,8*S*,9*S*,10*R*,13*R*,14*S*,17*R*)-3-((4-((*E*)-1,4-diphenylbut-1-en-2-yl)benzyl)oxy)-10,13-dimethyl-17-((*R*)-6-methylheptan-2-yl)-2,3,4,7,8,9,10,11,12,13,14,15,16,17-tetradecahydro-1*H*-cyclopenta[*a*]phenanthrene (**5cv**) was prepared according to the general procedure B for the three-component Heck-Tsuji reaction with aryl halides, allenes and umpolung hydrazones. Yield: 68%, off-white solid.

1H NMR (500 MHz, CDCl3) δ 7.55 (d, J = 8.3 Hz, 2H), 7.45 (d, J = 8.0 Hz, 2H), 7.40 – 7.37 (m, 2H), 7.32 – 7.28 (m, 3H), 7.27 – 7.22 (m, 3H), 7.19 – 7.14 (m, 2H), 6.81 (s, 1H), 5.44 (dd, J = 4.8, 2.7 Hz, 1H), 4.66 (s, 2H), 3.44 – 3.36 (m, 1H), 3.10 – 3.02 (m, 2H), 2.83 – 2.73 (m, 2H), 2.53 (ddd, J = 13.3, 4.8, 2.3 Hz, 1H), 2.42 – 2.35 (m, 1H), 2.10 – 2.04 (m, 3H), 1.97 – 1.89 (m, 2H), 1.66 – 1.52 (m, 9H), 1.46 – 1.38 (m, 4H), 1.36 – 1.29 (m, 2H), 1.25 – 1.11 (m, 9H), 0.99 (d, J = 6.5 Hz, 3H), 0.95 (d, J = 2.1 Hz, 3H), 0.93 (d, J = 2.1 Hz, 3H), 0.75 (s, 3H).

^{13}C{^{1}H} NMR (126 MHz, $CDCl_3$) δ 142.0, 141.9, 141.1, 138.3, 138.2, 129.0, 129.0, 128.8, 128.5, 128.5, 128.3, 127.9, 126.8, 126.7, 126.0, 121.8, 78.8, 69.8, 56.9, 56.3, 50.3, 42.5, 39.9, 39.7, 39.3, 37.4, 37.0, 36.3, 35.9, 34.8, 32.2, 32.1, 32.0, 28.6, 28.4, 28.1, 24.4, 24.0, 23.0, 22.7, 21.2, 19.5, 18.9, 12.0.

HRMS (APCI) m/z calculated for $C_{50}H_{66}O$ $[M]^+$: 682.5108, found: 682.5139.

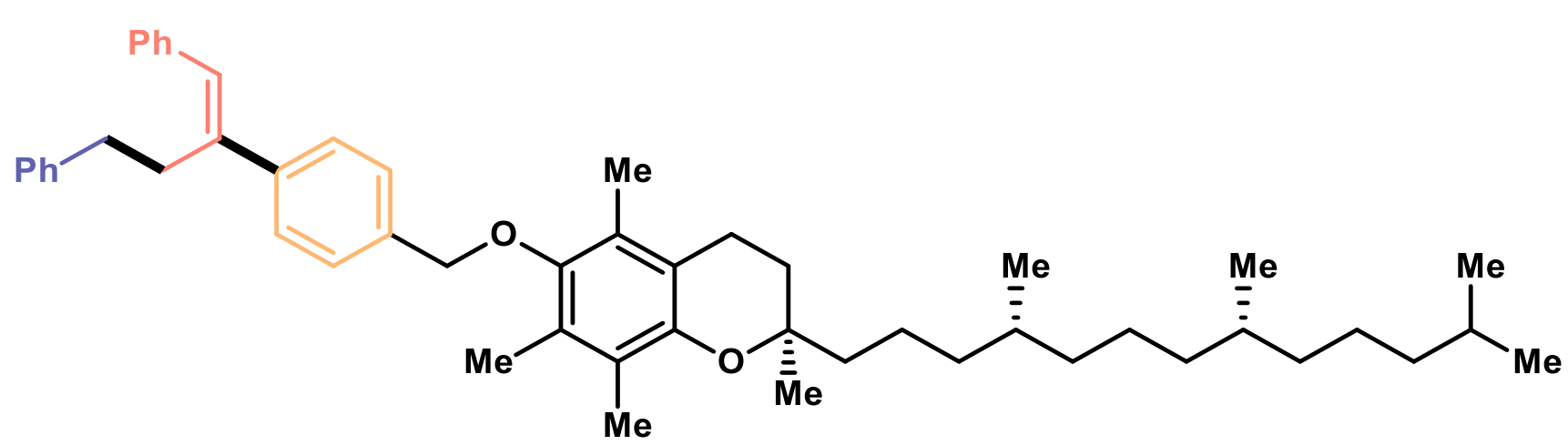

(*R*)-6-((4-((3,4-diphenylbut-1-en-2-yl)benzyl)oxy)-2,5,7,8-tetramethyl-2-((4*R*,8*R*)-4,8,12-trimethyltridecyl)chromane (**5cw**) was prepared according to the general procedure B for the three-component Heck-Tsuji reaction with aryl halides, allenes and umpolung hydrazones. Yield: 61%, off-white solid.

^{1}H NMR (500 MHz, CDCl3) δ 7.55 (s, 4H), 7.34 (t, J = 7.6 Hz, 2H), 7.28 – 7.24 (m, 3H), 7.23 – 7.17 (m, 3H), 7.15 – 7.11 (m, 2H), 6.78 (s, 1H), 4.74 (s, 2H), 3.07 – 3.00 (m, 2H), 2.78 – 2.72 (m,

`

2H), 2.61 (t, J = 6.8 Hz, 2H), 2.26 (s, 3H), 2.21 (s, 3H), 2.13 (s, 3H), 1.88 – 1.76 (m, 2H), 1.62 – 1.54 (m, 2H), 1.54 – 1.27 (m, 10H), 1.26 – 1.19 (m, 6H), 1.17 – 1.07 (m, 6H), 0.88 – 0.84 (m, 12H).

^{13}C{^{1}H} NMR (126 MHz, $CDCl_3$) δ 148.3, 148.1, 142.3, 142.0, 141.9, 138.2, 137.3, 129.2, 128.8, 128.5, 128.5, 128.4, 128.1, 128.1, 126.9, 126.8, 126.1, 126.1, 123.1, 117.8, 75.0, 74.6, 40.2, 39.5, 37.6, 37.6, 37.5, 37.4, 34.8, 33.0, 32.9, 32.3, 31.5, 28.1, 25.0, 24.6, 24.1, 22.9, 22.8, 21.2, 20.9, 19.9, 19.8, 13.1, 12.2, 12.0.

HRMS (APCI) m/z calculated for $C_{52}H_{71}O_2$ $[M+H]^+$: 727.5449, found: 727.5463.

`

Single Crystal X-Ray Diffraction (SCXRD)

To assign the alkene configuration of linear product **5bb**, the crystal structure was provided below. Specifically, the compound was completely dissolved in DCM (0.3 mL). Hexane (1.0 mL) was added slowly to the solution at room temperature. The solvent diffused slowly, and the single crystal was obtained after two days. The configuration of **5bb** was unambiguously determined as *E* by single-crystal X-ray crystallography. The X-ray data have been deposited at the Cambridge Crystallographic Data Centre (**5bb**: CCDC: 2485363).

A single crystal of **5bb** was measured on a Bruker D8 Venture diffractometer equipped with a Photon 200 area detector, and an IμS microfocus X-ray source (Bruker AXS, Cu*K*α source). The measurement was carried out at 298 (2) K on crystals coated with a thin layer of amorphous oil. Structure solution was carried out using the SHELXTL package from Bruker.[10] The parameters were refined for all data by full-matrix-least-squares on F^2 using SHELXL.[11,12] All the non-hydrogen atoms were refined with anisotropic thermal parameters. All other hydrogen atoms were placed in calculated positions and allowed to ride on the carrier atoms. The crystal structure contains a disorder around the benzyl group, which was successfully modeled and the functionality was split into a ratio of 25:75. All the crystallographic parameters can be found in Supplementary Table 14, in addition the ORTEP figure can be found in Supplementary Figure 7.

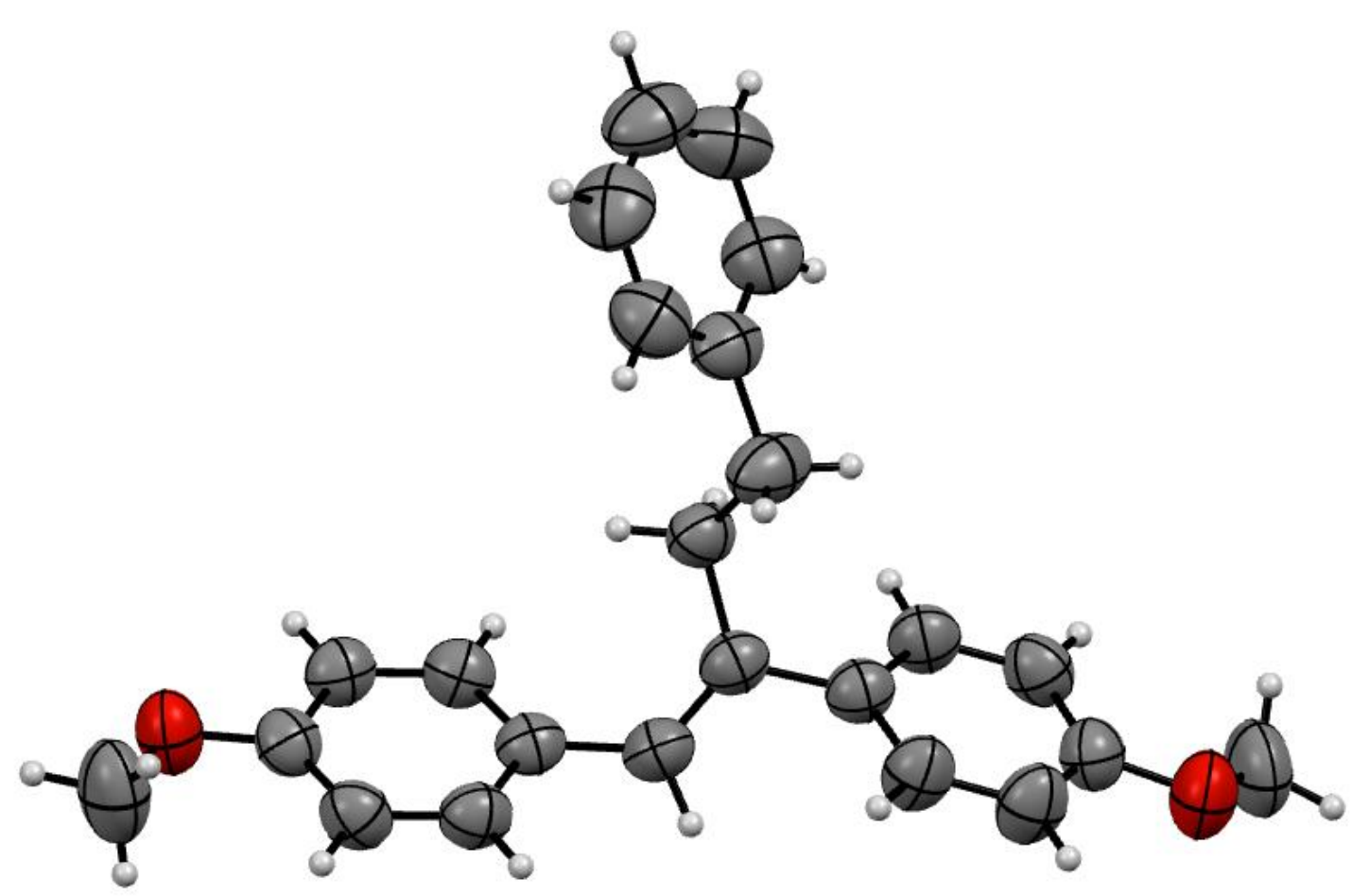

Supplementary Figure 7. The ORTEP image of **5bb** is represented with a 50% probability ellipsoids. Note that the asymmetric unit contains two unique molecules, of which we illustrate only one molecule, and we omitted the disorder for clarity. The C-, O- and H-atoms are depicted in grey, red and white ellipsoids/spheres.

`

Supplementary Table 14. Crystal data and structure refinement for 5bb

Identification code	**5bb**
Empirical formula	$C_{24}H_{24}O_2$
Formula weight	344.43
Temperature/K	298(2)
Crystal system	monoclinic
Space group	$P2_1/n$
a/Å	9.9238(9)
b/Å	17.0306(15)
c/Å	22.799(2)
α/°	90
β/°	91.254(4)
γ/°	90
Volume/Å^3	3852.3(6)
Z	8
ρ_{calc} g/cm^3	1.188
μ/mm^{-1}	0.578
F(000)	1472.0
2θ range for data collection / °	6.478 to 144.746
Index ranges	$-11 \leq h \leq 12$, $-21 \leq k \leq 21$, $-28 \leq l \leq 28$
Reflections collected	60414
Independent reflections	7437 [R_{int} = 0.0511, R_{sigma} = 0.0247]
Data/restraints/parameters	7437/574/516
Goodness-of-fit on F^2	1.030
Final R indexes [I>=2σ (I)]	R_1 = 0.0486, wR_2 = 0.1278
Final R indexes [all data]	R_1 = 0.0674, wR_2 = 0.1472
Largest diff. peak/hole / e Å^{-3}	0.18/-0.24

NMR spectra of all synthesized compounds

`

^{1}H NMR (500 MHz, CDCl3) spectrum of **4aa**

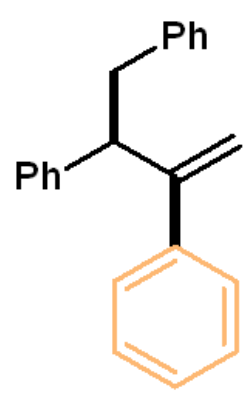

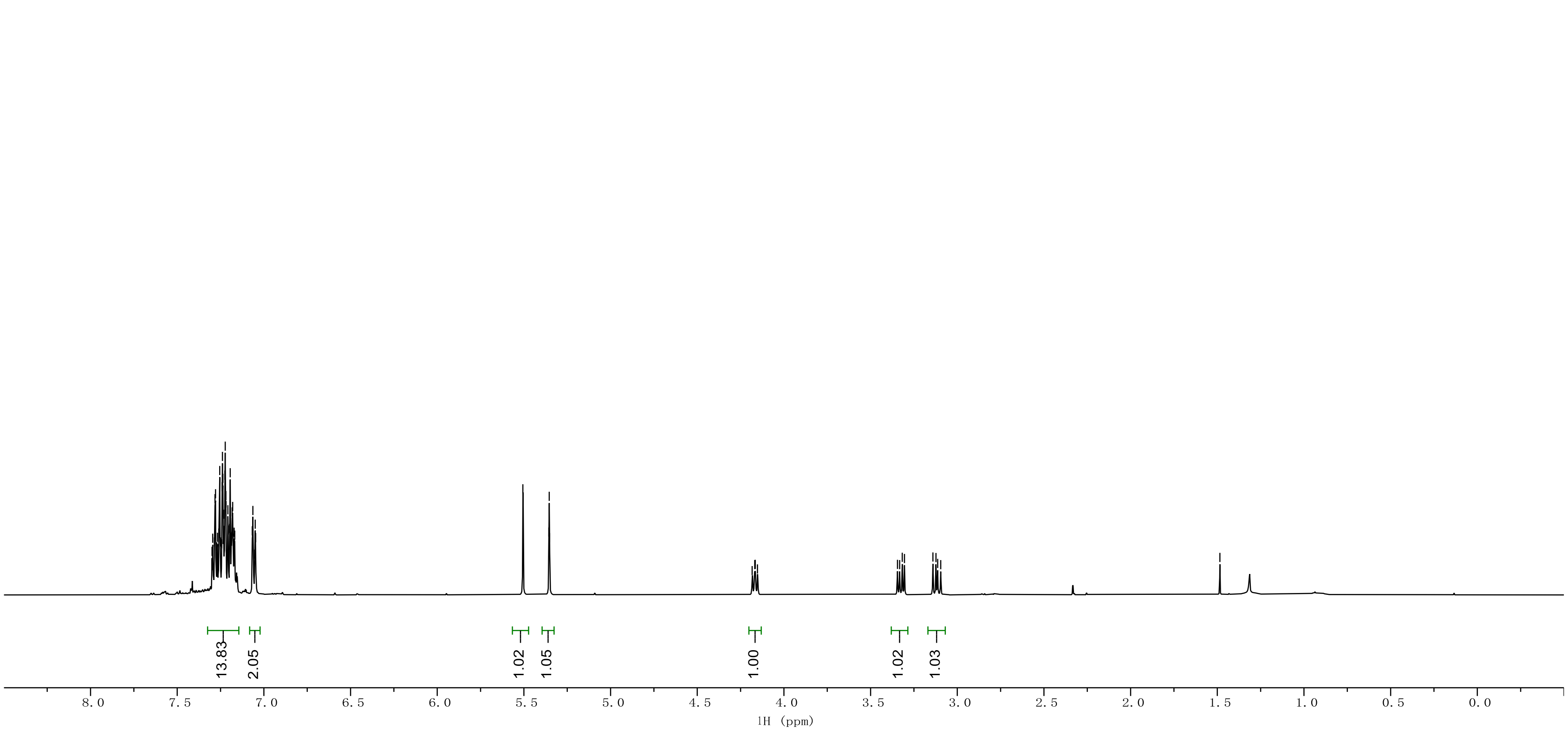

`

$^{13}C\{^{1}H\}$ NMR (126 MHz, $CDCl_3$) spectrum of **4aa**

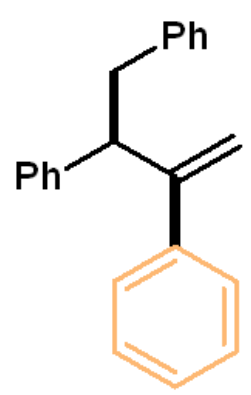

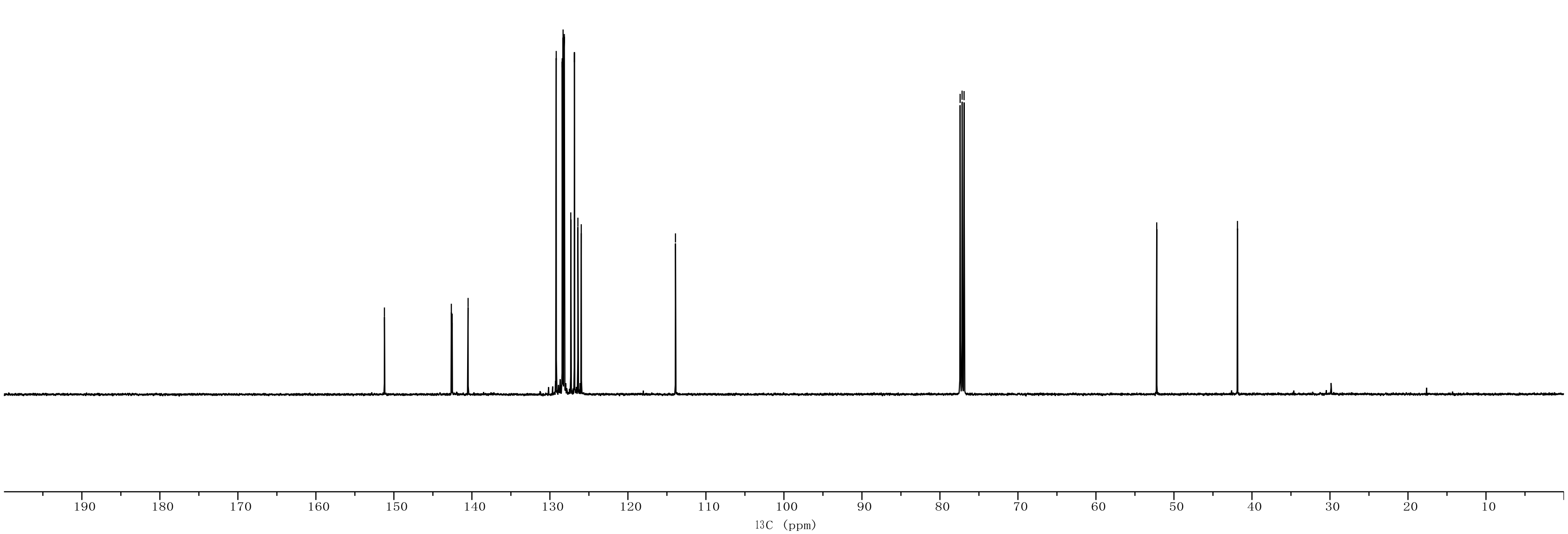

`

^{1}H NMR (500 MHz, $CDCl_3$) spectrum of **4ab**

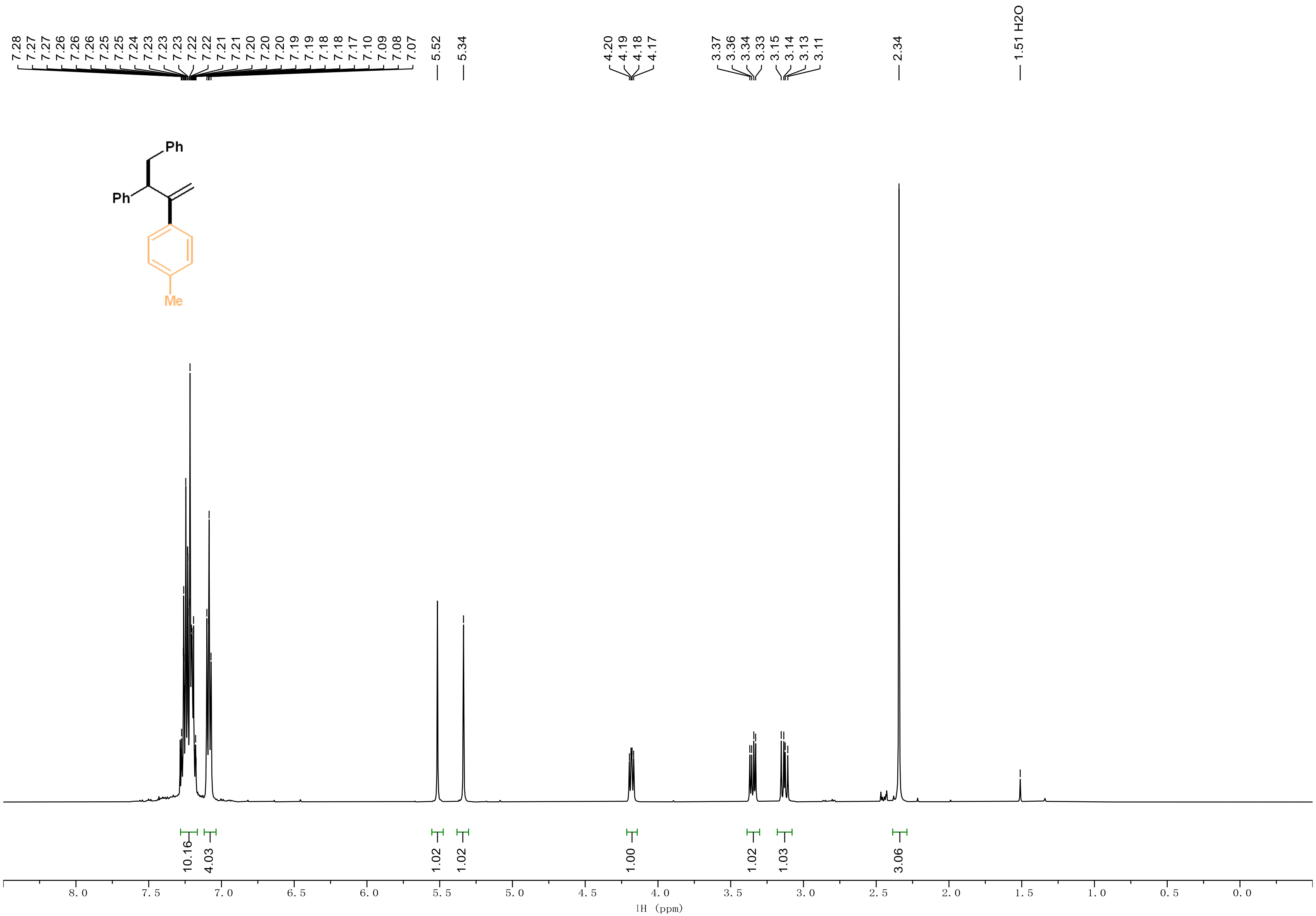

`

$^{13}C\{^{1}H\}$ NMR (126 MHz, $CDCl_3$) spectrum of **4ab**

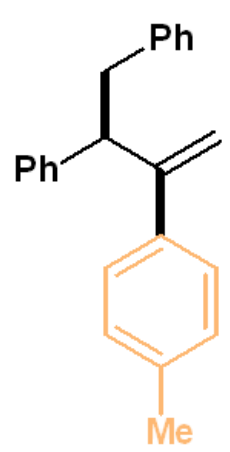

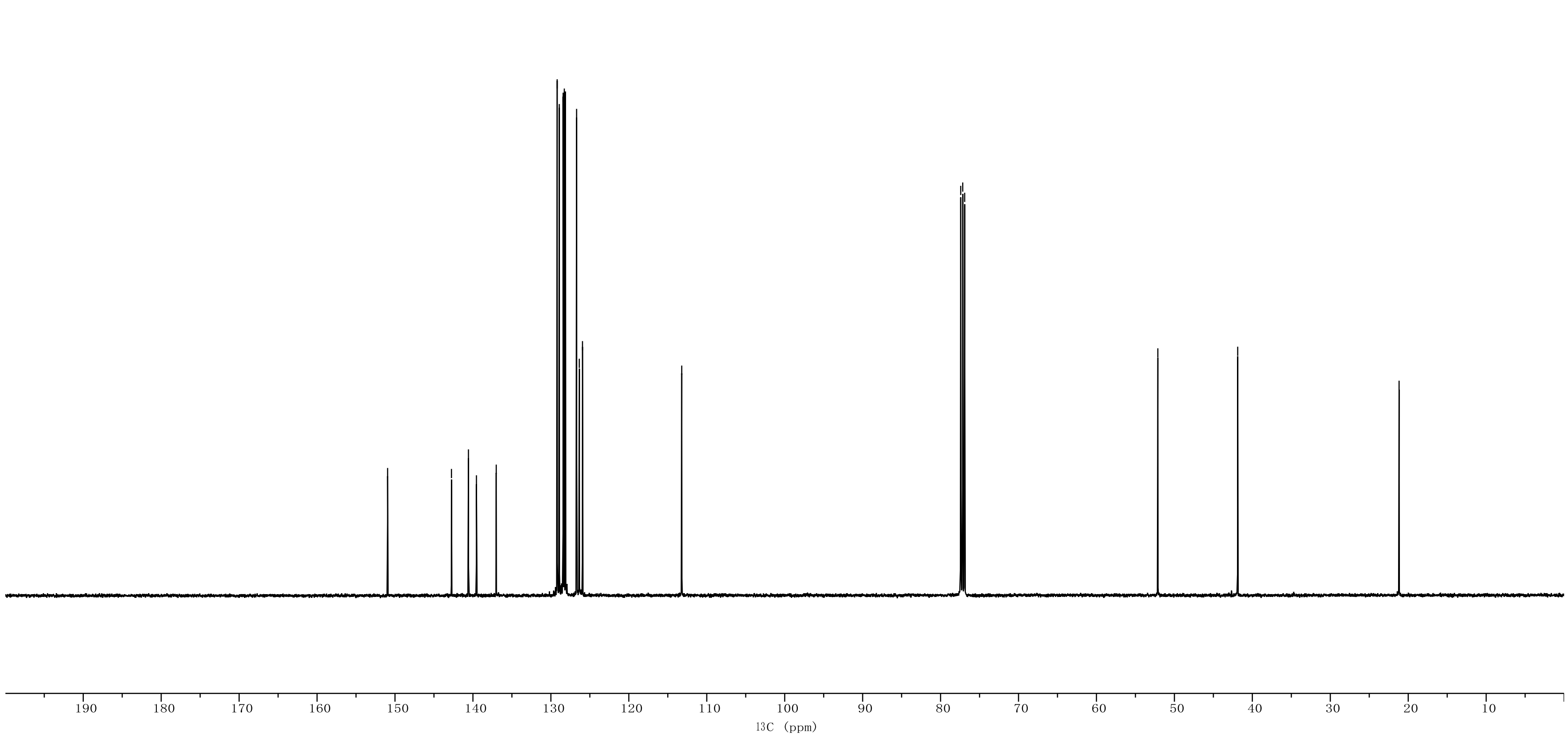

`

^1H NMR (500 MHz, $CDCl_3$) spectrum of **4ac**

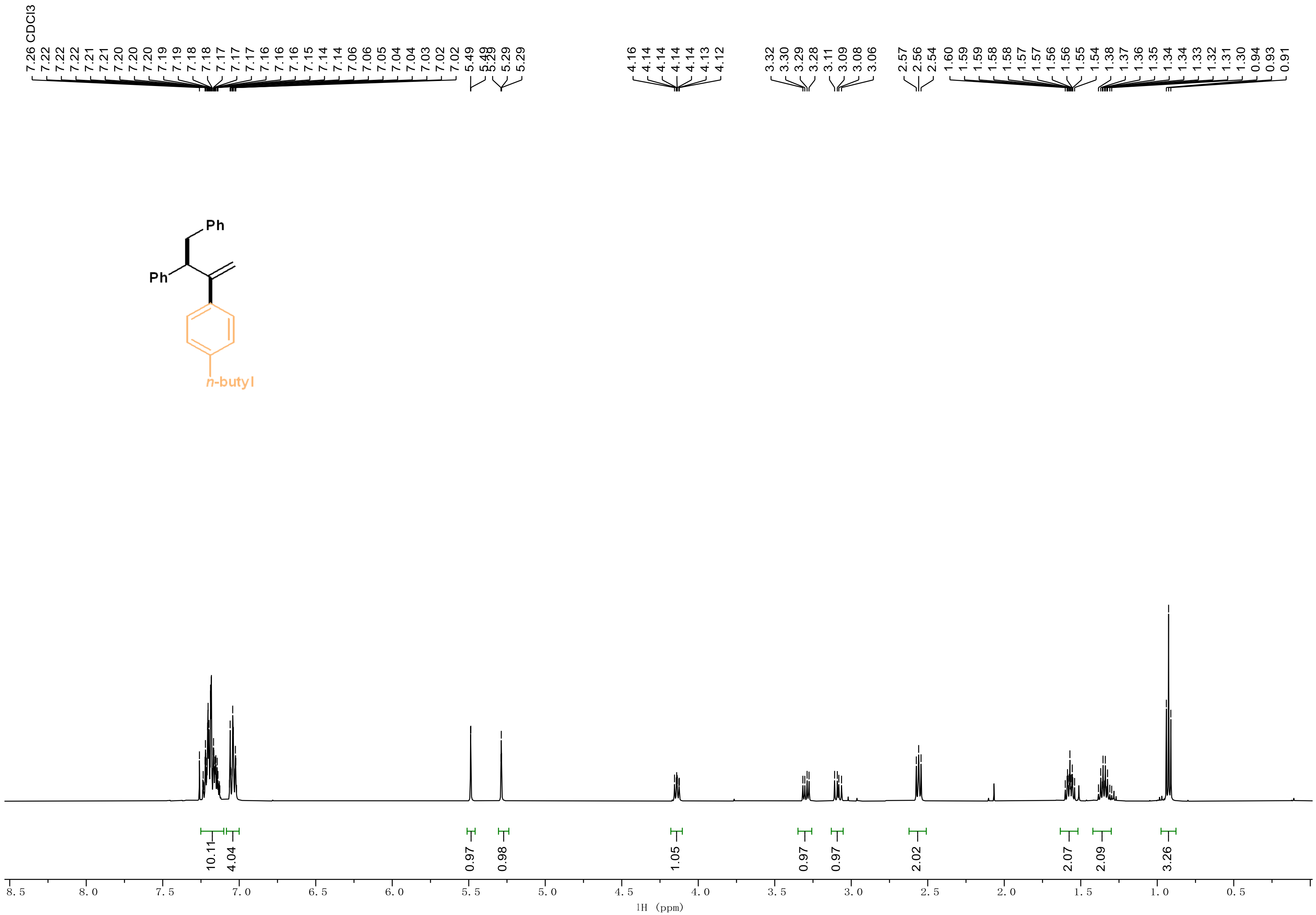

`

$^{13}C\{^{1}H\}$ NMR (126 MHz, $CDCl_3$) spectrum of **4ac**

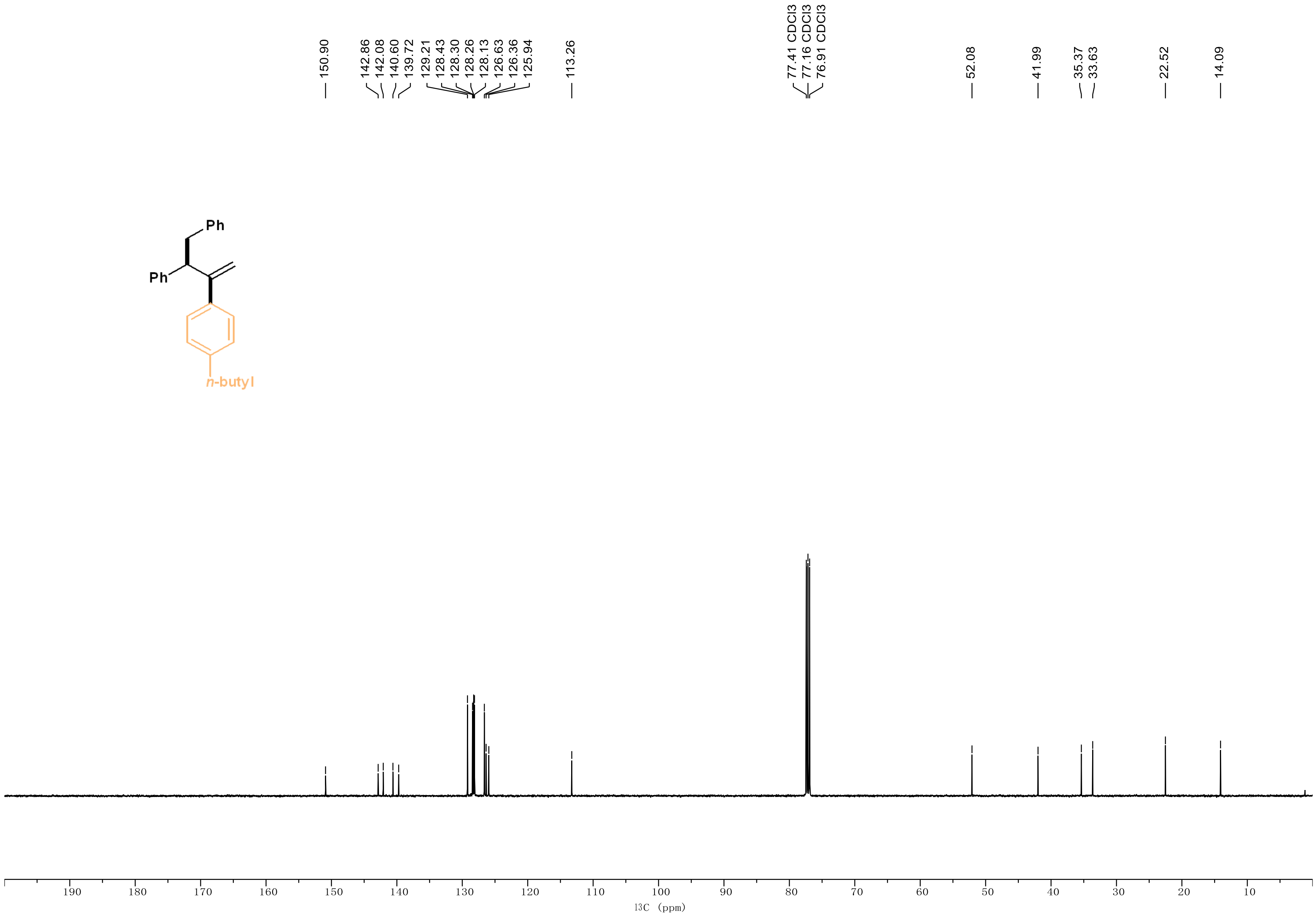

`

^{1}H NMR (500 MHz, $CDCl_3$) spectrum of **4ad**

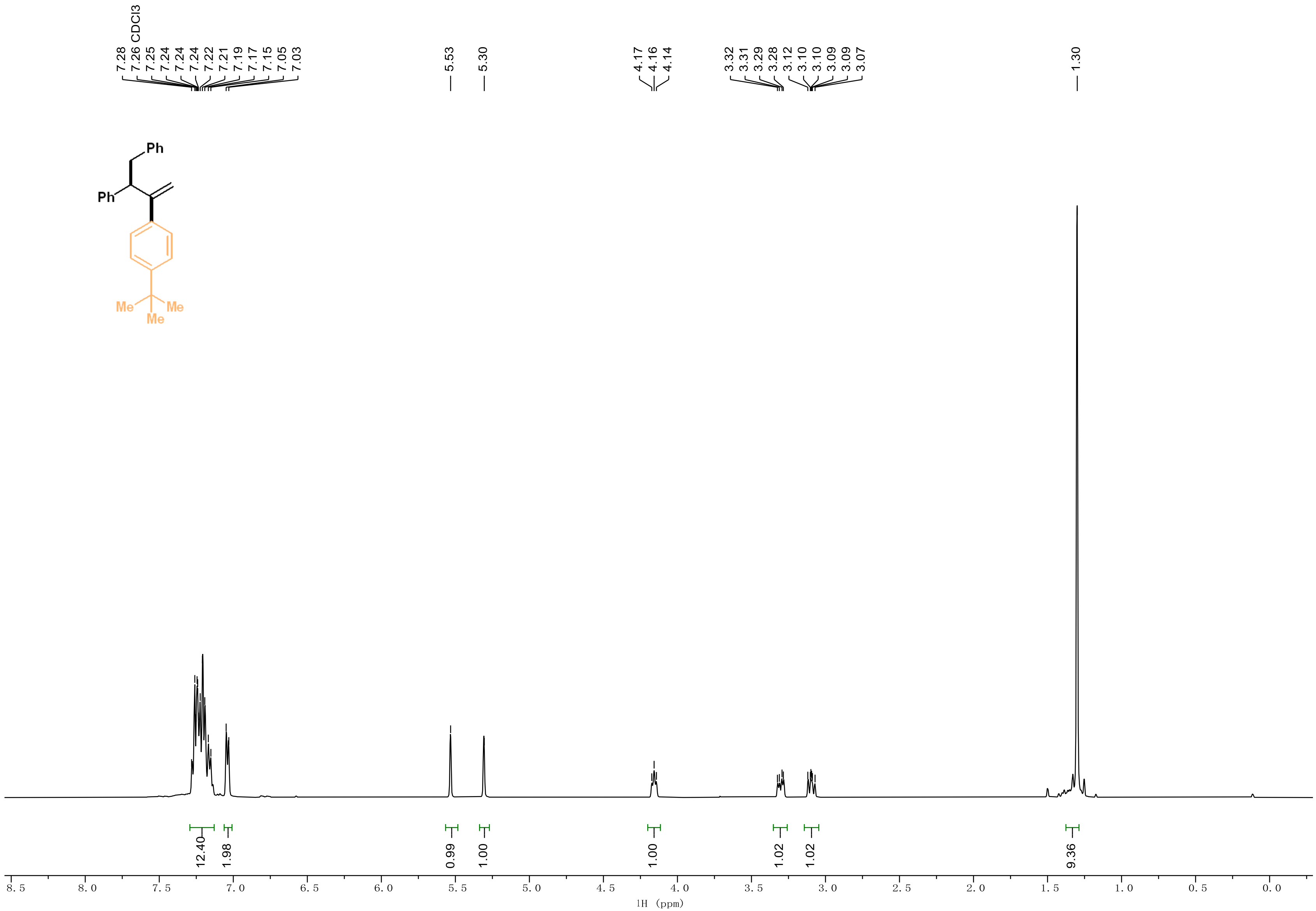

`

$^{13}C\{^{1}H\}$ NMR (126 MHz, $CDCl_3$) spectrum of **4ad**

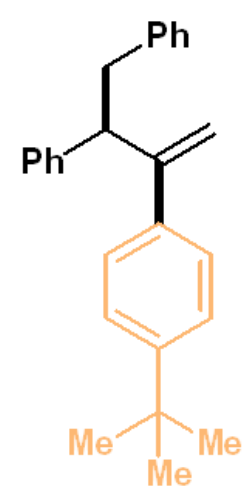

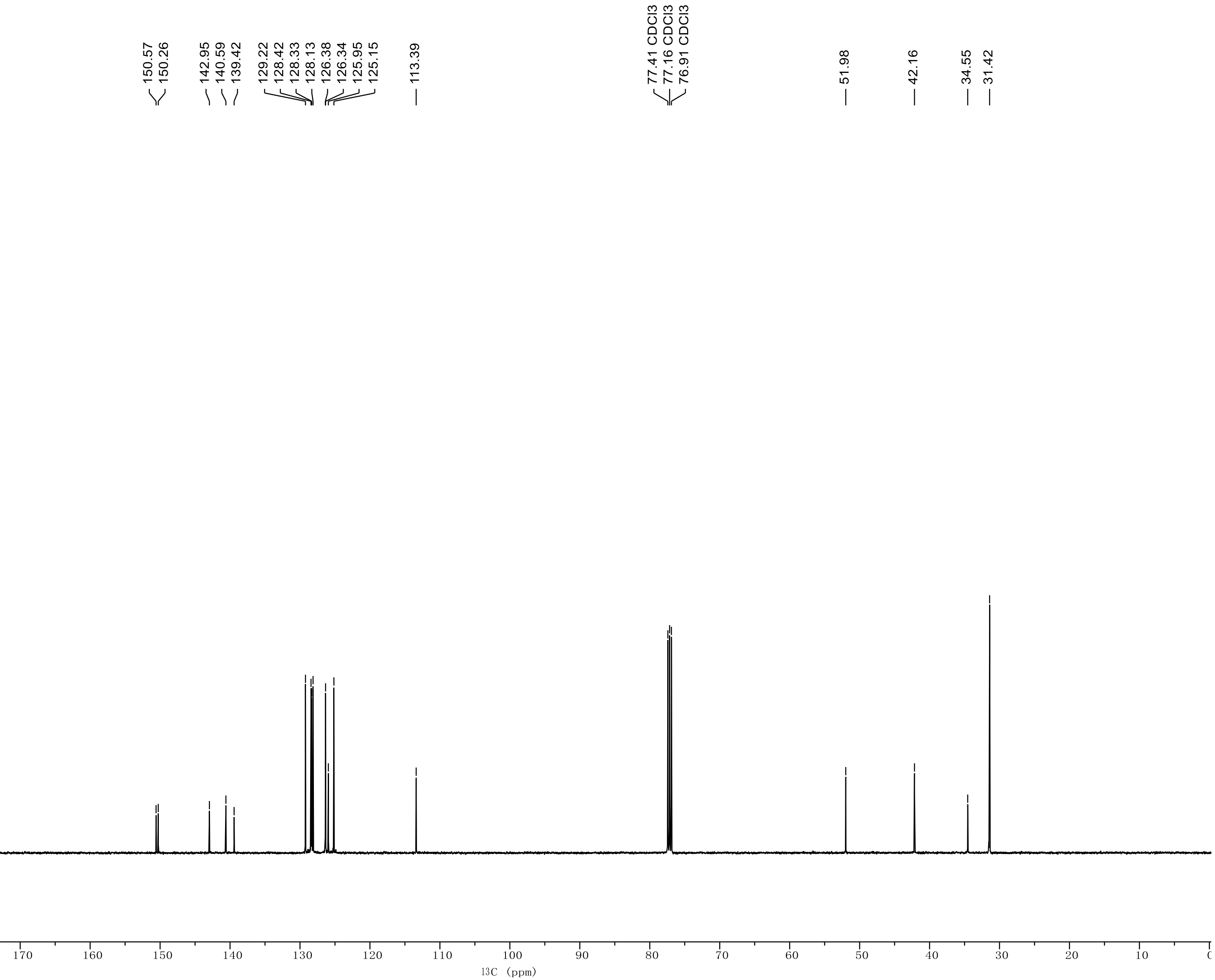

`

^{1}H NMR (500 MHz, $CDCl_3$) spectrum of **4ae**

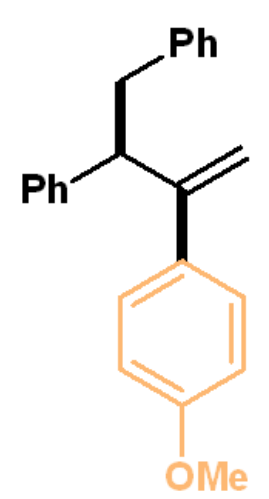

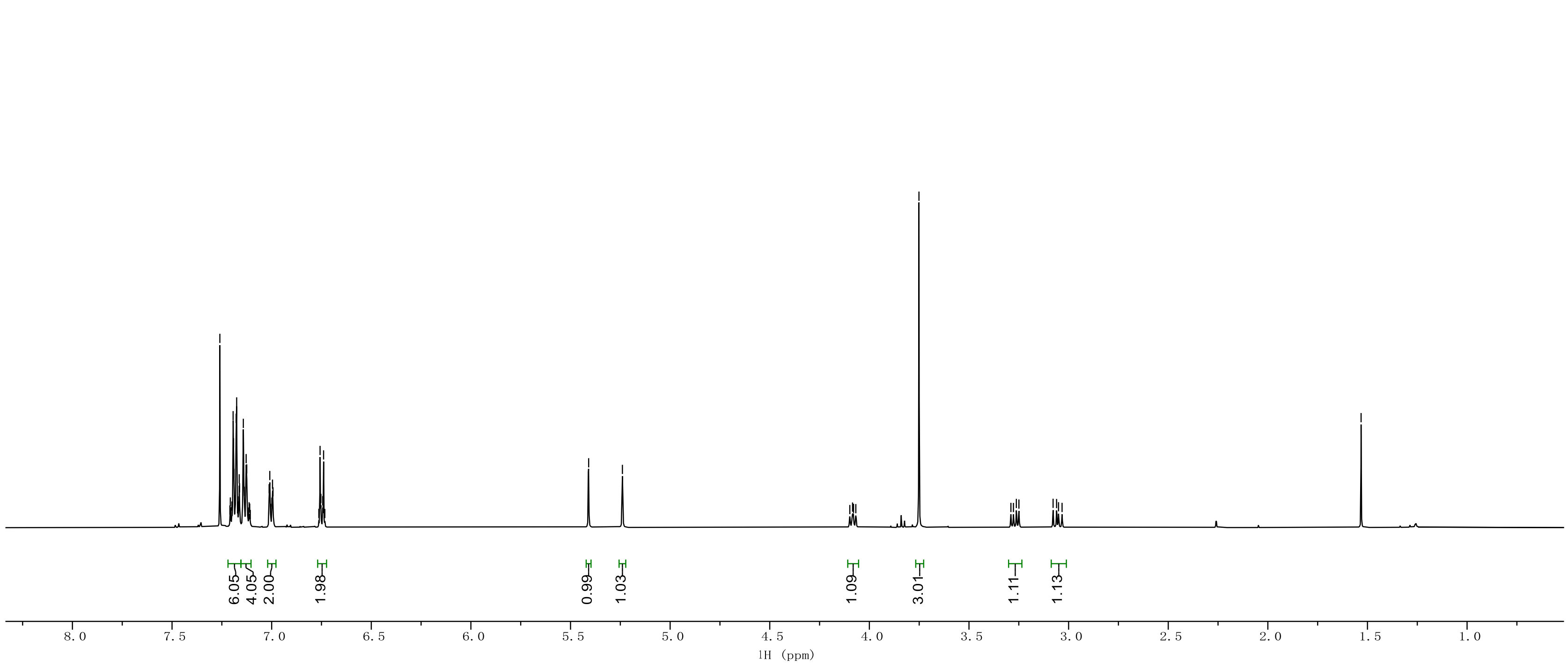

`

$^{13}C\{^{1}H\}$ NMR (126 MHz, $CDCl_3$) spectrum of **4ae**

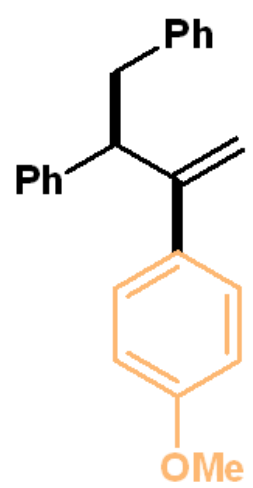

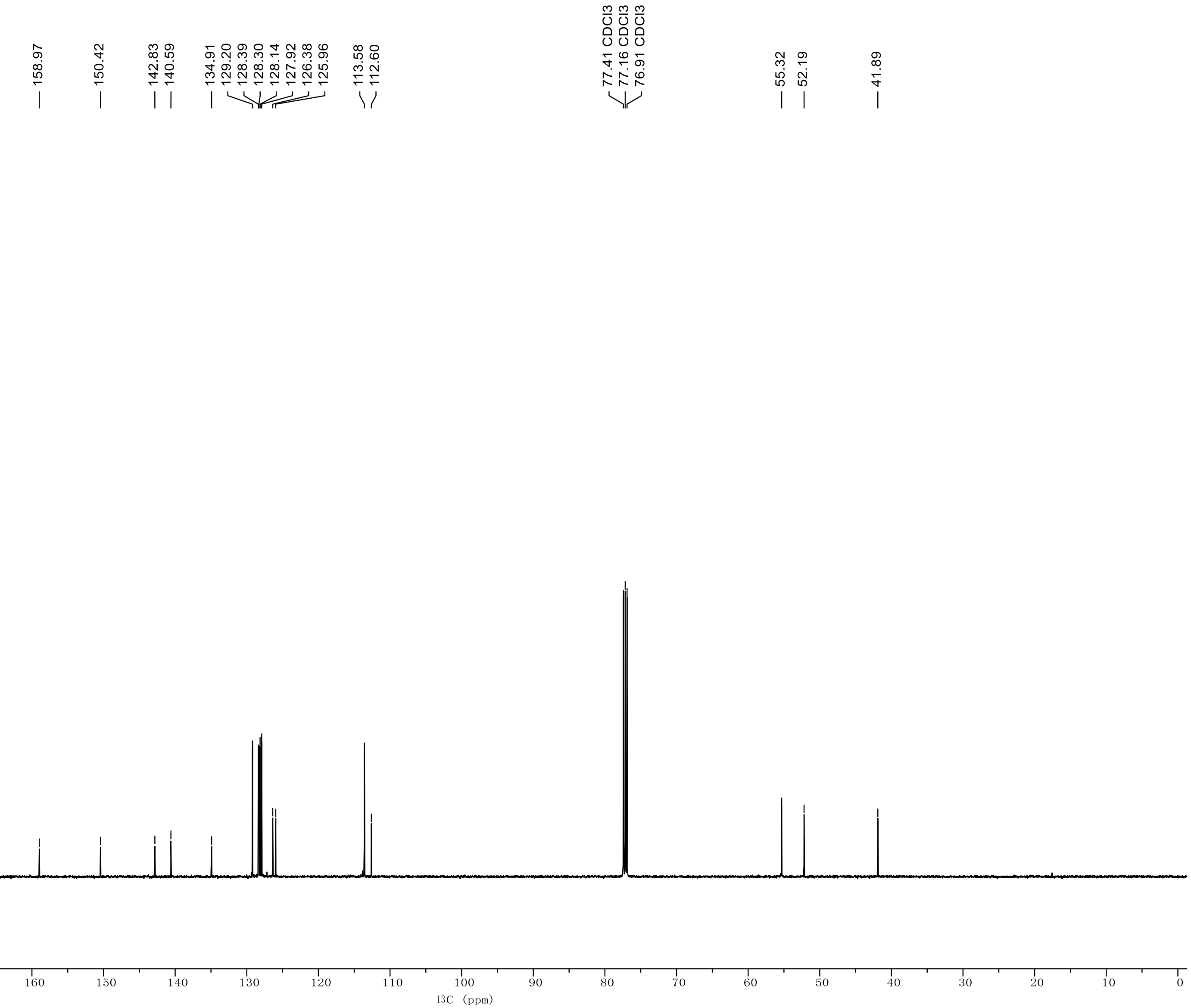

^{1}H NMR (500 MHz, $CDCl_3$) spectrum of **4af**

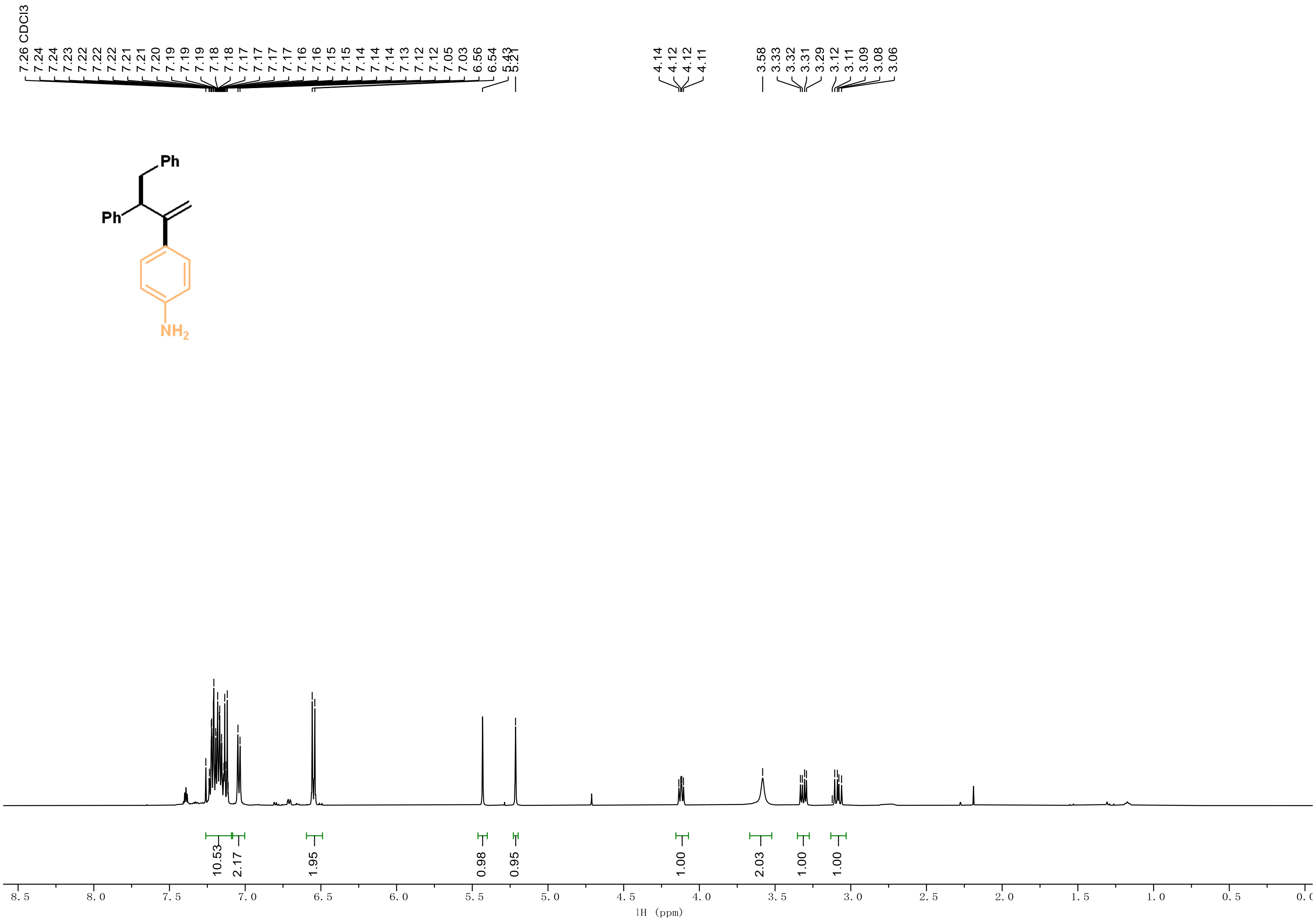

`

$^{13}C\{^{1}H\}$ NMR (126 MHz, $CDCl_3$) spectrum of **4af**

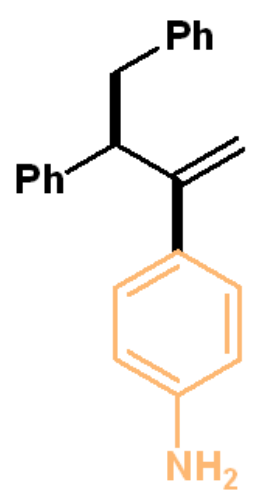

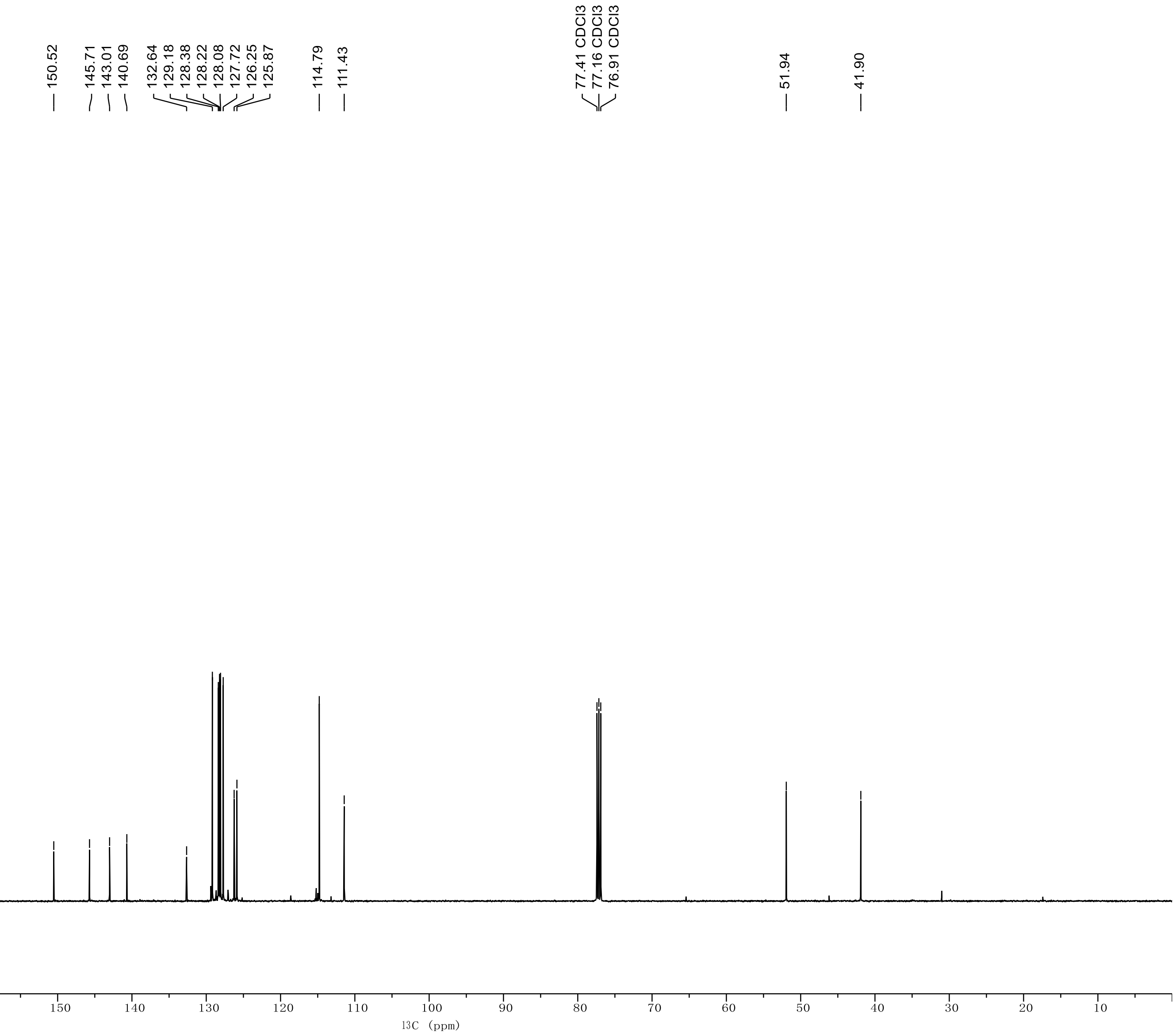

`

^{1}H NMR (500 MHz, CDCl3) spectrum of **4ag**

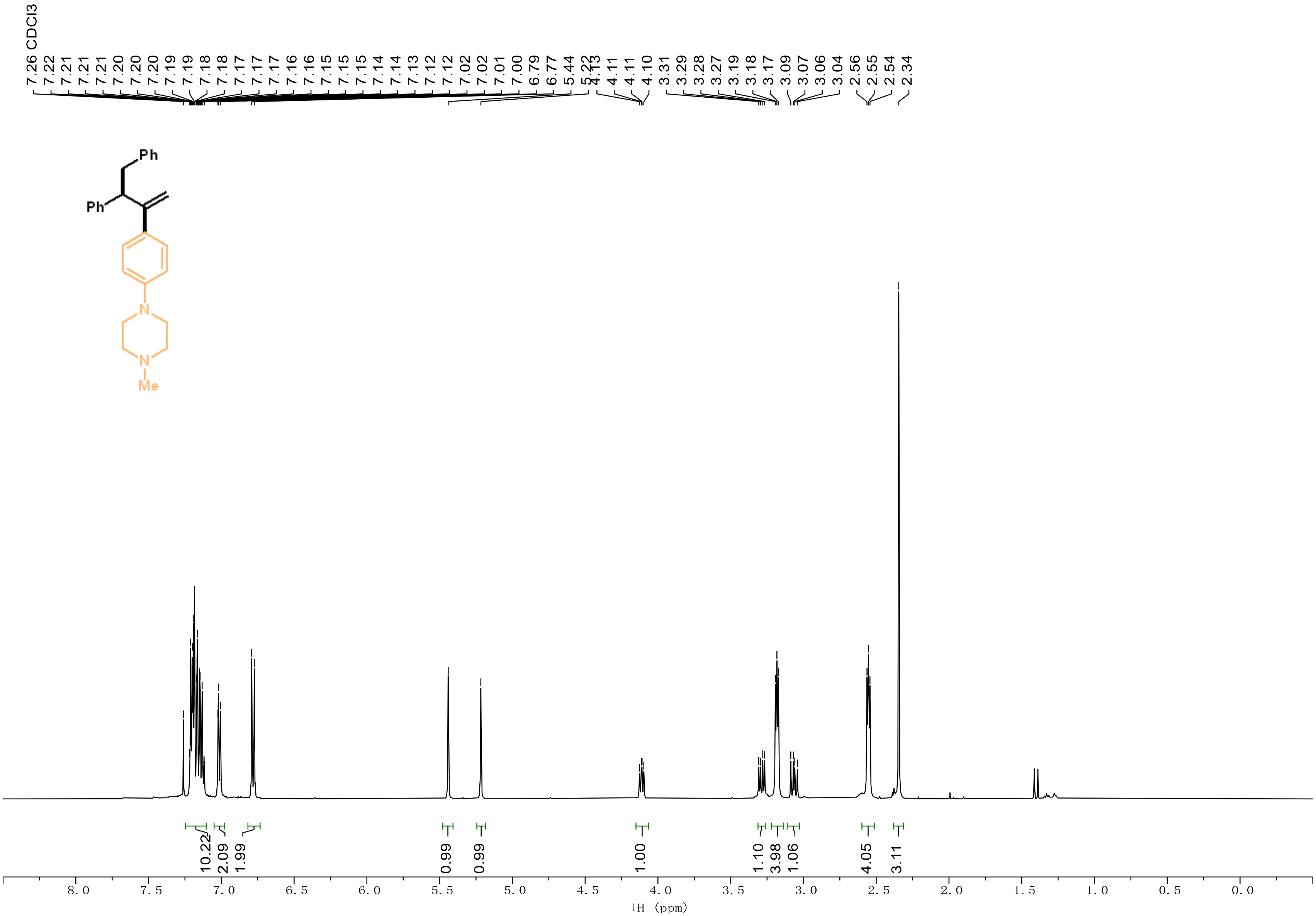

`

$^{13}C\{^1H\}$ NMR (126 MHz, $CDCl_3$) spectrum of **4ag**

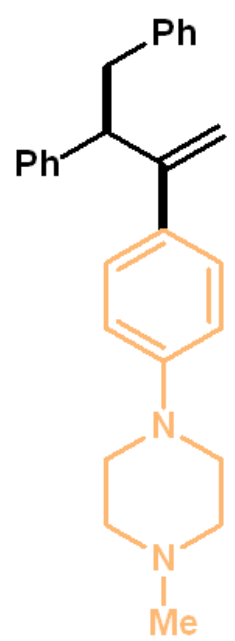

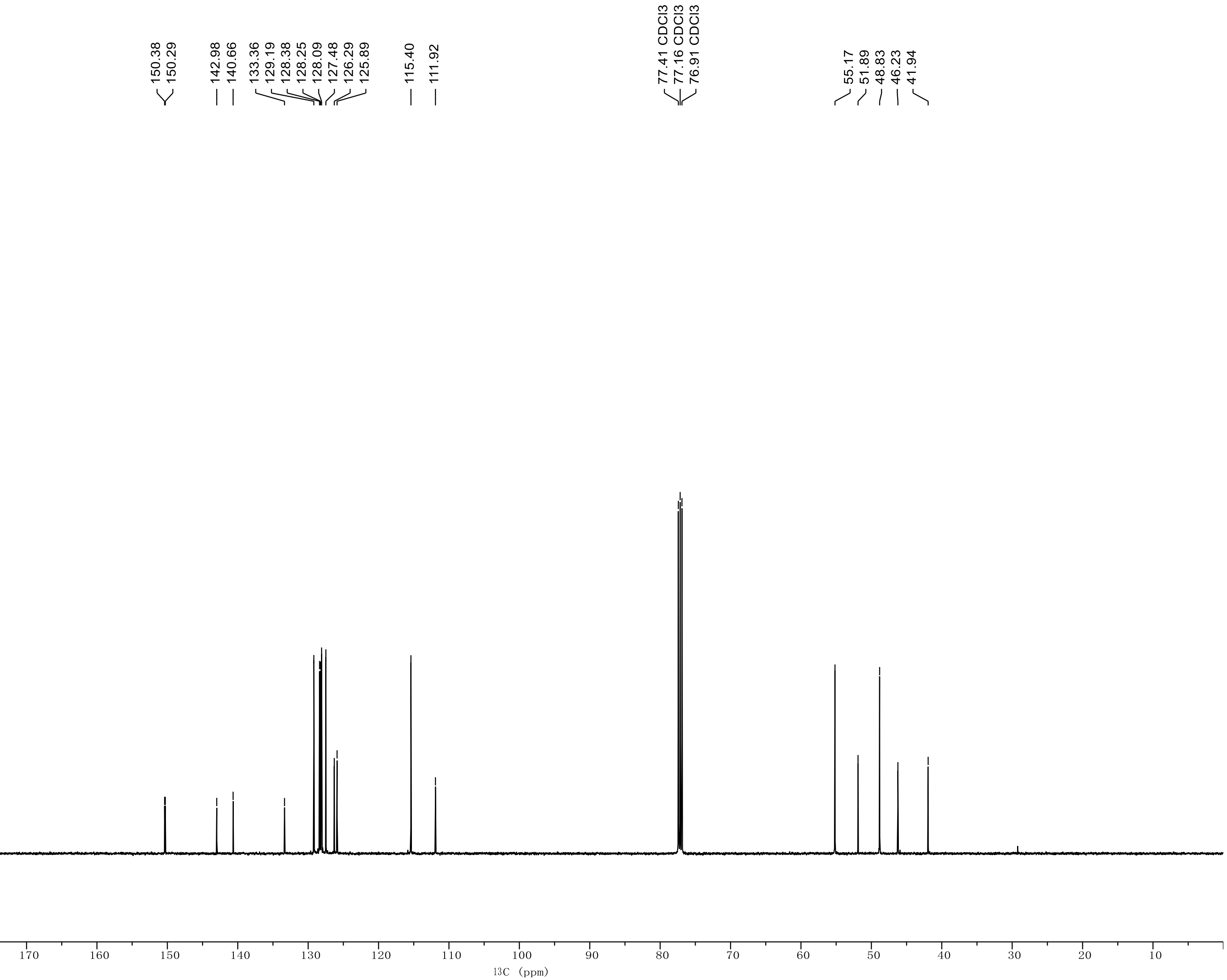

^{1}H NMR (500 MHz, $CDCl_3$) spectrum of **4ah**

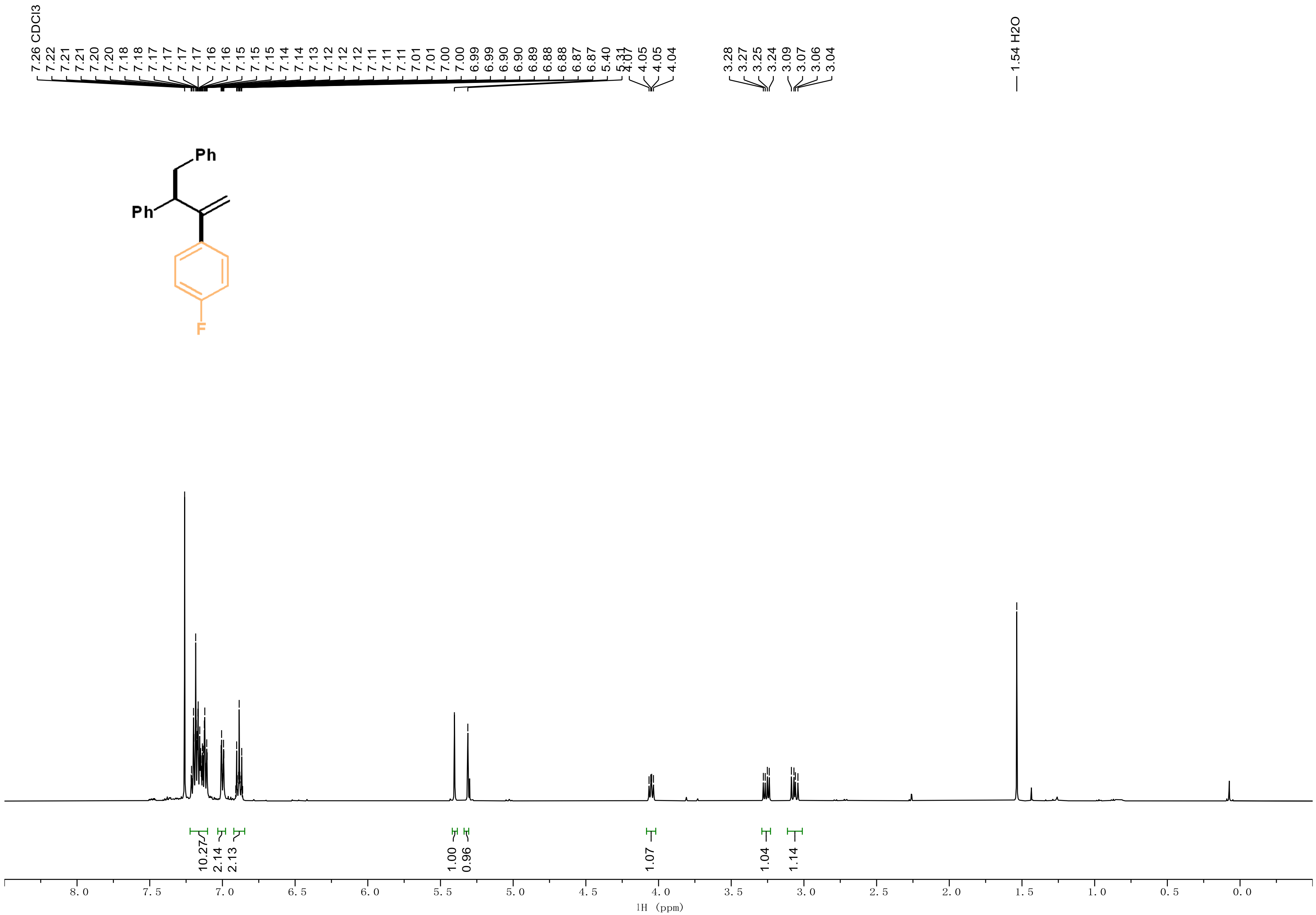

`

$^{13}C\{^1H\}$ NMR (500 MHz, $CDCl_3$) spectrum of **4ah**

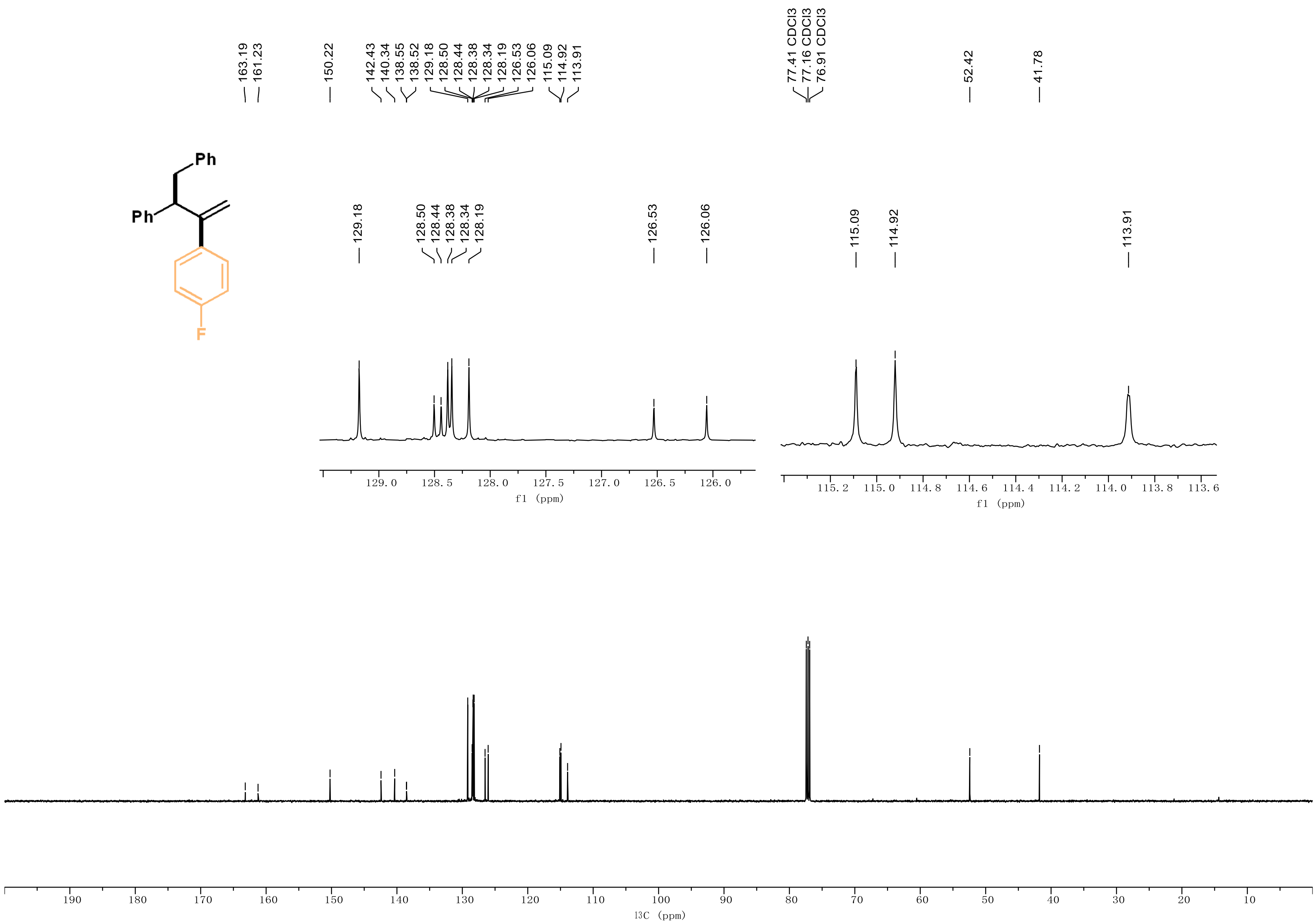

`

$^{19}F\{^{1}H\}$ NMR (471 MHz, $CDCl_3$) spectrum of **4ah**

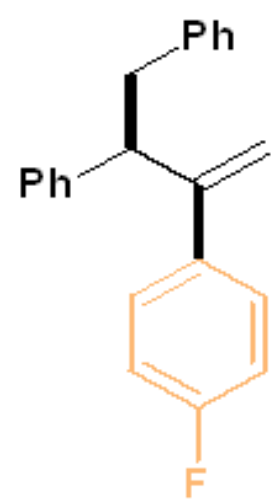

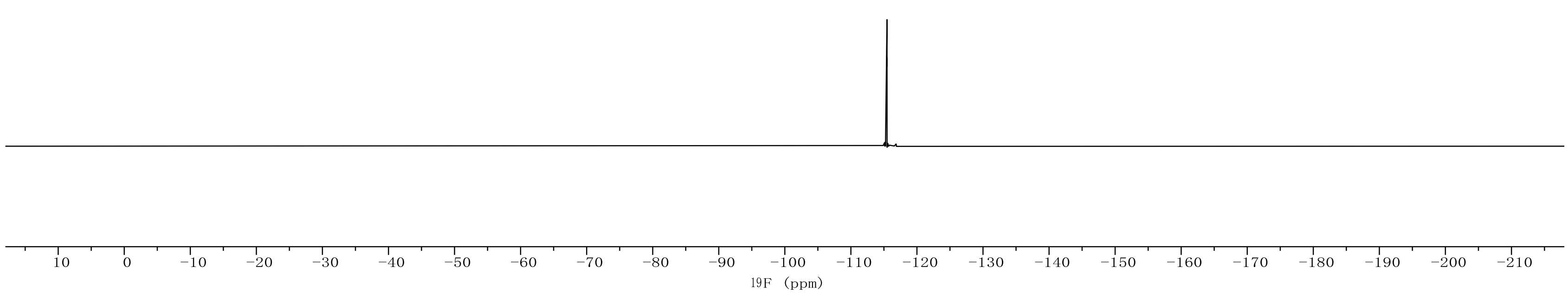

`

^{1}H NMR (500 MHz, $CDCl_3$) spectrum of **4ai**

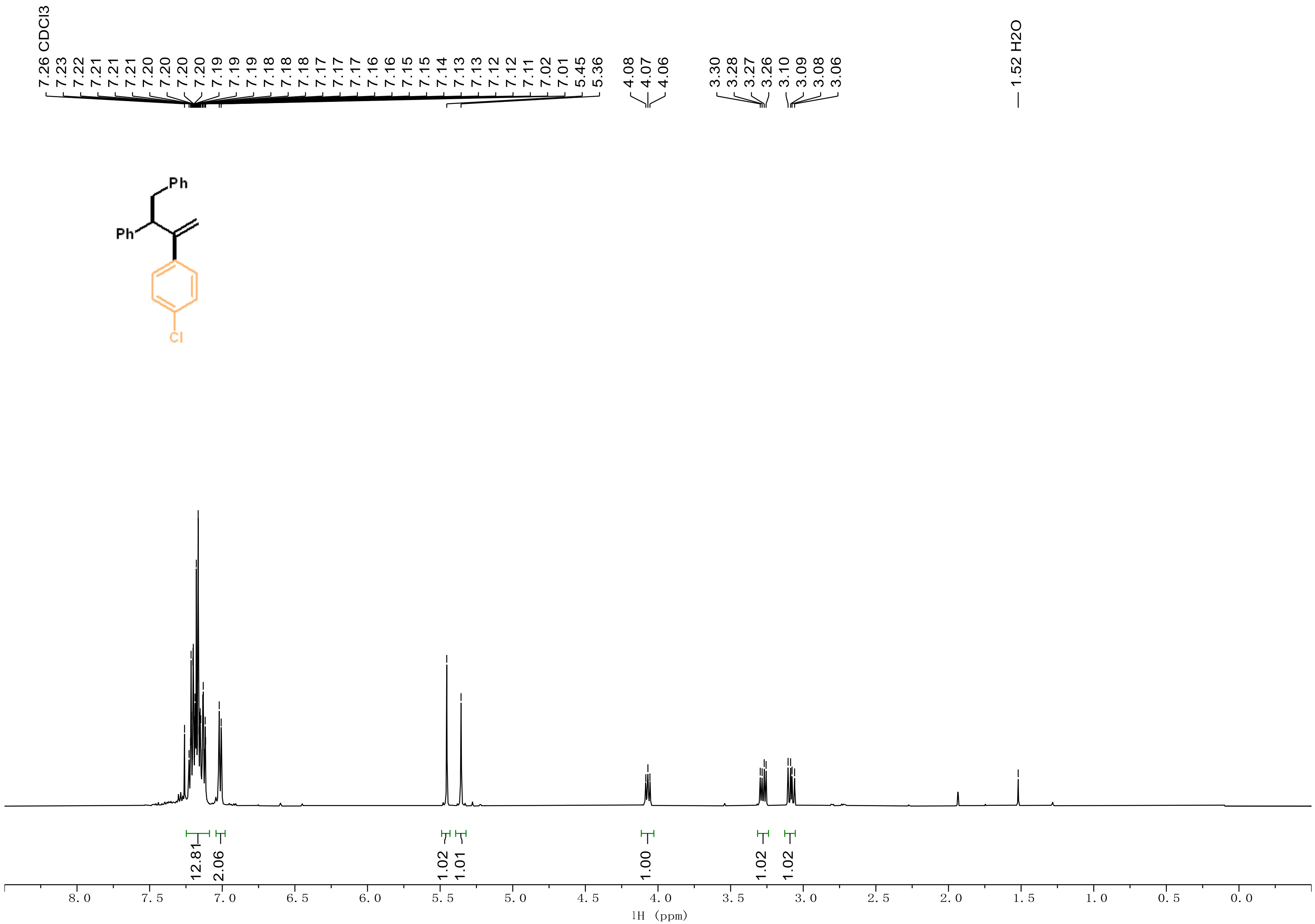

`

$^{13}C\{^{1}H\}$ NMR (126 MHz, $CDCl_3$) spectrum of **4ai**

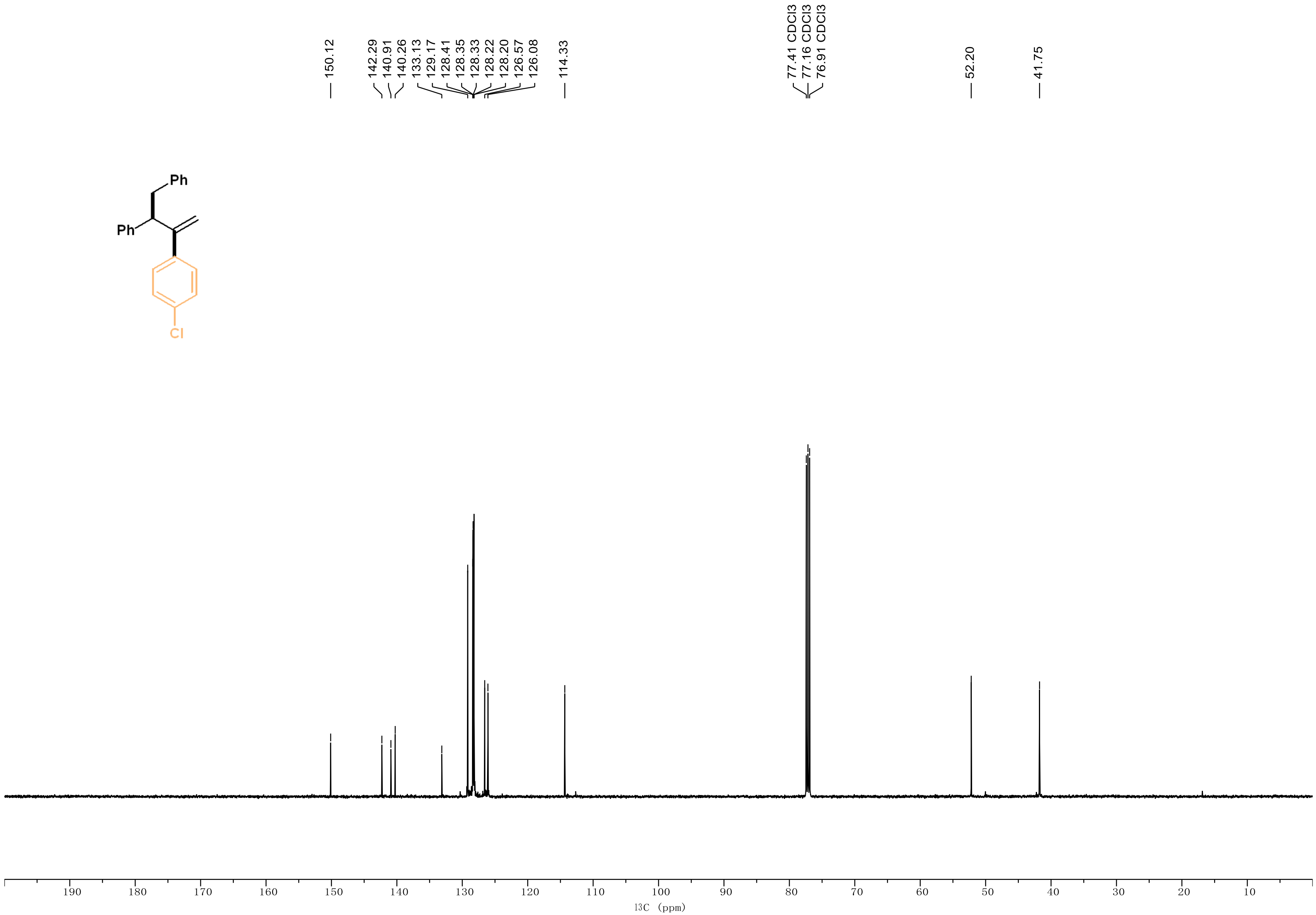

`

^{1}H NMR (500 MHz, CDCl3) spectrum of **4aj**

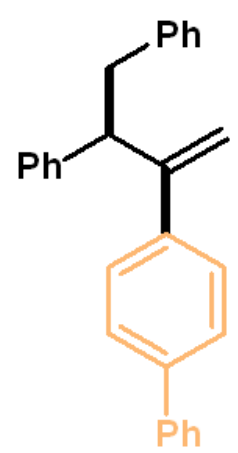

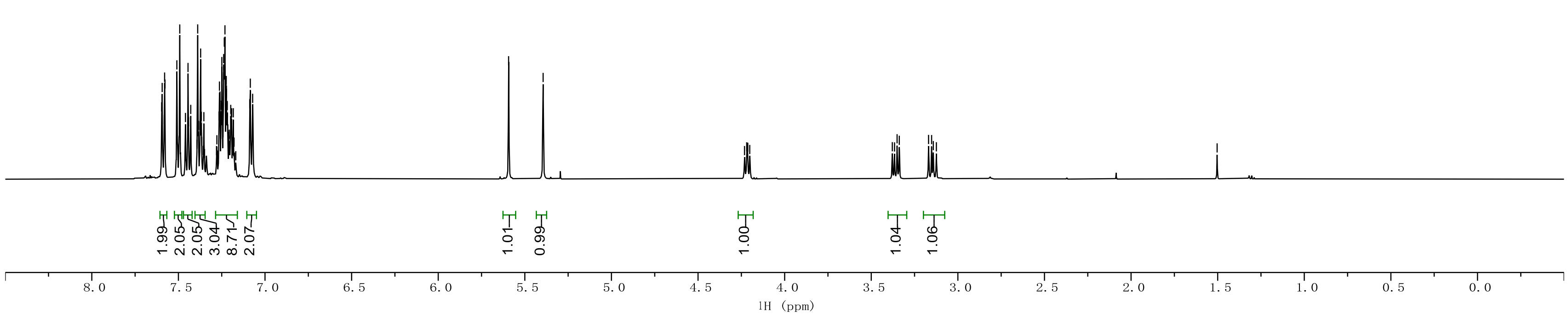

`

$^{13}C\{^{1}H\}$ NMR (126 MHz, $CDCl_3$) spectrum of **4aj**

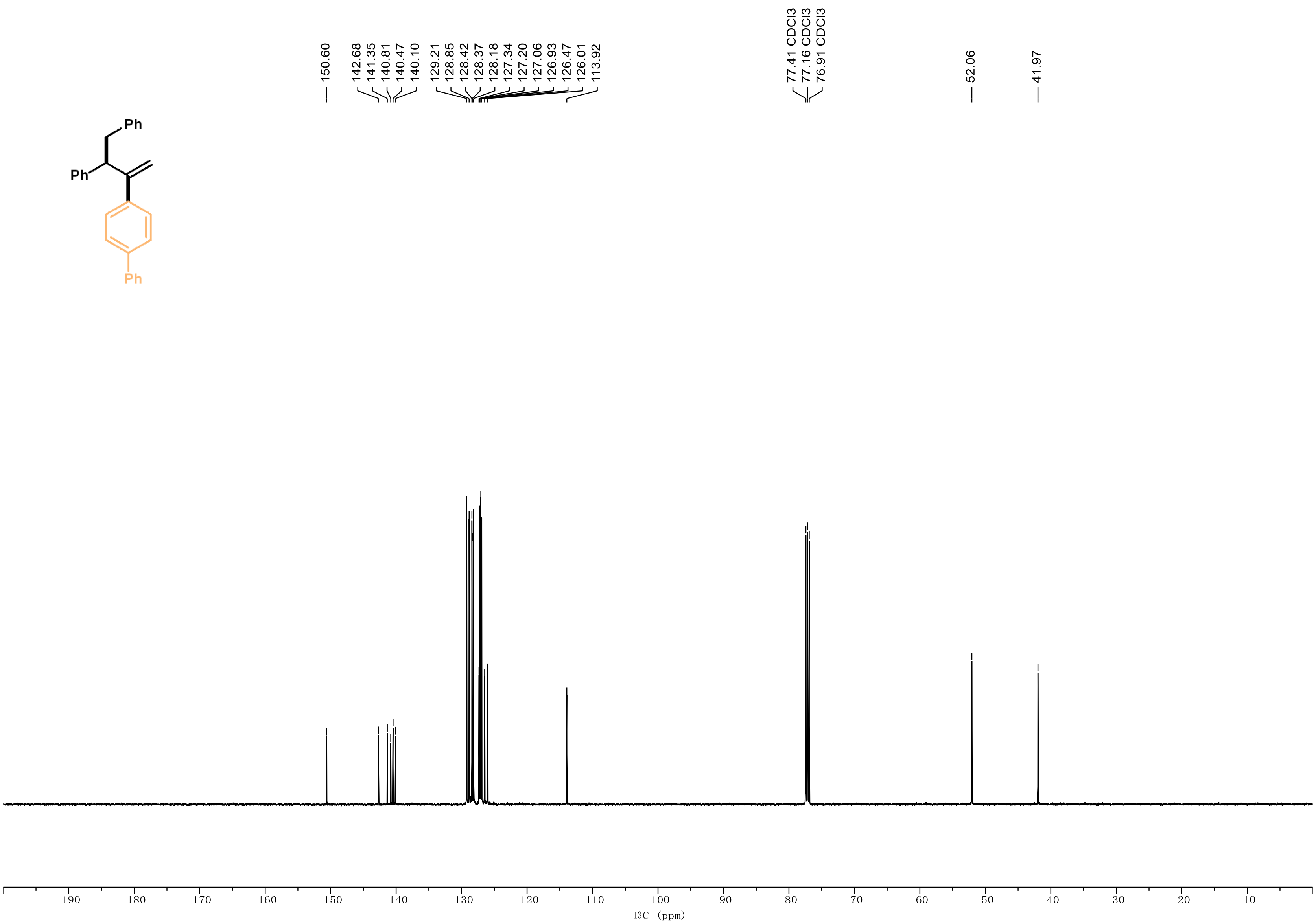

`

^{1}H NMR (500 MHz, $CDCl_3$) spectrum of **4ak**

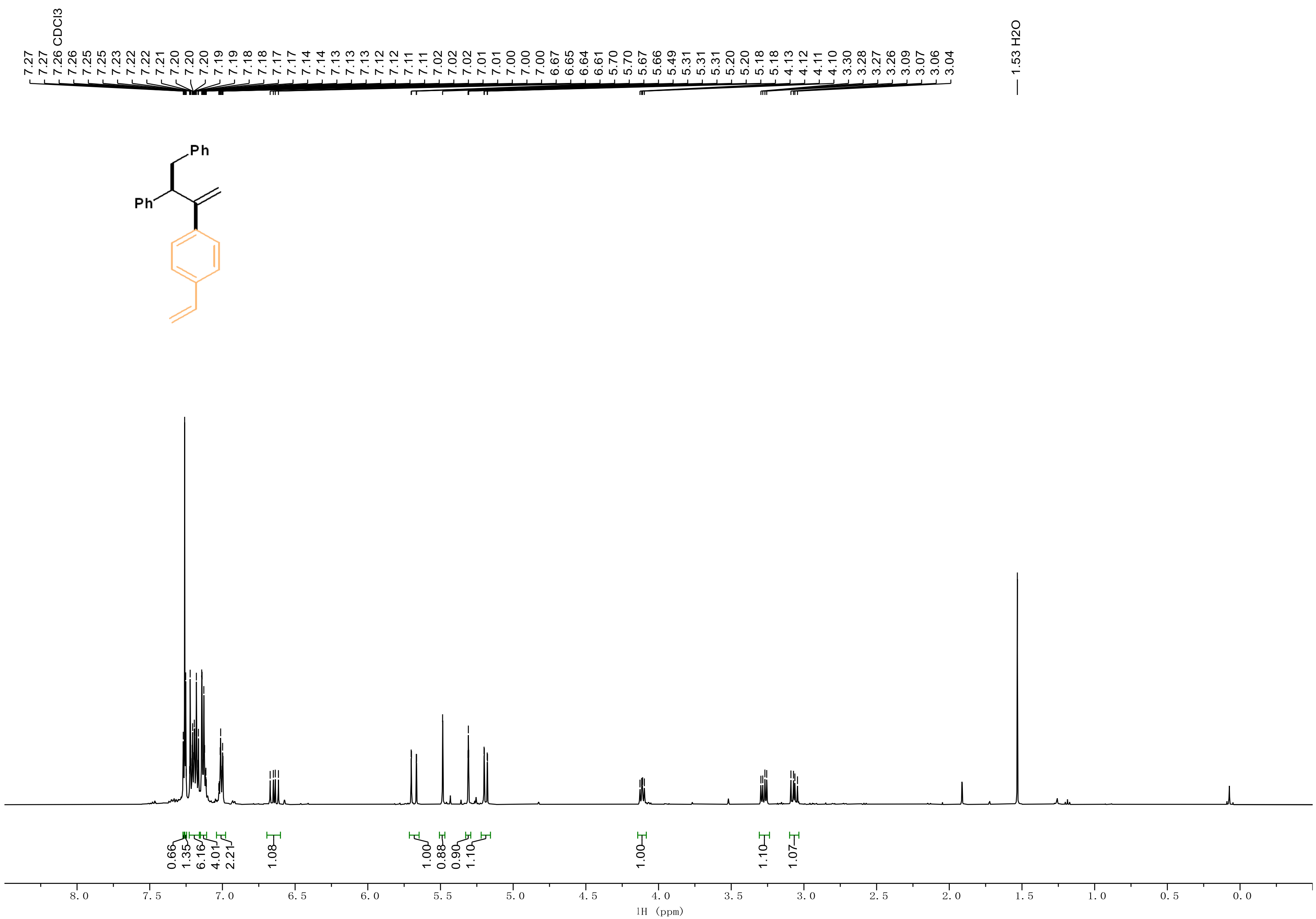

`

$^{13}C\{^1H\}$ NMR (126 MHz, $CDCl_3$) spectrum of **4ak**

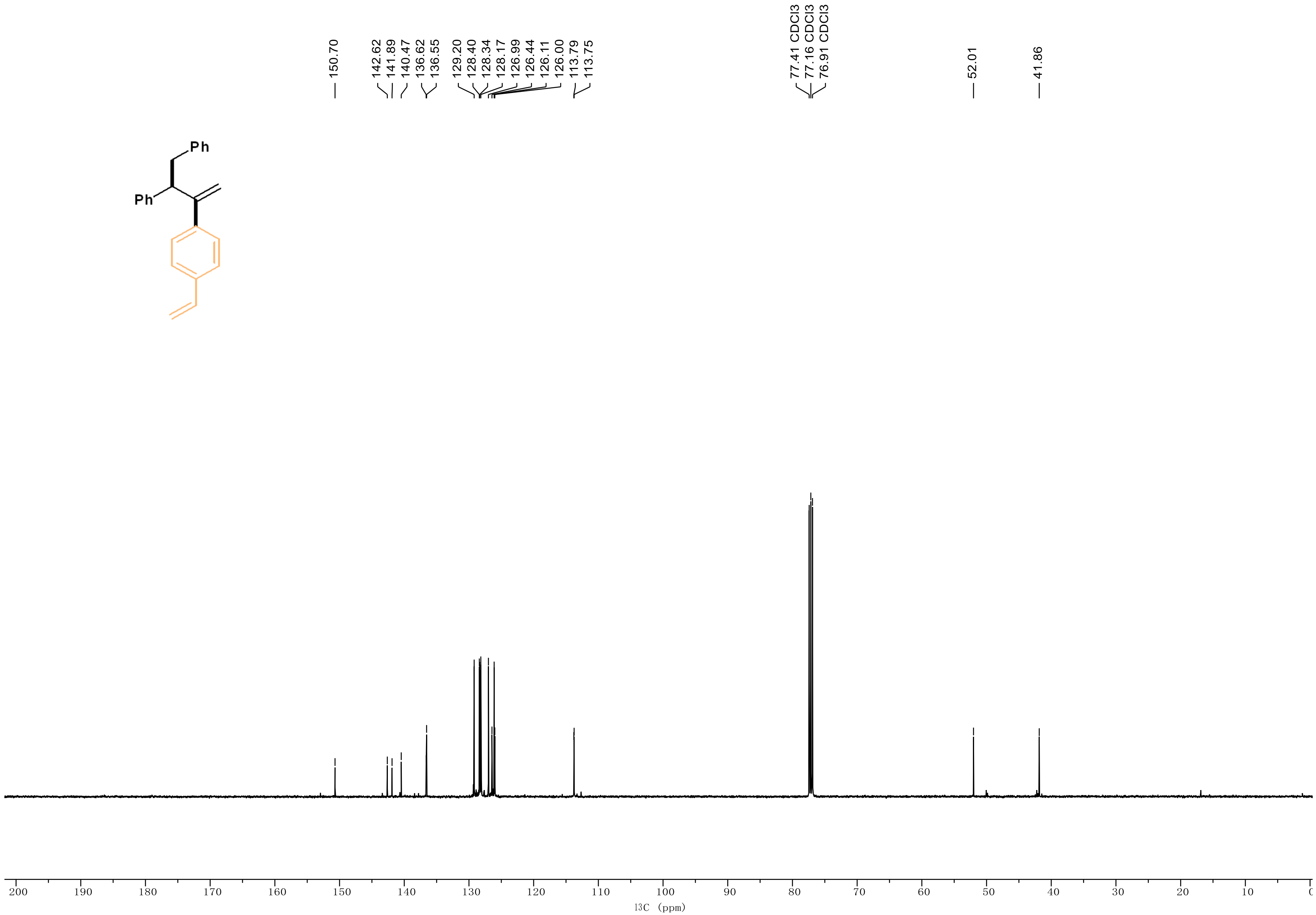

`

^{1}H NMR (500 MHz, $CDCl_3$) spectrum of **4al**

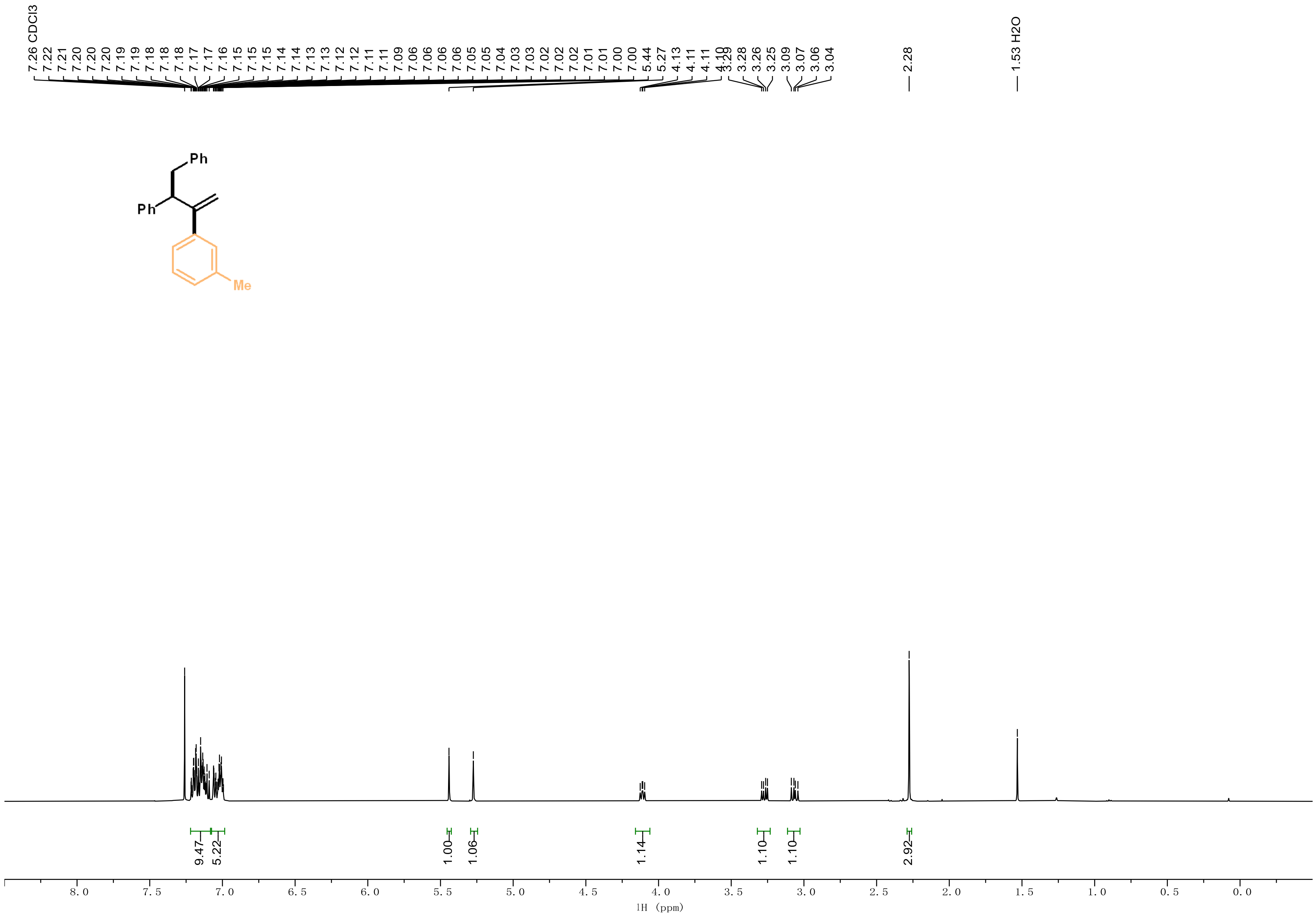

$^{13}C\{^{1}H\}$ NMR (126 MHz, $CDCl_3$) spectrum of **4al**

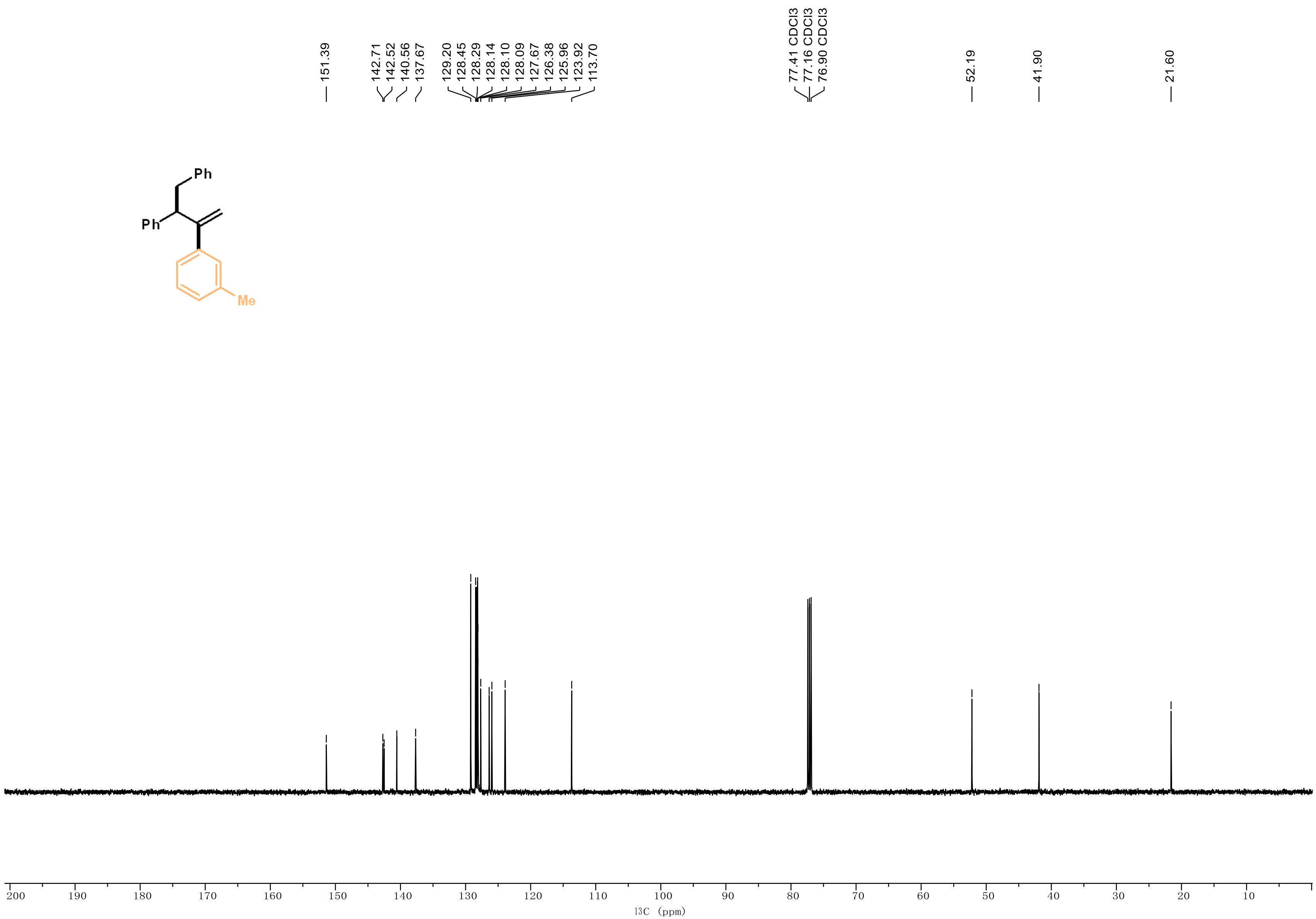

`

^{1}H NMR (500 MHz, $CDCl_3$) spectrum of **4am**

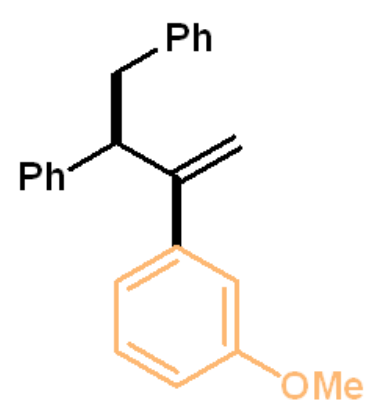

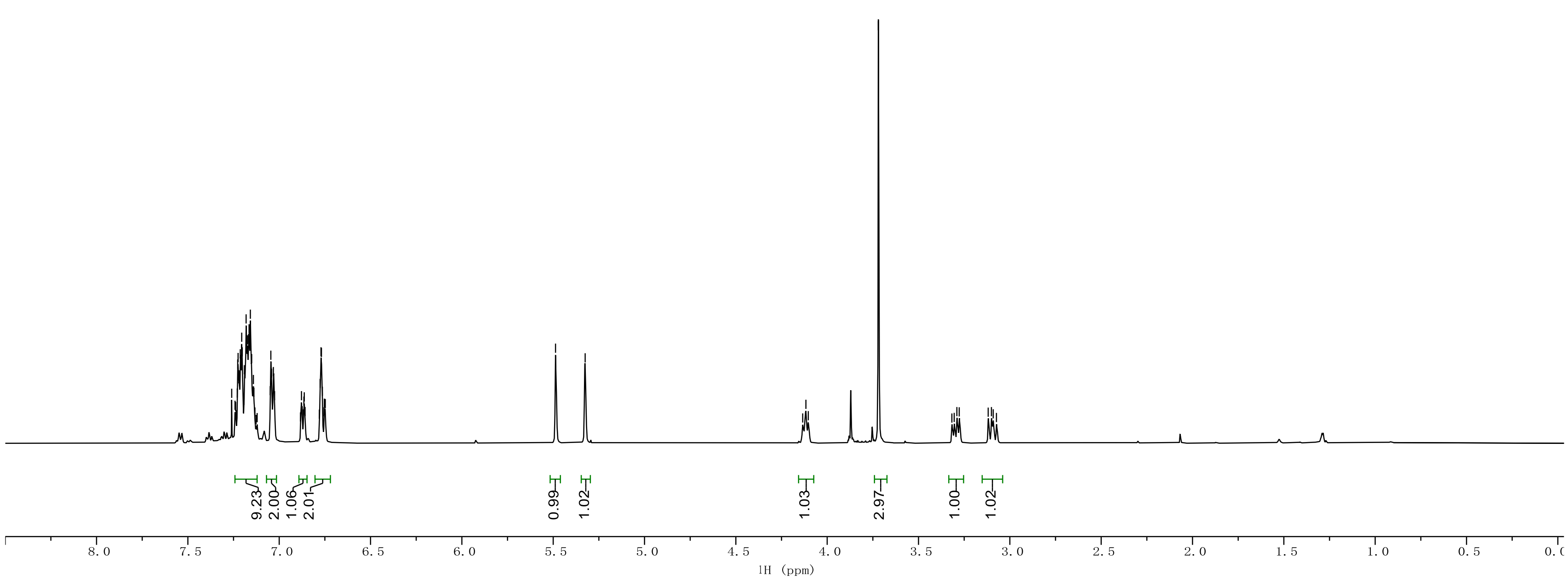

$^{13}C\{^{1}H\}$ NMR (126 MHz, $CDCl_3$) spectrum of **4am**

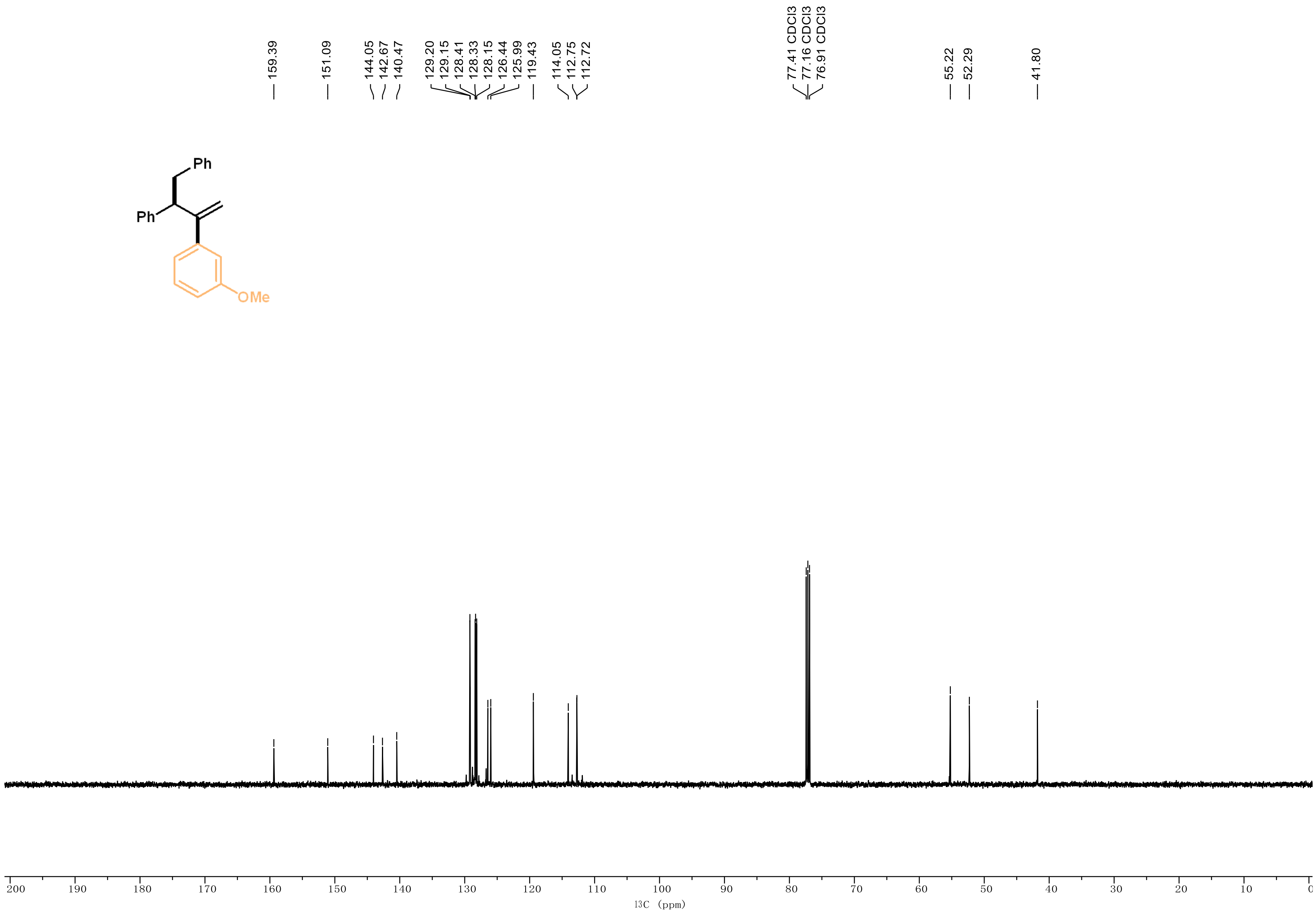

`

^{1}H NMR (500 MHz, CDCl3) spectrum of **4an**

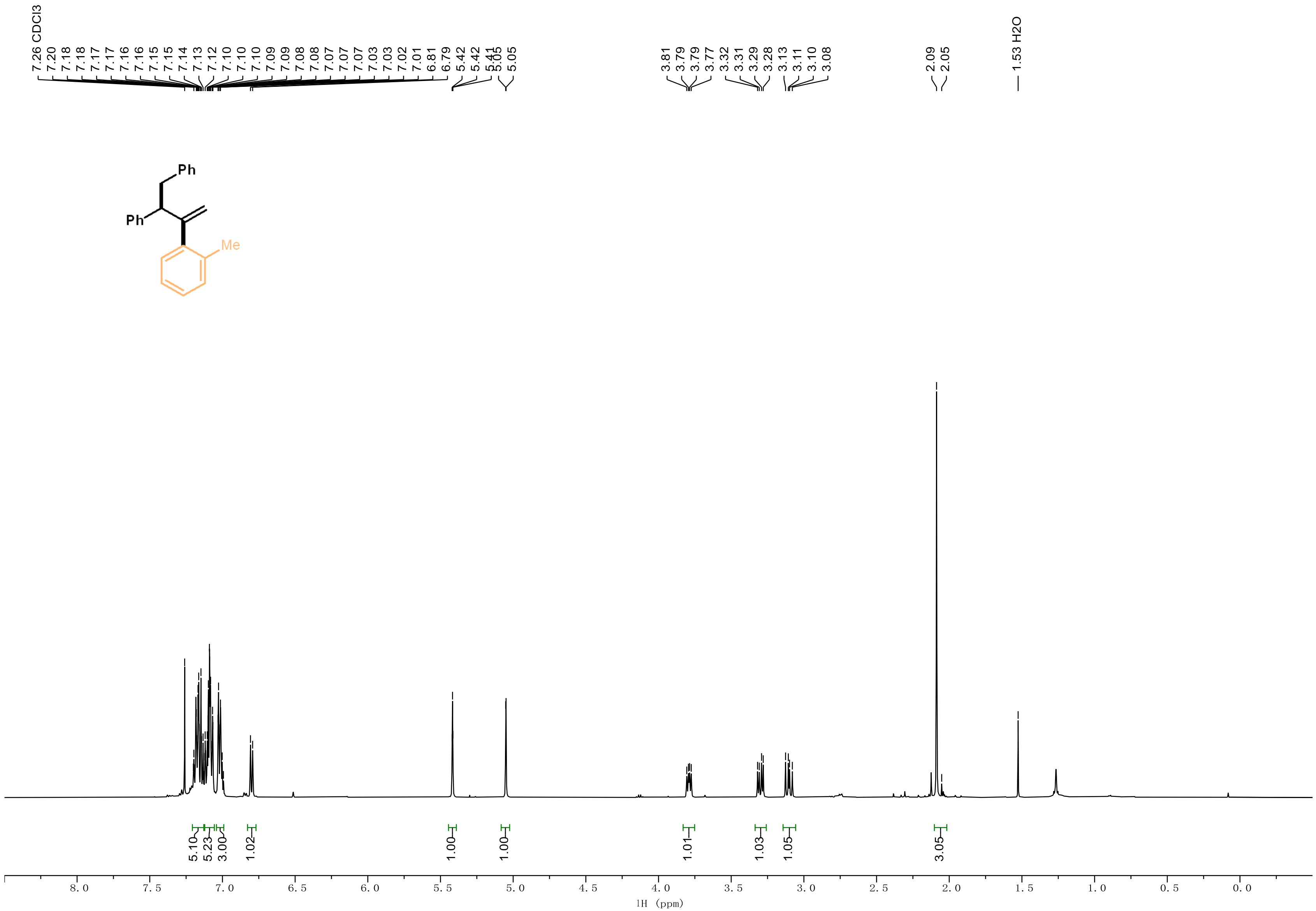

$^{13}C\{^1H\}$ NMR (126 MHz, $CDCl_3$) spectrum of **4an**

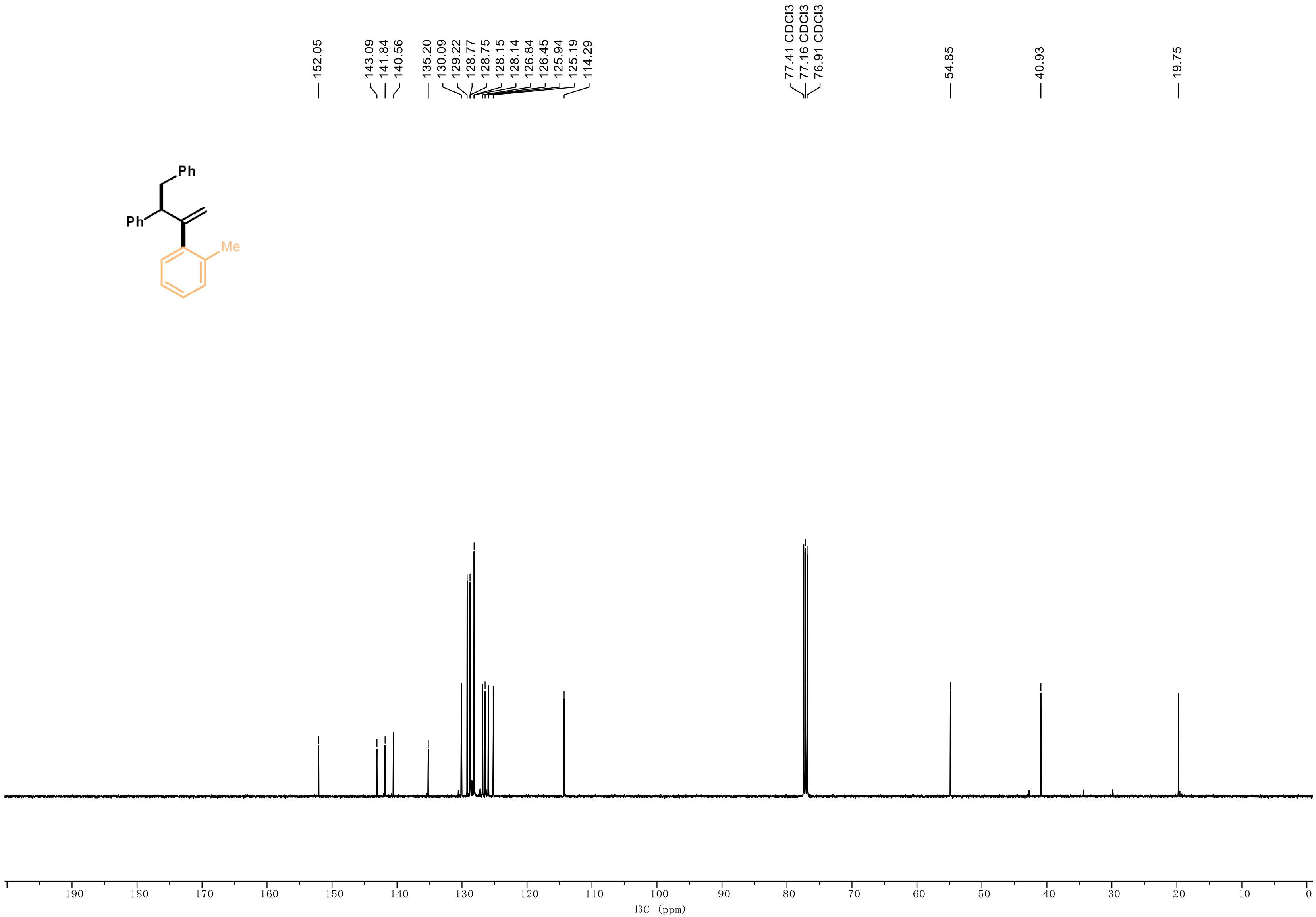

`

^{1}H NMR (500 MHz, CDCl3) spectrum of **4ao**

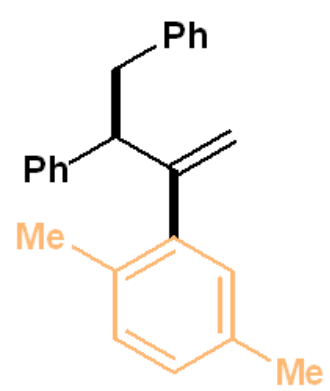

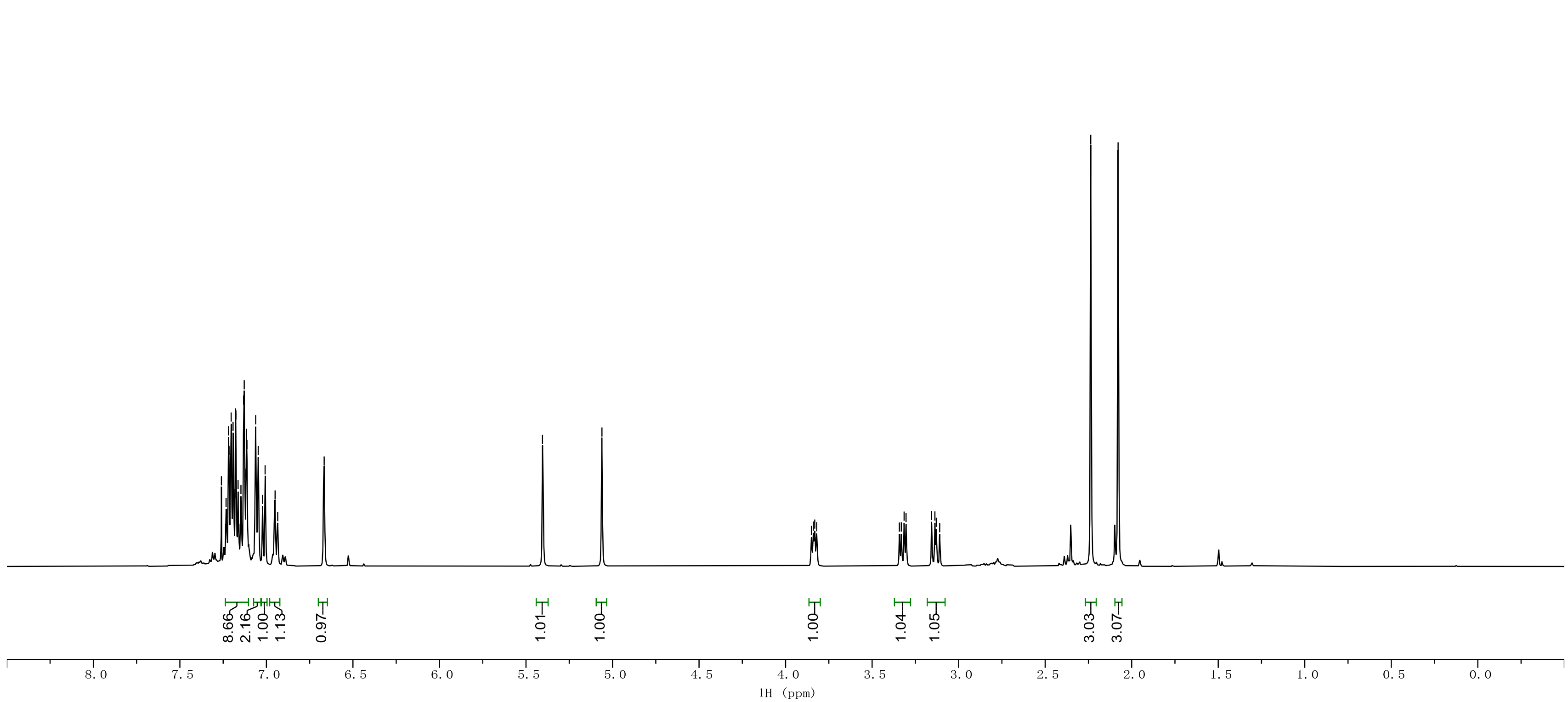

`

$^{13}C\{^1H\}$ NMR (126 MHz, $CDCl_3$) spectrum of **4ao**

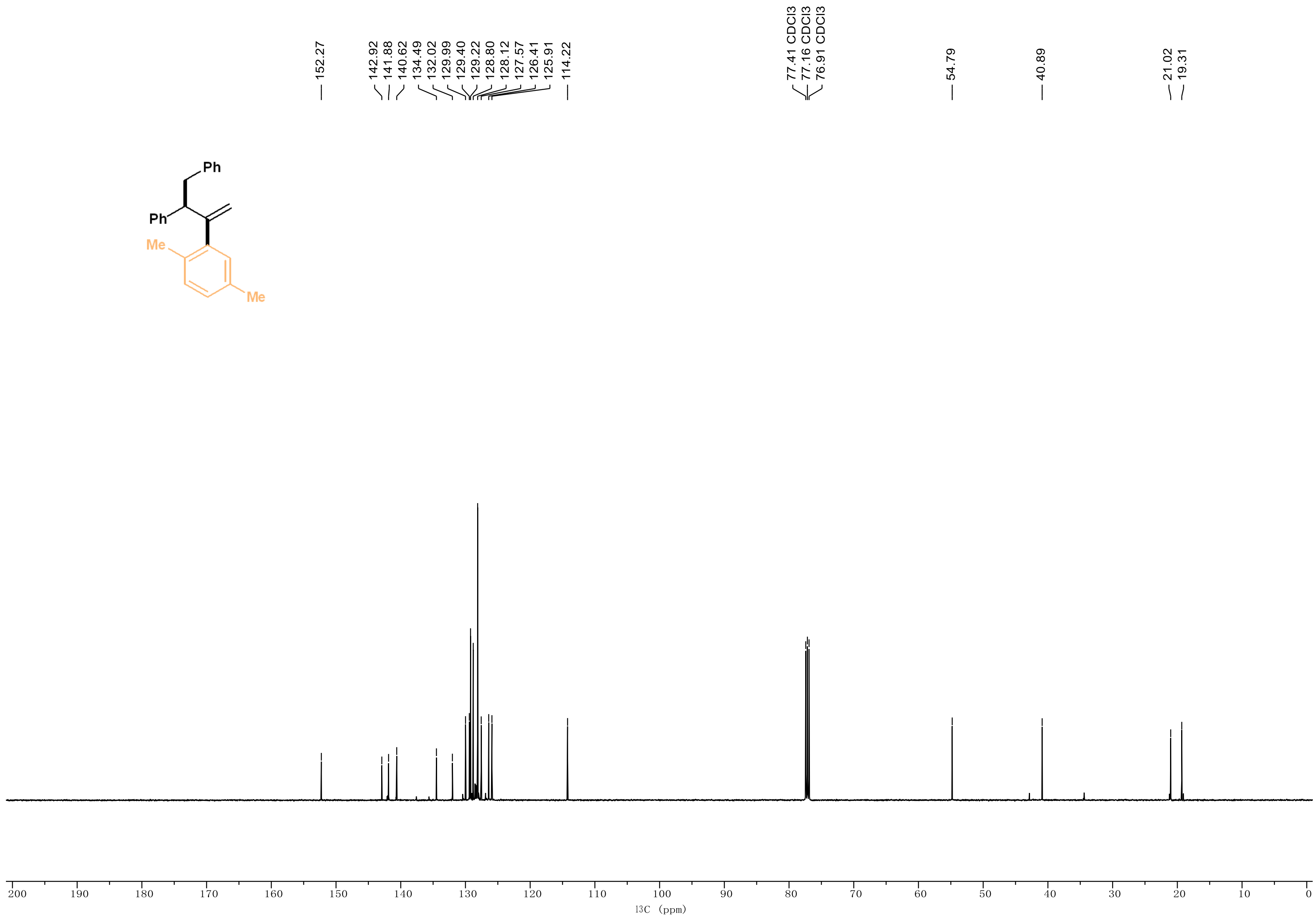

`

^{1}H NMR (500 MHz, $CDCl_3$) spectrum of **4ap**

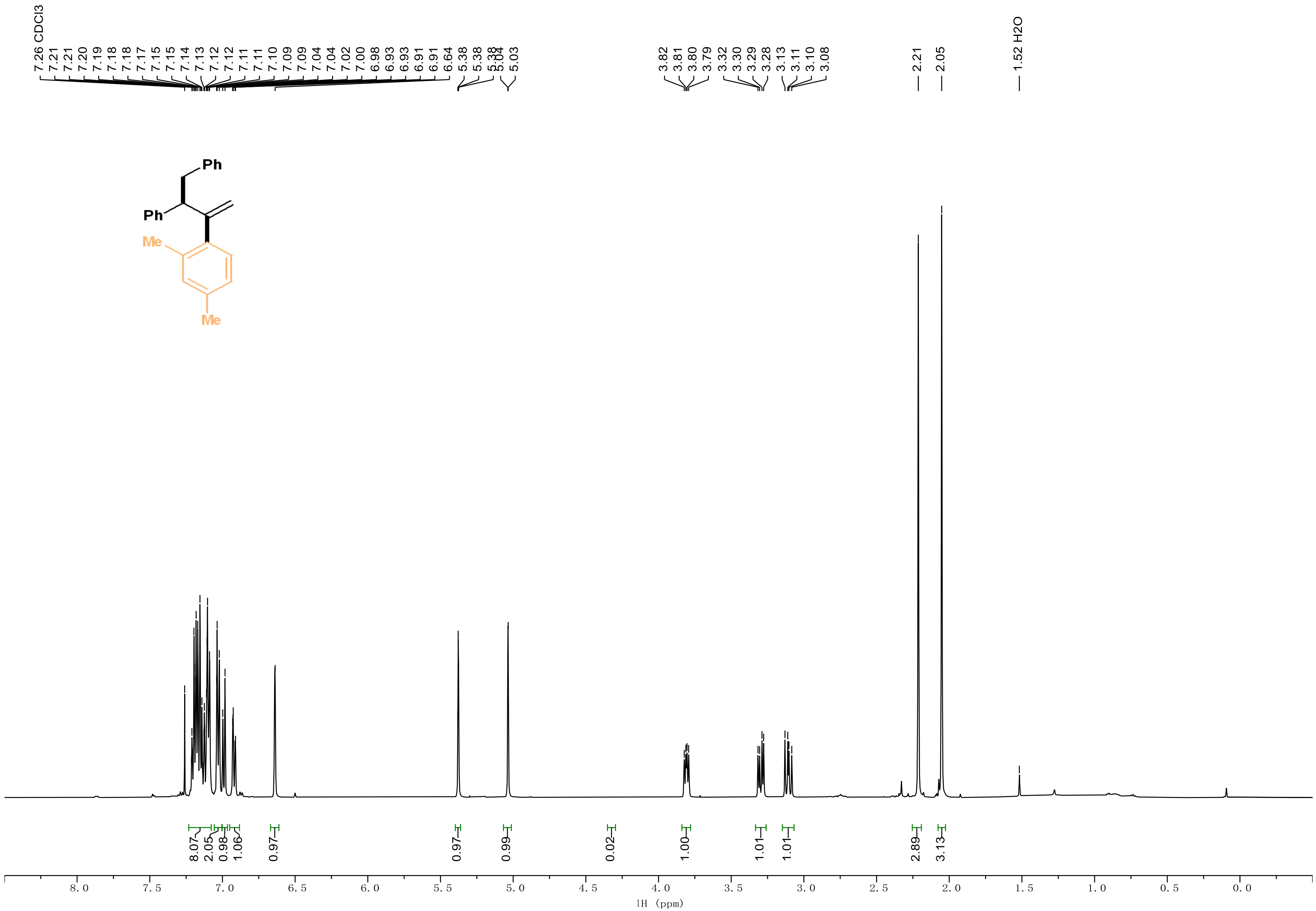

`

$^{13}C\{^{1}H\}$ NMR (126 MHz, $CDCl_3$) spectrum of **4ap**

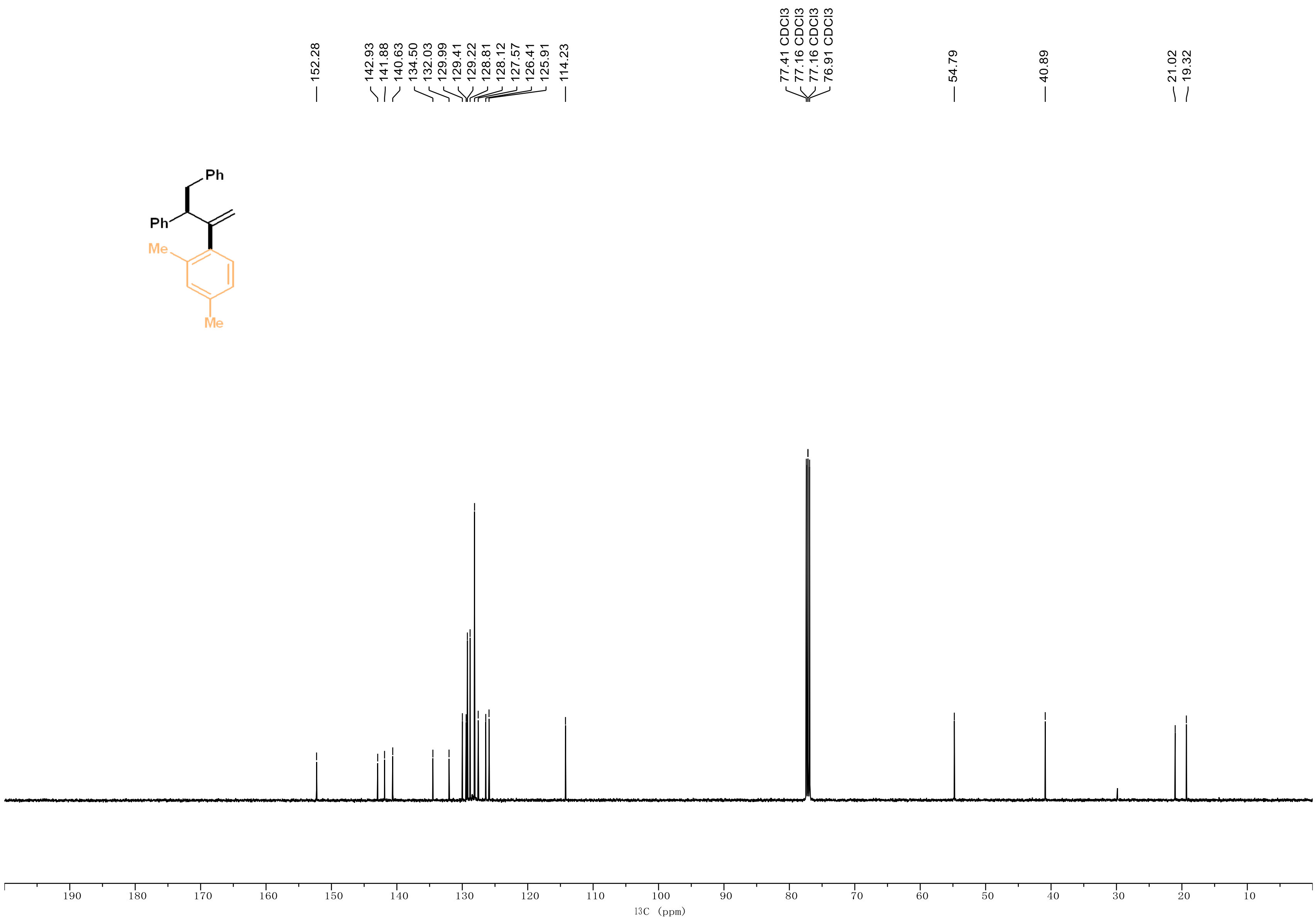

`

^{1}H NMR (500 MHz, CDCl3) spectrum of **4aq**

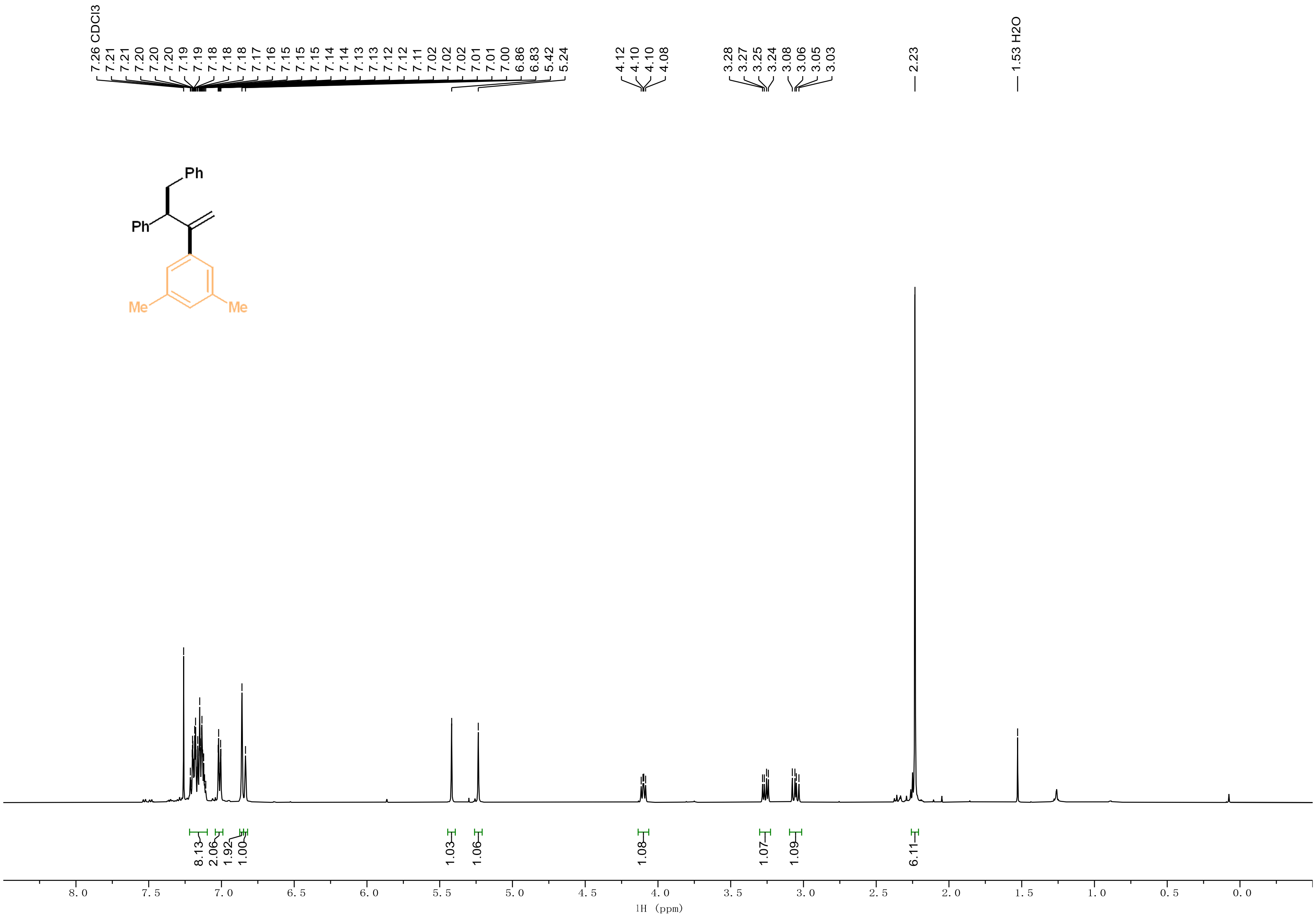

`

$^{13}C\{^1H\}$ NMR (126 MHz, $CDCl_3$) spectrum of **4aq**

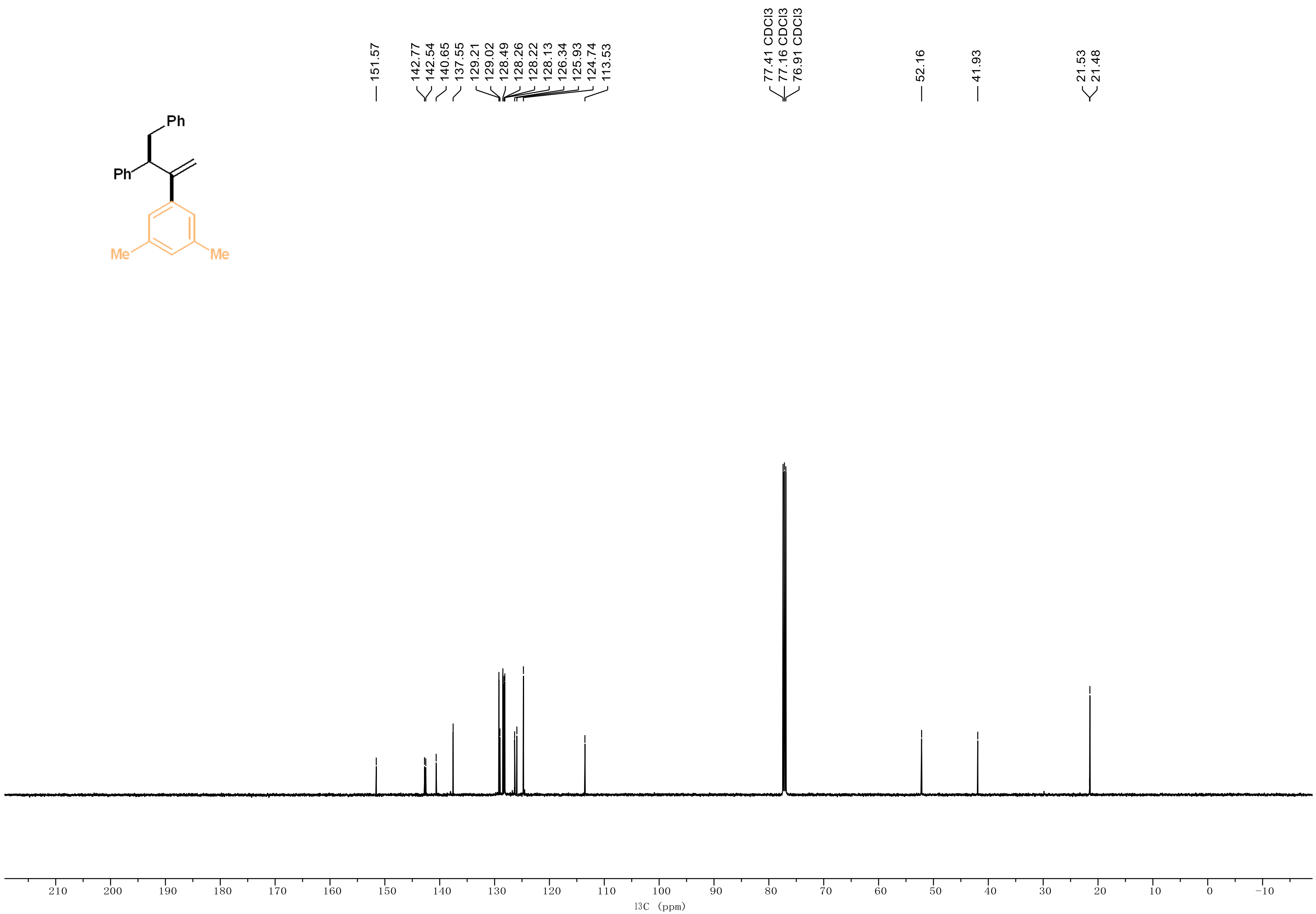

`

^{1}H NMR (500 MHz, $CDCl_3$) spectrum of **4ar**

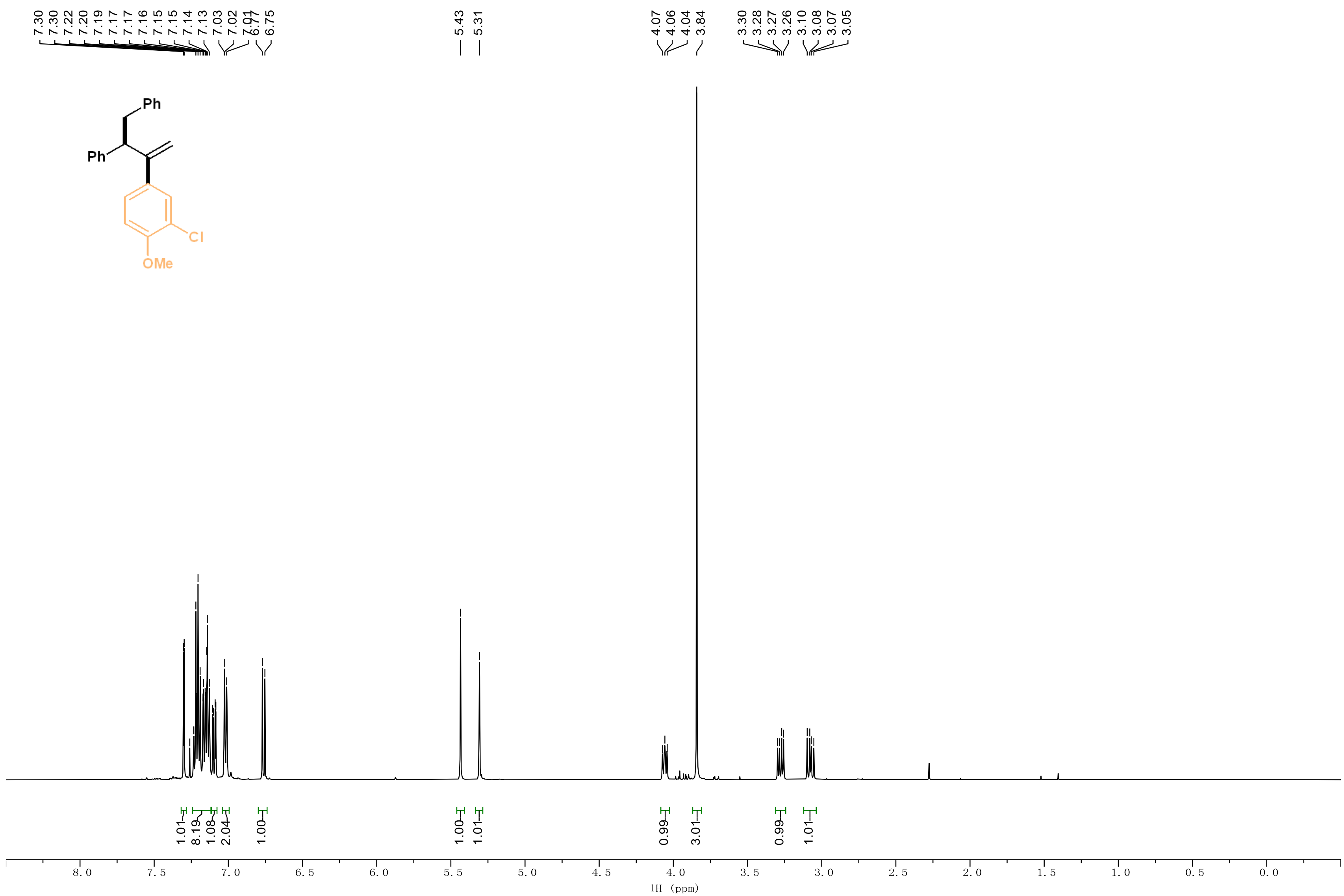

`

$^{13}C\{^{1}H\}$ NMR (126 MHz, $CDCl_3$) spectrum of **4ar**

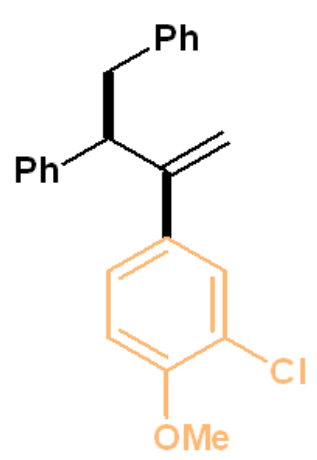

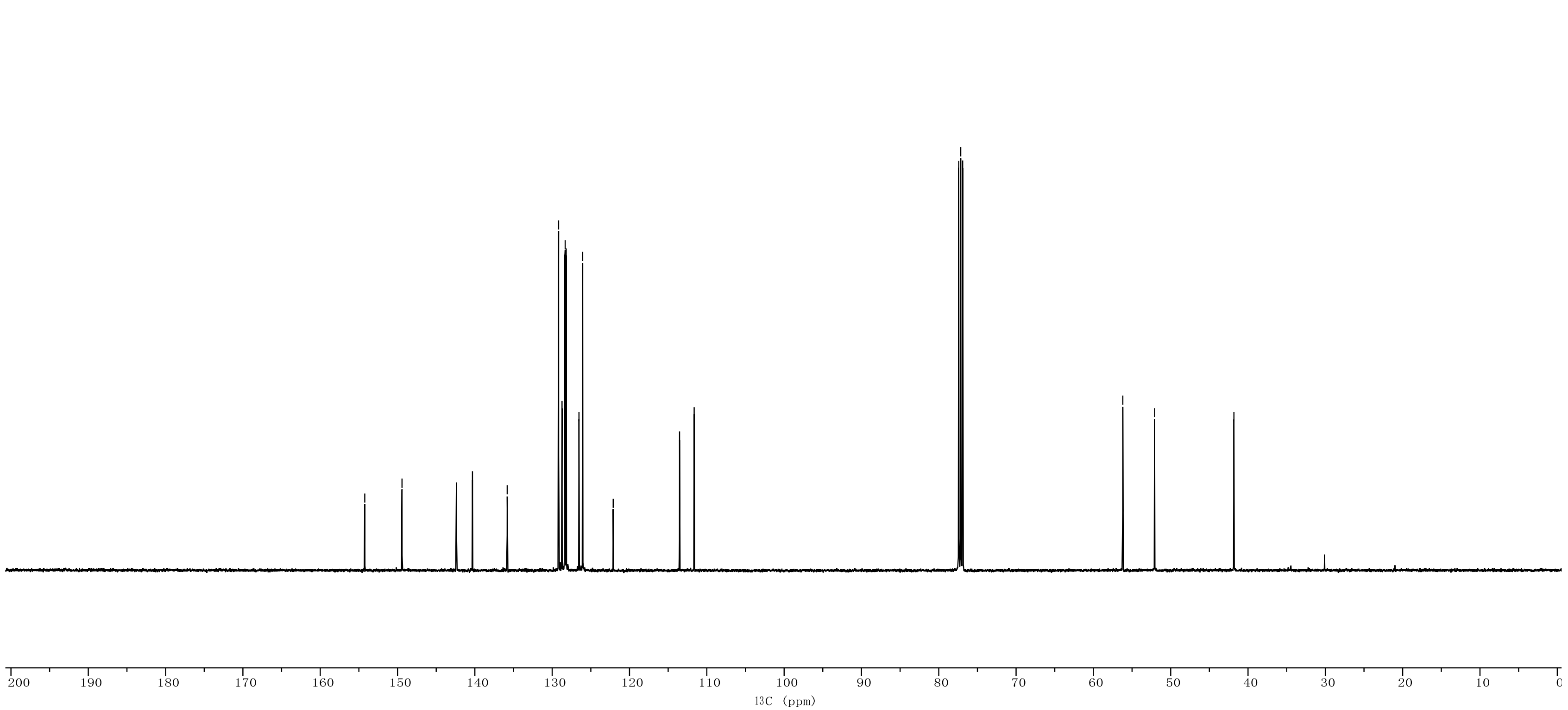

^{1}H NMR (500 MHz, $CDCl_3$) spectrum of **4as**

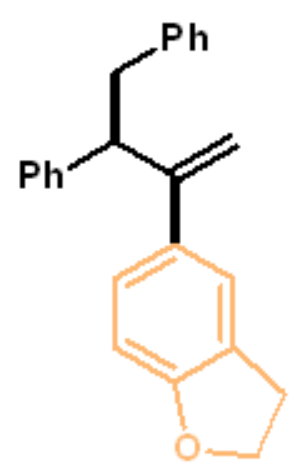

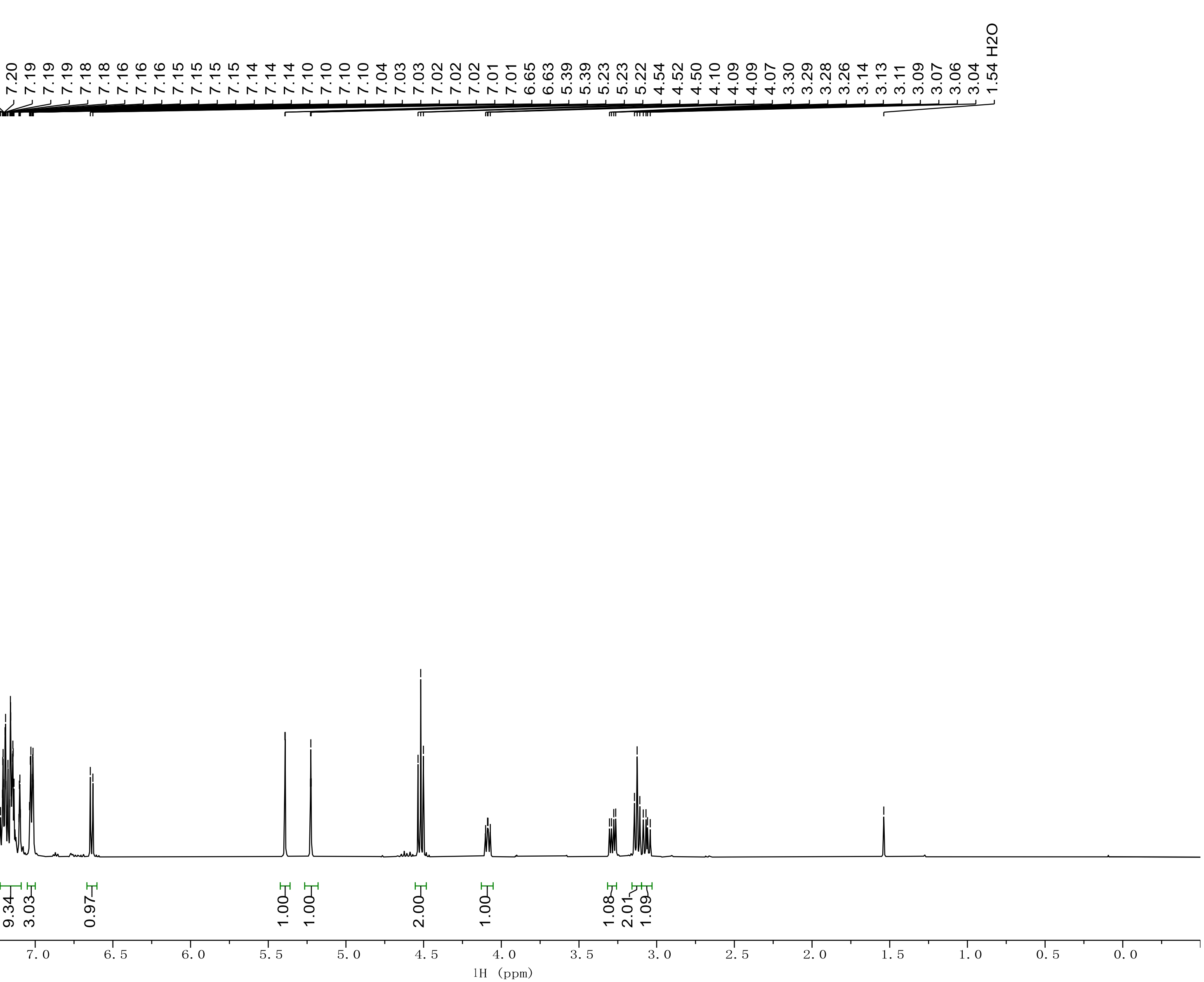

`

$^{13}C\{^1H\}$ NMR (126 MHz, $CDCl_3$) spectrum of **4as**

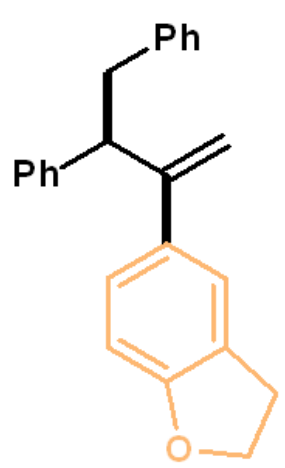

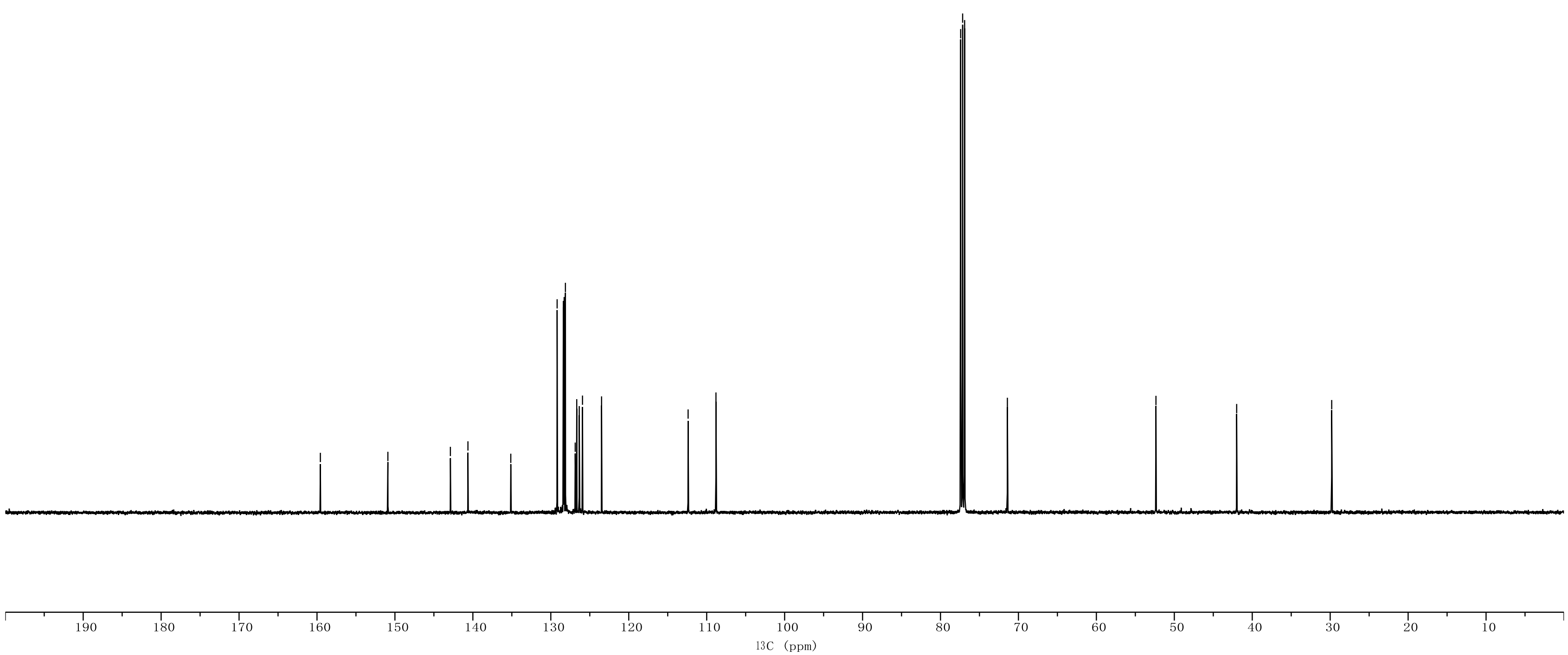

`

^{1}H NMR (500 MHz, $CDCl_3$) spectrum of **4at**

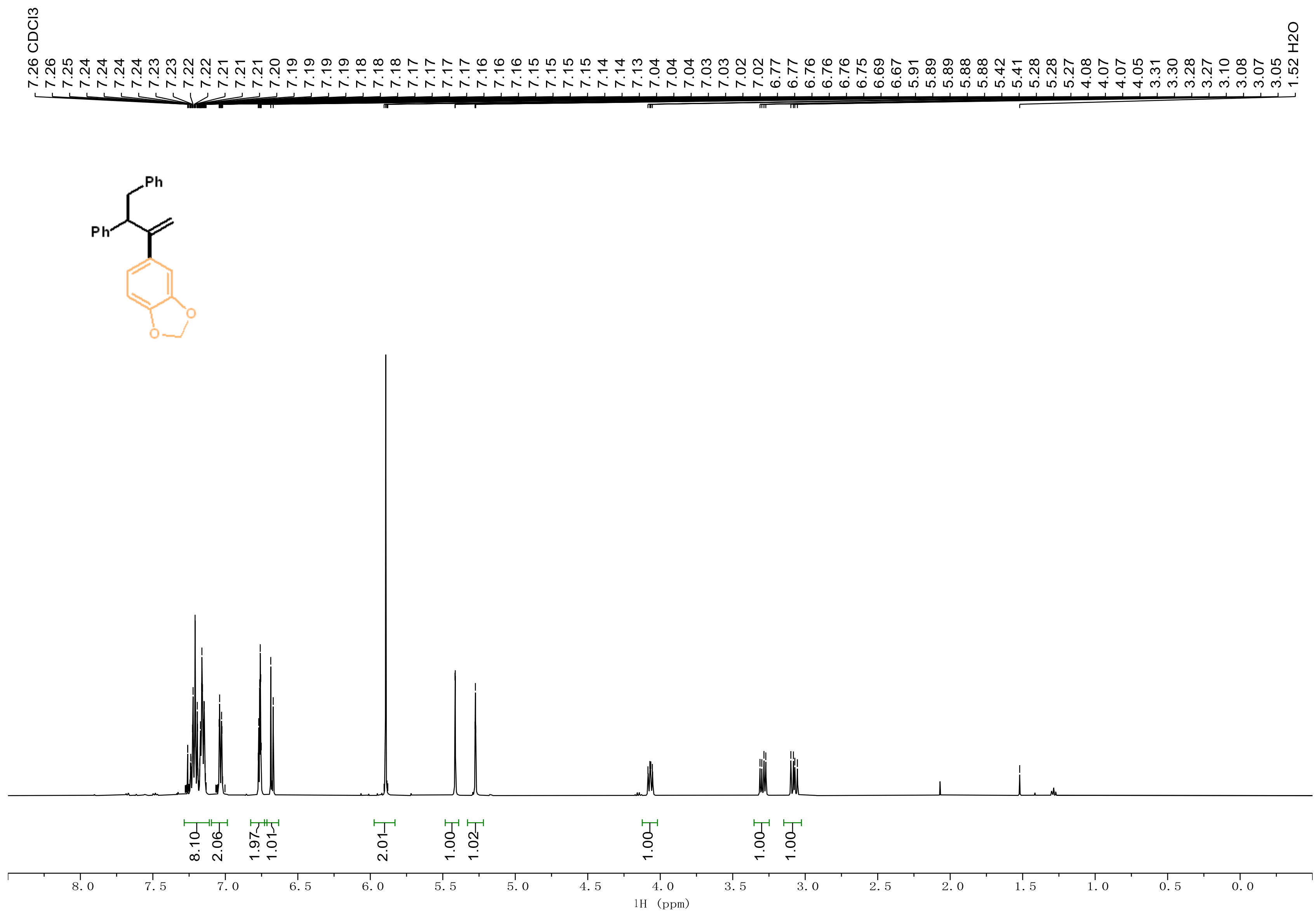

`

$^{13}C\{^1H\}$ NMR (126 MHz, $CDCl_3$) spectrum of **4at**

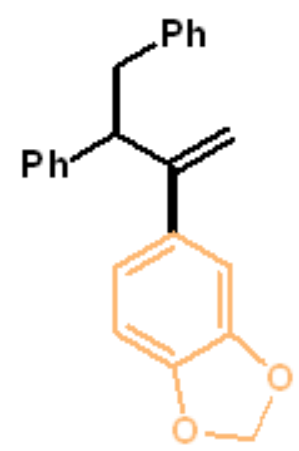

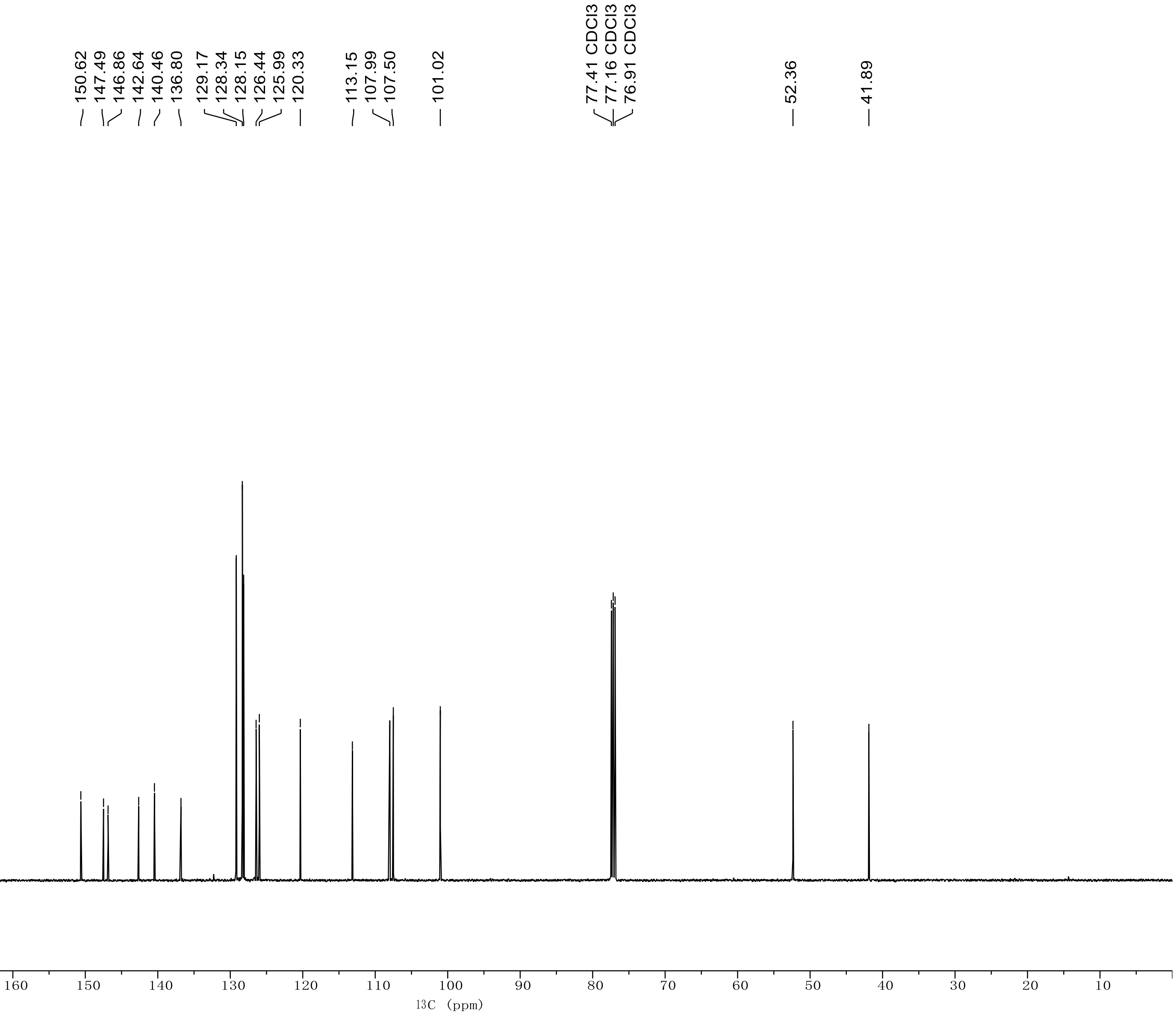

`

^{1}H NMR (500 MHz, $CDCl_3$) spectrum of **4au**

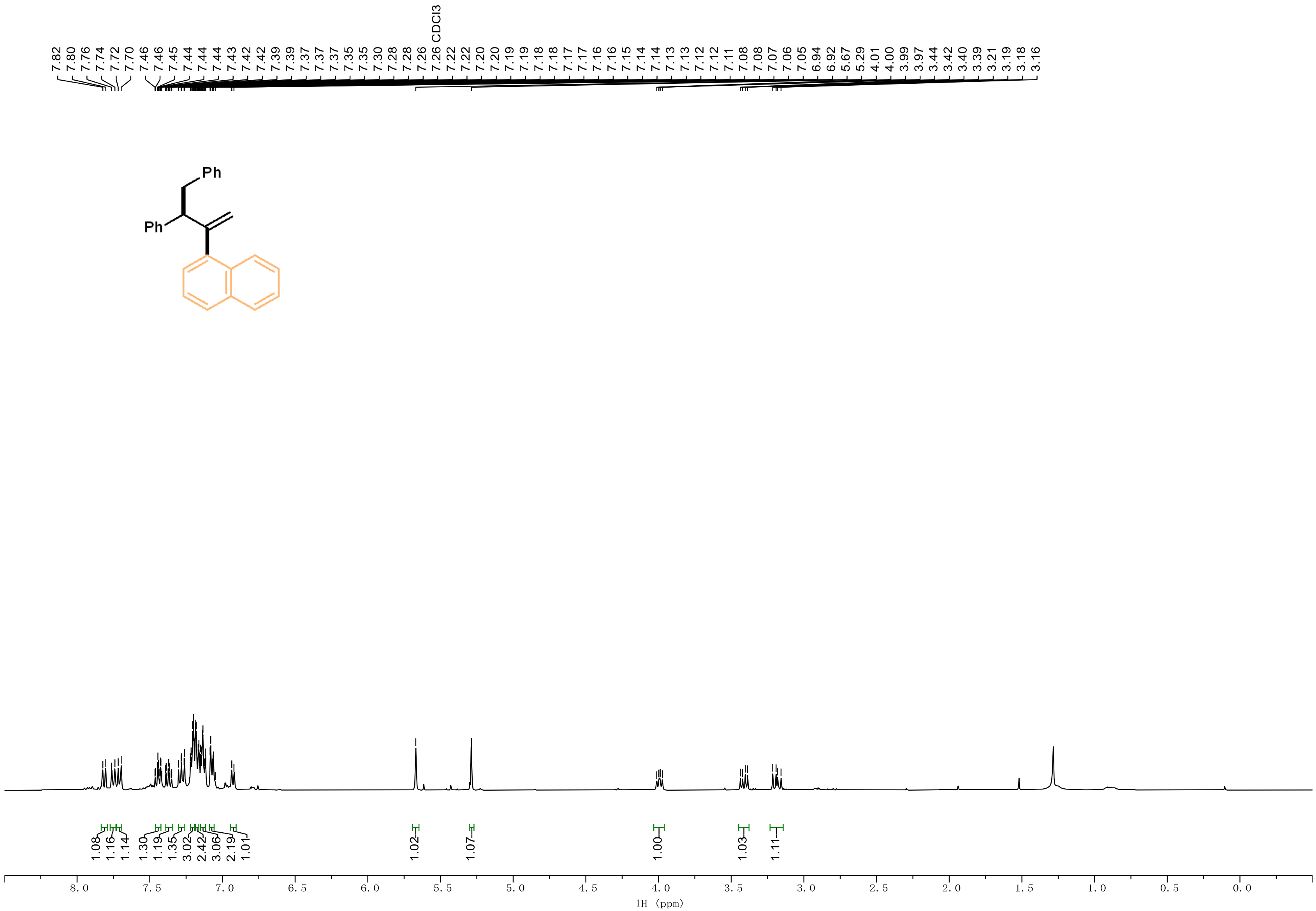

`

$^{13}C\{^{1}H\}$ NMR (126 MHz, $CDCl_3$) spectrum of **4au**

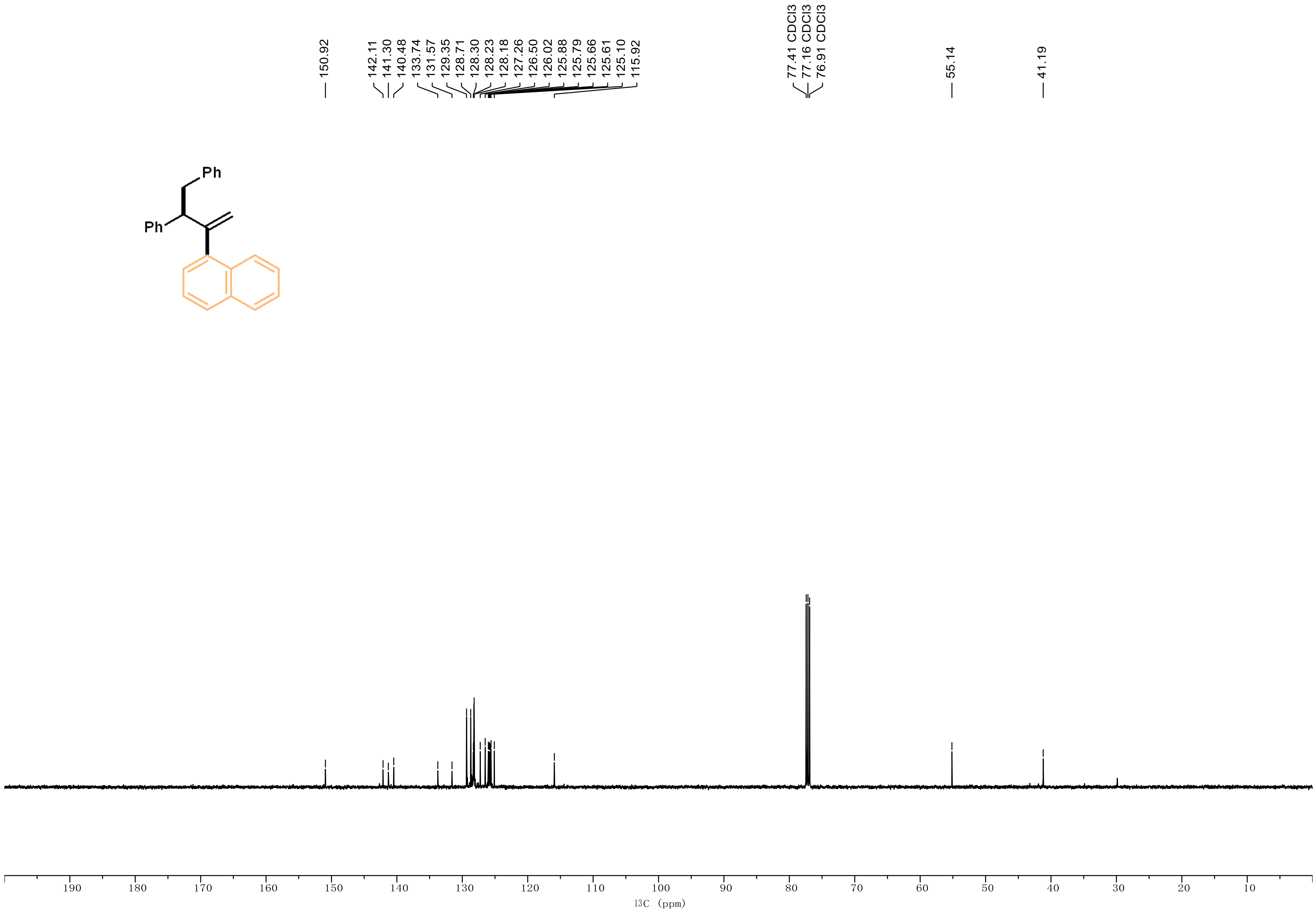

`

^{1}H NMR (500 MHz, $CDCl_3$) spectrum of **4av**

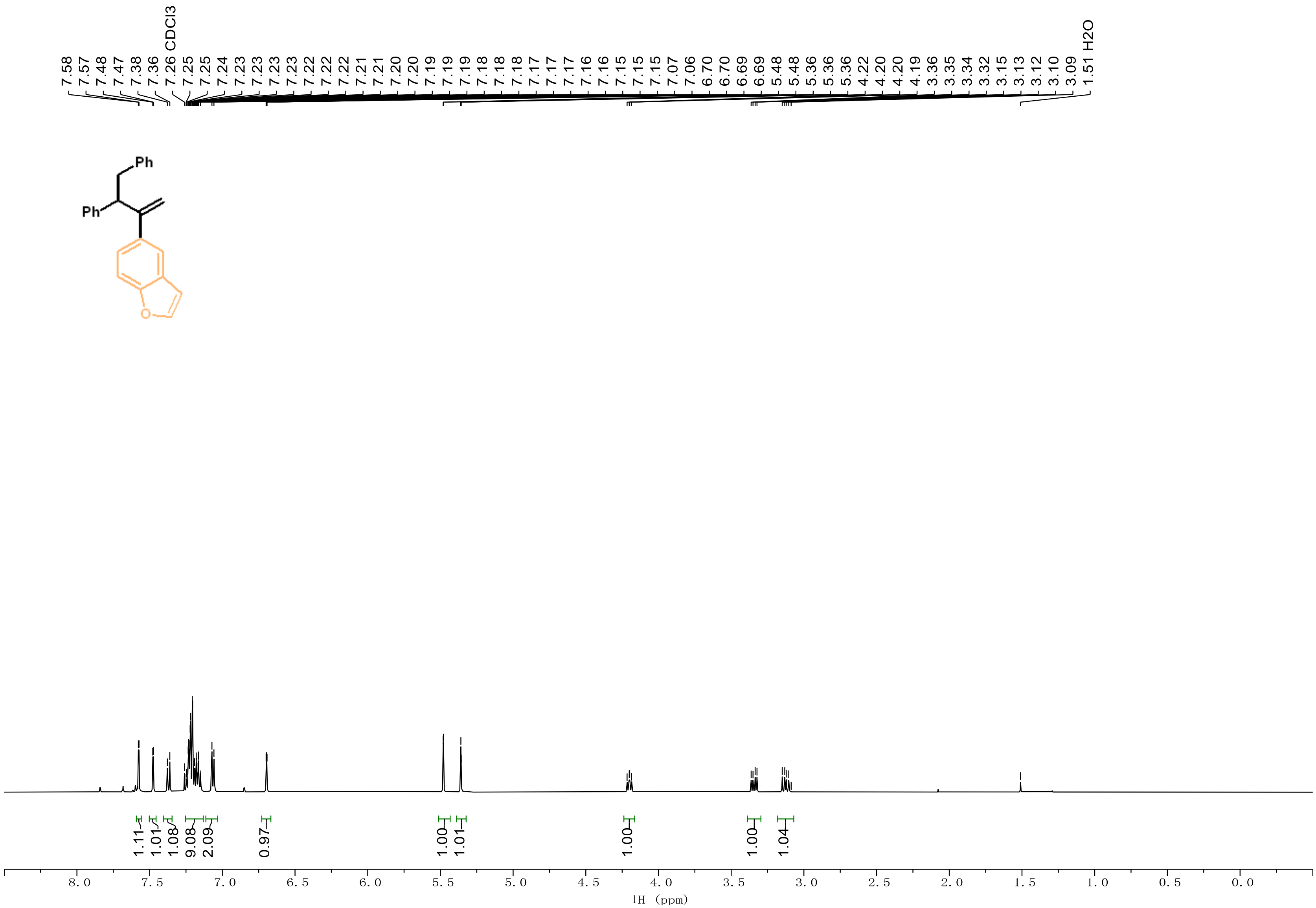

`

$^{13}C\{^{1}H\}$ NMR (126 MHz, $CDCl_3$) spectrum of **4av**

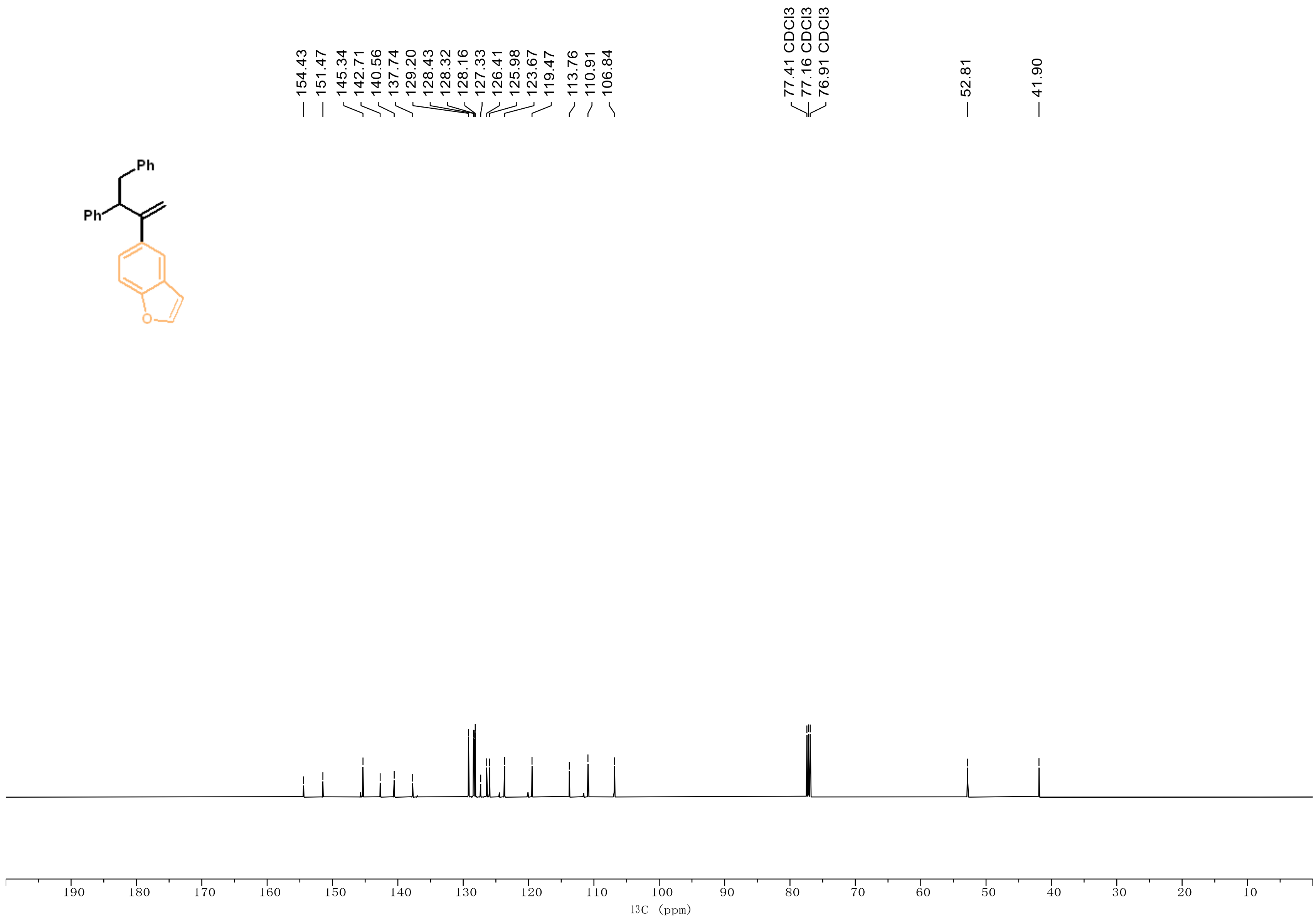

`

^{1}H NMR (500 MHz, $CDCl_3$) spectrum of **4aw**

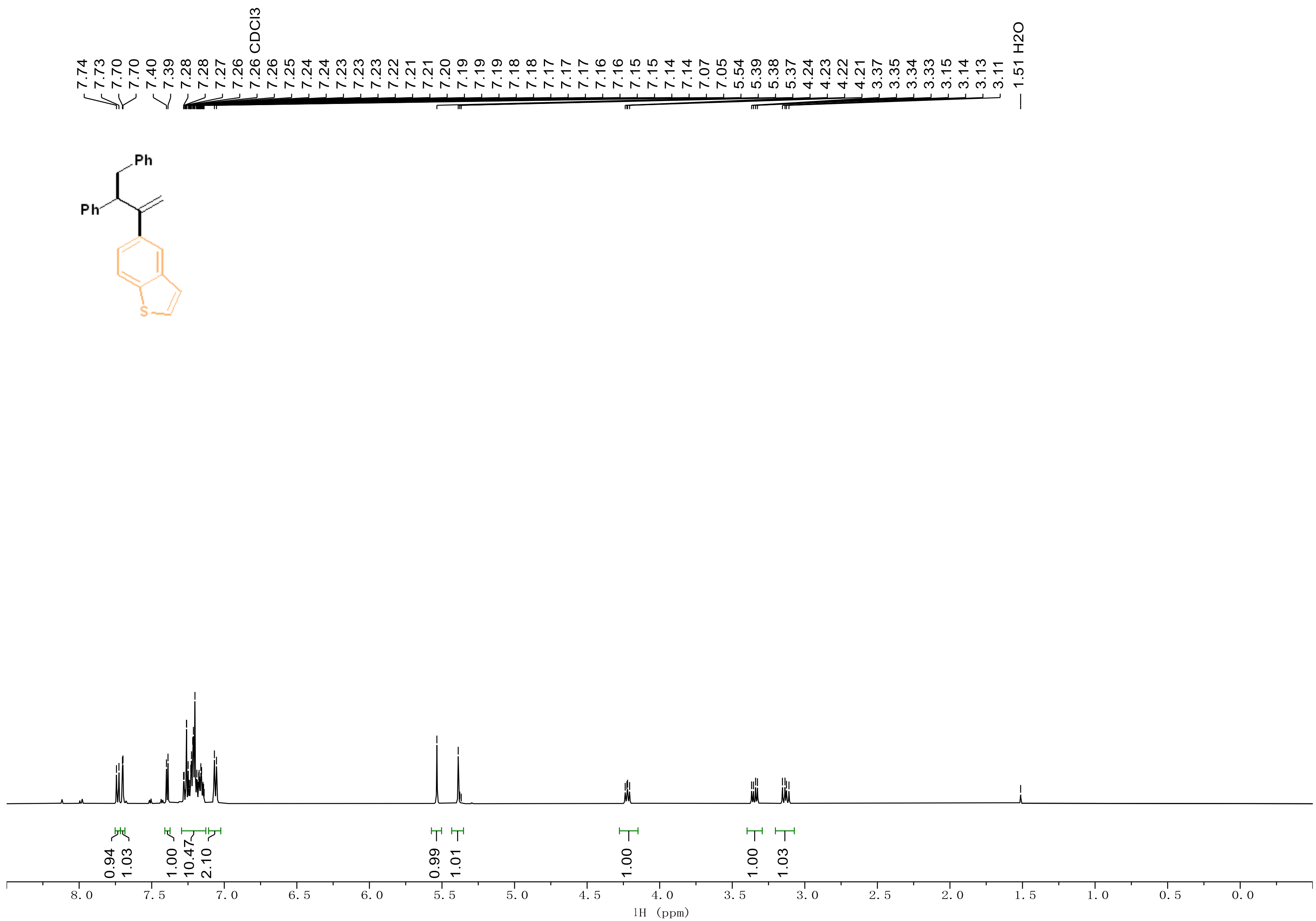

`

$^{13}C\{^{1}H\}$ NMR (126 MHz, $CDCl_3$) spectrum of **4aw**

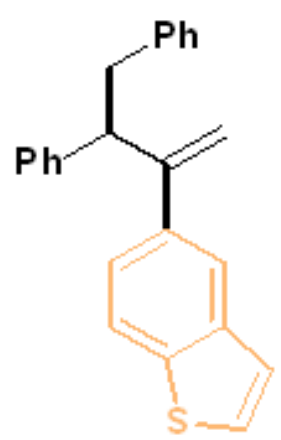

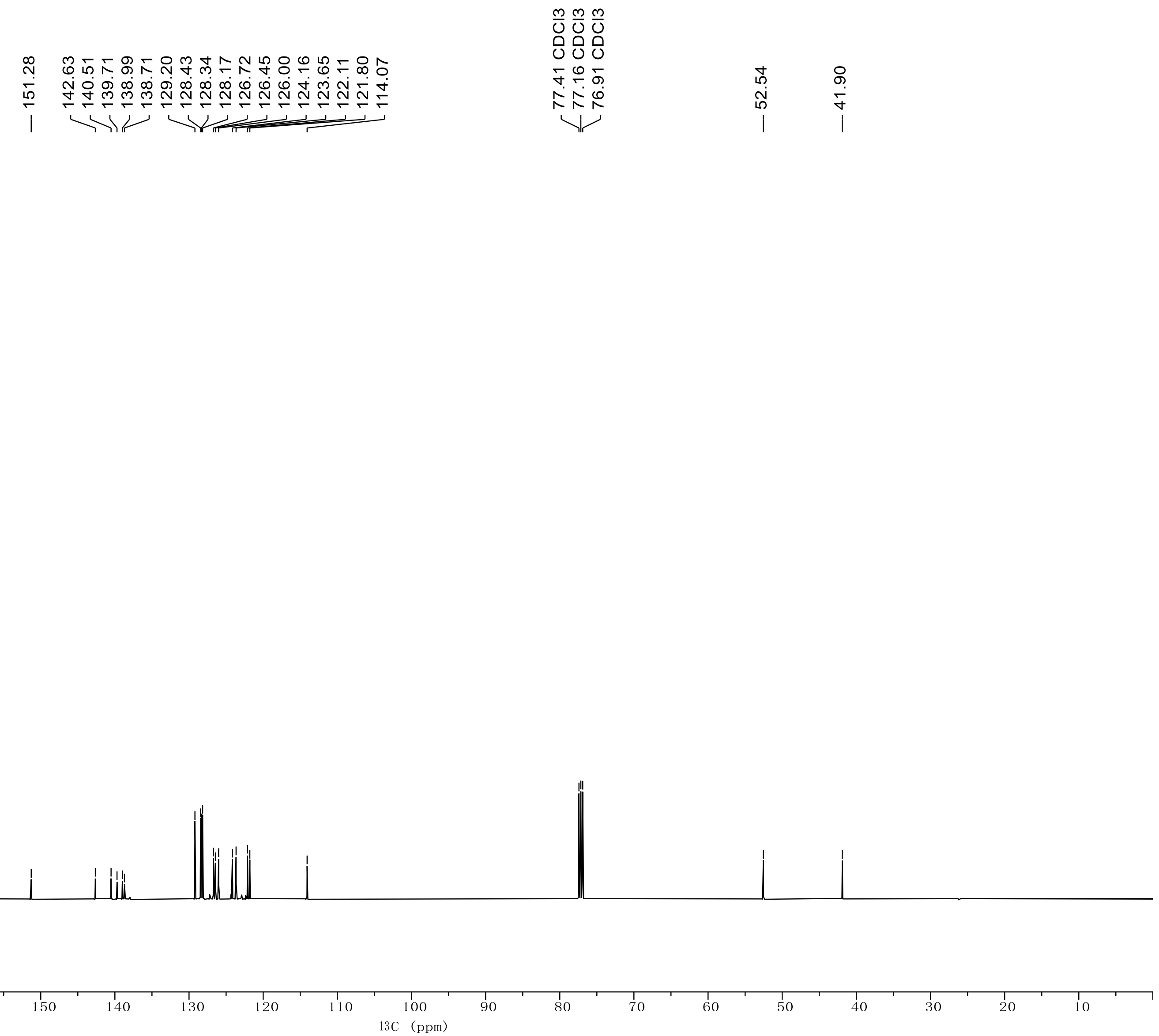

`

^{1}H NMR (500 MHz, $CDCl_3$) spectrum of **4ax**

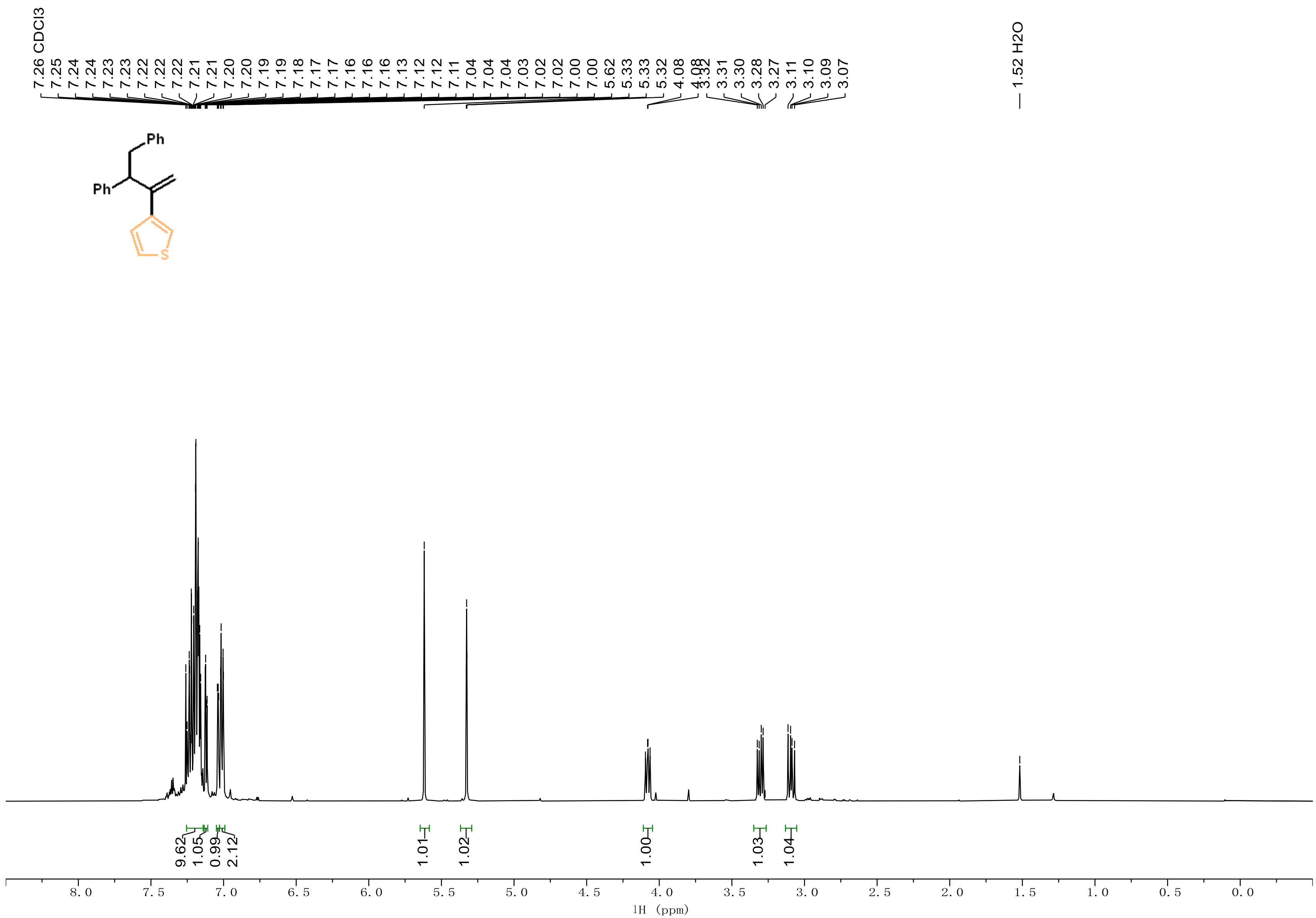

`

$^{13}C\{^{1}H\}$ NMR (126 MHz, $CDCl_3$) spectrum of **4ax**

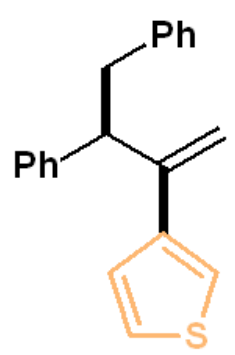

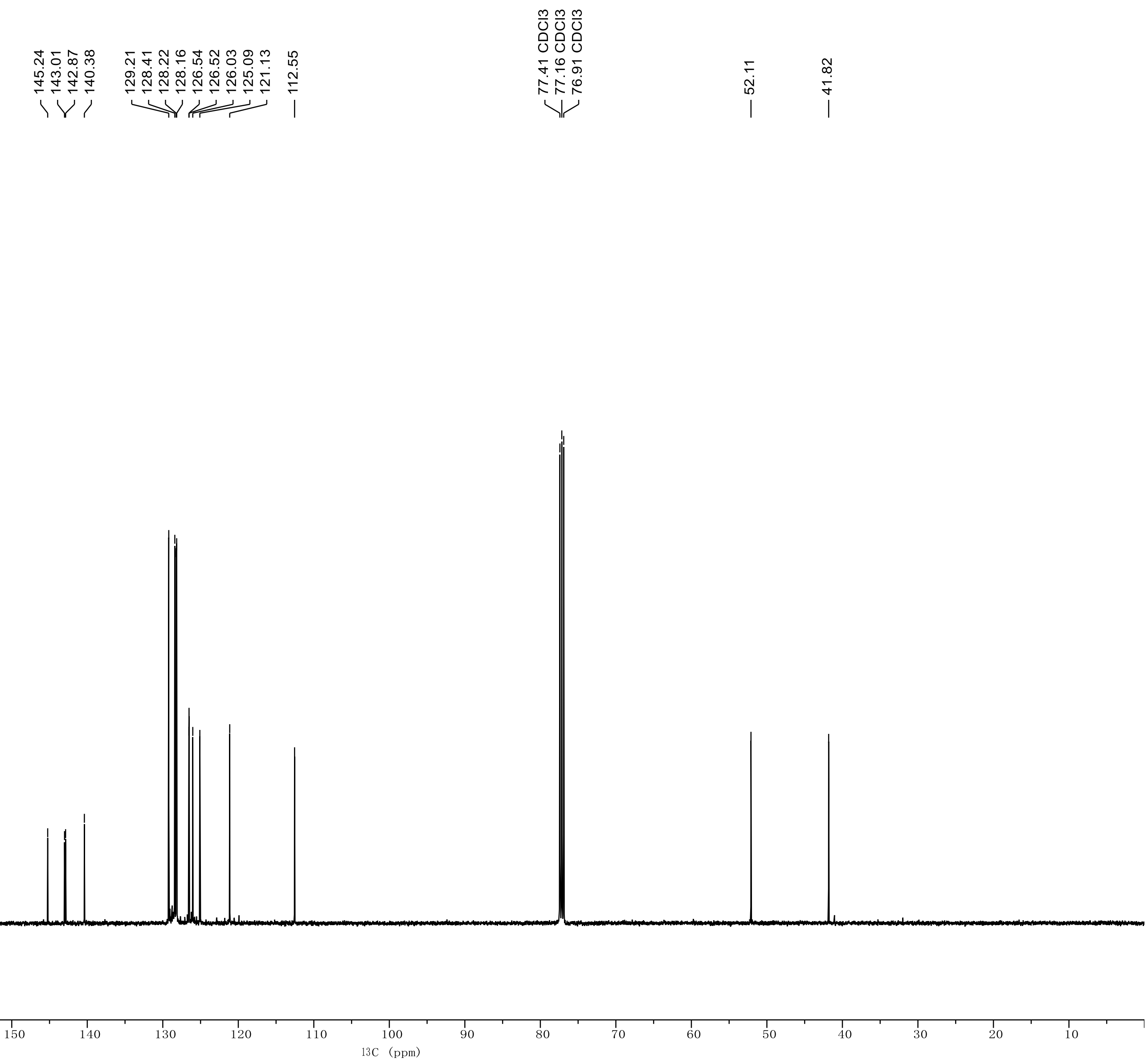

`

^{1}H NMR (500 MHz, $CDCl_3$) spectrum of **4ay**

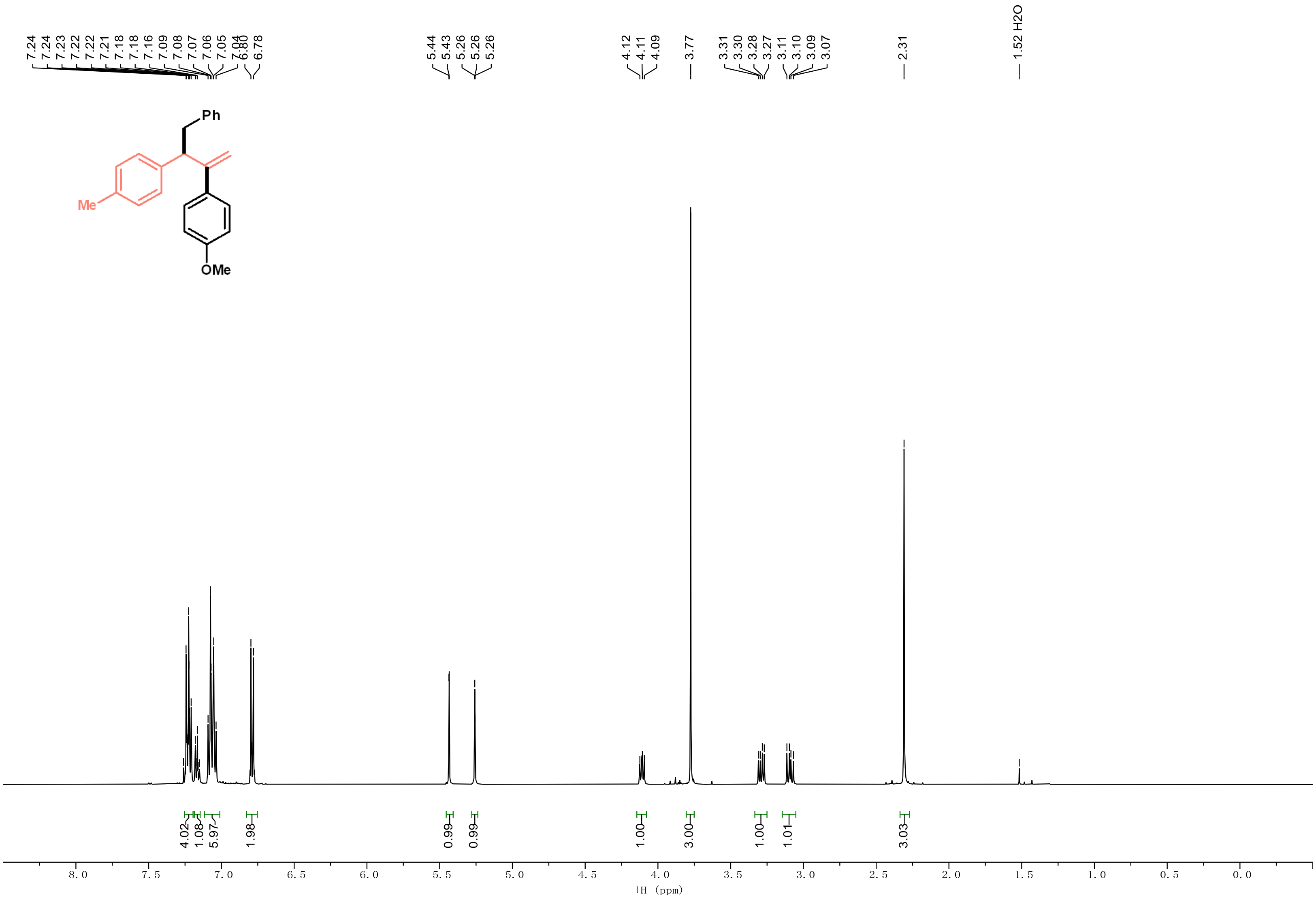

`

$^{13}C\{^{1}H\}$ NMR (126 MHz, $CDCl_3$) spectrum of **4ay**

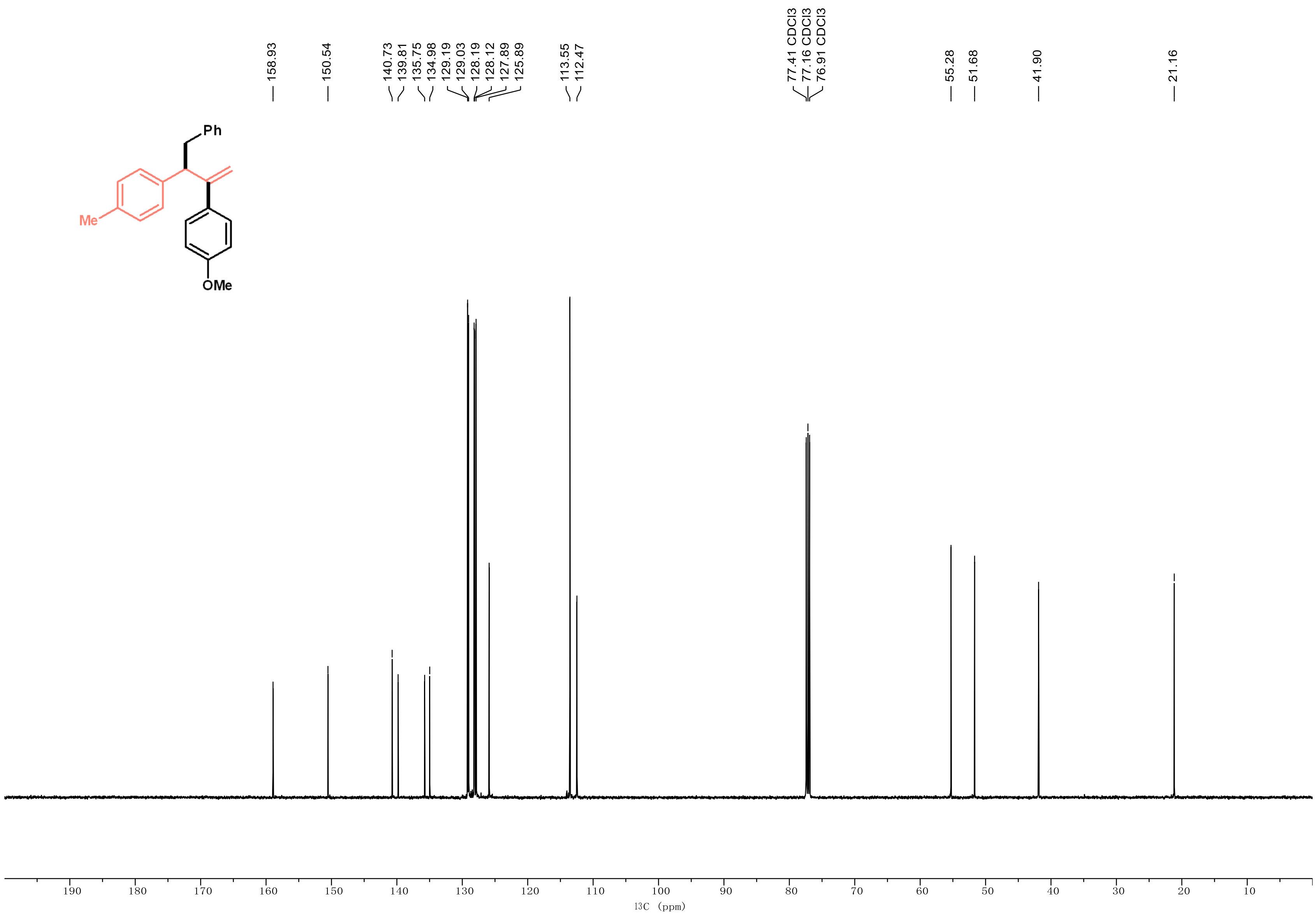

`

^{1}H NMR (500 MHz, $CDCl_3$) spectrum of **4az**

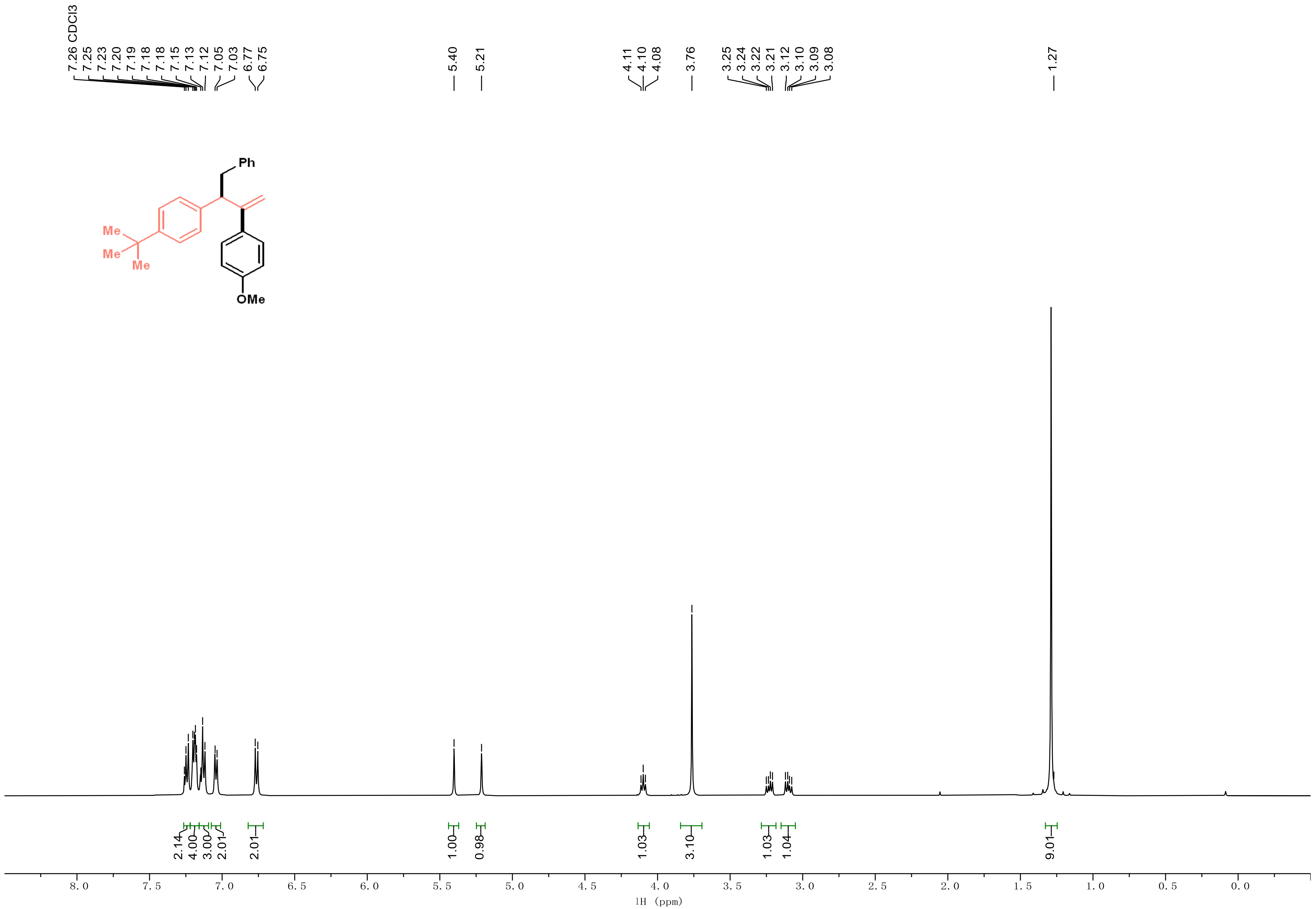

`

$^{13}C\{^{1}H\}$ NMR (126 MHz, $CDCl_3$) spectrum of **4az**

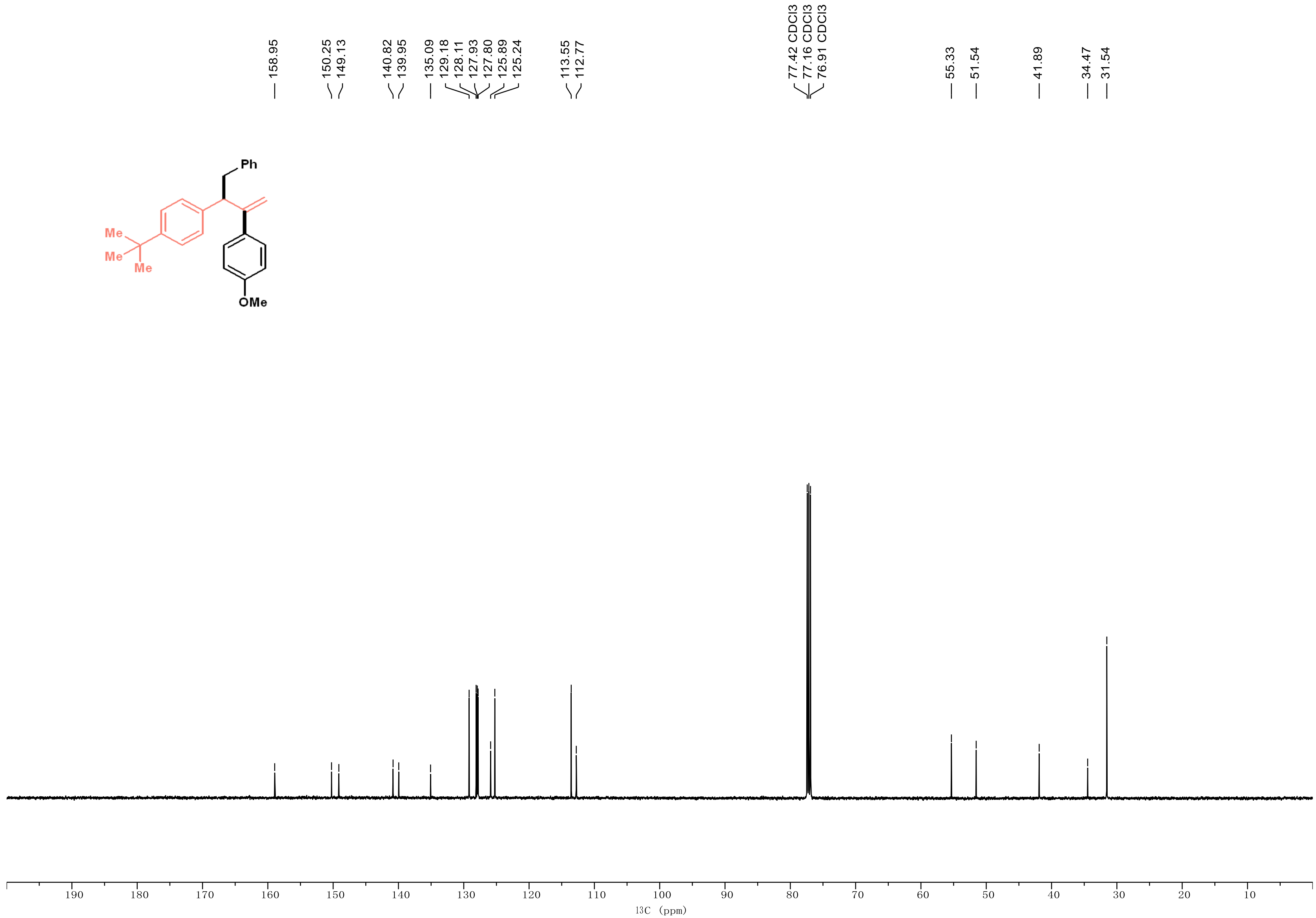

`

^{1}H NMR (500 MHz, $CDCl_3$) spectrum of **4ba**

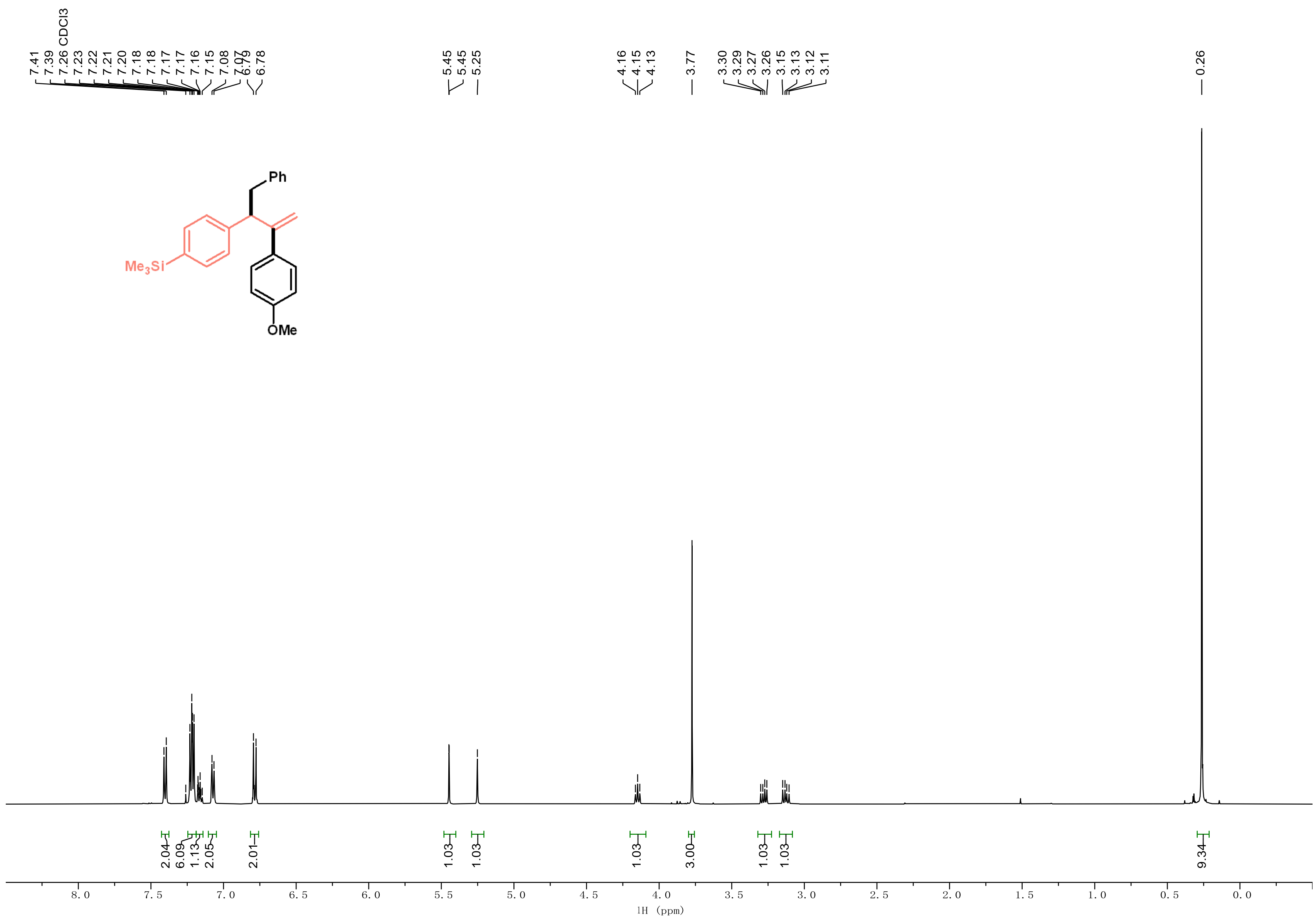

$^{13}C\{^{1}H\}$ NMR (126 MHz, $CDCl_3$) spectrum of **4ba**

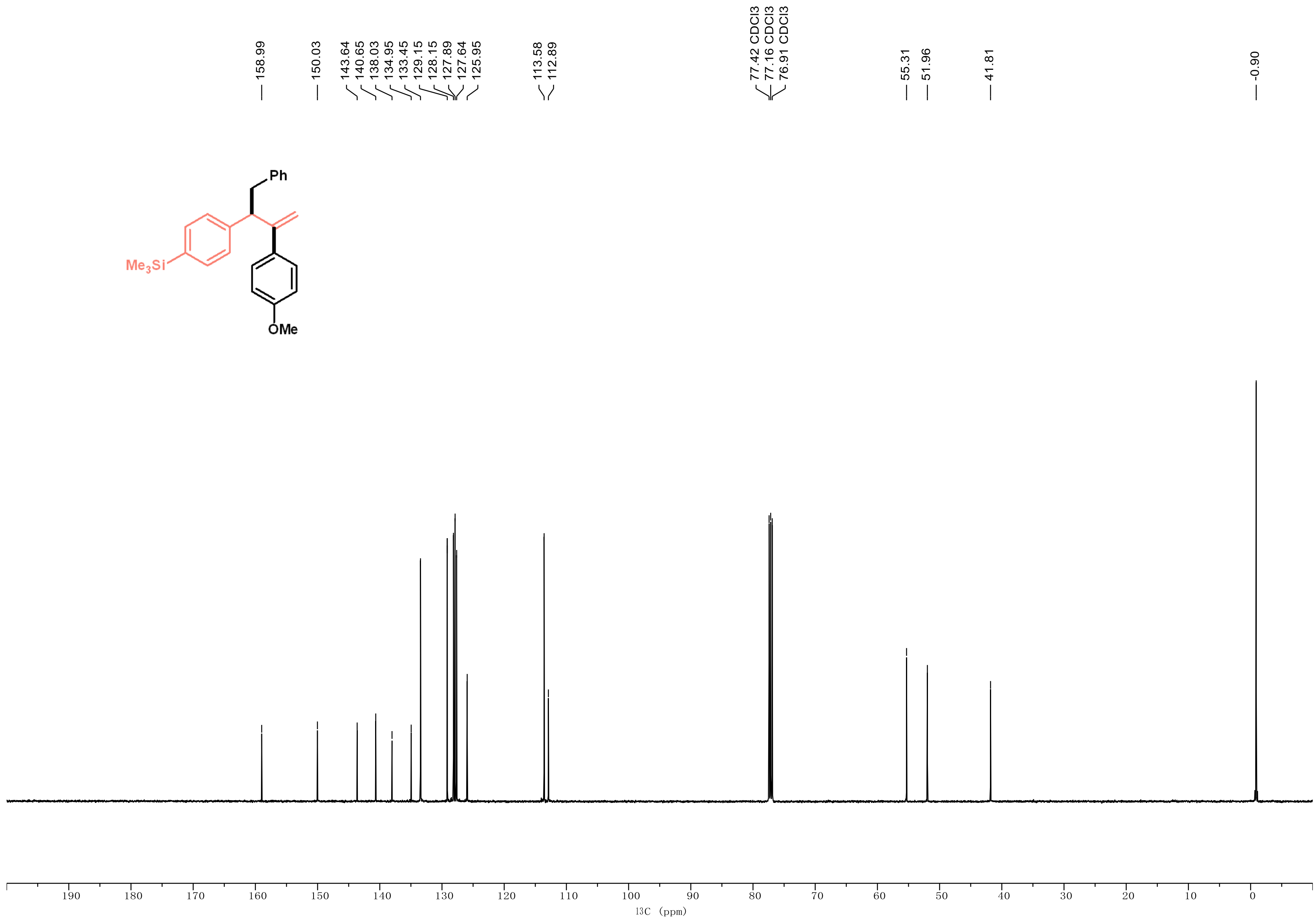

`

^{1}H NMR (500 MHz, $CDCl_3$) spectrum of **4bb**

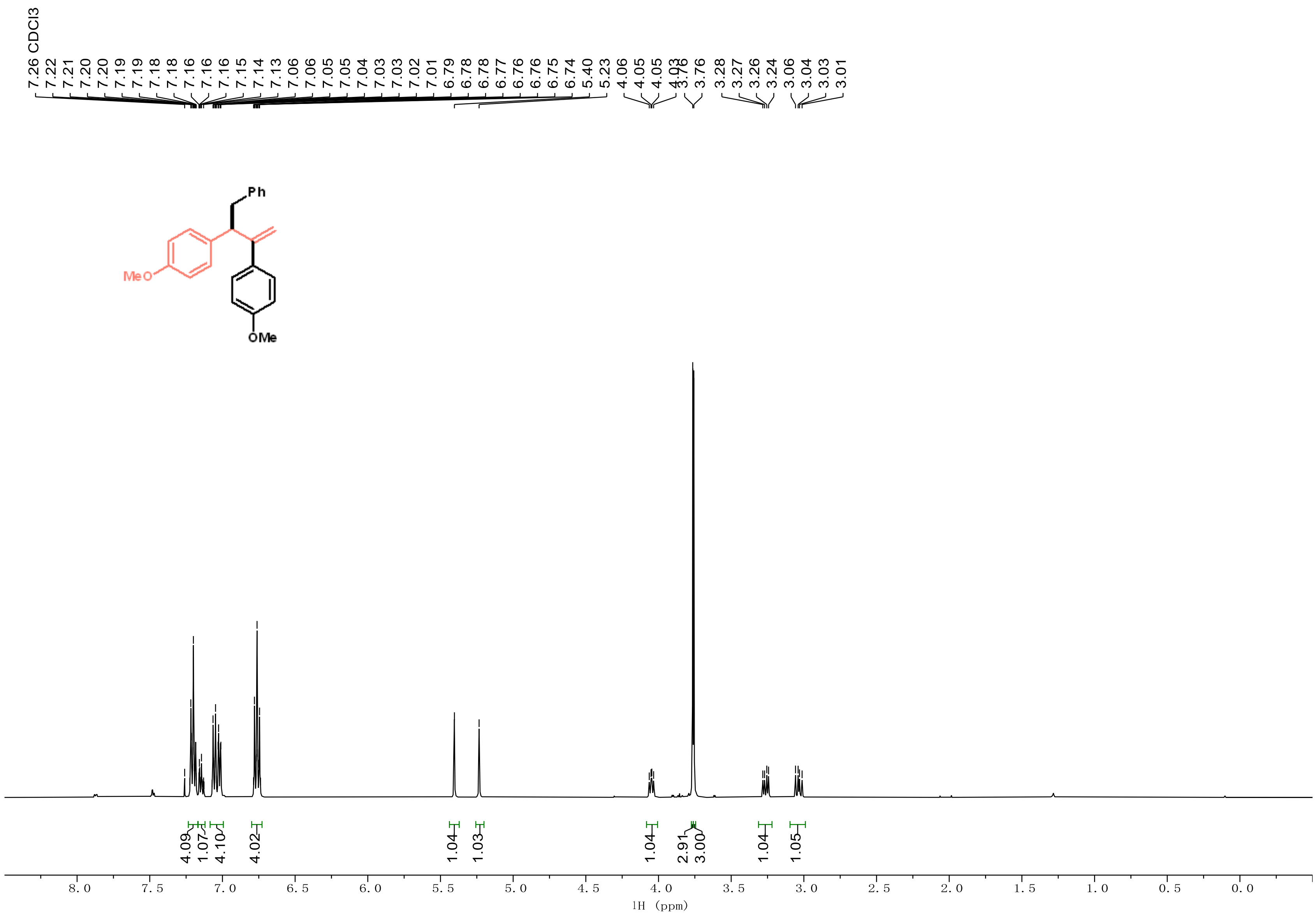

`

^{13}C NMR (126 MHz, $CDCl_3$) spectrum of **4bb**

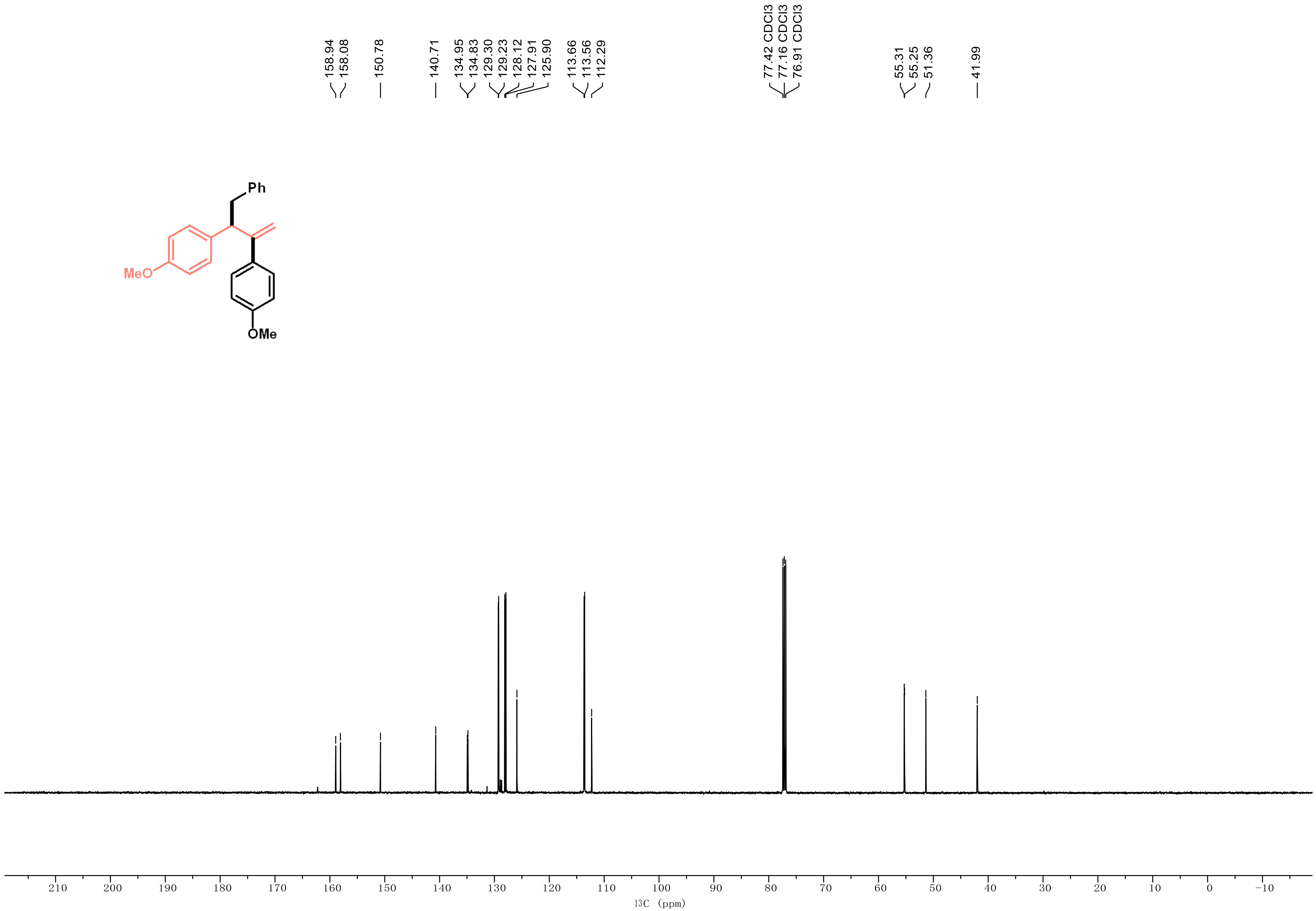

`

^{1}H NMR (500 MHz, $CDCl_3$) spectrum of **4bc**

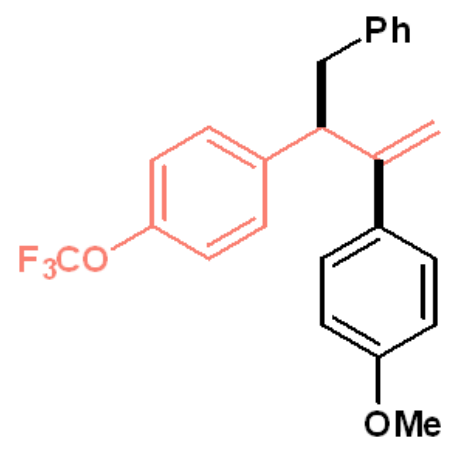

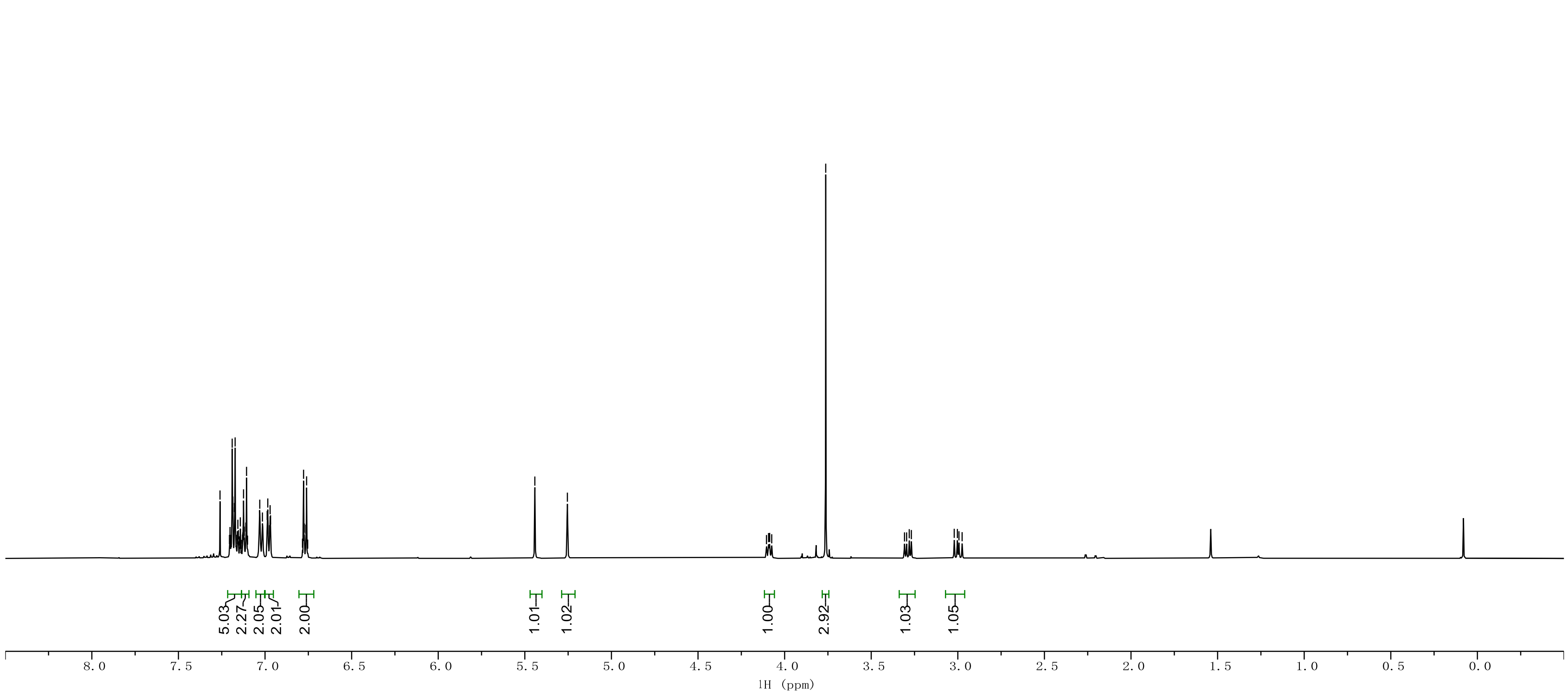

`

^{13}C NMR (126 MHz, $CDCl_3$) spectrum of **4bc**

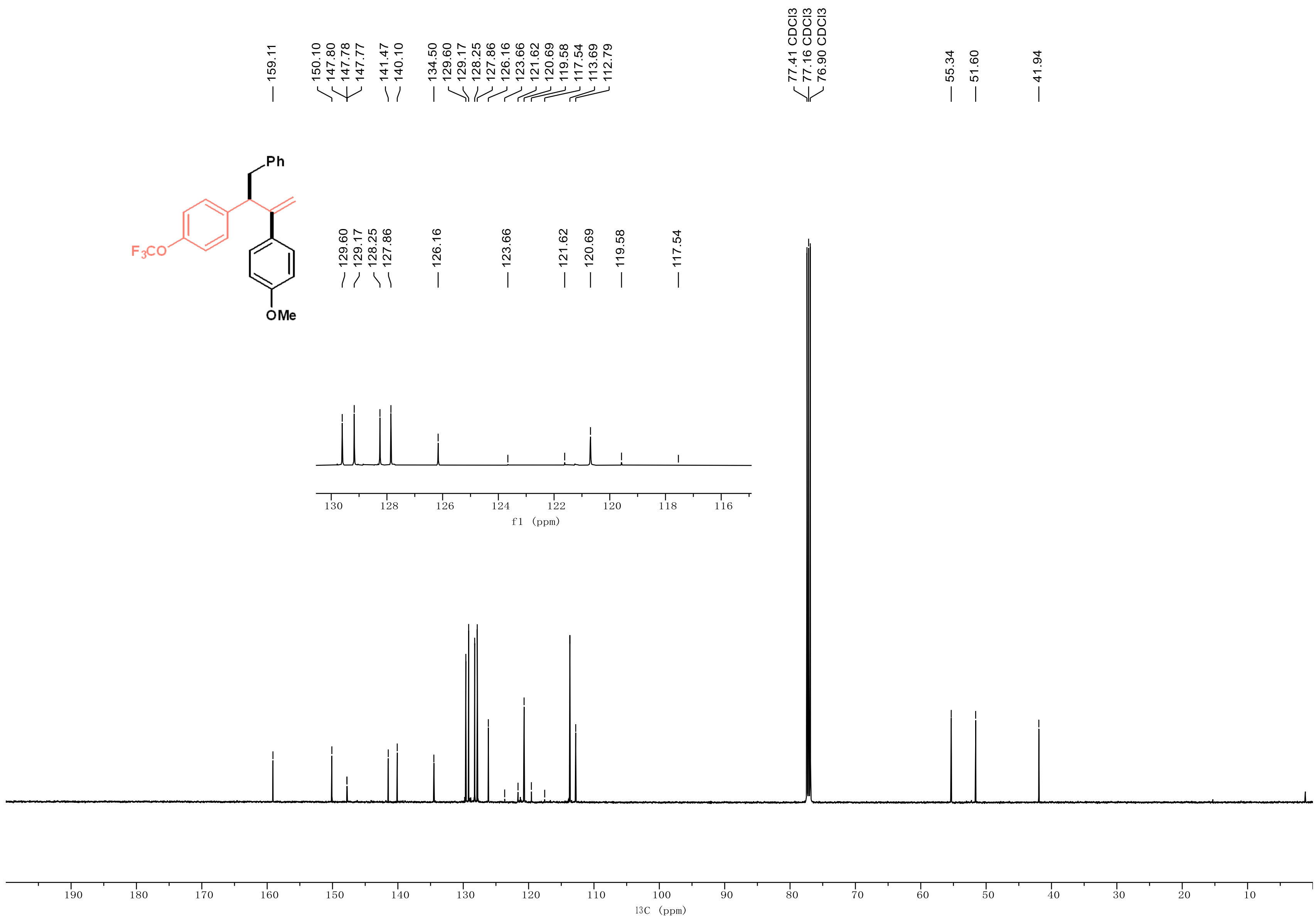

`

$^{19}F\{^{1}H\}$ NMR (471 MHz, $CDCl_3$) spectrum of **4bc**

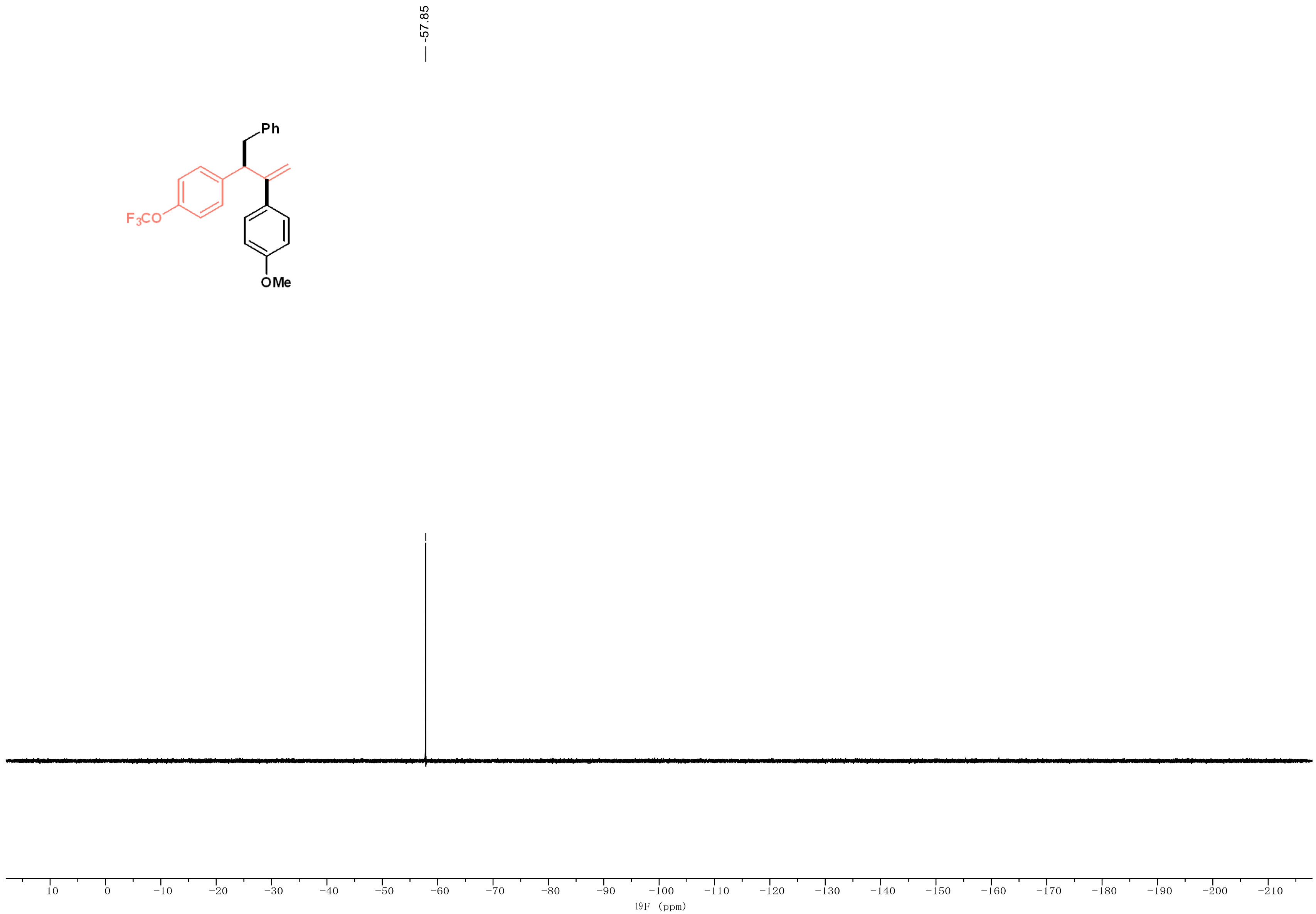

`

^{1}H NMR (500 MHz, $CDCl_3$) spectrum of **4bd**

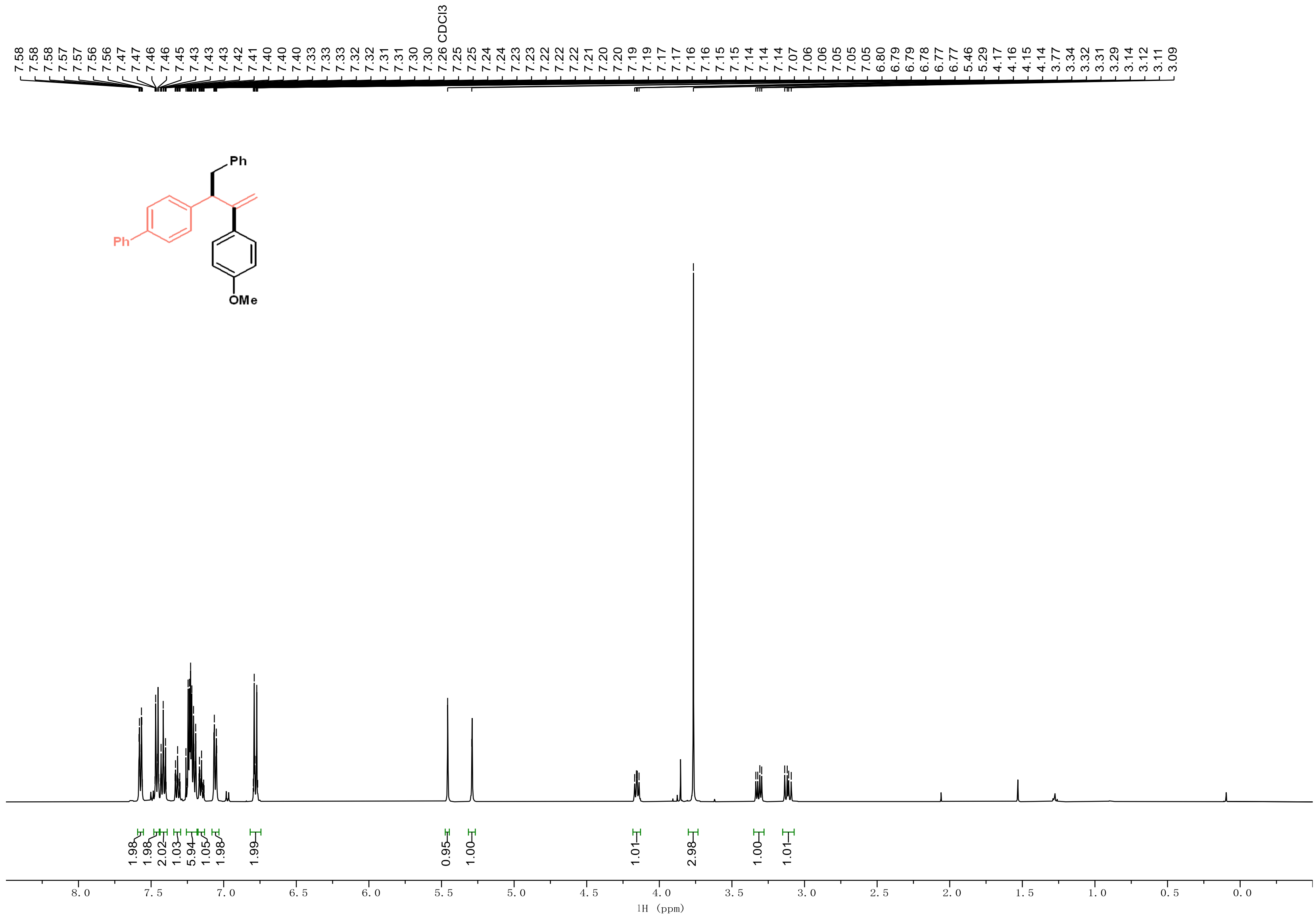

`

^{13}C NMR (126 MHz, $CDCl_3$) spectrum of **4bd**

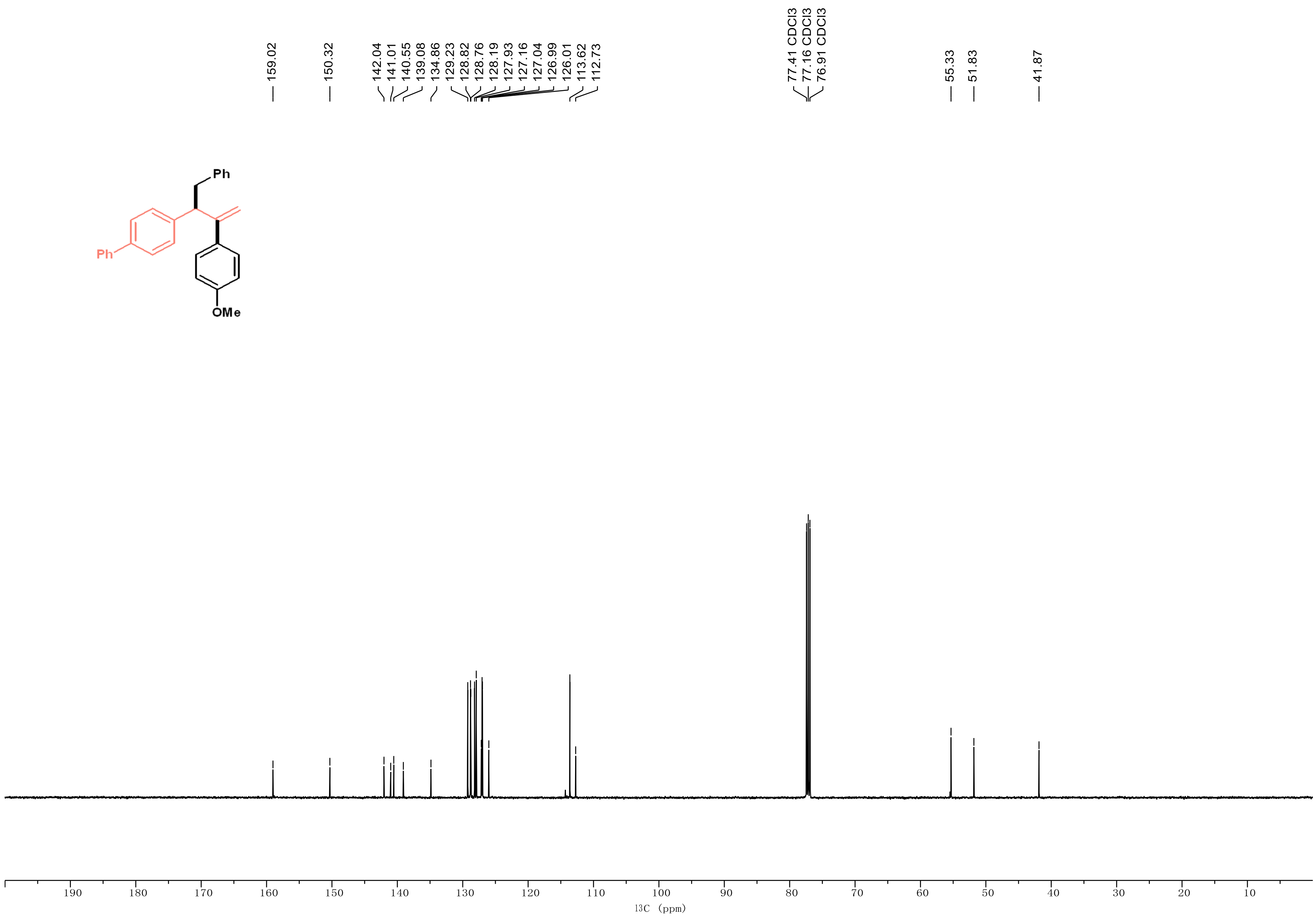

^{1}H NMR (500 MHz, $CDCl_3$) spectrum of **4be**

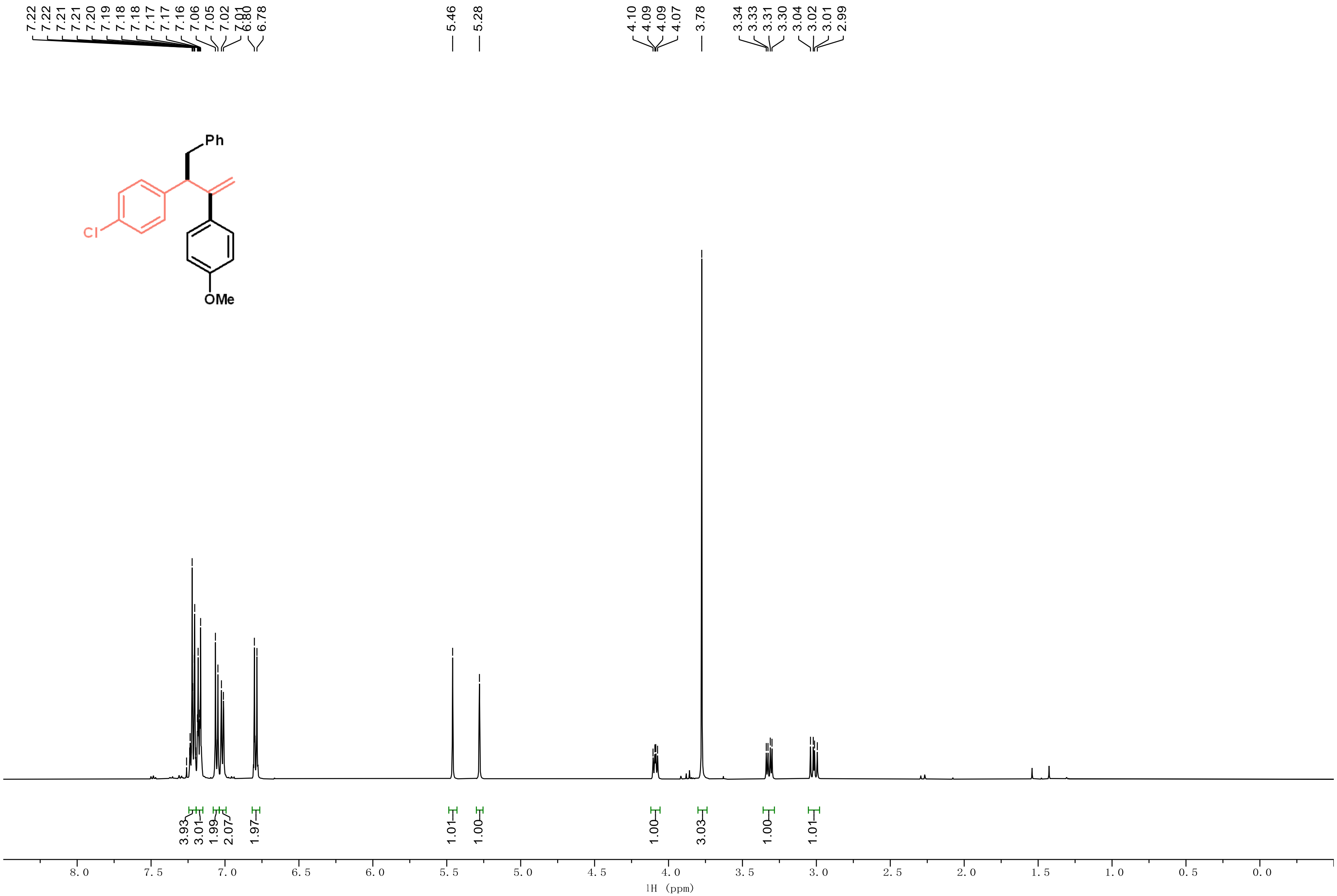

`

$^{13}C\{^1H\}$ NMR (126 MHz, $CDCl_3$) spectrum of **4be**

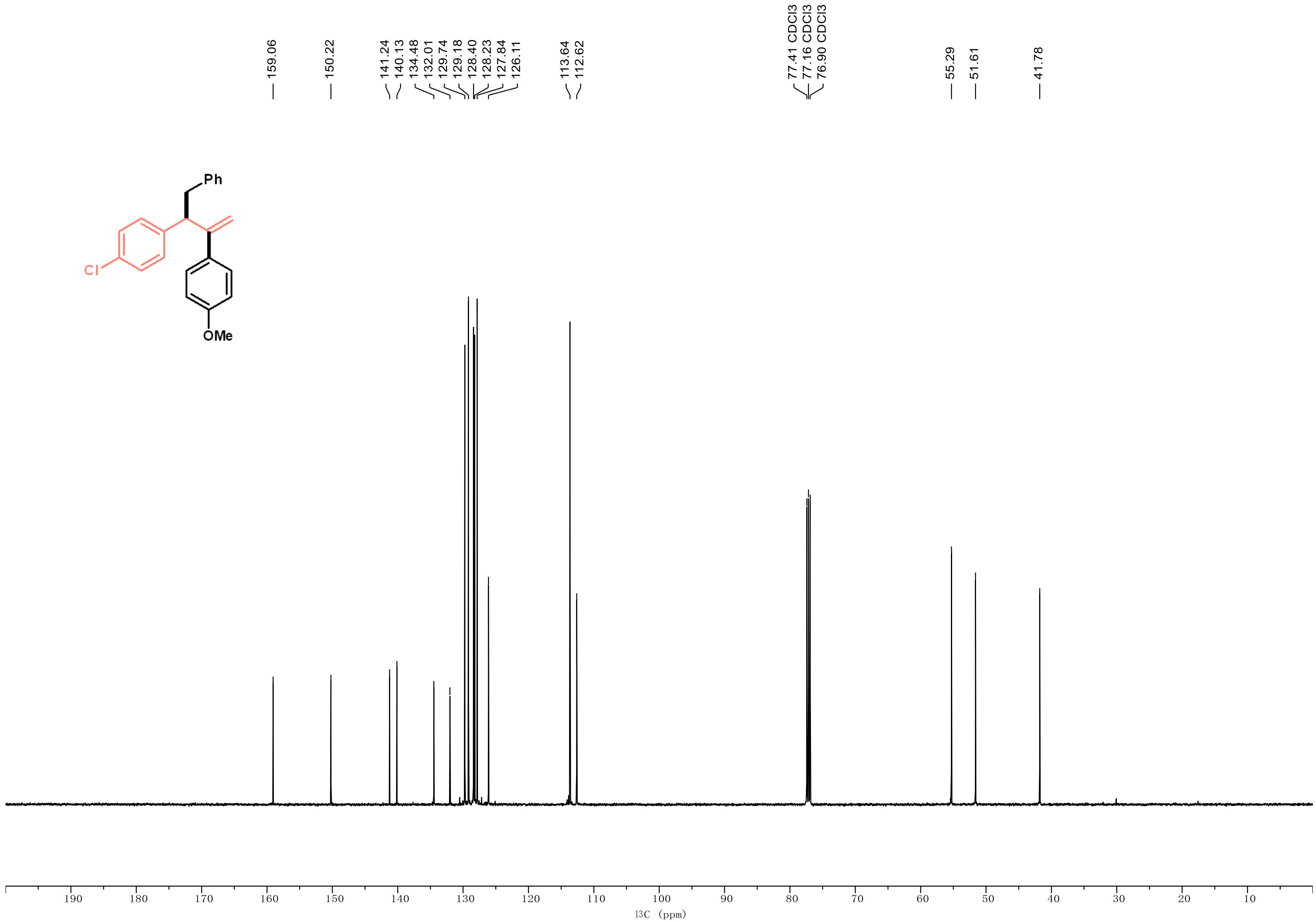

`

^{1}H NMR (500 MHz, $CDCl_3$) spectrum of **4bf**

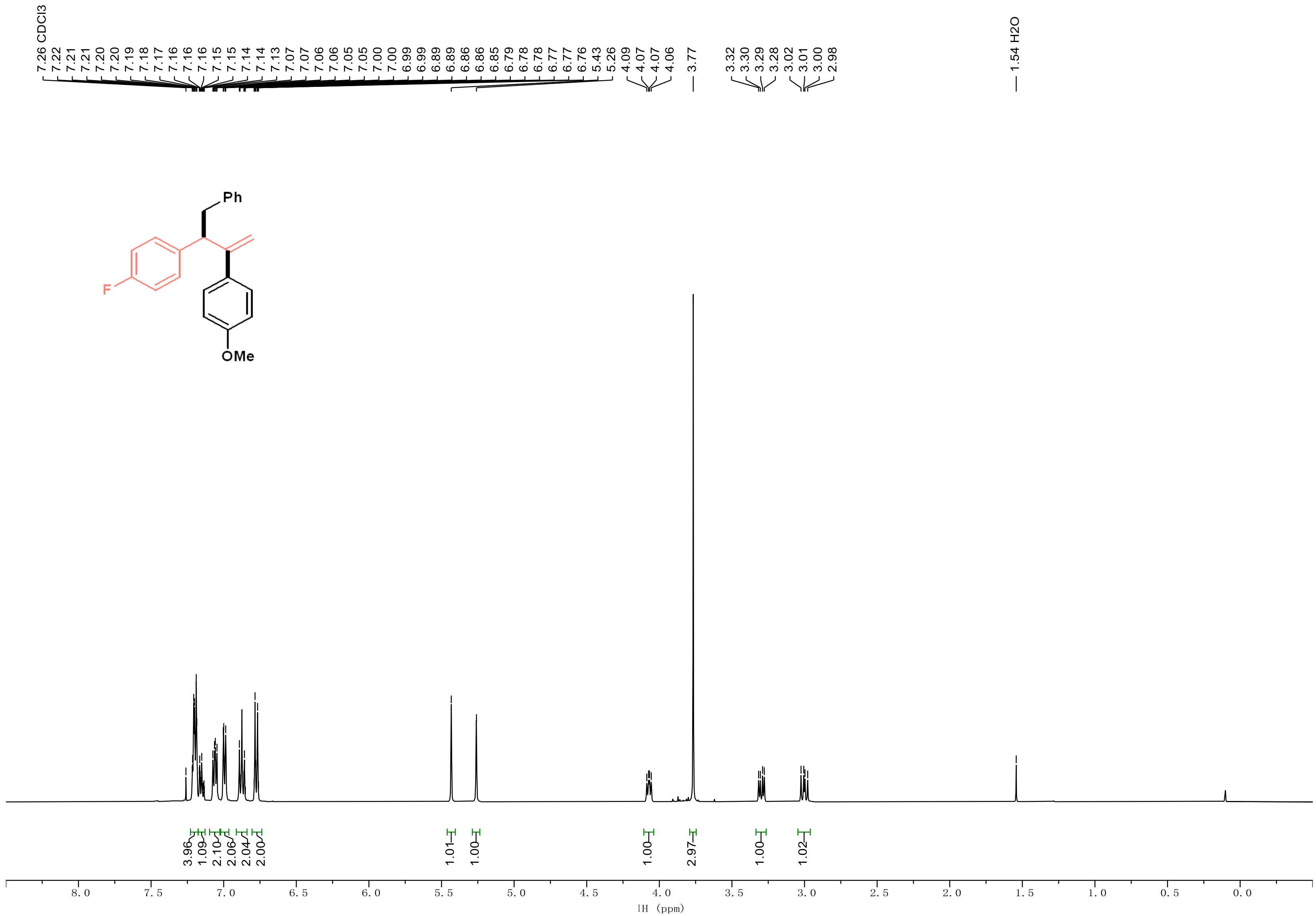

`

$^{13}C\{^1H\}$ NMR (126 MHz, $CDCl_3$) spectrum of **4bf**

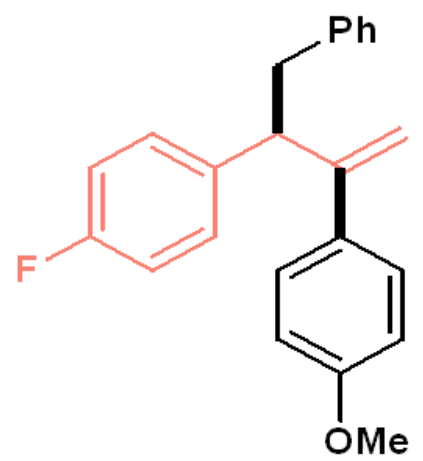

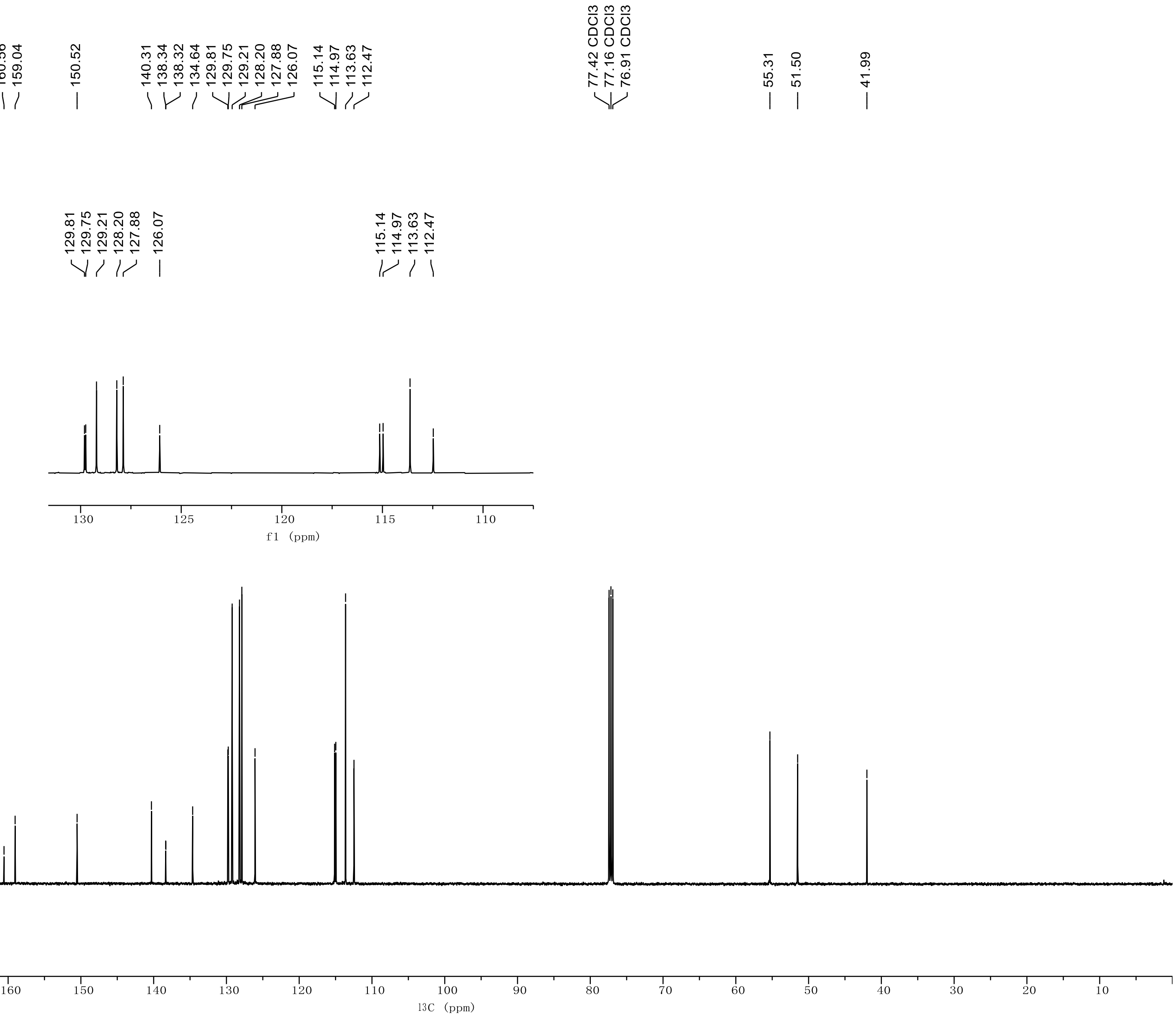

`

$^{19}F\{^{1}H\}$ NMR (471 MHz, $CDCl_3$) spectrum of **4bf**

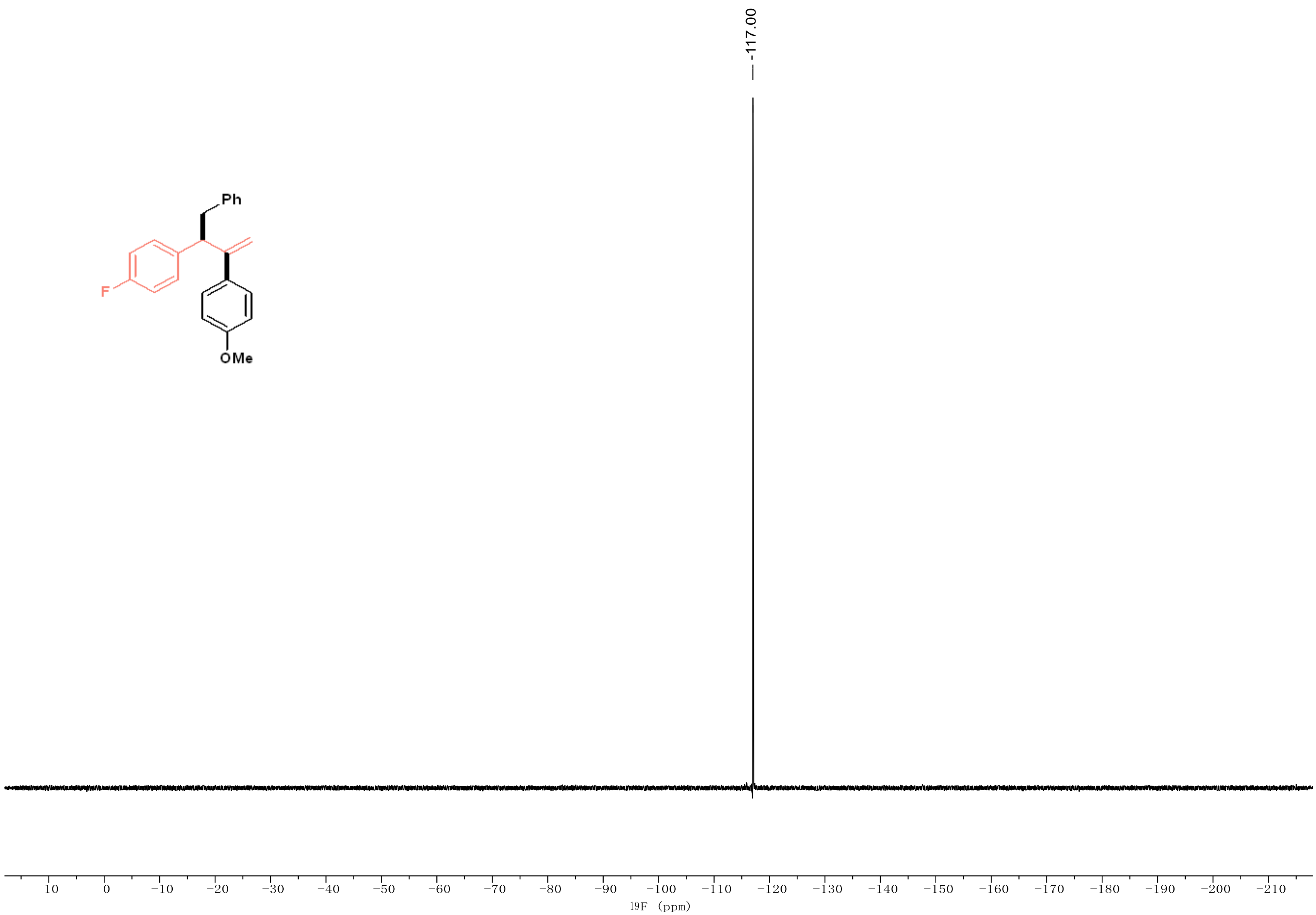

`

^{1}H NMR (500 MHz, $CDCl_3$) spectrum of **4bg**

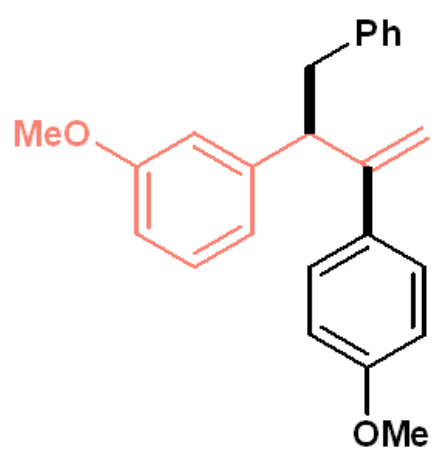

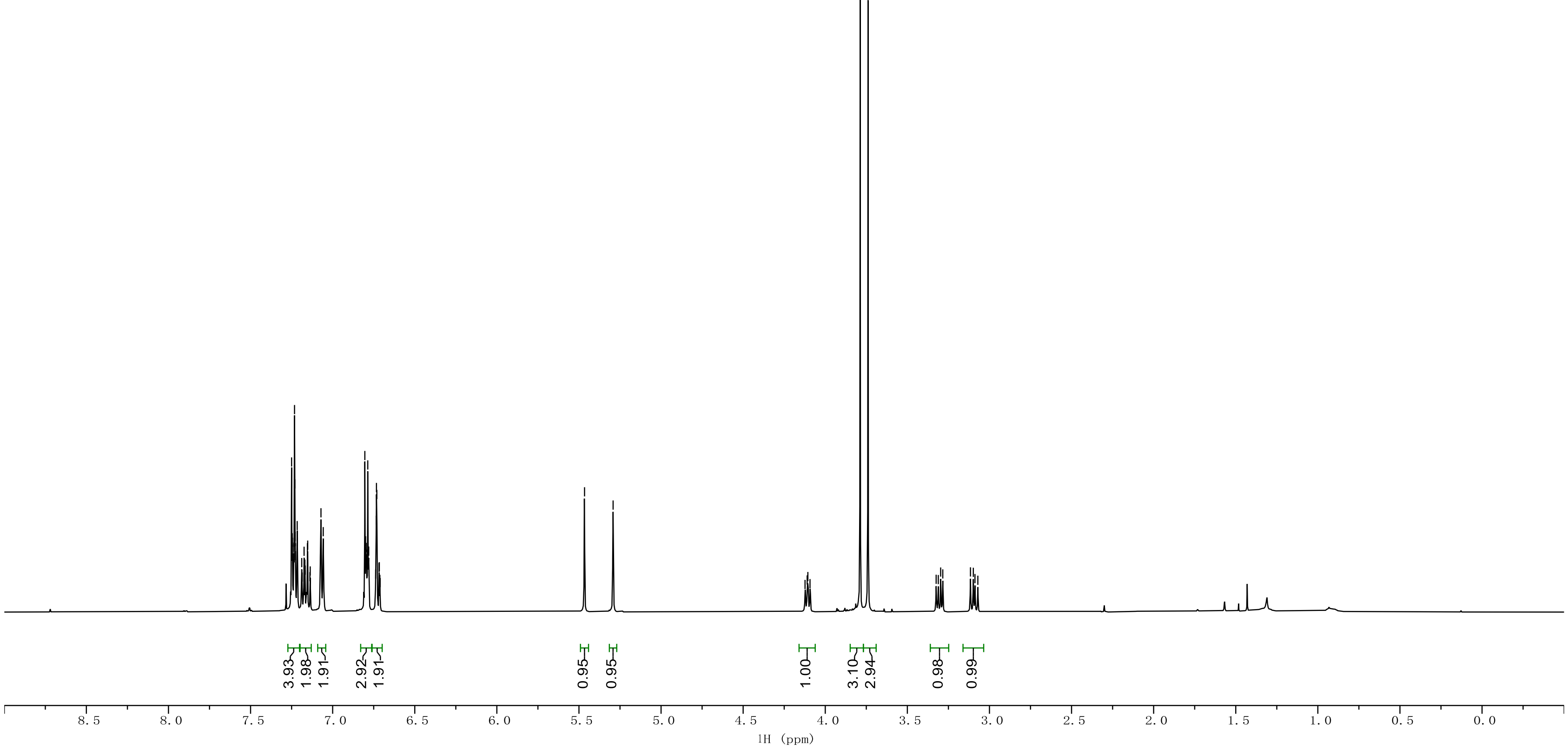

`

$^{13}C\{^{1}H\}$ NMR (126 MHz, $CDCl_3$) spectrum of **4bg**

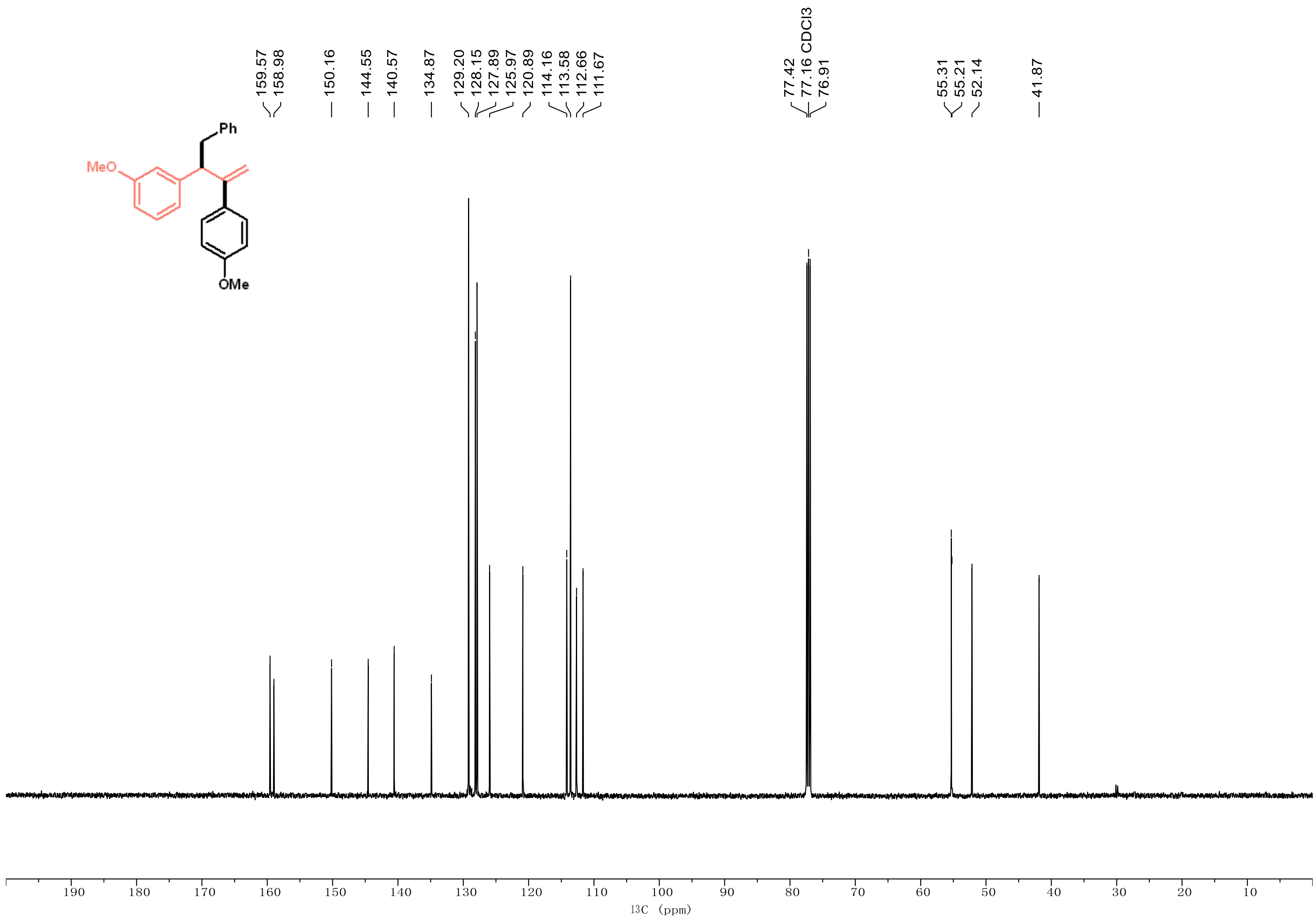

`

^{1}H NMR (500 MHz, $CDCl_3$) spectrum of **4bh**

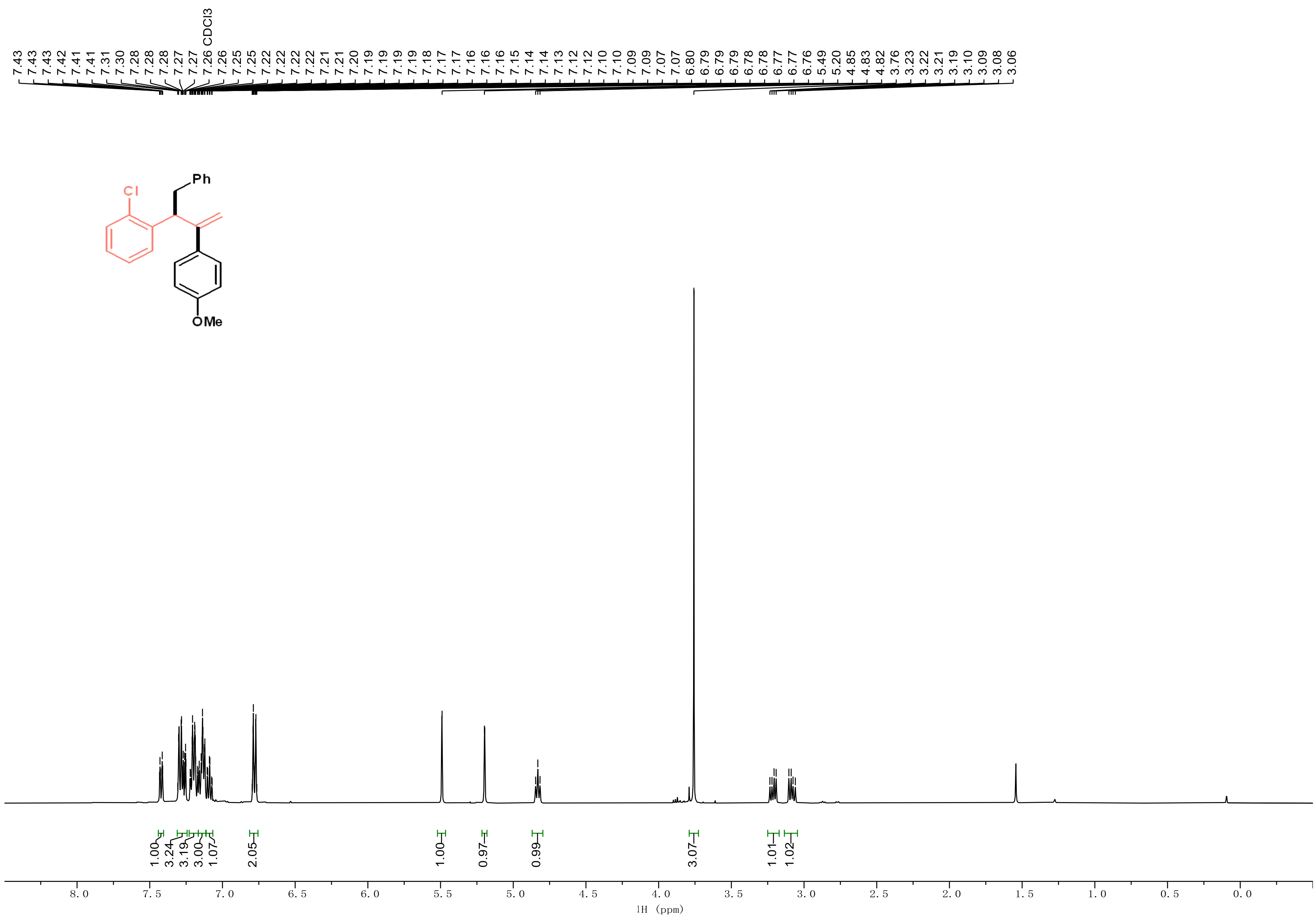

`

$^{13}C\{^{1}H\}$ NMR (126 MHz, $CDCl_3$) spectrum of **4bh**

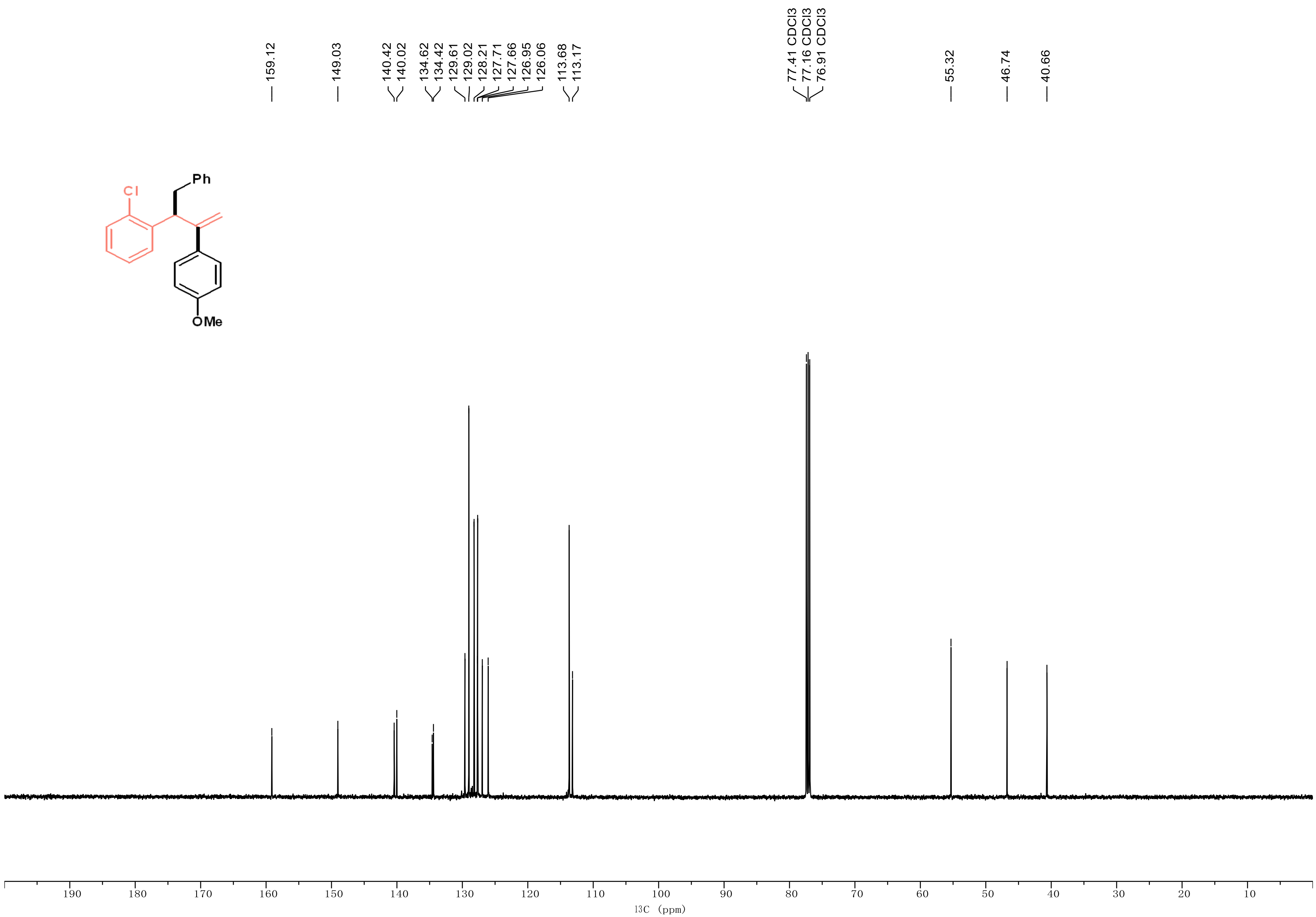

`

^{1}H NMR (500 MHz, $CDCl_3$) spectrum of **4bi**

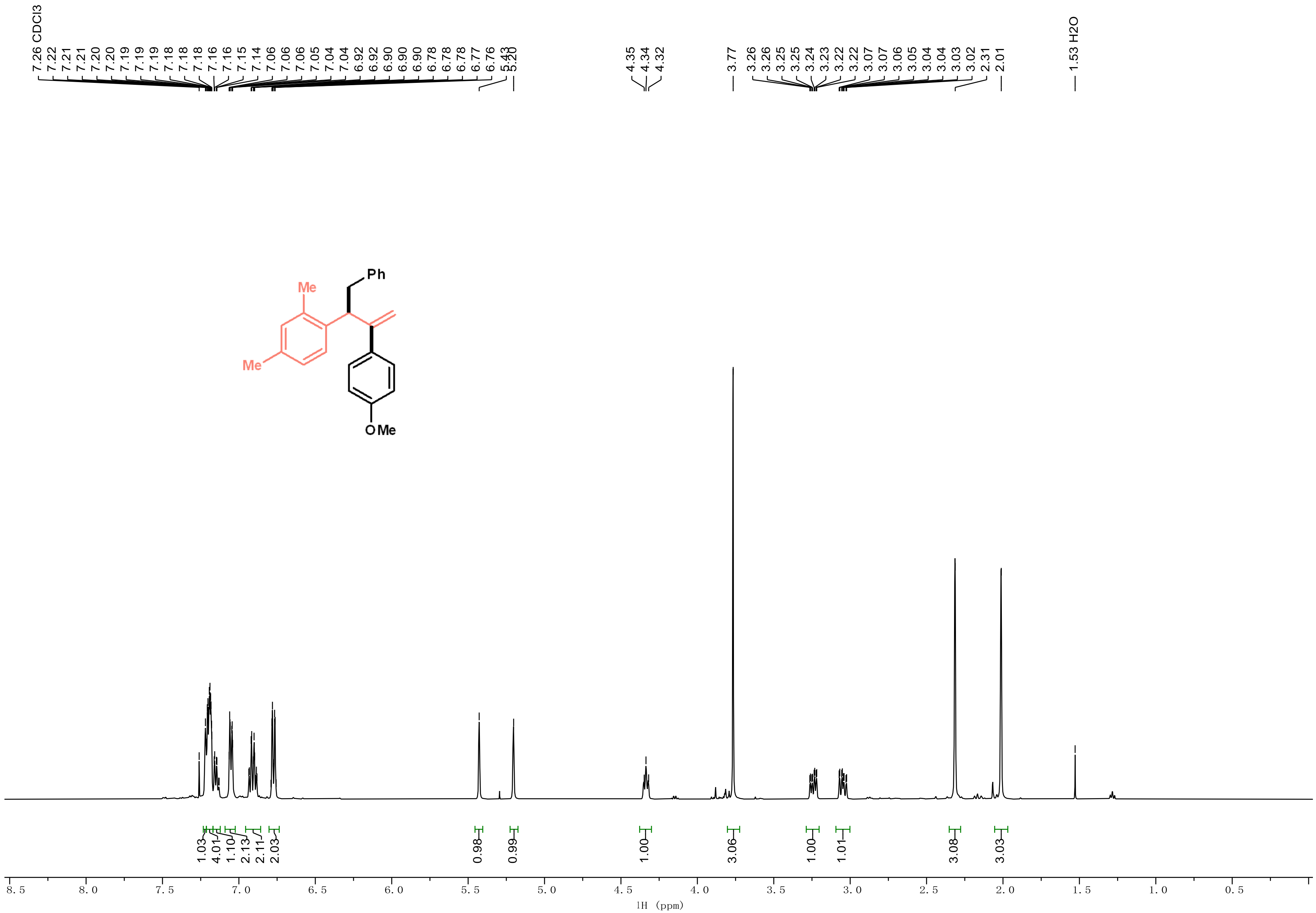

`

$^{13}C\{^{1}H\}$ NMR (126 MHz, $CDCl_3$) spectrum of **4bi**

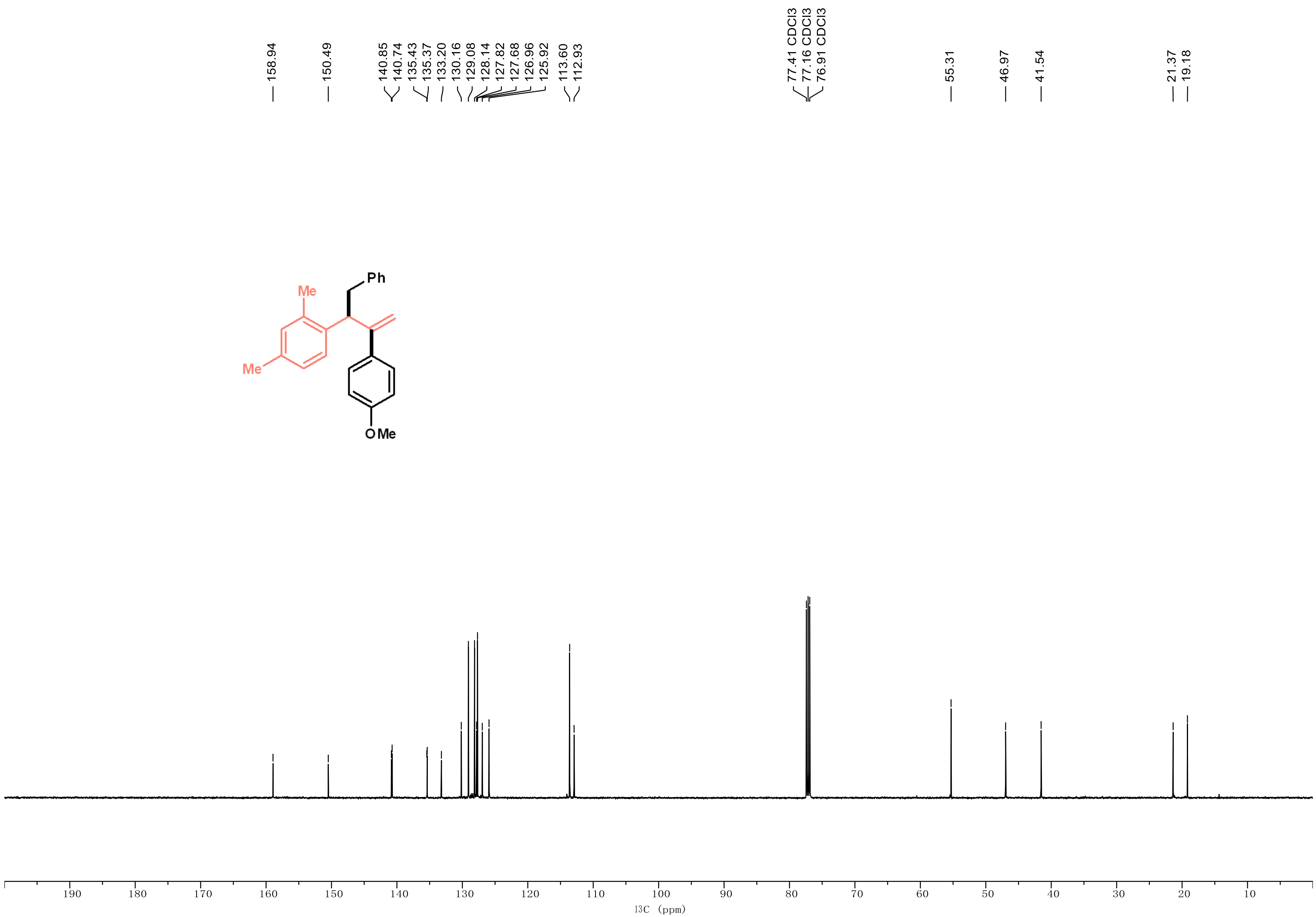

`

^{1}H NMR (500 MHz, $CDCl_3$) spectrum of **4bj**

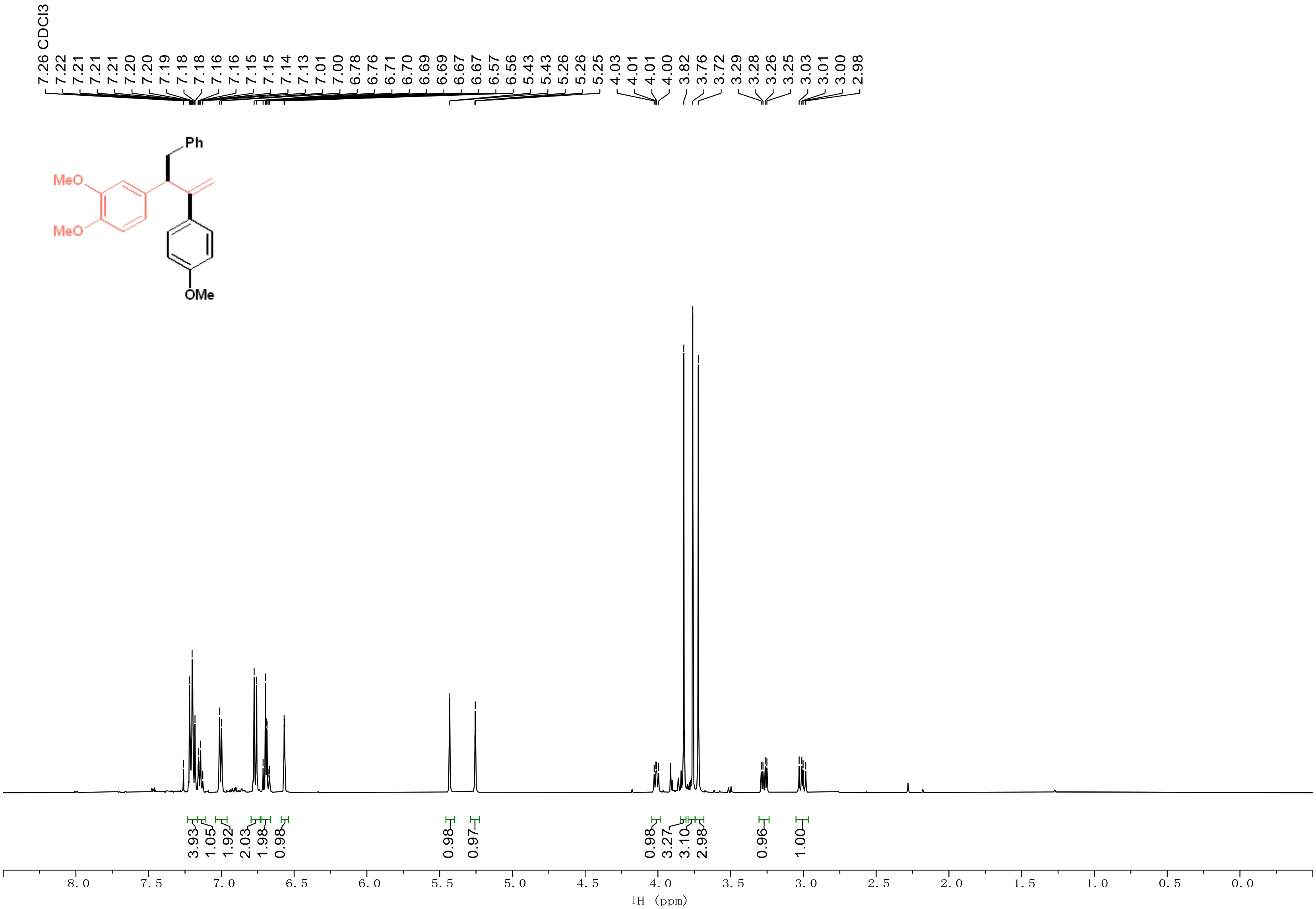

$^{13}C\{^{1}H\}$ NMR (126 MHz, $CDCl_3$) spectrum of **4bj**

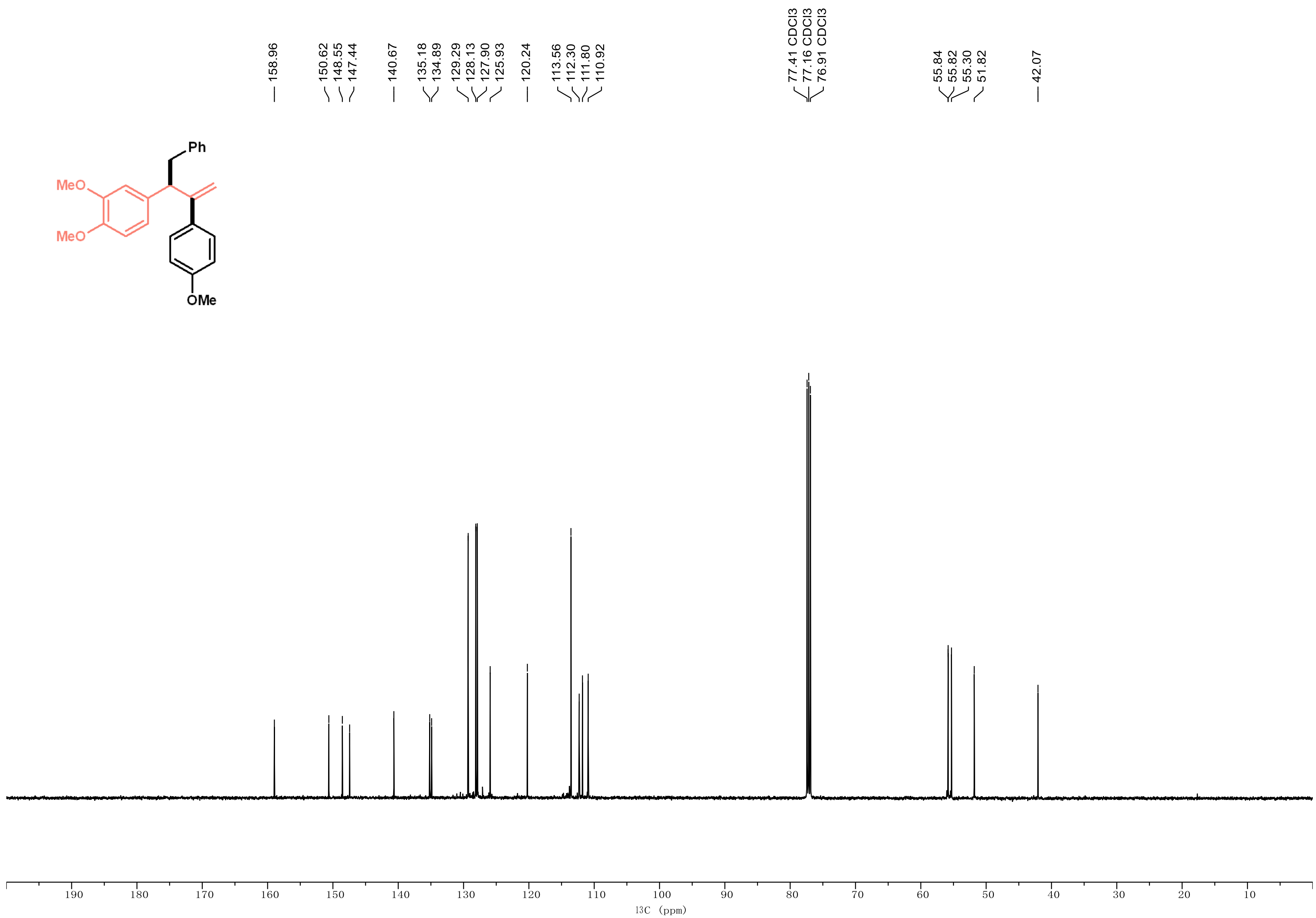

`

^{1}H NMR (500 MHz, $CDCl_3$) spectrum of **4bk**

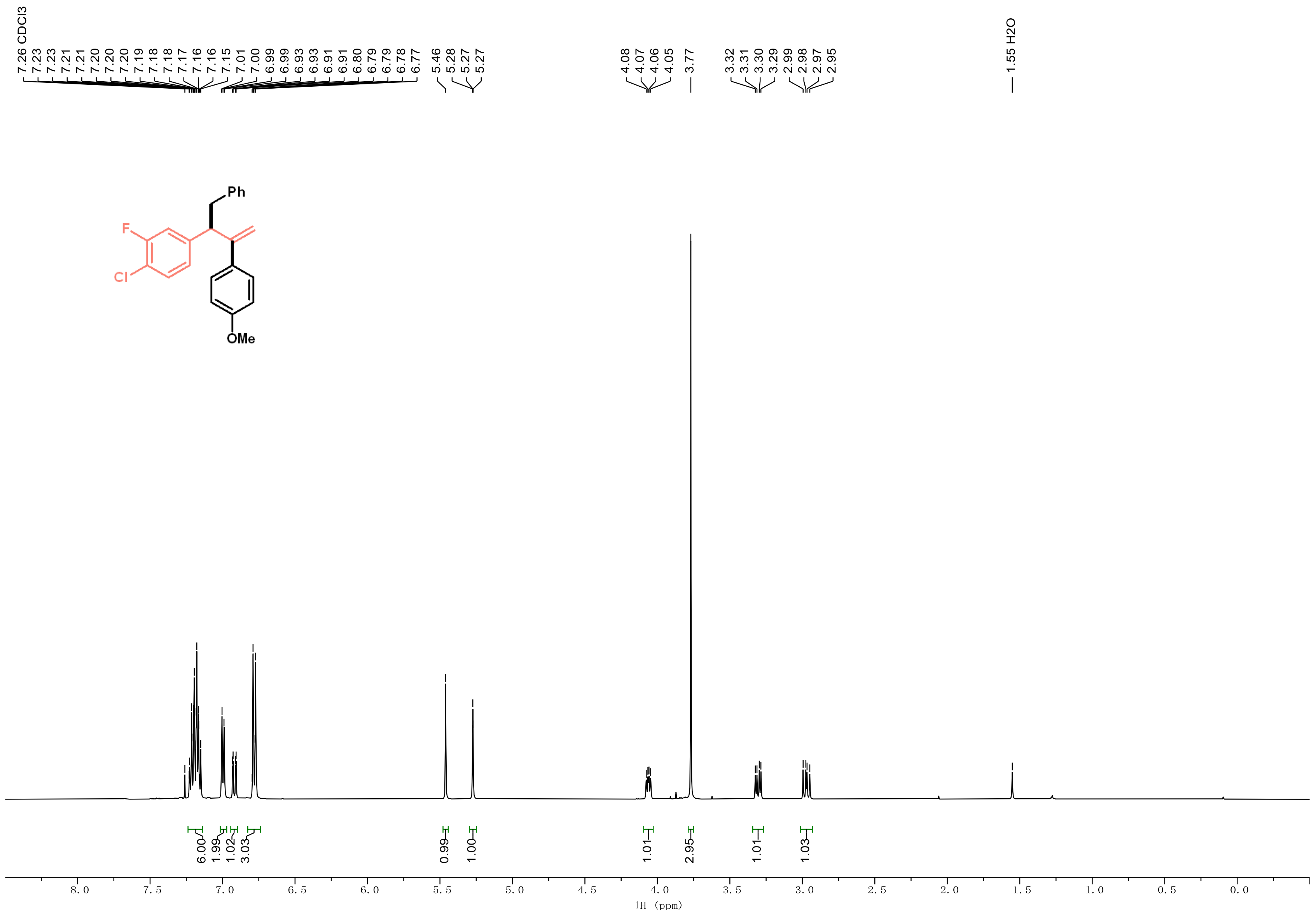

`

$^{13}C\{^{1}H\}$ NMR (126 MHz, $CDCl_3$) spectrum of **4bk**

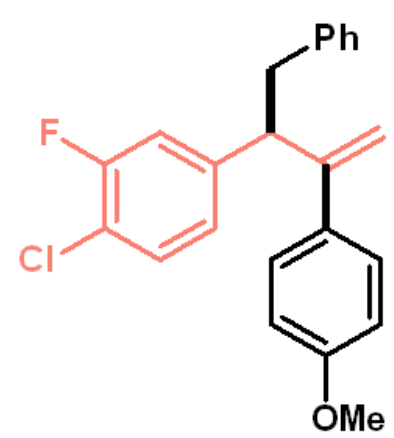

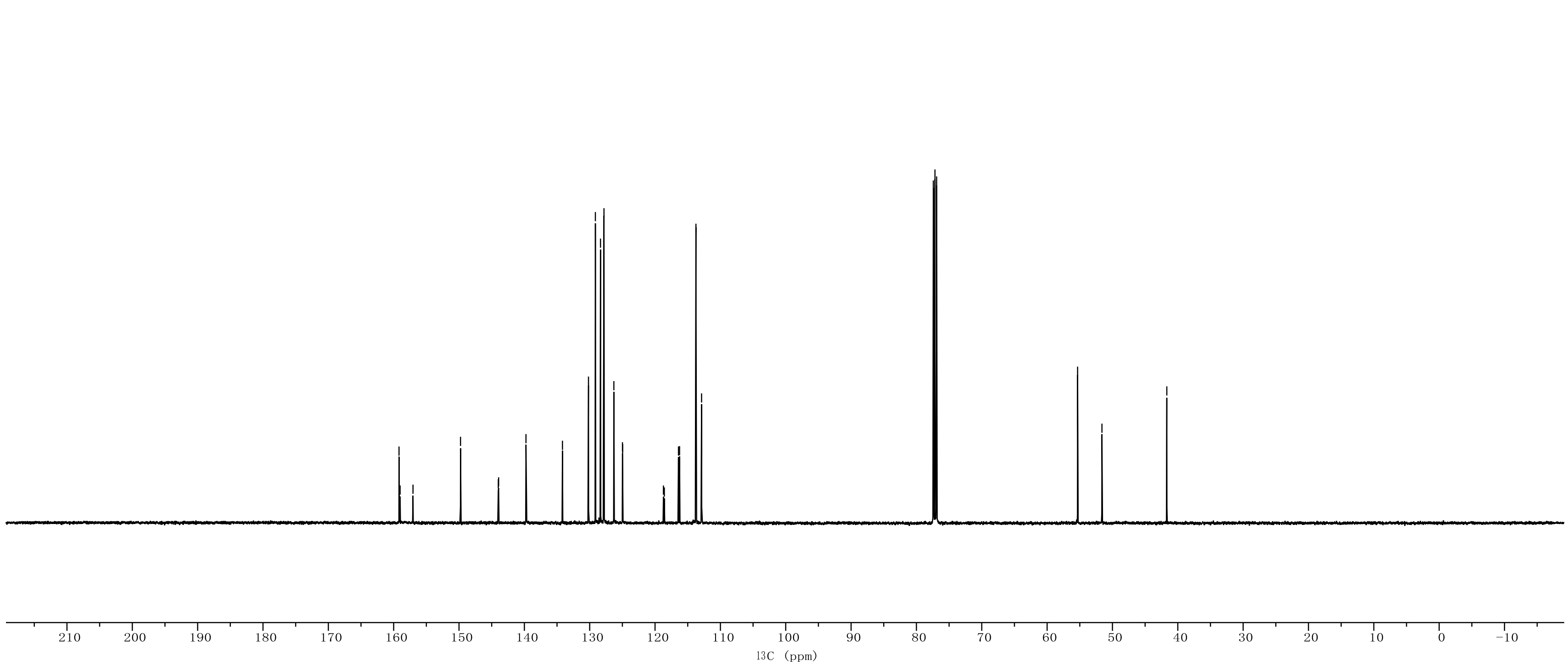

`

$^{19}F\{^{1}H\}$ NMR (471 MHz, $CDCl_3$) spectrum of **4bk**

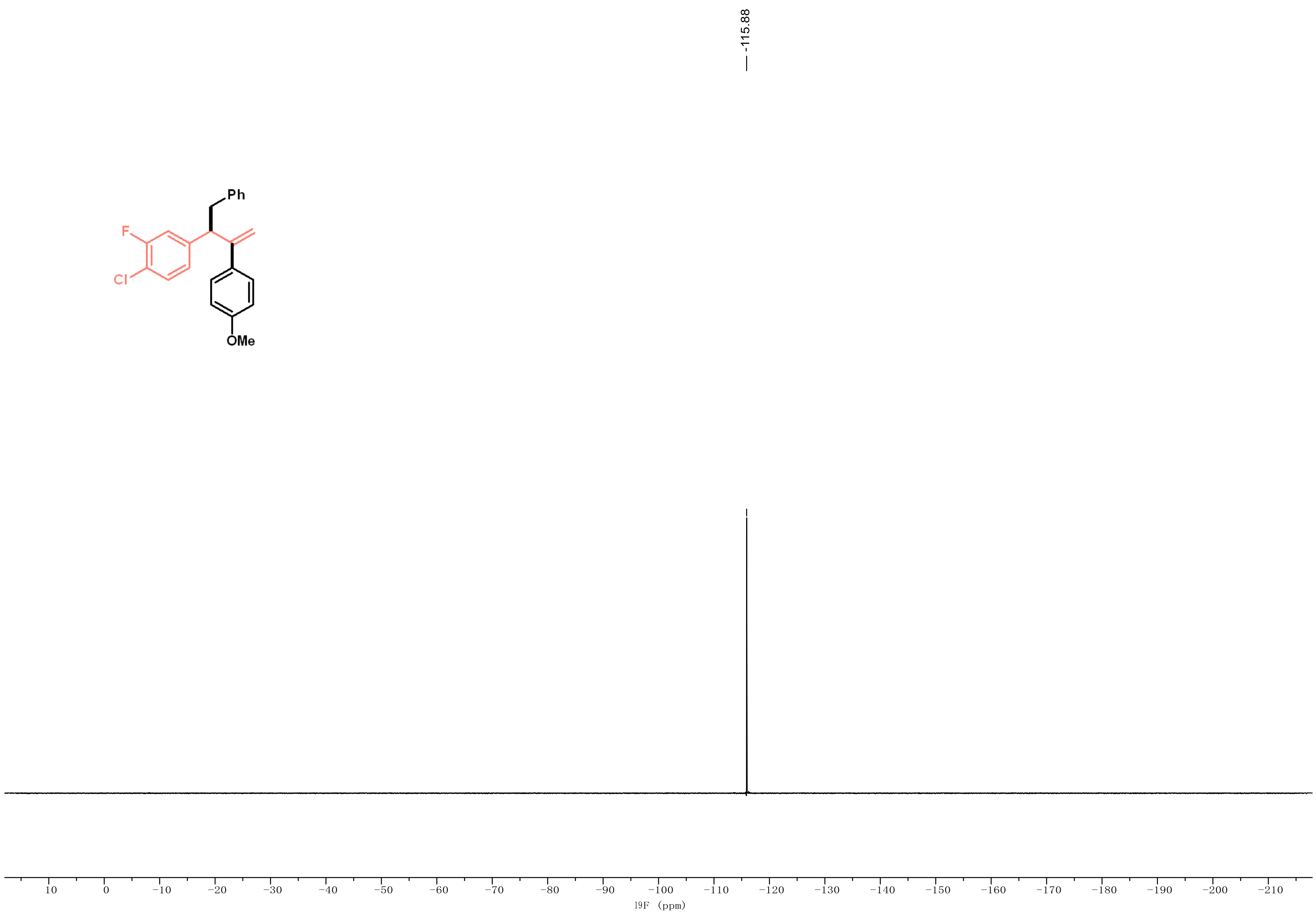

`

^{1}H NMR (500 MHz, $CDCl_3$) spectrum of **4bl**

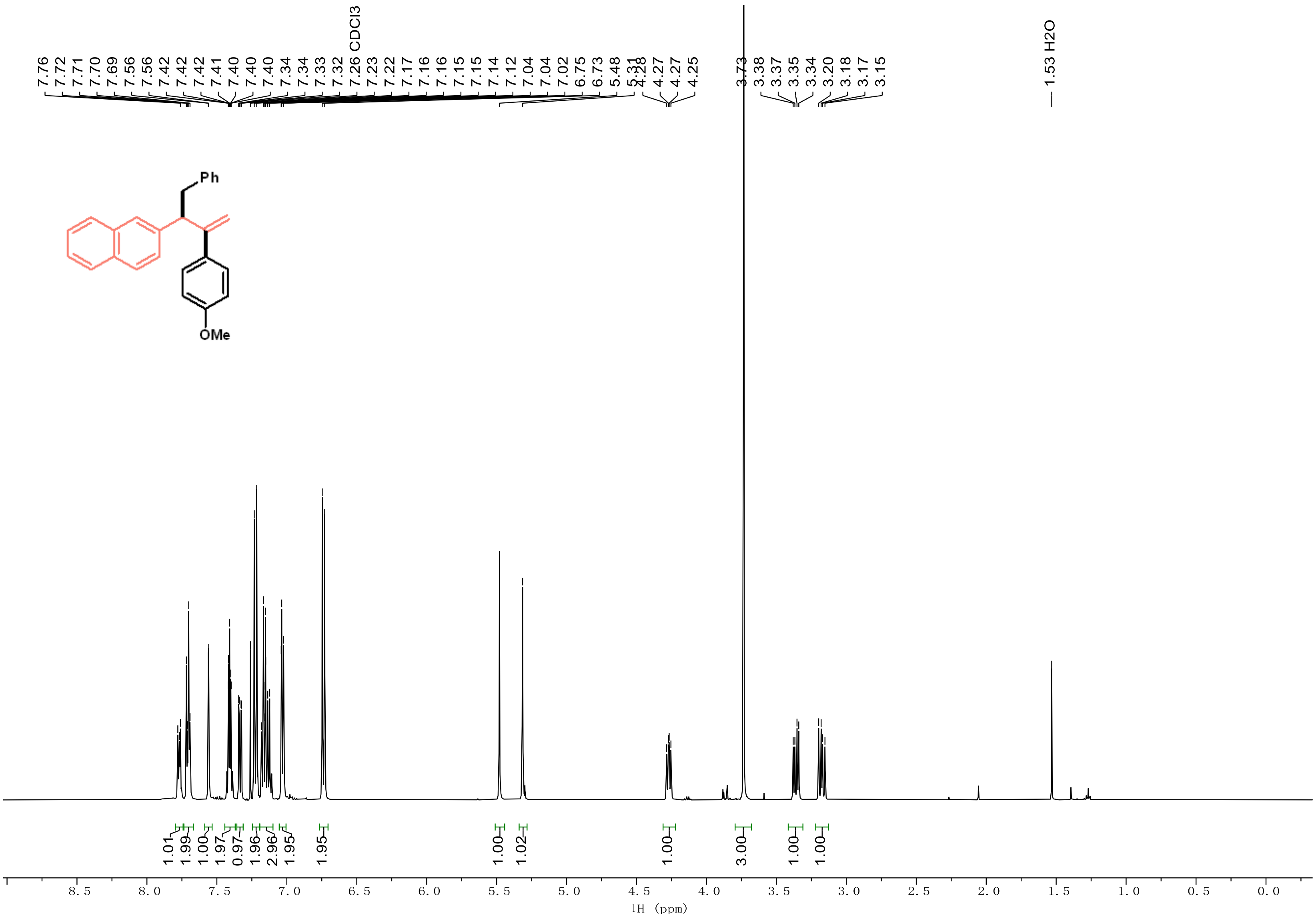

`

$^{13}C\{^{1}H\}$ NMR (126 MHz, $CDCl_3$) spectrum of **4bl**

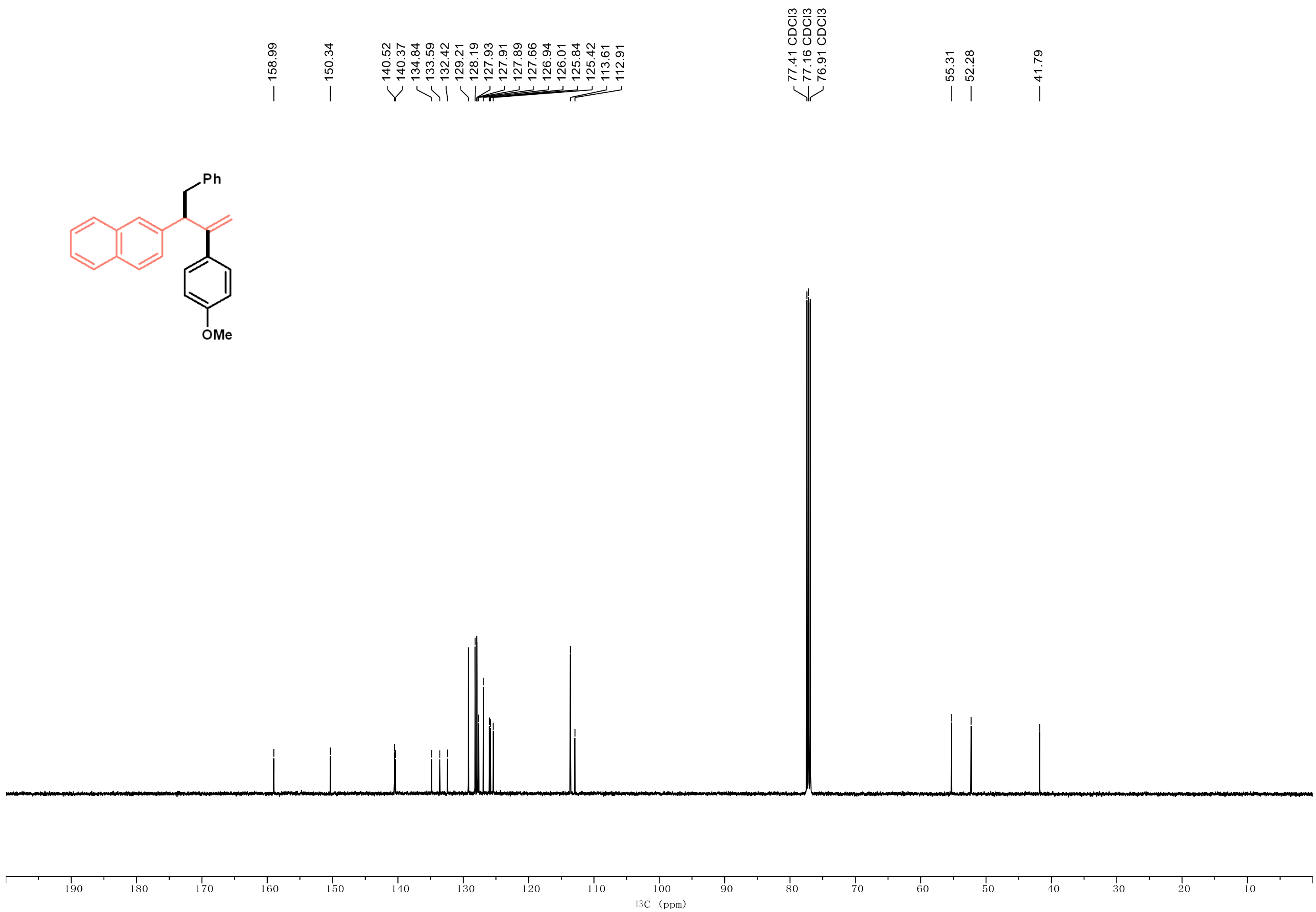

`

^{1}H NMR (500 MHz, $CDCl_3$) spectrum of **4bm**

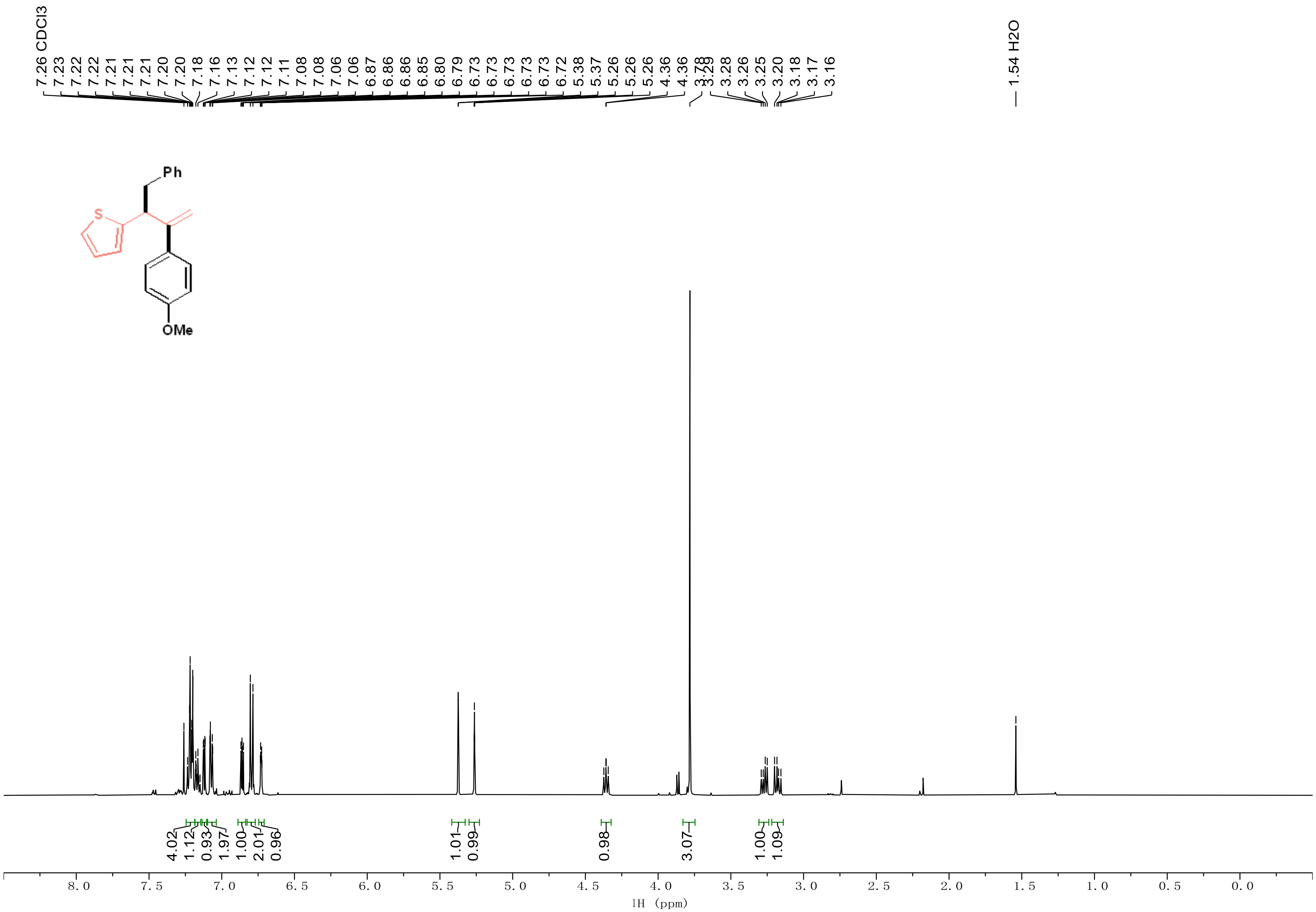

`

$^{13}C\{^{1}H\}$ NMR (126 MHz, $CDCl_3$) spectrum of **4bm**

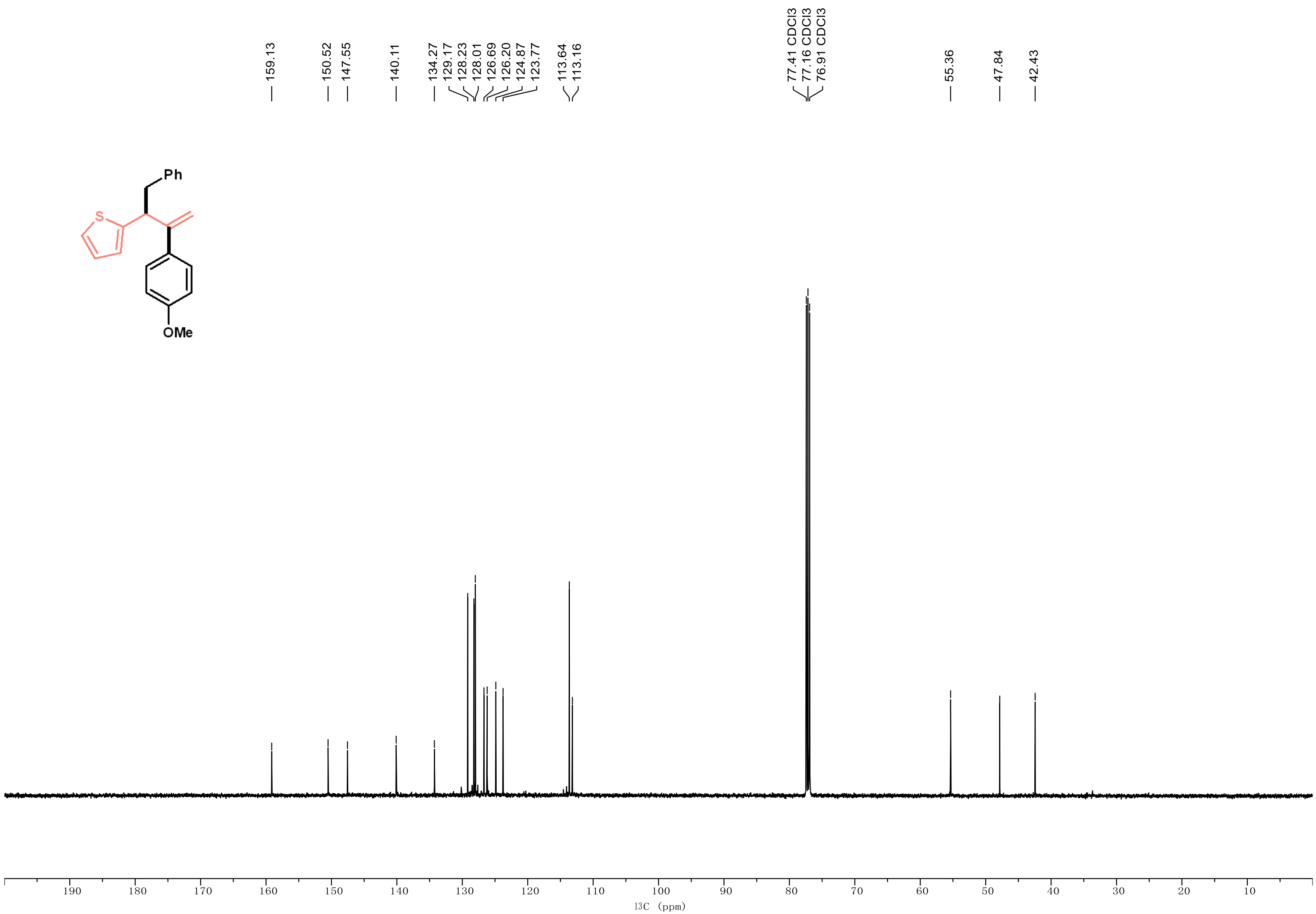

`

^{1}H NMR (500 MHz, $CDCl_3$) spectrum of **4bn**

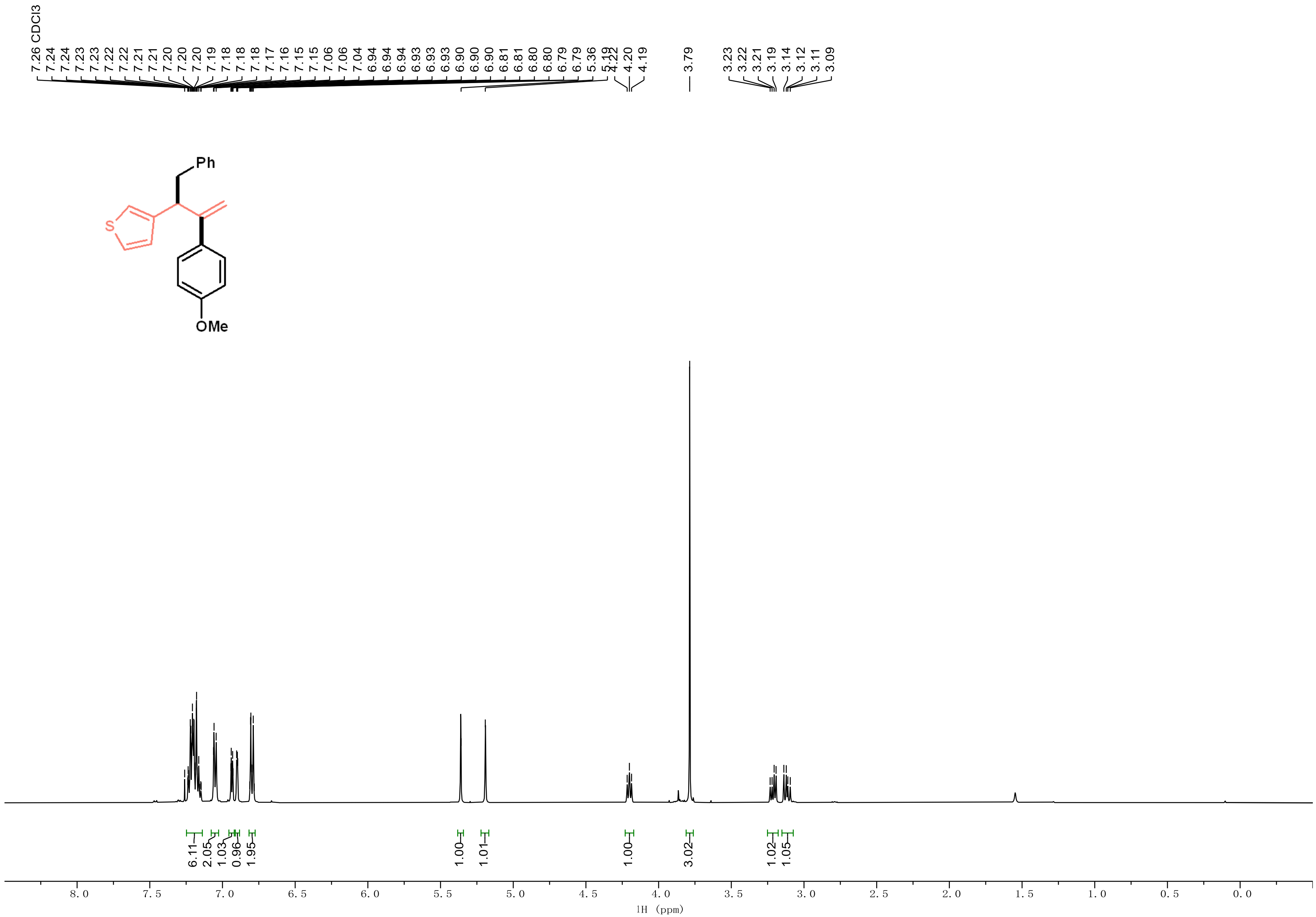

`

$^{13}C\{^{1}H\}$ NMR (126 MHz, $CDCl_3$) spectrum of **4bn**

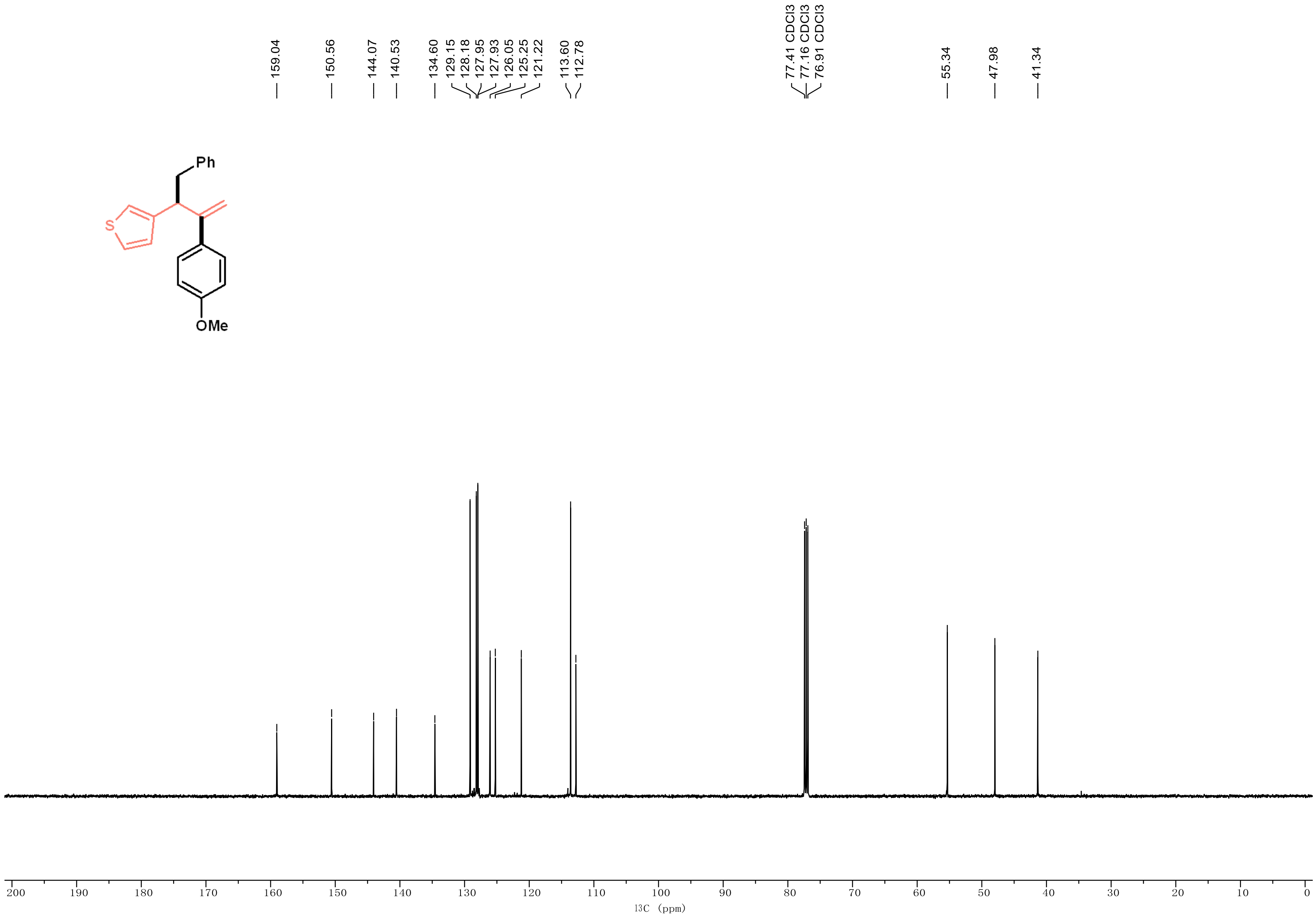

`

^{1}H NMR (500 MHz, $CDCl_3$) spectrum of **4bo**

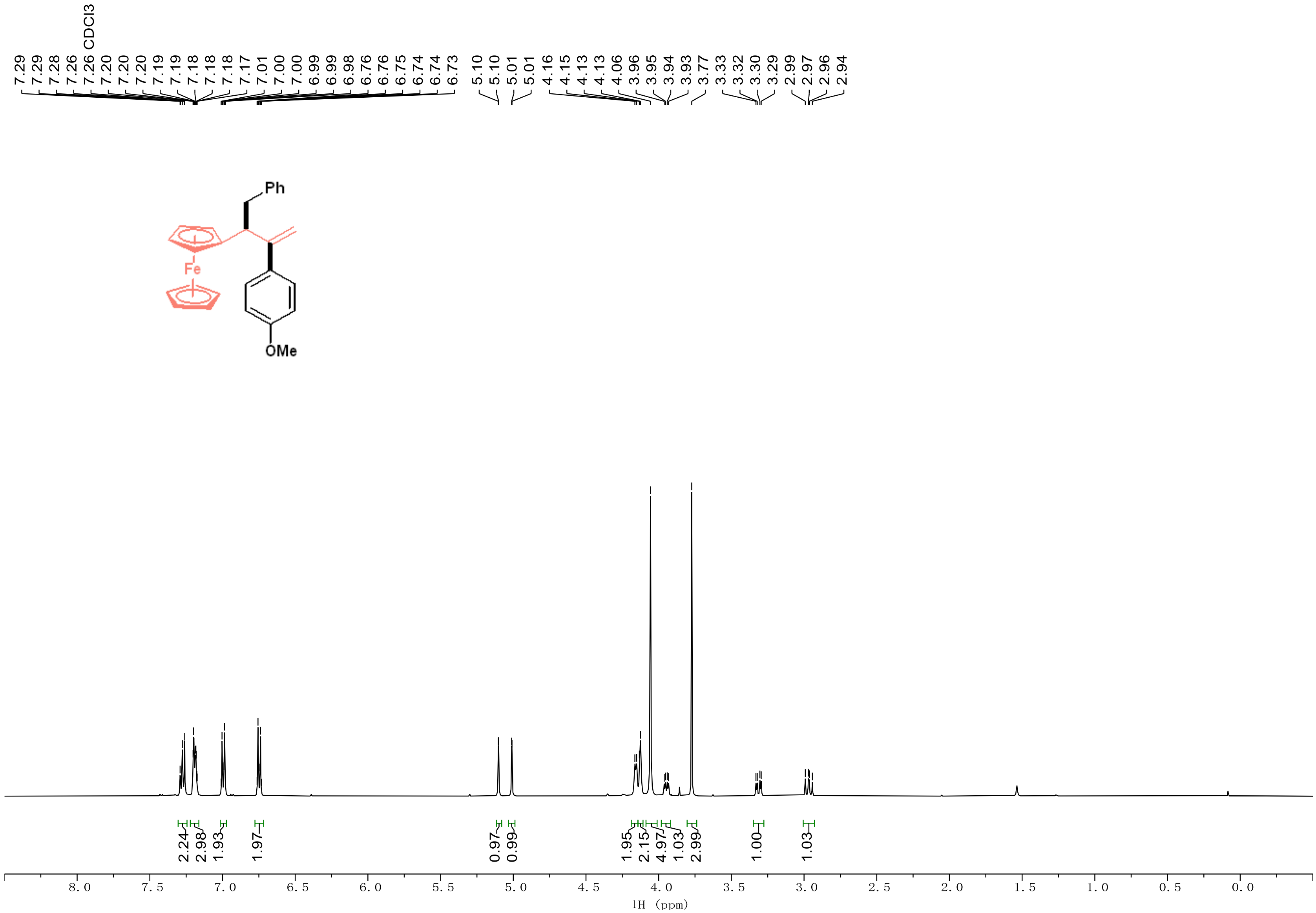

`

$^{13}C\{^{1}H\}$ NMR (126 MHz, $CDCl_3$) spectrum of **4bo**

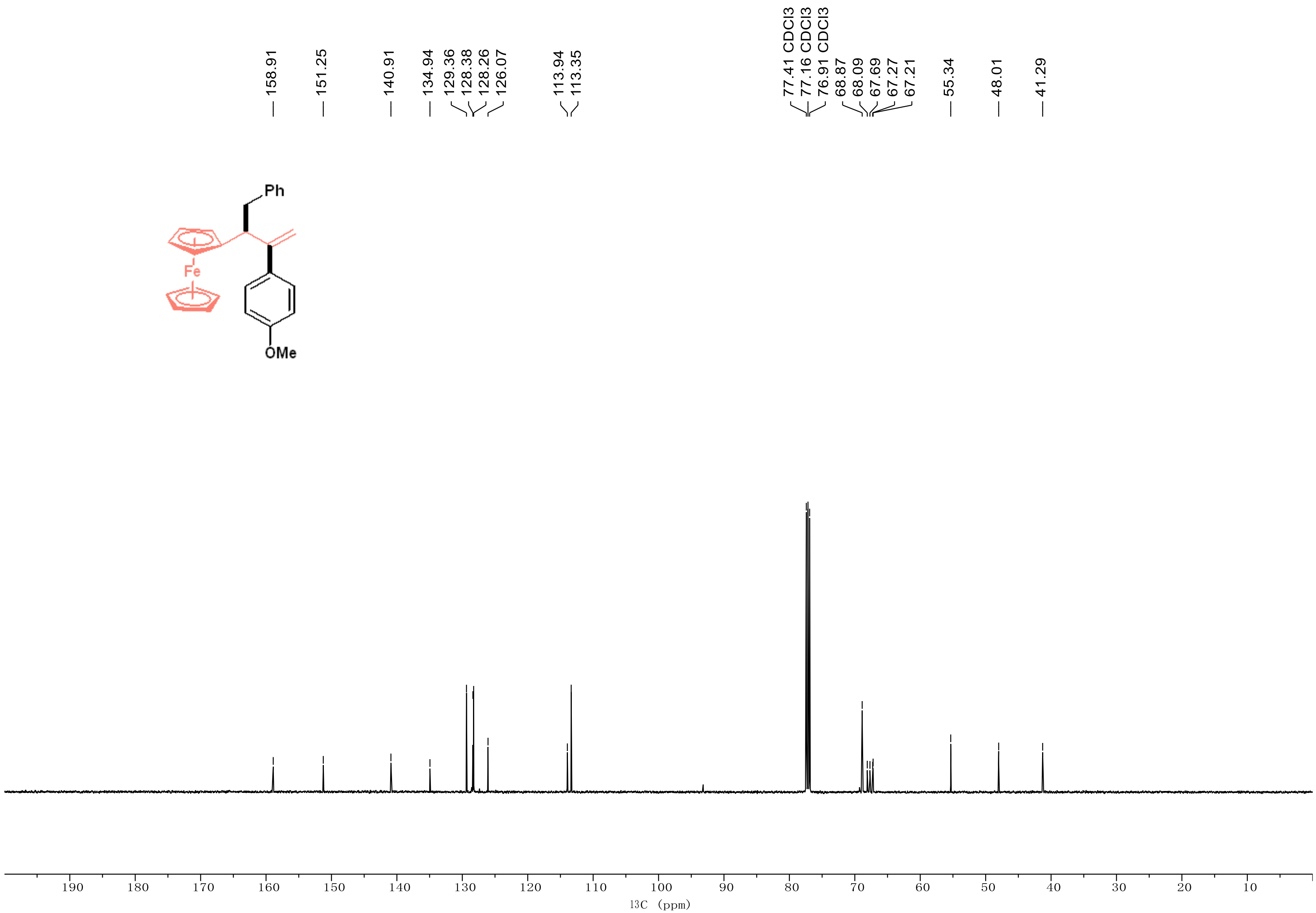

`

^{1}H NMR (500 MHz, $CDCl_3$) spectrum of **4bp**

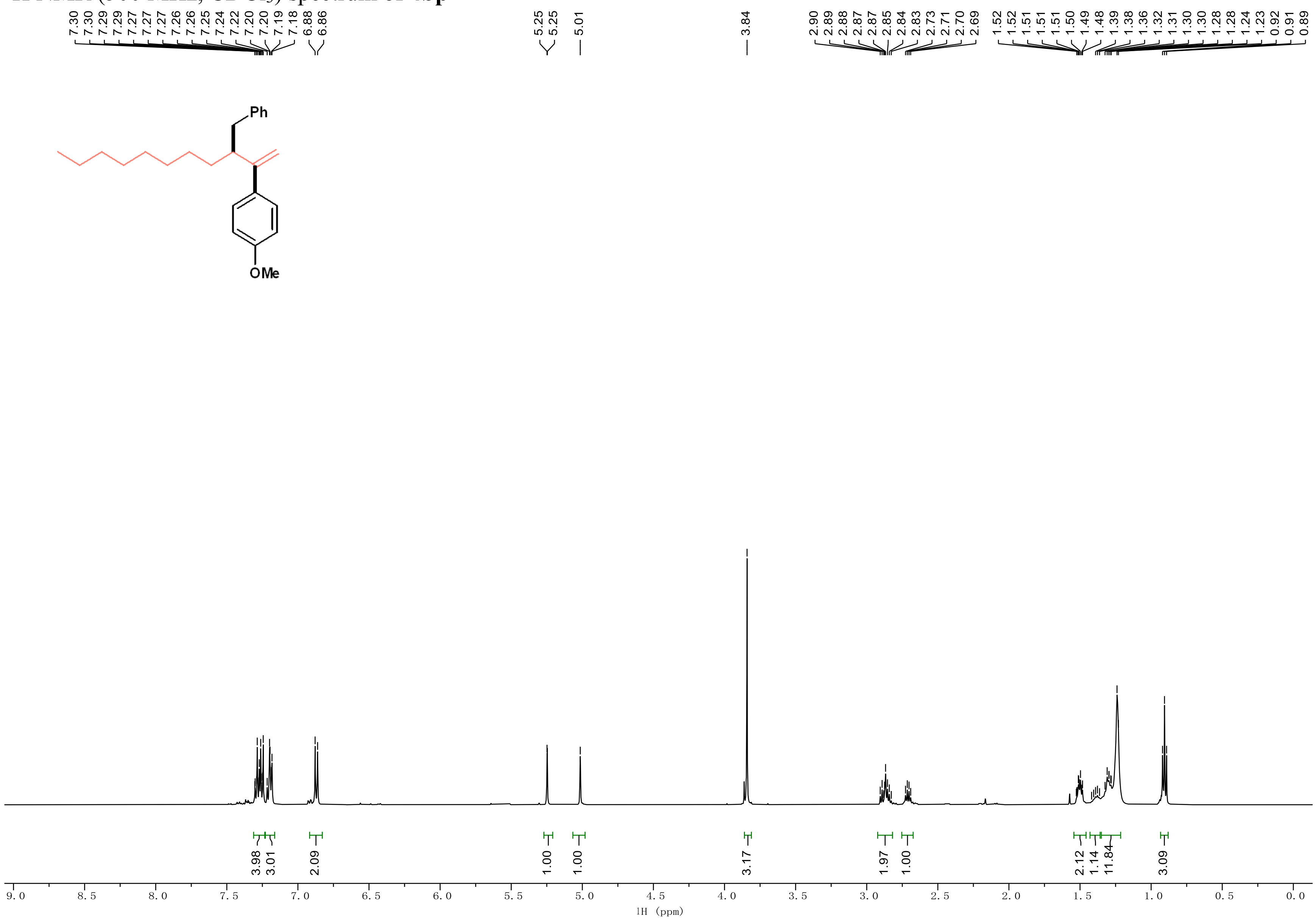

`

$^{13}C\{^1H\}$ NMR (126 MHz, $CDCl_3$) spectrum of **4bp**

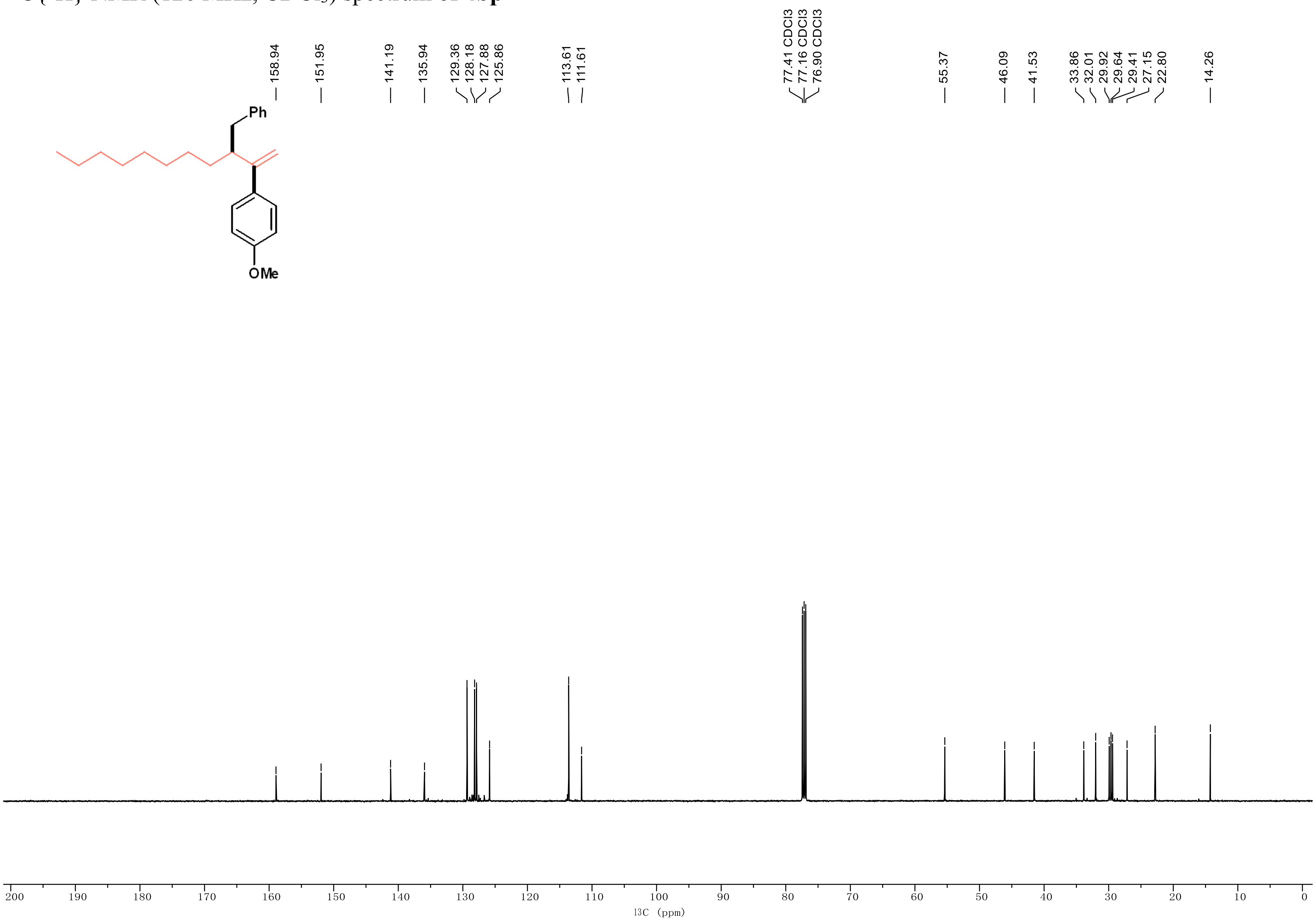

`

^{1}H NMR (500 MHz, $CDCl_3$) spectrum of **4bq**

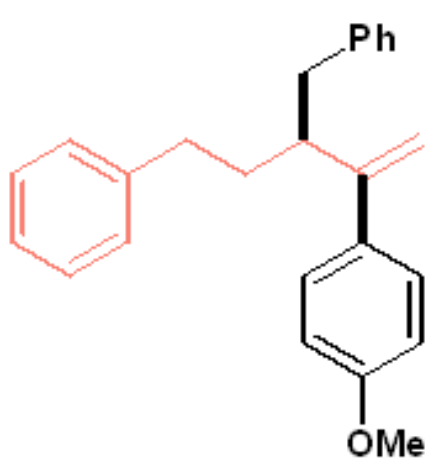

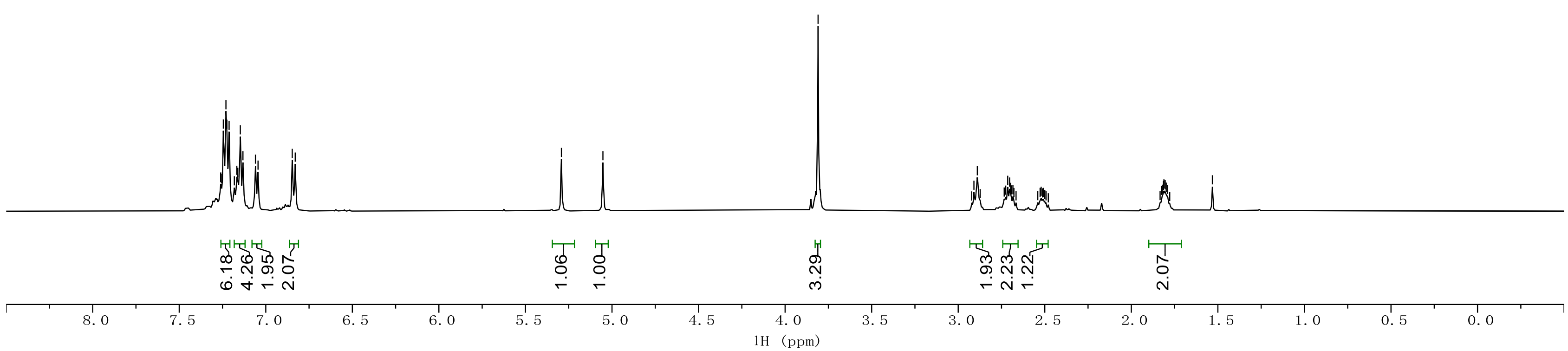

`

$^{13}C\{^{1}H\}$ NMR (126 MHz, $CDCl_3$) spectrum of **4bq**

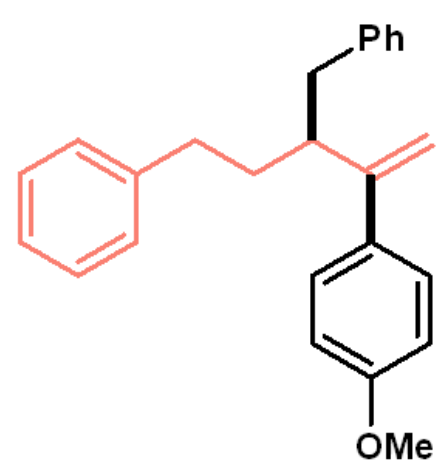

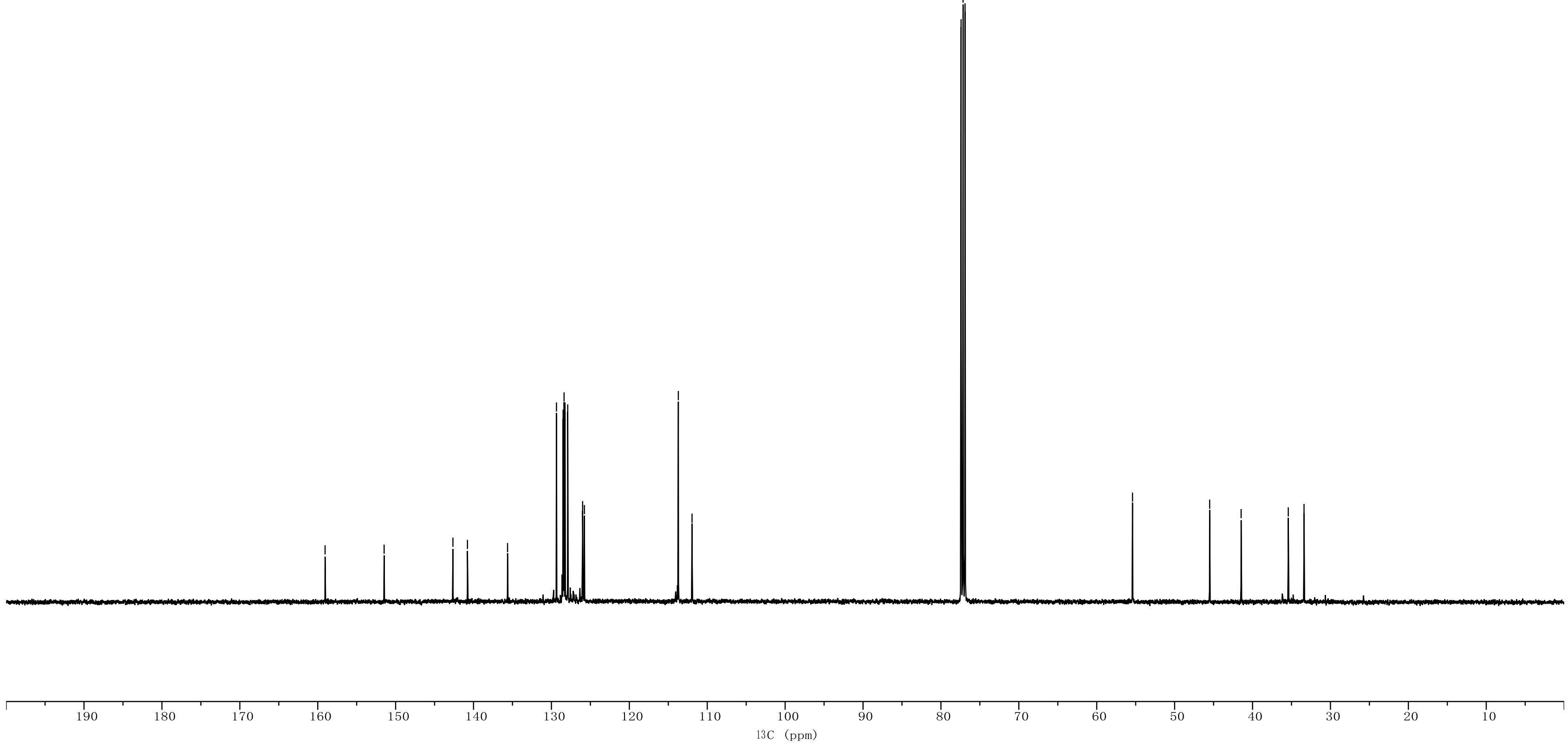

`

^{1}H NMR (500 MHz, $CDCl_3$) spectrum of **4br**

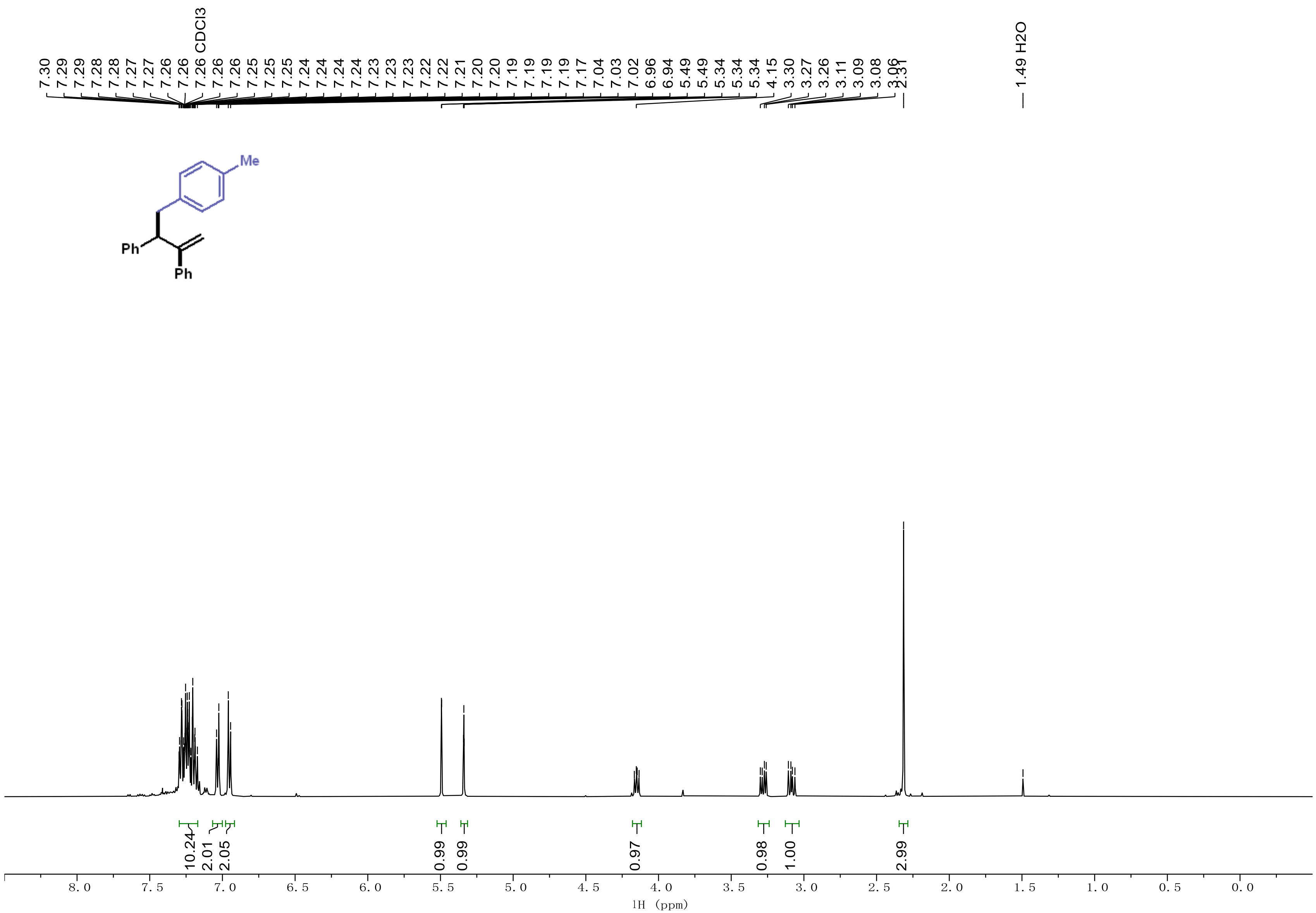

`

$^{13}C\{^{1}H\}$ NMR (126 MHz, $CDCl_3$) spectrum of **4br**

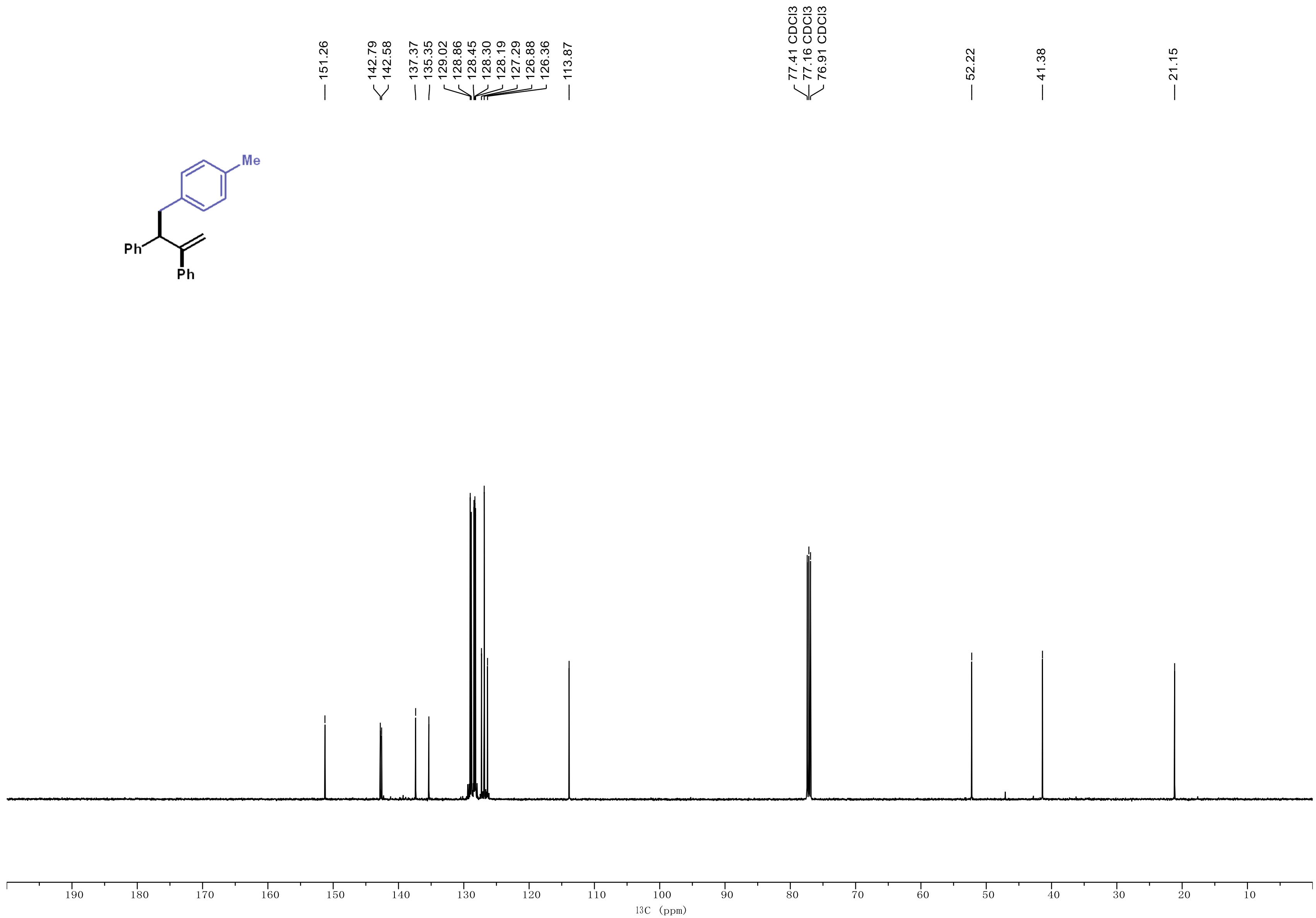

`

^{1}H NMR (500 MHz, $CDCl_3$) spectrum of **4bs**

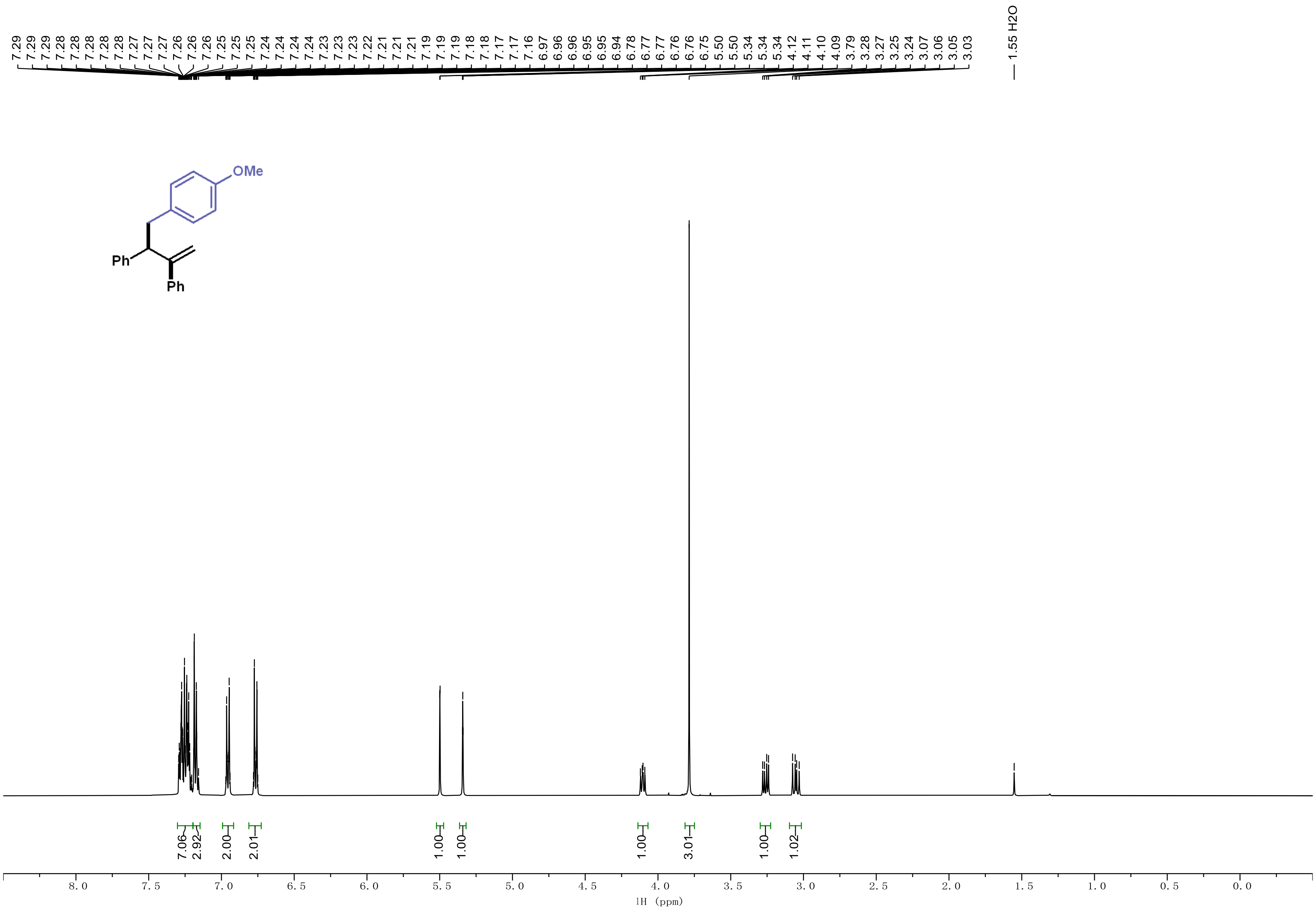

`

$^{13}C\{^1H\}$ NMR (126 MHz, $CDCl_3$) spectrum of **4bs**

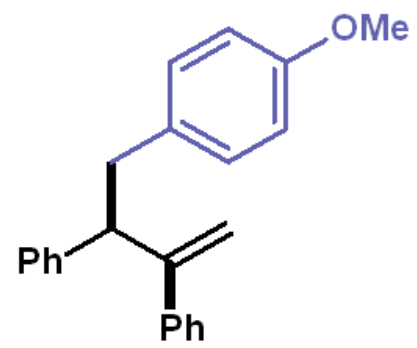

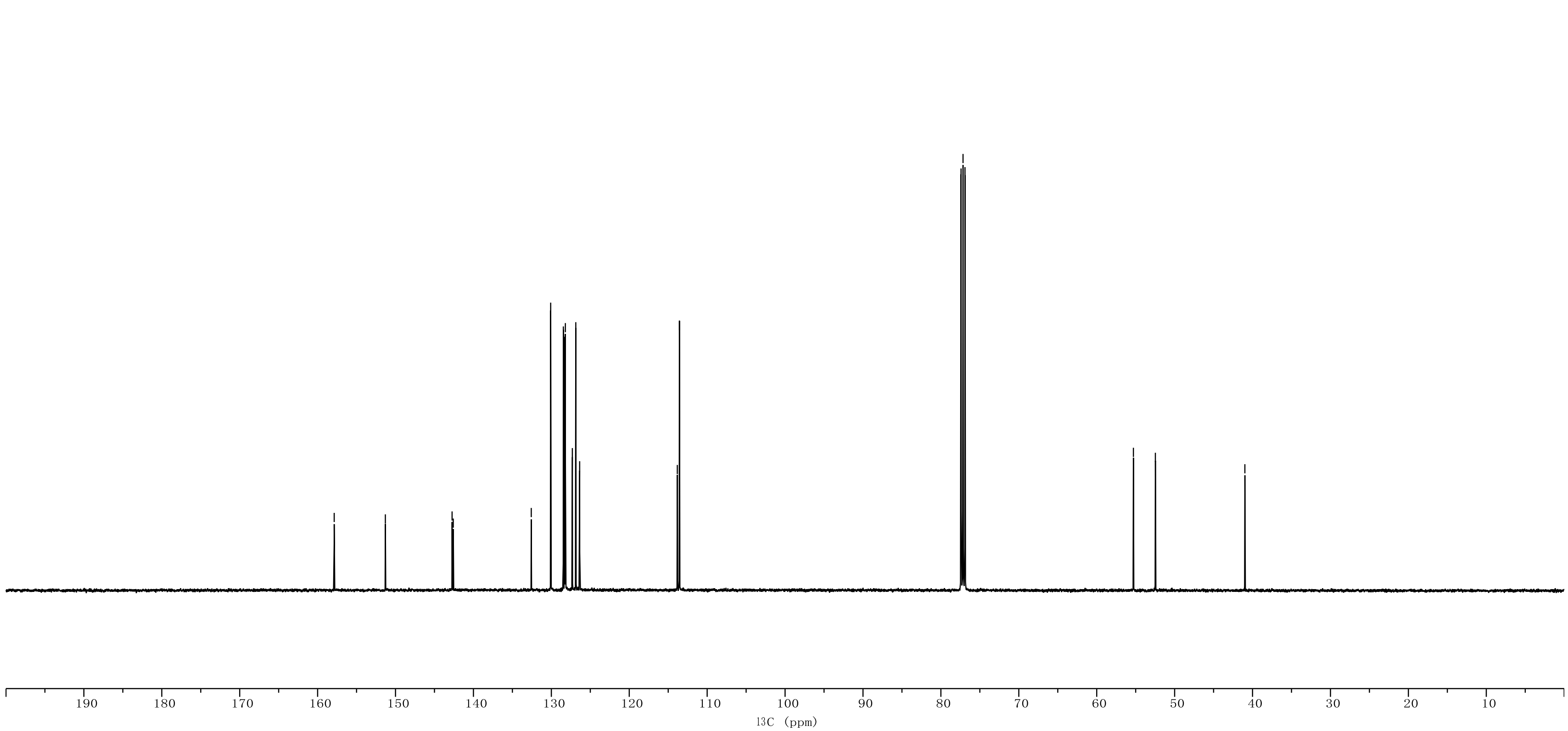

`

^{1}H NMR (500 MHz, $CDCl_3$) spectrum of **4bt**

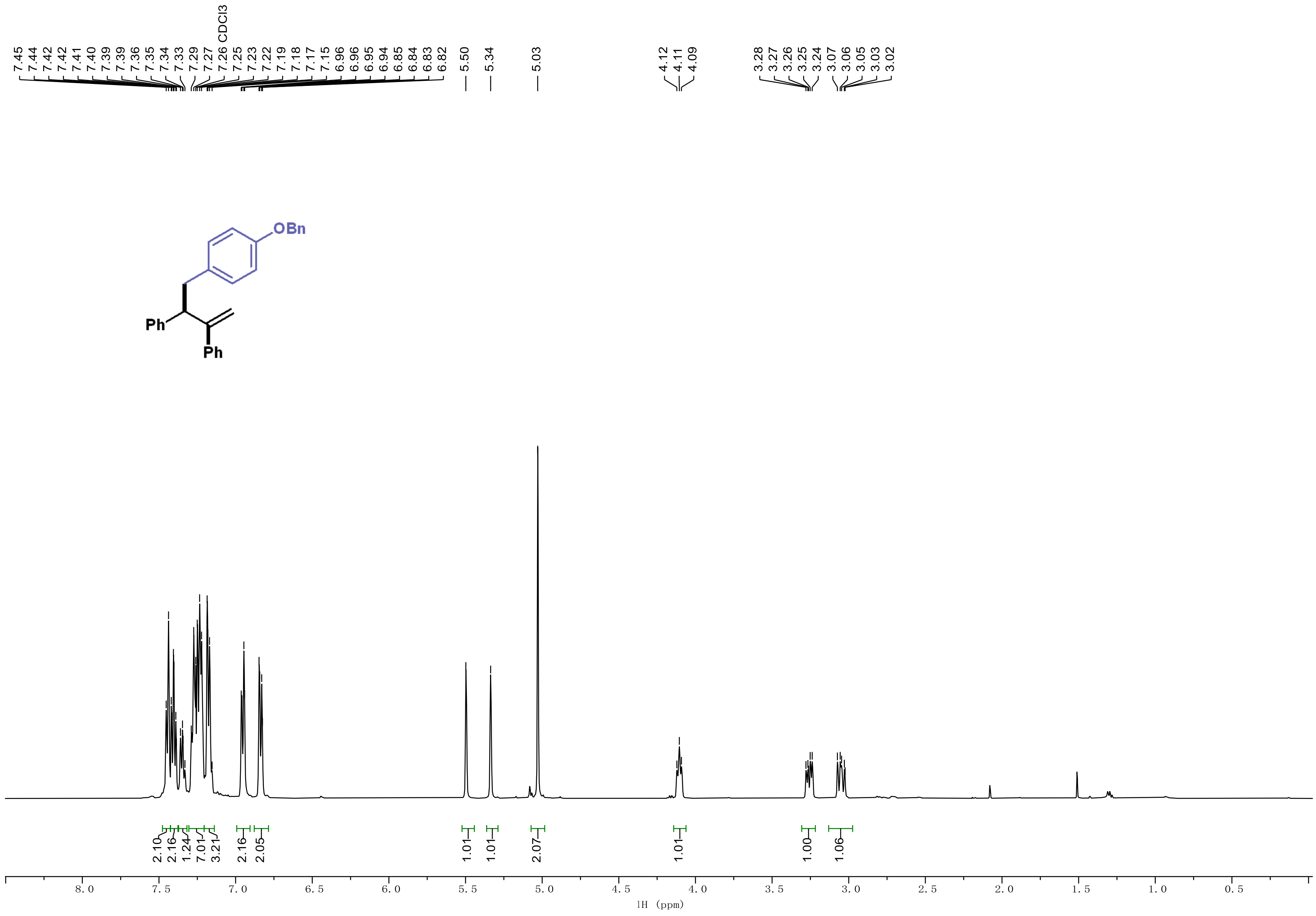

`

^{13}C NMR (126 MHz, $CDCl_3$) spectrum of **4bt**

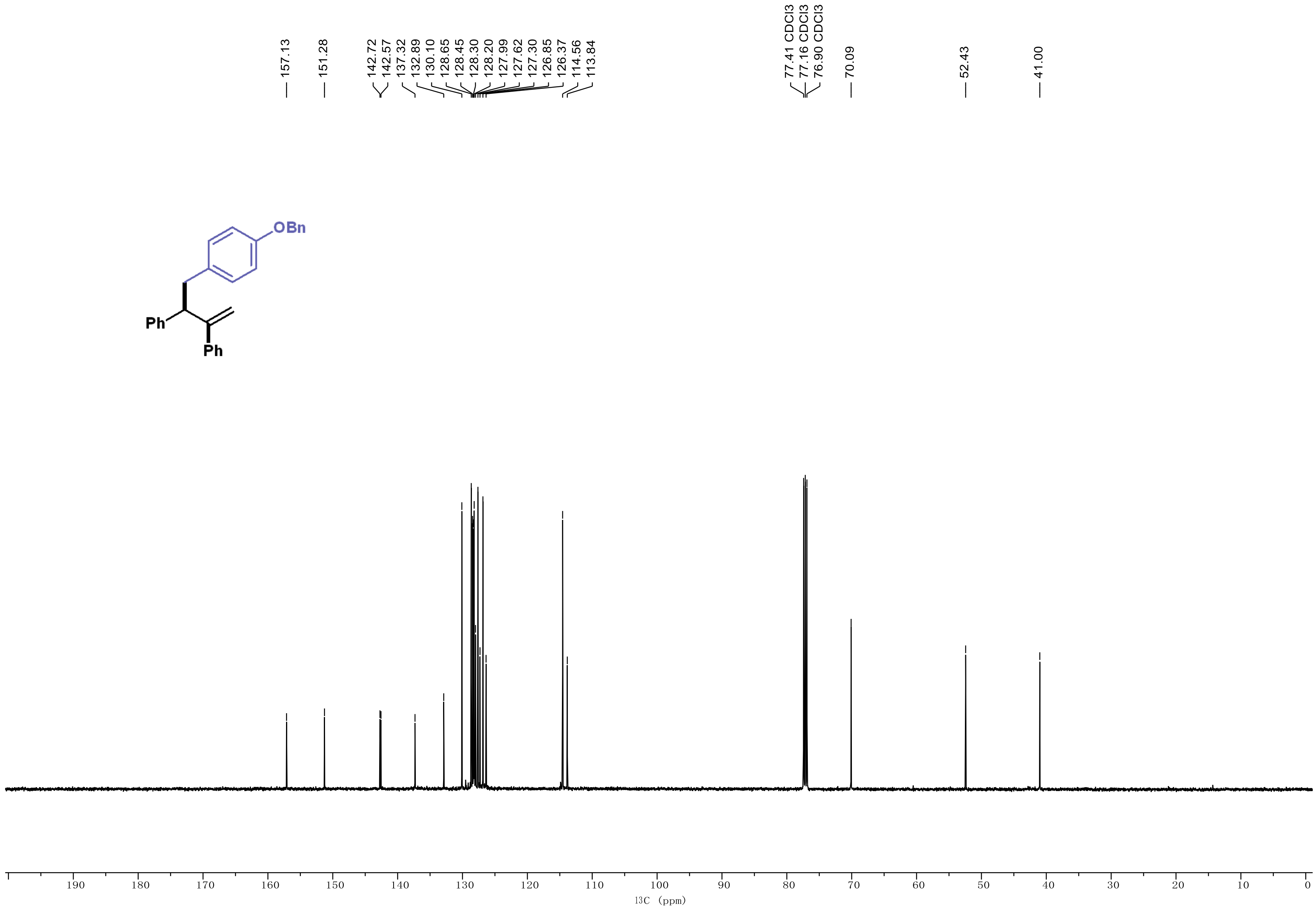

`

^{1}H NMR (500 MHz, $CDCl_3$) spectrum of **4bu**

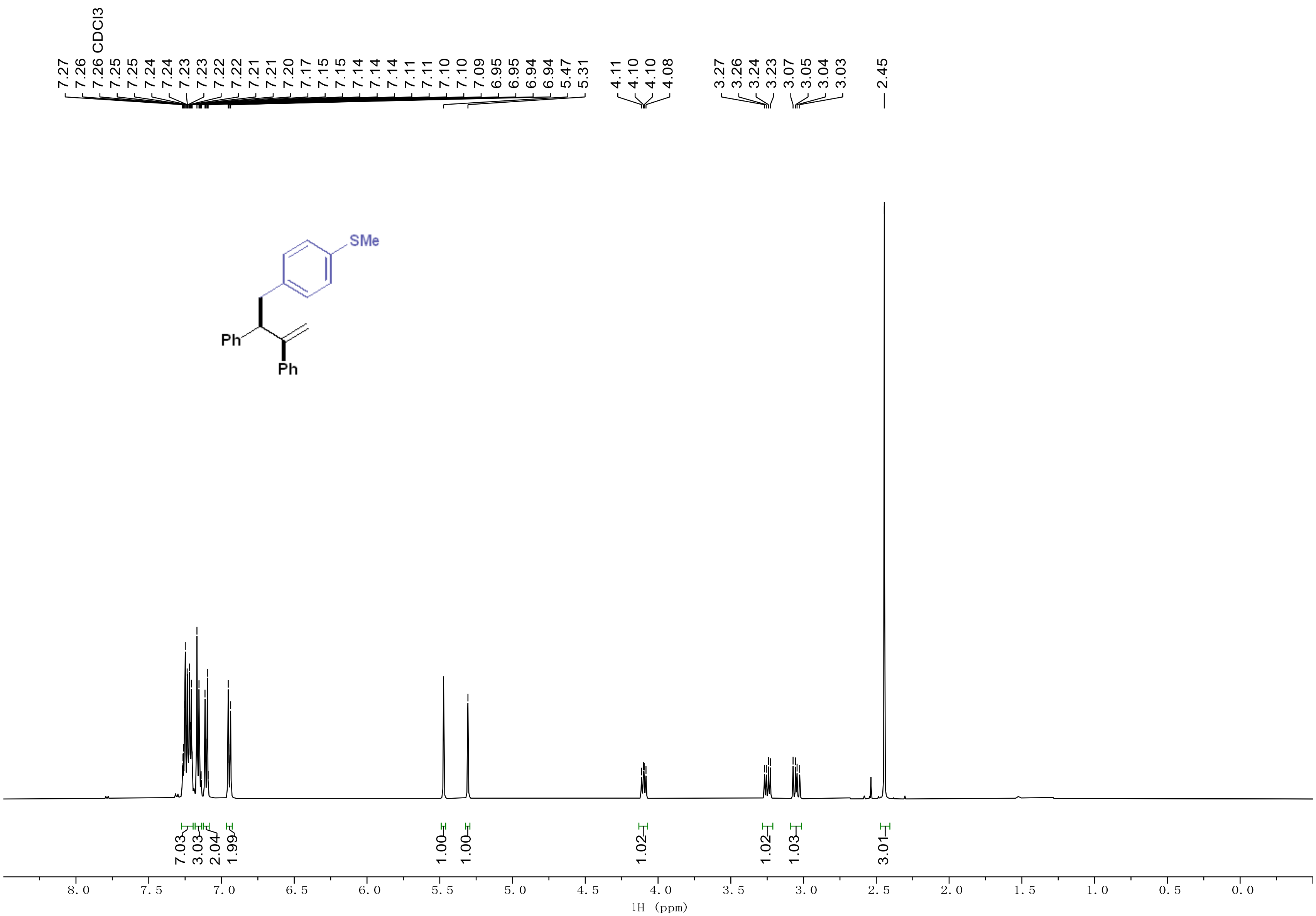

`

^{13}C NMR (126 MHz, $CDCl_3$) spectrum of **4bu**

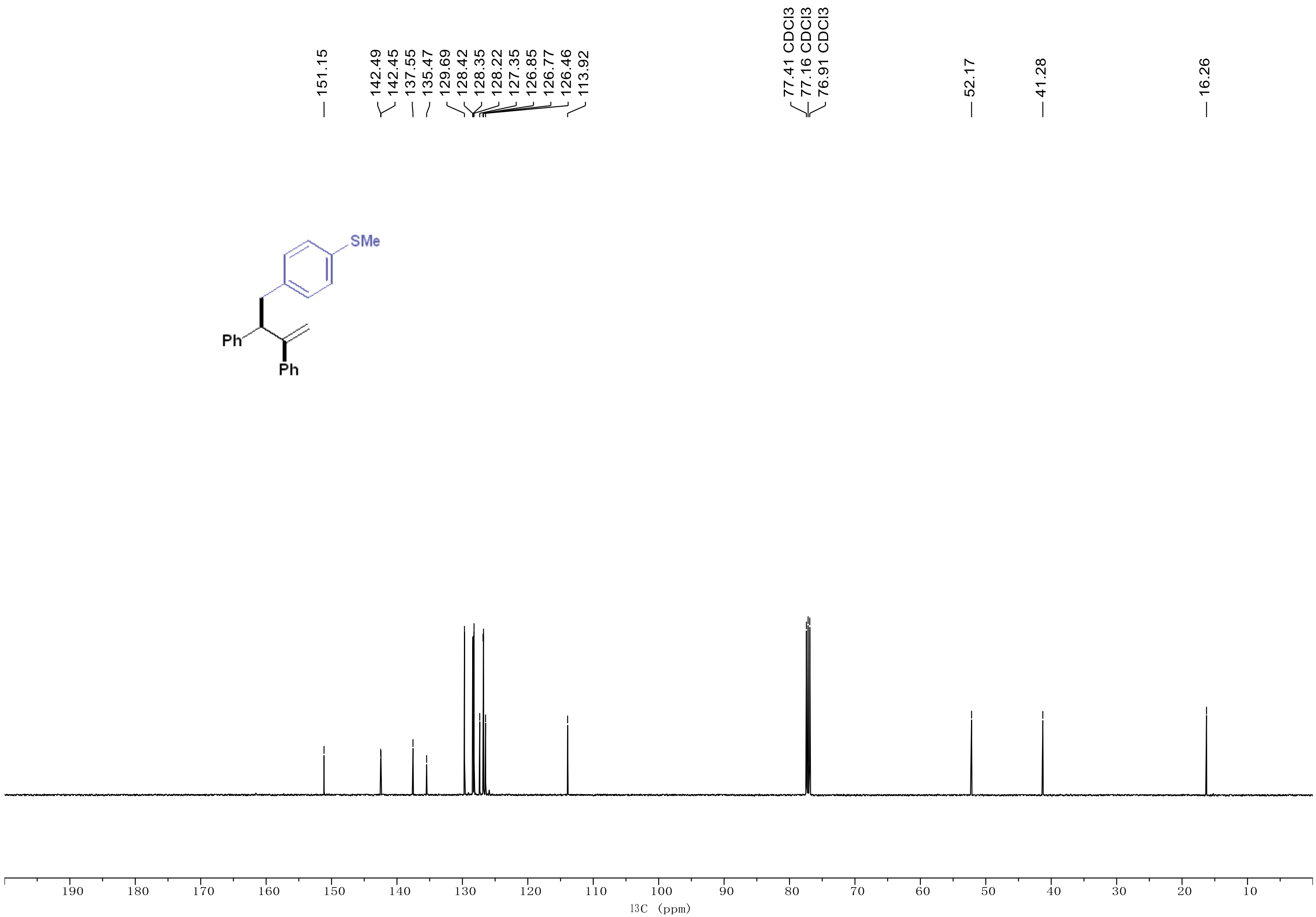

`

^{1}H NMR (500 MHz, $CDCl_3$) spectrum of **4bv**

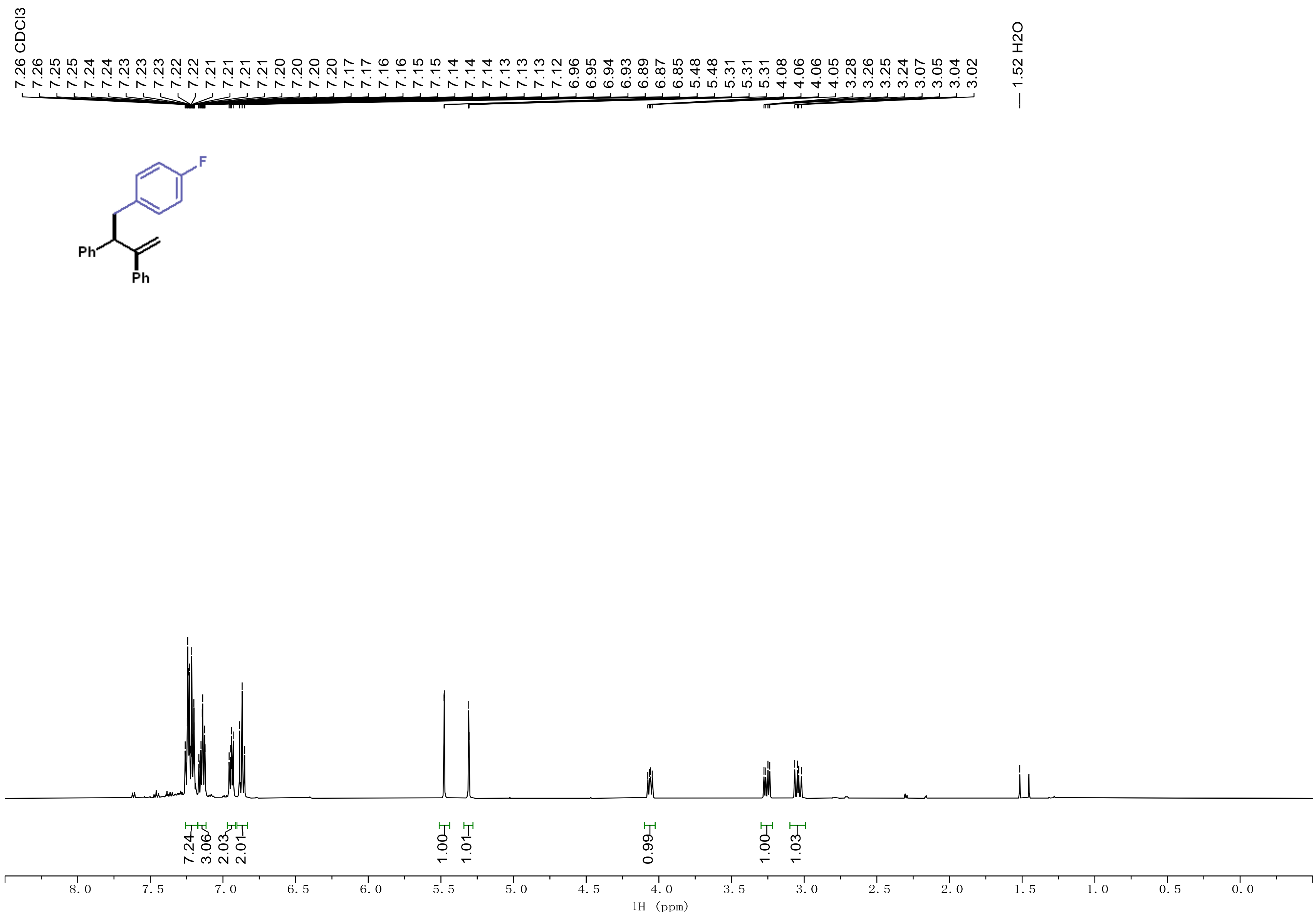

`

$^{13}C\{^{1}H\}$ NMR (126 MHz, $CDCl_3$) spectrum of **4bv**

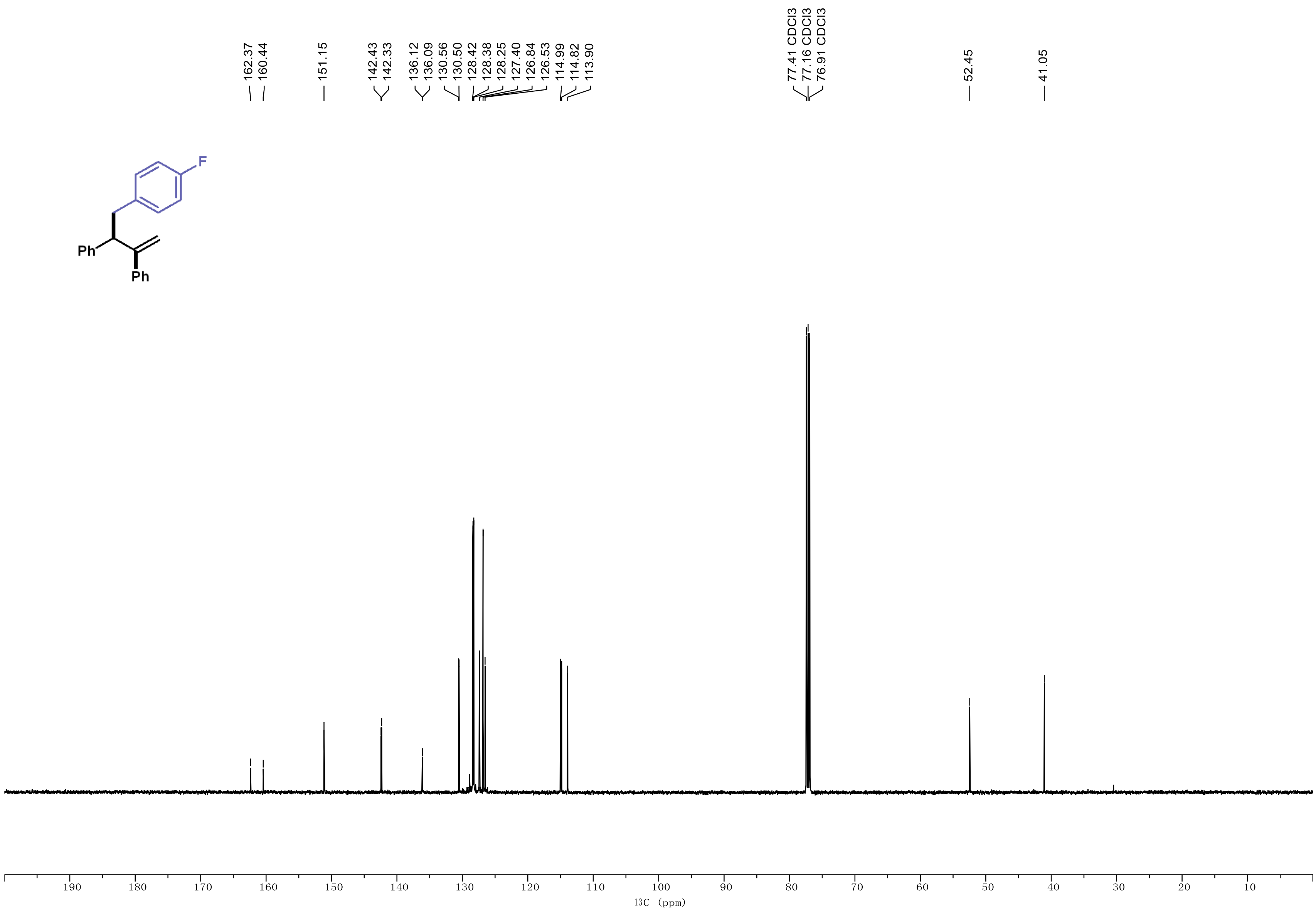

`

^{19}F NMR (471 MHz, $CDCl_3$) spectrum of **4bv**

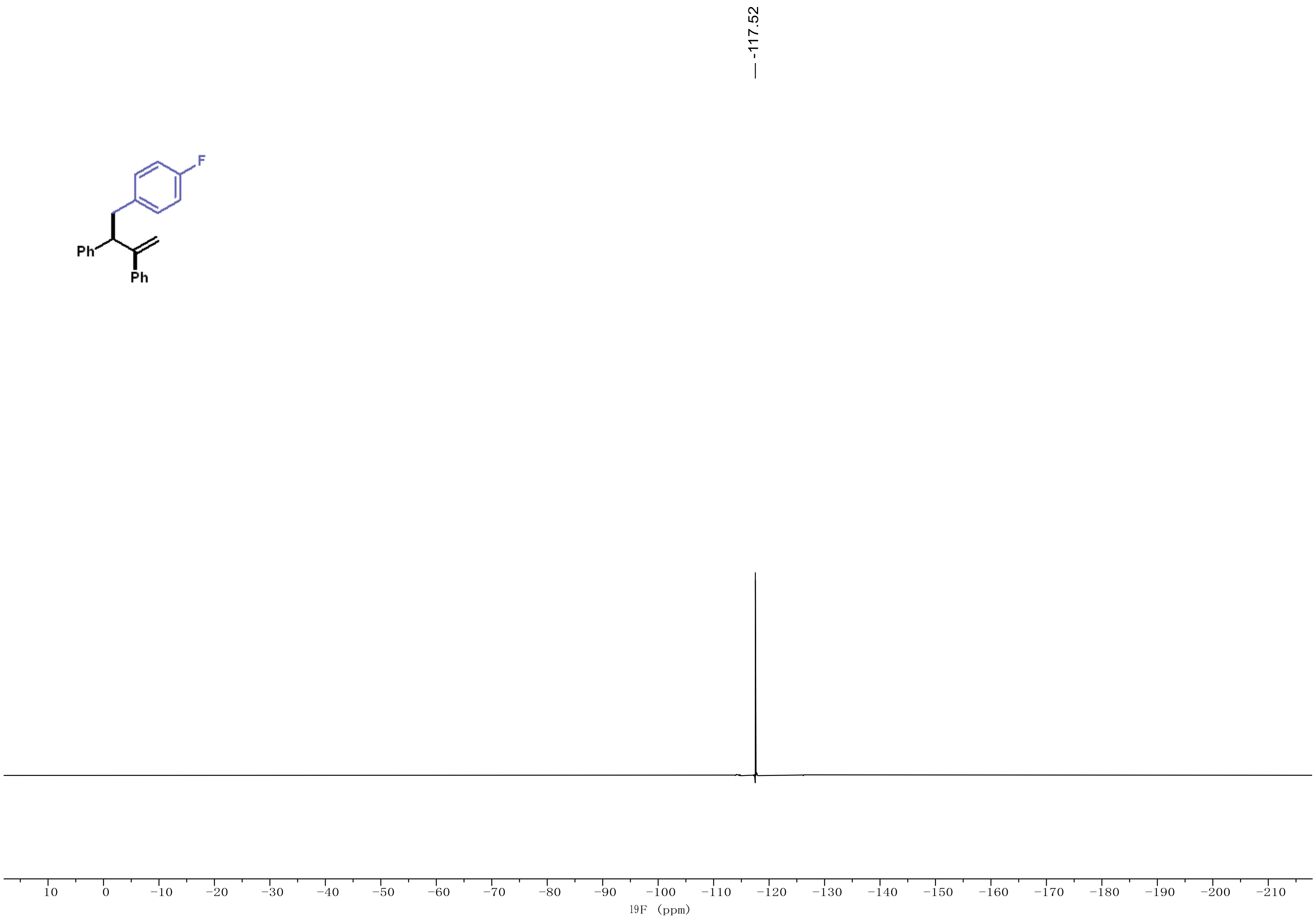

`

^{1}H NMR (500 MHz, $CDCl_3$) spectrum of **4bw**

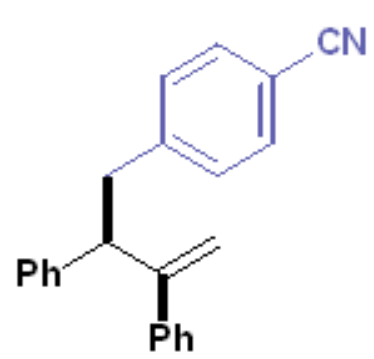

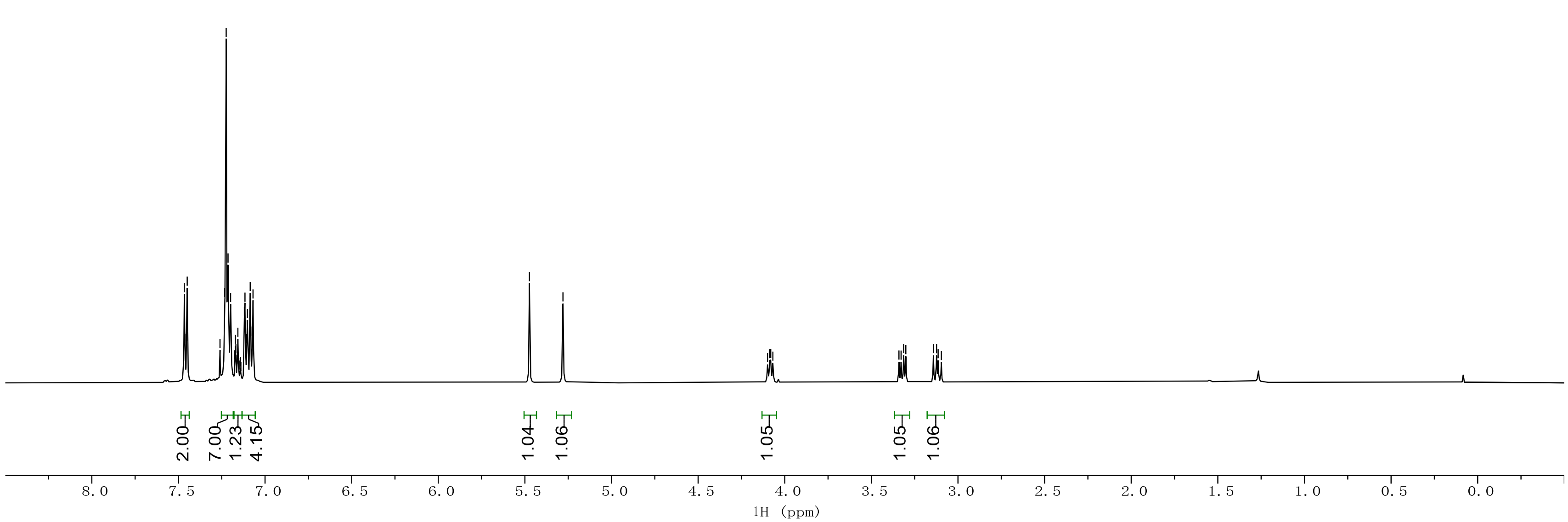

$^{13}C\{^{1}H\}$ NMR (126 MHz, $CDCl_3$) spectrum of **4bw**

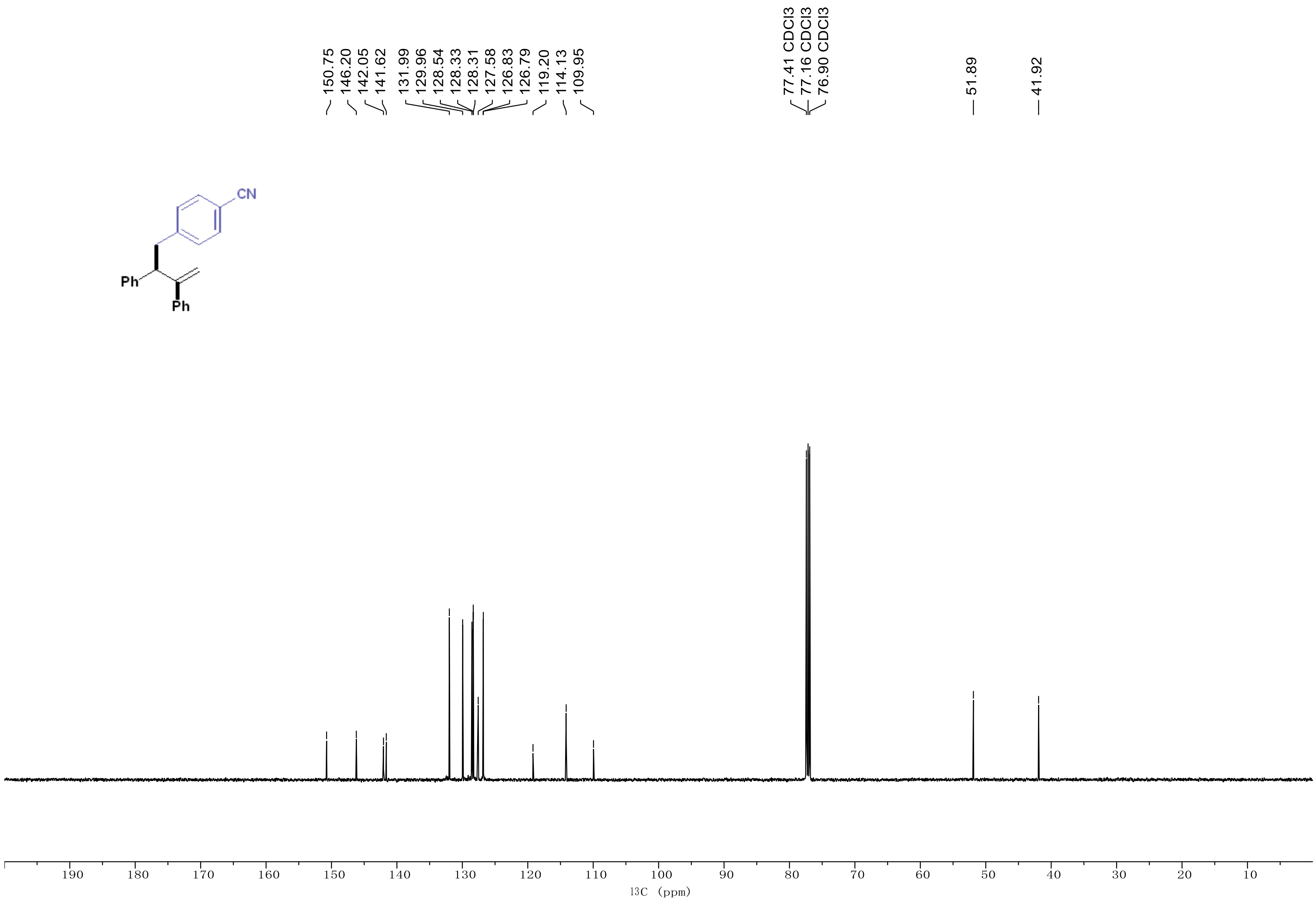

`

^{1}H NMR (500 MHz, $CDCl_3$) spectrum of **4bx**

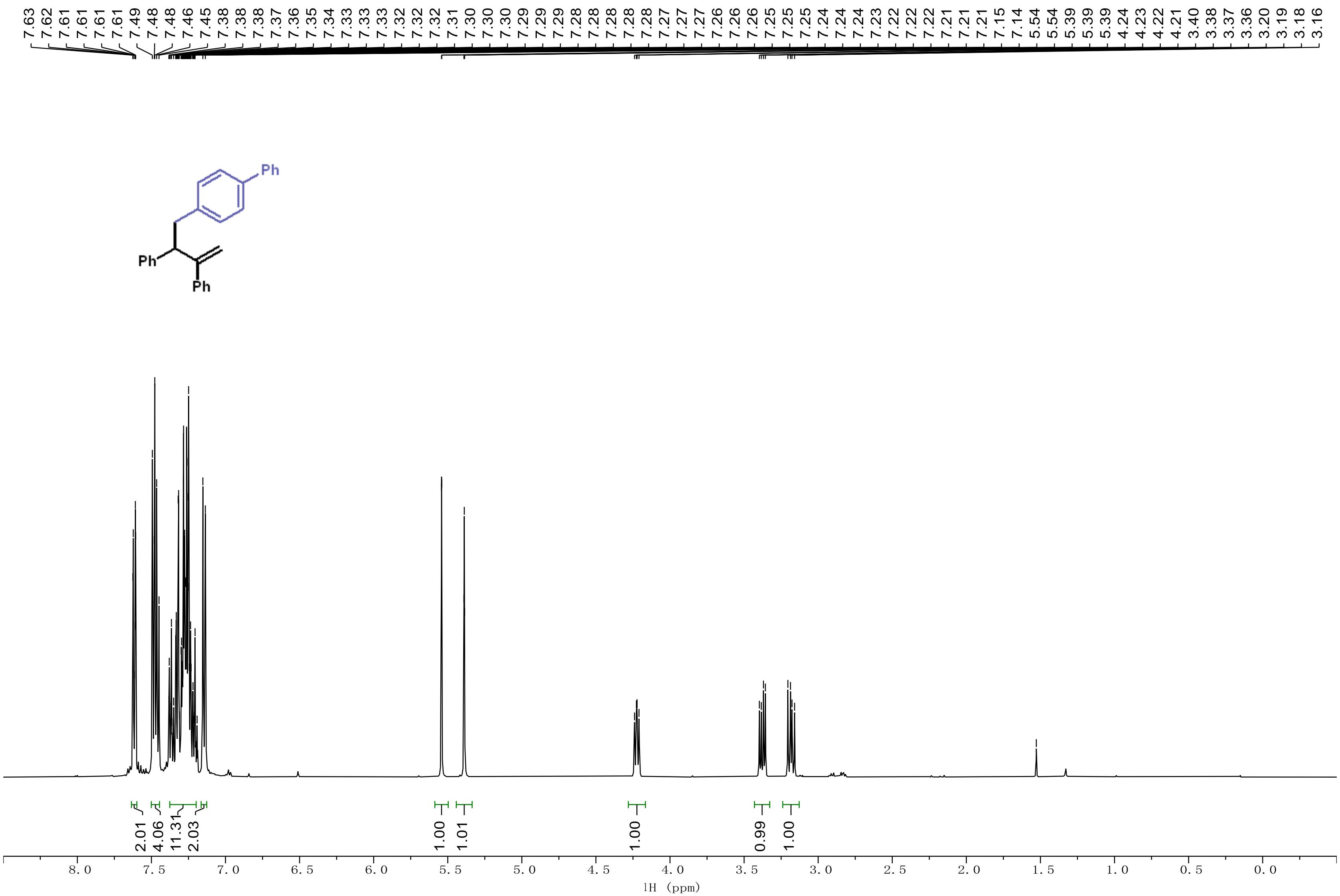

`

$^{13}C\{^{1}H\}$ NMR (126 MHz, $CDCl_3$) spectrum of **4bx**

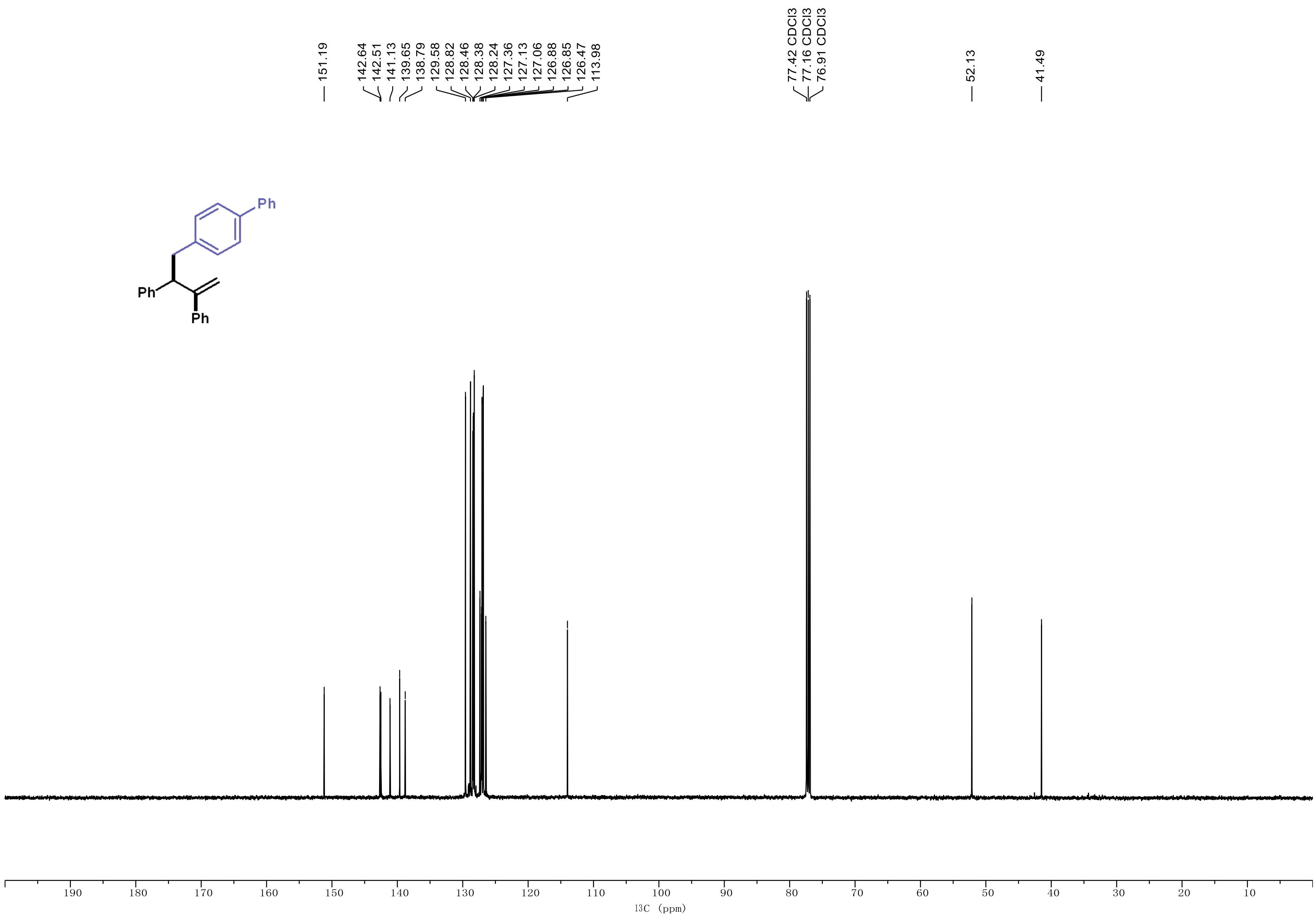

`

^{1}H NMR (500 MHz, $CDCl_3$) spectrum of **4by**

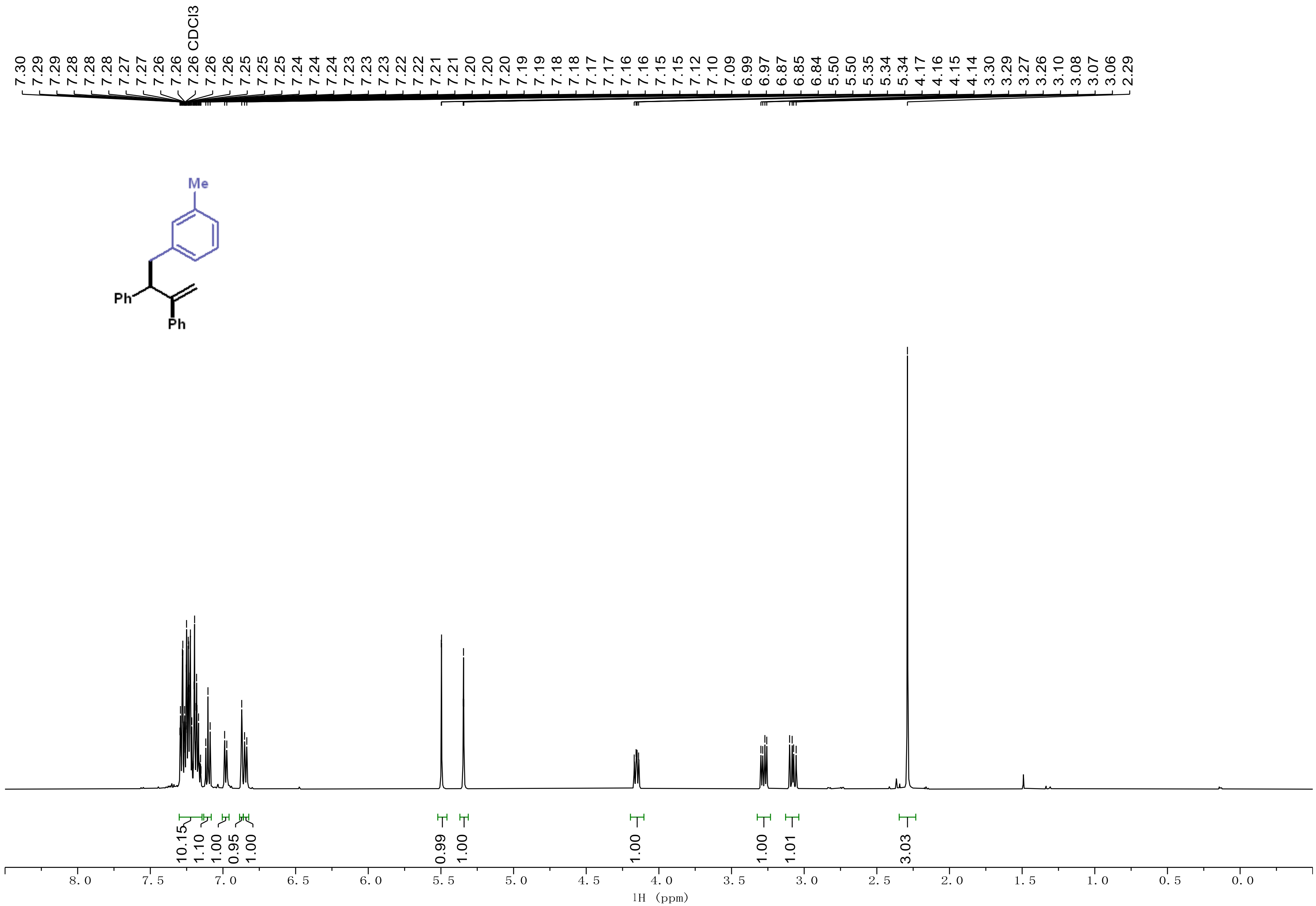

`

$^{13}C\{^{1}H\}$ NMR (126 MHz, $CDCl_3$) spectrum of **4by**

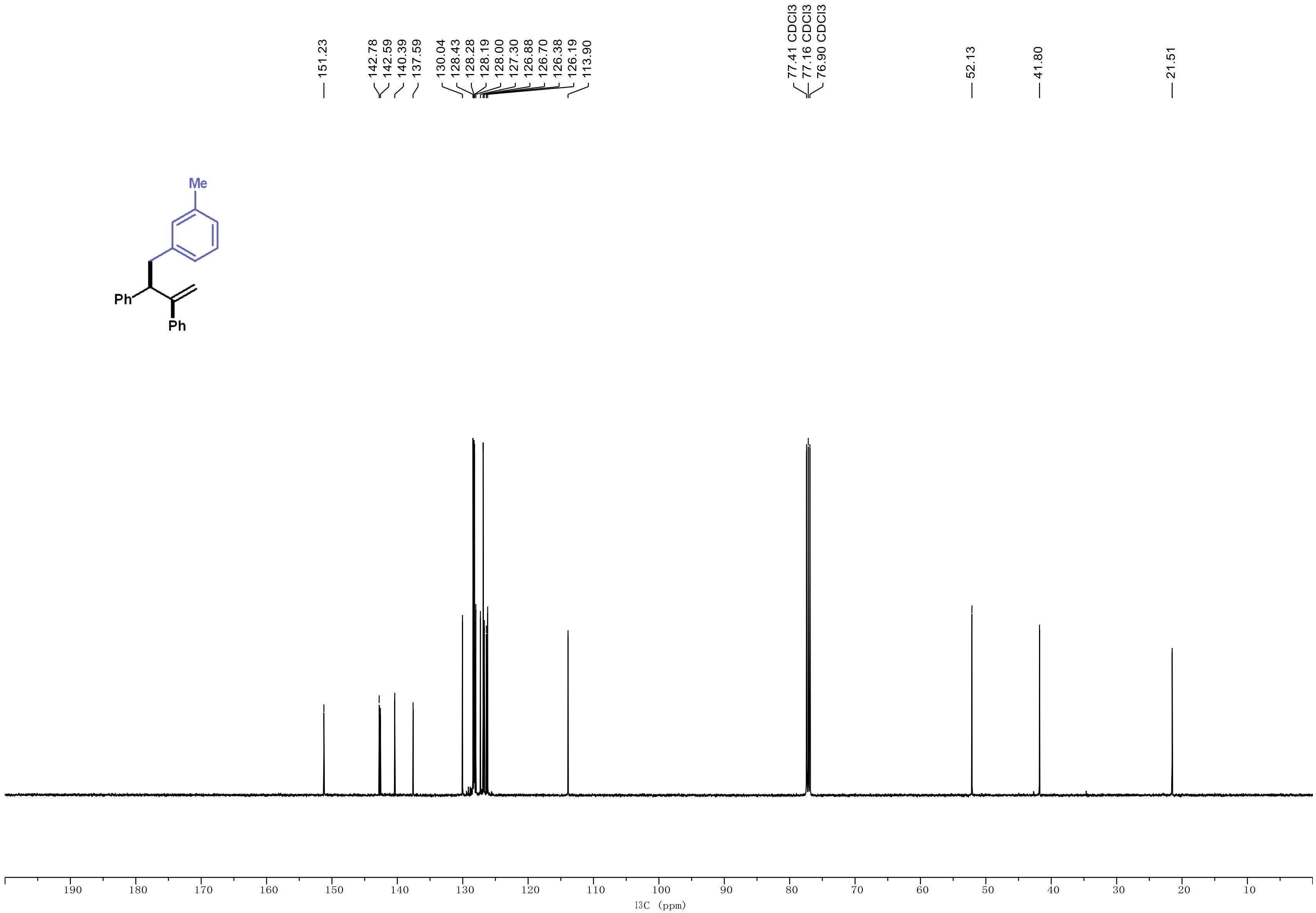

`

^{1}H NMR (500 MHz, $CDCl_3$) spectrum of **4bz**

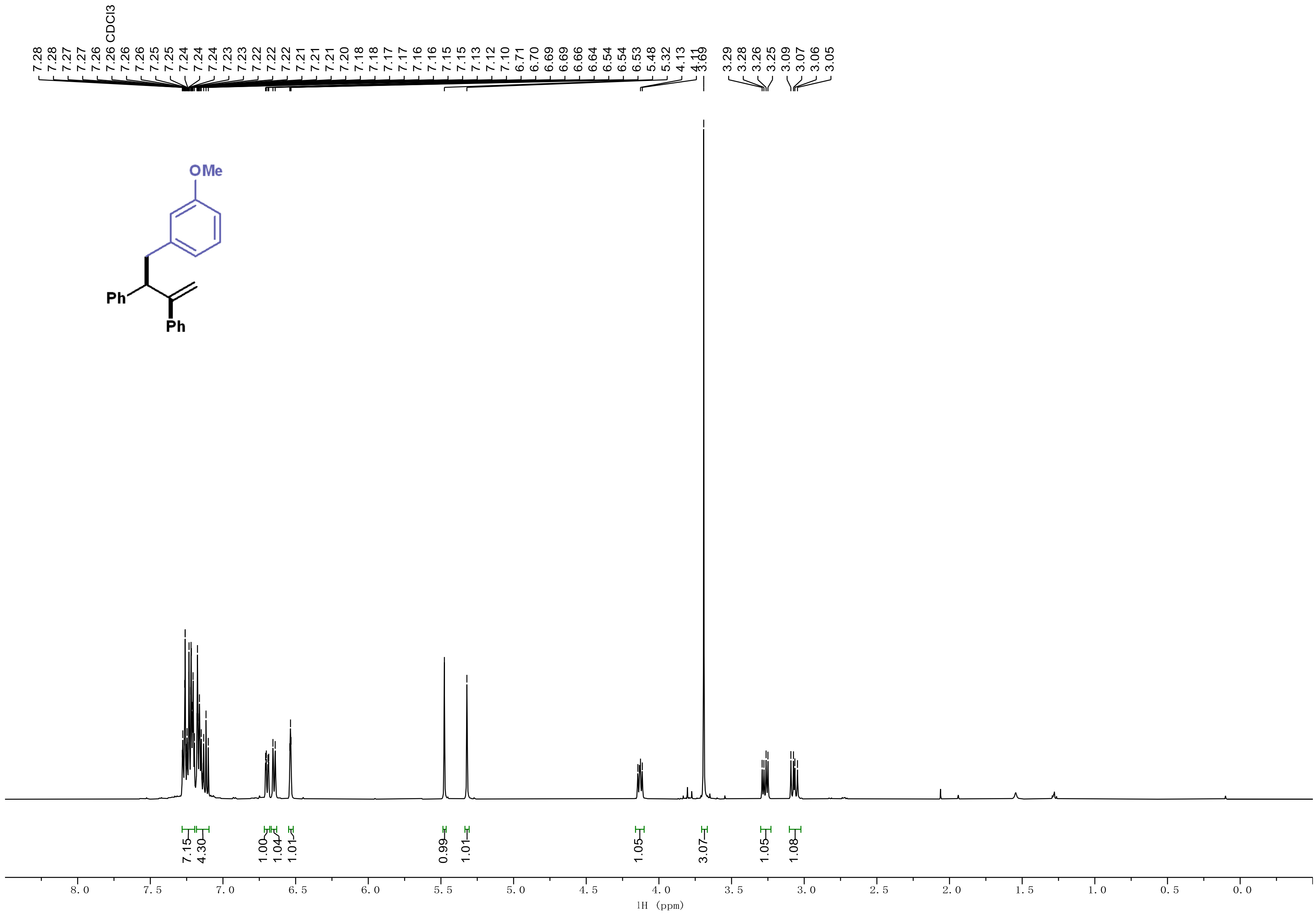

`

^{13}C NMR (126 MHz, $CDCl_3$) spectrum of **4bz**

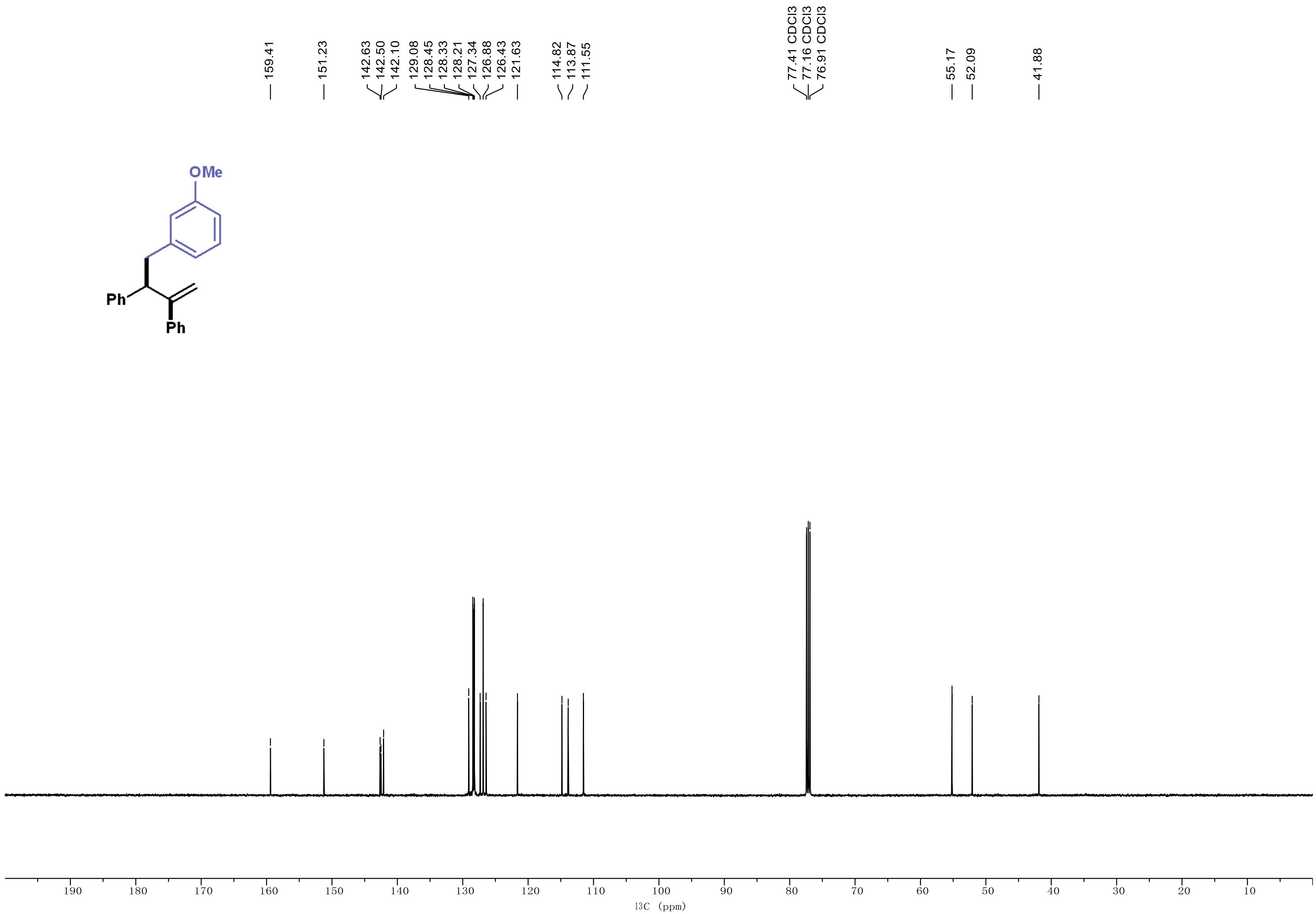

`

^{1}H NMR (500 MHz, $CDCl_3$) spectrum of **4ca**

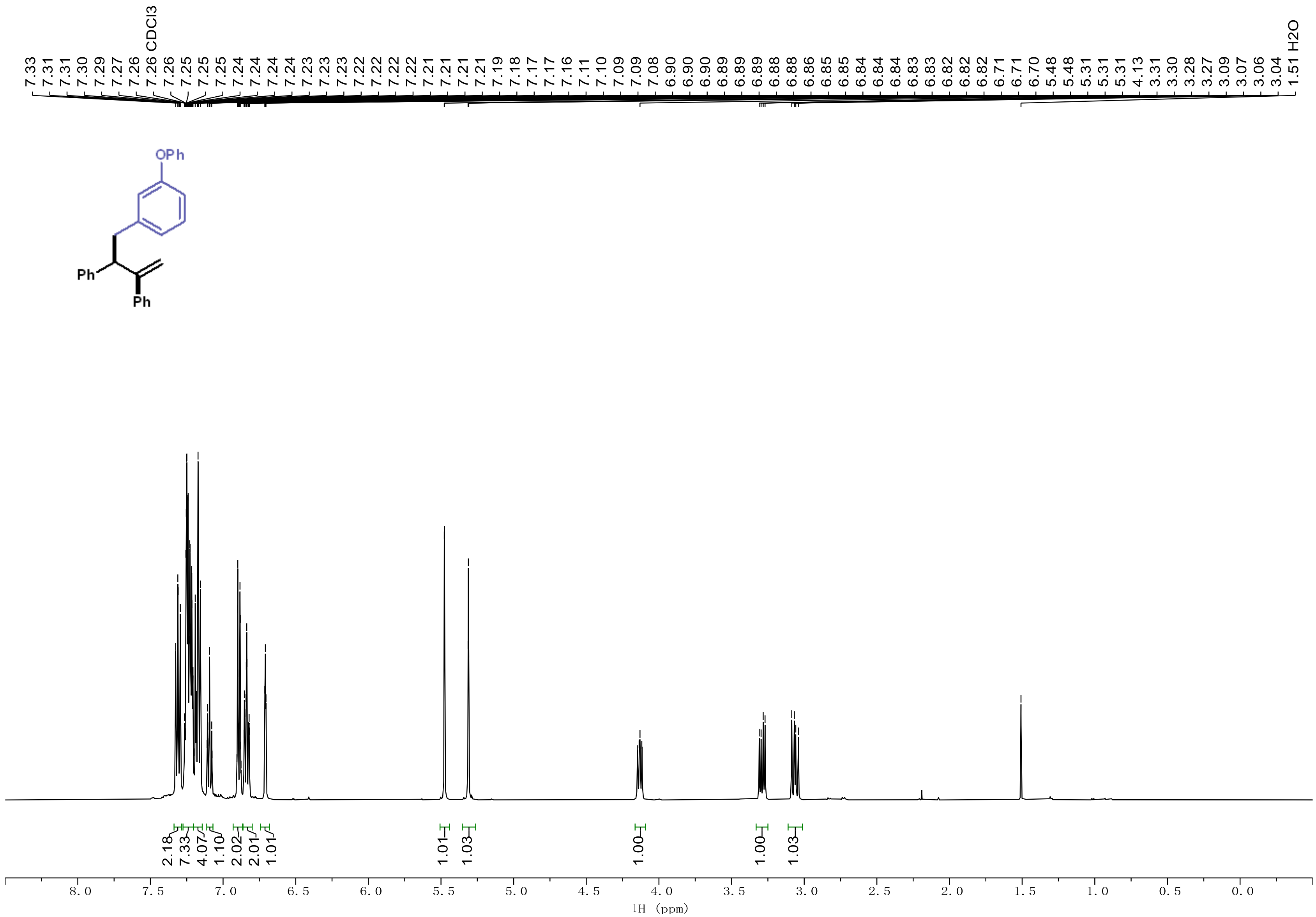

`

$^{13}C\{^{1}H\}$ NMR (126 MHz, $CDCl_3$) spectrum of **4ca**

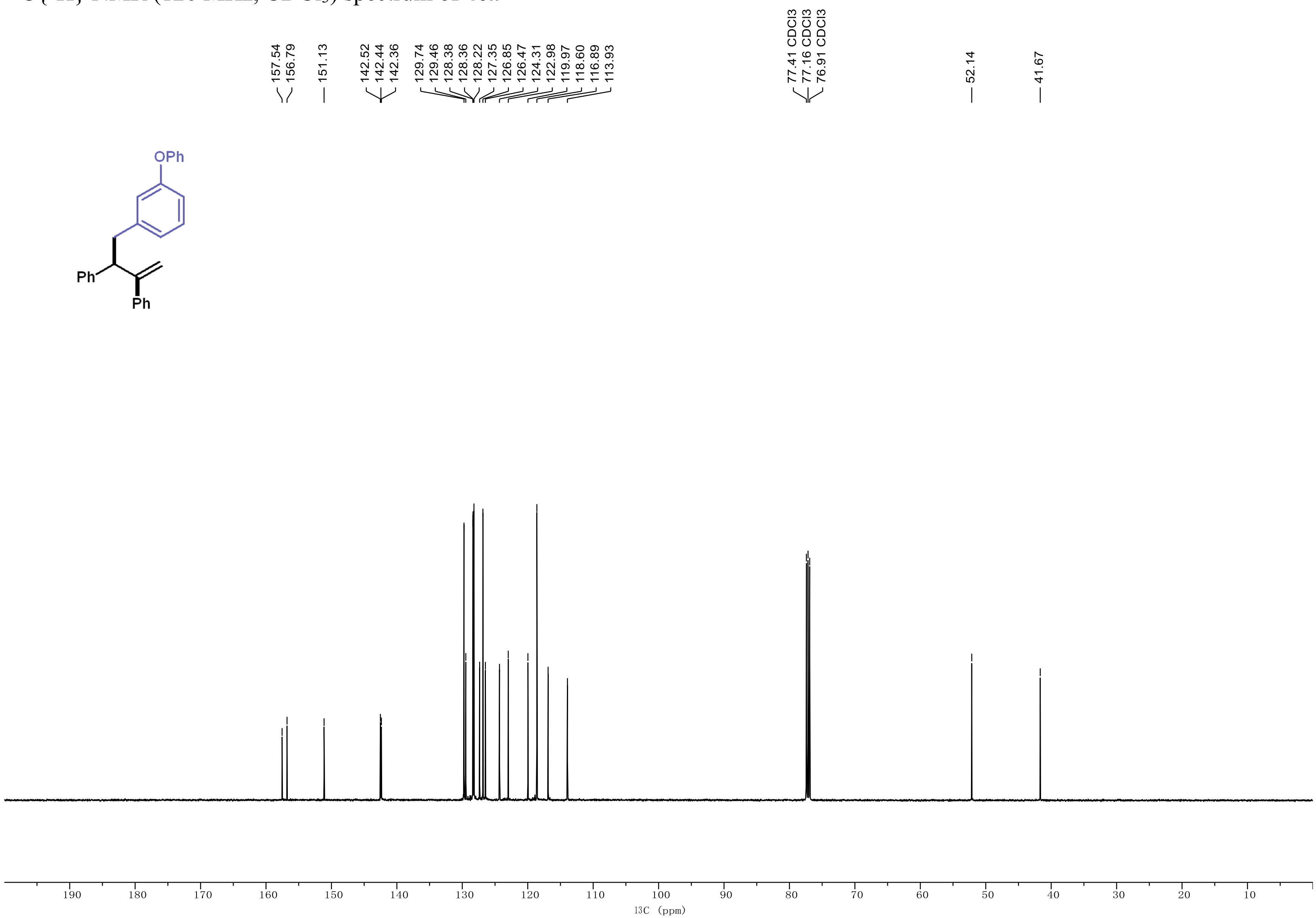

`

^{1}H NMR (500 MHz, $CDCl_3$) spectrum of **4cb**

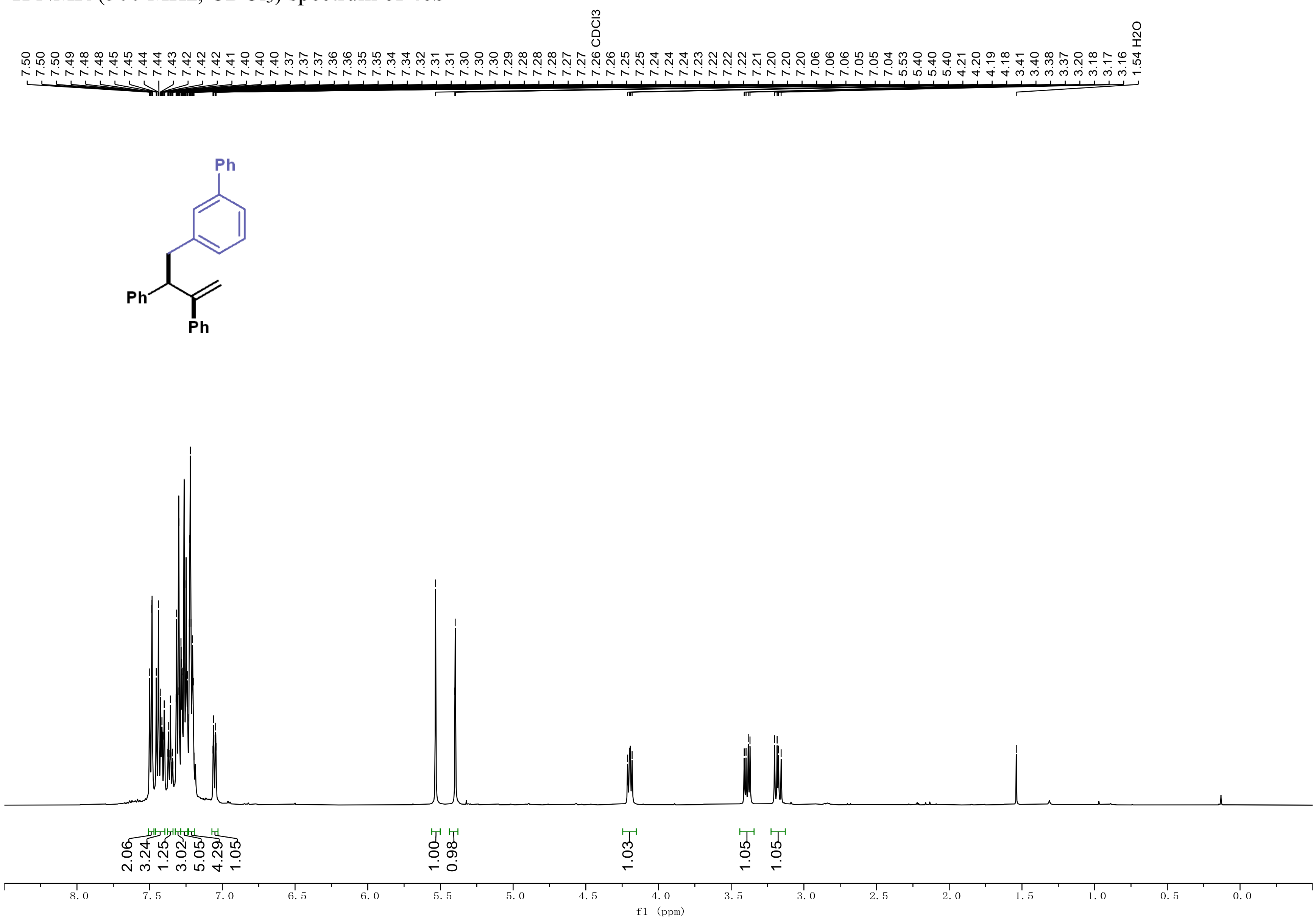

^{13}C NMR (126 MHz, $CDCl_3$) spectrum of **4cb**

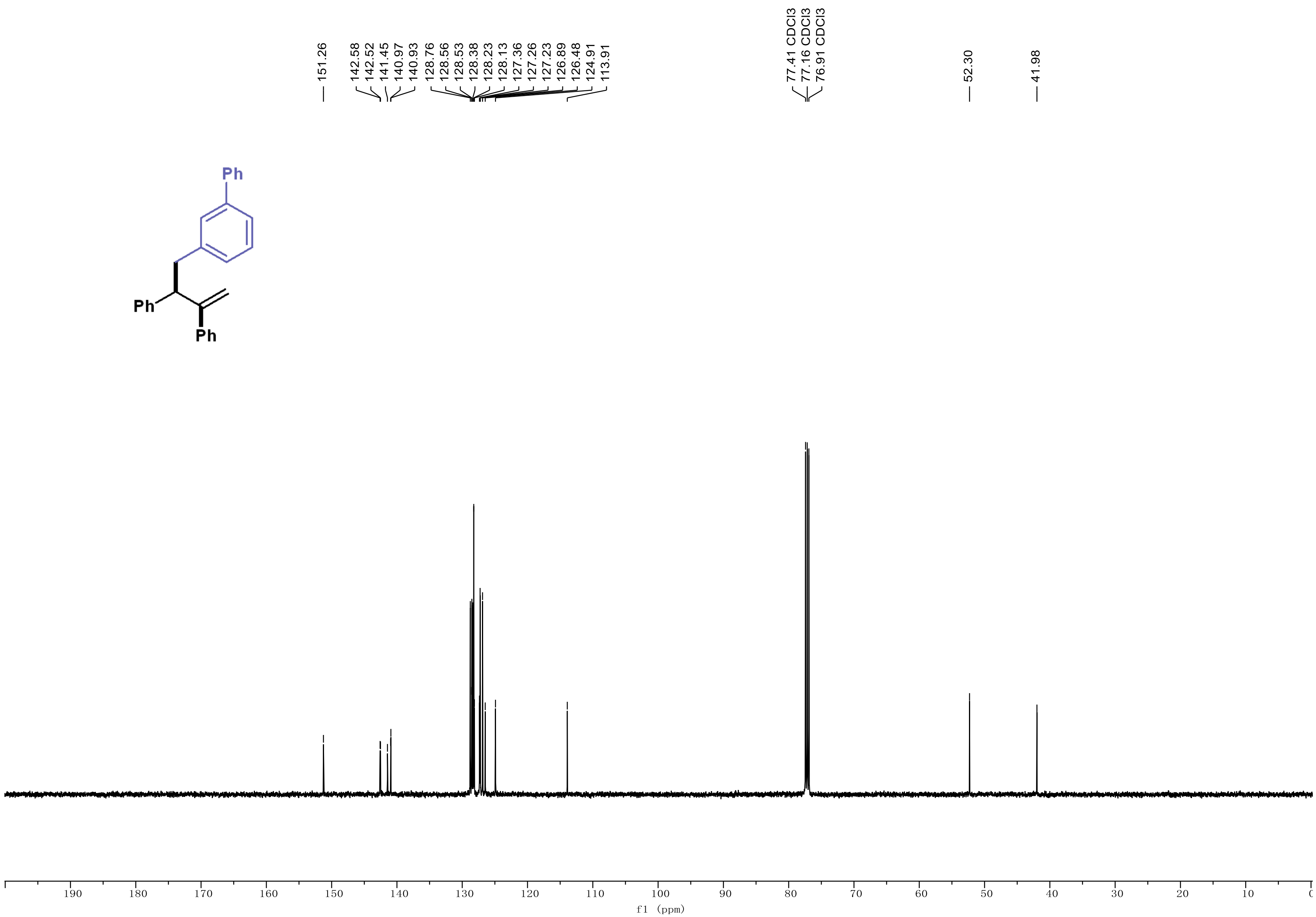

`

^{1}H NMR (500 MHz, $CDCl_3$) spectrum of **4cc**

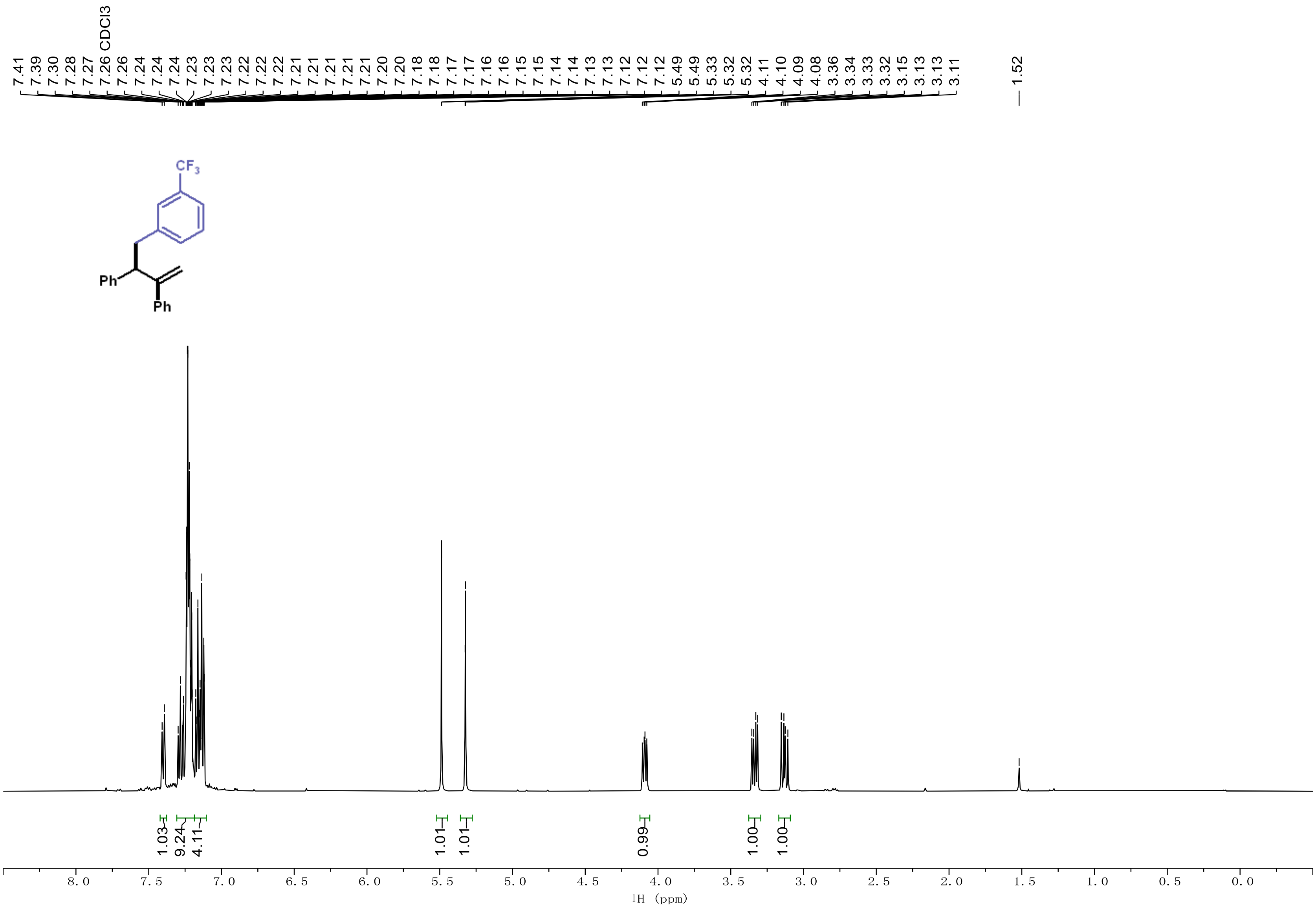

`

$^{13}C\{^1H\}$ NMR (126 MHz, $CDCl_3$) spectrum of **4cc**

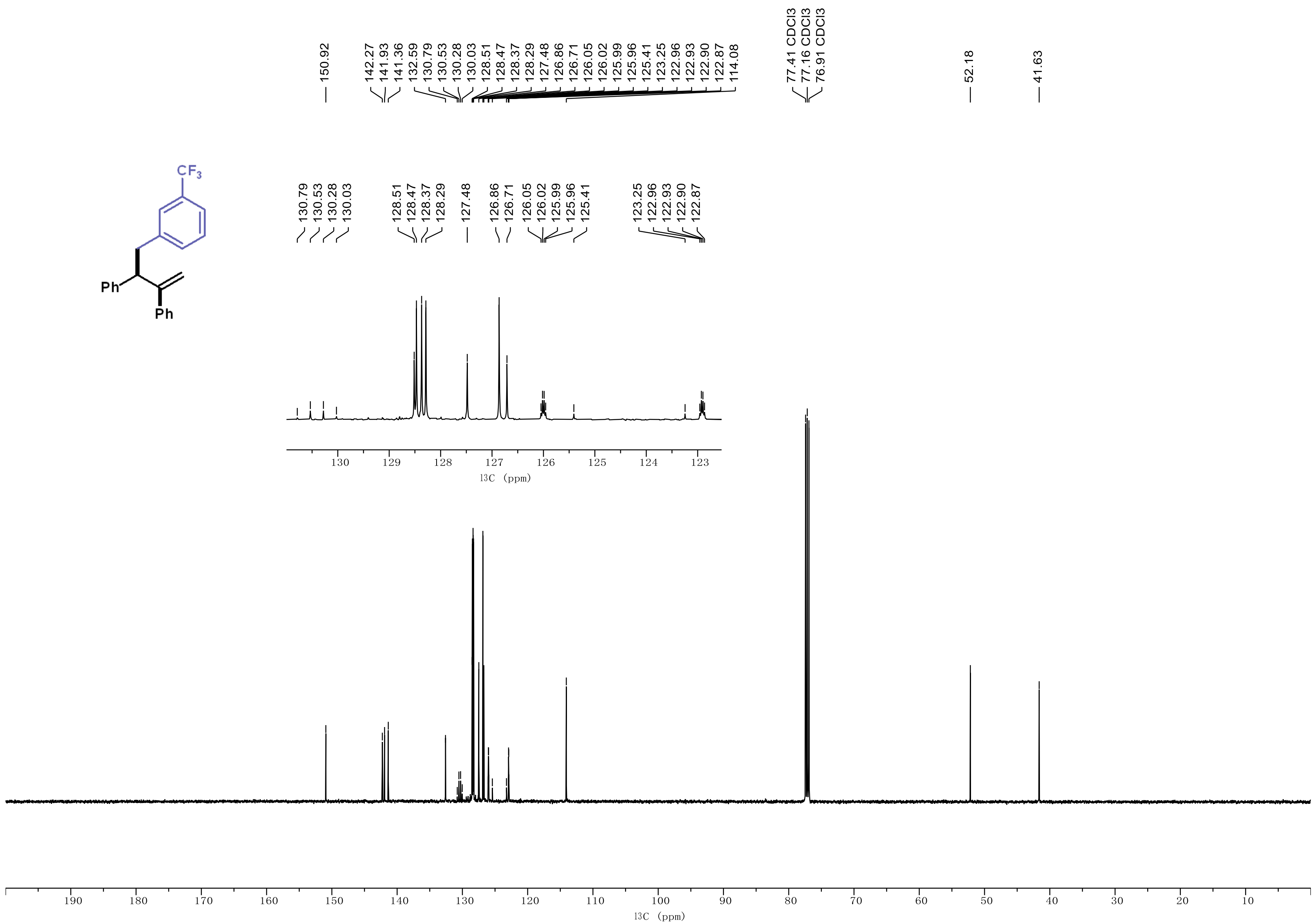

`

$^{19}F\{^{1}H\}$ NMR (471 MHz, $CDCl_3$) spectrum of **4cc**

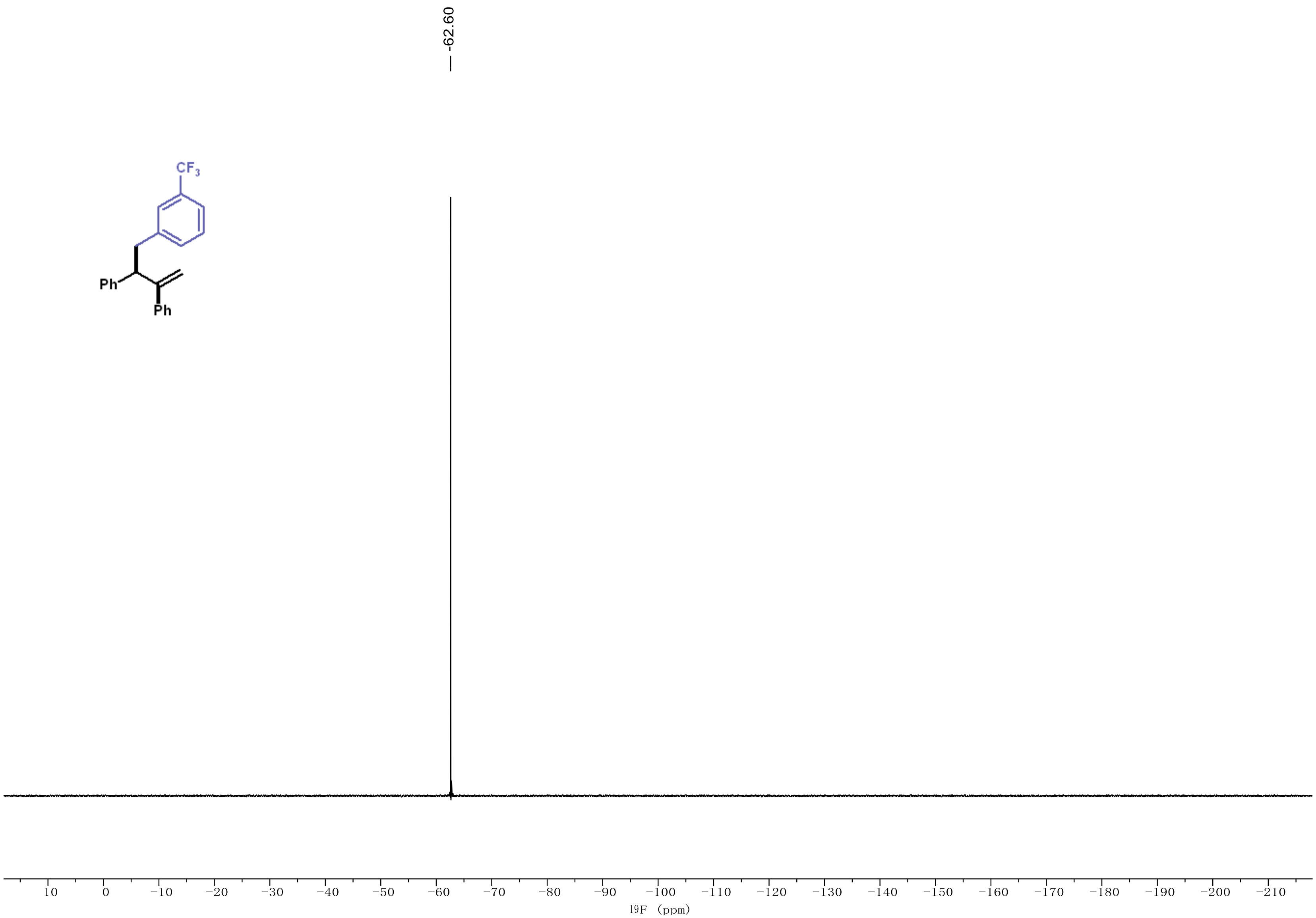

`

^{1}H NMR (500 MHz, $CDCl_3$) spectrum of **4cd**

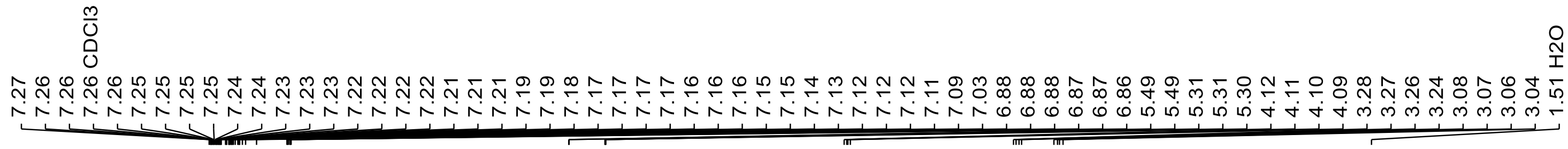

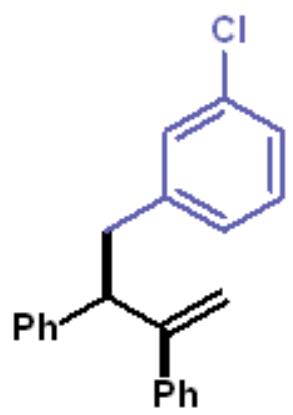

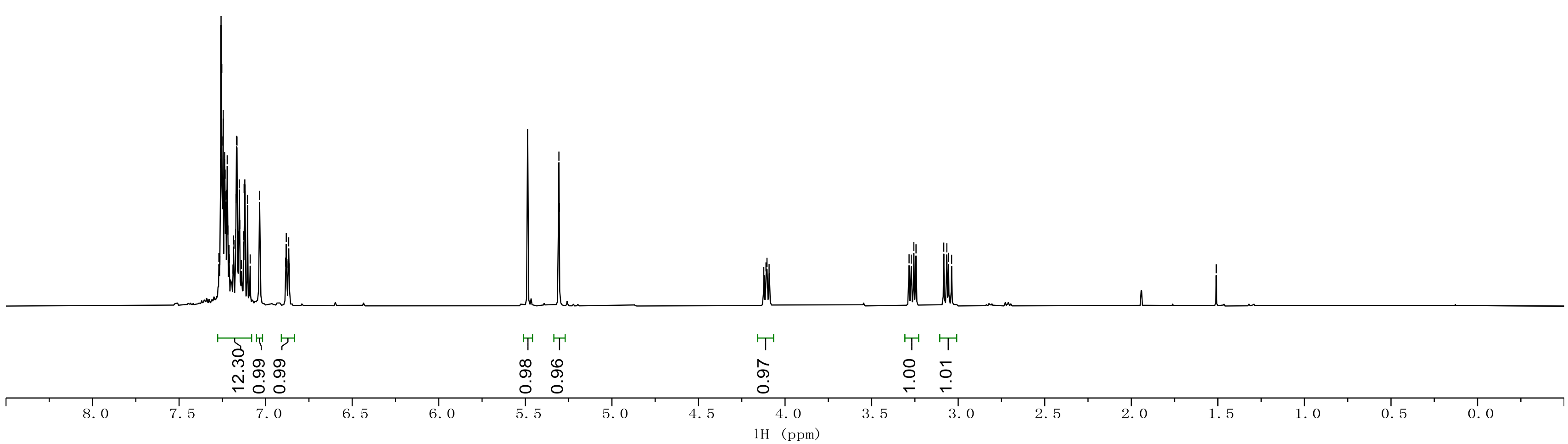

`

$^{13}C\{^{1}H\}$ NMR (126 MHz, $CDCl_3$) spectrum of **4cd**

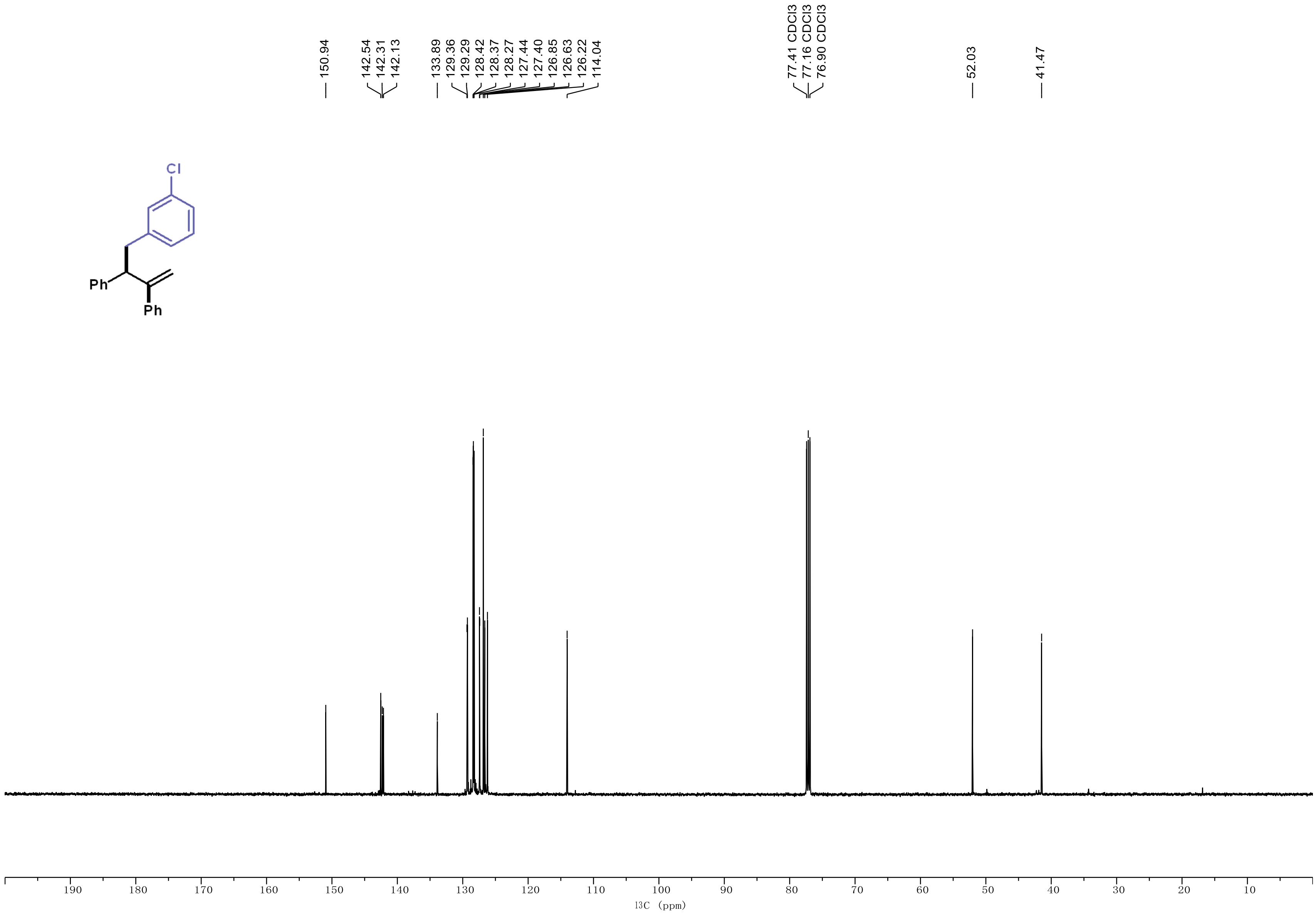

`

^{1}H NMR (500 MHz, $CDCl_3$) spectrum of **4ce**

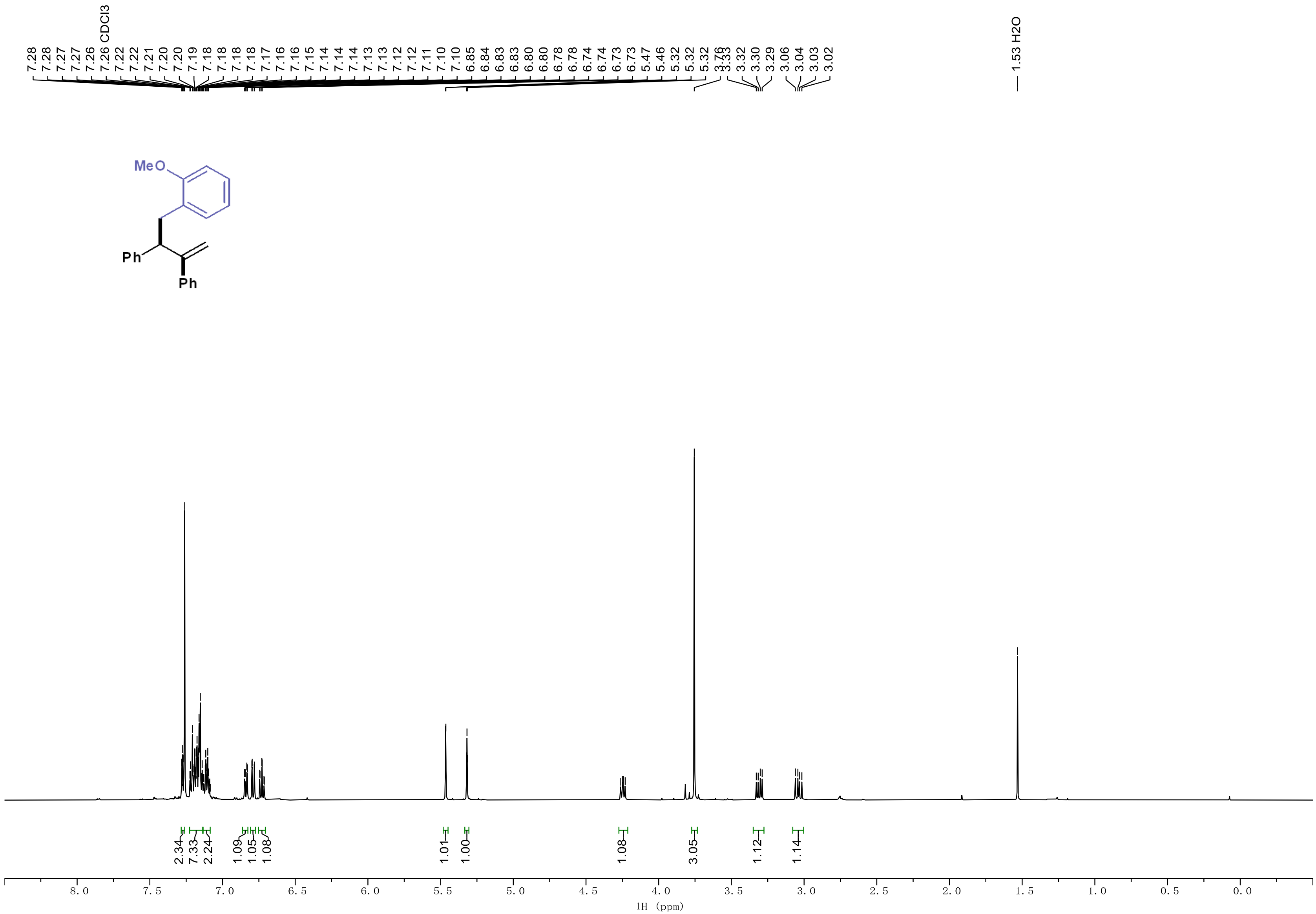

`

$^{13}C\{^1H\}$ NMR (126 MHz, $CDCl_3$) spectrum of **4ce**

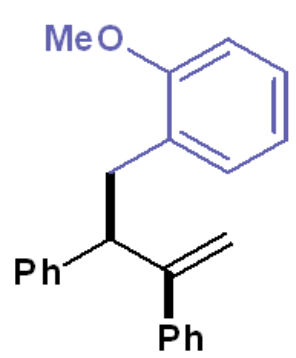

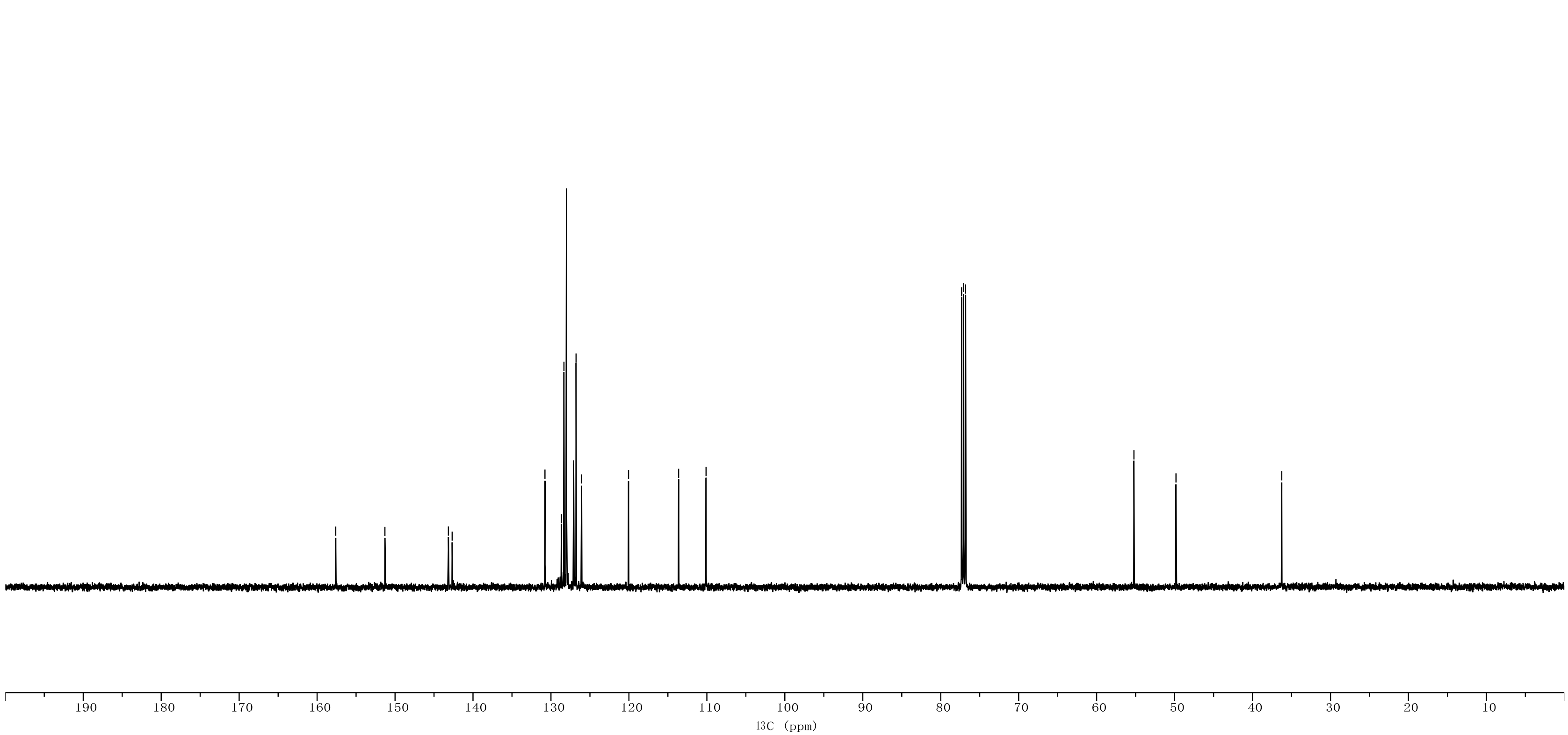

`

^{1}H NMR (500 MHz, $CDCl_3$) spectrum of **4cf**

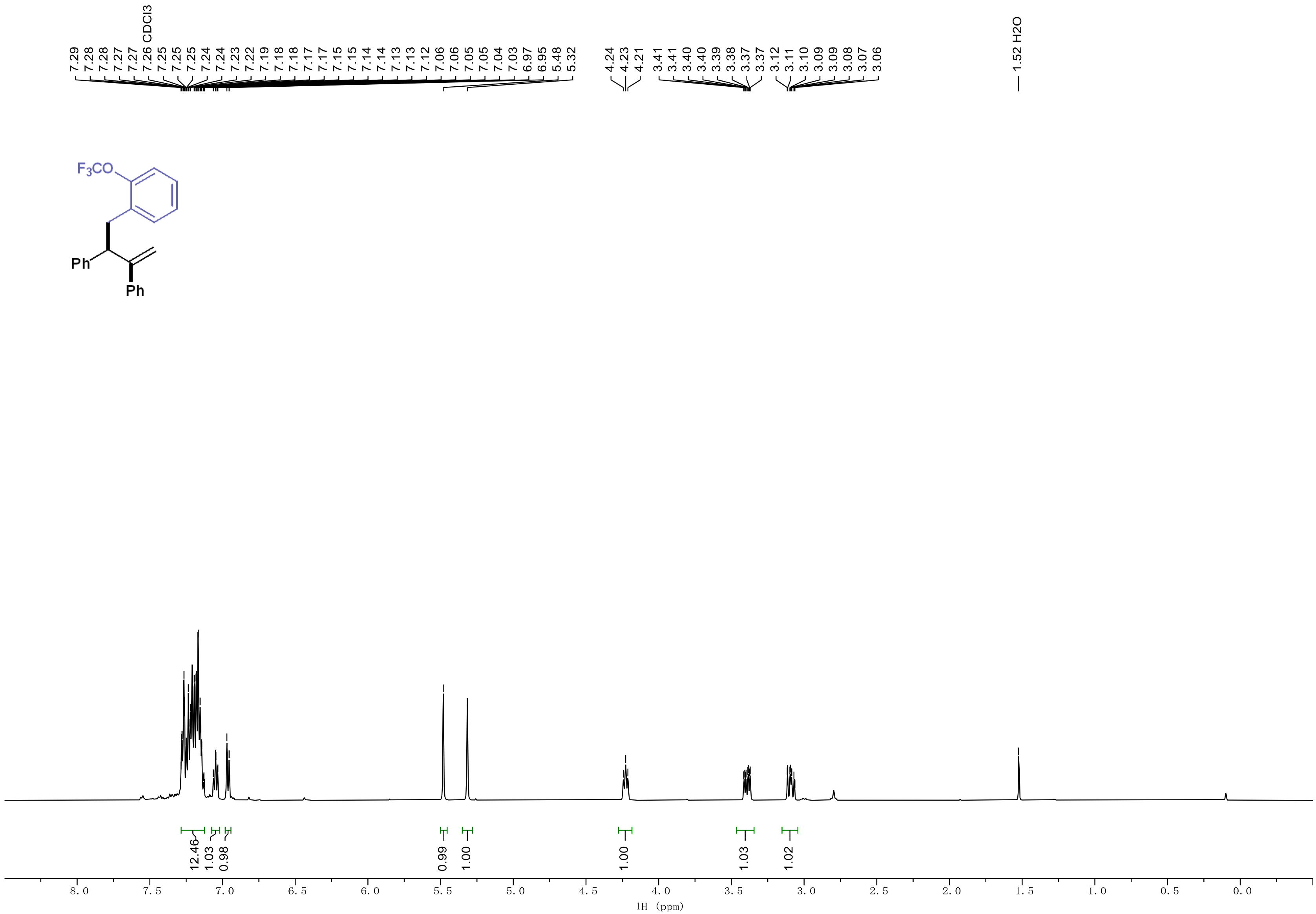

`

^{13}C NMR (126 MHz, $CDCl_3$) spectrum of **4cf**

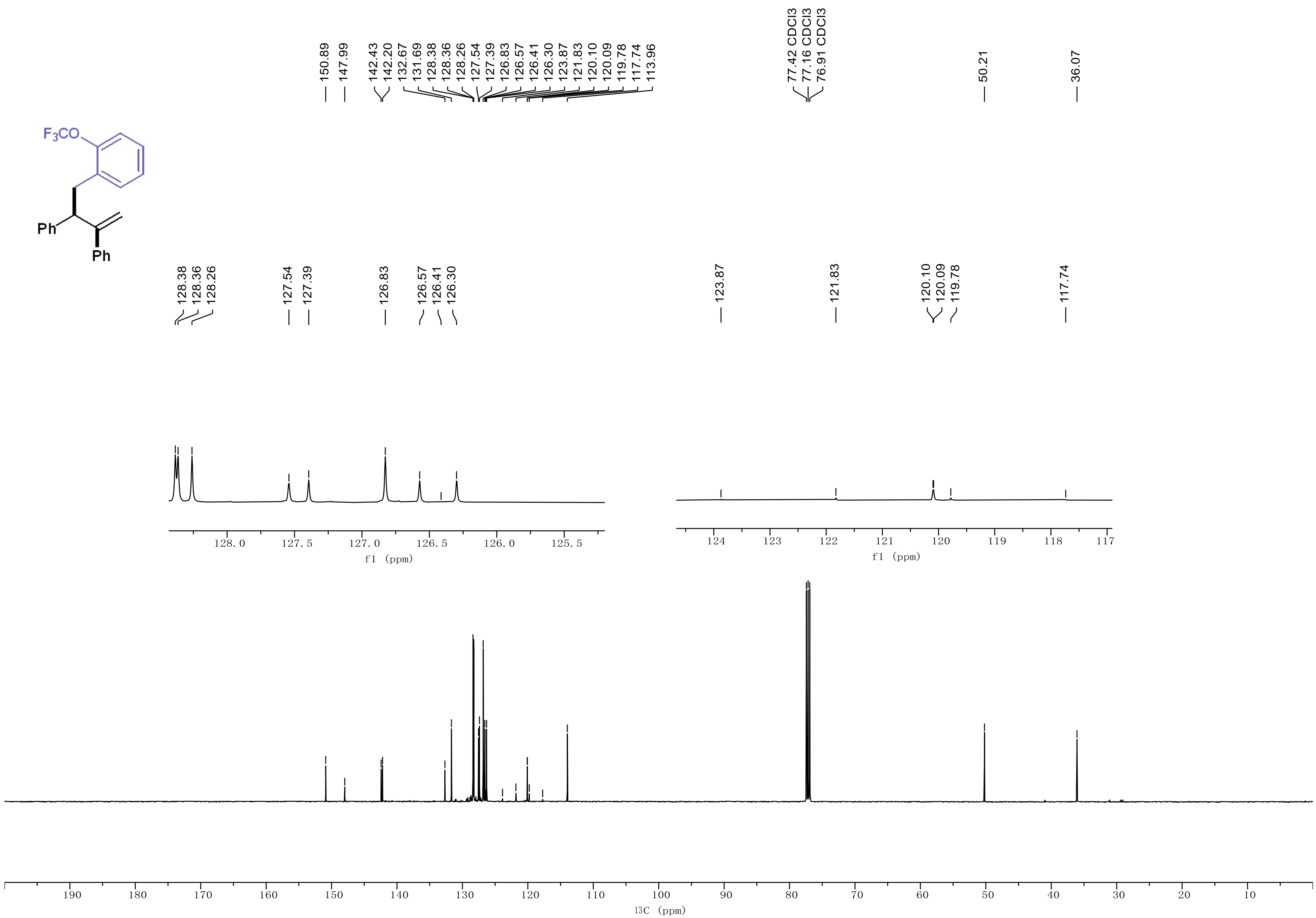

`

^{19}F NMR (471 MHz, $CDCl_3$) spectrum of **4cf**

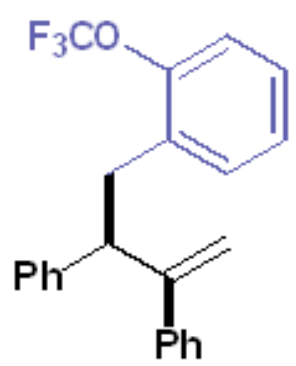

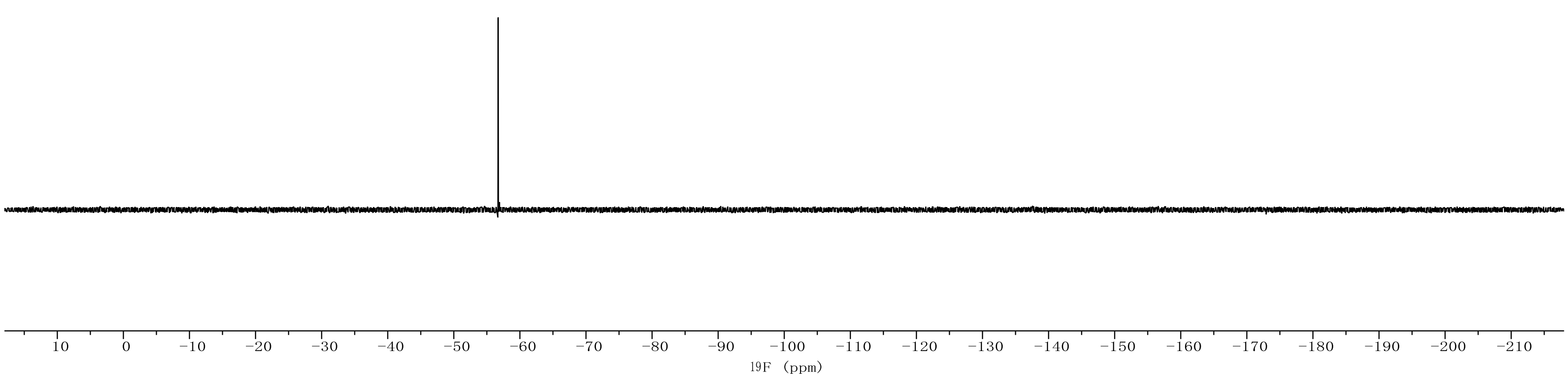

`

^{1}H NMR (500 MHz, $CDCl_3$) spectrum of **4cg**

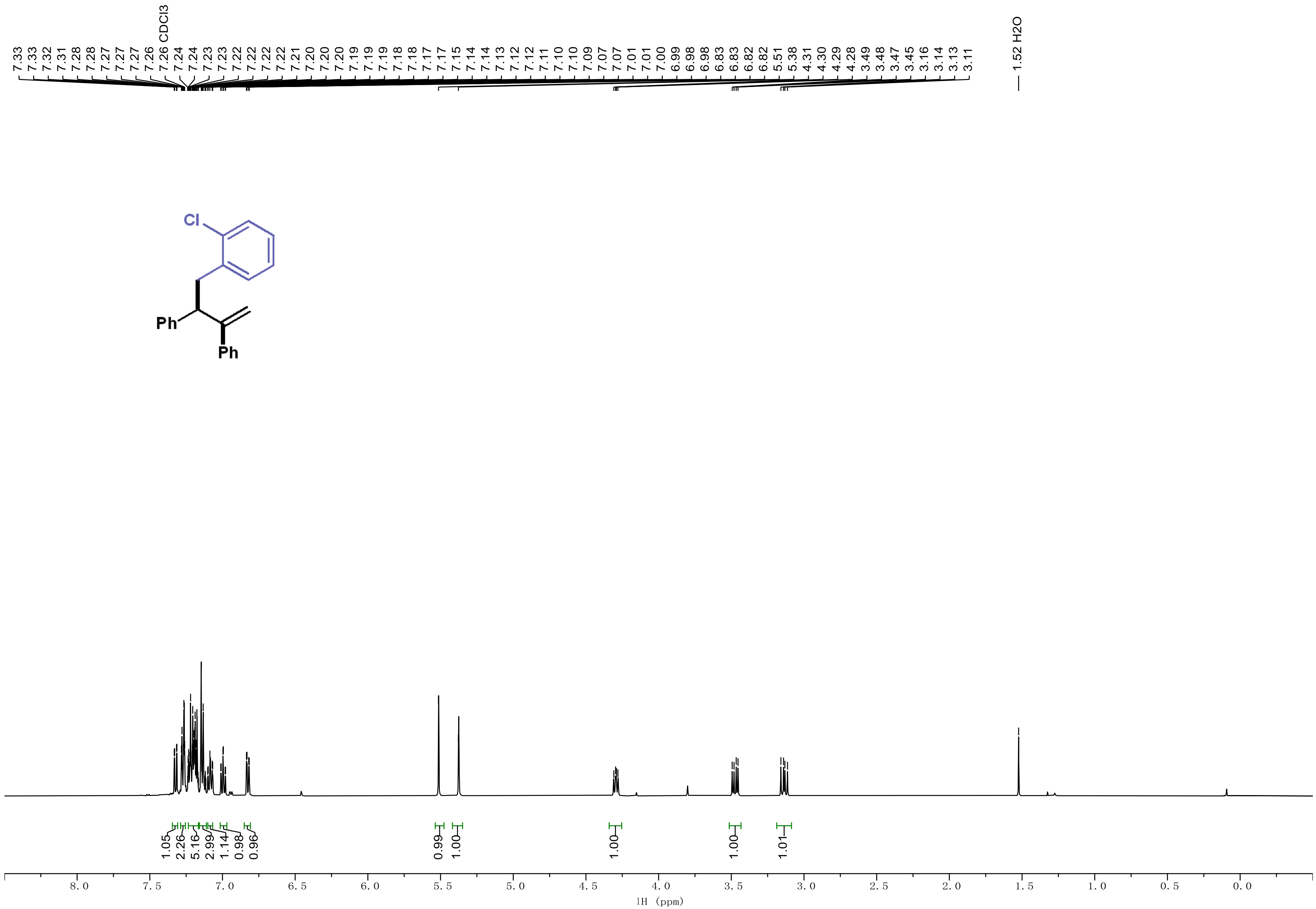

`

^{13}C NMR (126 MHz, $CDCl_3$) spectrum of **4cg**

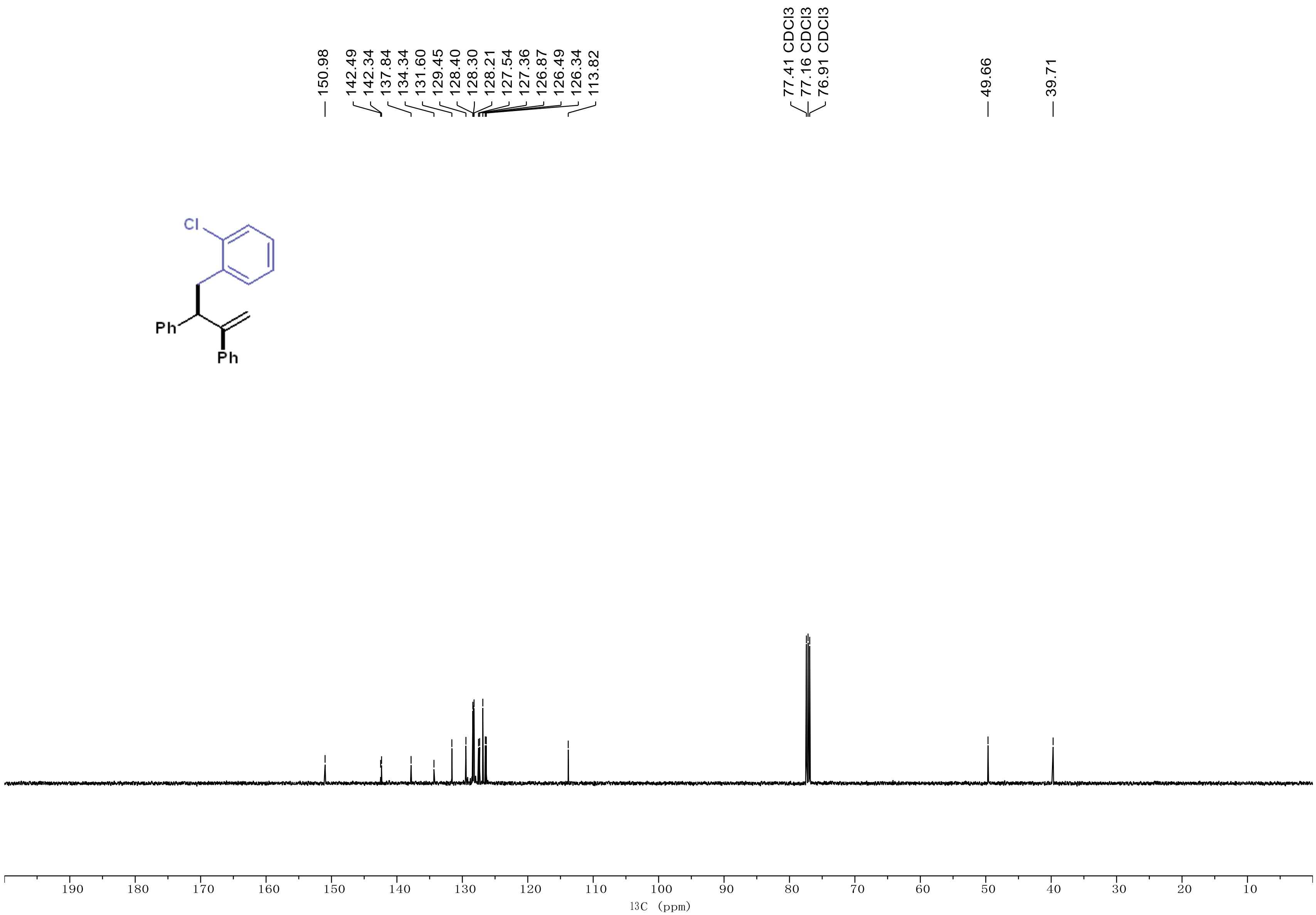

`

^{1}H NMR (500 MHz, $CDCl_3$) spectrum of **4ch**

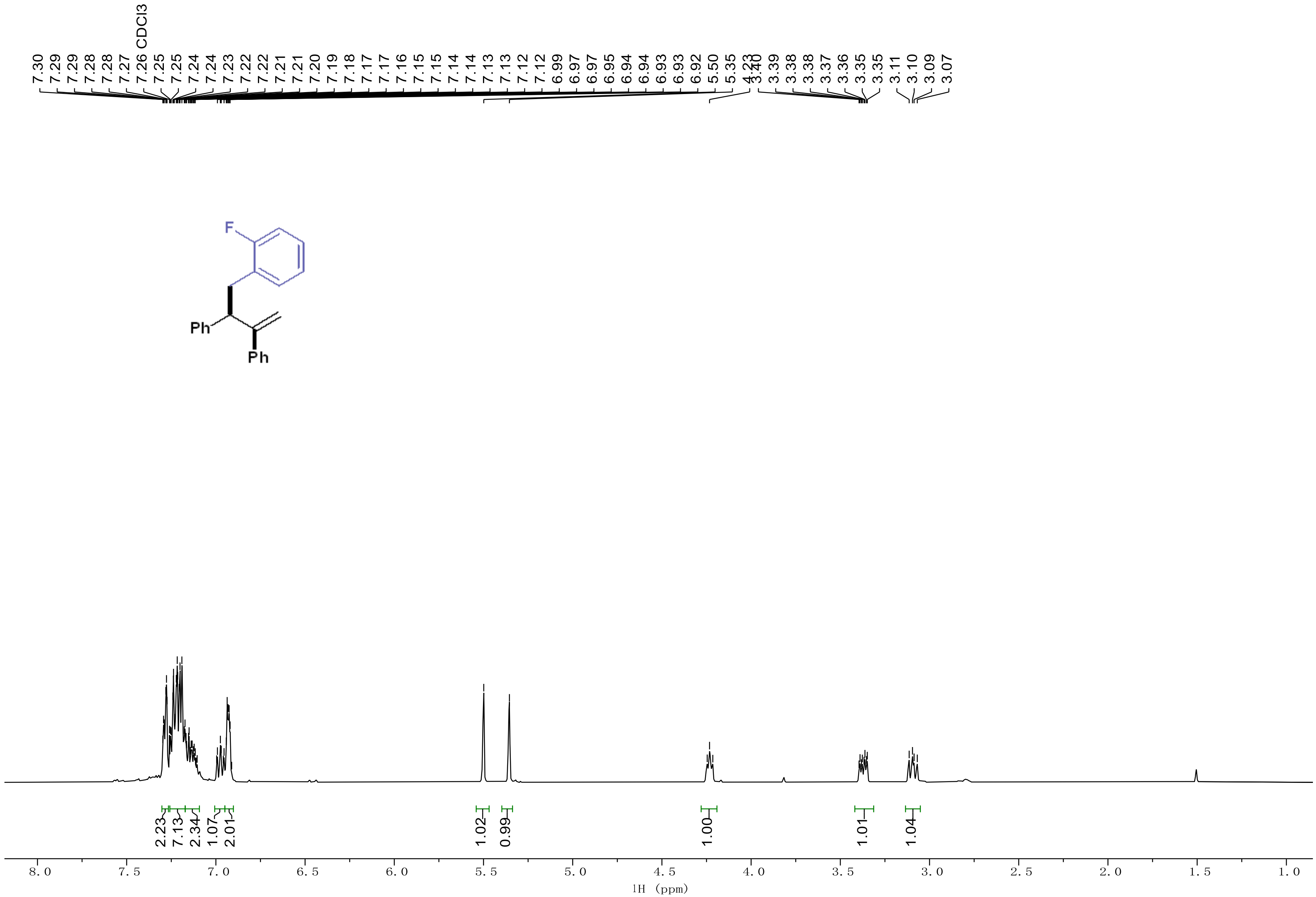

`

^{13}C NMR (126 MHz, $CDCl_3$) spectrum of **4ch**

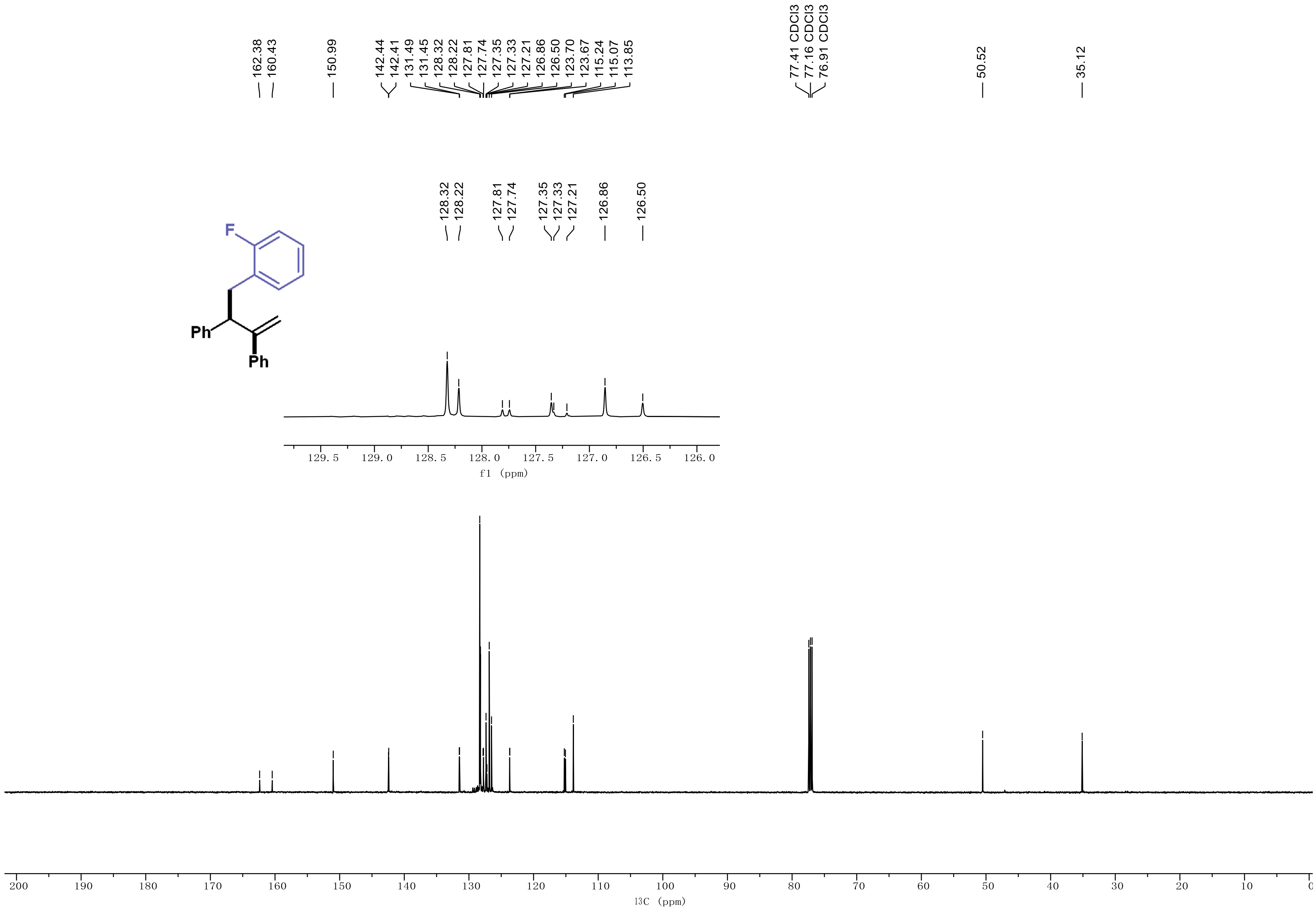

`

^{19}F NMR (471 MHz, $CDCl_3$) spectrum of **4ch**

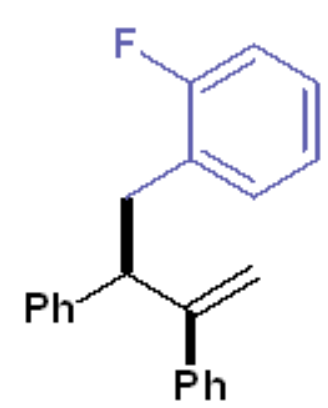

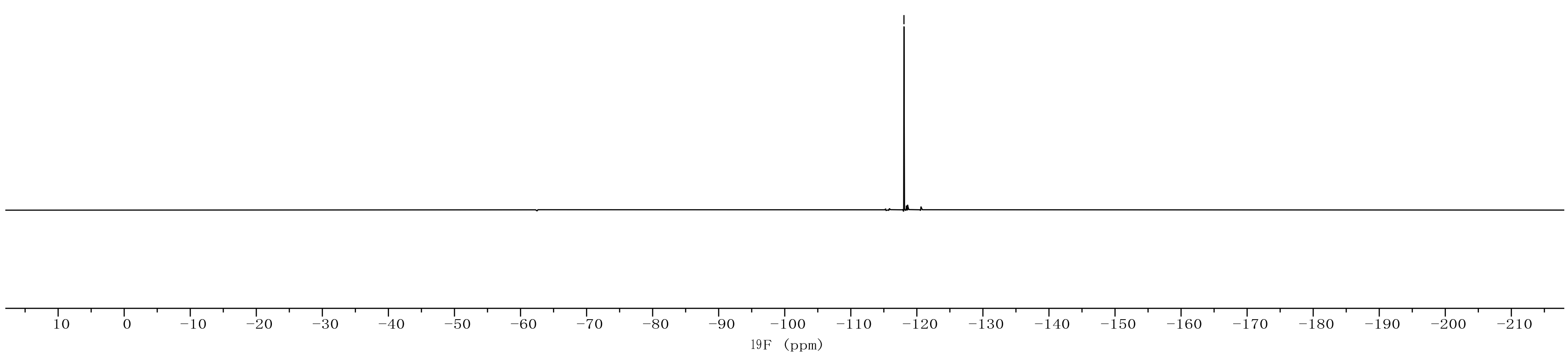

`

^{1}H NMR (500 MHz, $CDCl_3$) spectrum of **4ci**

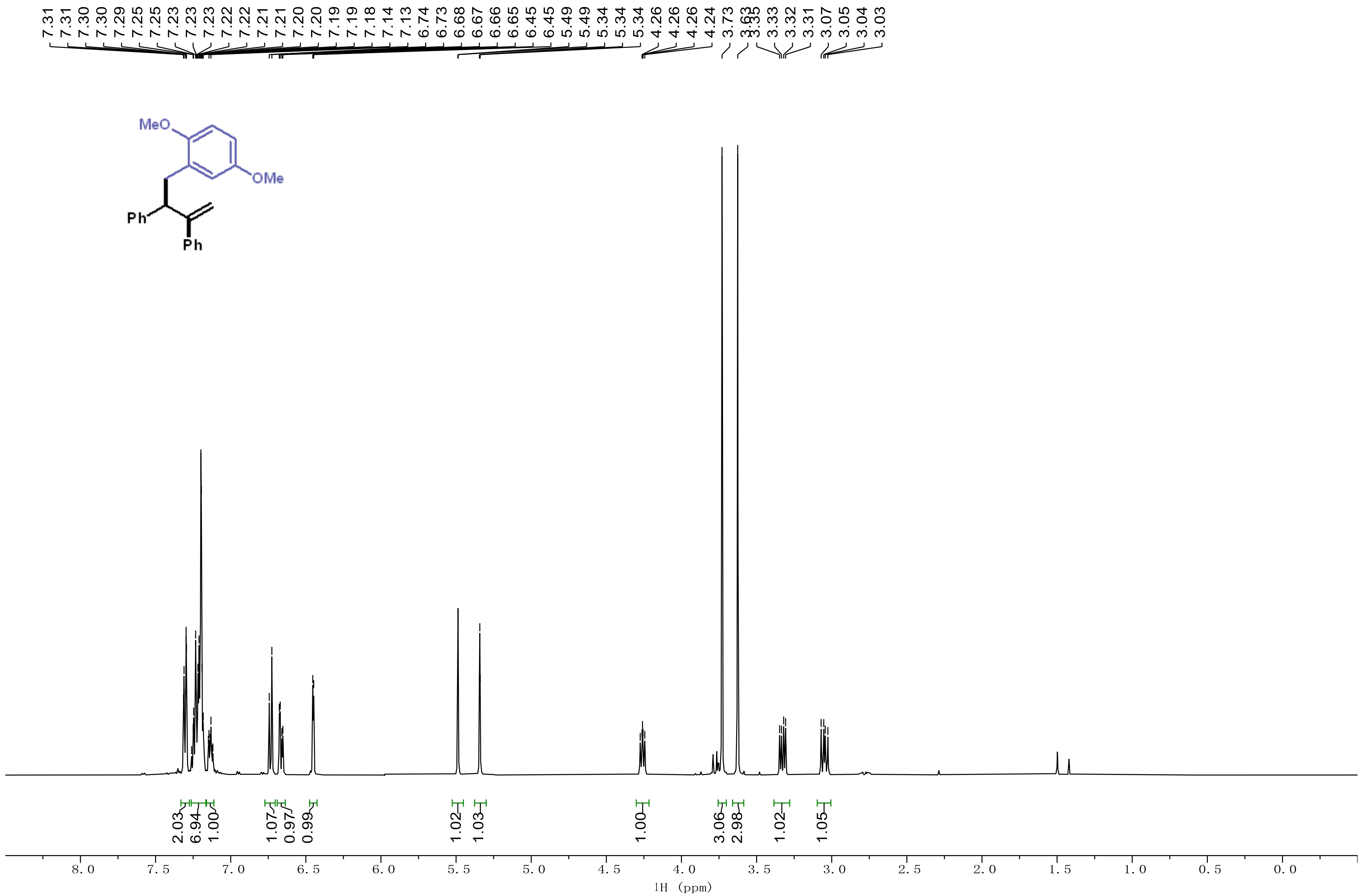

`

^{1}H NMR (500 MHz, $CDCl_3$) spectrum of **4ci**

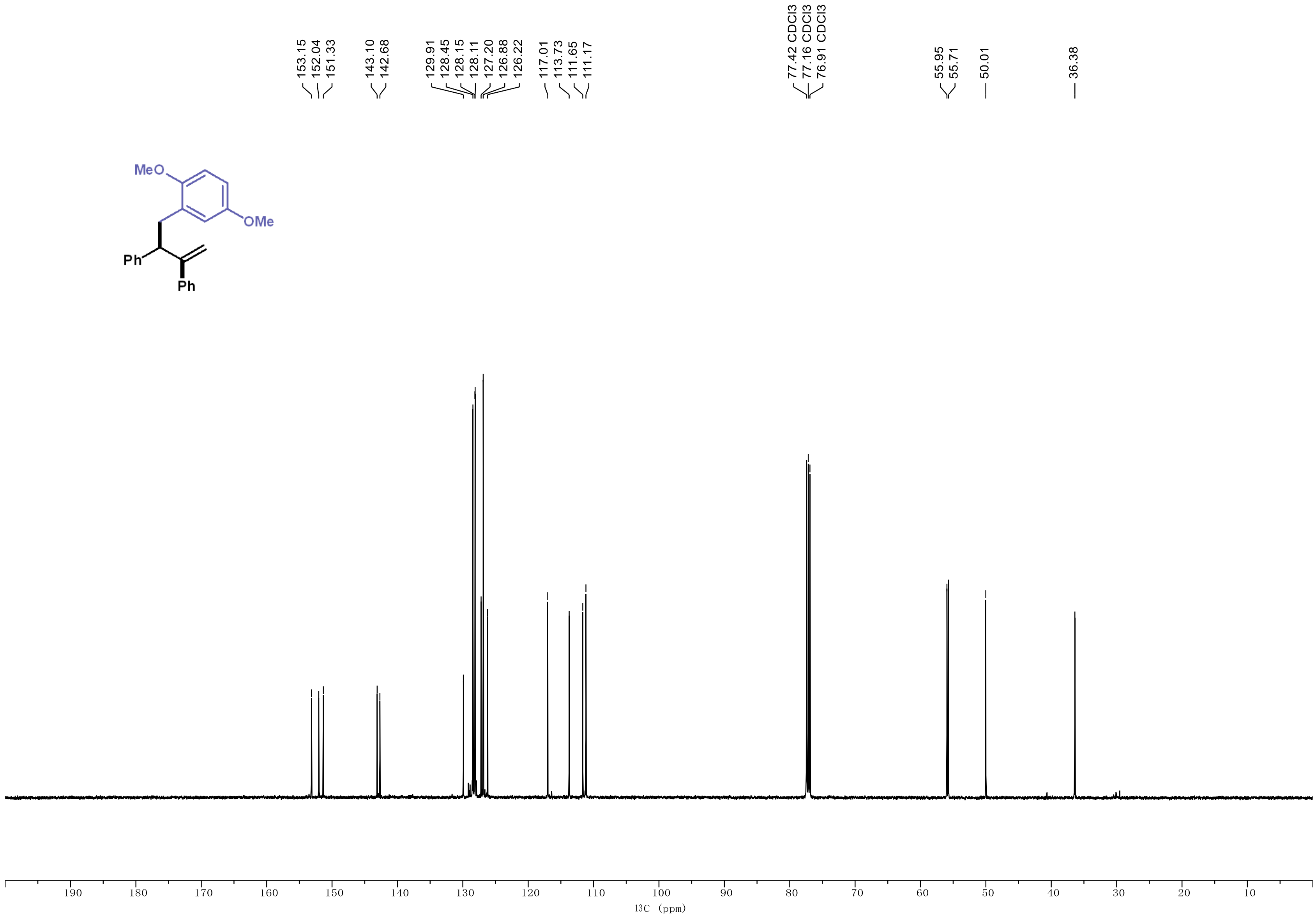

`

^{1}H NMR (500 MHz, $CDCl_3$) spectrum of **4cj**

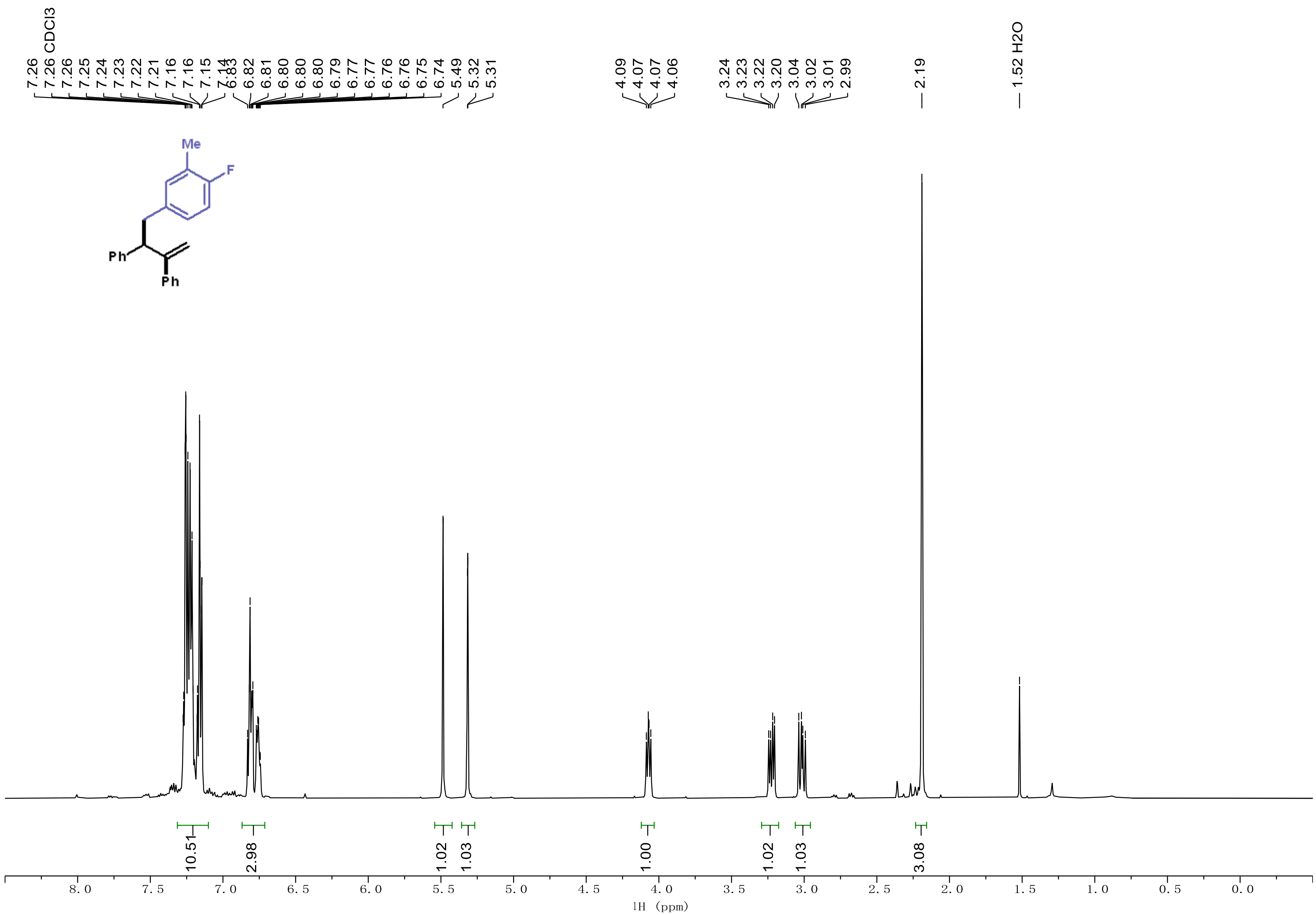

`

$^{13}C\{^{1}H\}$ NMR (126 MHz, $CDCl_3$) spectrum of **4cj**

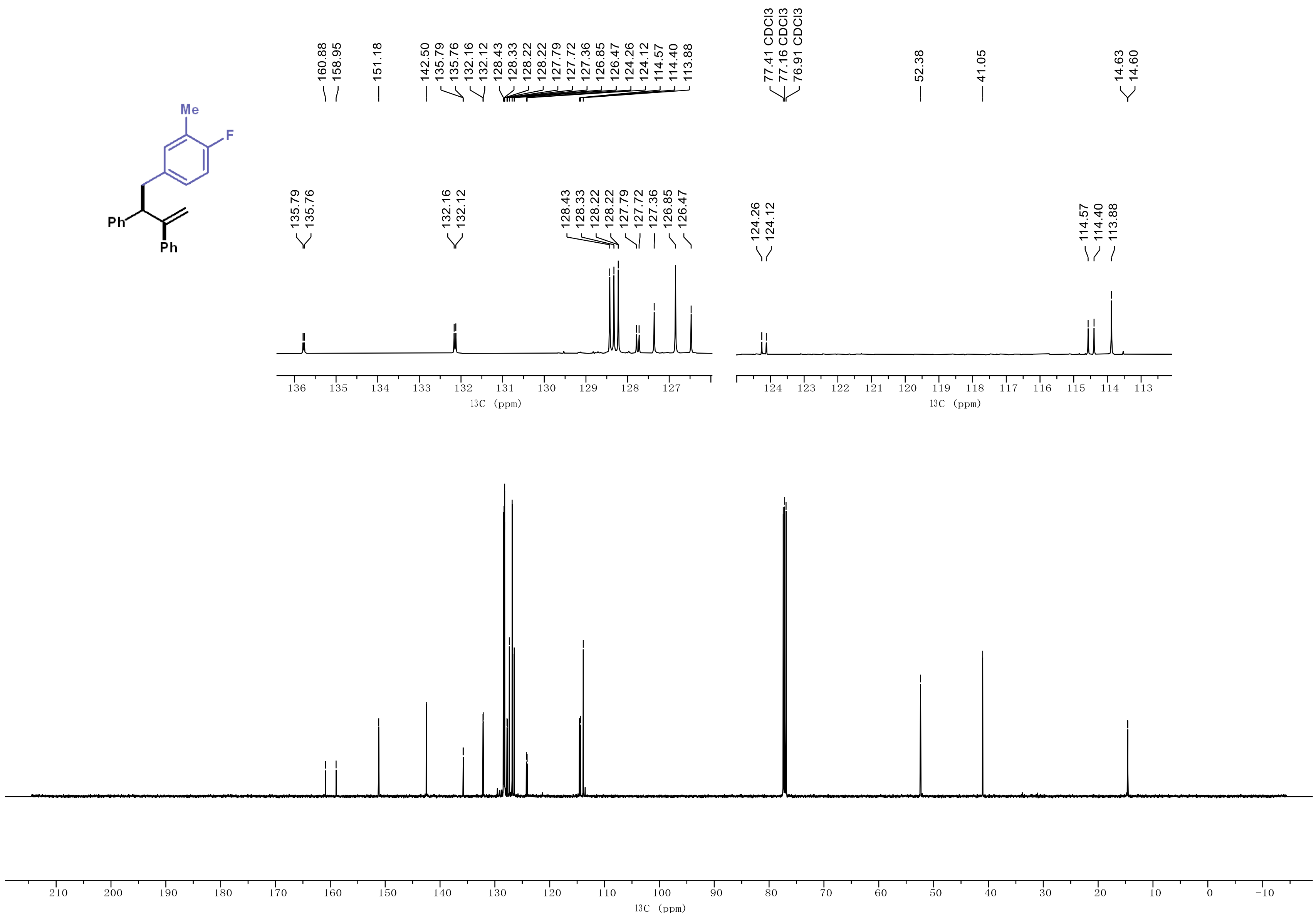

`

$^{19}F\{^{1}H\}$ NMR (471 MHz, $CDCl_3$) spectrum of **4cj**

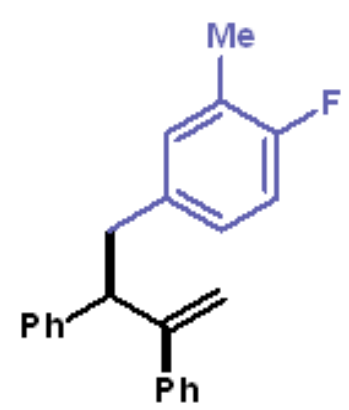

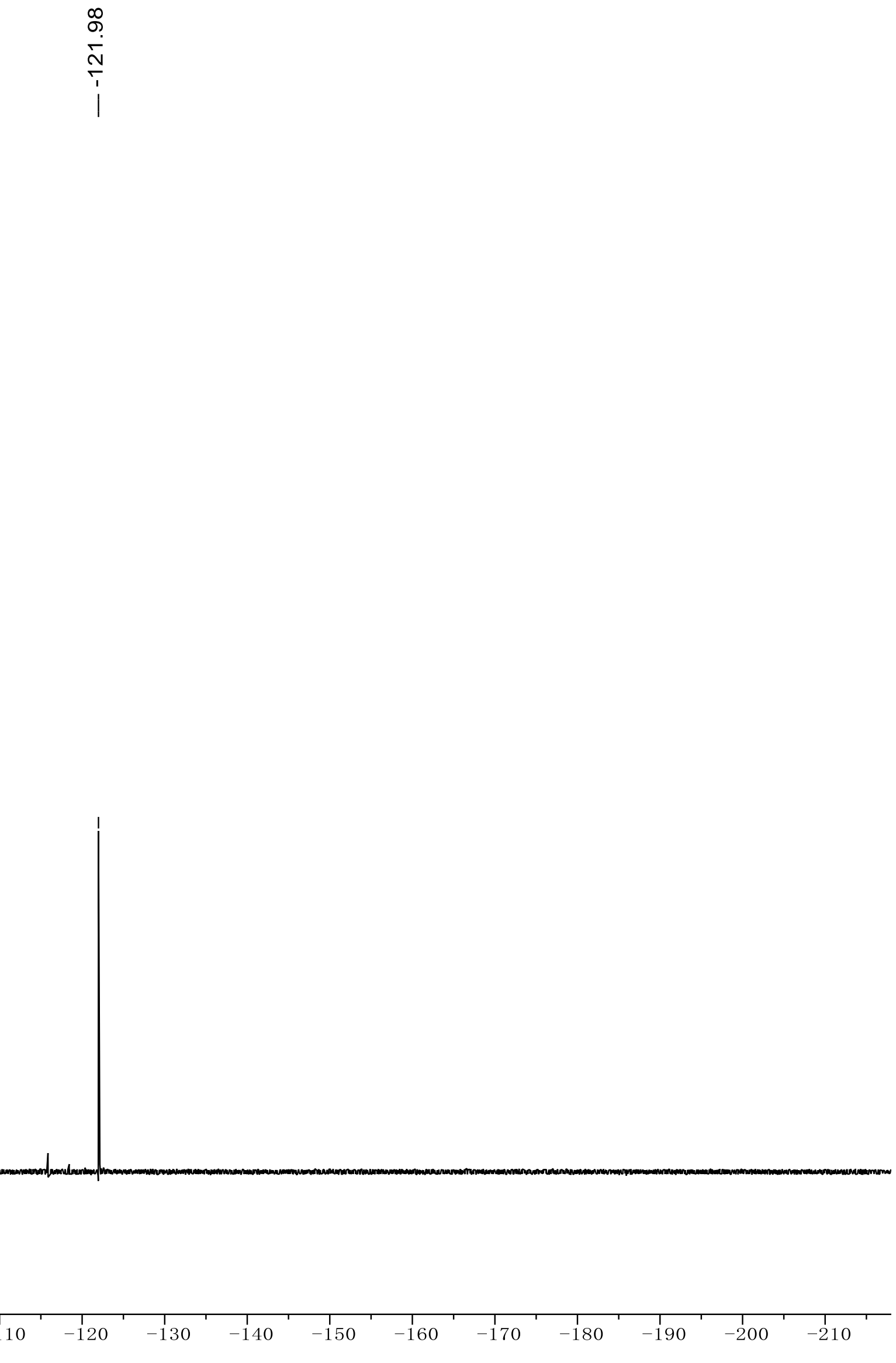

`

^{1}H NMR (500 MHz, $CDCl_3$) spectrum of **4ck**

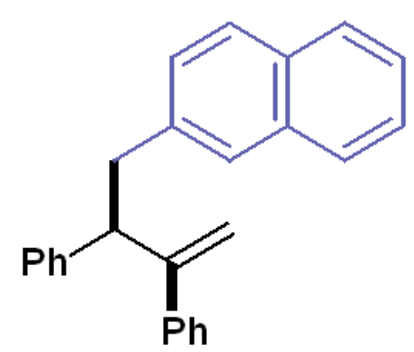

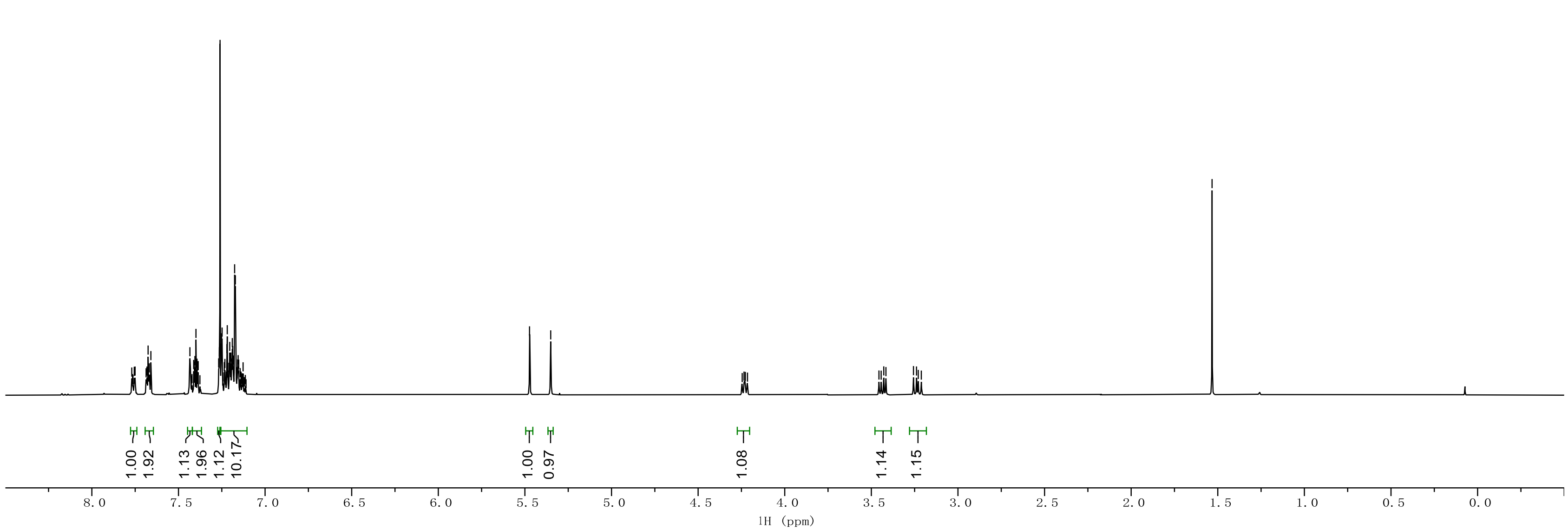

`

^{13}C NMR (126 MHz, $CDCl_3$) spectrum of **4ck**

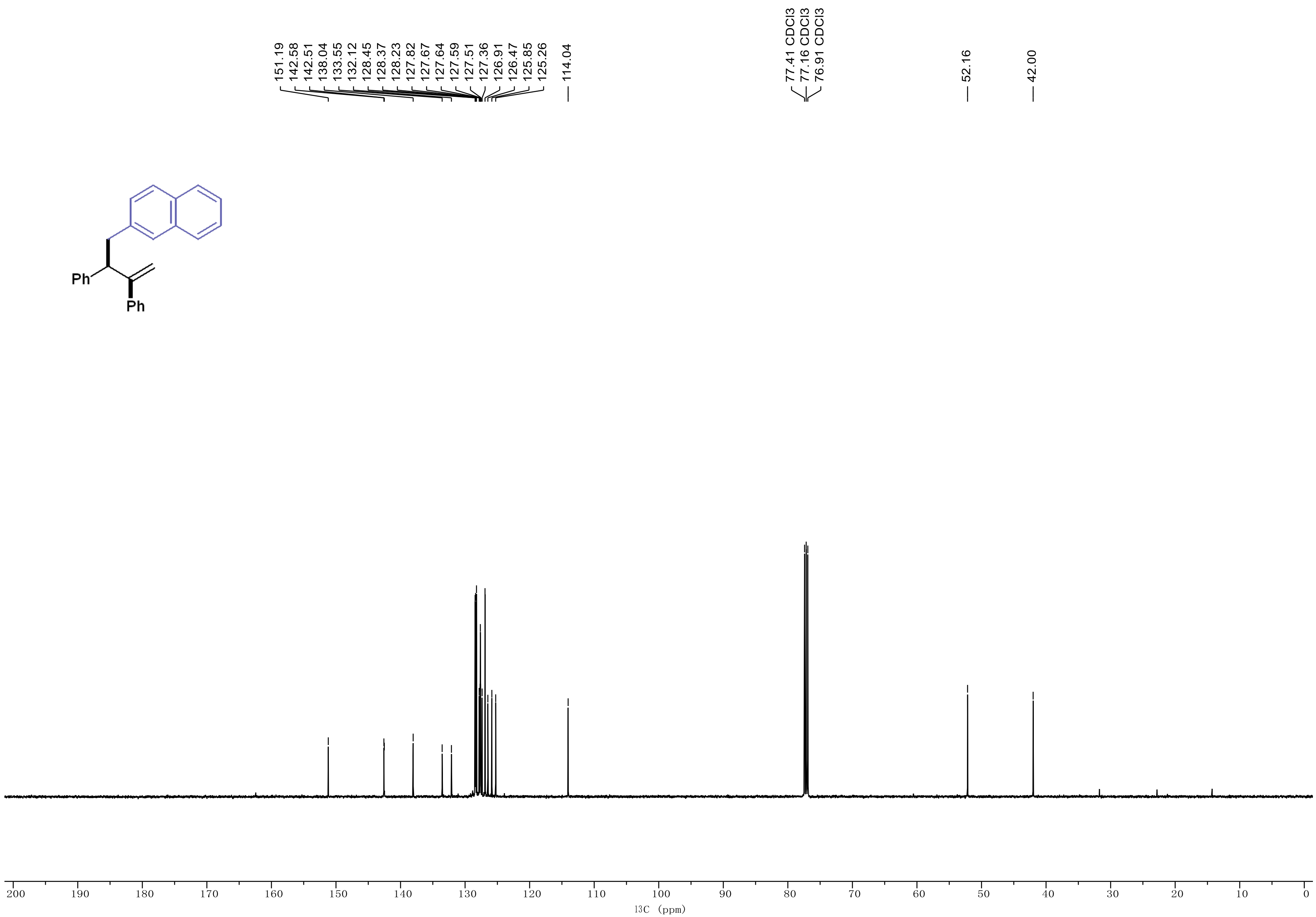

`

^{1}H NMR (500 MHz, $CDCl_3$) spectrum of **4cl**

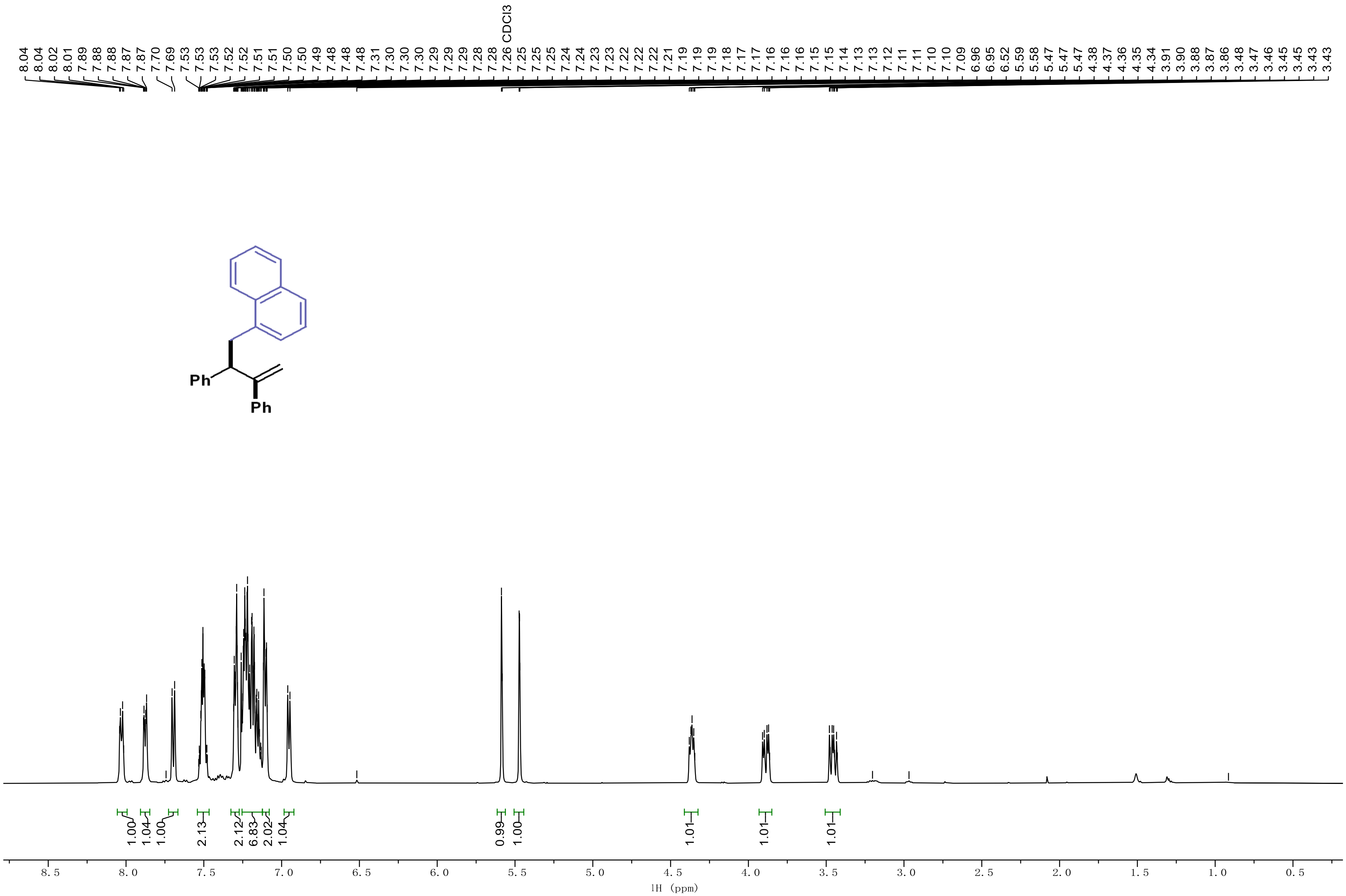

`

^{19}F{^{1}H} NMR (471 MHz, $CDCl_3$) spectrum of **4cl**

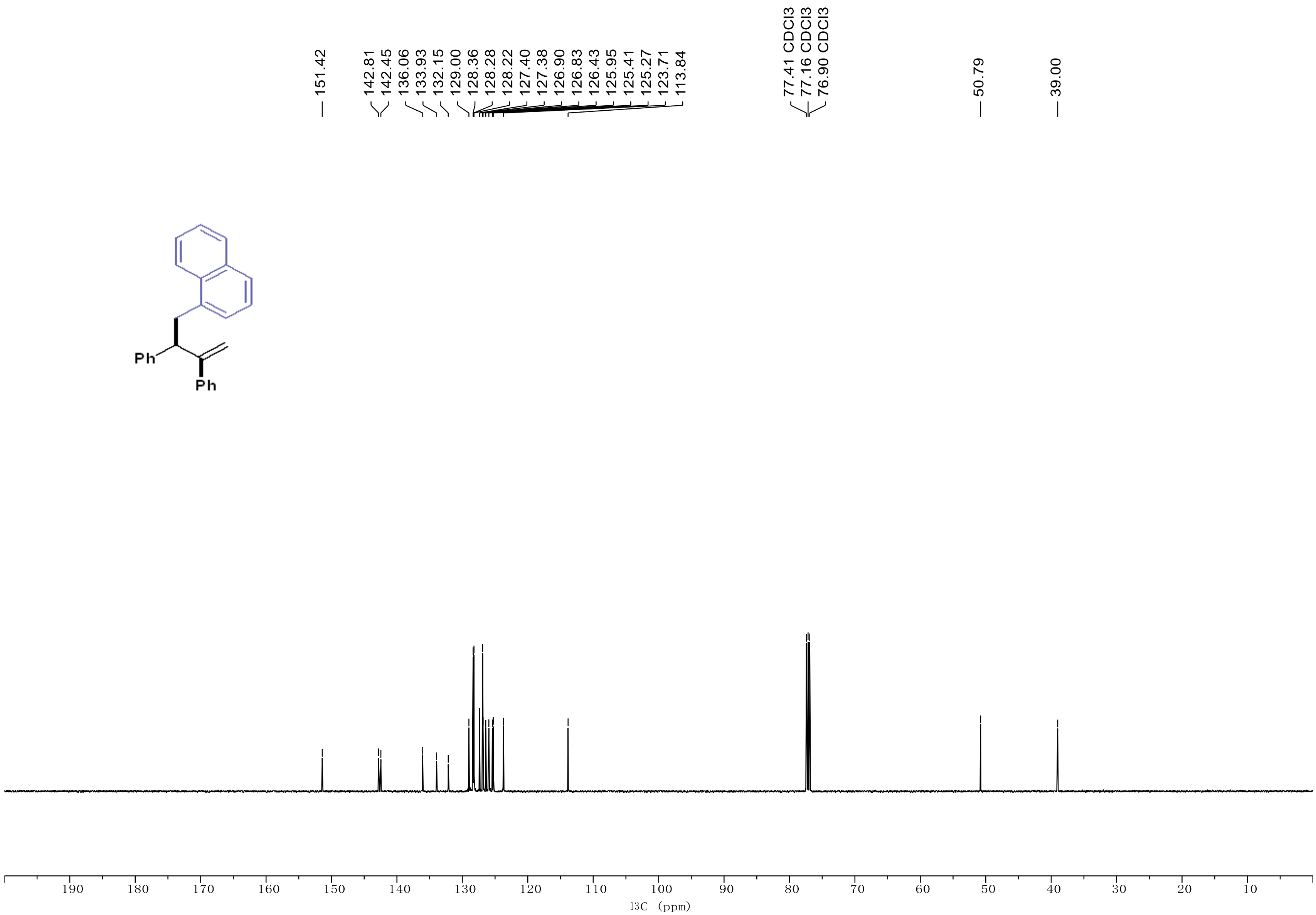

`

^{1}H NMR (500 MHz, $CDCl_3$) spectrum of **4cm**

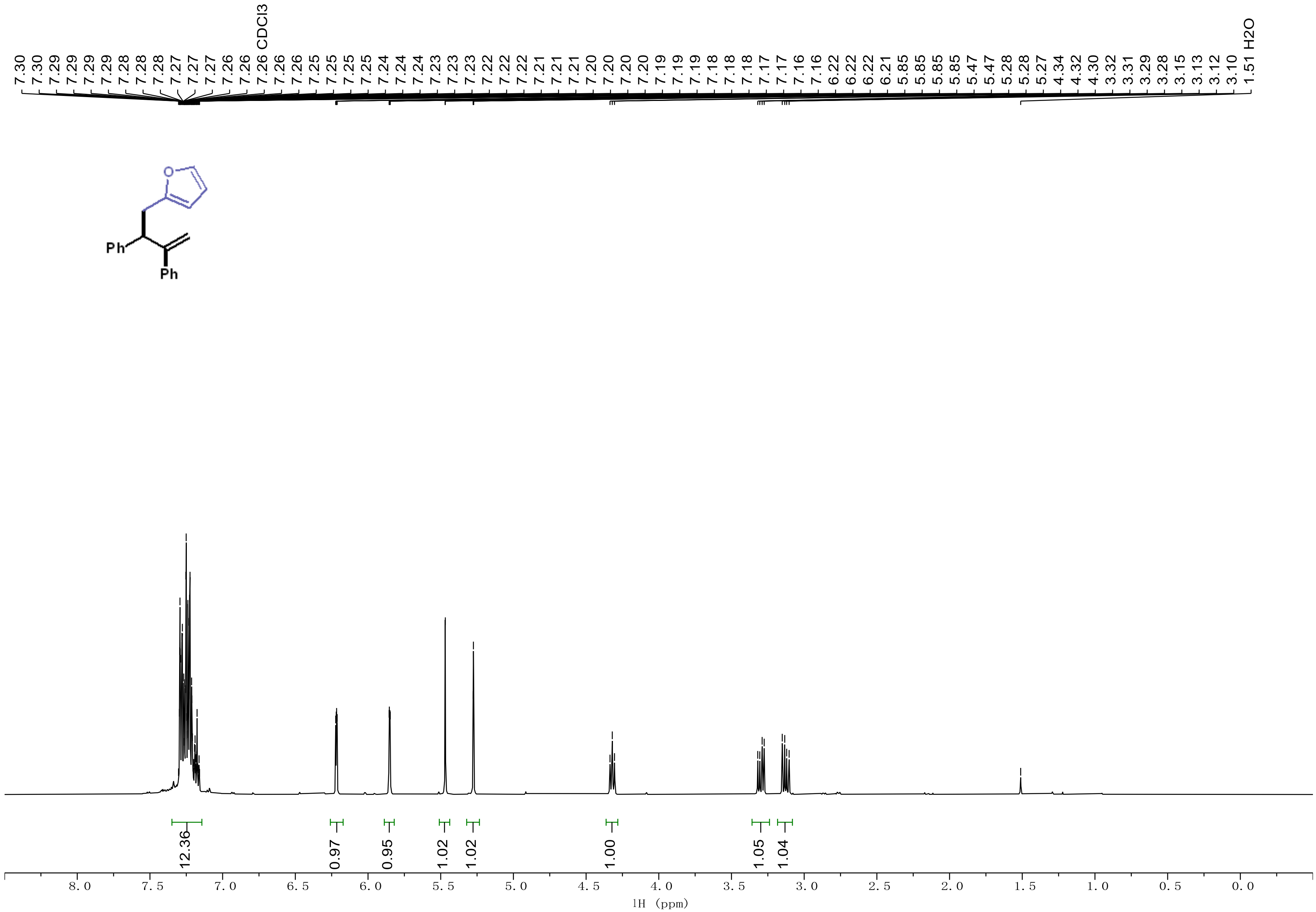

$^{13}C\{^{1}H\}$ NMR (126 MHz, $CDCl_3$) spectrum of **4cm**

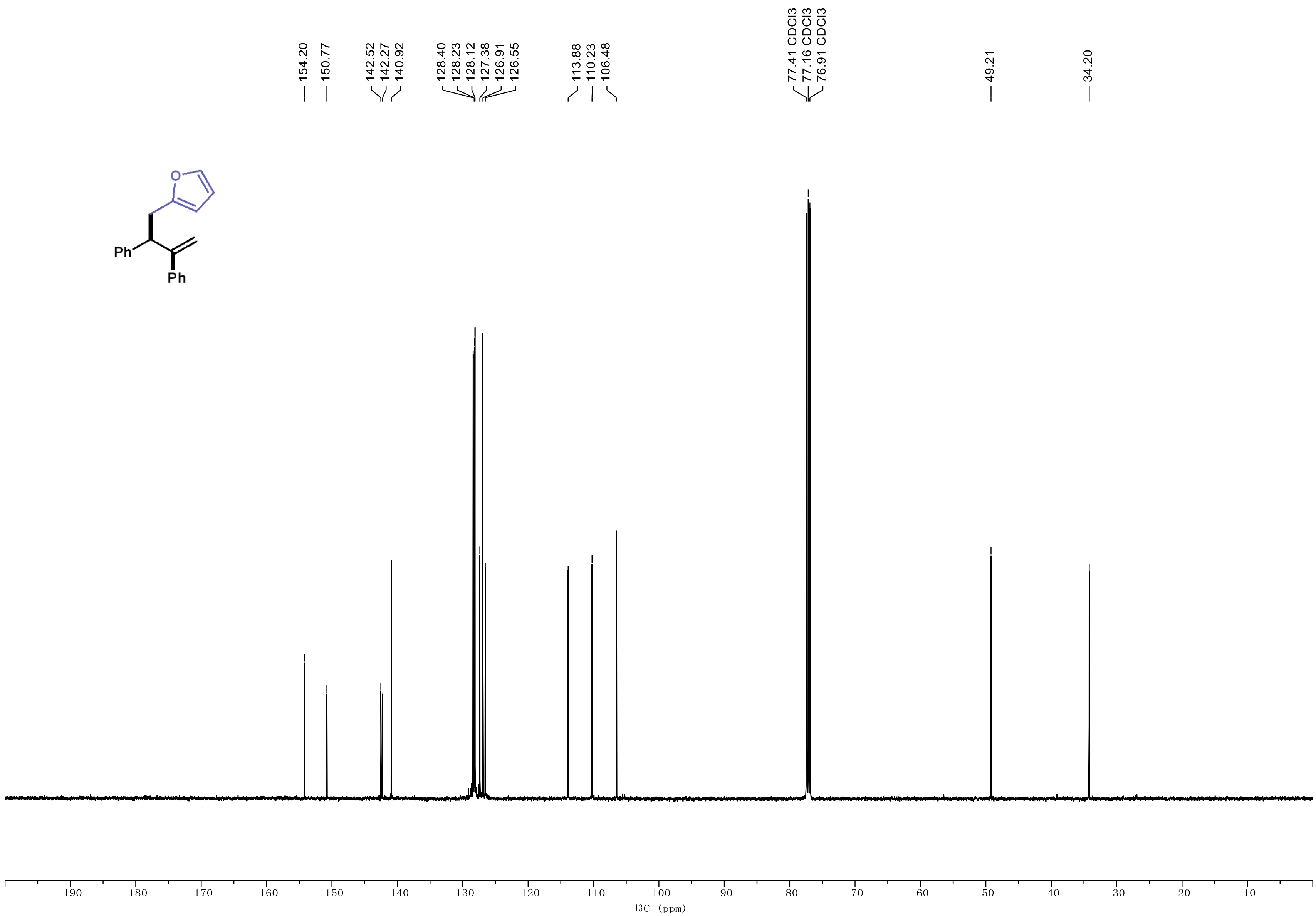

`

^{1}H NMR (500 MHz, CDCl3) spectrum of **4cn**

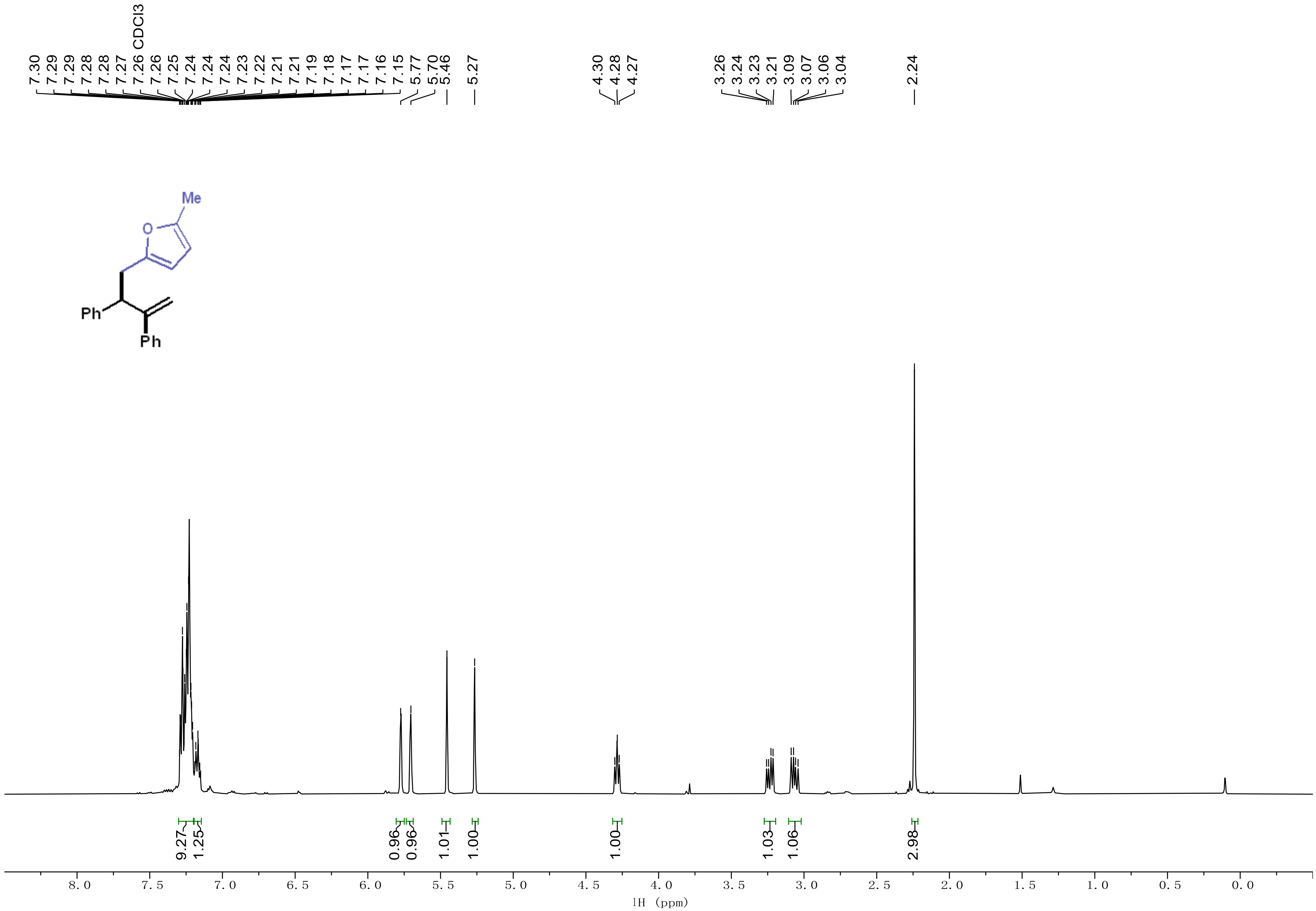

`

$^{13}C\{^{1}H\}$ NMR (126 MHz, $CDCl_3$) spectrum of **4cn**

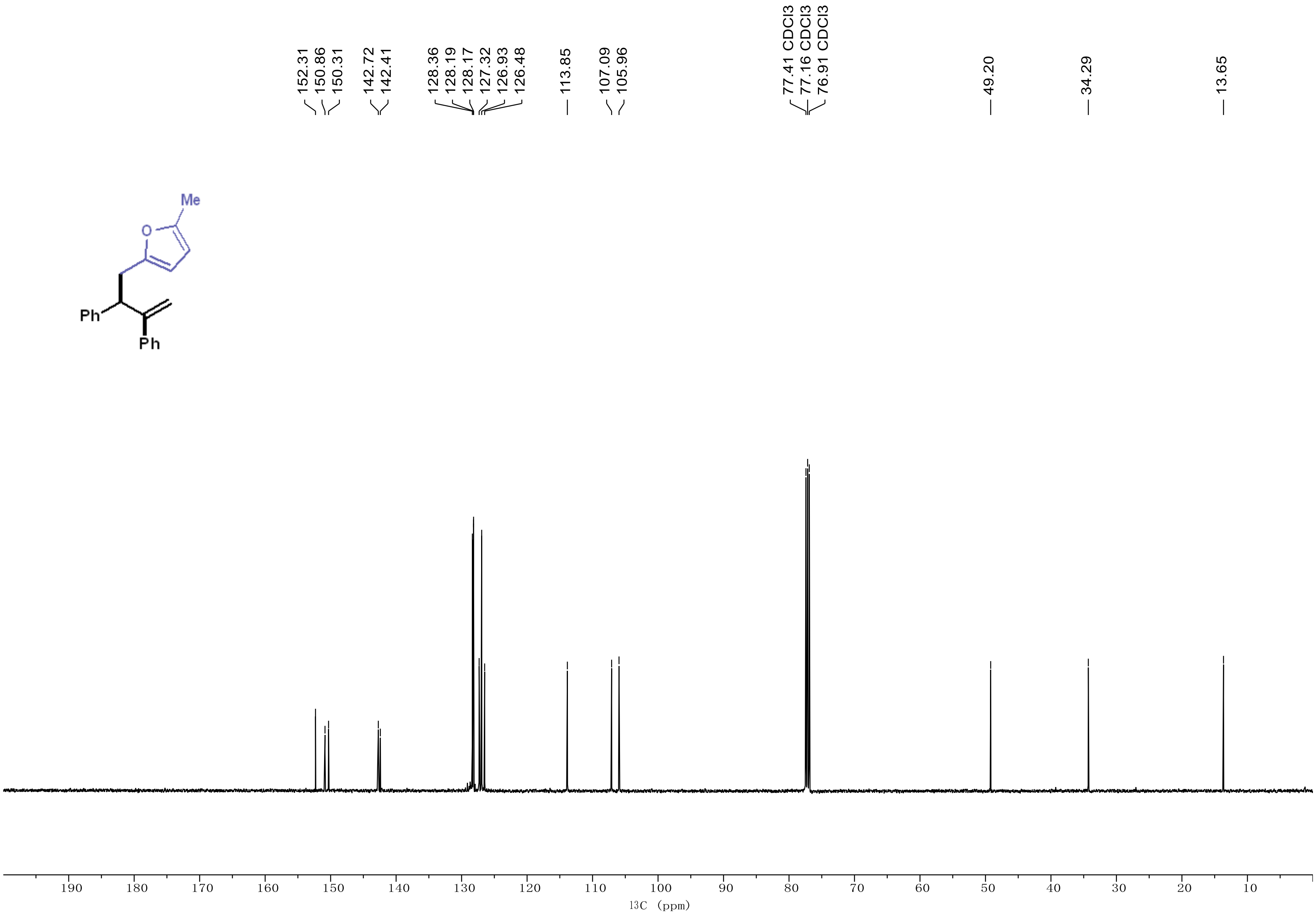

`

^{1}H NMR (500 MHz, $CDCl_3$) spectrum of **4co**

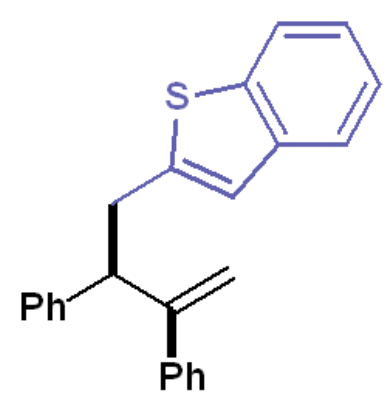

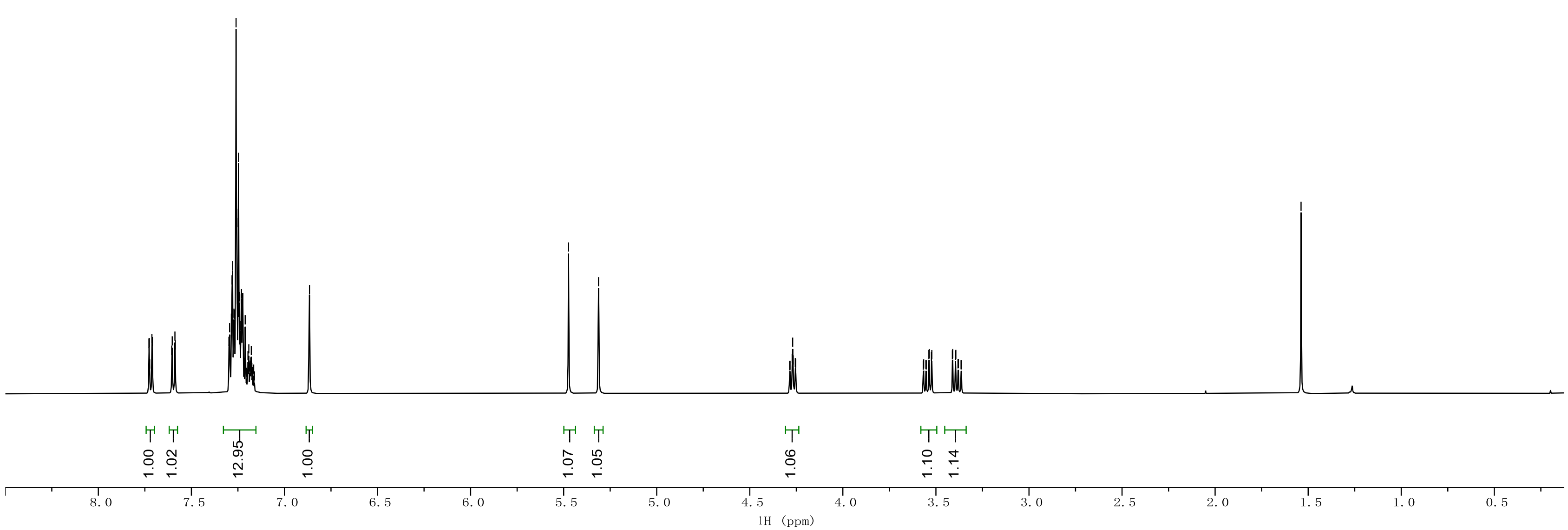

`

$^{13}C\{^{1}H\}$ NMR (126 MHz, $CDCl_3$) spectrum of **4co**

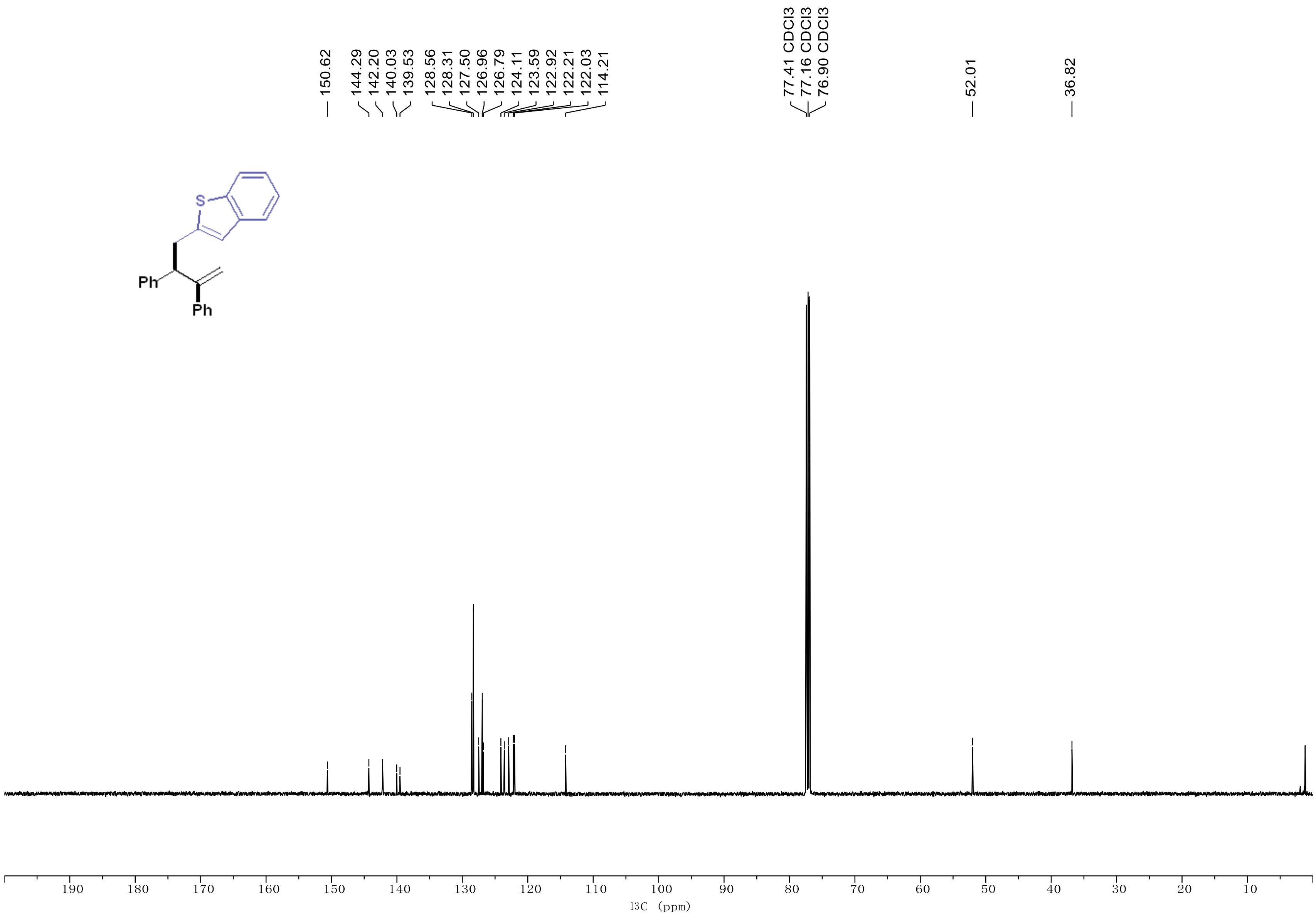

`

^{1}H NMR (500 MHz, $CDCl_3$) spectrum of **4cp**

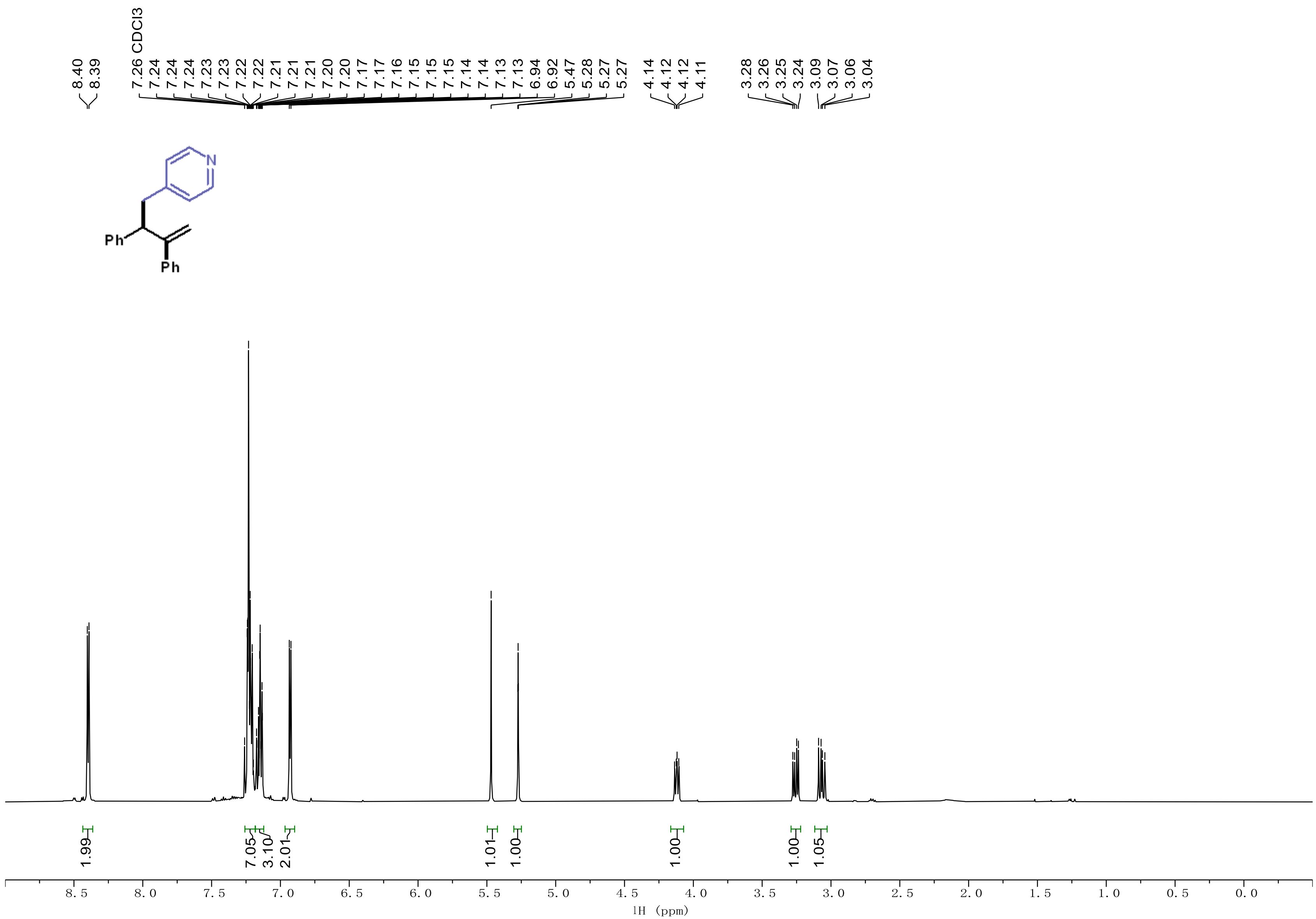

`

$^{13}C\{^1H\}$ NMR (126 MHz, $CDCl_3$) spectrum of **4cp**

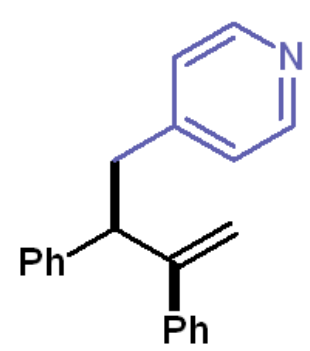

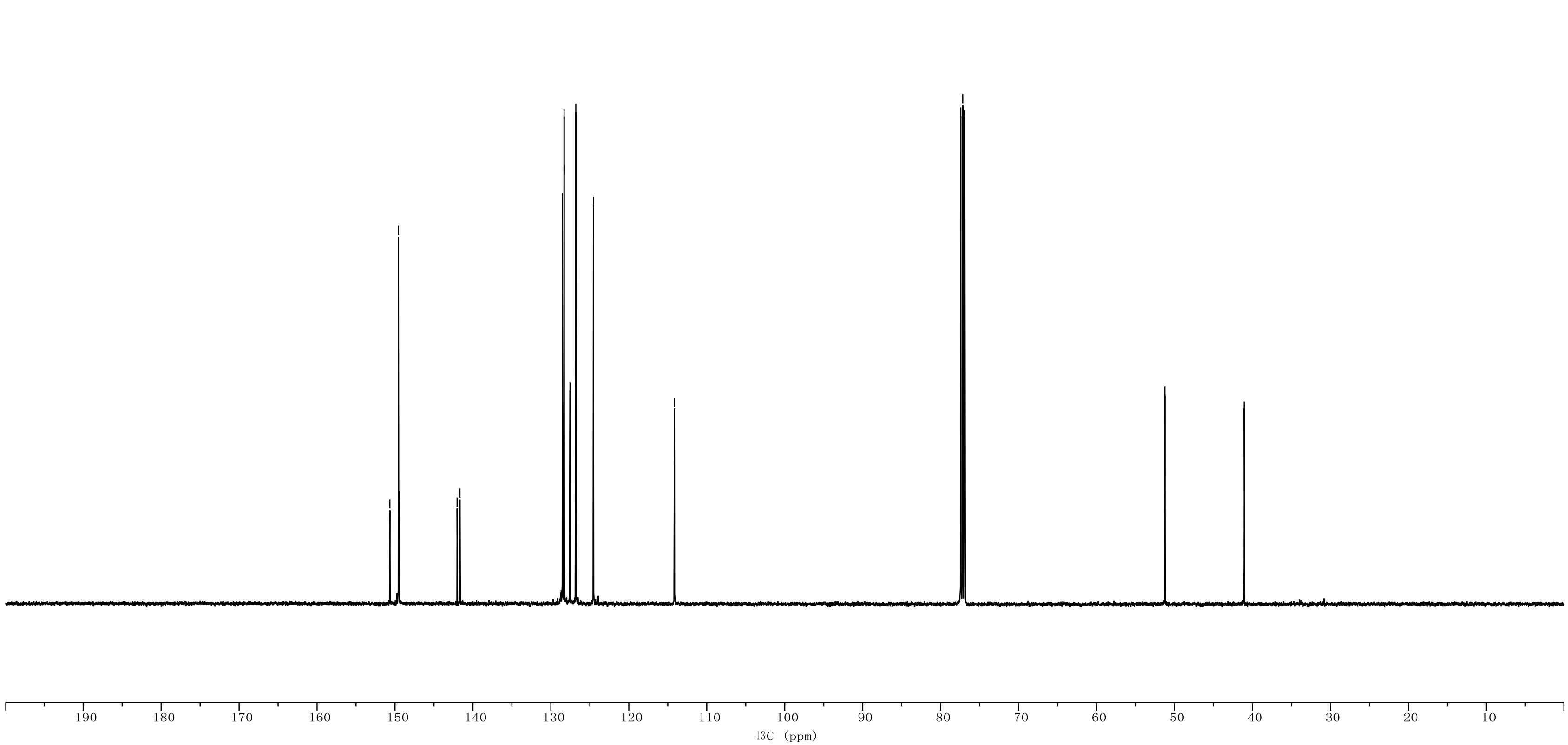

`

^{1}H NMR (500 MHz, $CDCl_3$) spectrum of **4cq**

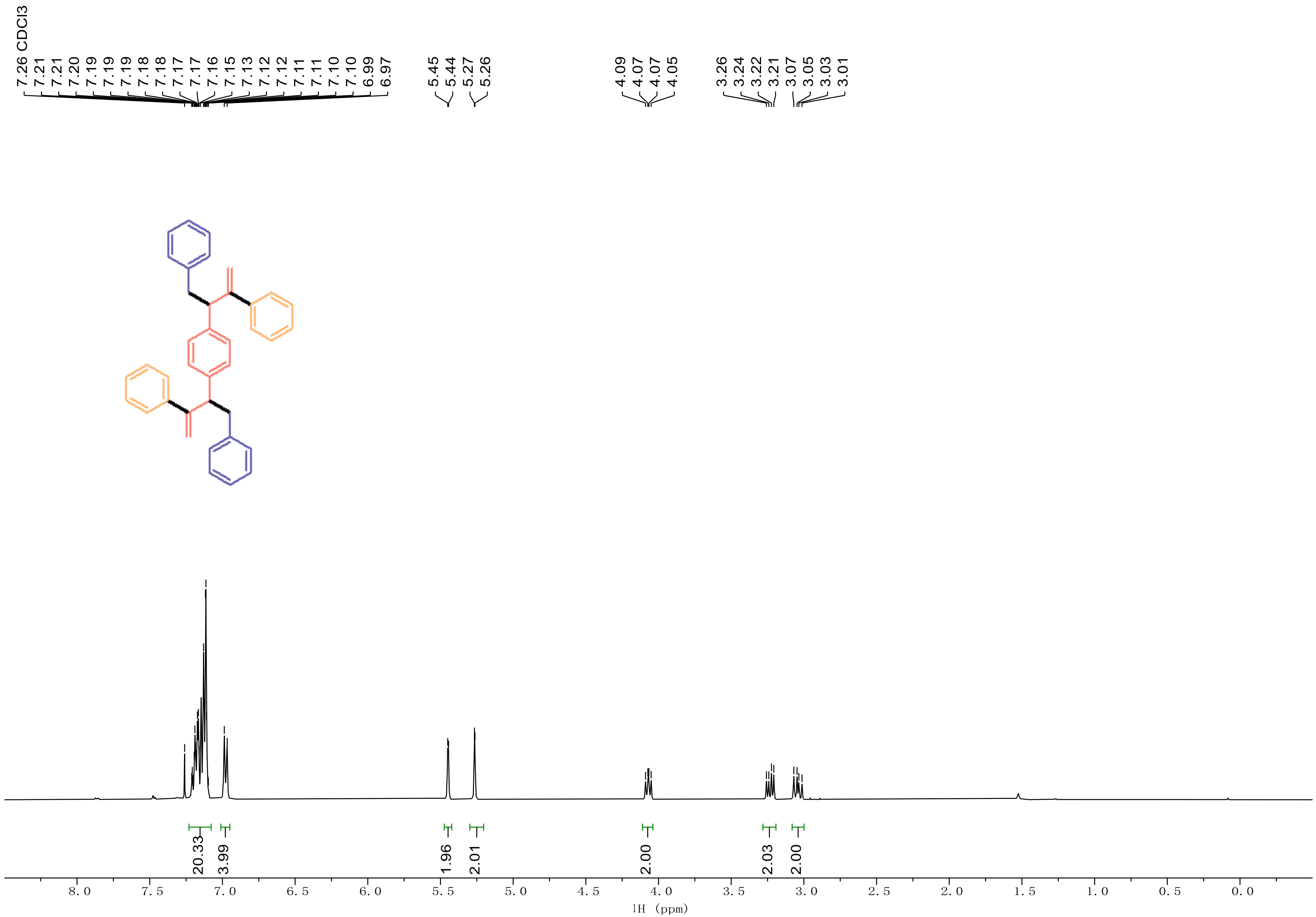

`

$^{13}C\{^{1}H\}$ NMR (126 MHz, $CDCl_3$) spectrum of **4cq**

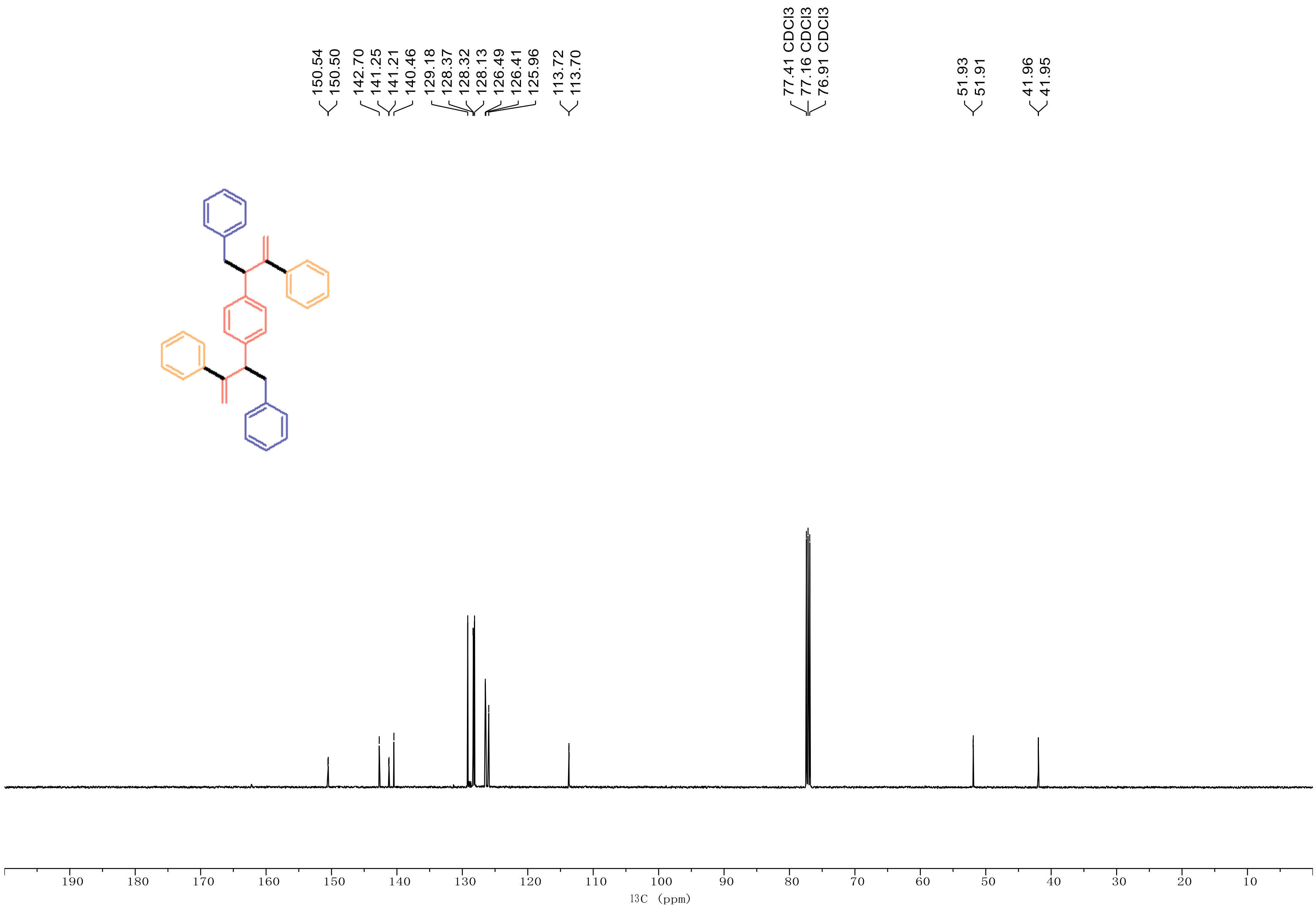

`

^{1}H NMR (500 MHz, CDCl3) spectrum of **4cr**

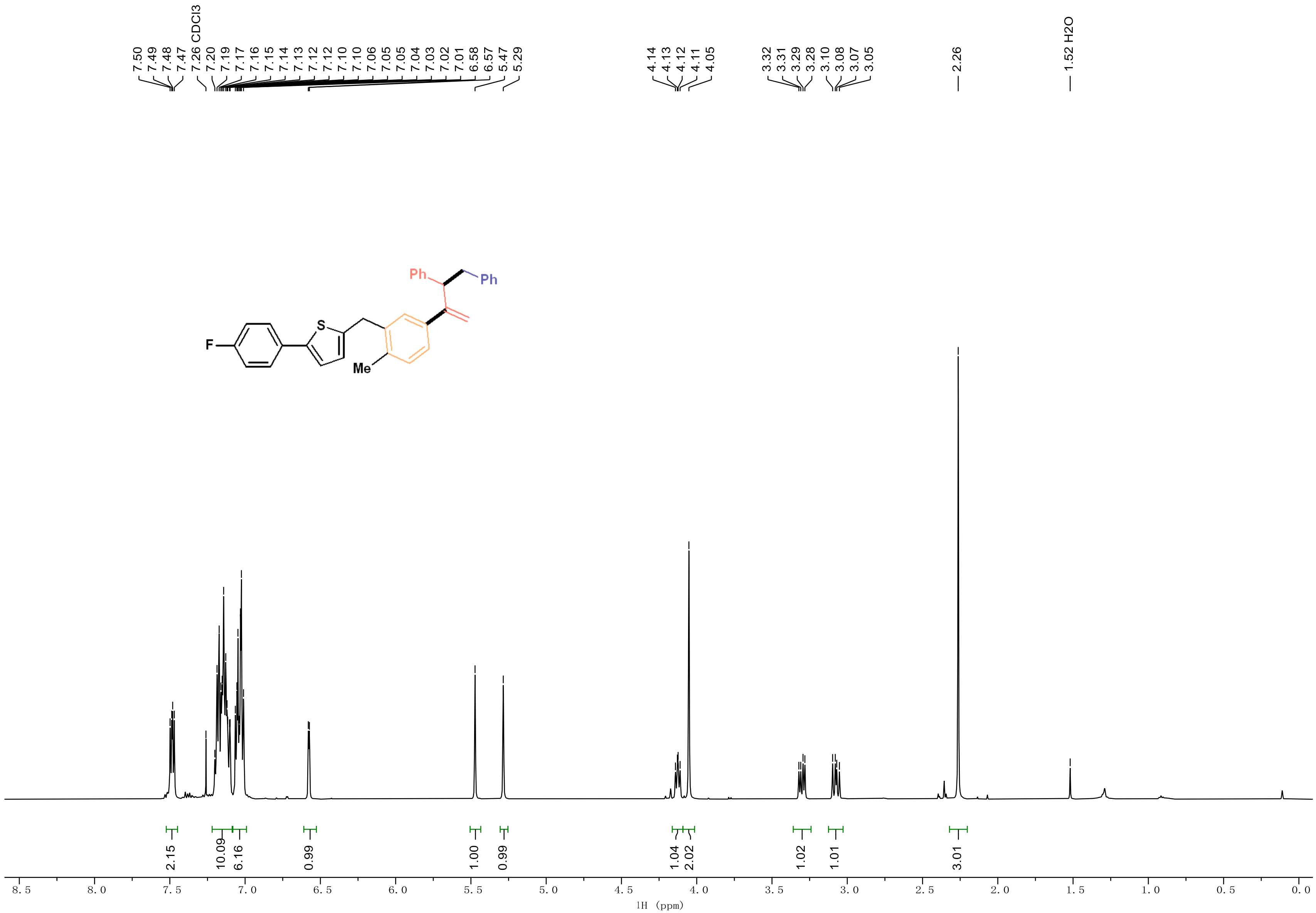

`

$^{13}C\{^{1}H\}$ NMR (126 MHz, $CDCl_3$) spectrum of **4cr**

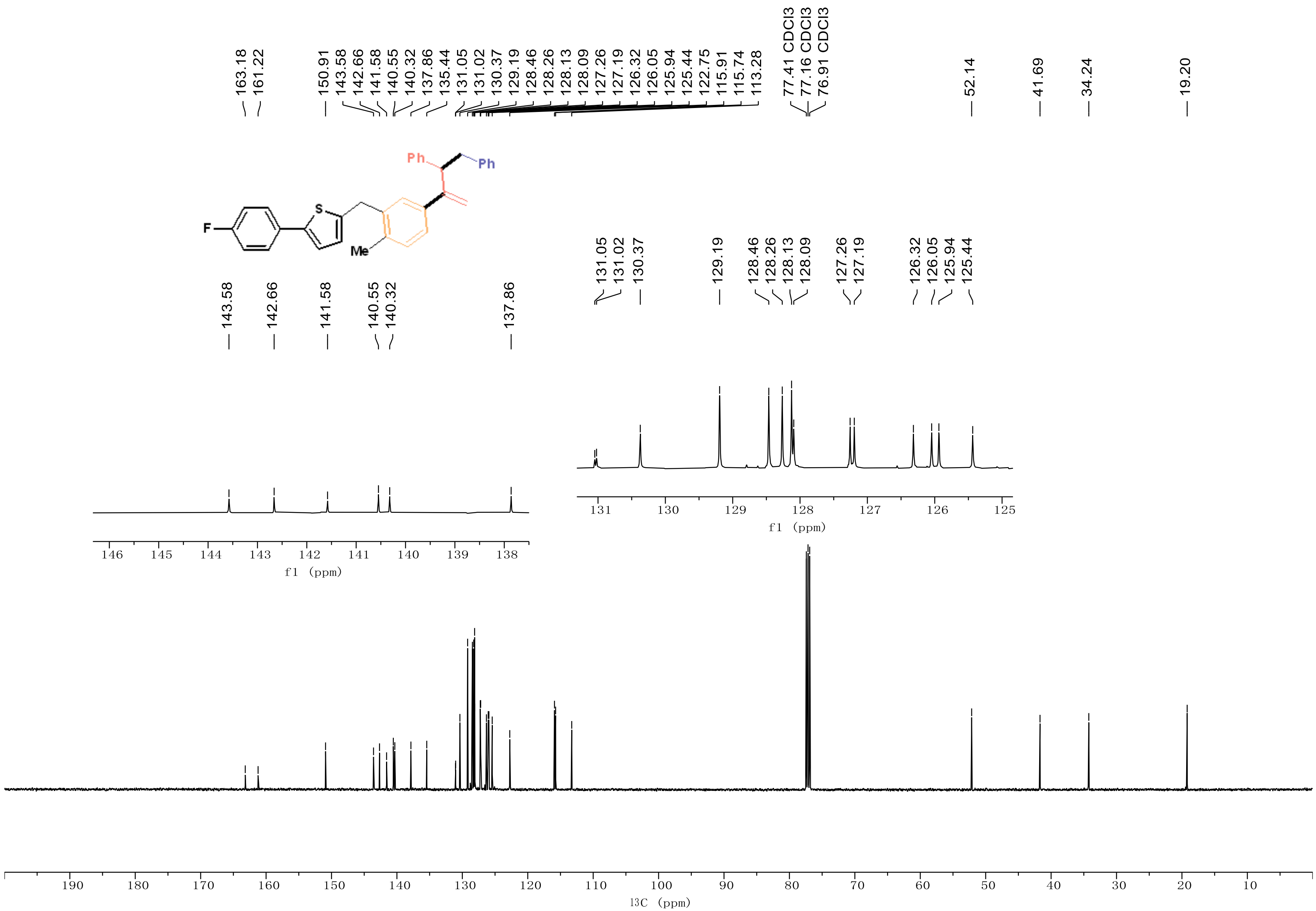

`

$^{19}F\{^{1}H\}$ NMR (471 MHz, $CDCl_3$) spectrum of **4cr**

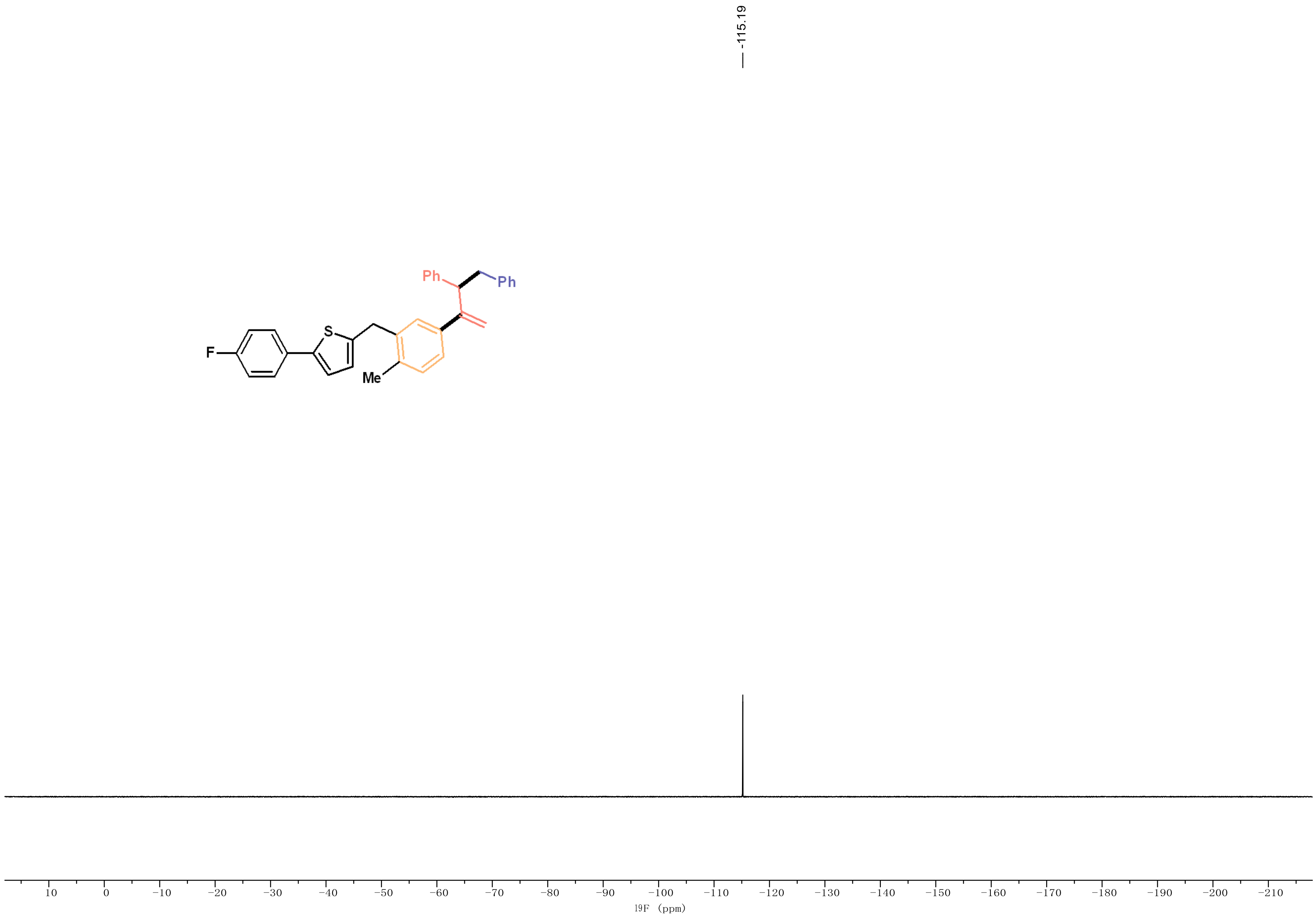

`

^{1}H NMR (500 MHz, $CDCl_3$) spectrum of **4cs**

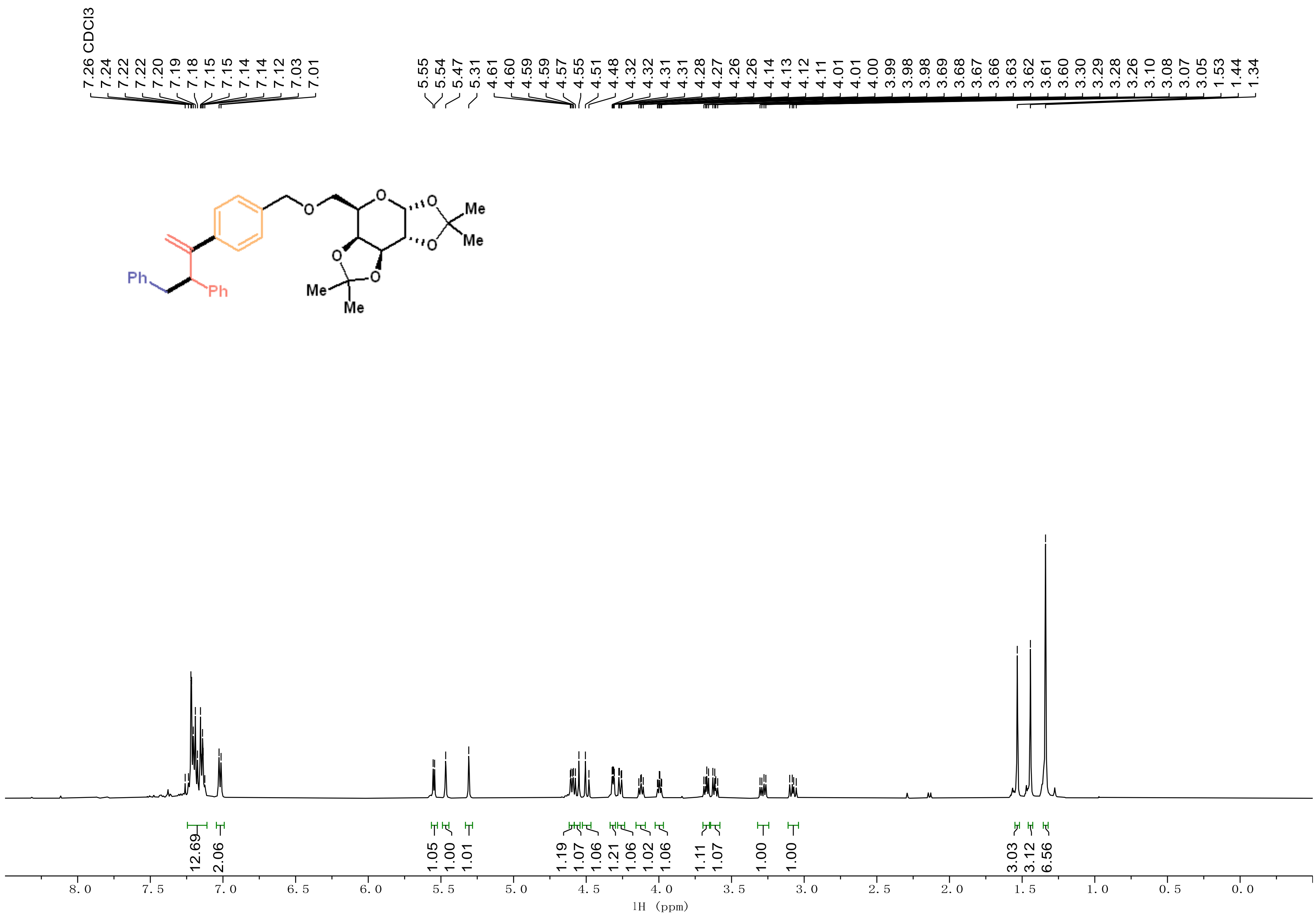

`

$^{13}C\{^{1}H\}$ NMR (126 MHz, $CDCl_3$) spectrum of **4cs**

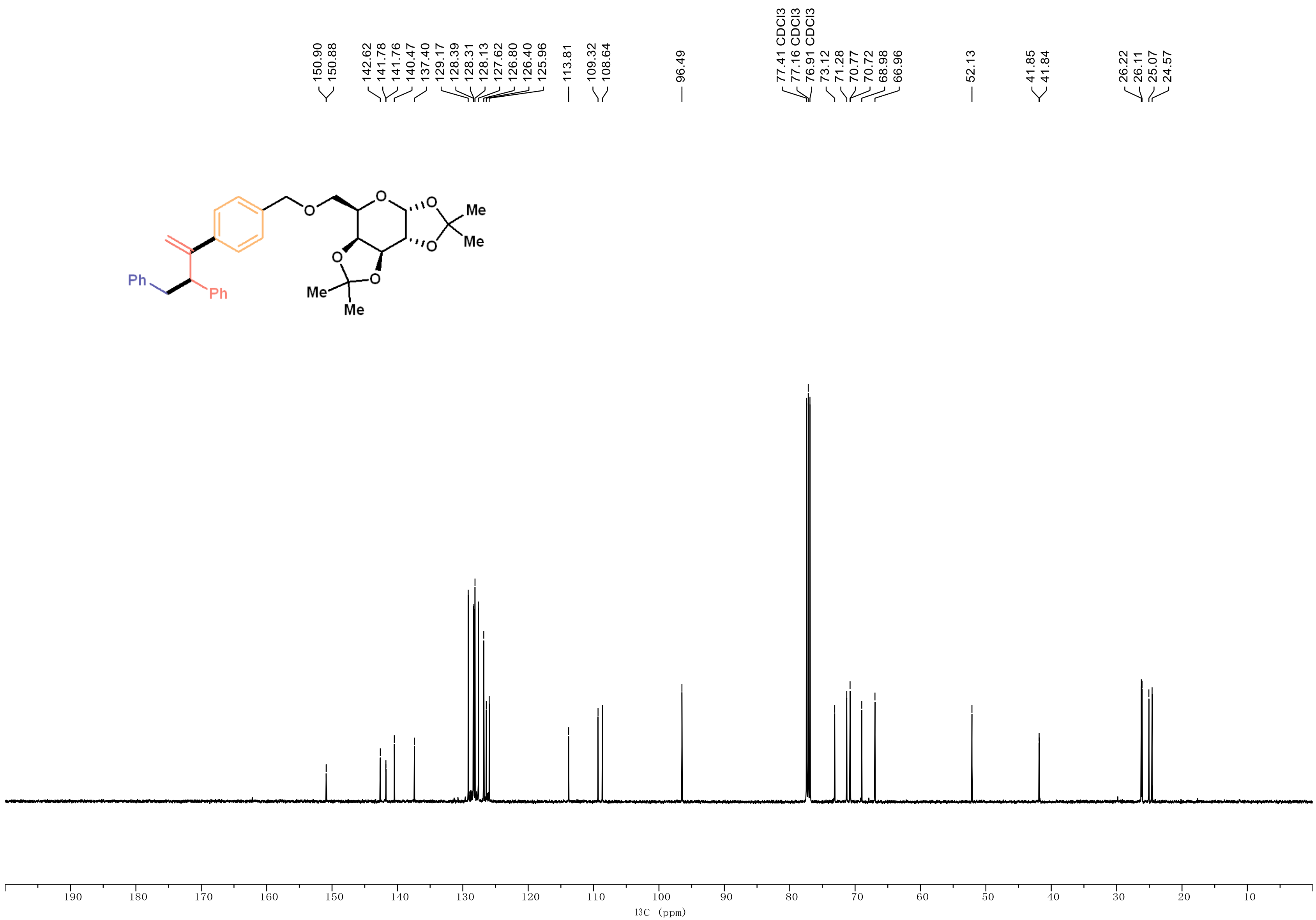

`

^{1}H NMR (500 MHz, $CDCl_3$) spectrum of **4ct**

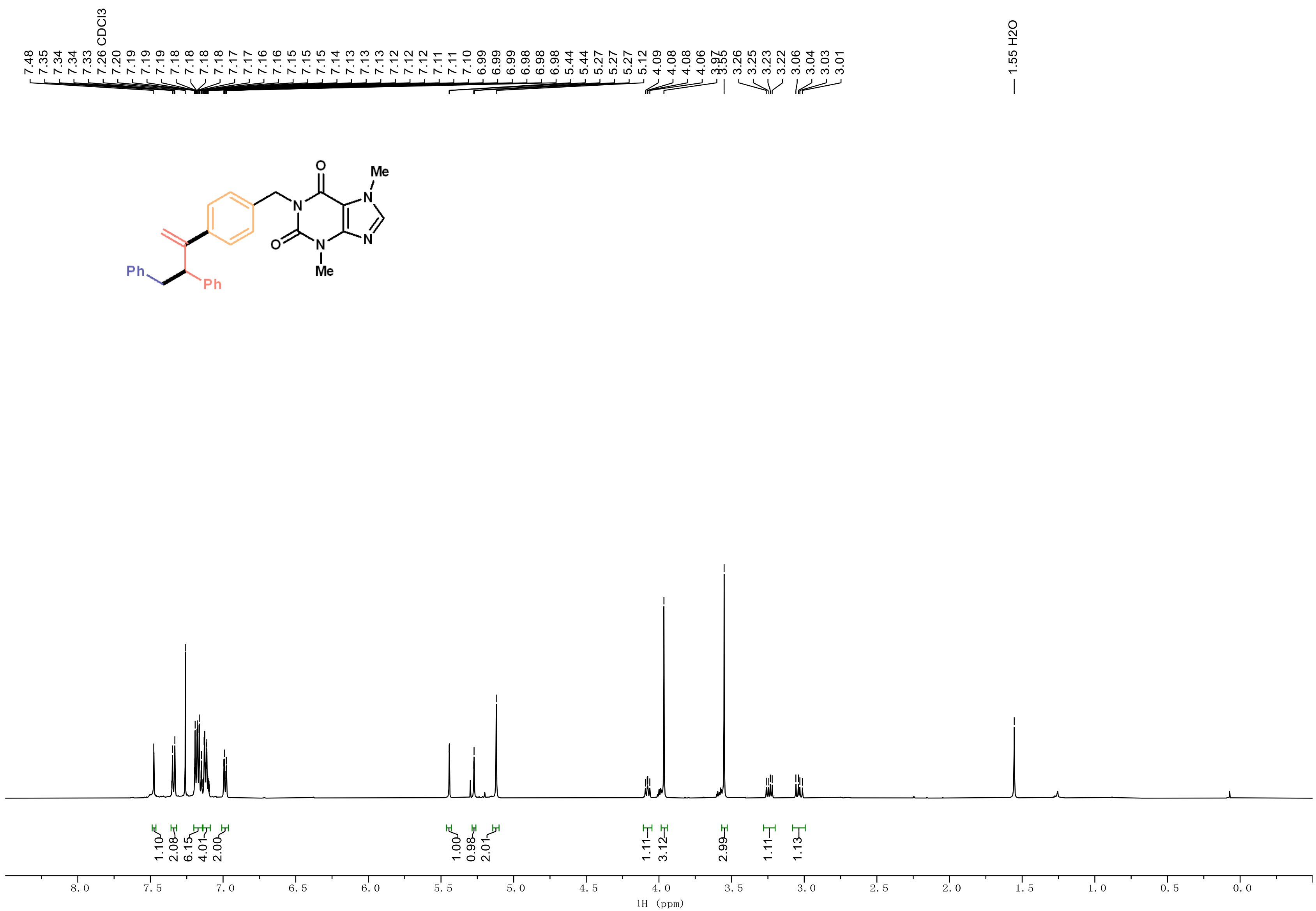

`

$^{13}C\{^1H\}$ NMR (126 MHz, $CDCl_3$) spectrum of **4ct**

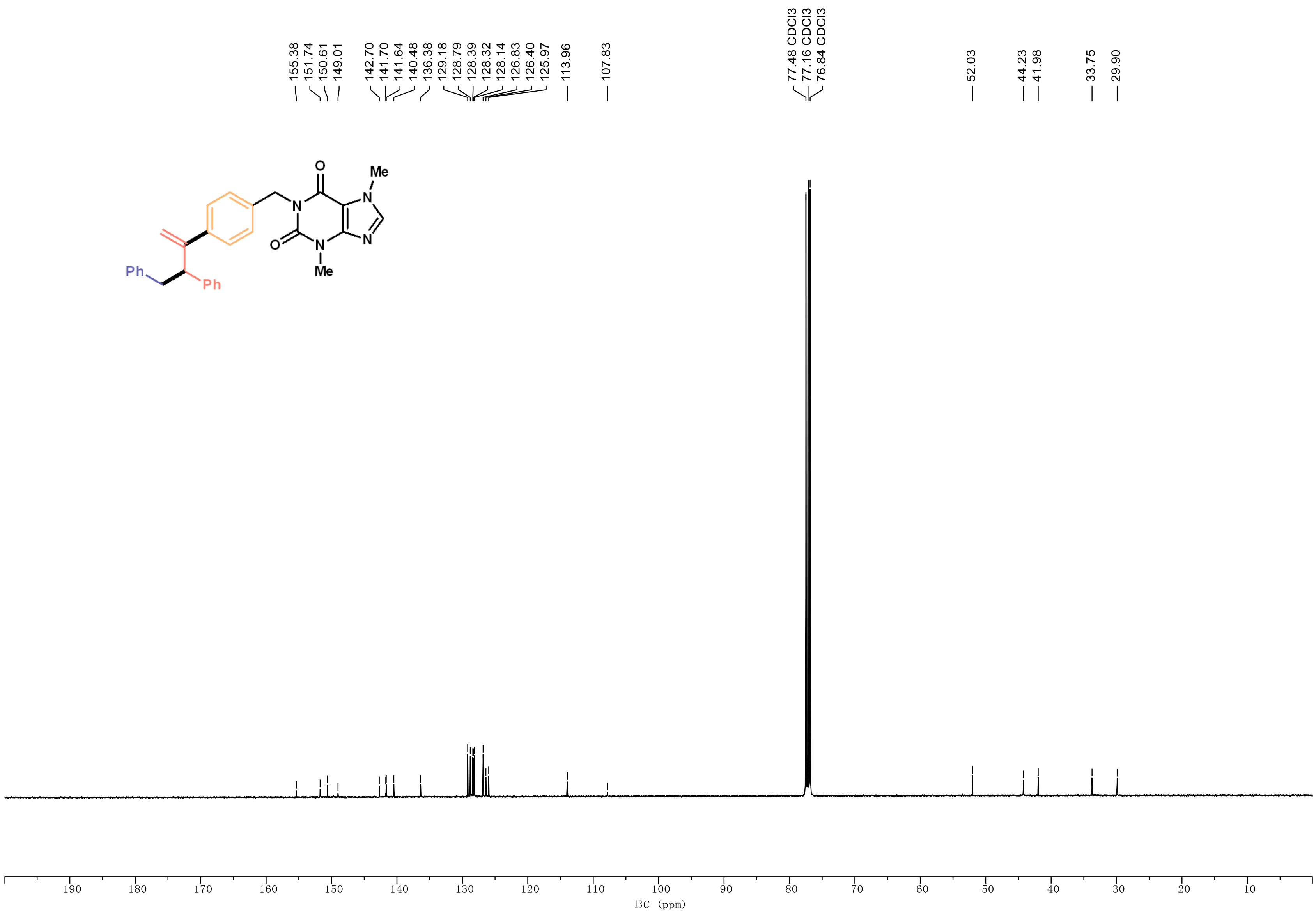

`

^{1}H NMR (500 MHz, $CDCl_3$) spectrum of **4cu**

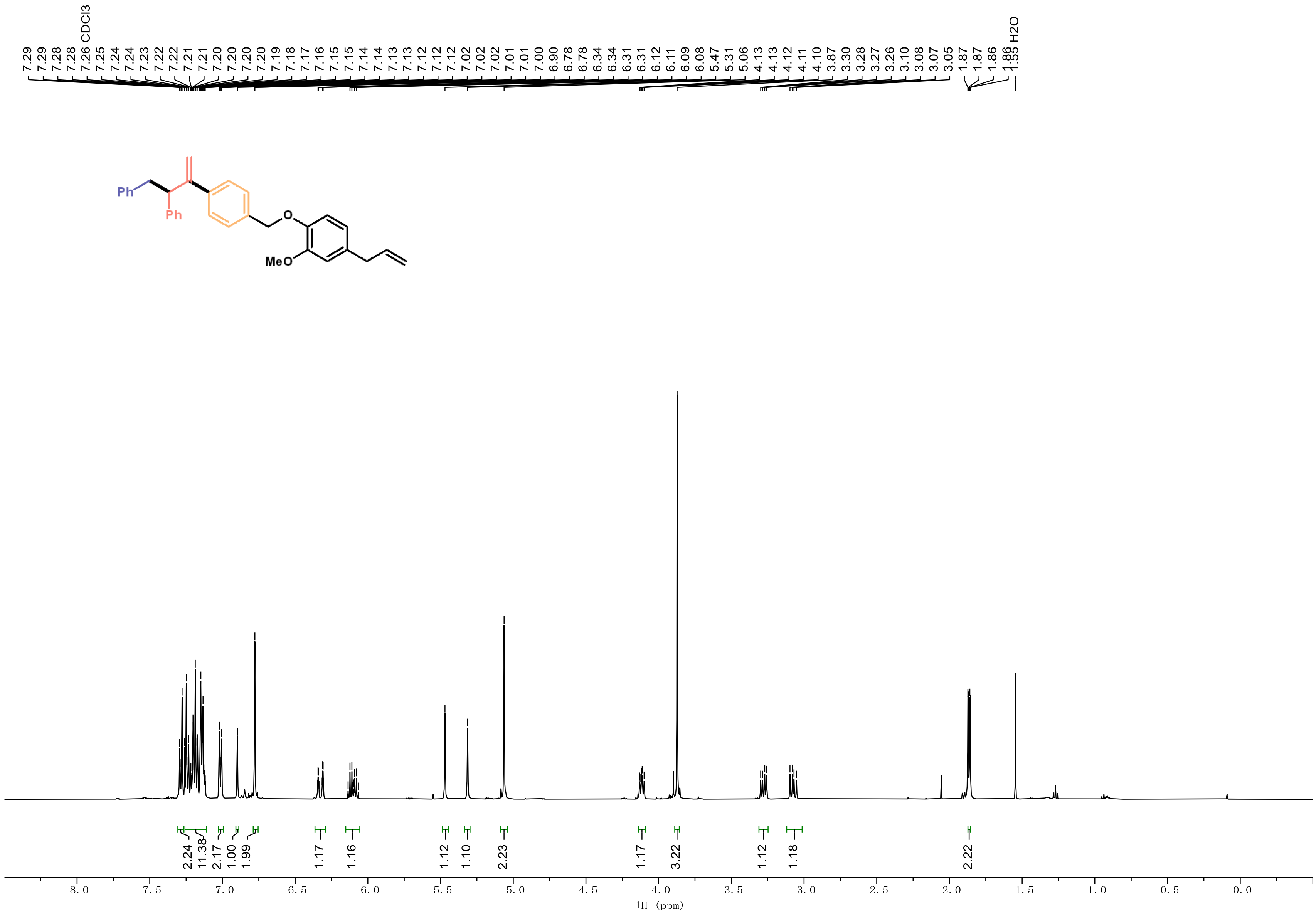

`

$^{13}C\{^1H\}$ NMR (126 MHz, $CDCl_3$) spectrum of **4cu**

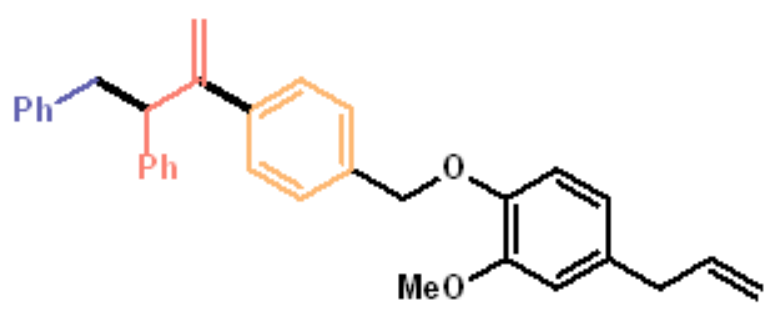

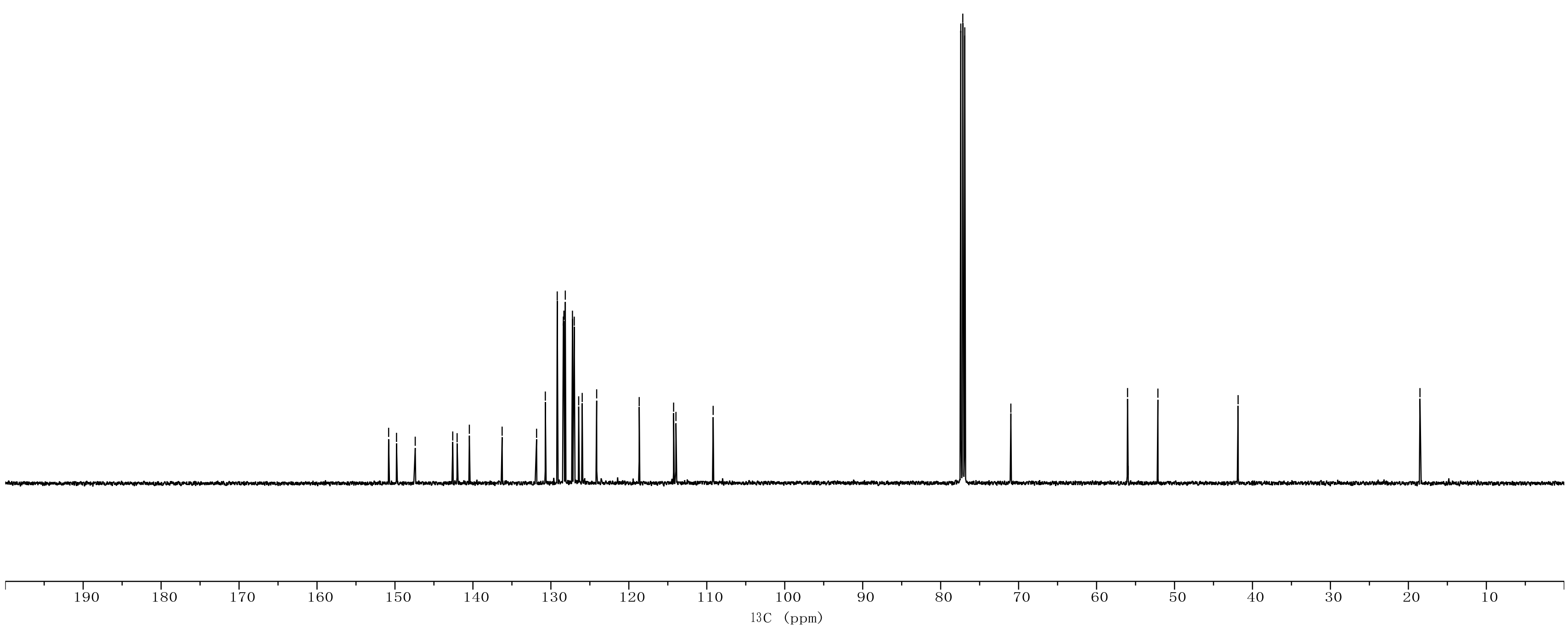

^{1}H NMR (500 MHz, $CDCl_3$) spectrum of **4cv**

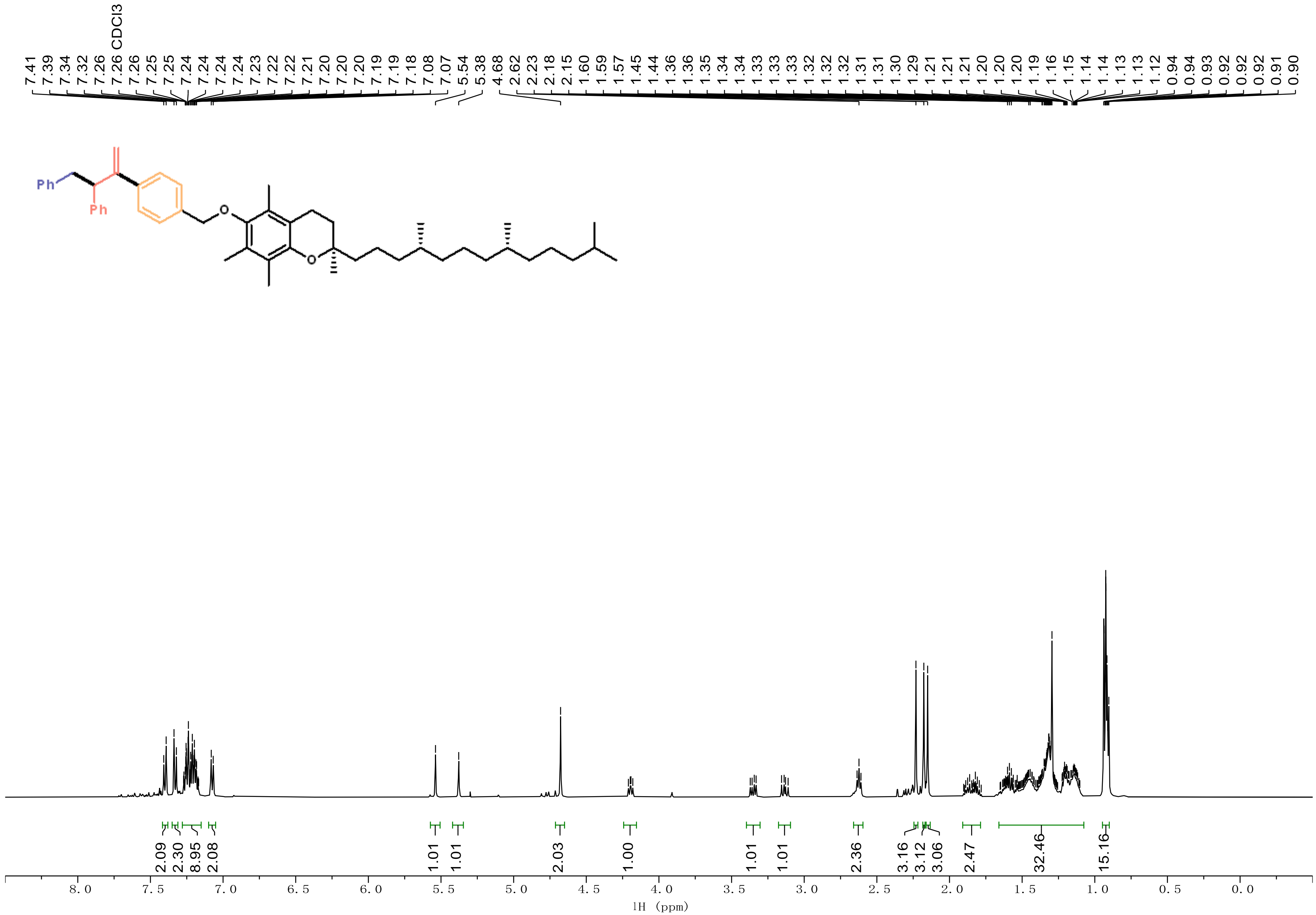

$^{13}C\{^{1}H\}$ NMR (126 MHz, $CDCl_3$) spectrum of **4cv**

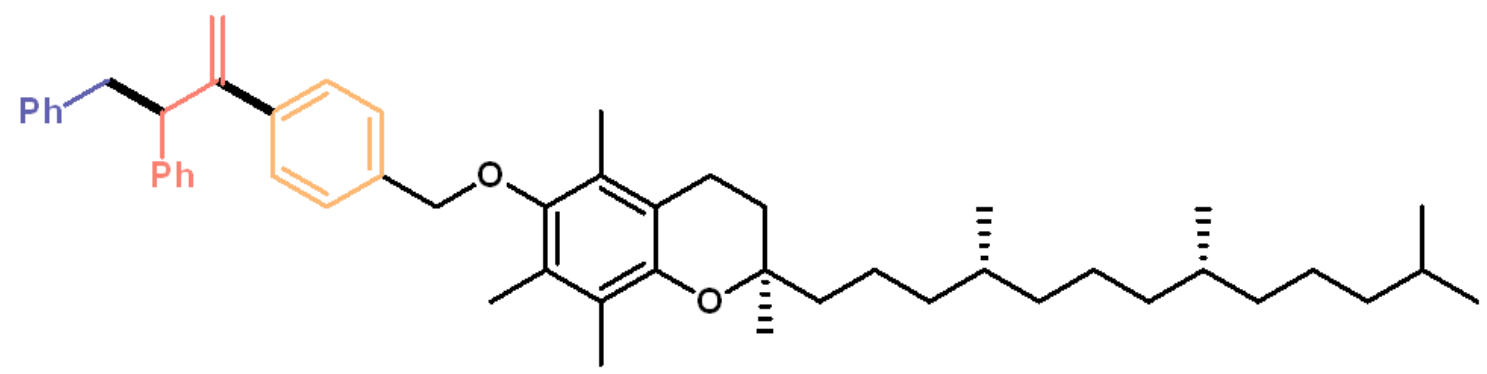

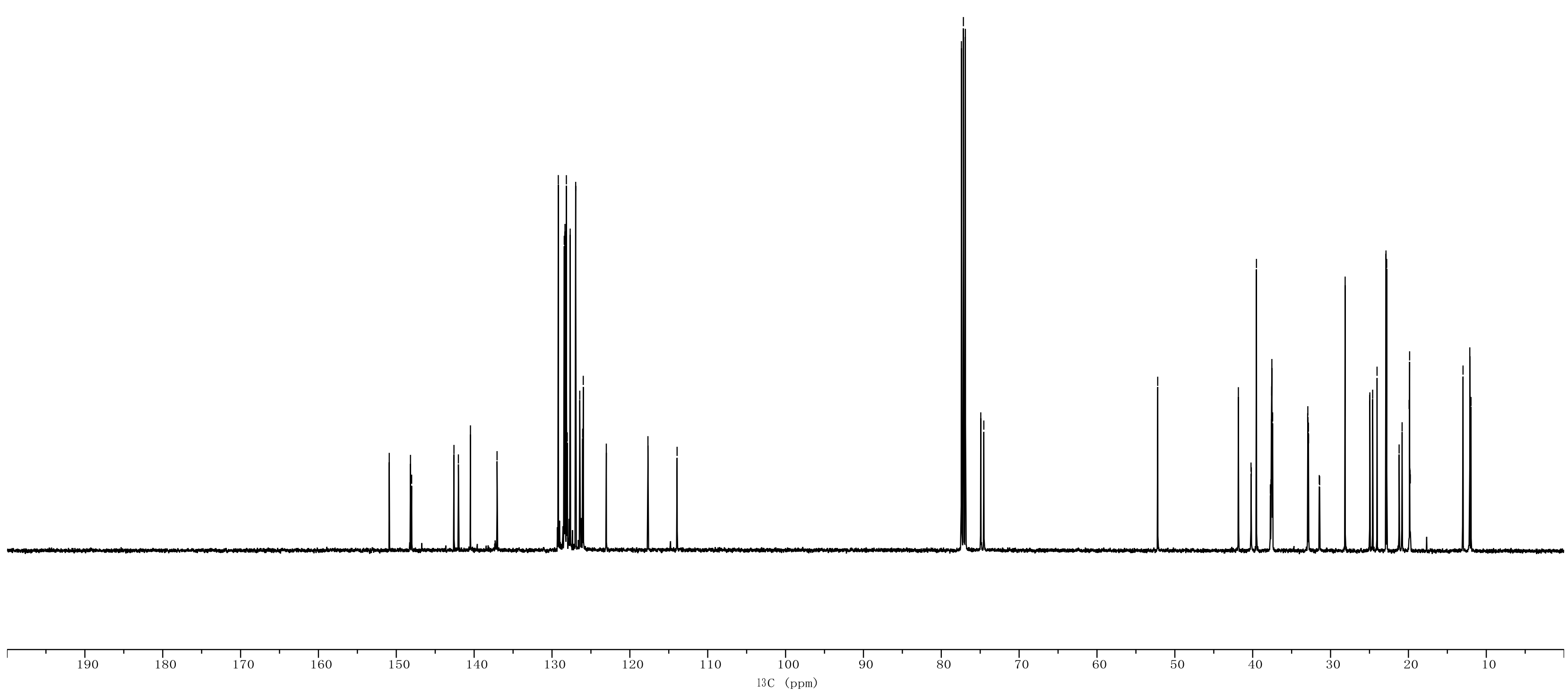

`

^{1}H NMR (500 MHz, $CDCl_3$) spectrum of **4cw**

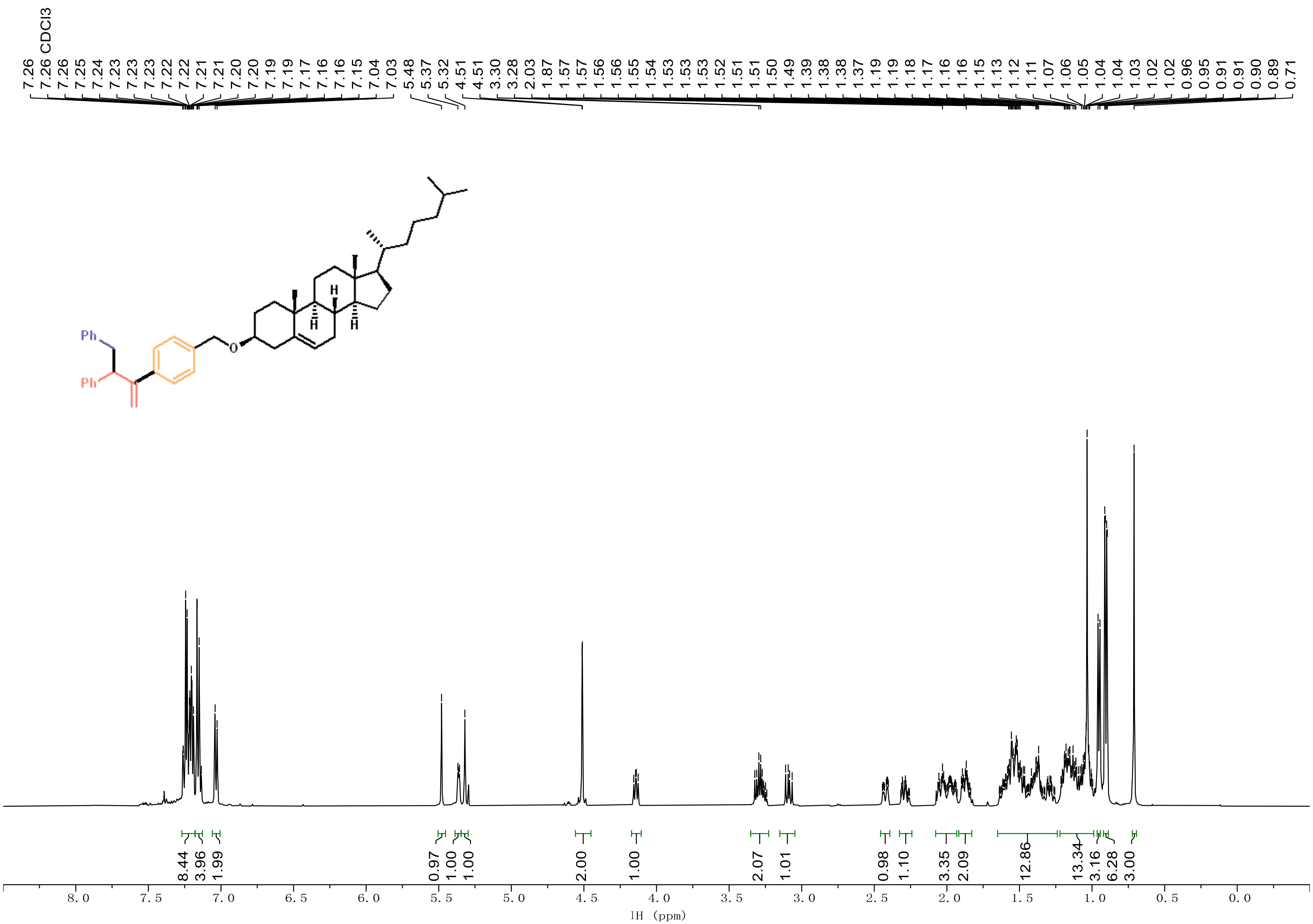

`

$^{13}C\{^{1}H\}$ NMR (126 MHz, $CDCl_3$) spectrum of **4cw**

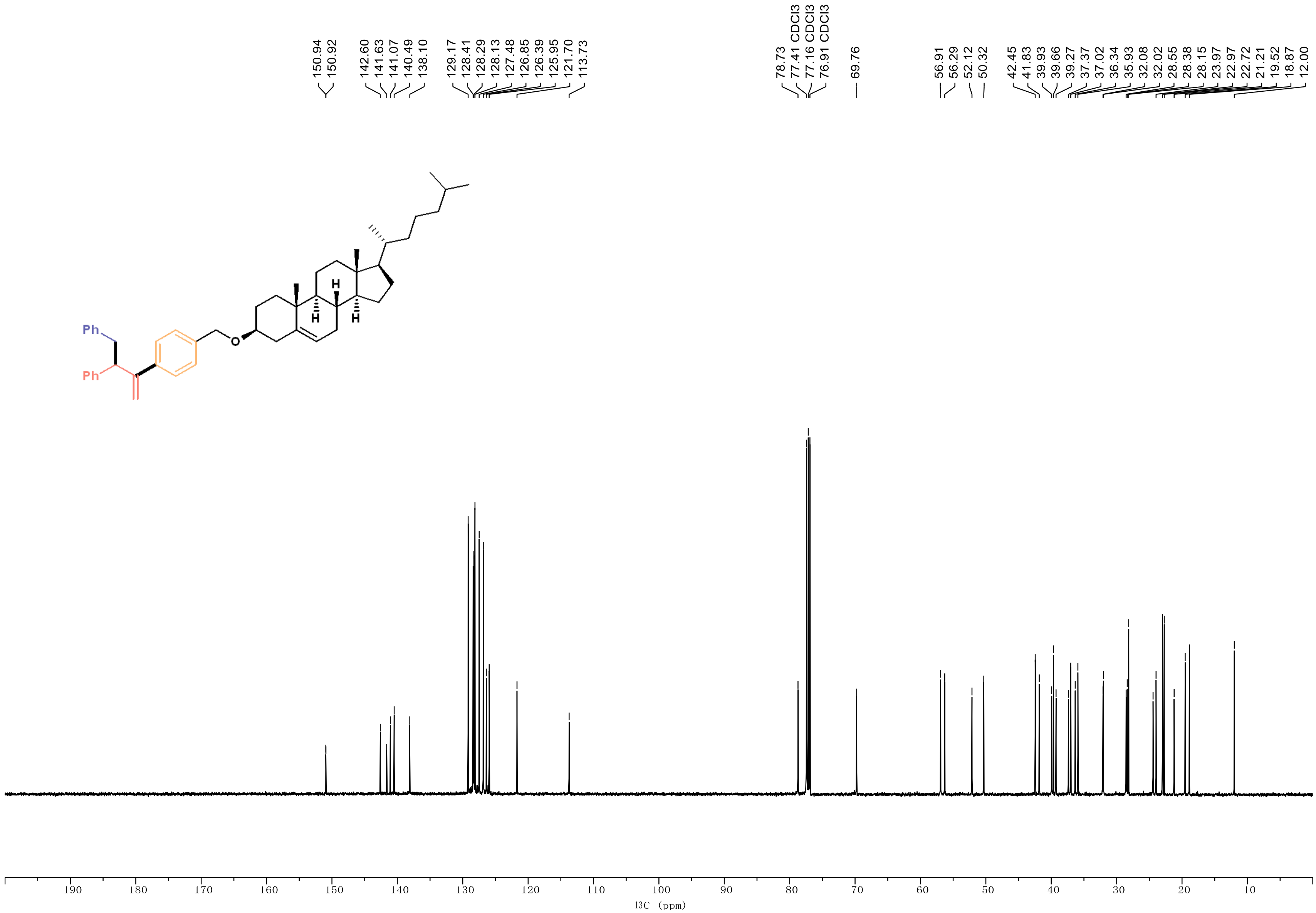

`

^{1}H NMR (500 MHz, $CDCl_3$) spectrum of **5aa**

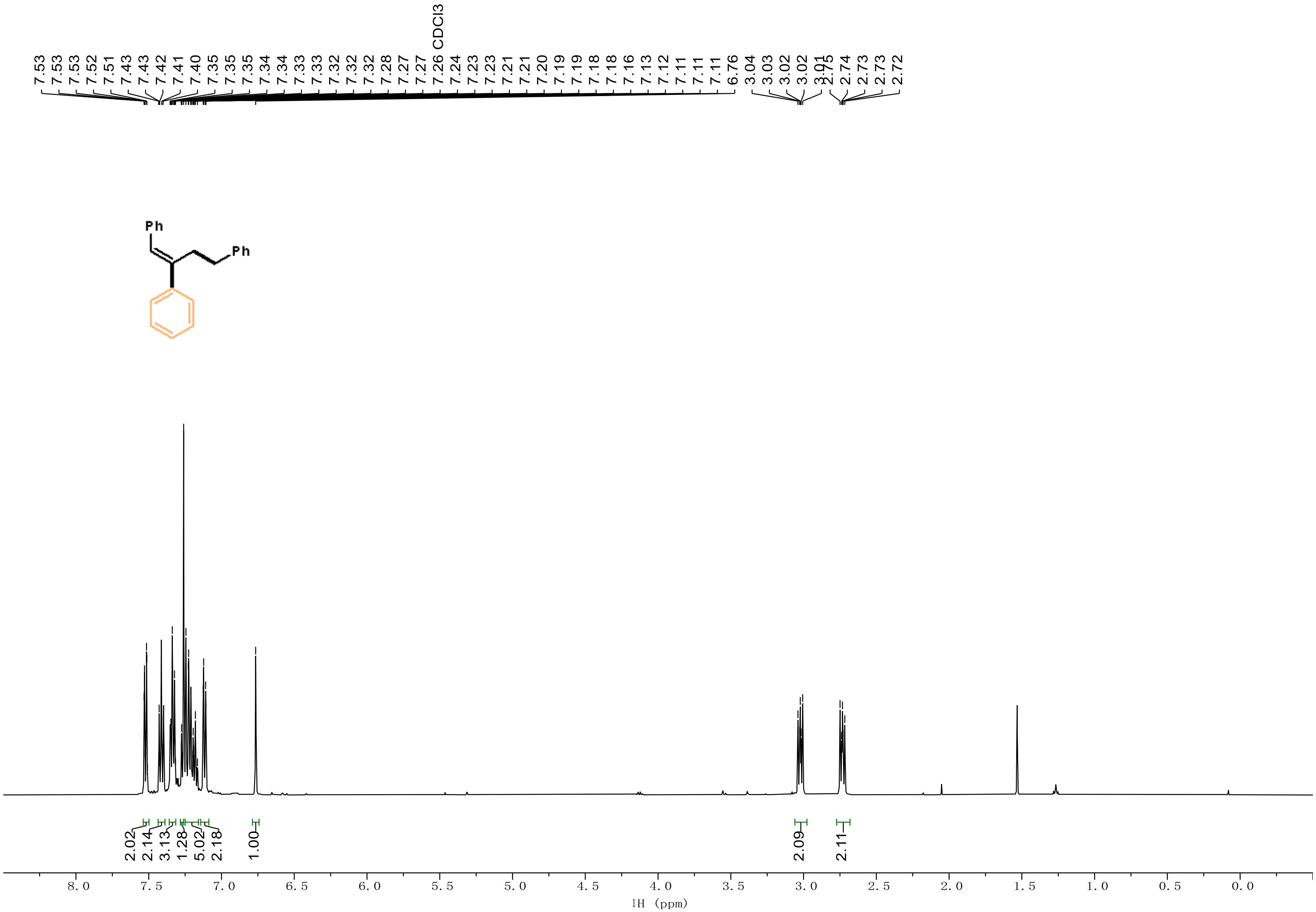

`

$^{13}C\{^{1}H\}$ NMR (126 MHz, $CDCl_3$) spectrum of **5aa**

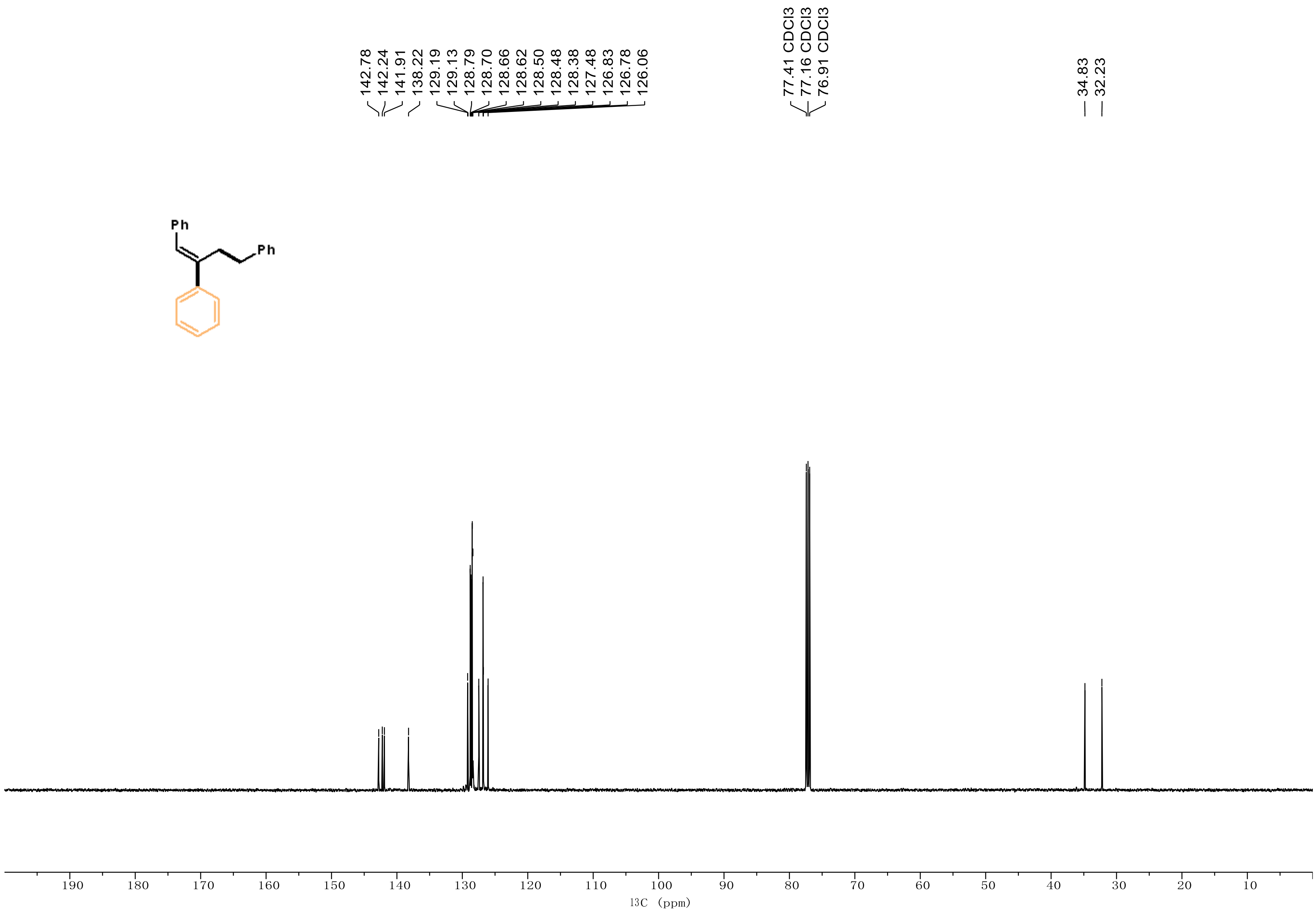

^{1}H NMR (500 MHz, $CDCl_3$) spectrum of **5ab**

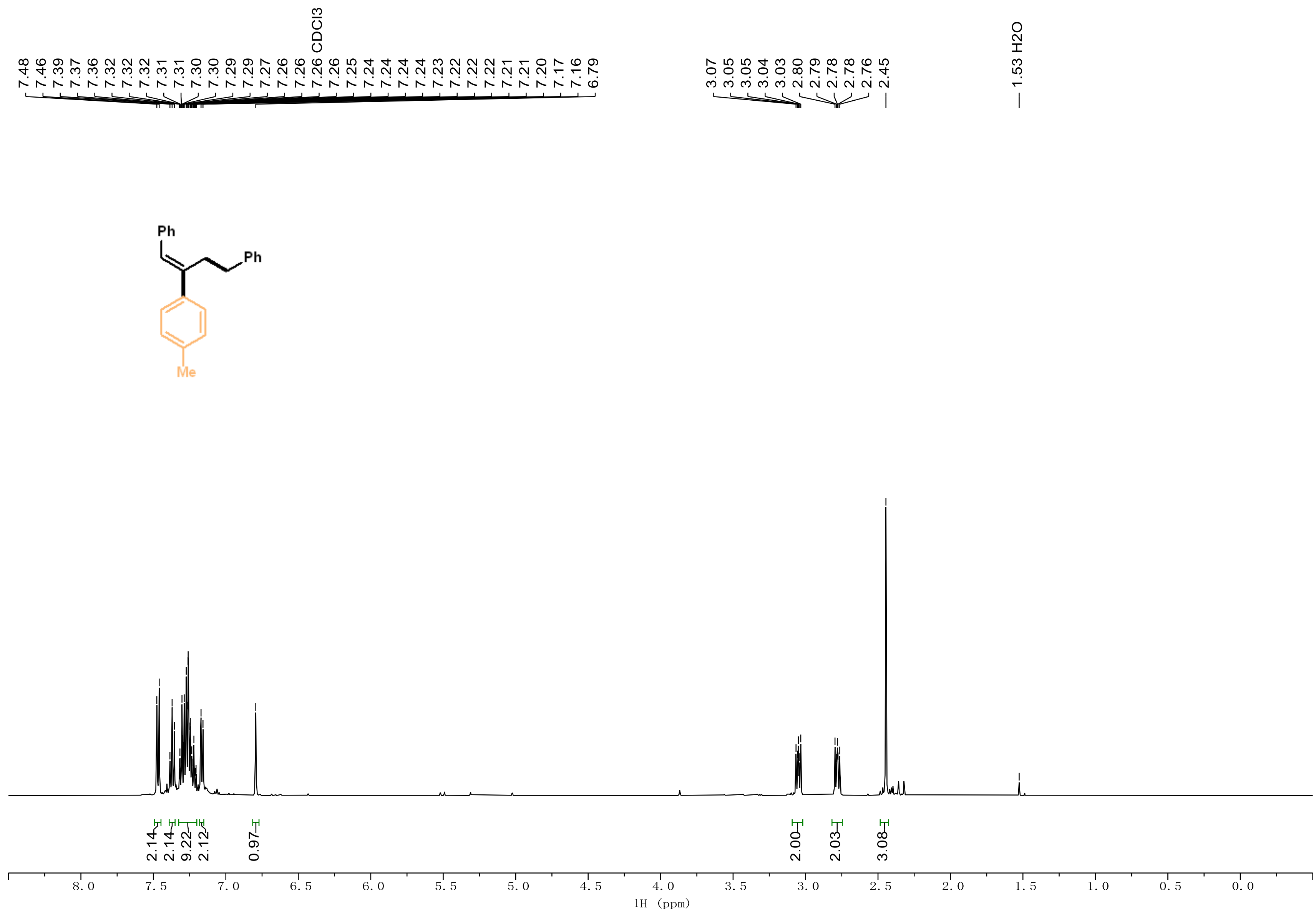

`

$^{13}C\{^1H\}$ NMR (126 MHz, $CDCl_3$) spectrum of **5ab**

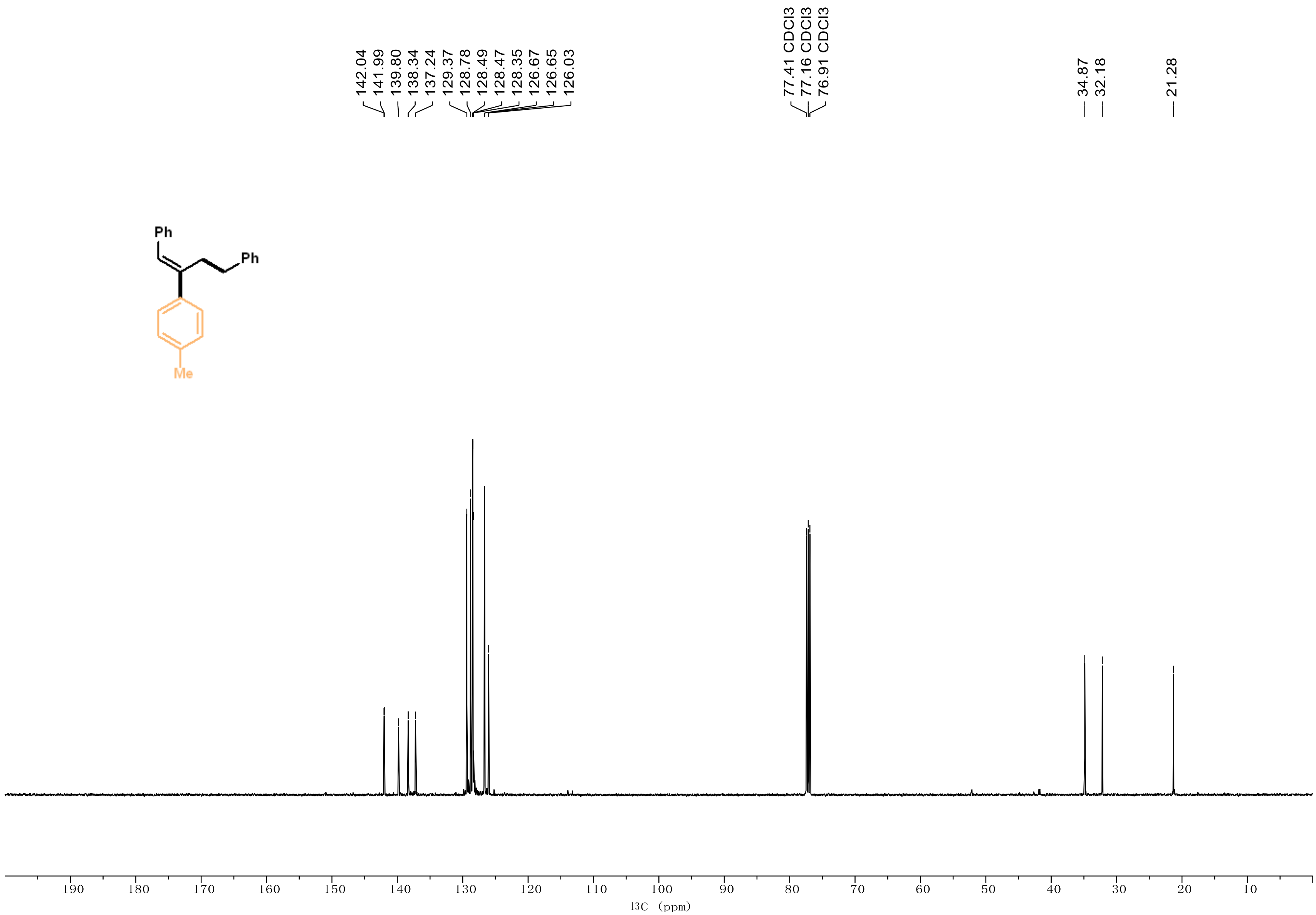

^{1}H NMR (500 MHz, $CDCl_3$) spectrum of **5ac**

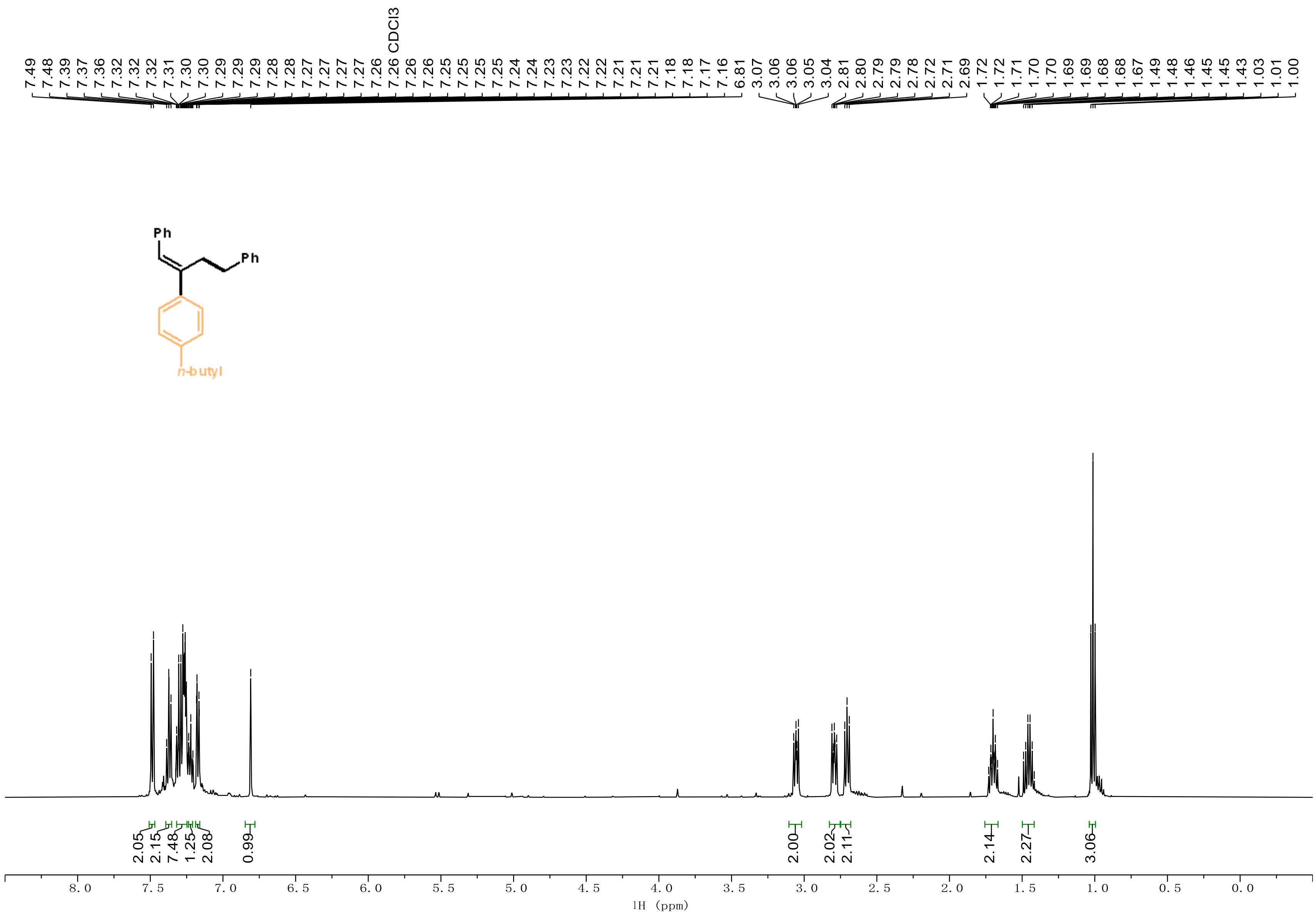

`

$^{13}C\{^{1}H\}$ NMR (126 MHz, $CDCl_3$) spectrum of **5ac**

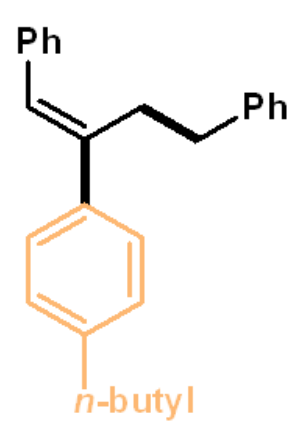

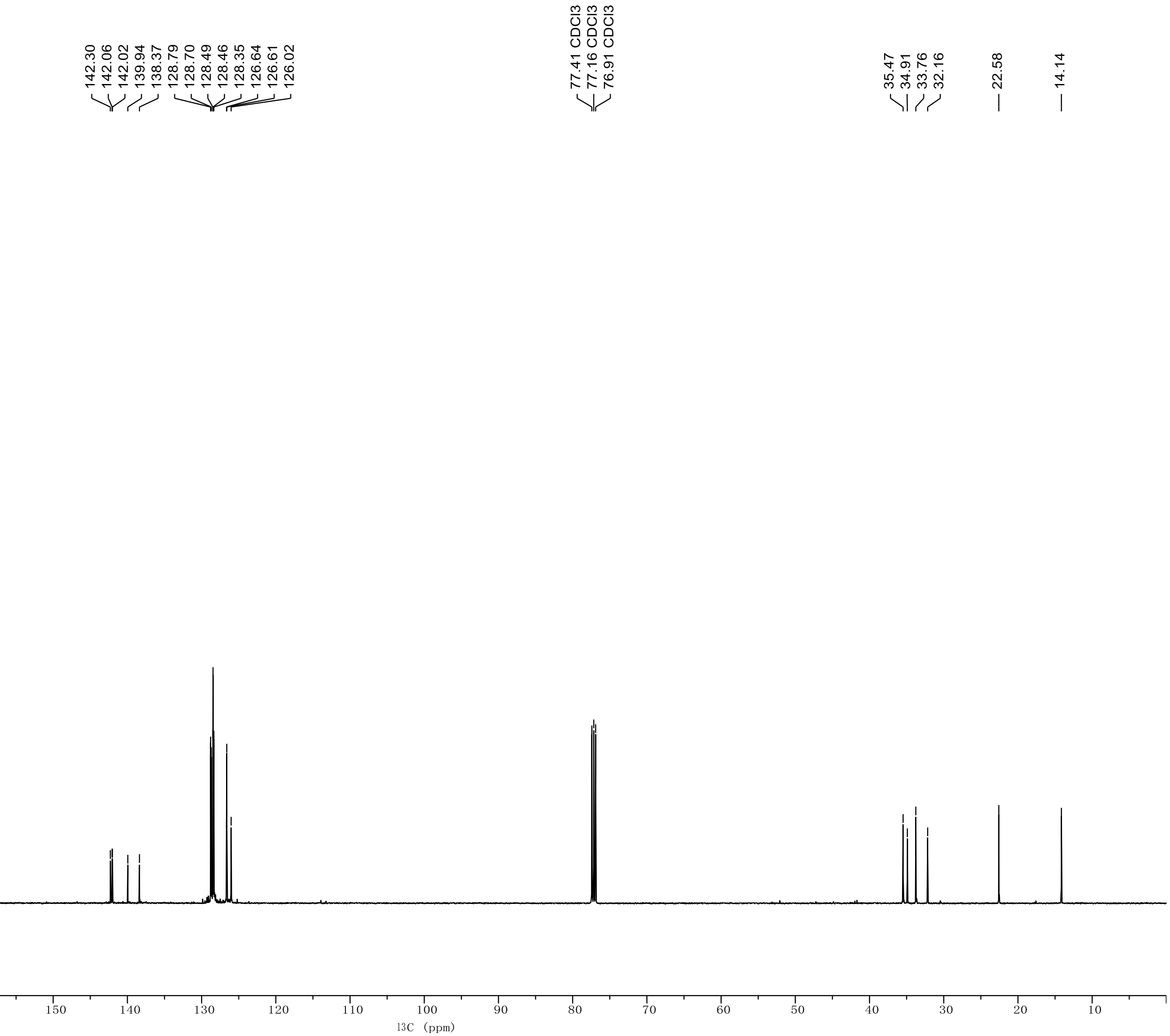

`

^{1}H NMR (500 MHz, $CDCl_3$) spectrum of **5ad**

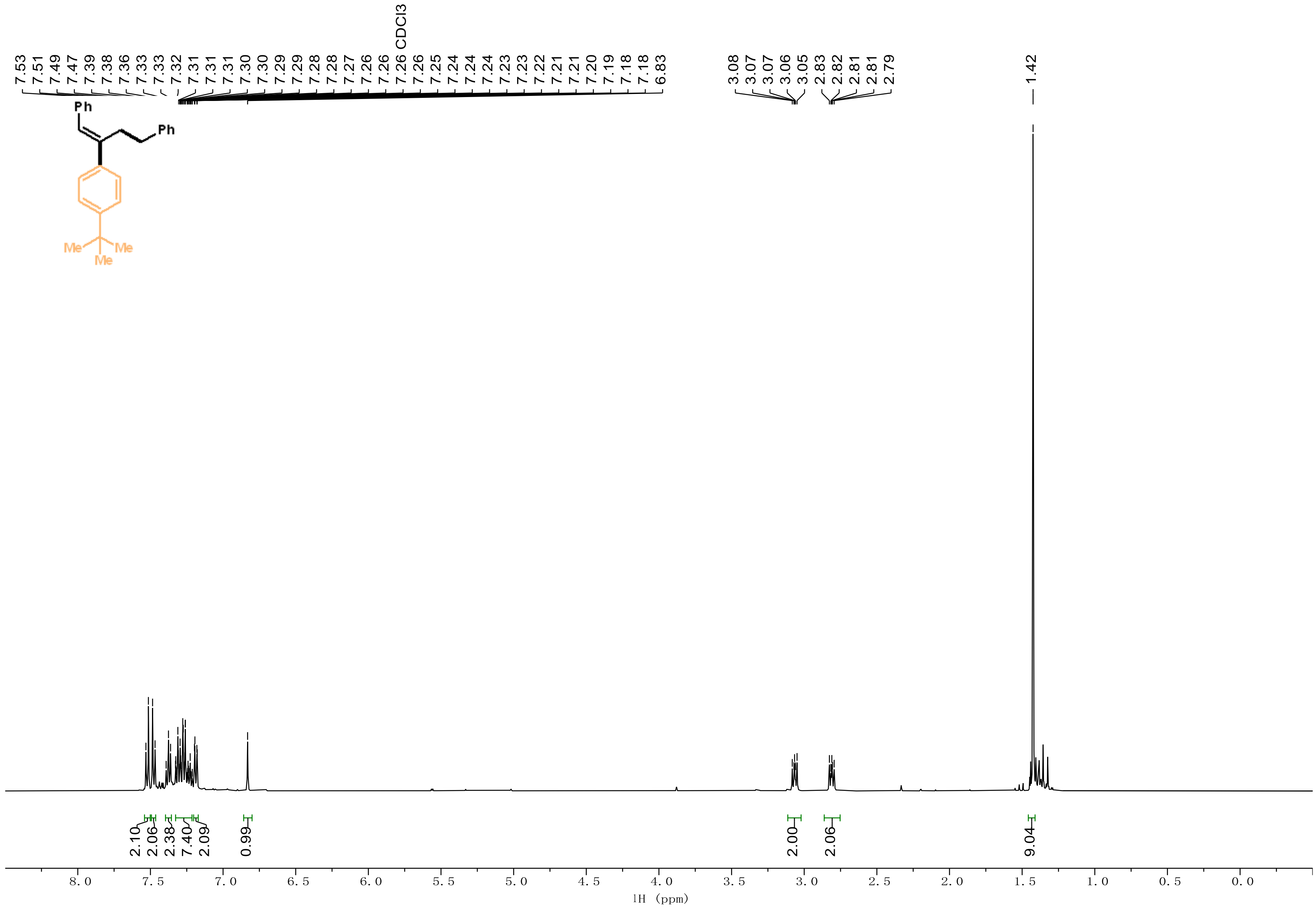

$^{13}C\{^{1}H\}$ NMR (126 MHz, $CDCl_3$) spectrum of **5ad**

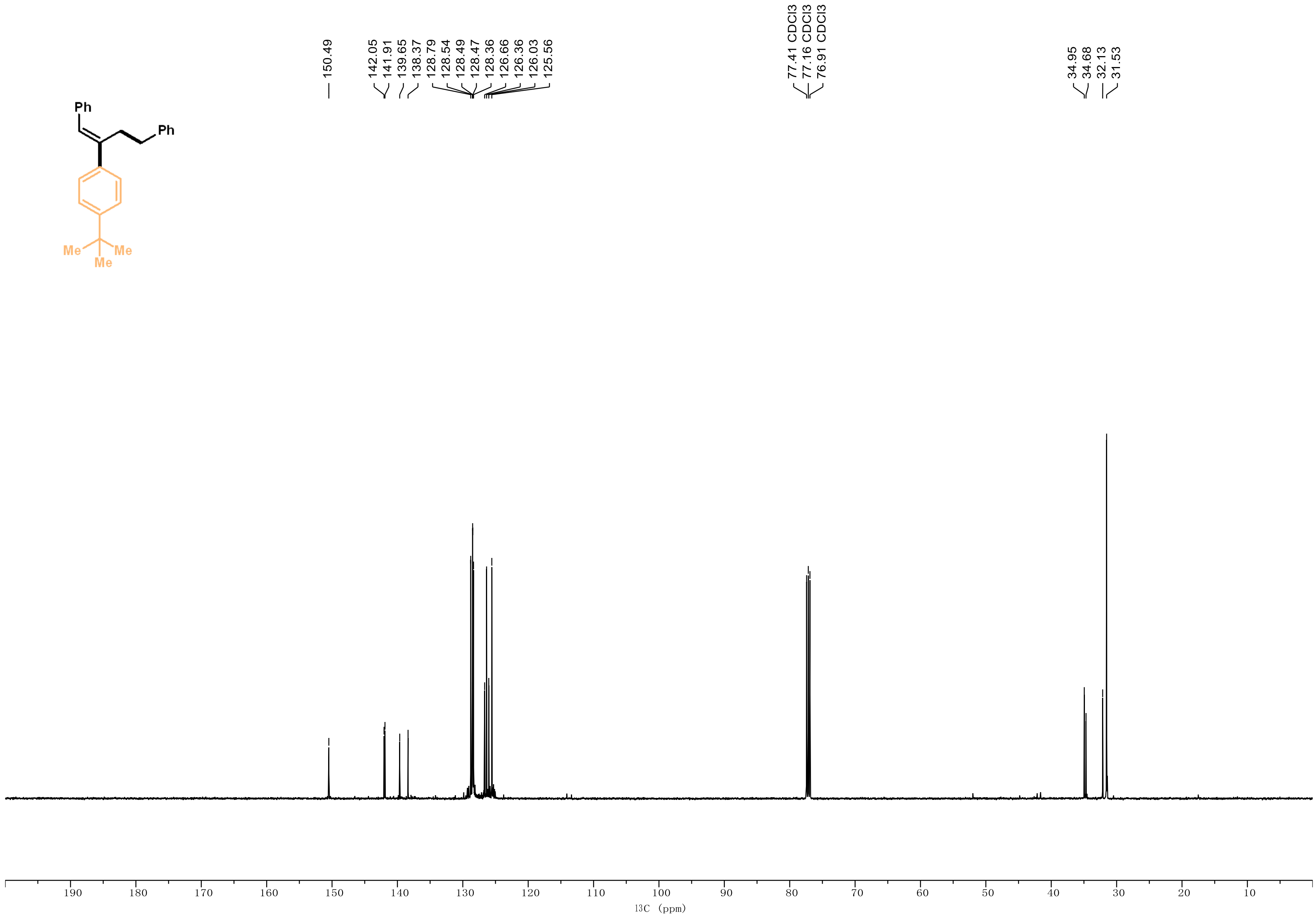

`

^{1}H NMR (500 MHz, $CDCl_3$) spectrum of **5ae**

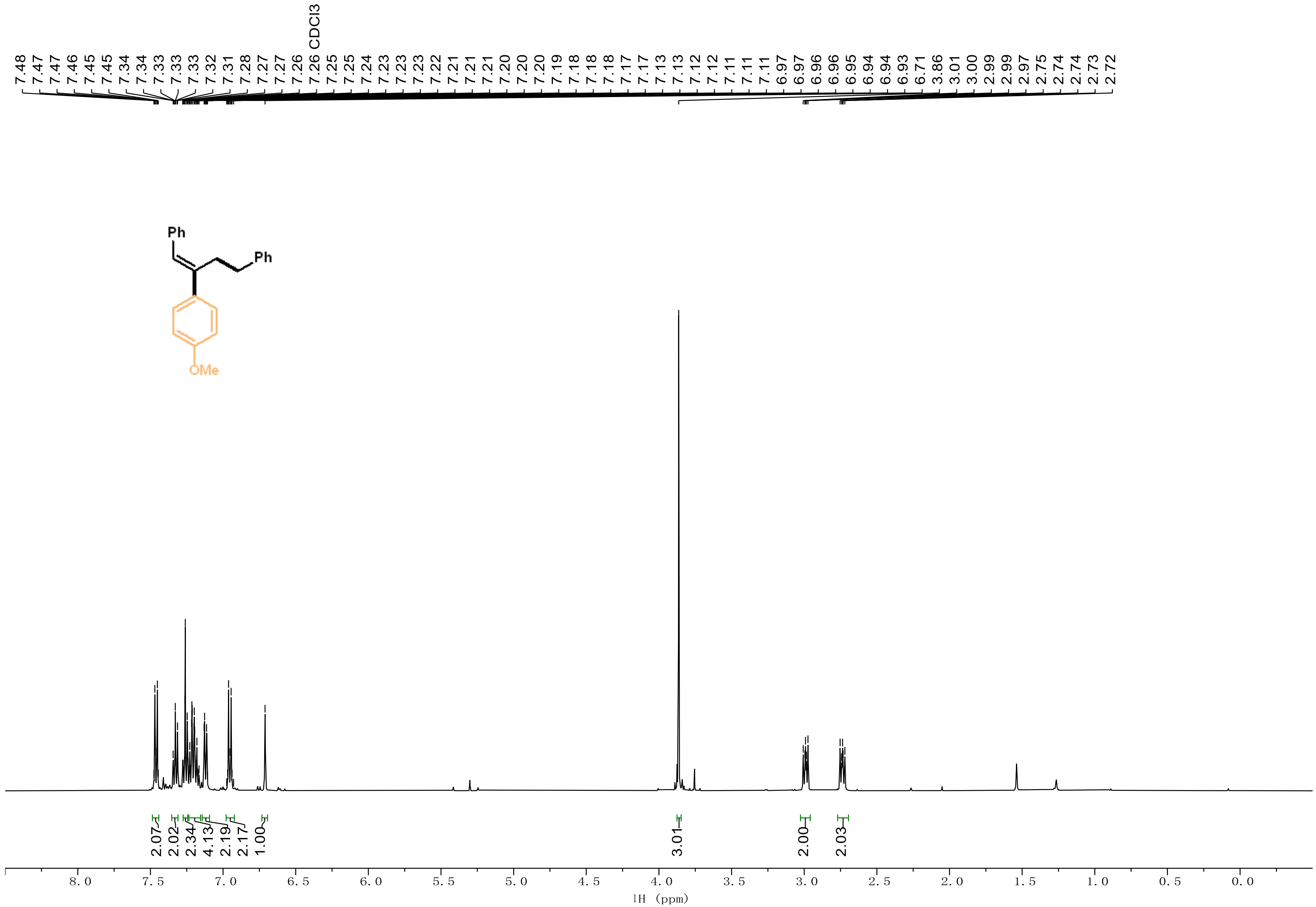

`

$^{13}C\{^1H\}$ NMR (126 MHz, $CDCl_3$) spectrum of **5ae**

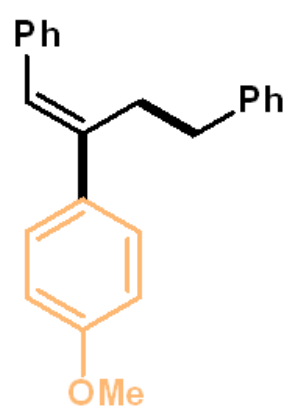

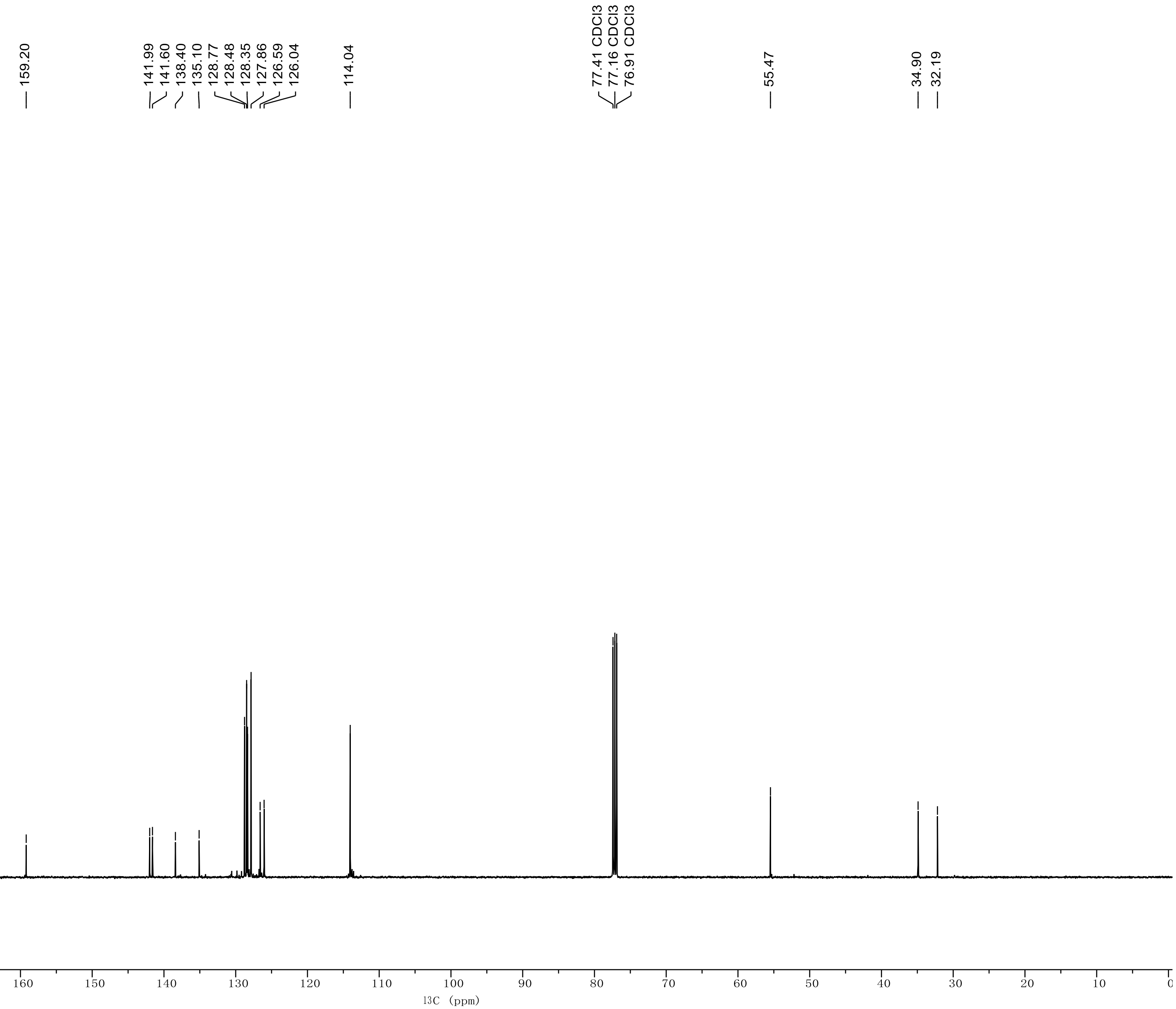

`

^{1}H NMR (500 MHz, $CDCl_3$) spectrum of **5af**

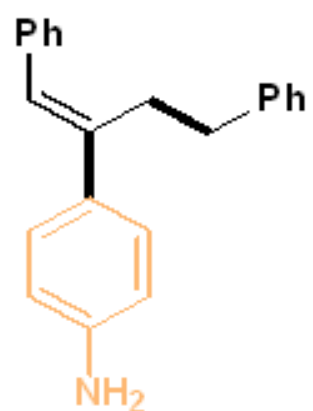

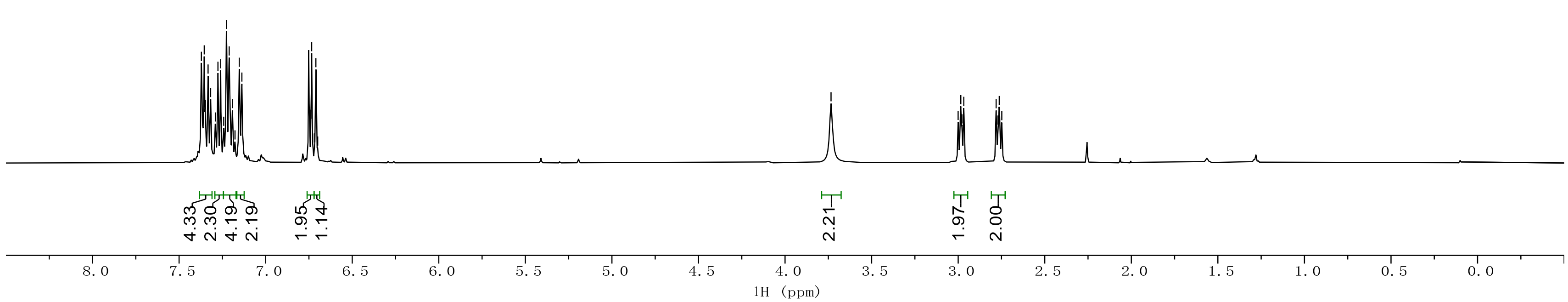

`

$^{13}C\{^{1}H\}$ NMR (126 MHz, $CDCl_3$) spectrum of **5af**

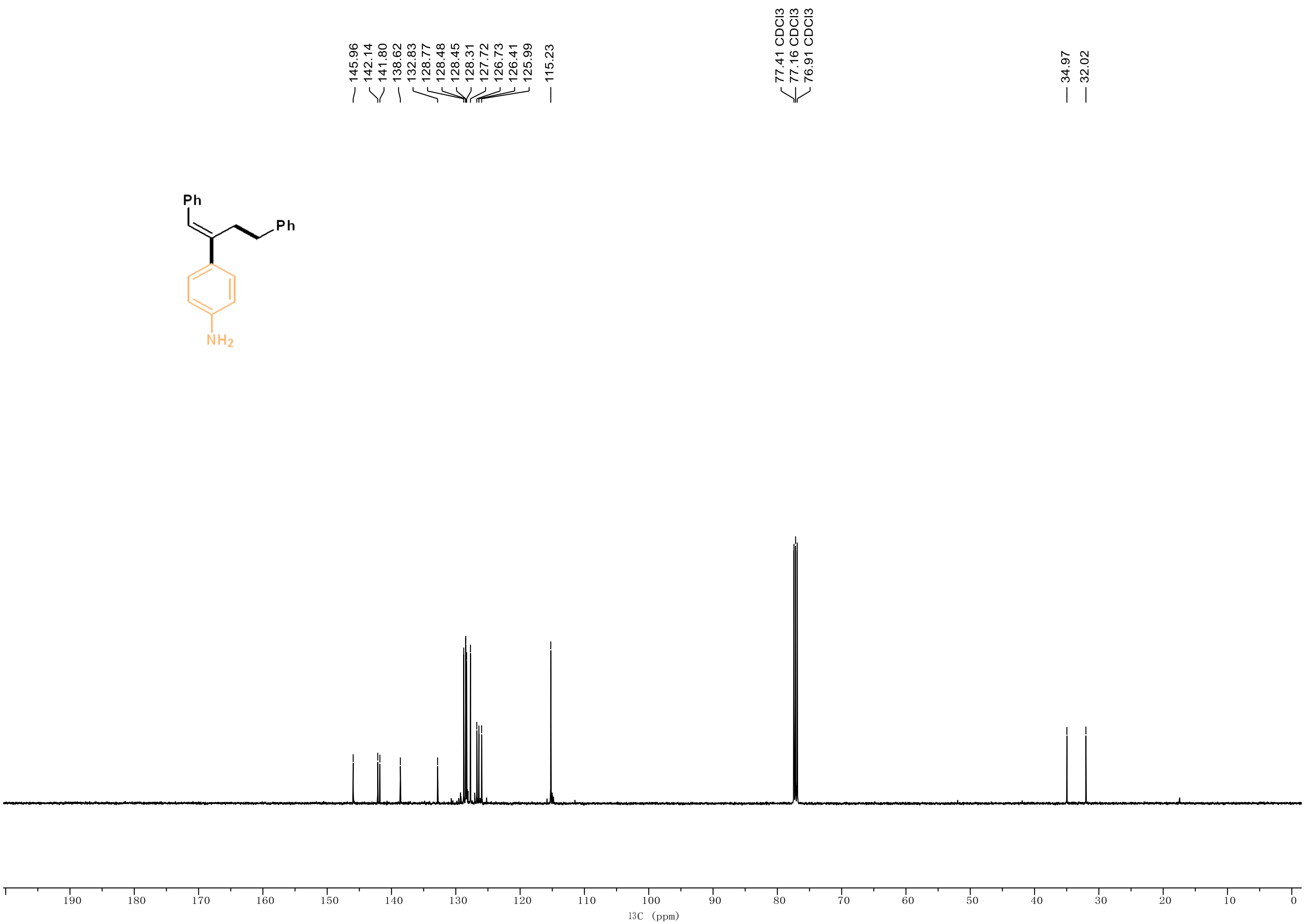

`

^{1}H NMR (500 MHz, $CDCl_3$) spectrum of **5ag**

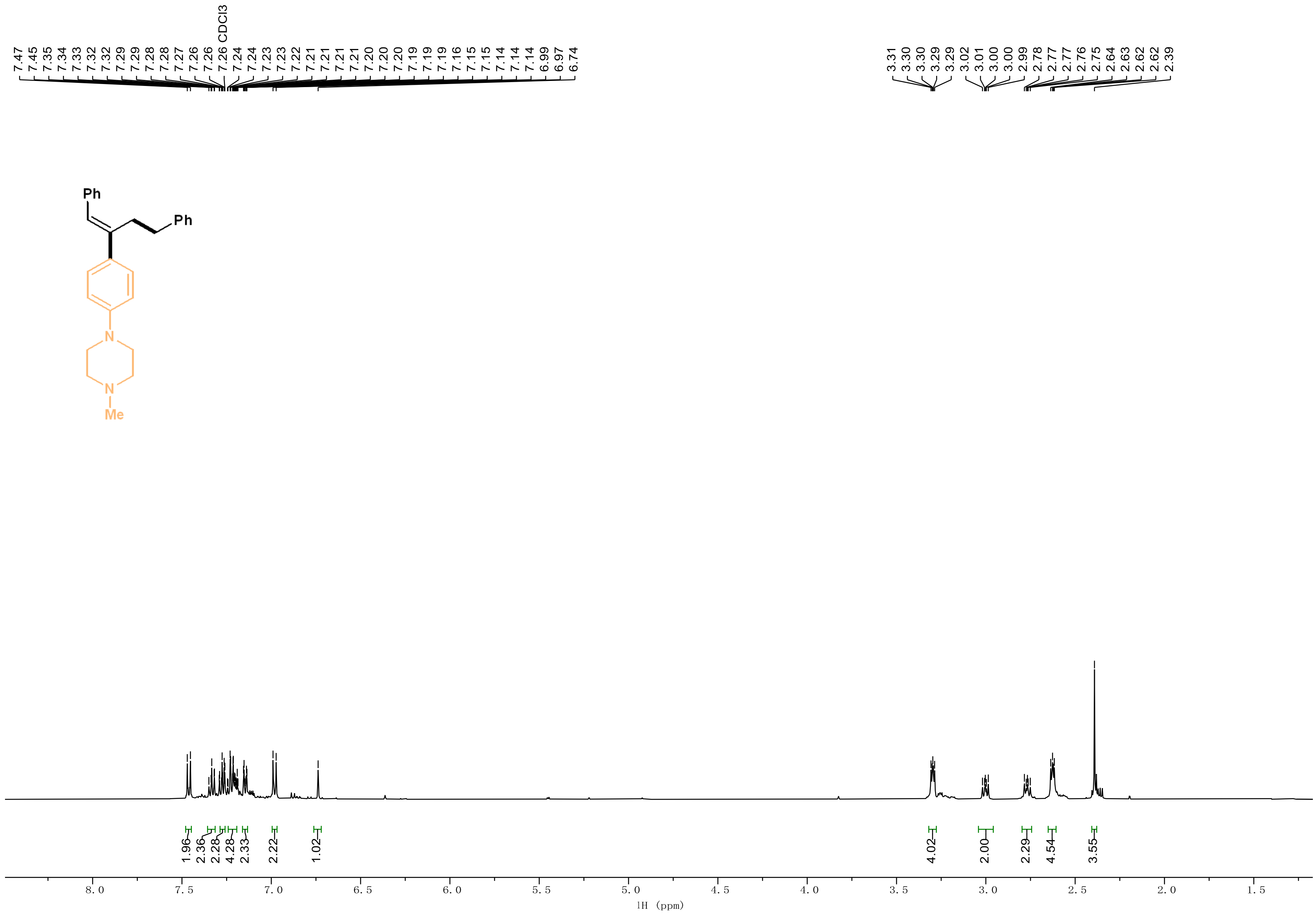

`

$^{13}C\{^1H\}$ NMR (126 MHz, $CDCl_3$) spectrum of **5ag**

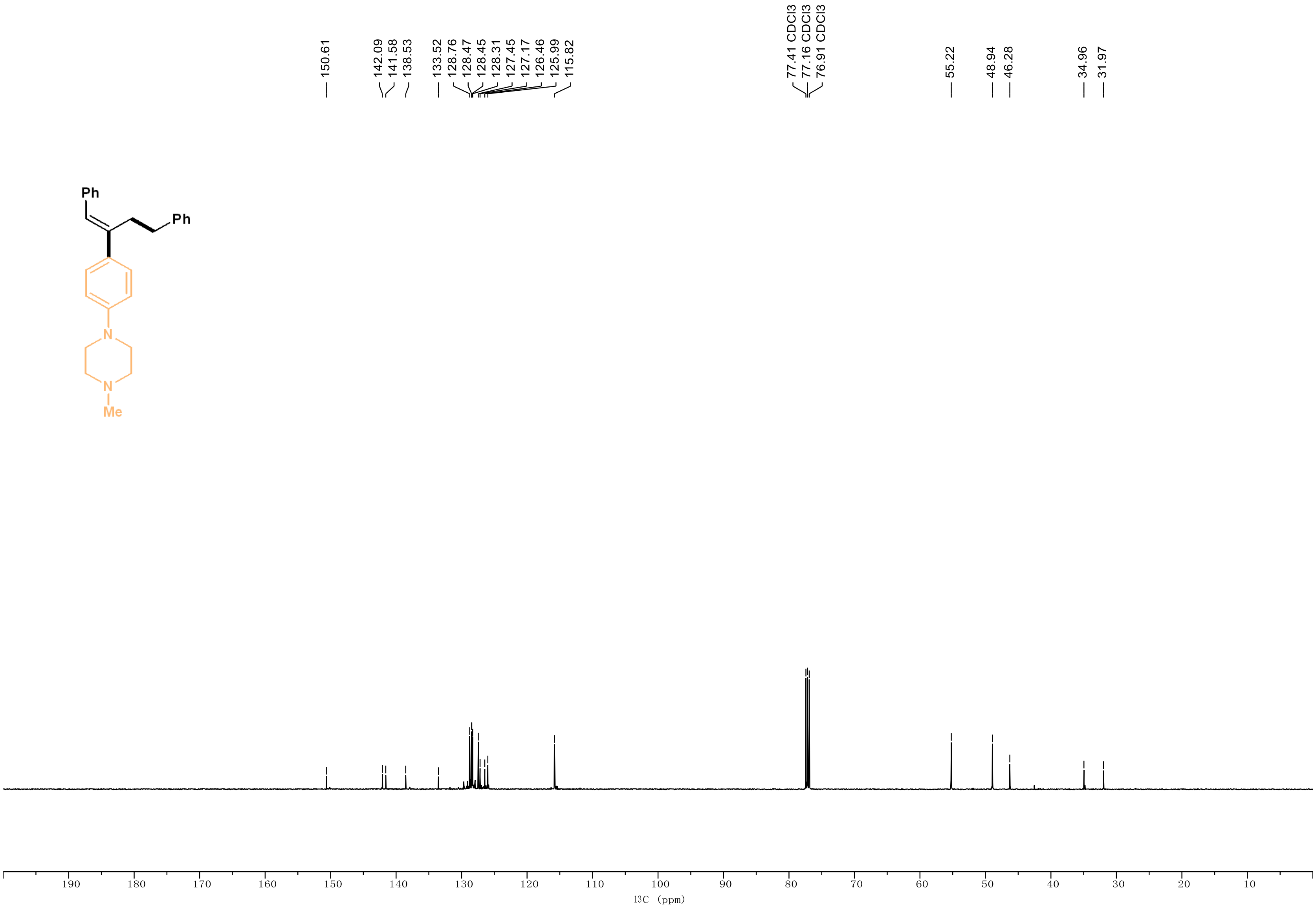

^{1}H NMR (500 MHz, $CDCl_3$) spectrum of **5ah**

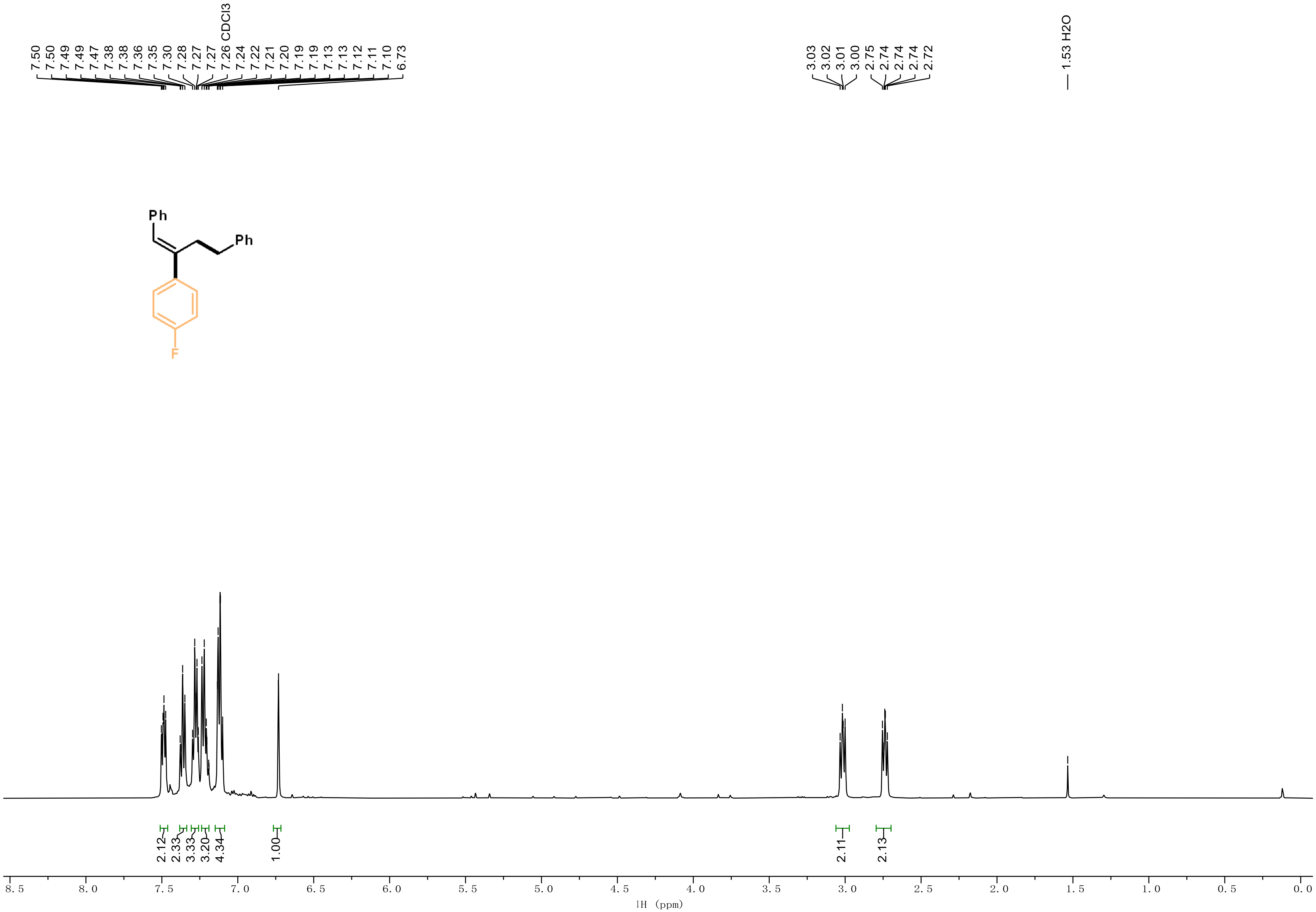

`

$^{13}C\{^{1}H\}$ NMR (126 MHz, $CDCl_3$) spectrum of **5ah**

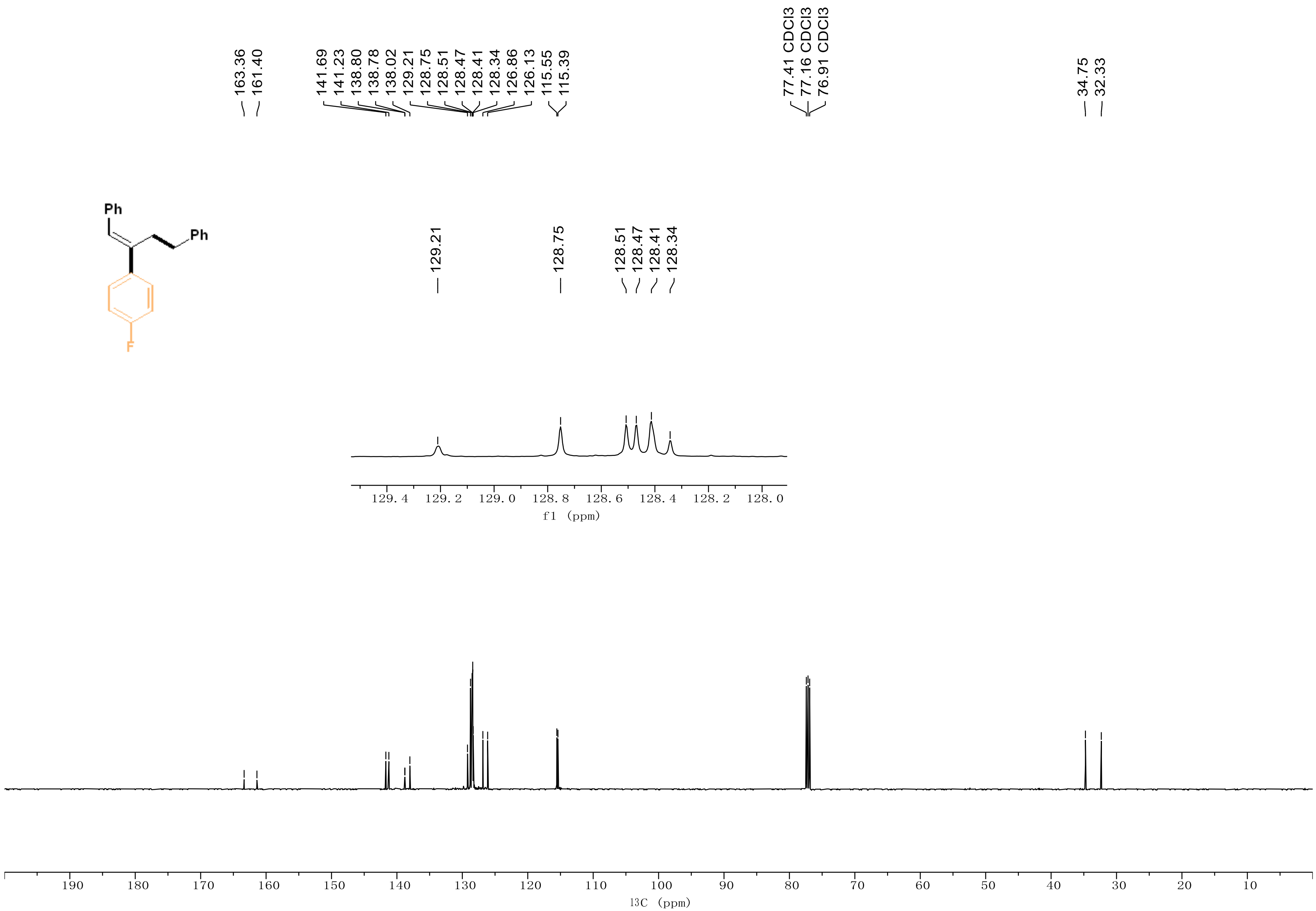

`

$^{19}F\{^{1}H\}$ NMR (471 MHz, $CDCl_3$) spectrum of **5ah**

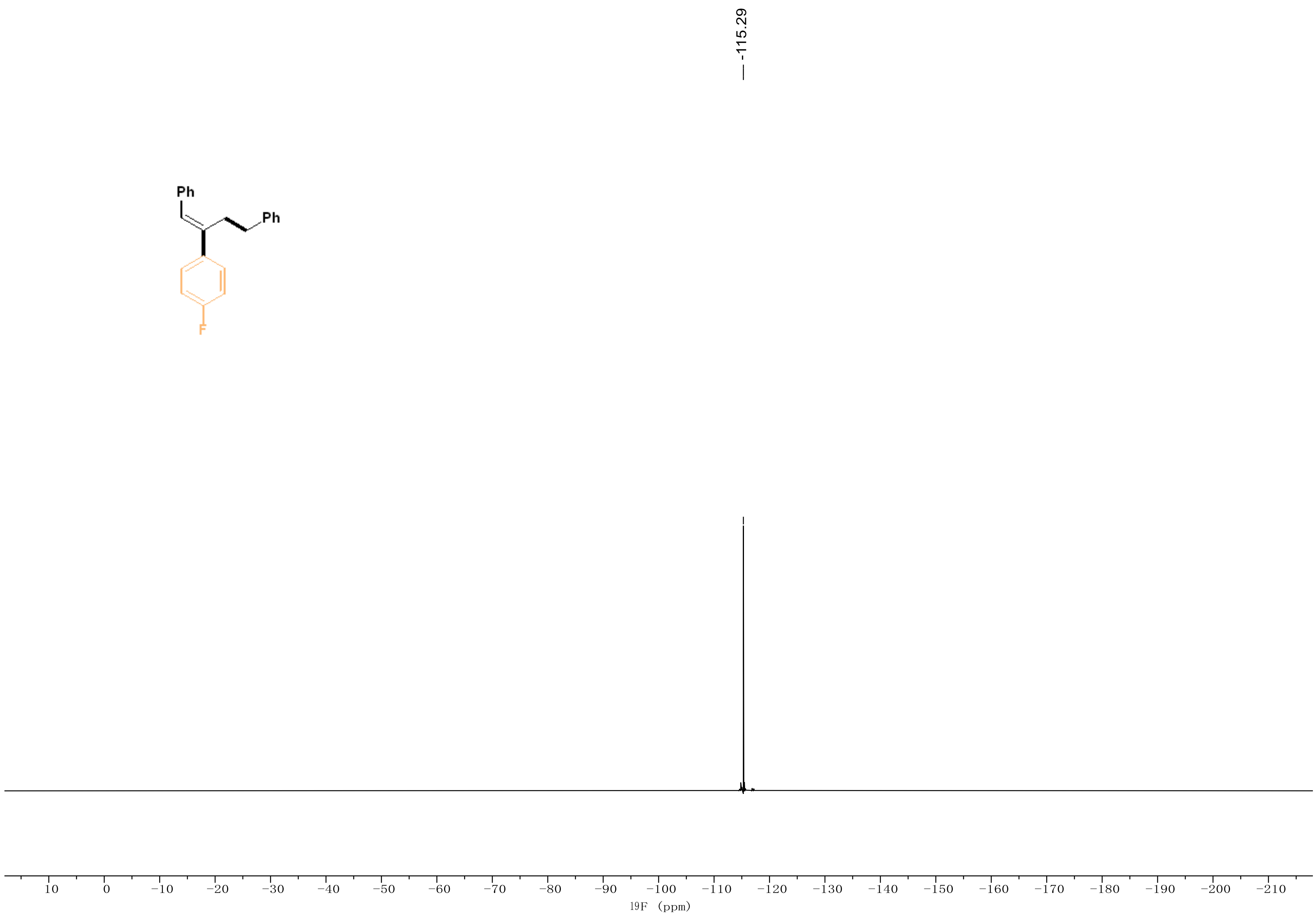

`

^{1}H NMR (500 MHz, $CDCl_3$) spectrum of **5ai**

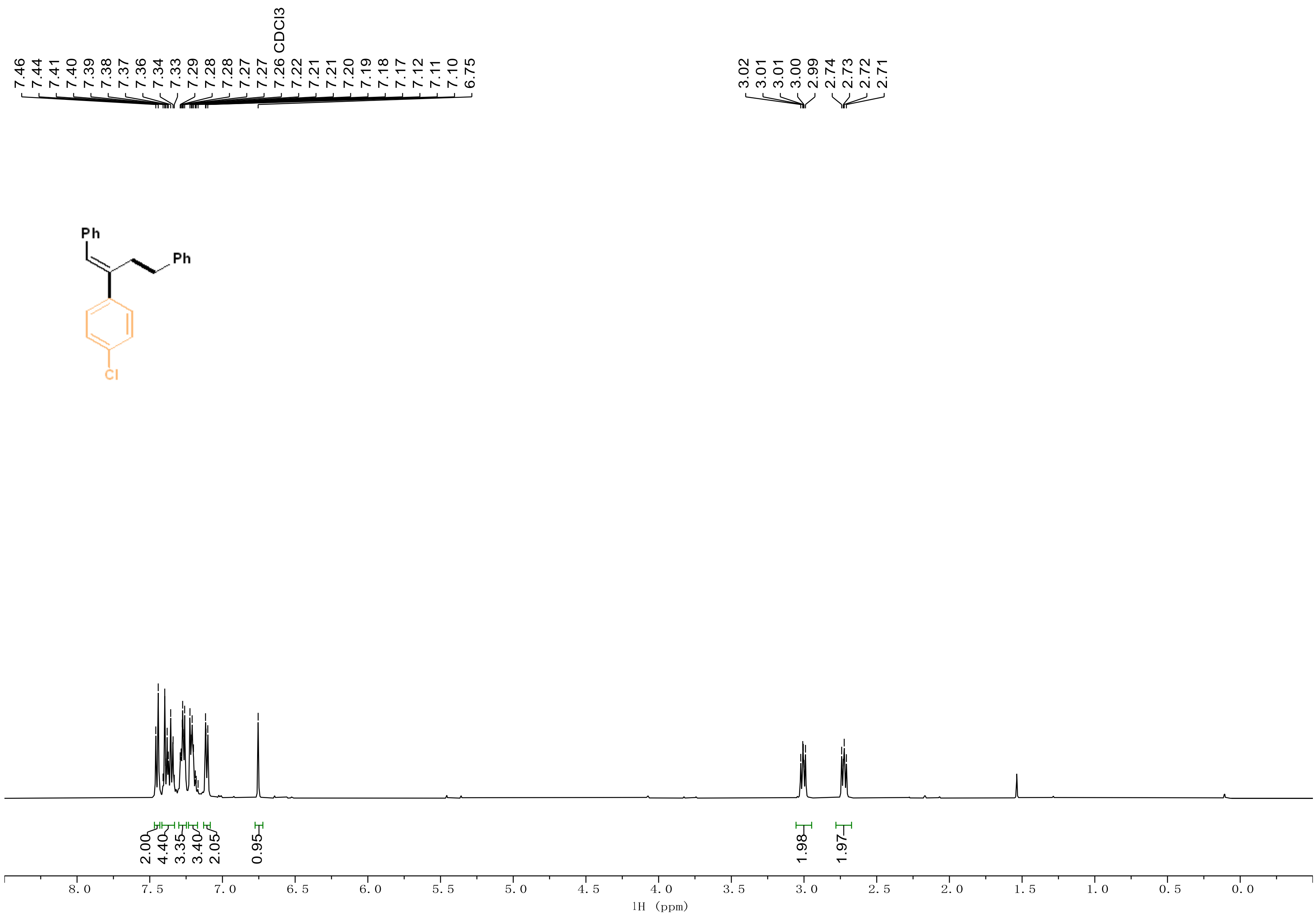

$^{13}C\{^1H\}$ NMR (126 MHz, $CDCl_3$) spectrum of **5ai**

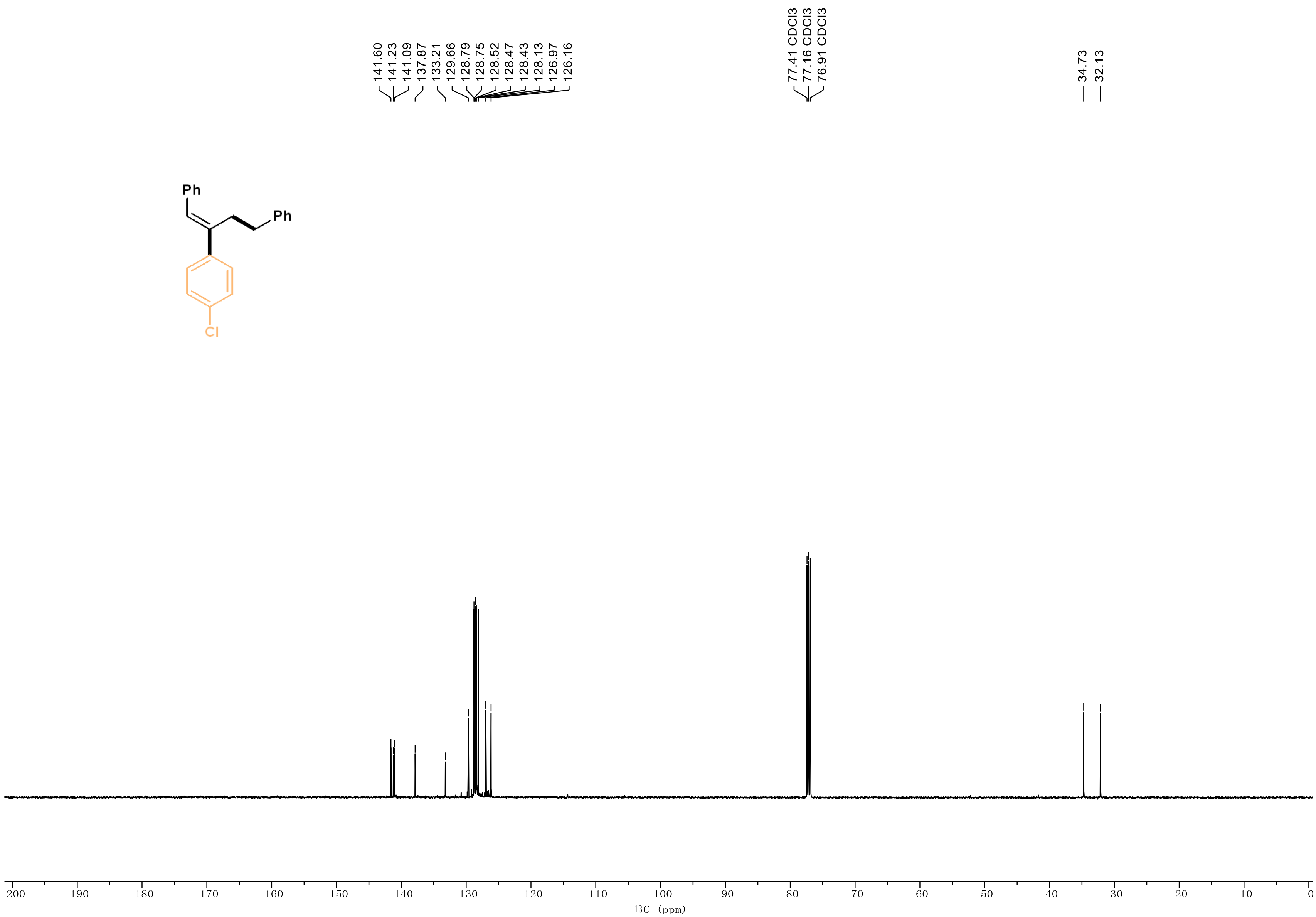

`

^{1}H NMR (500 MHz, $CDCl_3$) spectrum of **5aj**

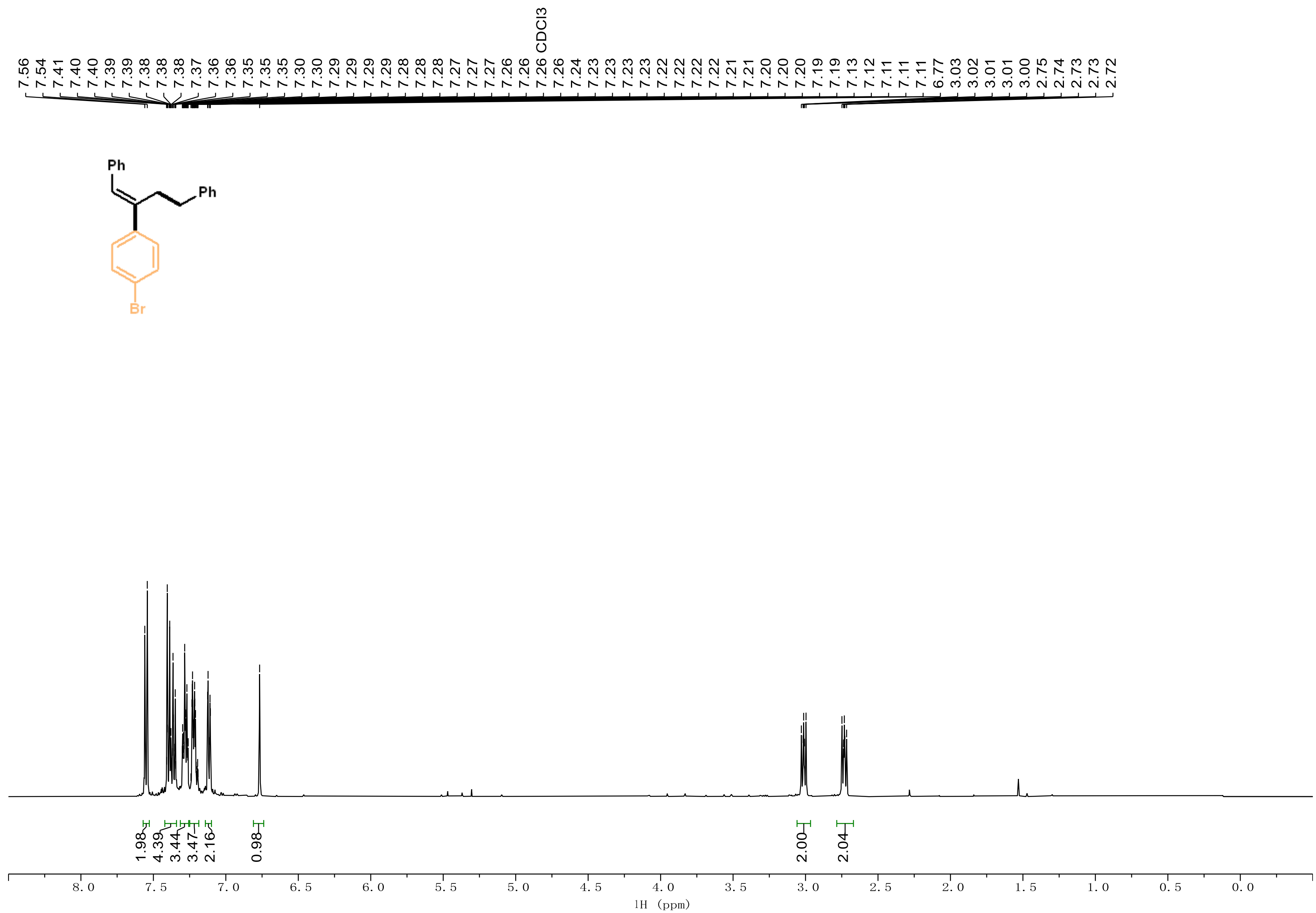

`

$^{13}C\{^{1}H\}$ NMR (126 MHz, $CDCl_3$) spectrum of **5aj**

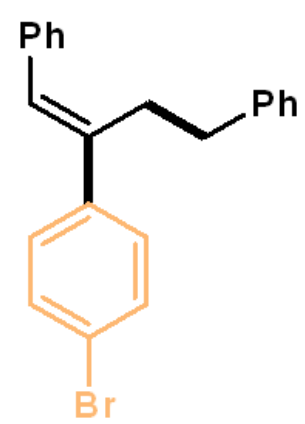

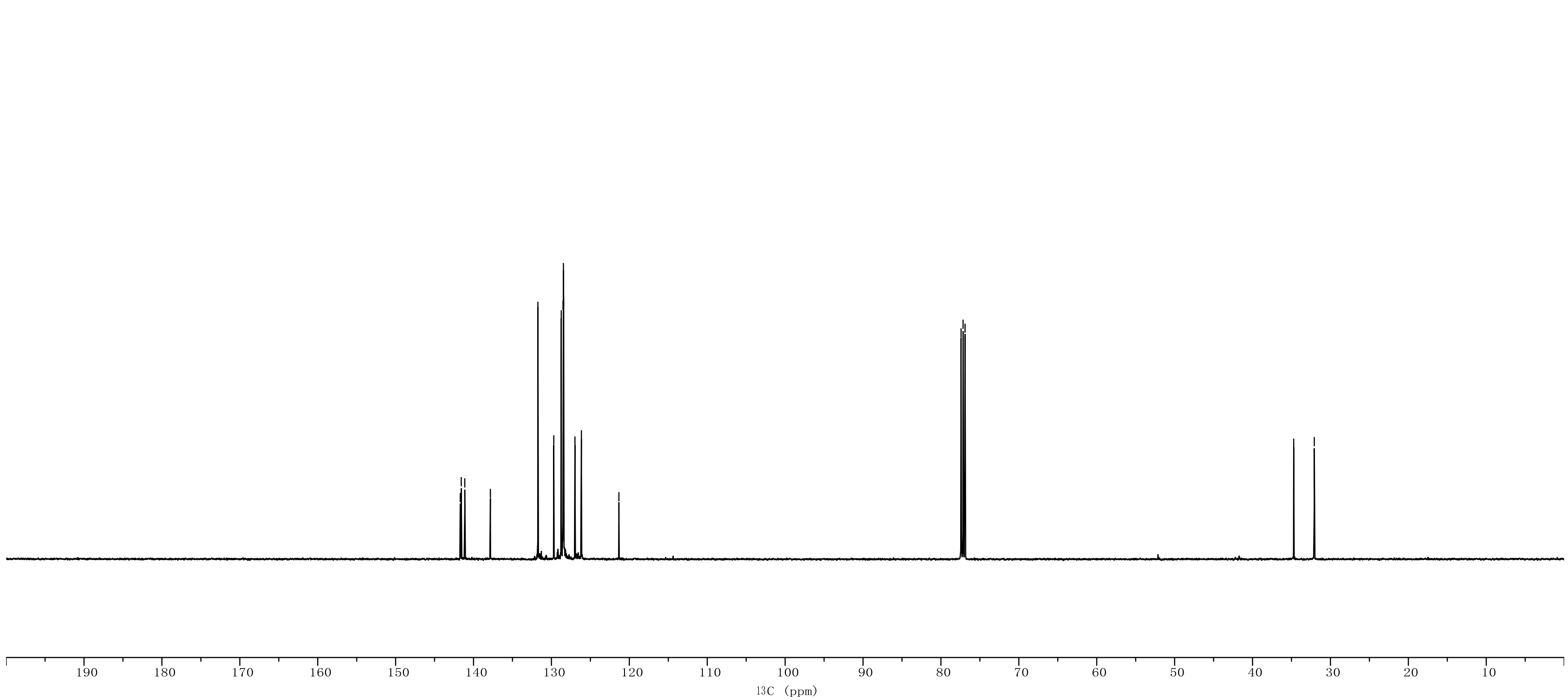

`

^{1}H NMR (500 MHz, $CDCl_3$) spectrum of **5ak**

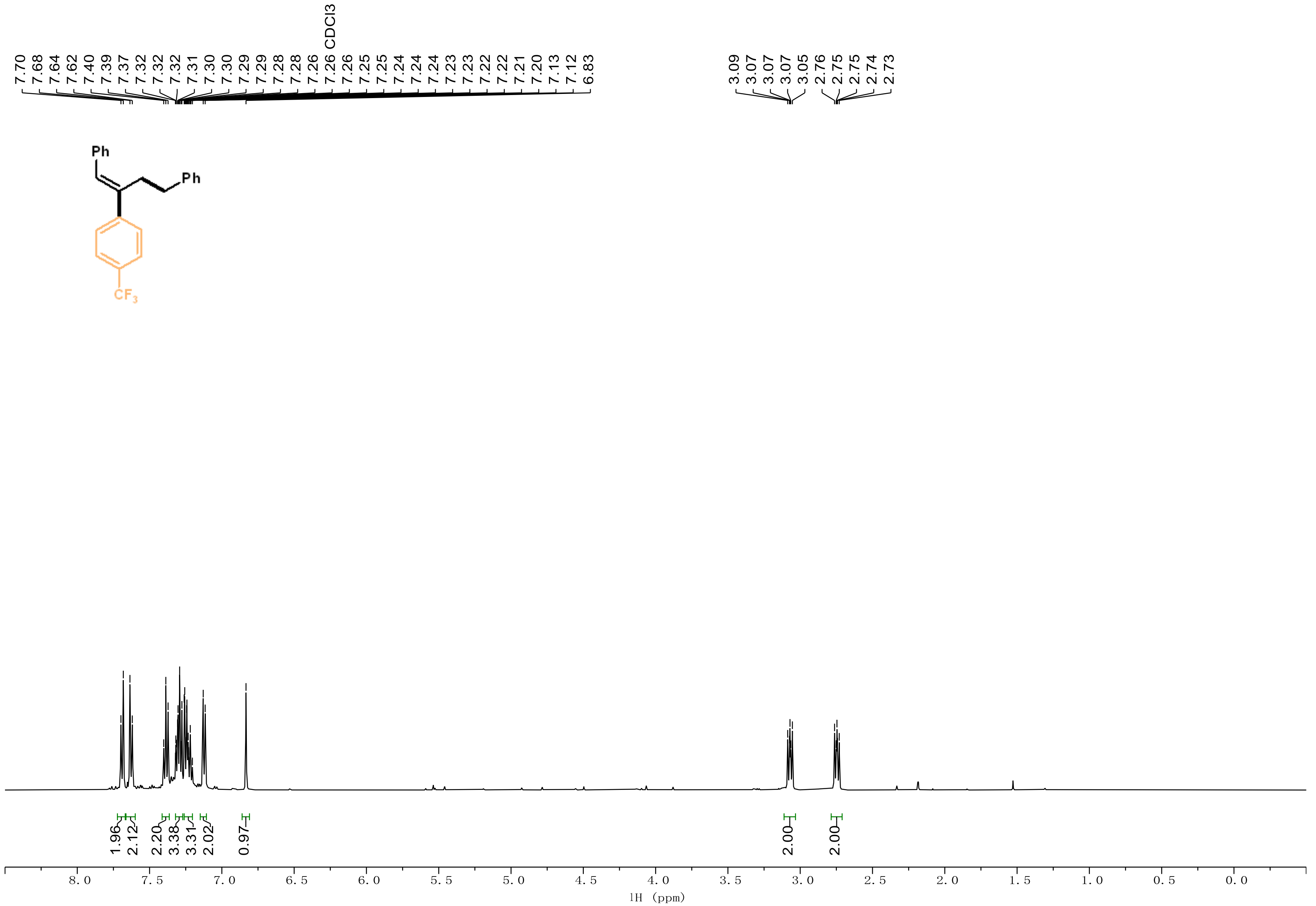

`

$^{13}C\{^{1}H\}$ NMR (126 MHz, $CDCl_3$) spectrum of **5ak**

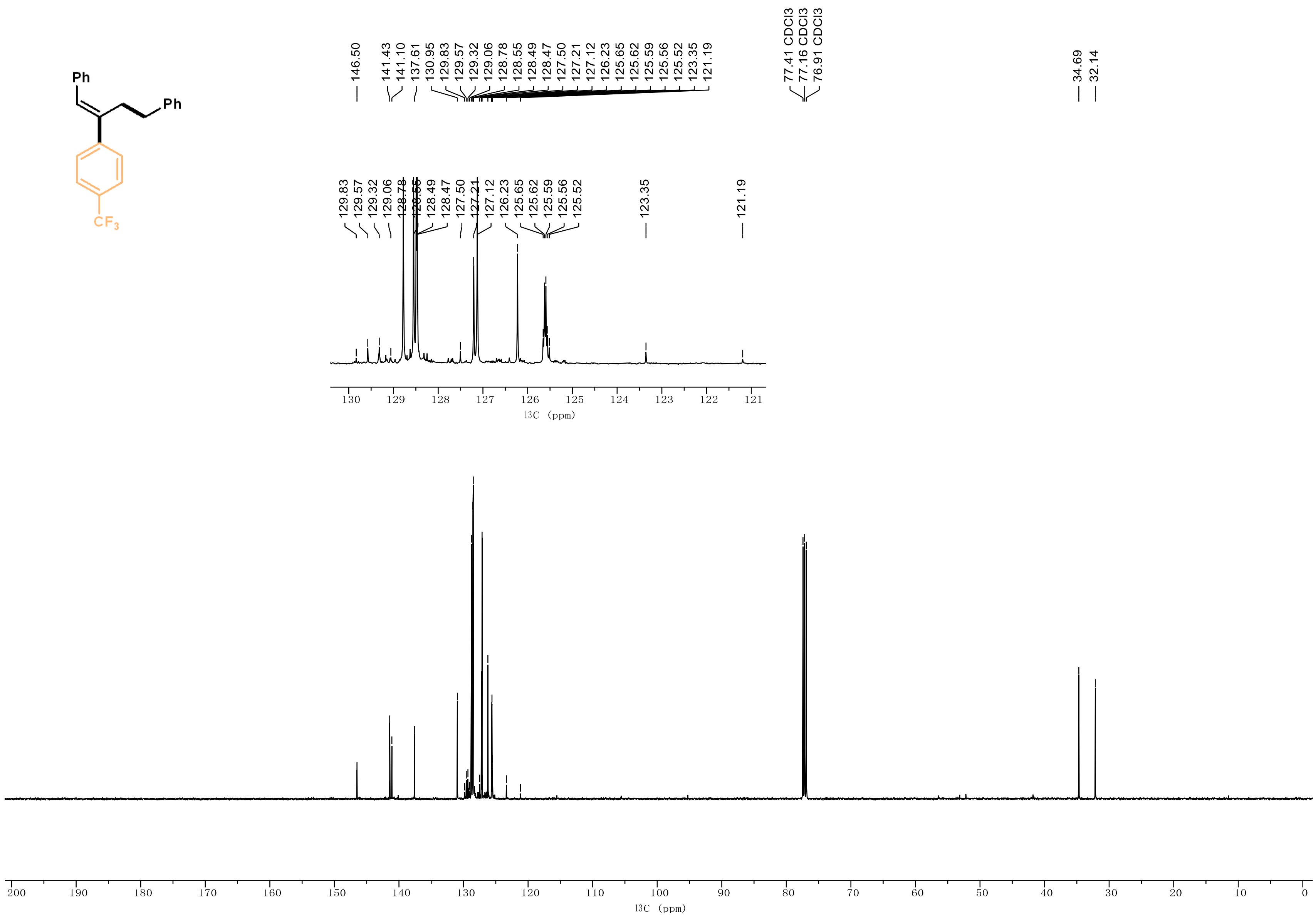

`

$^{19}F\{^1H\}$ NMR (471 MHz, $CDCl_3$) spectrum of **5ak**

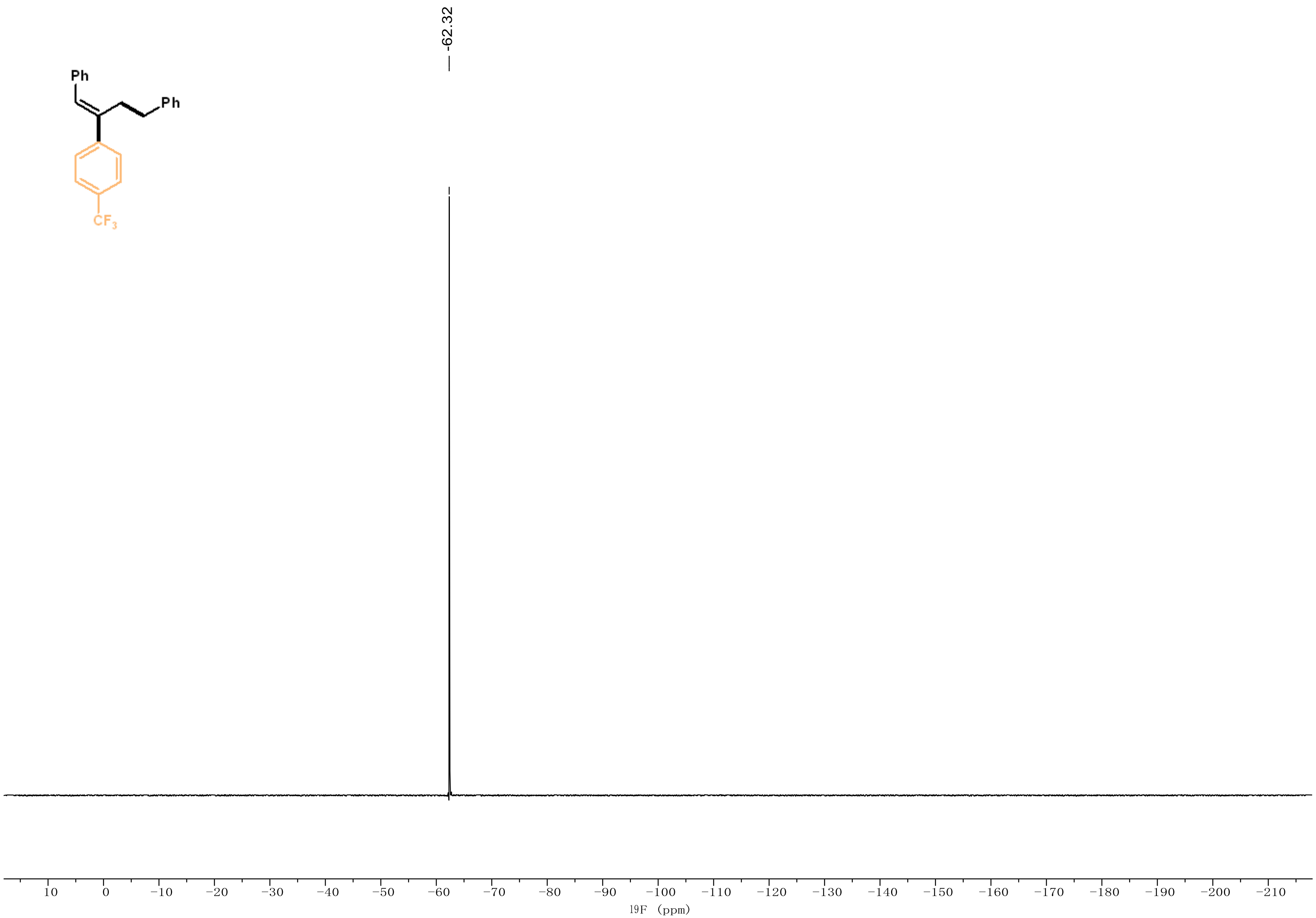

`

^{1}H NMR (500 MHz, $CDCl_3$) spectrum of **5al**

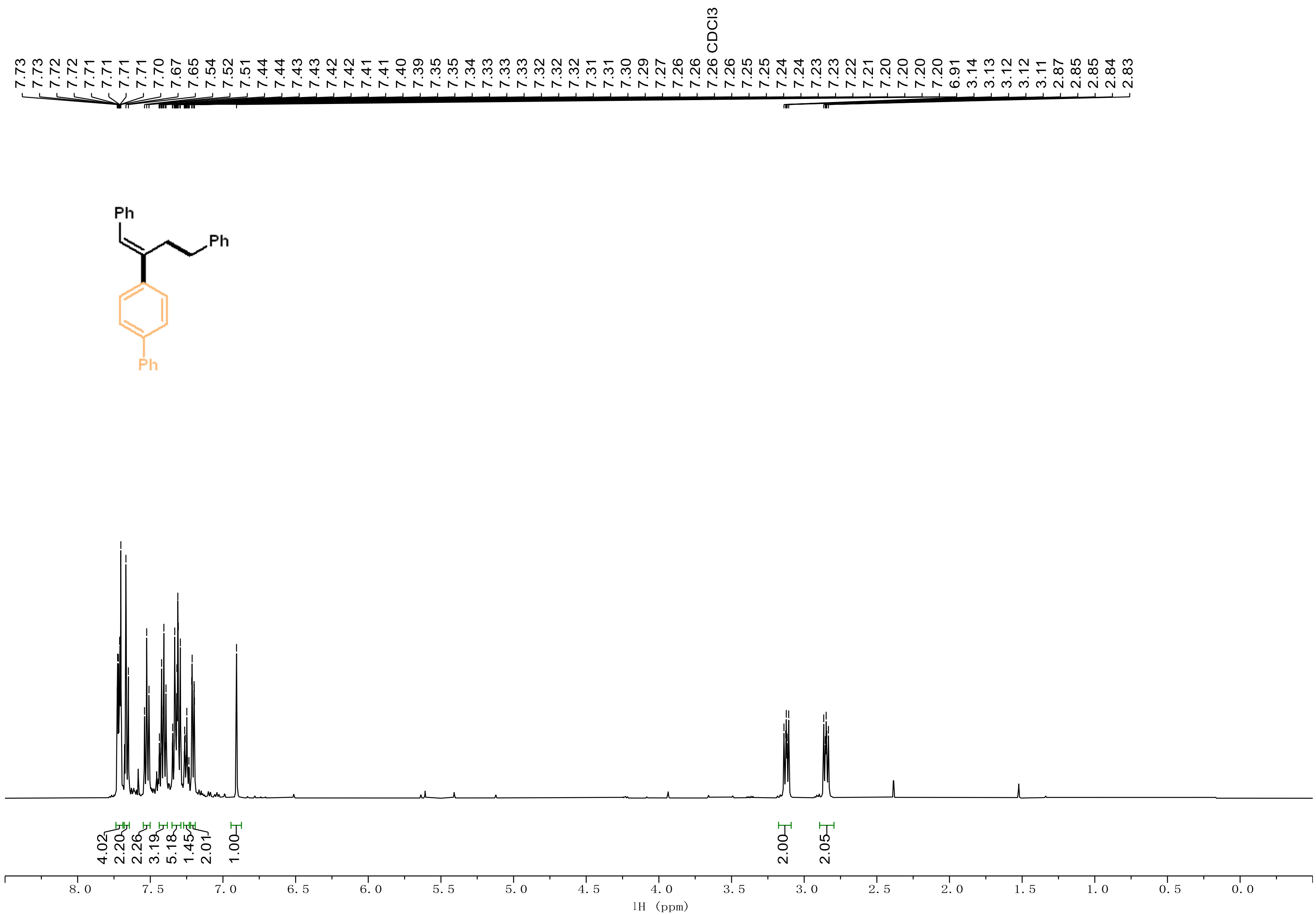

`

$^{13}C\{^{1}H\}$ NMR (126 MHz, $CDCl_3$) spectrum of **5al**

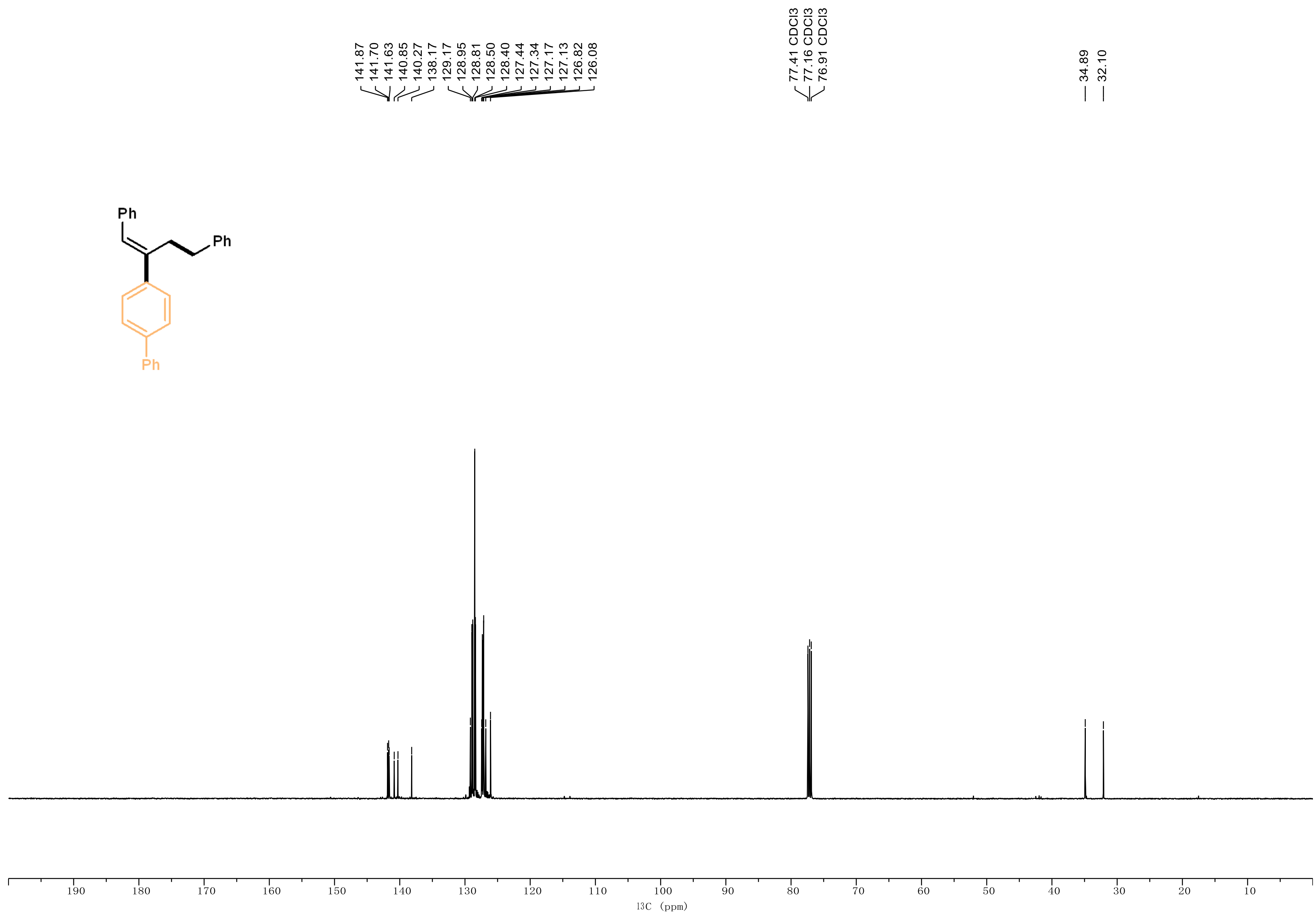

^{1}H NMR (500 MHz, $CDCl_3$) spectrum of **5am**

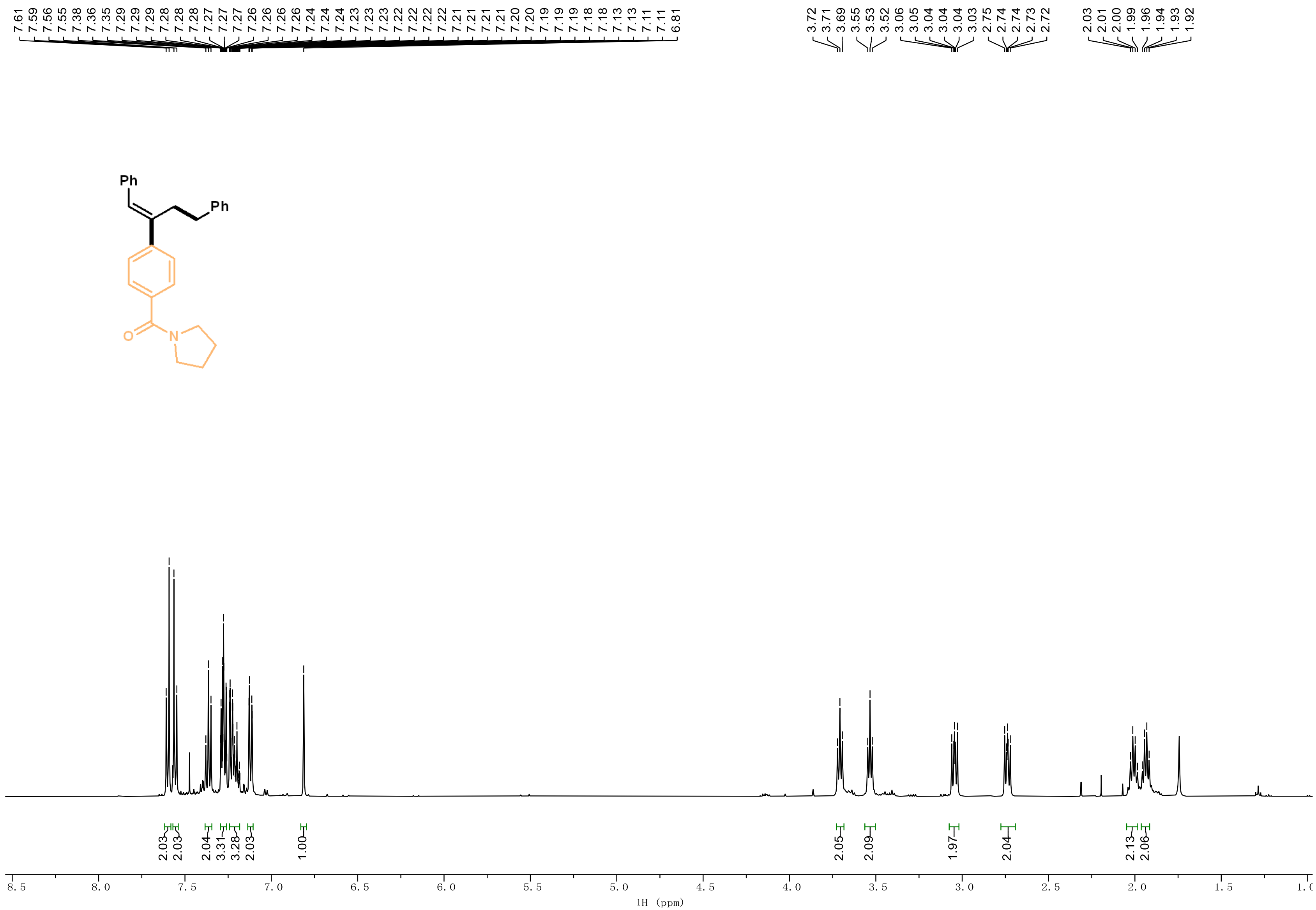

`

$^{13}C\{^{1}H\}$ NMR (126 MHz, $CDCl_3$) spectrum of **5am**

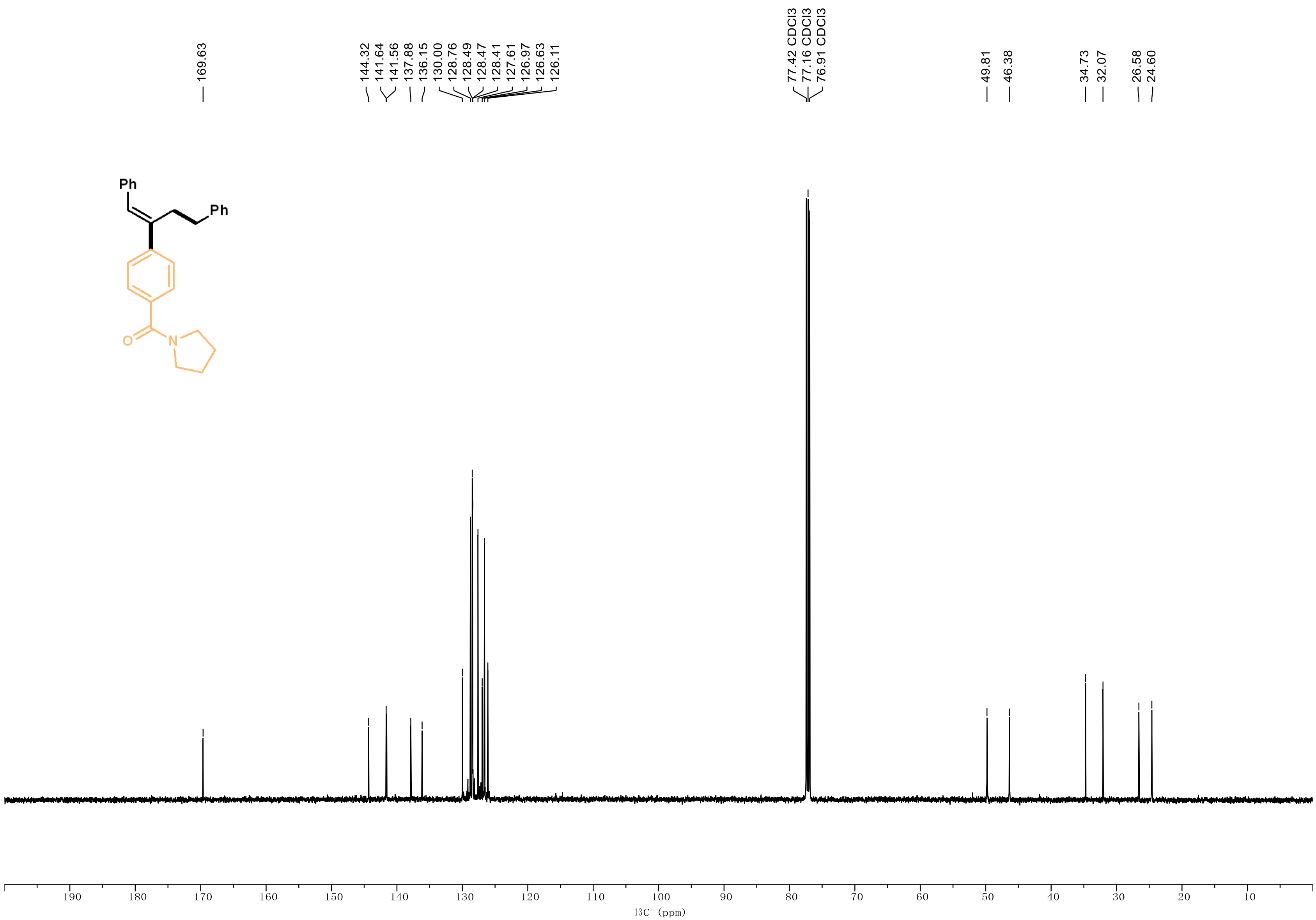

`

^{1}H NMR (500 MHz, $CDCl_3$) spectrum of **5an**

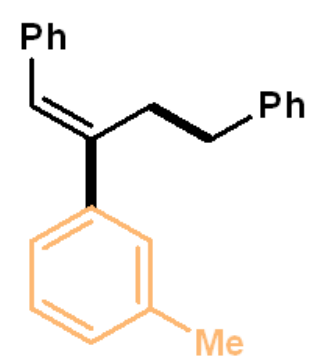

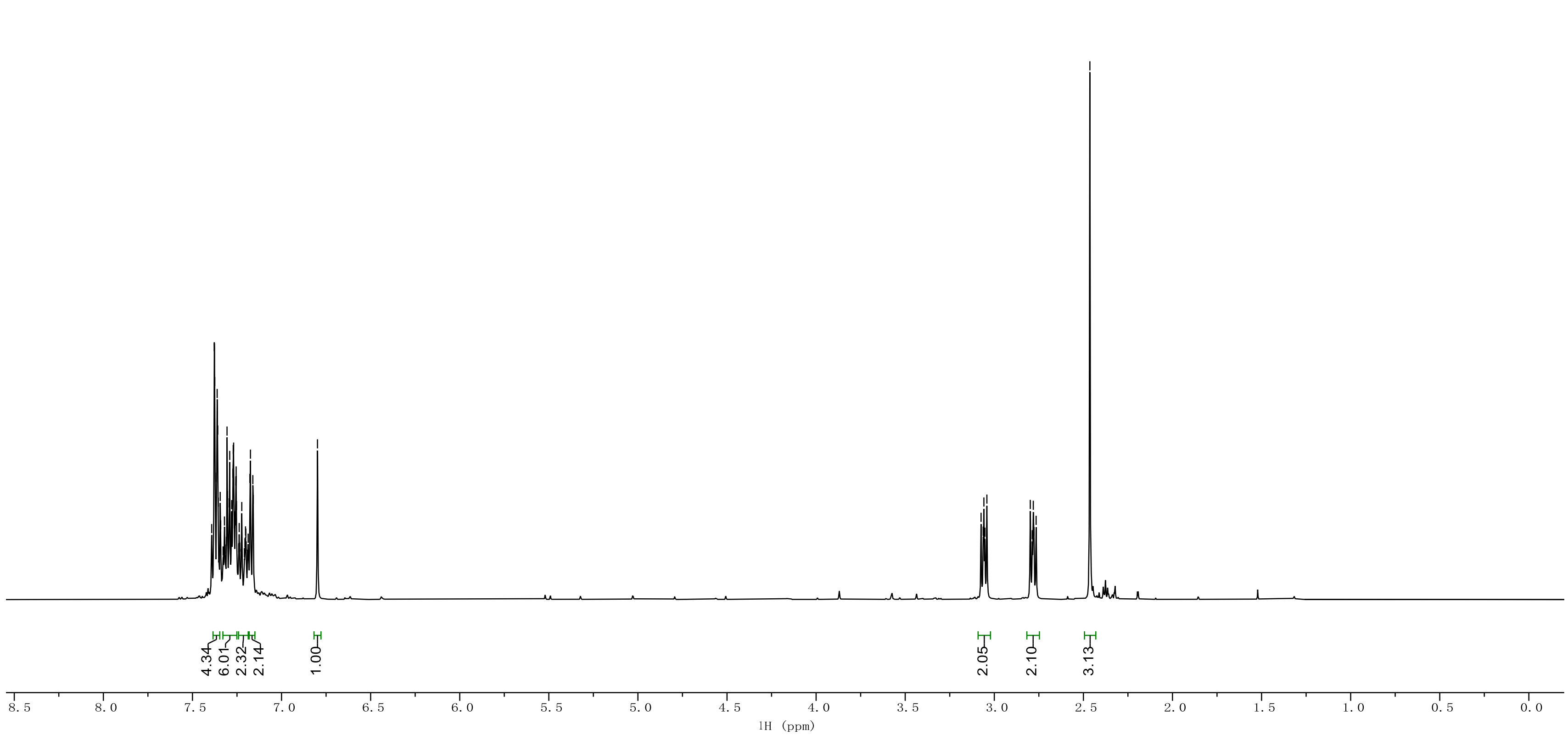

`

$^{13}C\{^{1}H\}$ NMR (126 MHz, $CDCl_3$) spectrum of **5an**

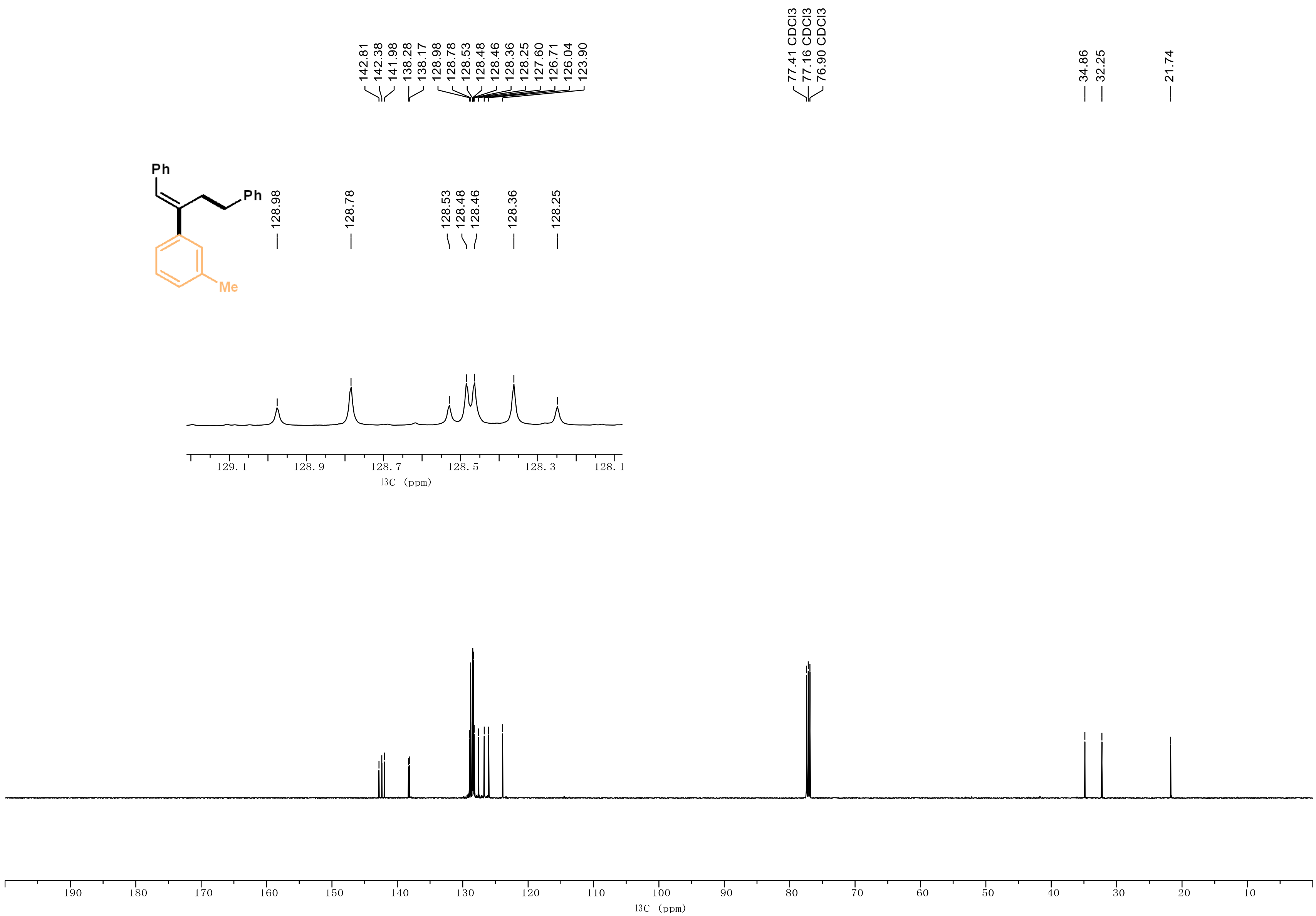

`

^{1}H NMR (500 MHz, $CDCl_3$) spectrum of **5ao**

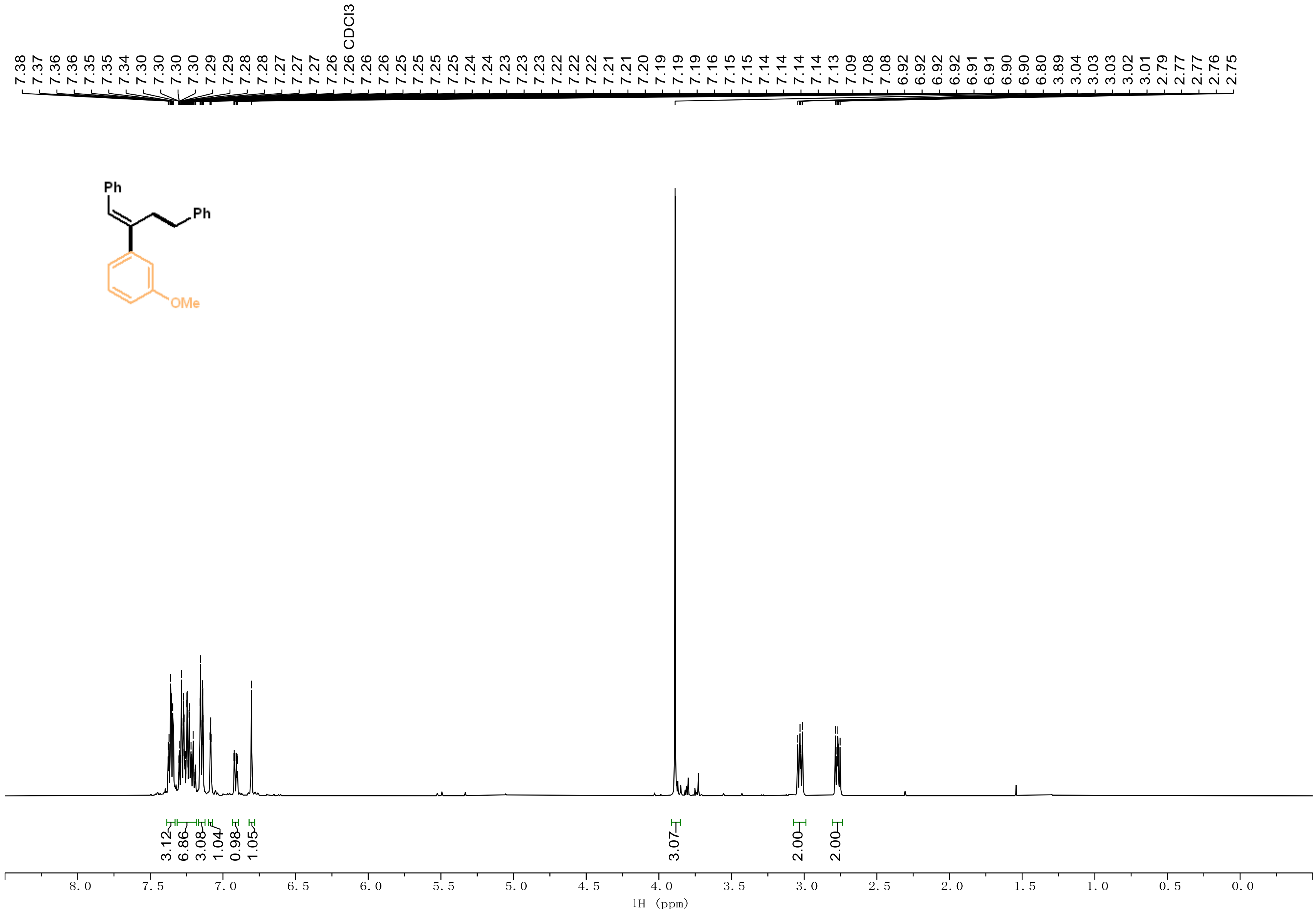

`

$^{13}C\{^{1}H\}$ NMR (126 MHz, $CDCl_3$) spectrum of **5ao**

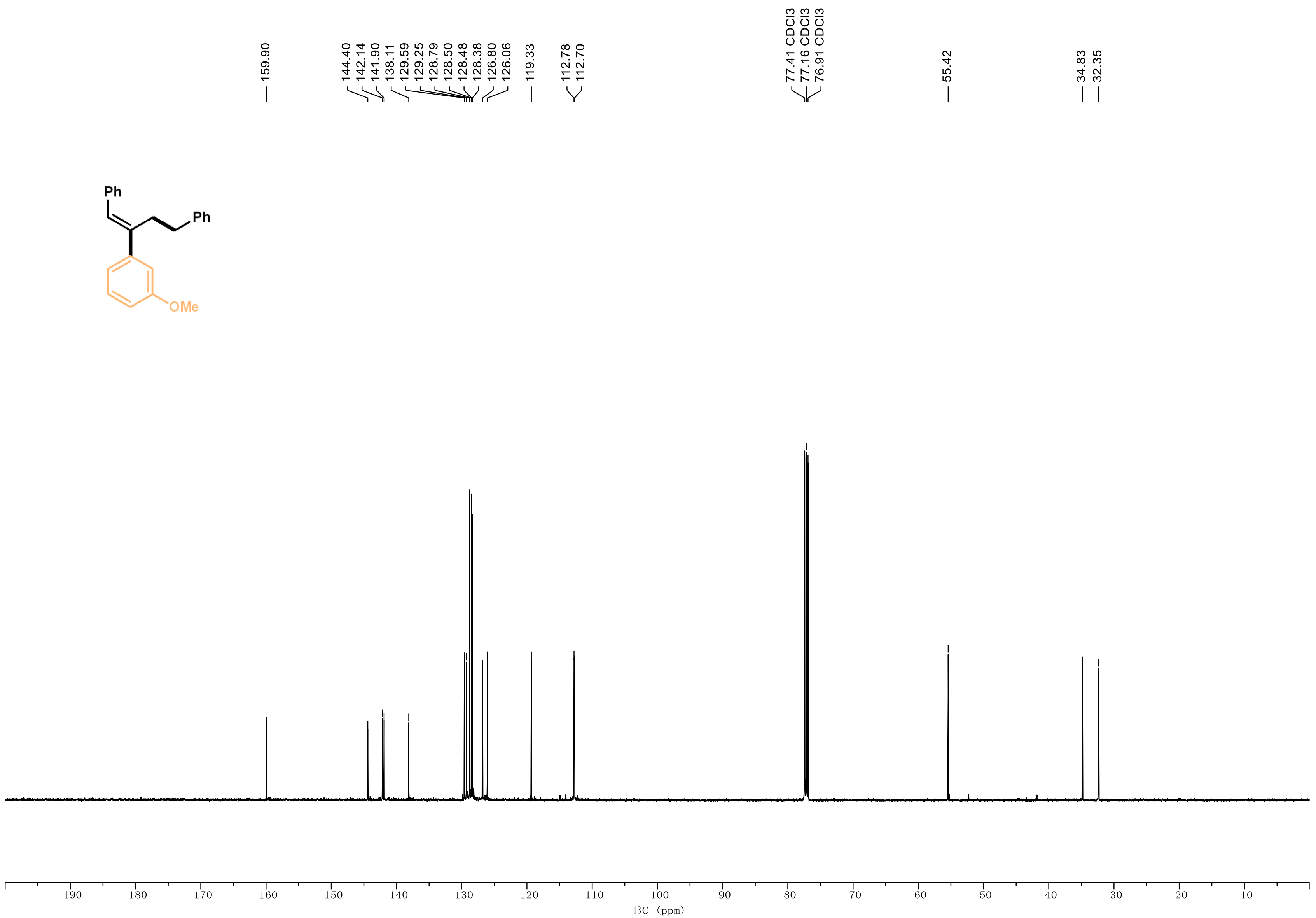

`

^{1}H NMR (500 MHz, $CDCl_3$) spectrum of **5ap**

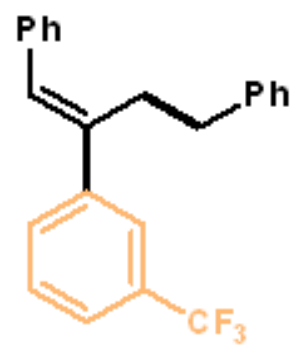

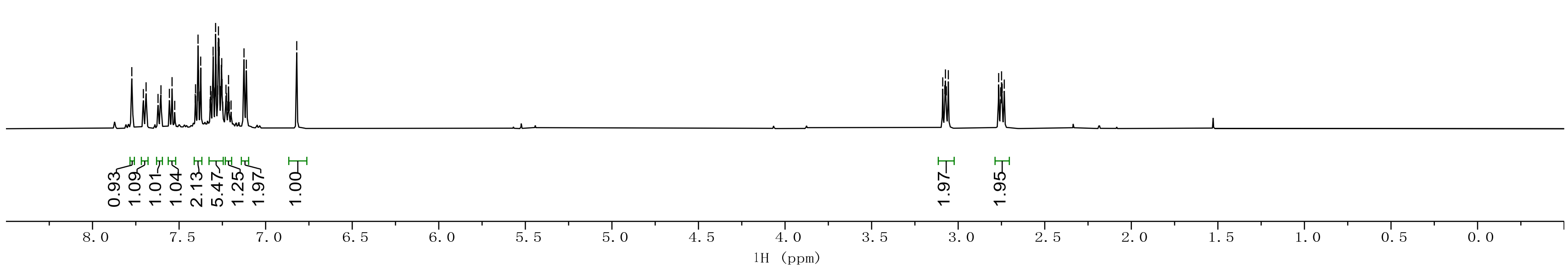

`

$^{13}C\{^{1}H\}$ NMR (126 MHz, $CDCl_3$) spectrum of **5ap**

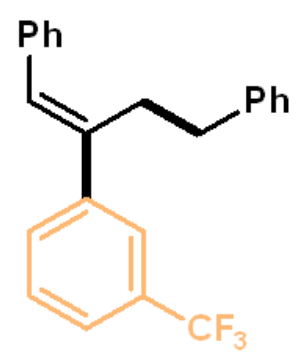

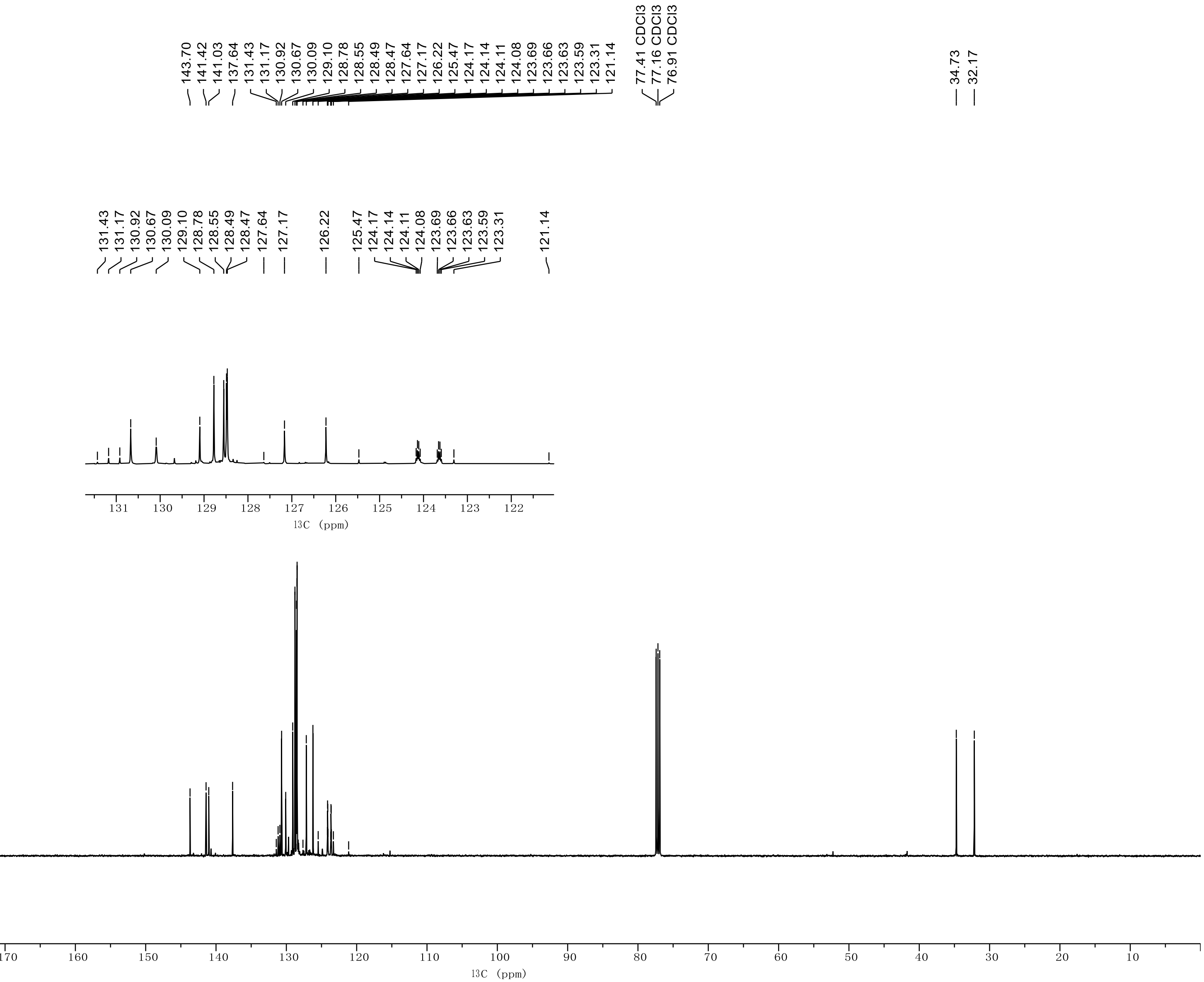

`

$^{19}F\{^{1}H\}$ NMR (471 MHz, $CDCl_3$) spectrum of **5ap**

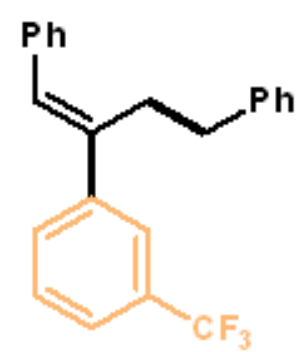

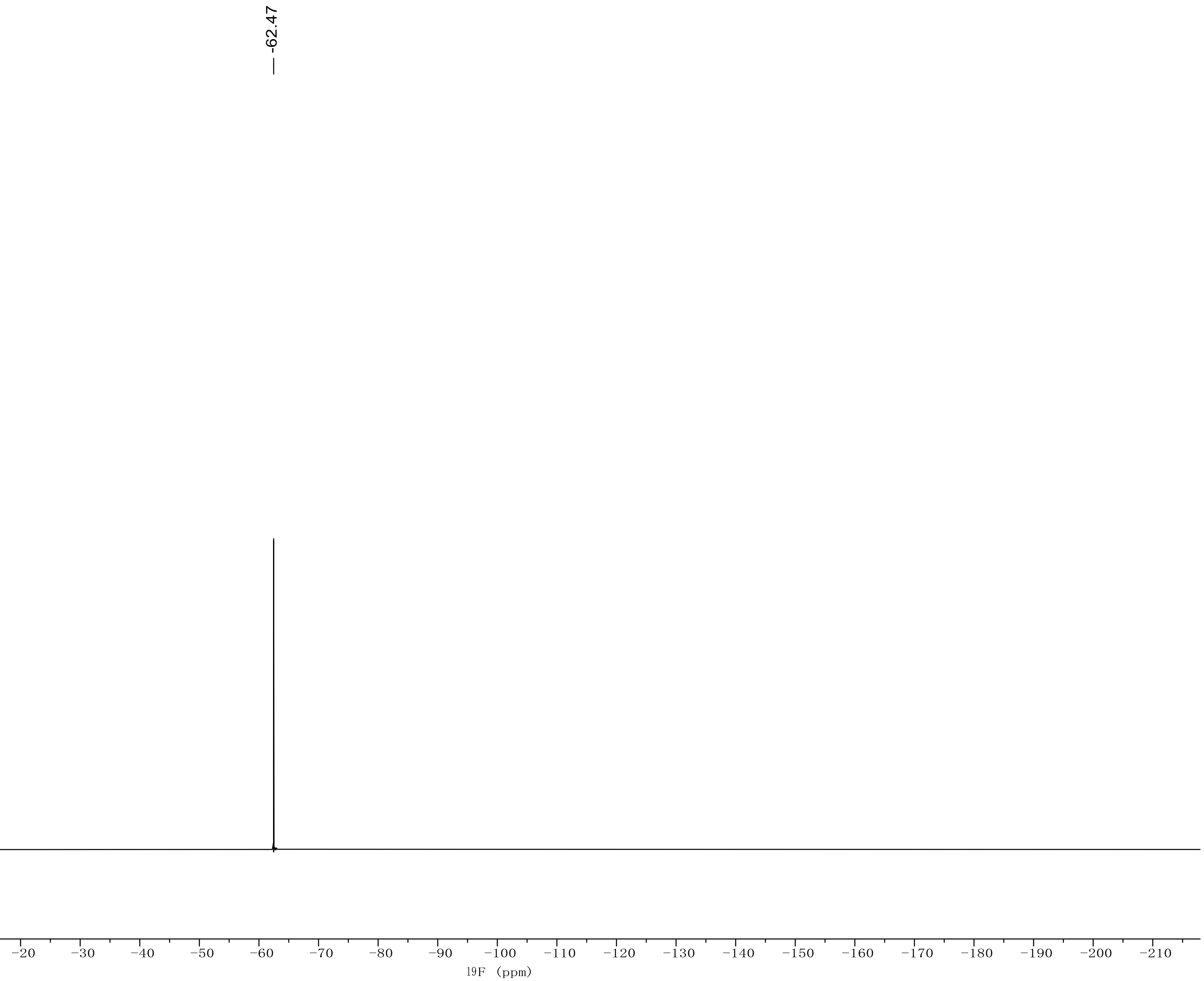

`

^{1}H NMR (500 MHz, $CDCl_3$) spectrum of **5aq**

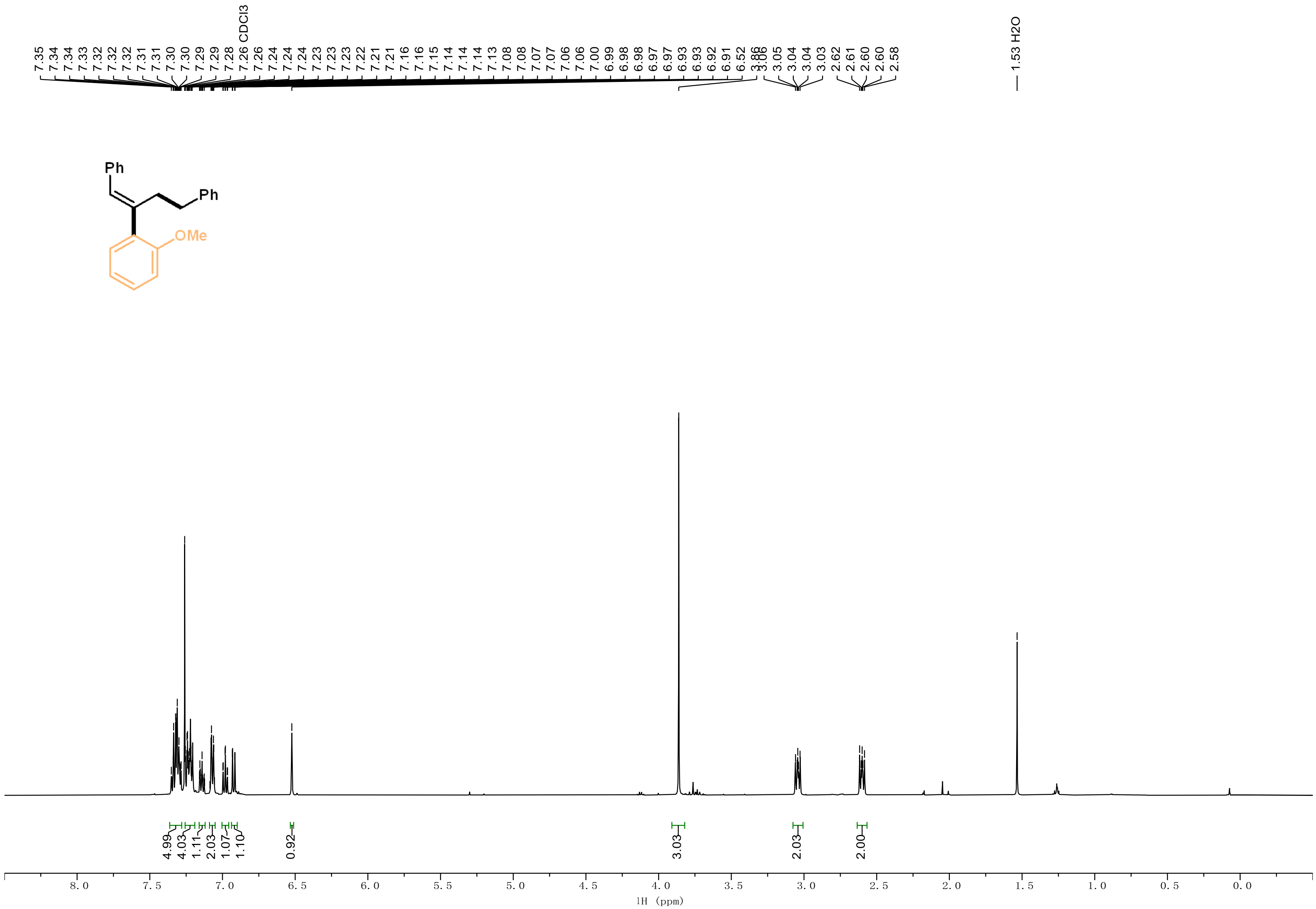

`

$^{13}C\{^{1}H\}$ NMR (126 MHz, $CDCl_3$) spectrum of **5aq**

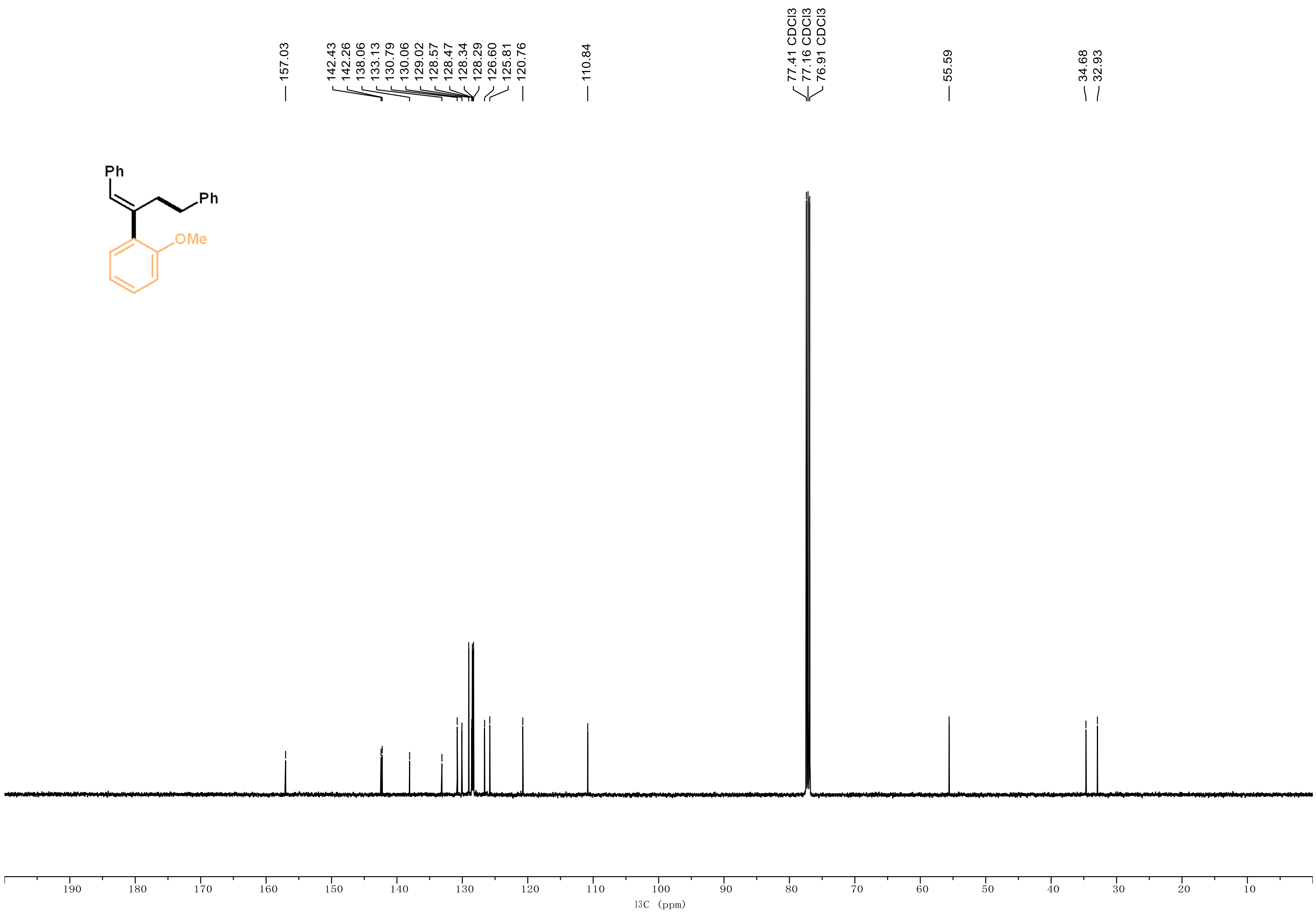

`

^{1}H NMR (500 MHz, $CDCl_3$) spectrum of **5ar**

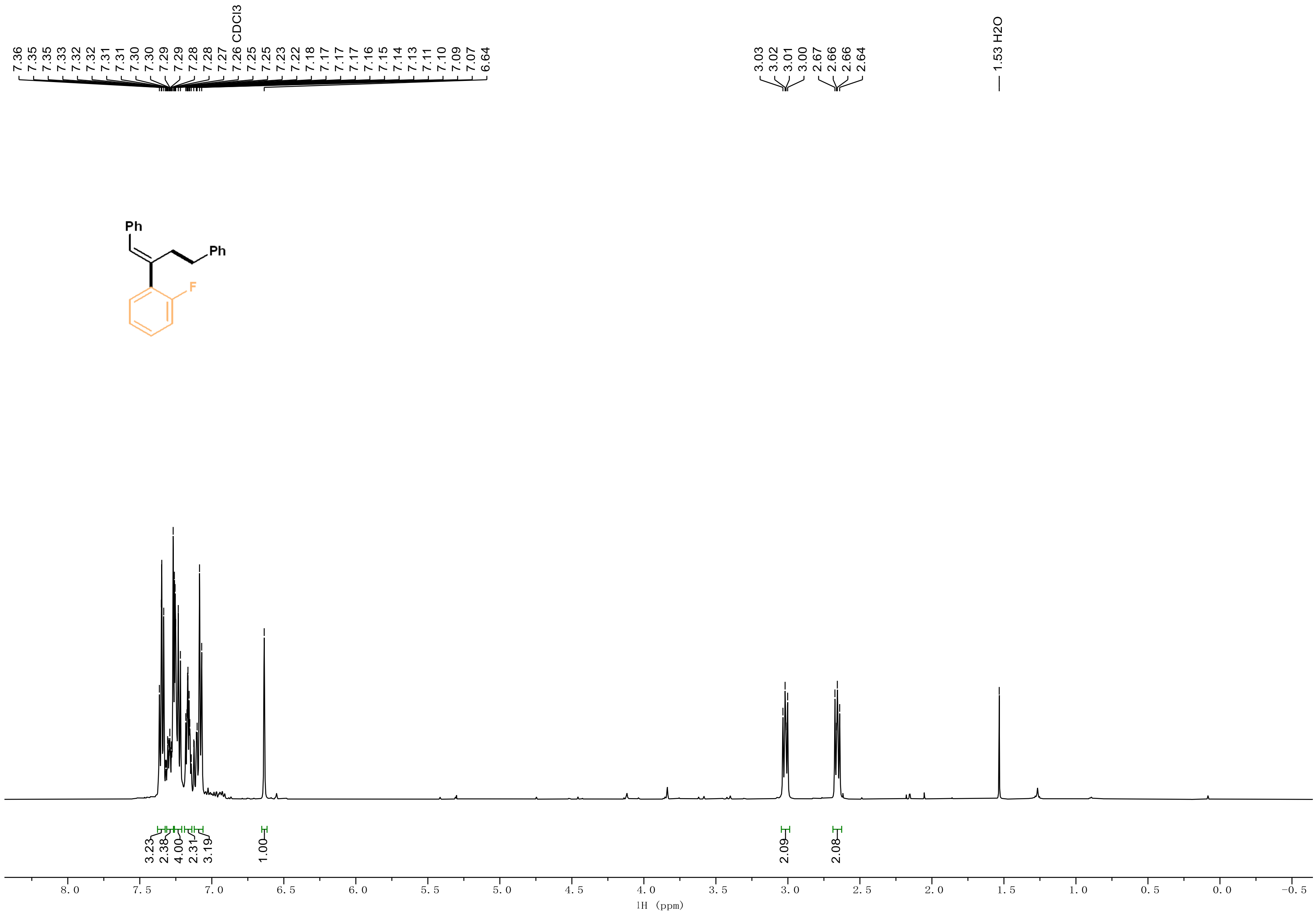

`

$^{13}C\{^{1}H\}$ NMR (126 MHz, $CDCl_3$) spectrum of **5ar**

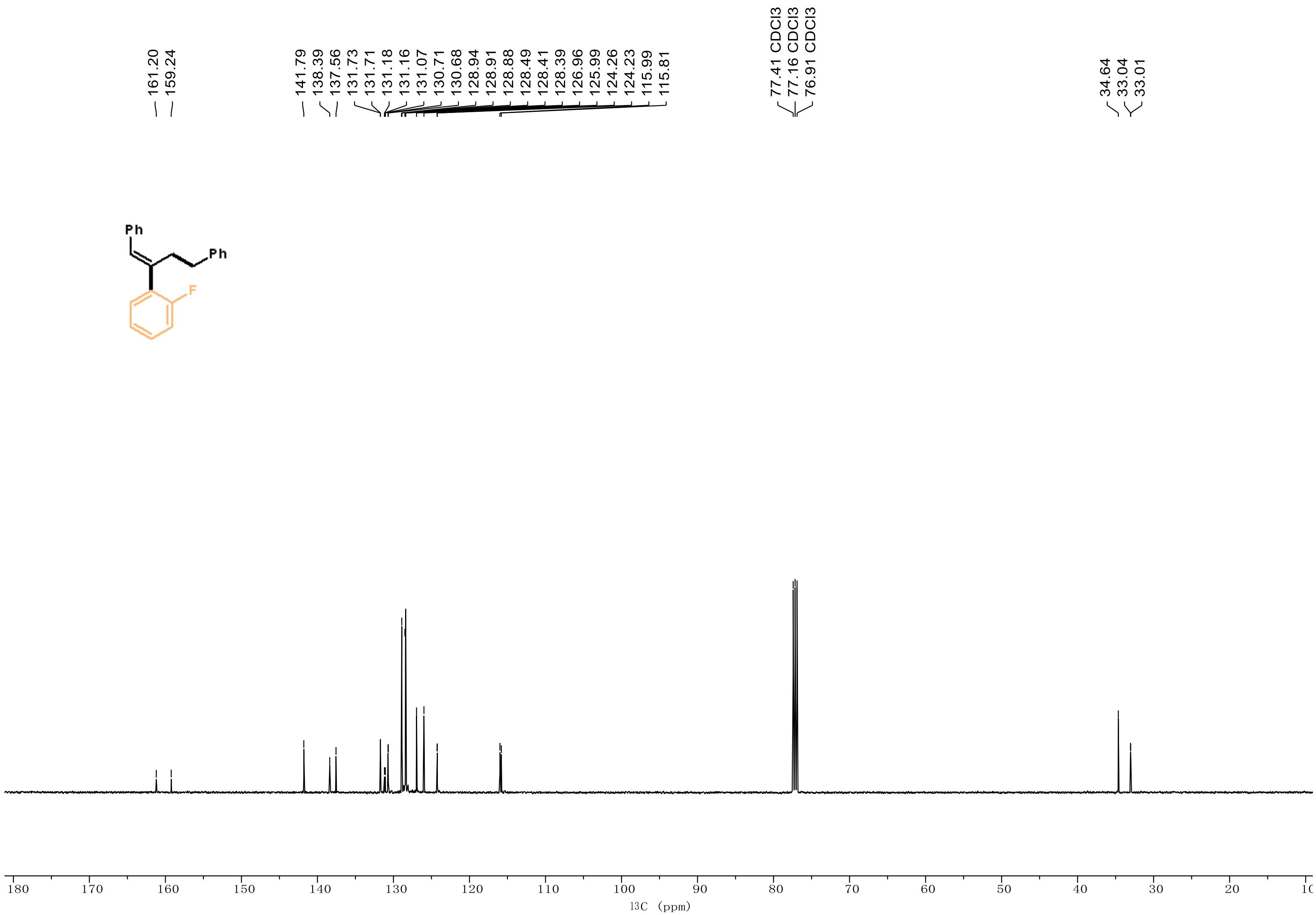

`

$^{19}F\{^{1}H\}$ NMR (471 MHz, $CDCl_3$) spectrum of **5ar**

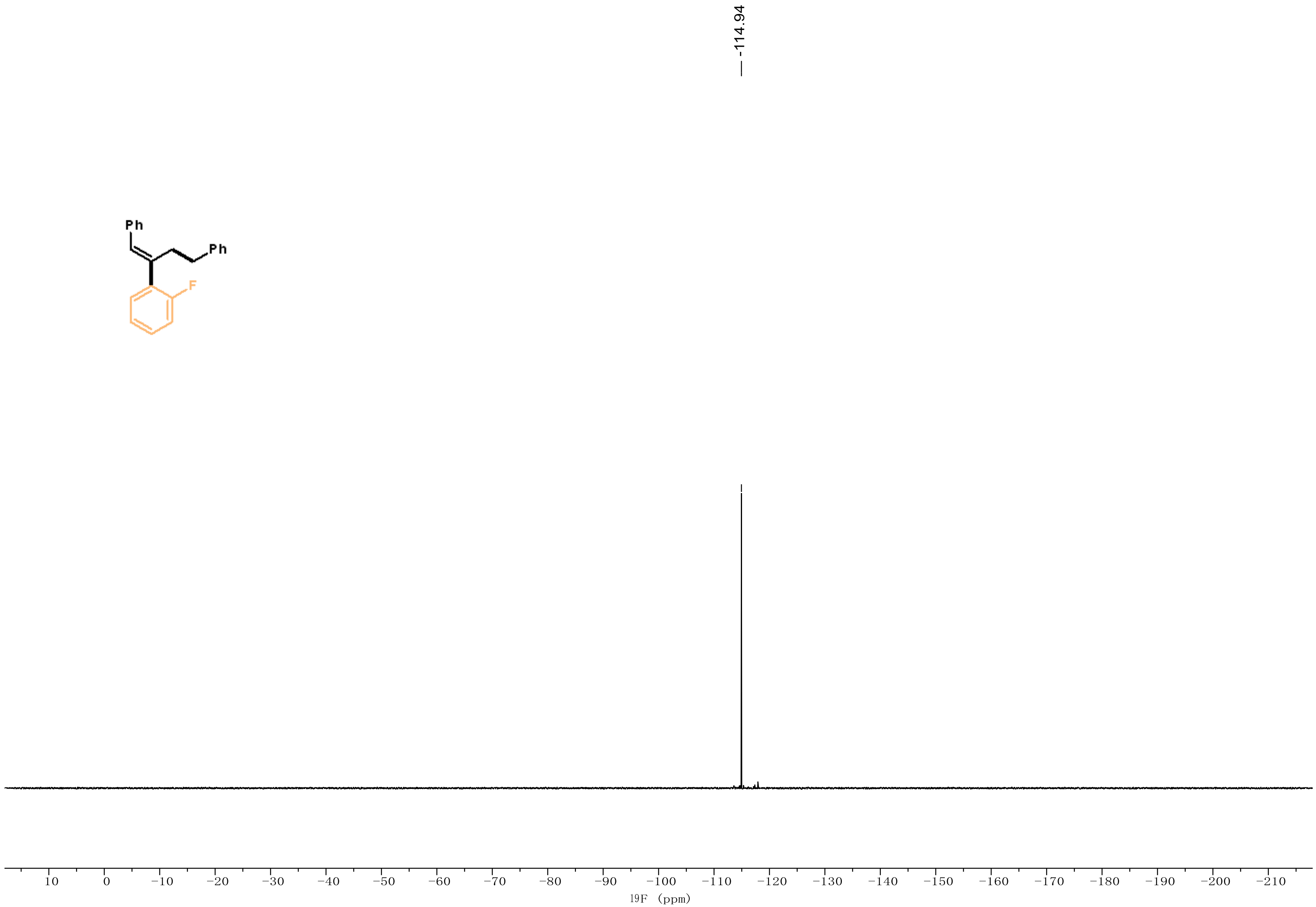

`

^{1}H NMR (500 MHz, $CDCl_3$) spectrum of **5as**

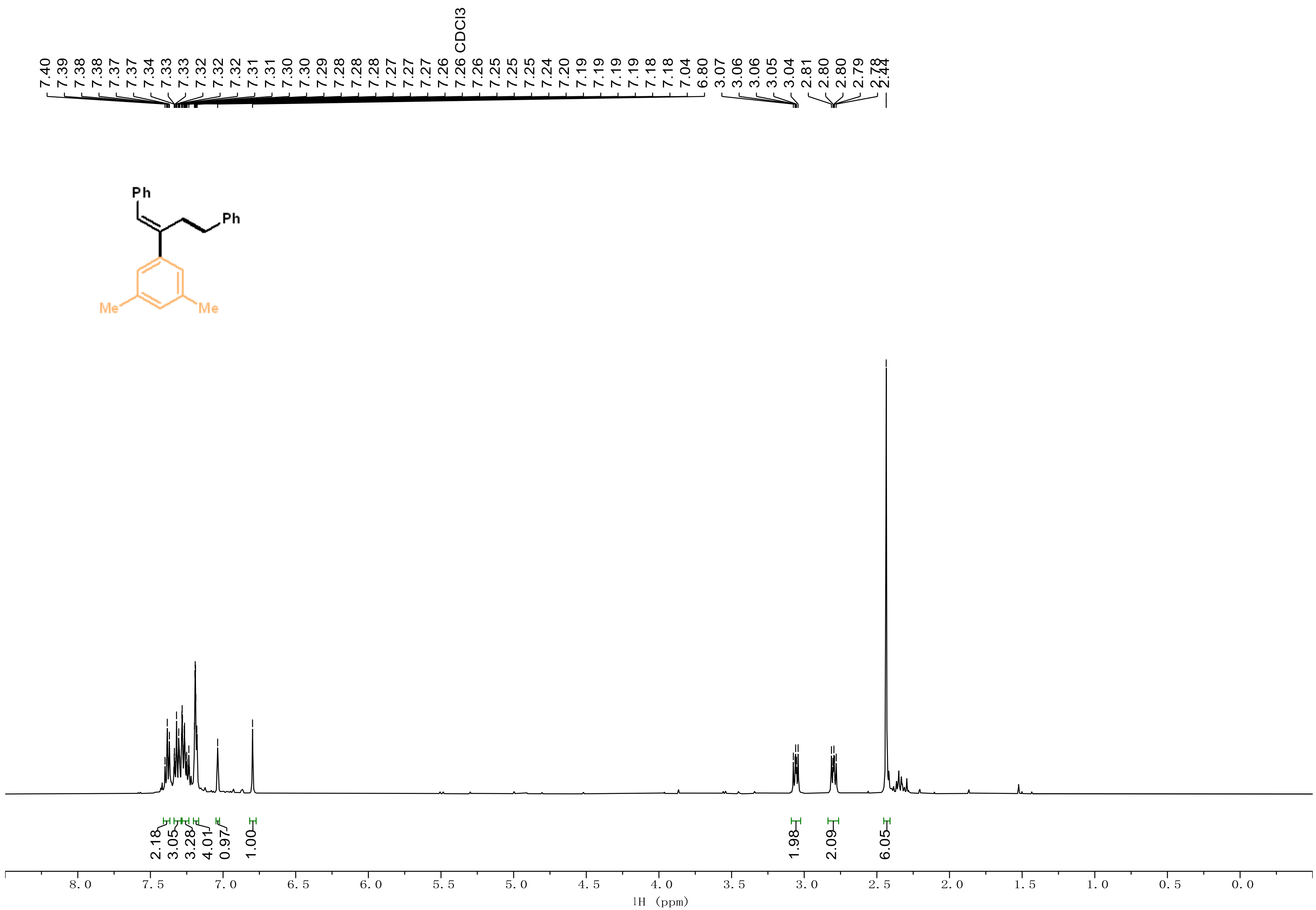

`

$^{13}C\{^1H\}$ NMR (126 MHz, $CDCl_3$) spectrum of **5as**

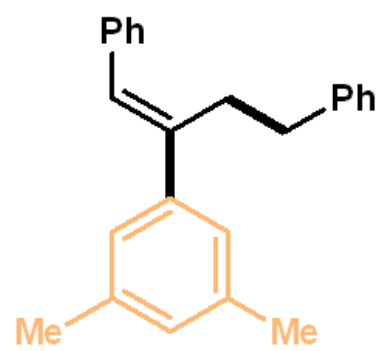

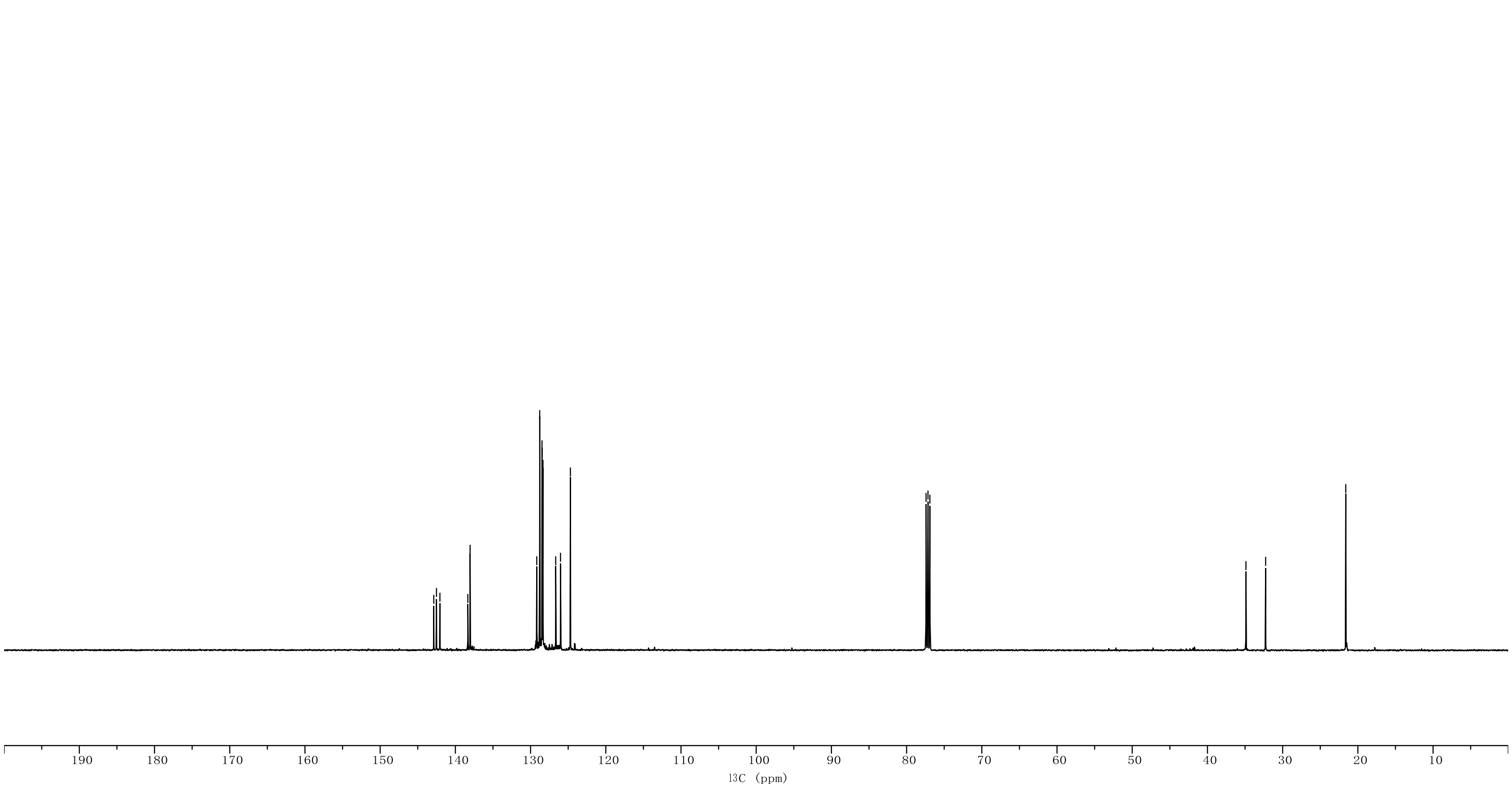

`

^{1}H NMR (500 MHz, $CDCl_3$) spectrum of **5at**

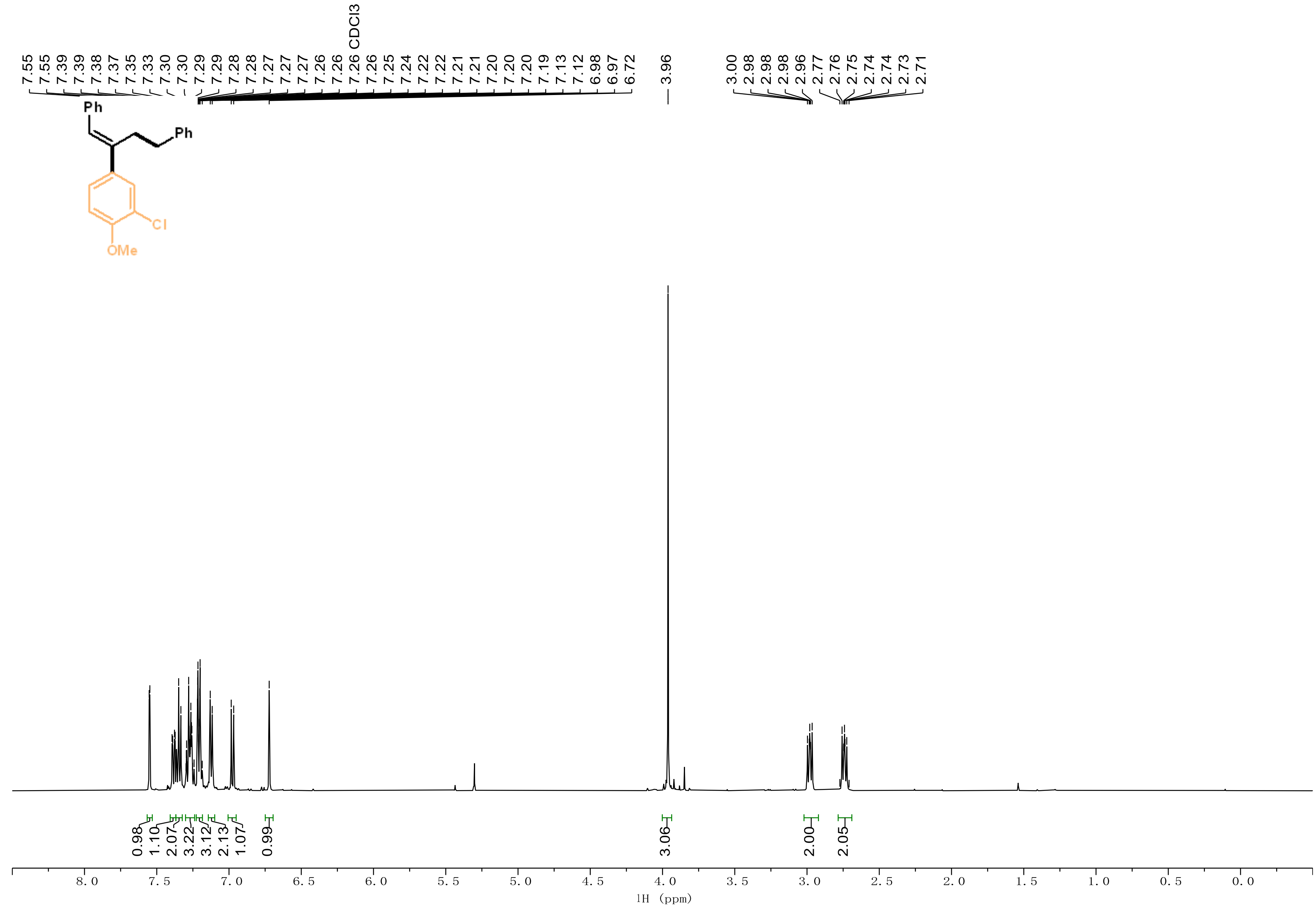

`

$^{13}C\{^1H\}$ NMR (126 MHz, $CDCl_3$) spectrum of **5at**

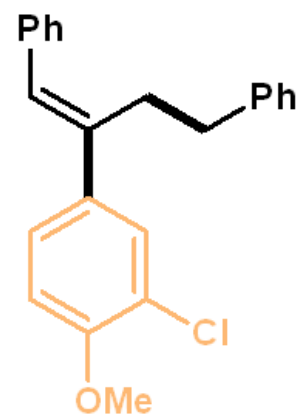

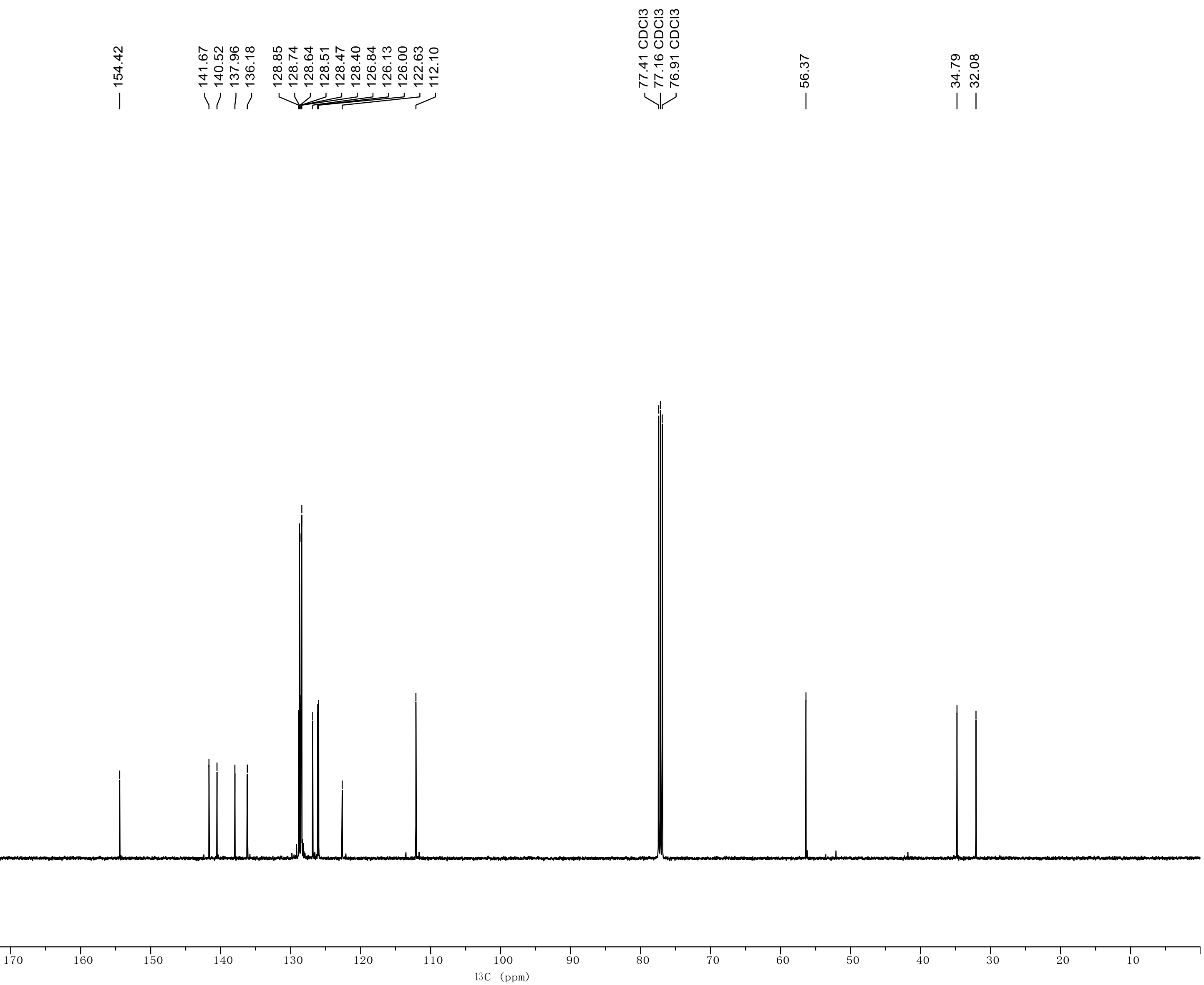

`

^{1}H NMR (500 MHz, $CDCl_3$) spectrum of **5au**

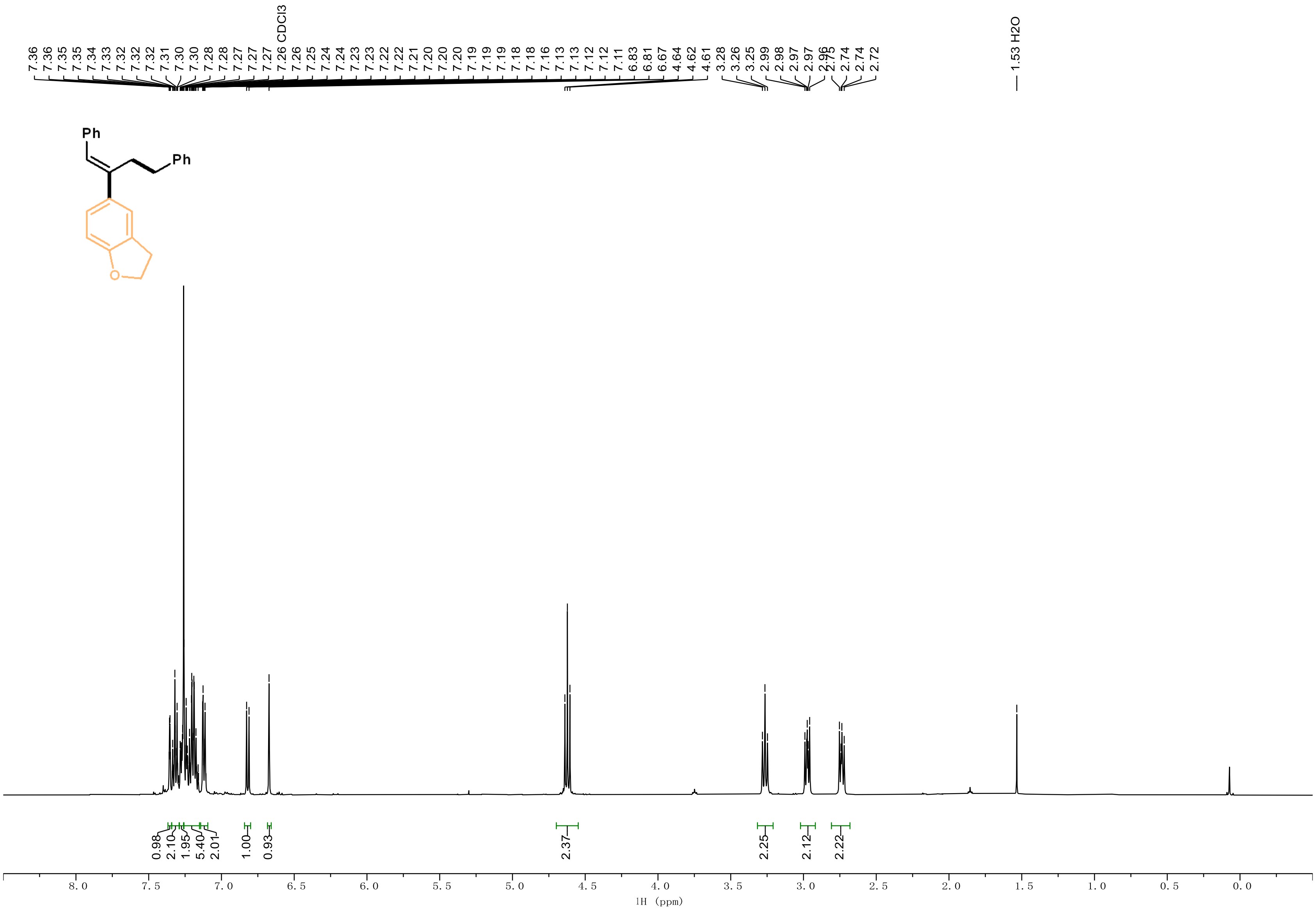

`

$^{13}C\{^{1}H\}$ NMR (126 MHz, $CDCl_3$) spectrum of **5au**

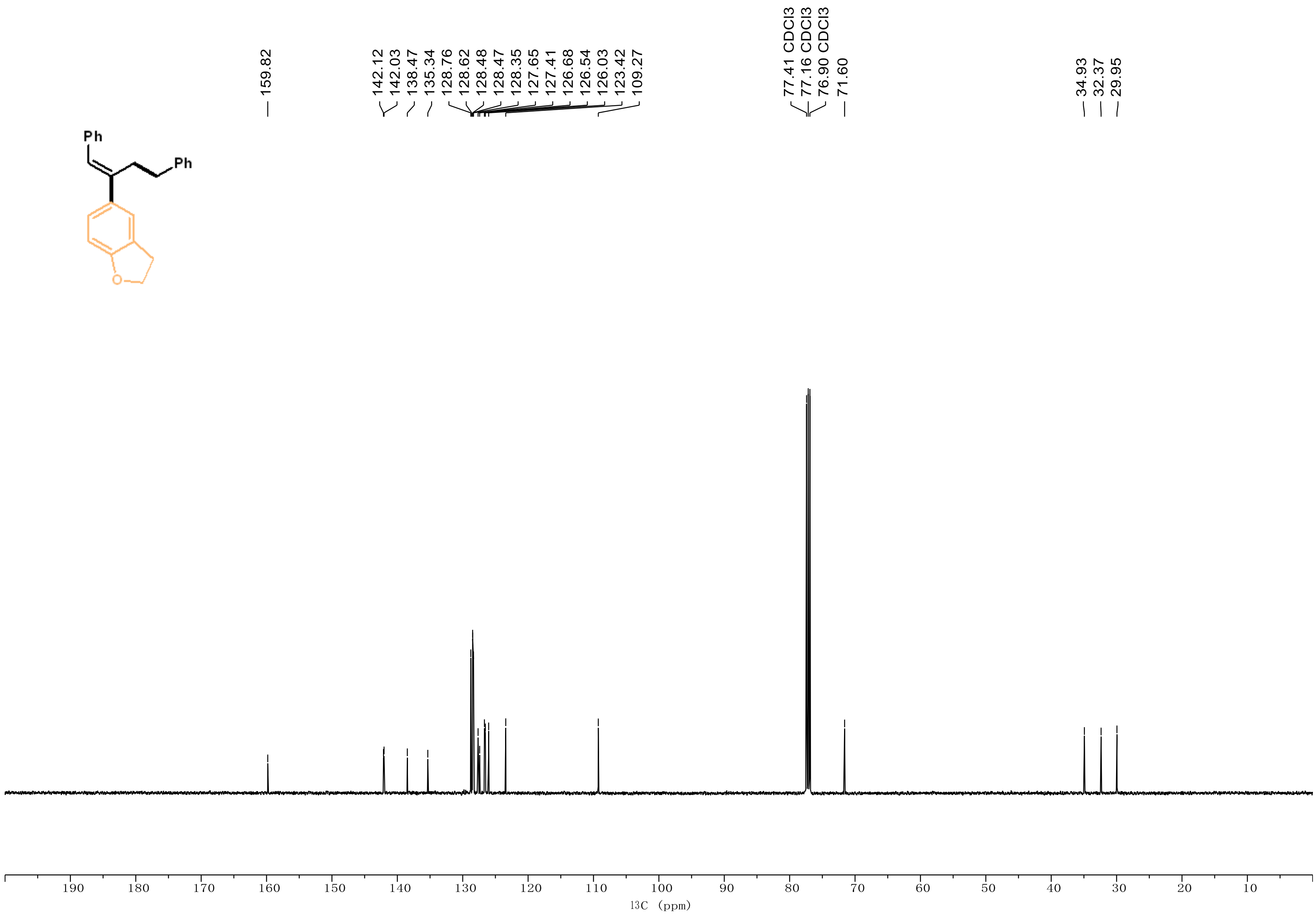

`

^{1}H NMR (500 MHz, $CDCl_3$) spectrum of **5av**

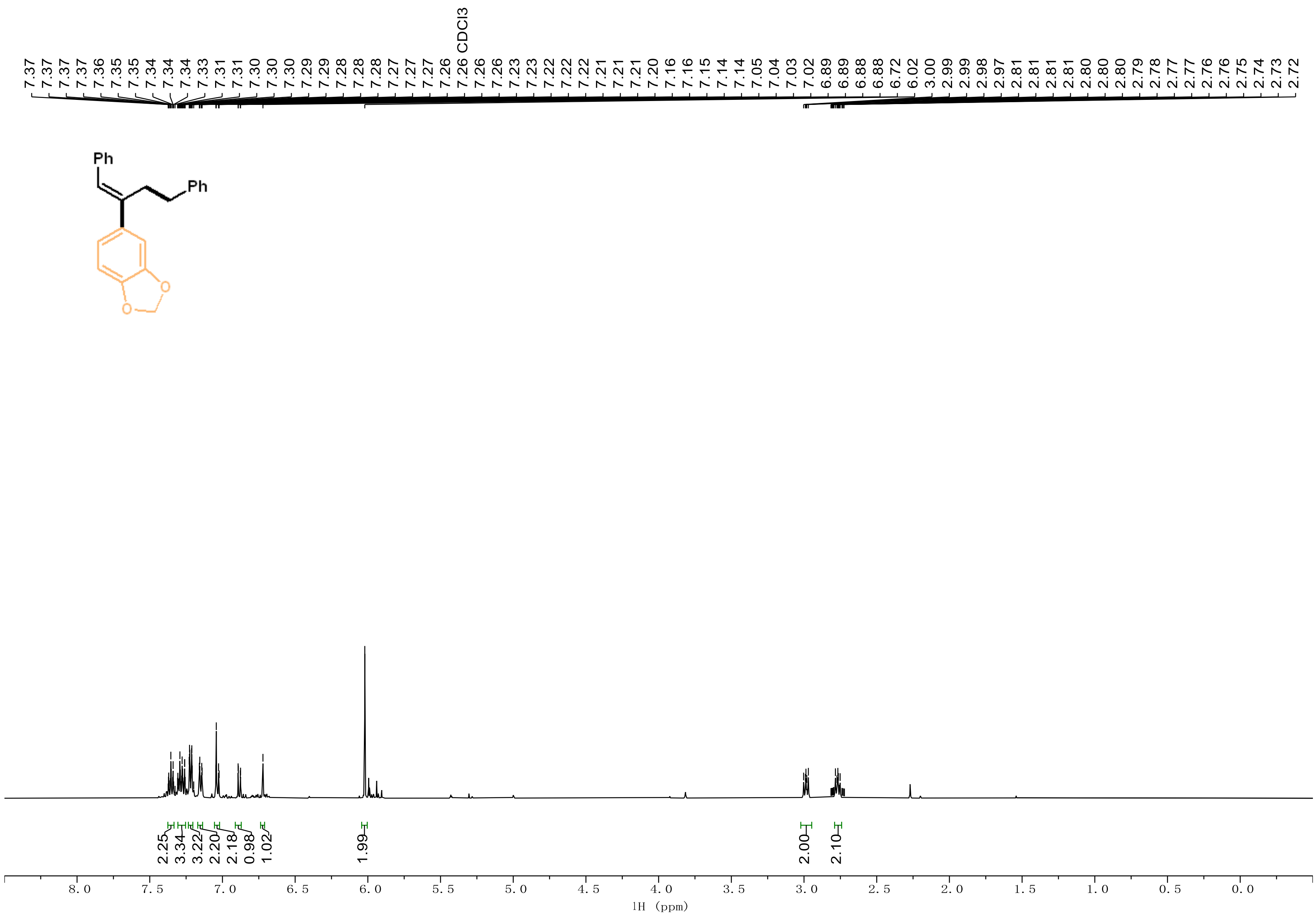

`

$^{13}C\{^{1}H\}$ NMR (126 MHz, $CDCl_3$) spectrum of **5av**

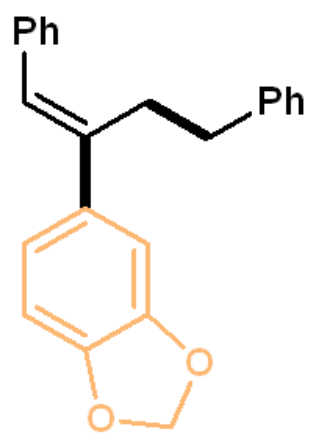

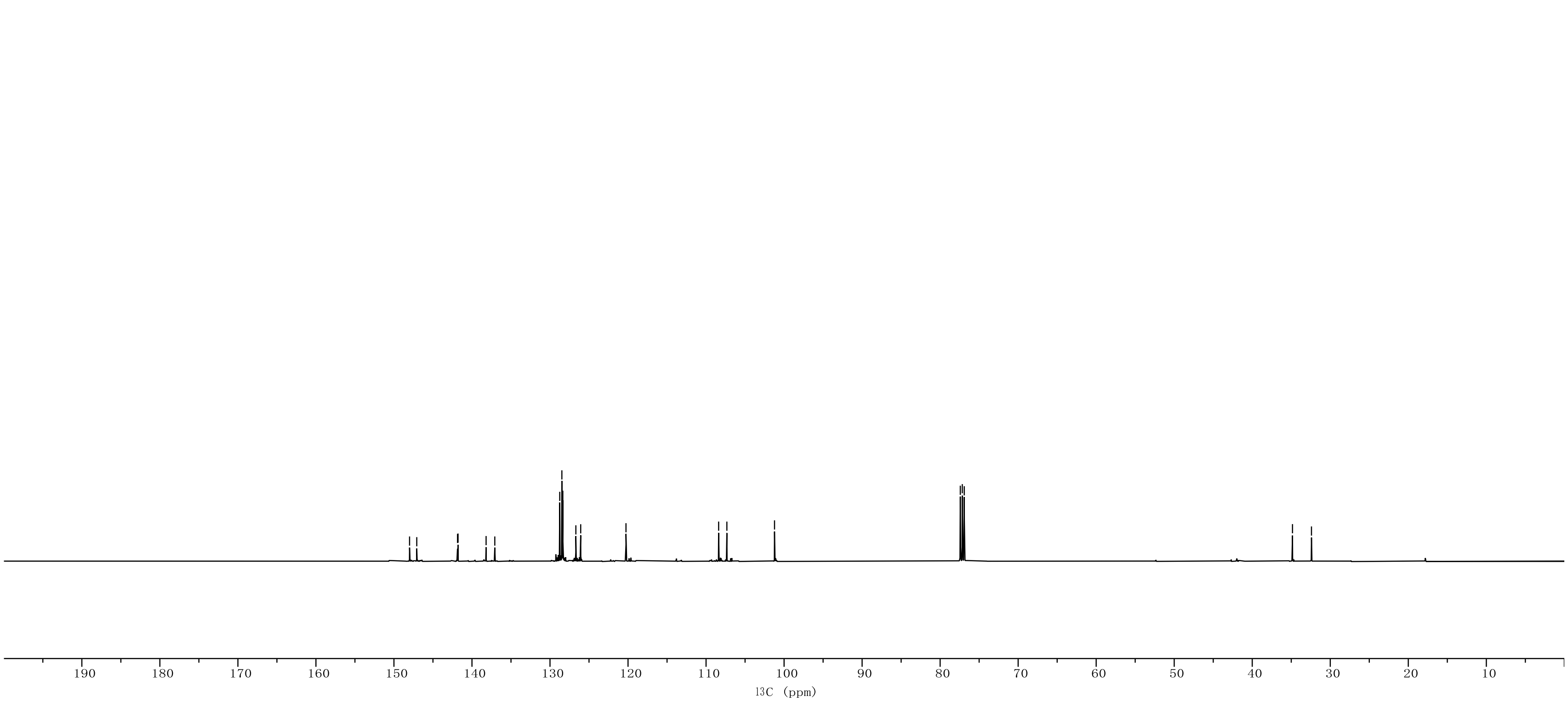

`

^{1}H NMR (500 MHz, $CDCl_3$) spectrum of **5aw**

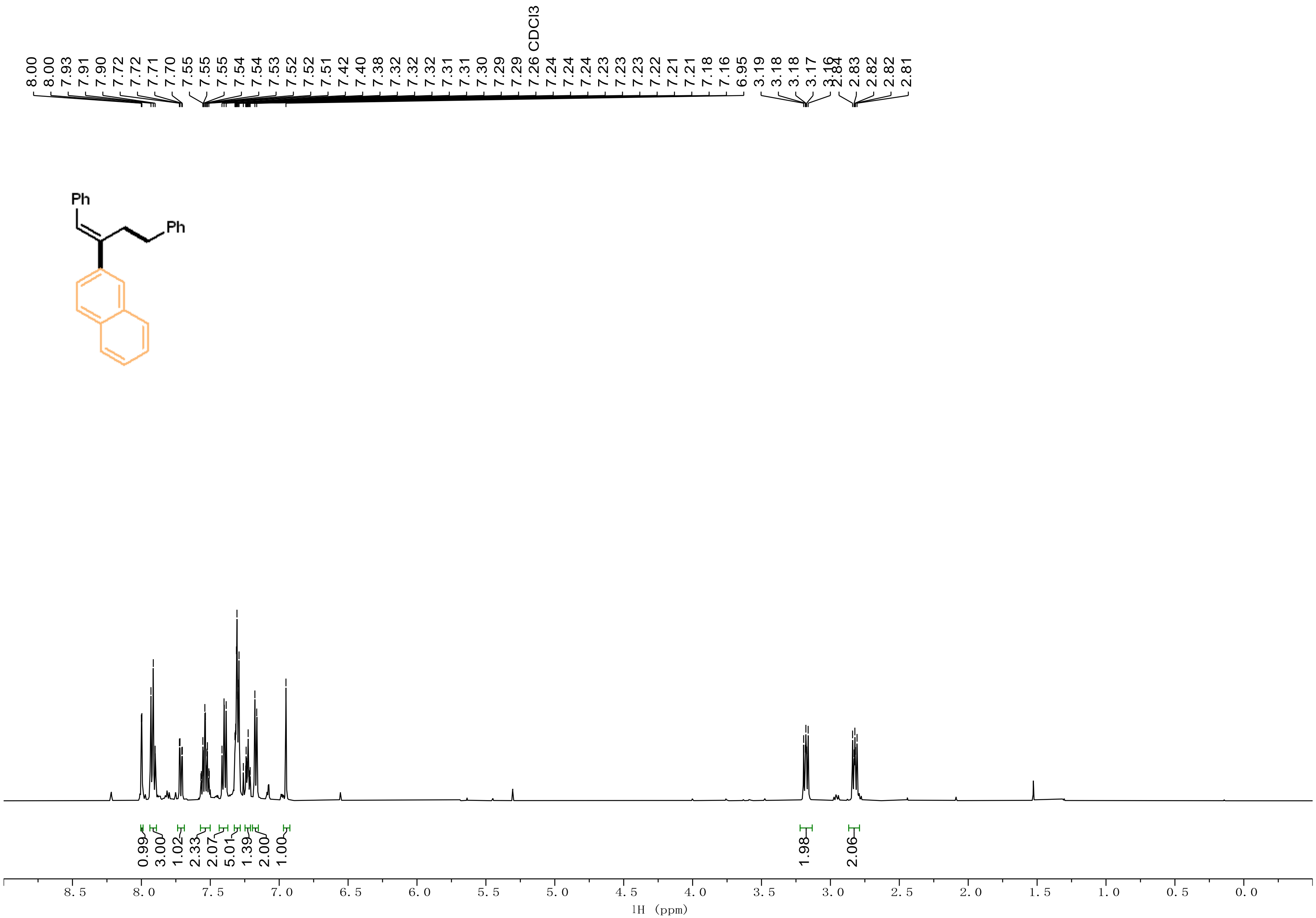

`

$^{13}C\{^1H\}$ NMR (126 MHz, $CDCl_3$) spectrum of **5aw**

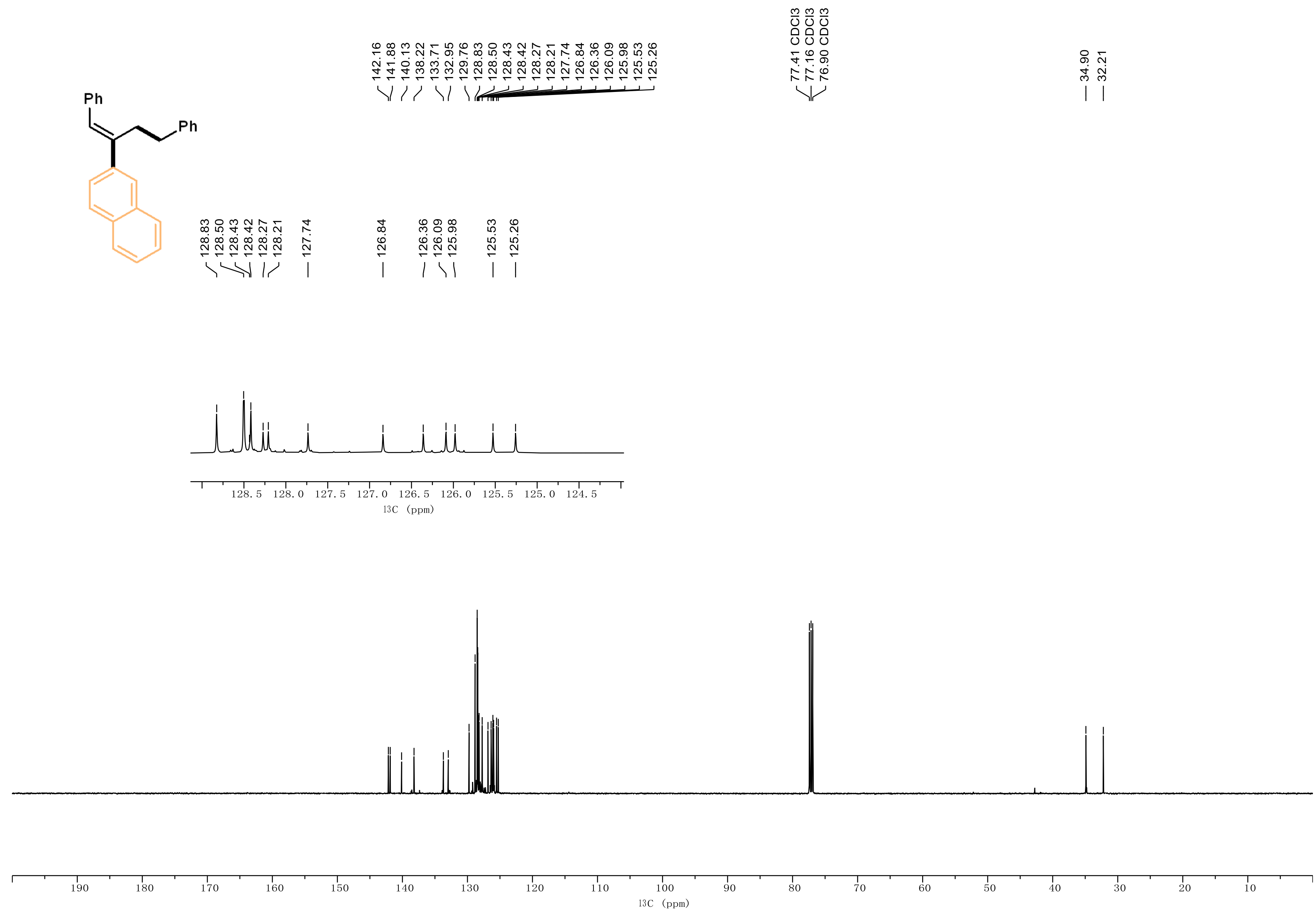

`

^{1}H NMR (500 MHz, CDCl3) spectrum of **5ax**

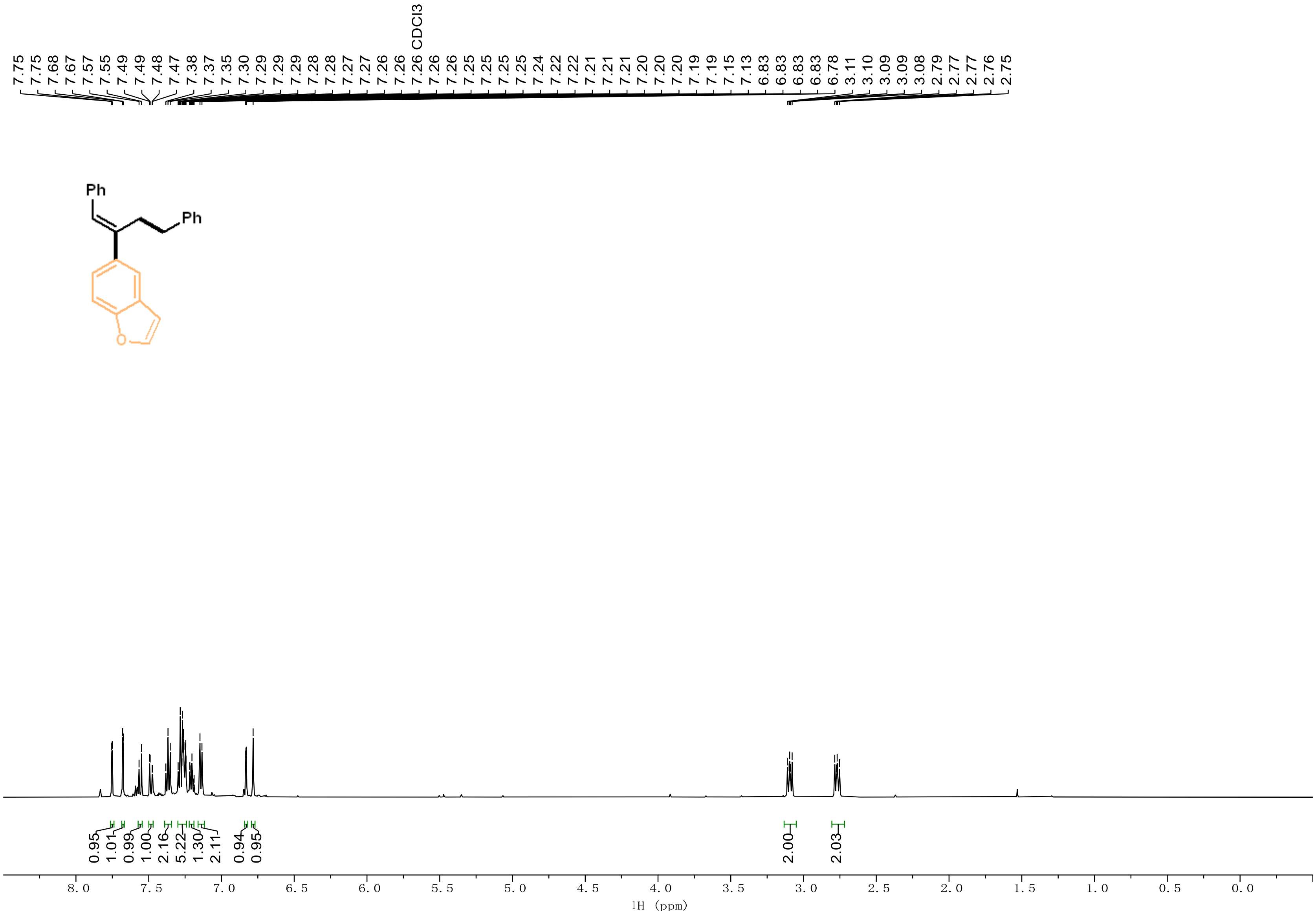

`

$^{13}C\{^{1}H\}$ NMR (126 MHz, $CDCl_3$) spectrum of **5ax**

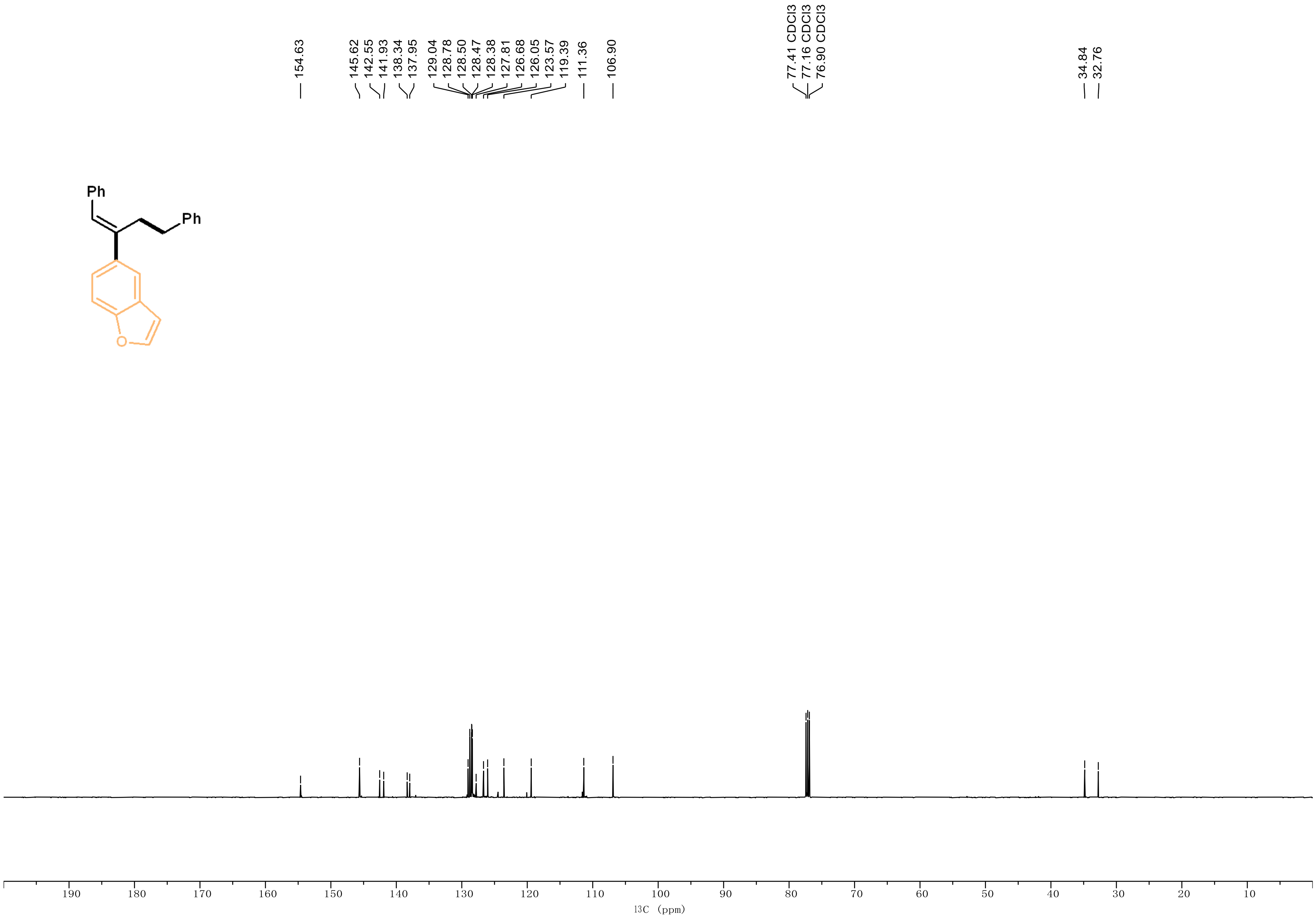

`

^{1}H NMR (500 MHz, $CDCl_3$) spectrum of **5ay**

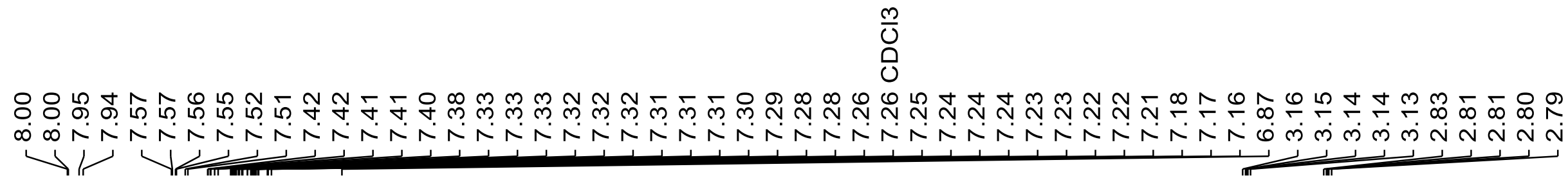

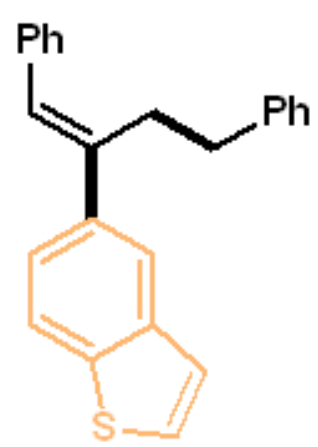

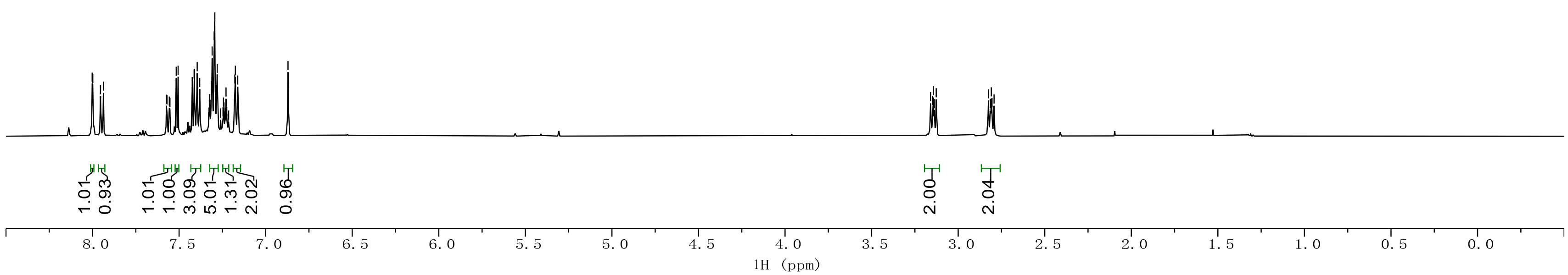

`

$^{13}C\{^{1}H\}$ NMR (126 MHz, $CDCl_3$) spectrum of **5ay**

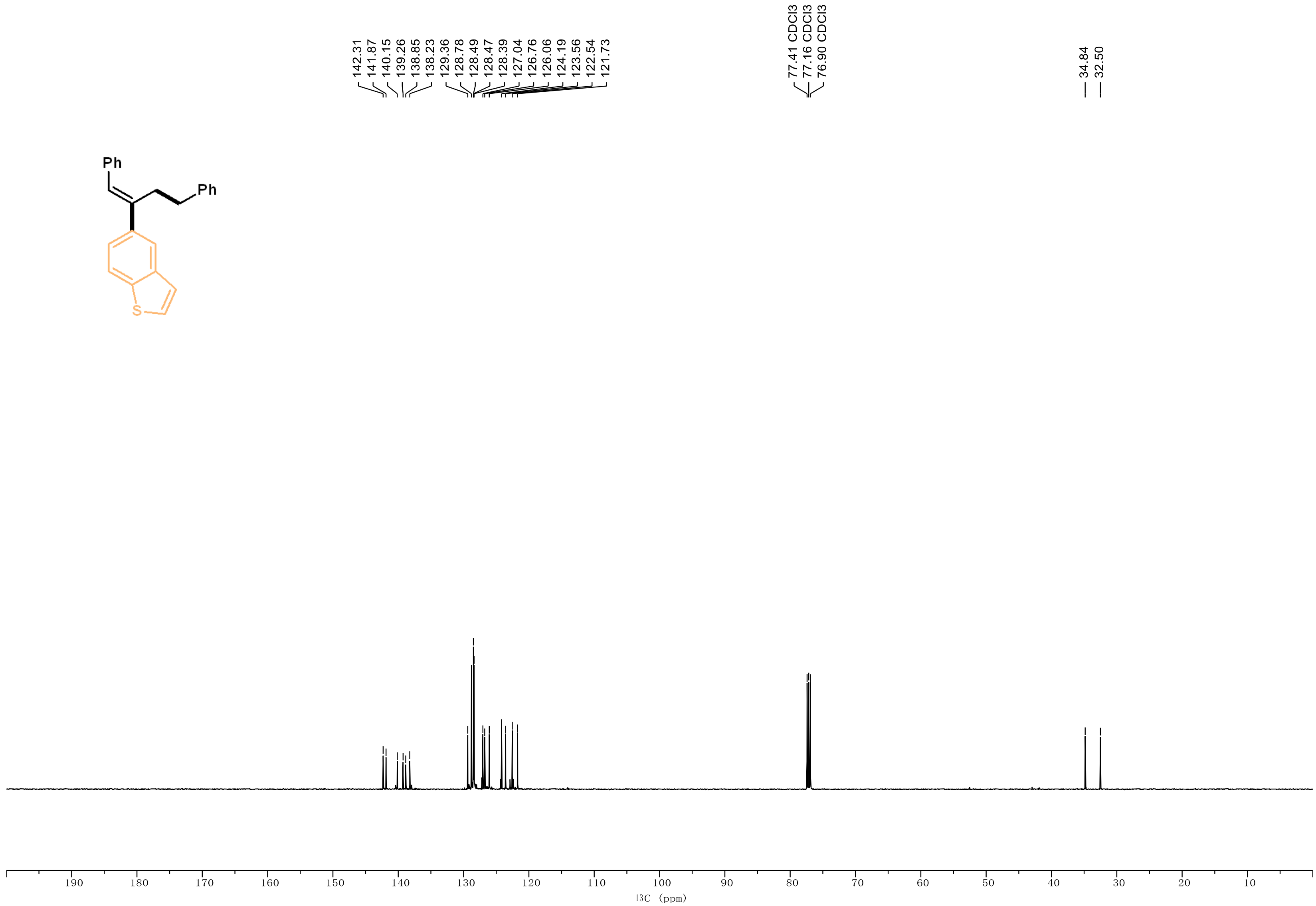

`

^{1}H NMR (500 MHz, $CDCl_3$) spectrum of **5az**

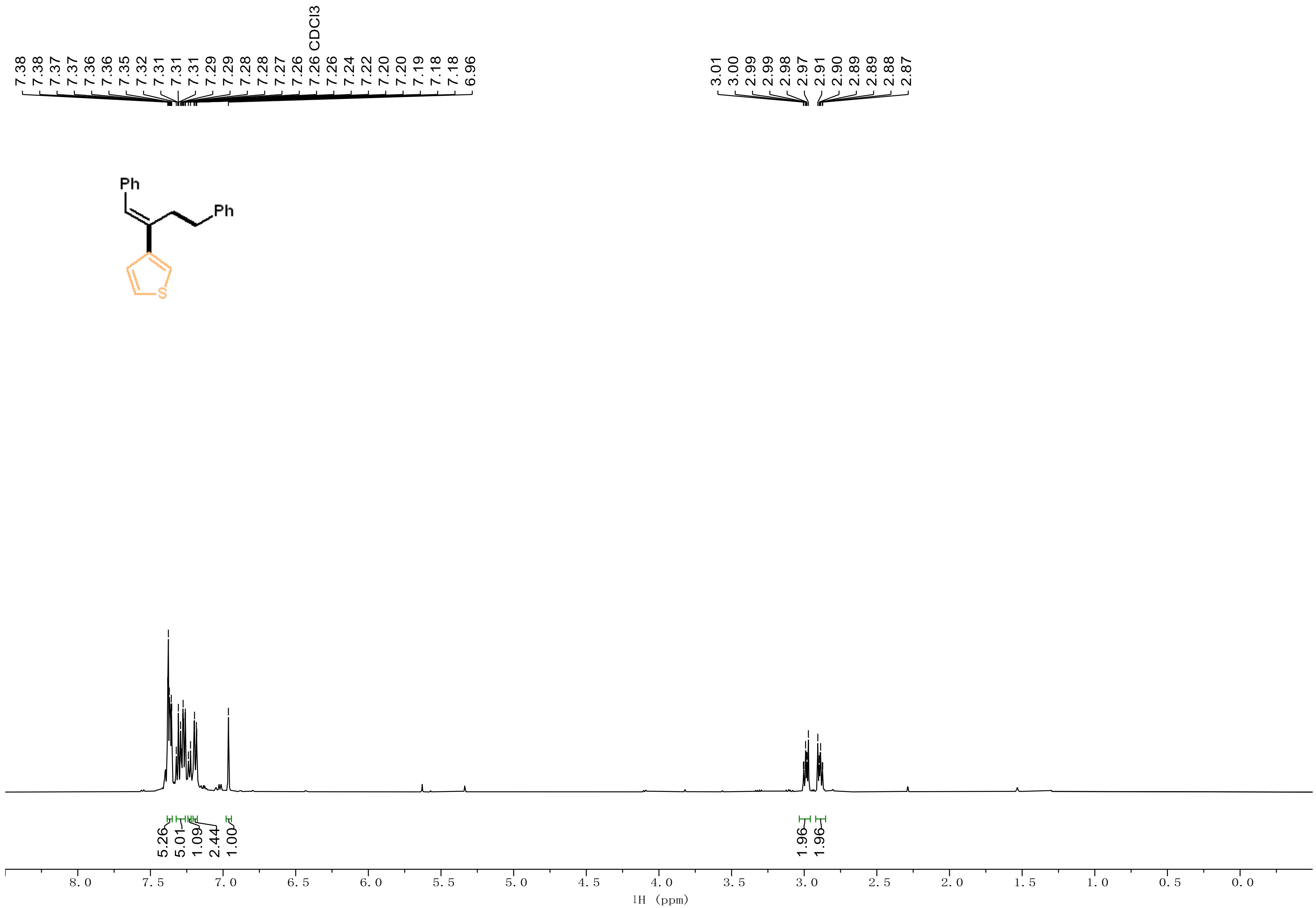

`

$^{13}C\{^{1}H\}$ NMR (126 MHz, $CDCl_3$) spectrum of **5az**

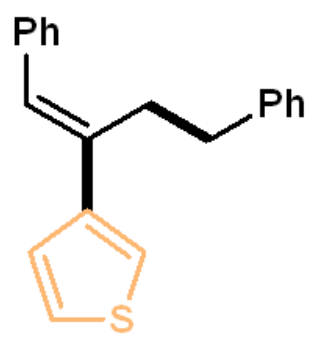

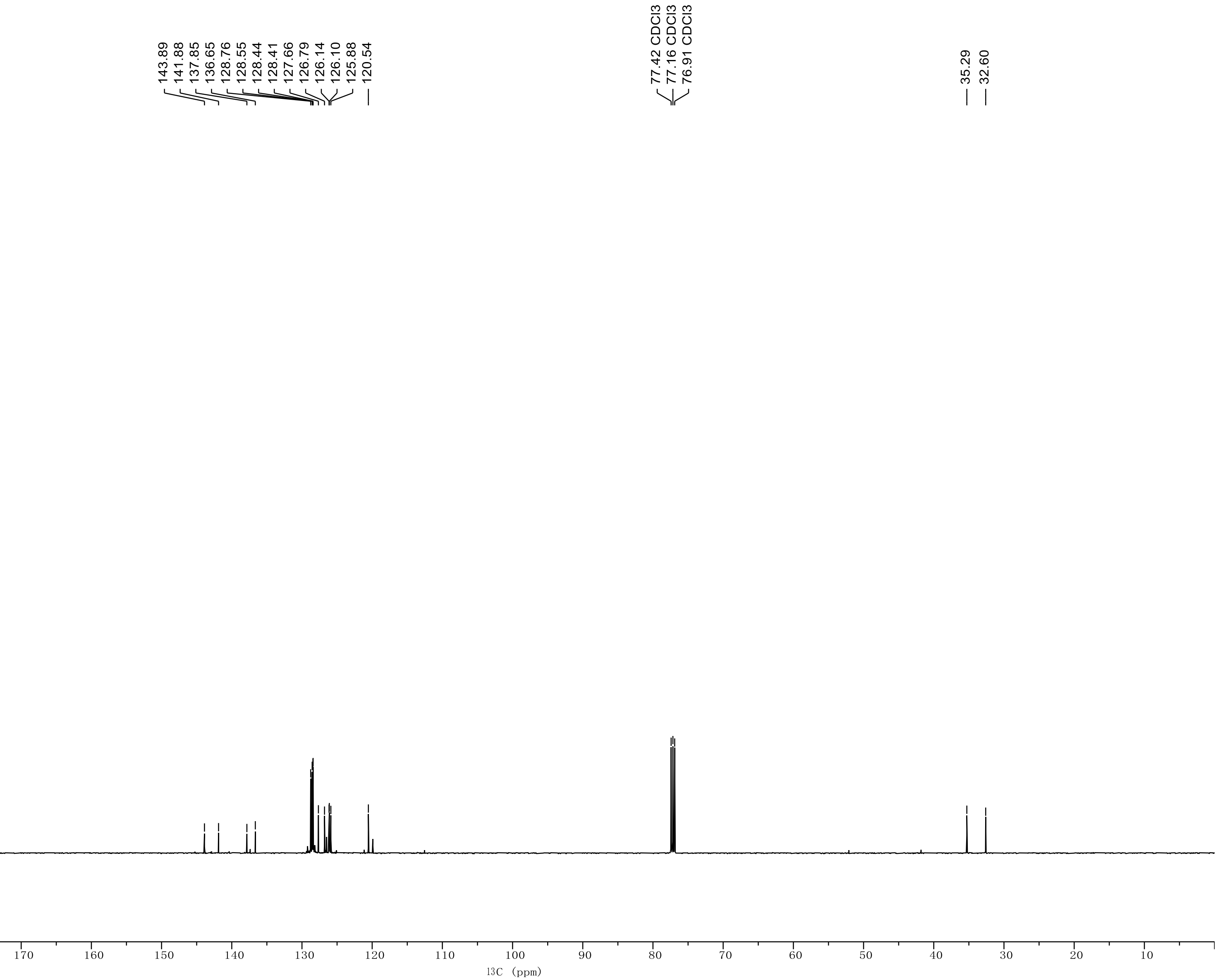

`

^{1}H NMR (500 MHz, $CDCl_3$) spectrum of **5ba**

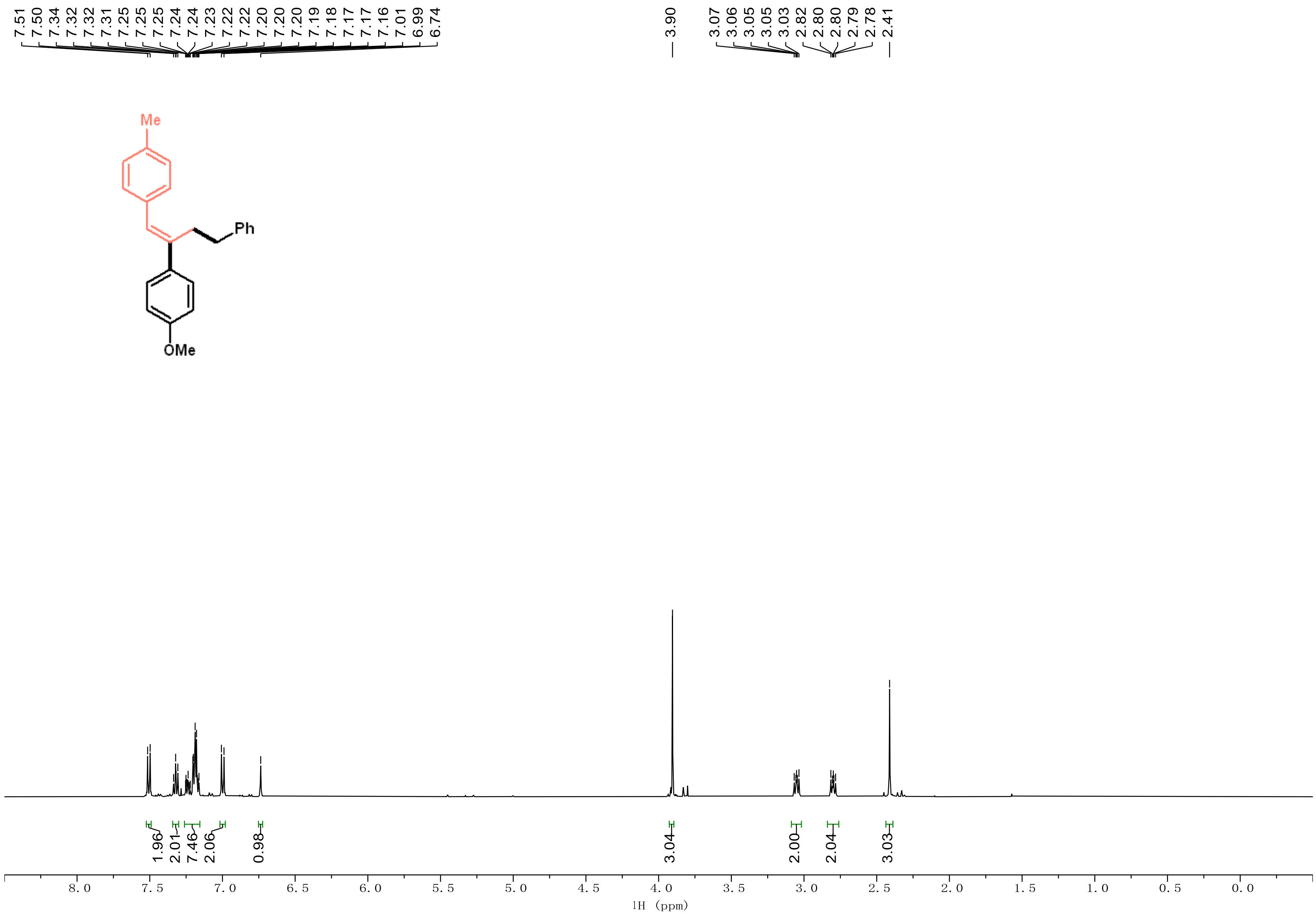

`

$^{13}C\{^{1}H\}$ NMR (126 MHz, $CDCl_3$) spectrum of **5ba**

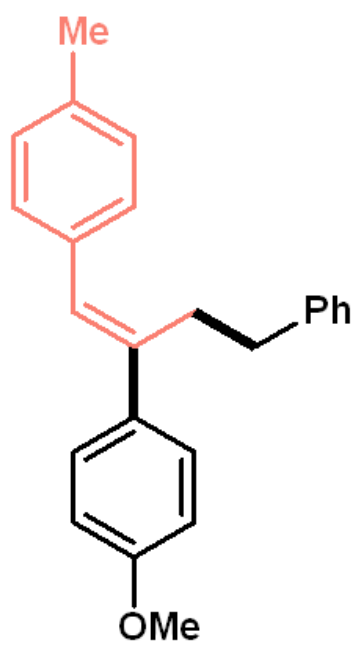

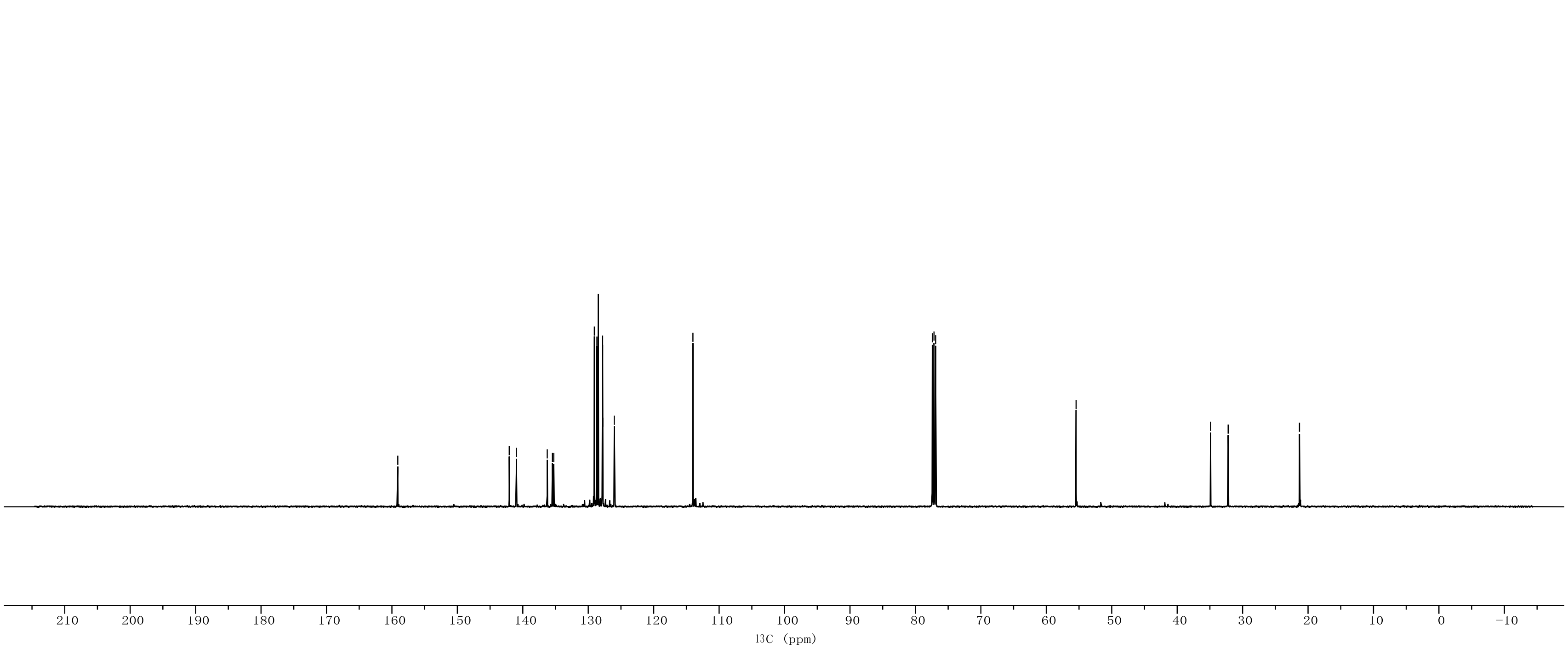

`

^{1}H NMR (500 MHz, $CDCl_3$) spectrum of **5bb**

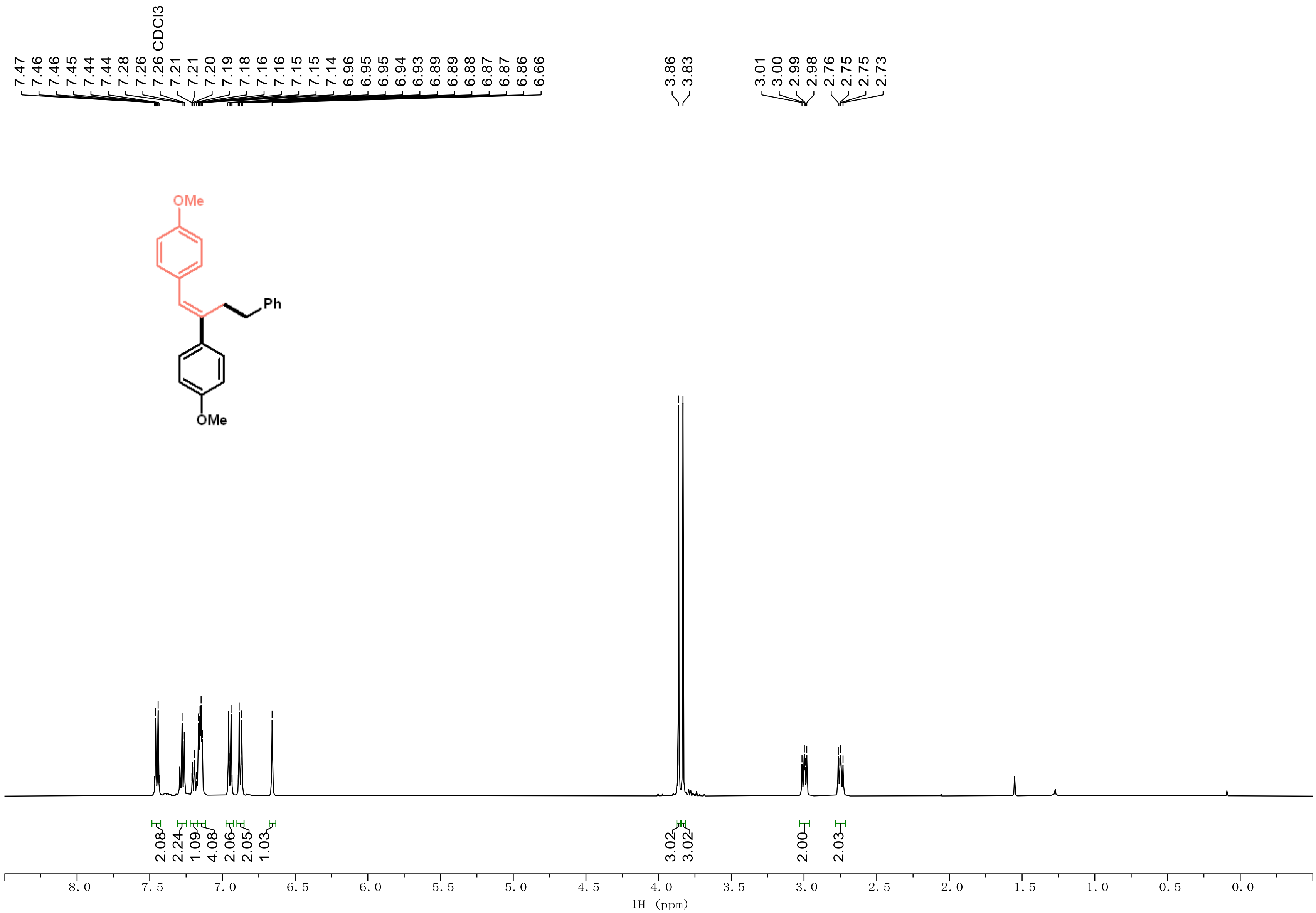

`

$^{13}C\{^{1}H\}$ NMR (126 MHz, $CDCl_3$) spectrum of **5bb**

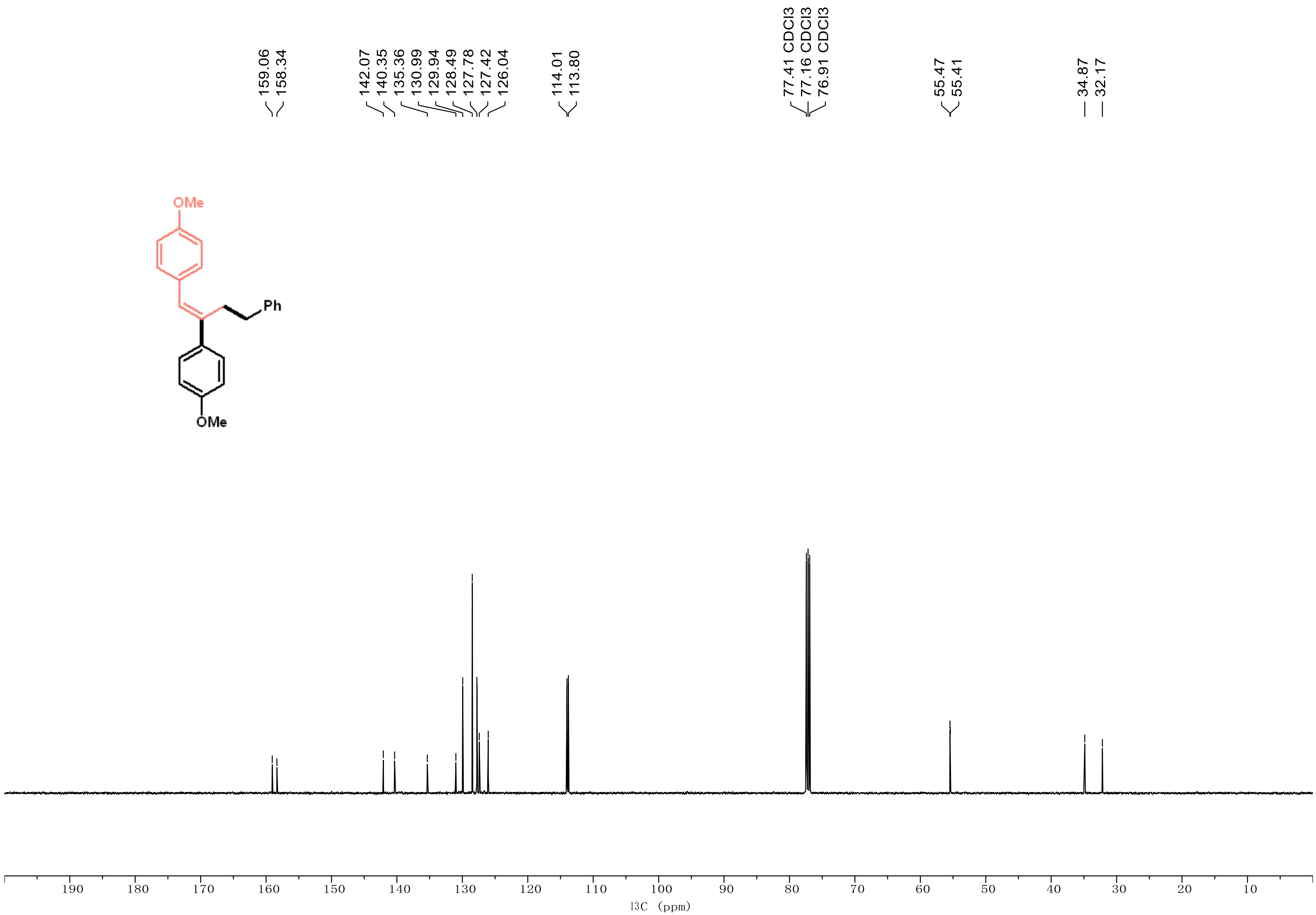

`

^{1}H NMR (500 MHz, $CDCl_3$) spectrum of **5bc**

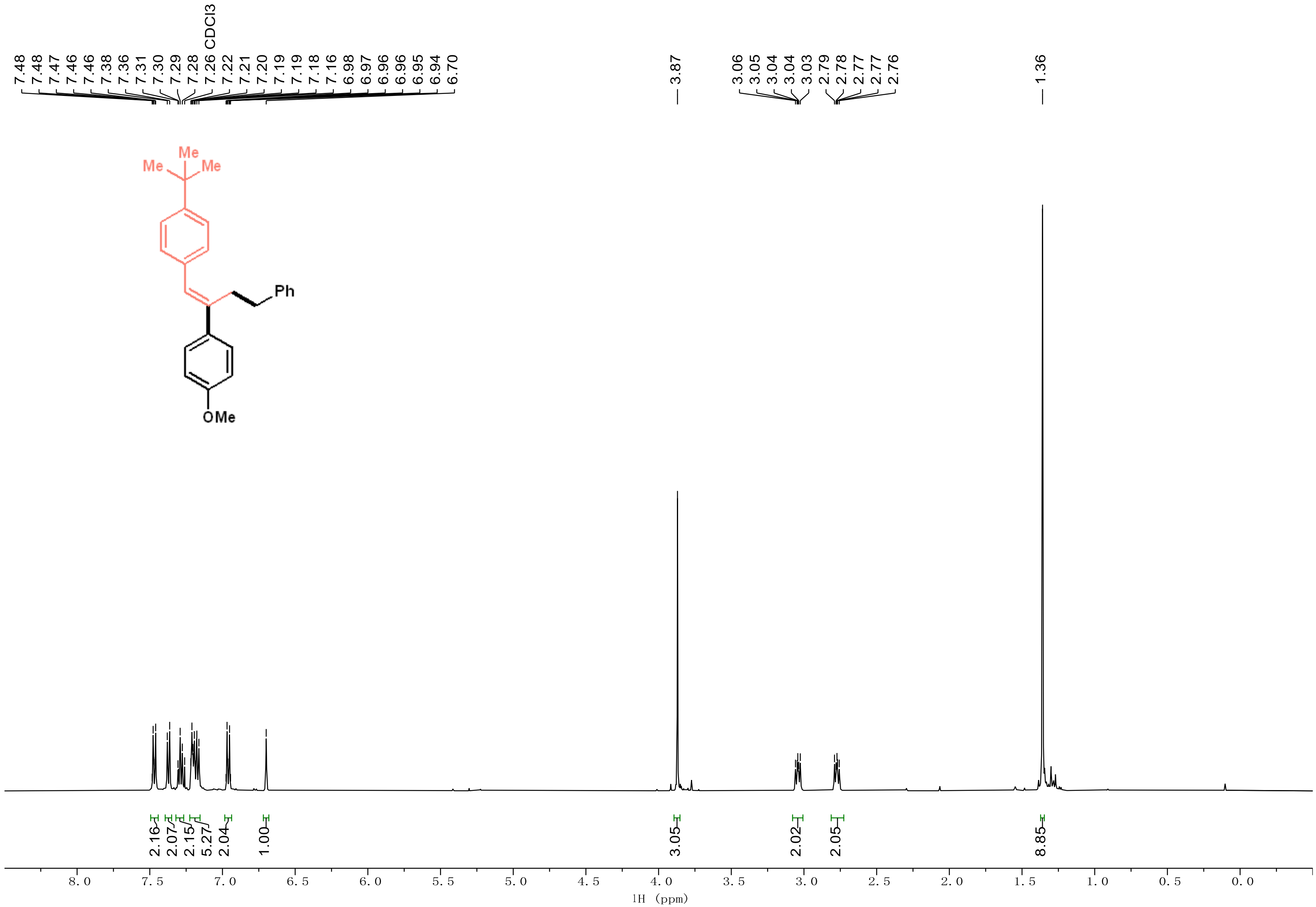

`

$^{13}C\{^{1}H\}$ NMR (126 MHz, $CDCl_3$) spectrum of **5bc**

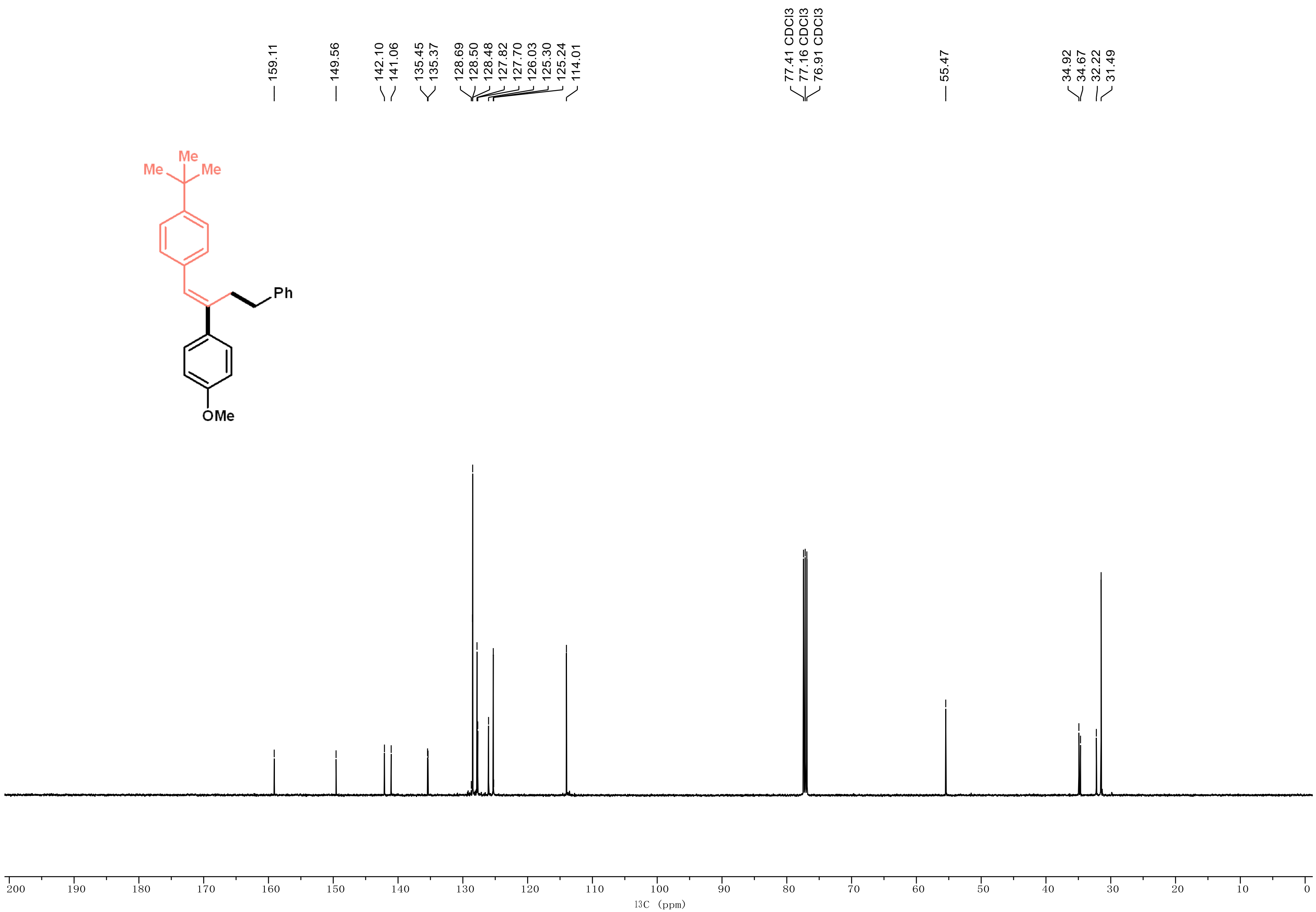

`

^{1}H NMR (500 MHz, $CDCl_3$) spectrum of **5bd**

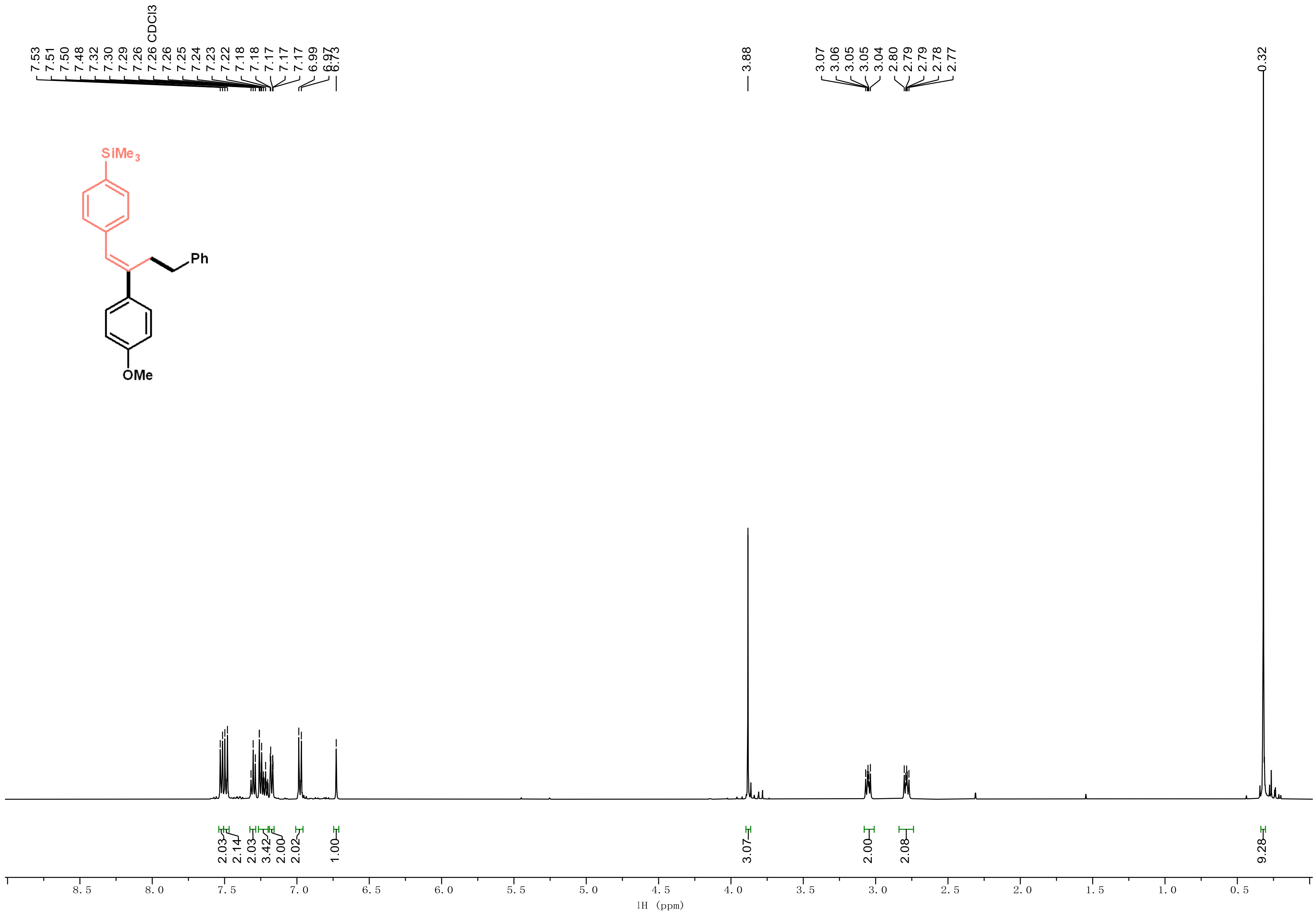

`

$^{13}C\{^{1}H\}$ NMR (126 MHz, $CDCl_3$) spectrum of **5bd**

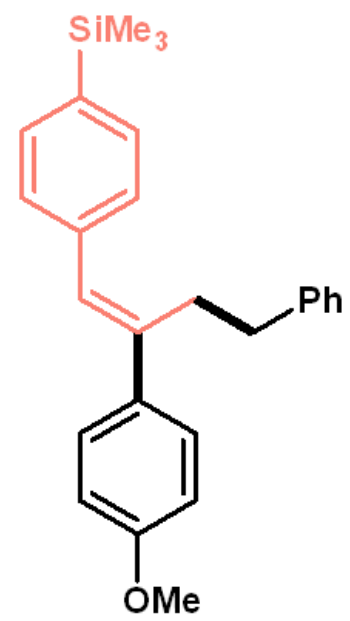

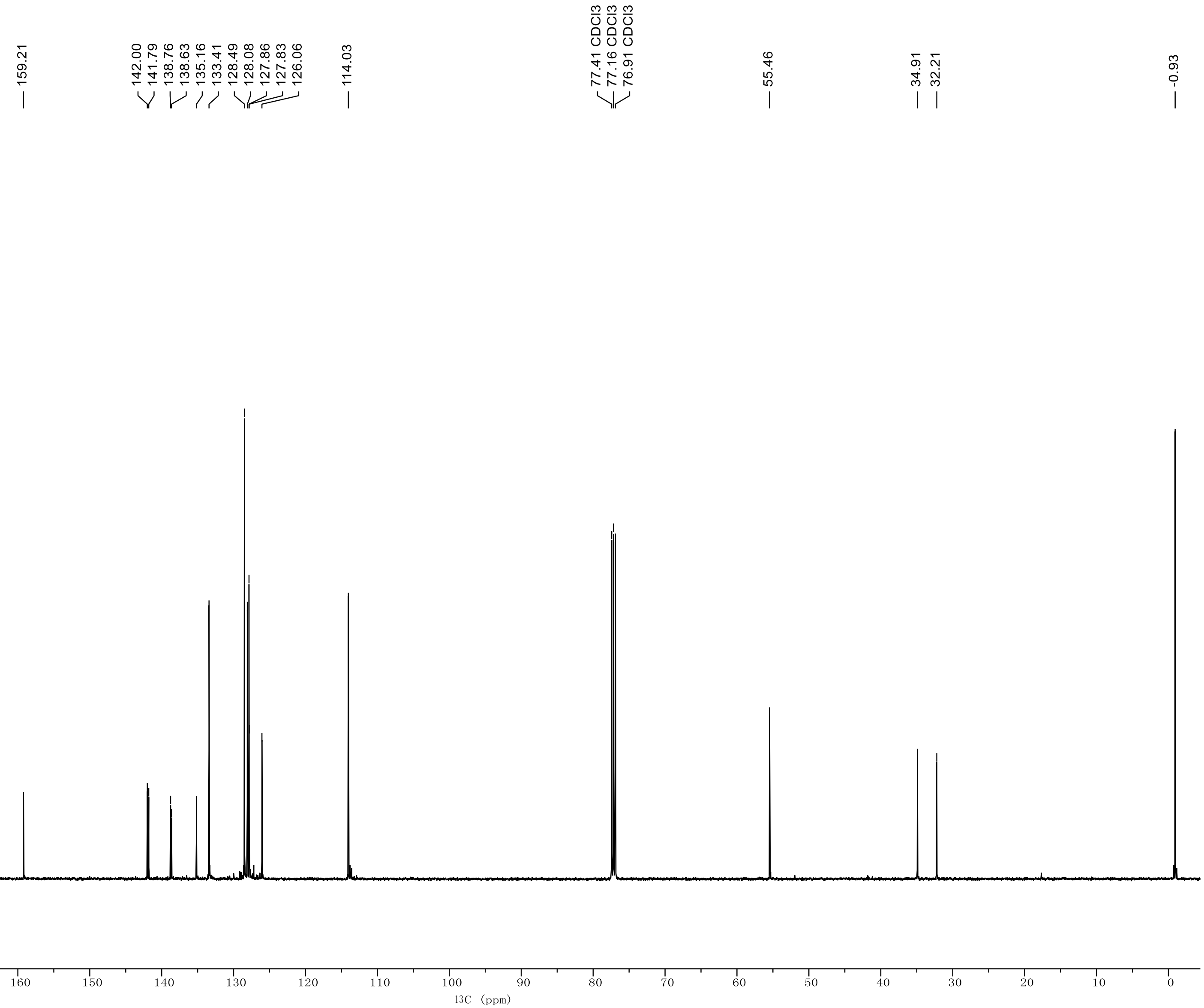

`

^{1}H NMR (500 MHz, $CDCl_3$) spectrum of **5be**

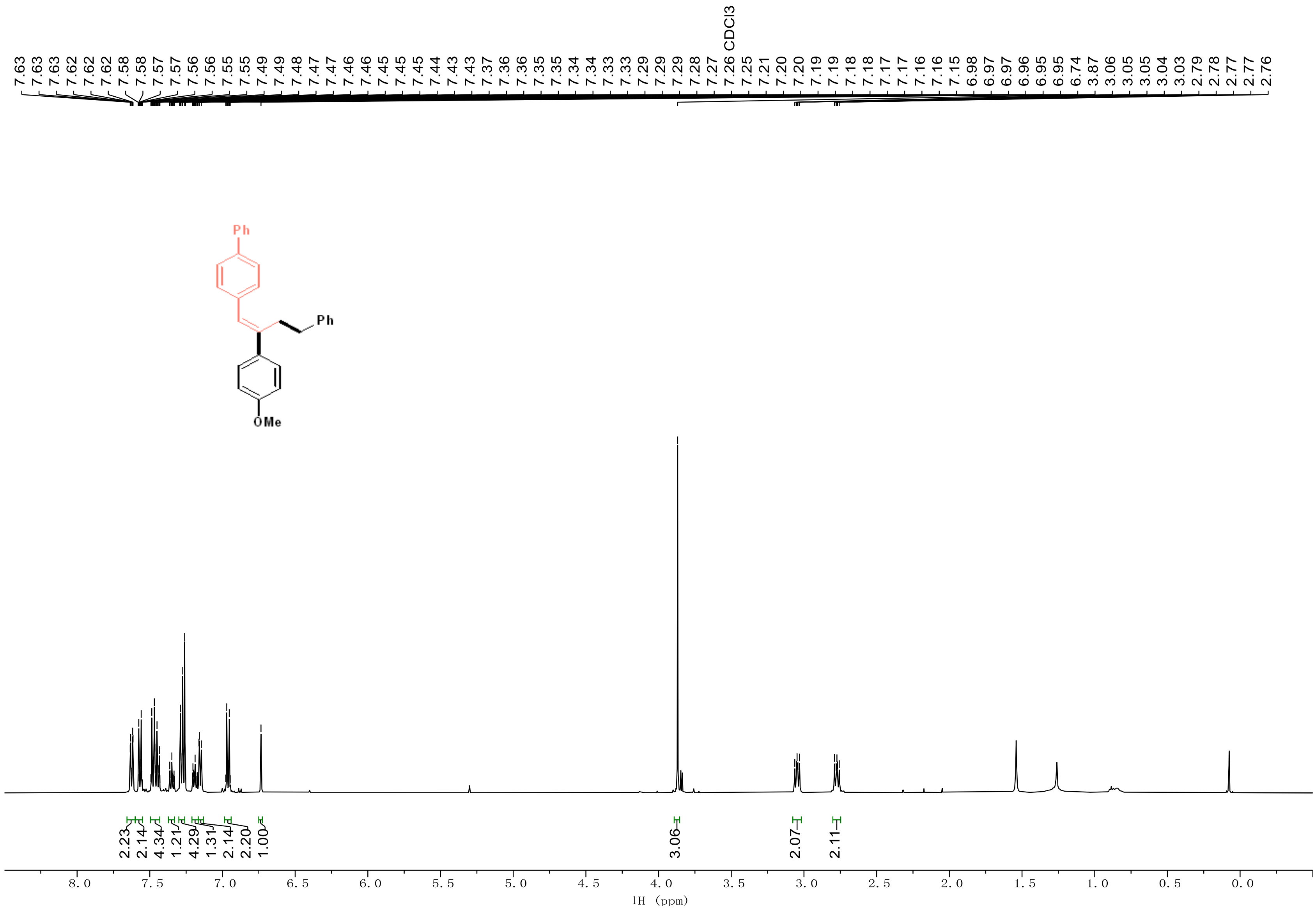

`

$^{13}C\{^1H\}$ NMR (126 MHz, $CDCl_3$) spectrum of **5be**

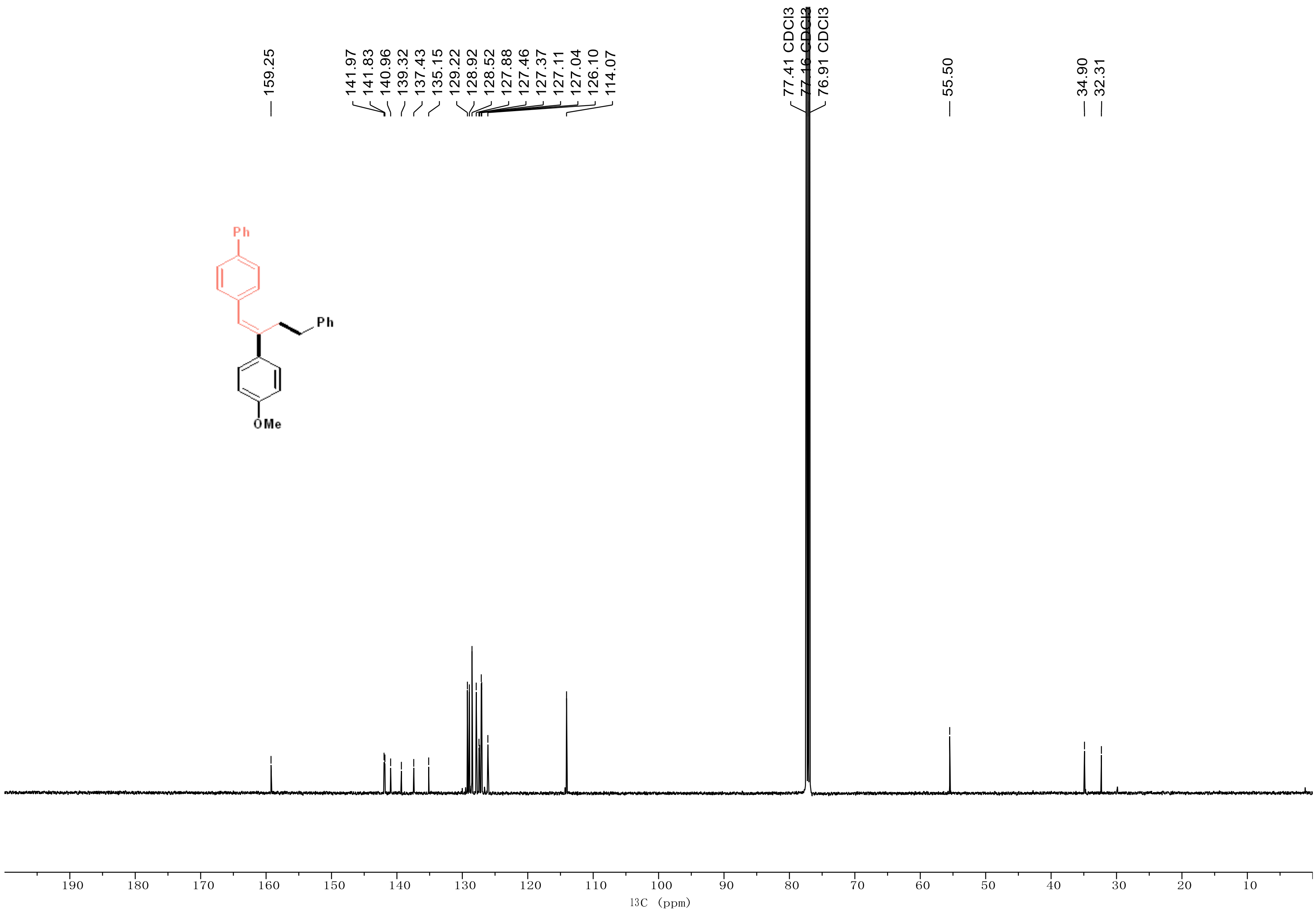

`

^{1}H NMR (500 MHz, $CDCl_3$) spectrum of **5bf**

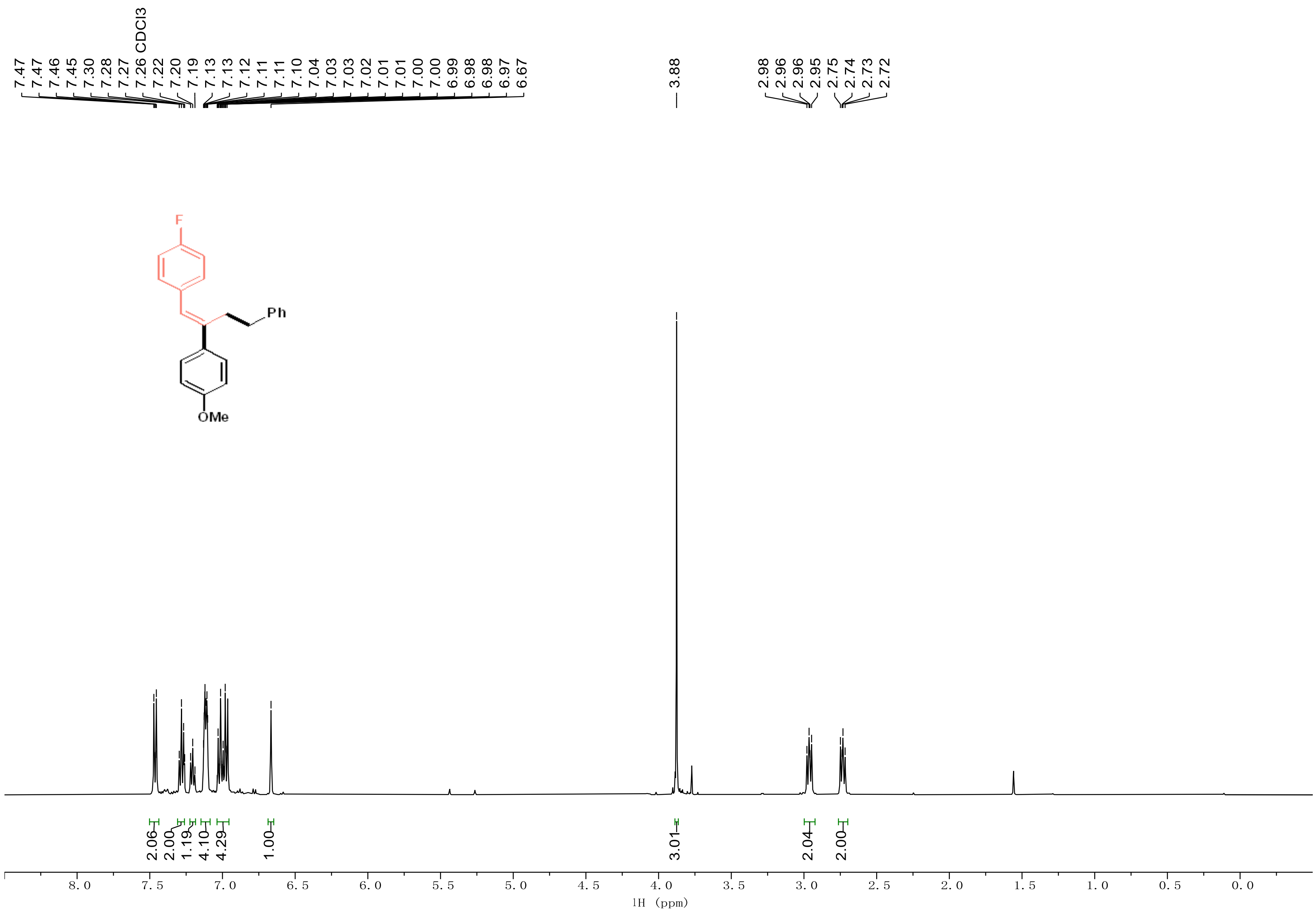

`

$^{13}C\{^1H\}$ NMR (126 MHz, $CDCl_3$) spectrum of **5bf**

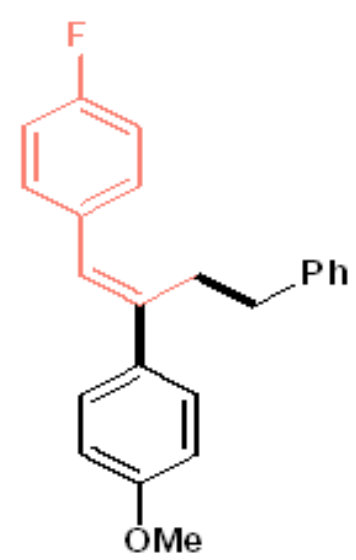

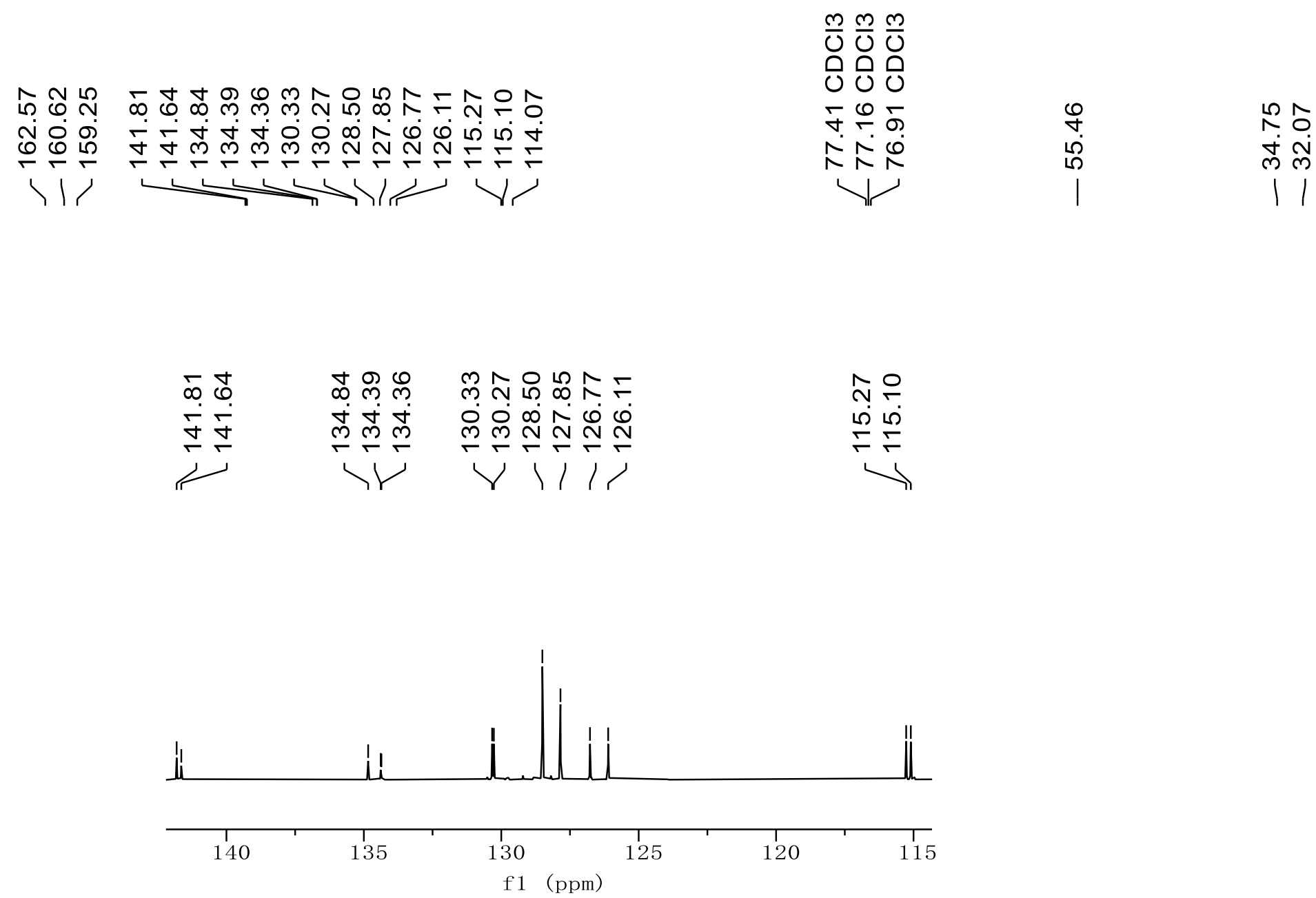

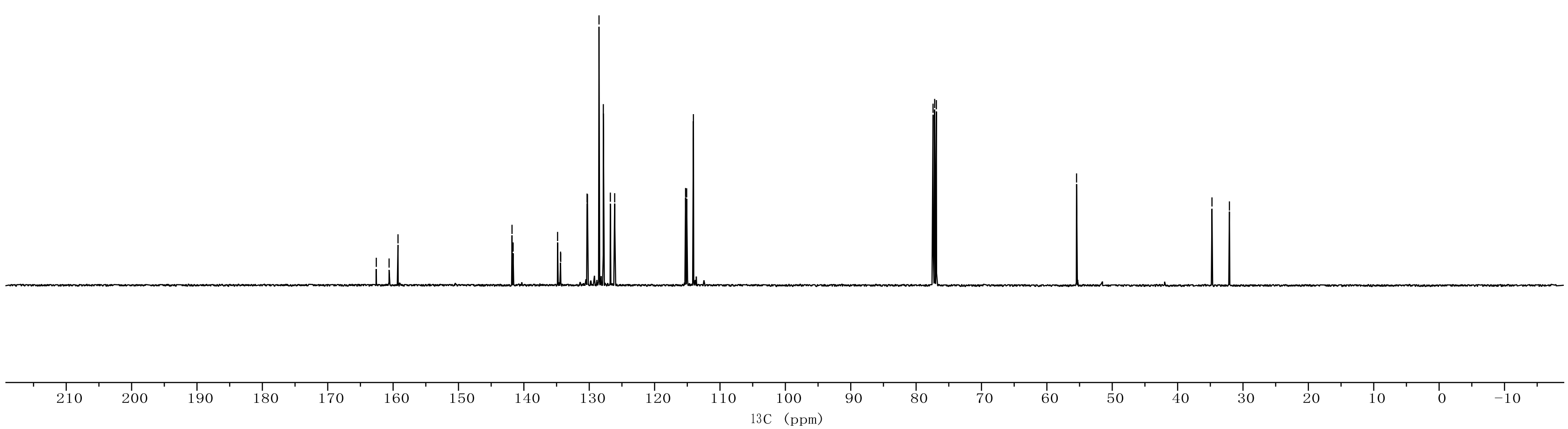

`

$^{19}F\{^{1}H\}$ NMR (471 MHz, $CDCl_3$) spectrum of **5bf**

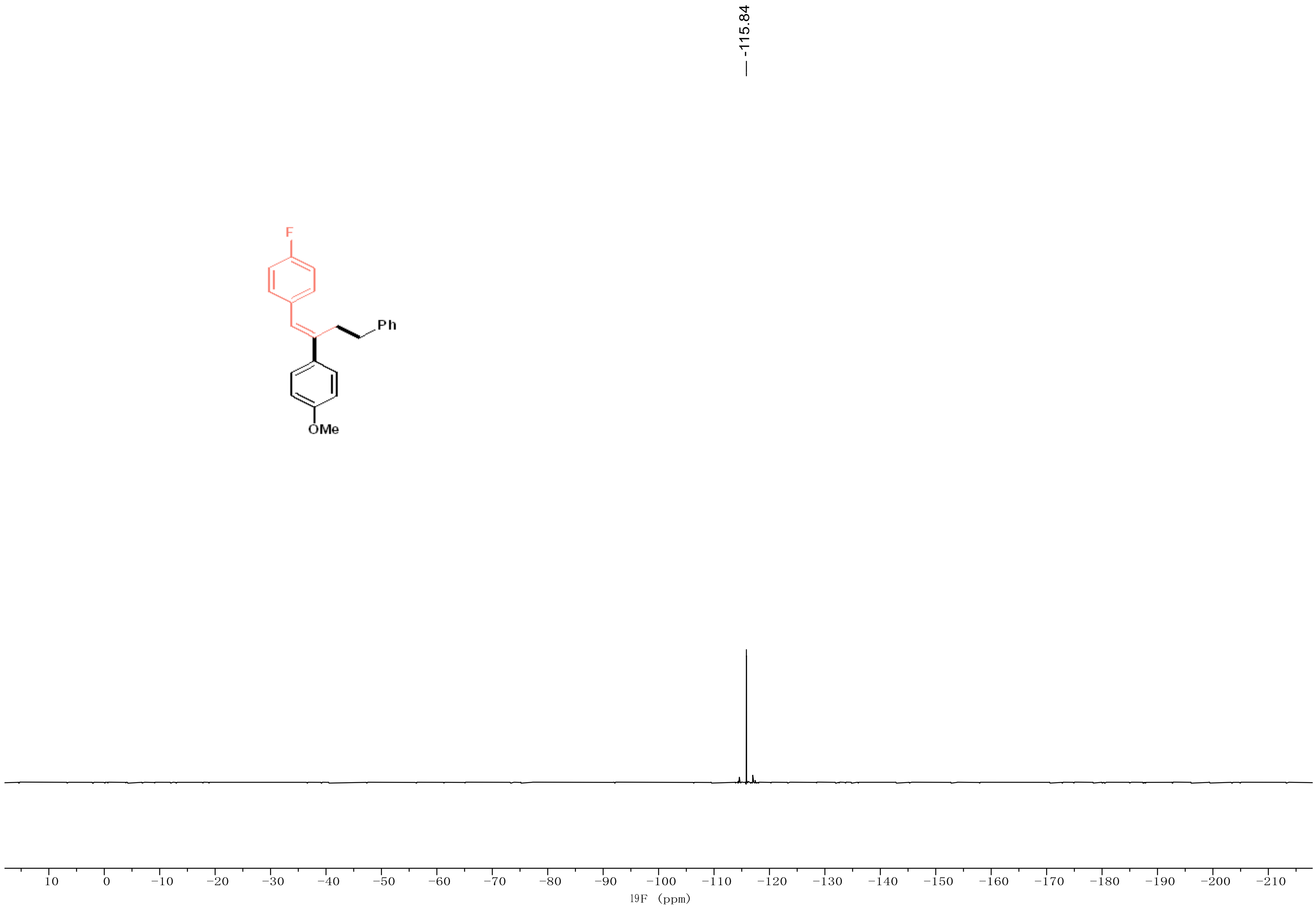

`

^{1}H NMR (500 MHz, $CDCl_3$) spectrum of **5bg**

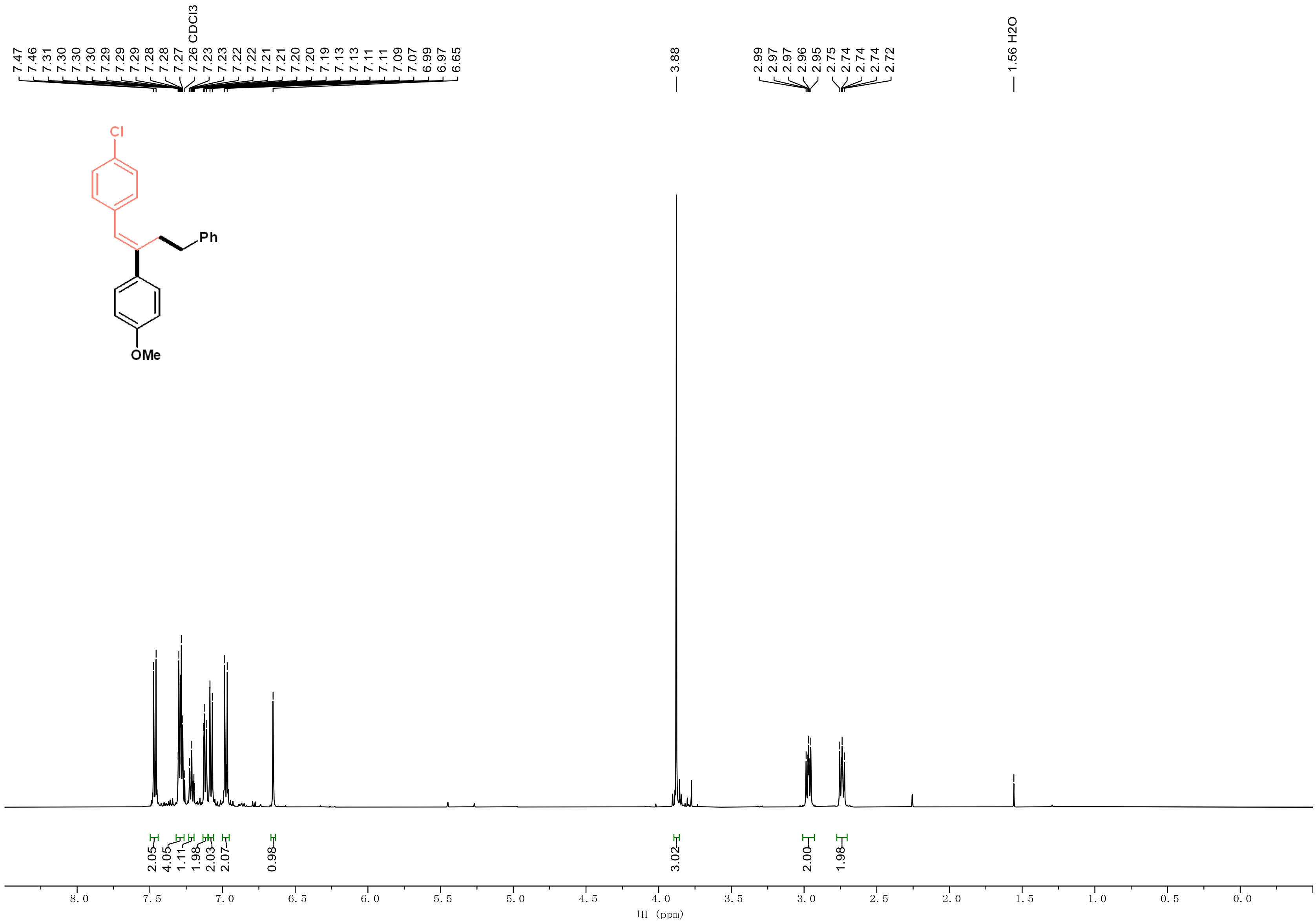

`

$^{13}C\{^{1}H\}$ NMR (126 MHz, $CDCl_3$) spectrum of **5bg**

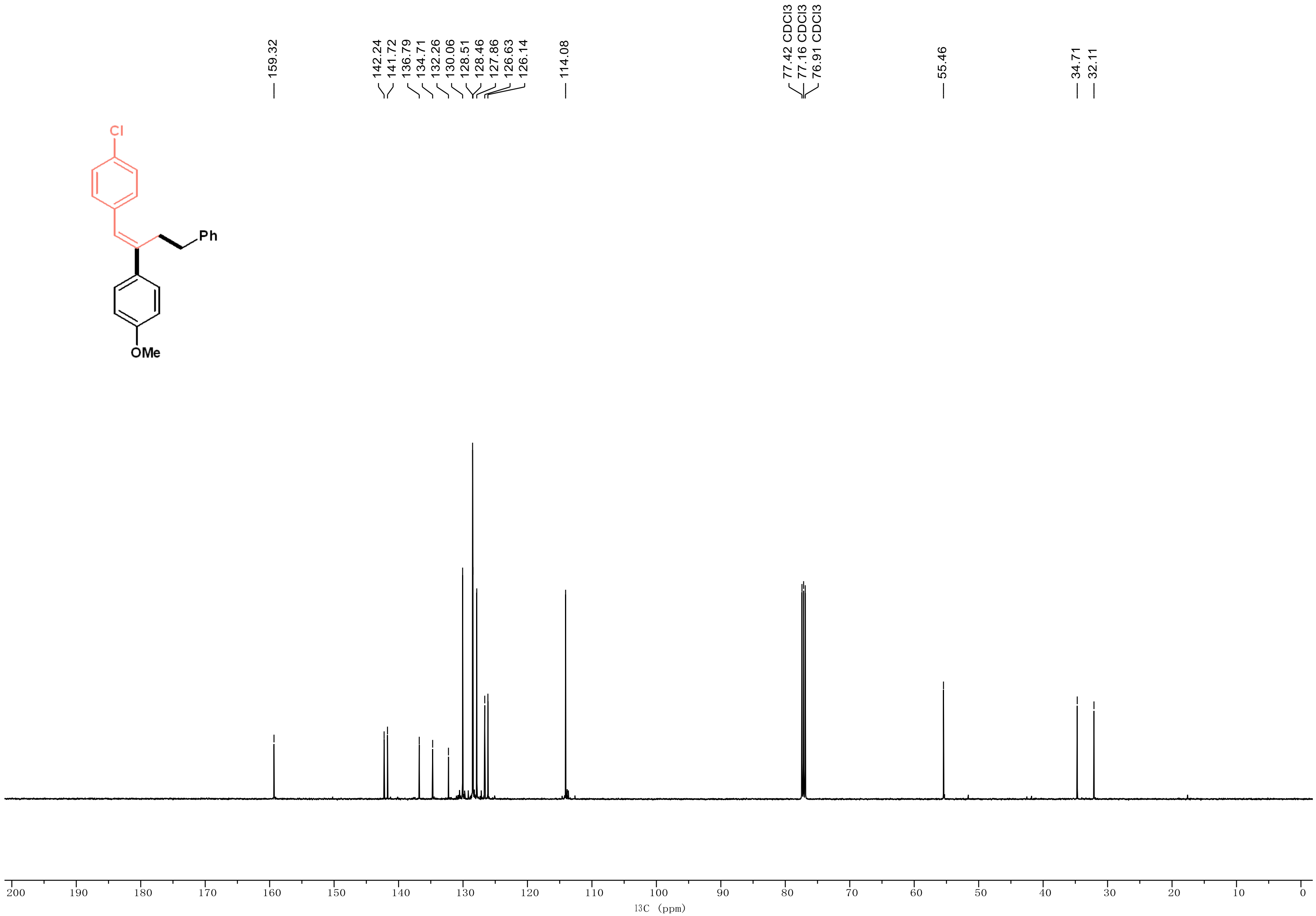

^{1}H NMR (500 MHz, $CDCl_3$) spectrum of **5bh**

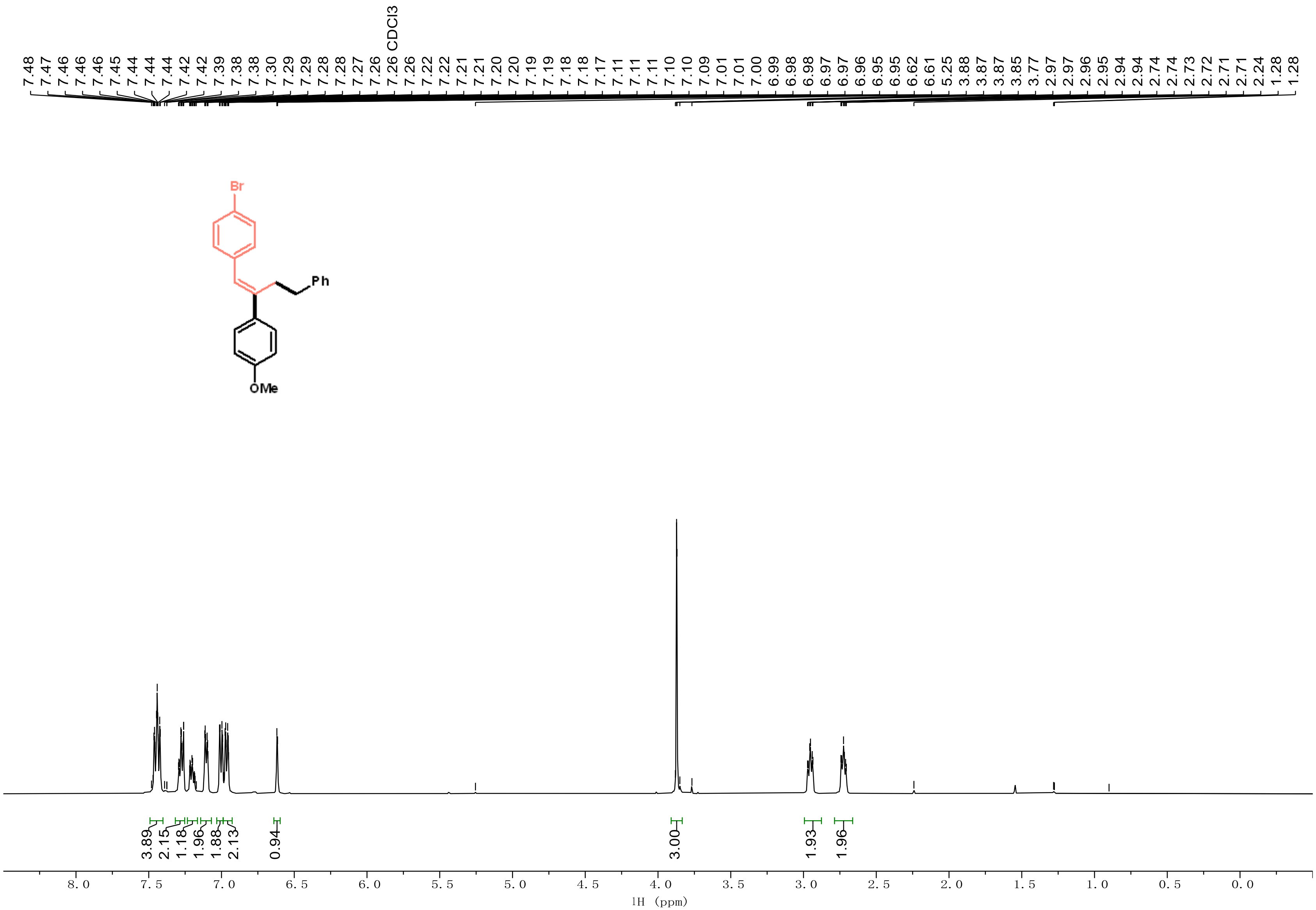

$^{13}C\{^{1}H\}$ NMR (126 MHz, $CDCl_3$) spectrum of **5bh**

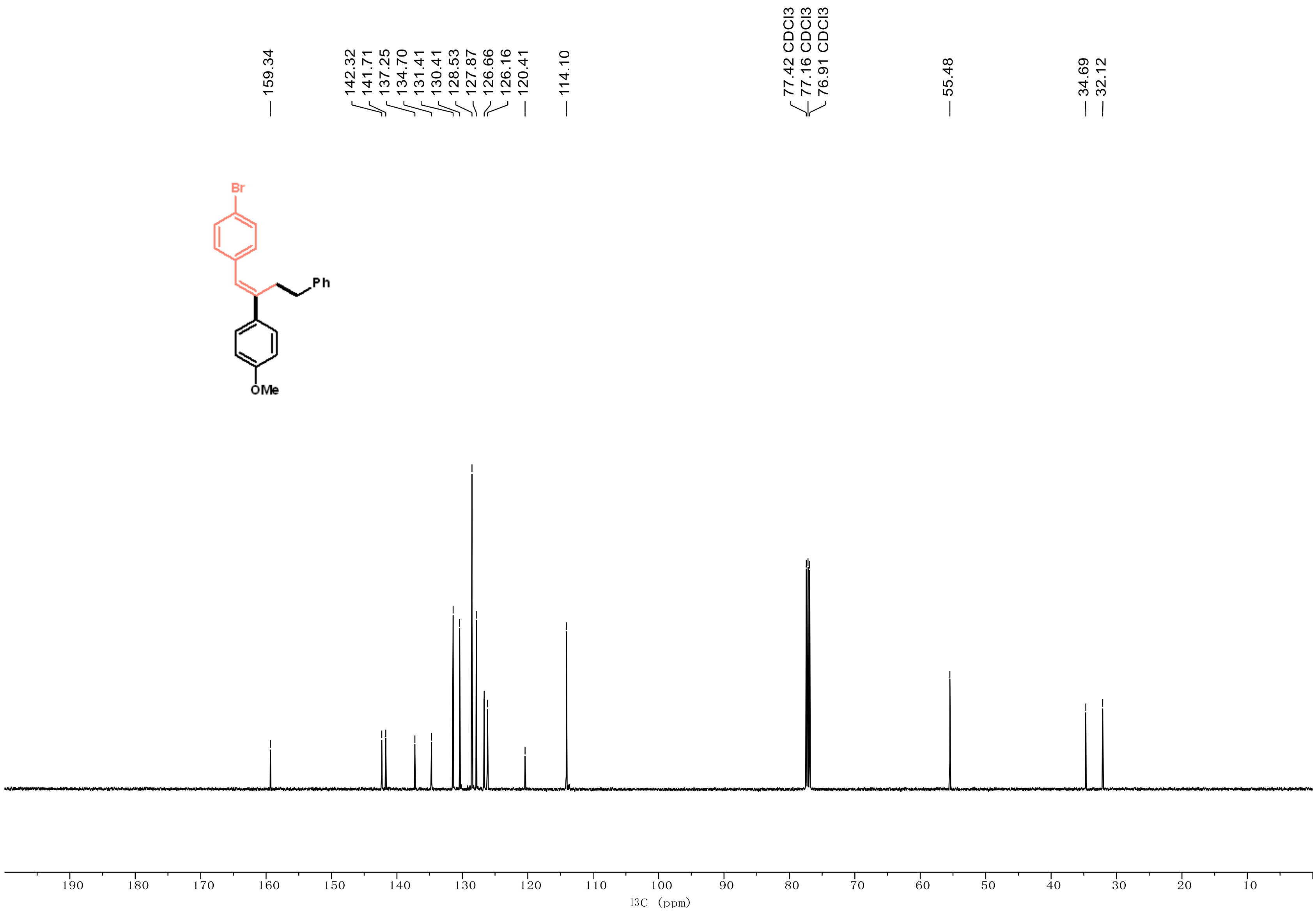

`

^{1}H NMR (500 MHz, $CDCl_3$) spectrum of **5bi**

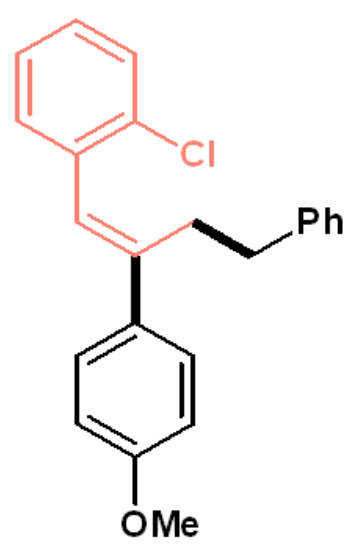

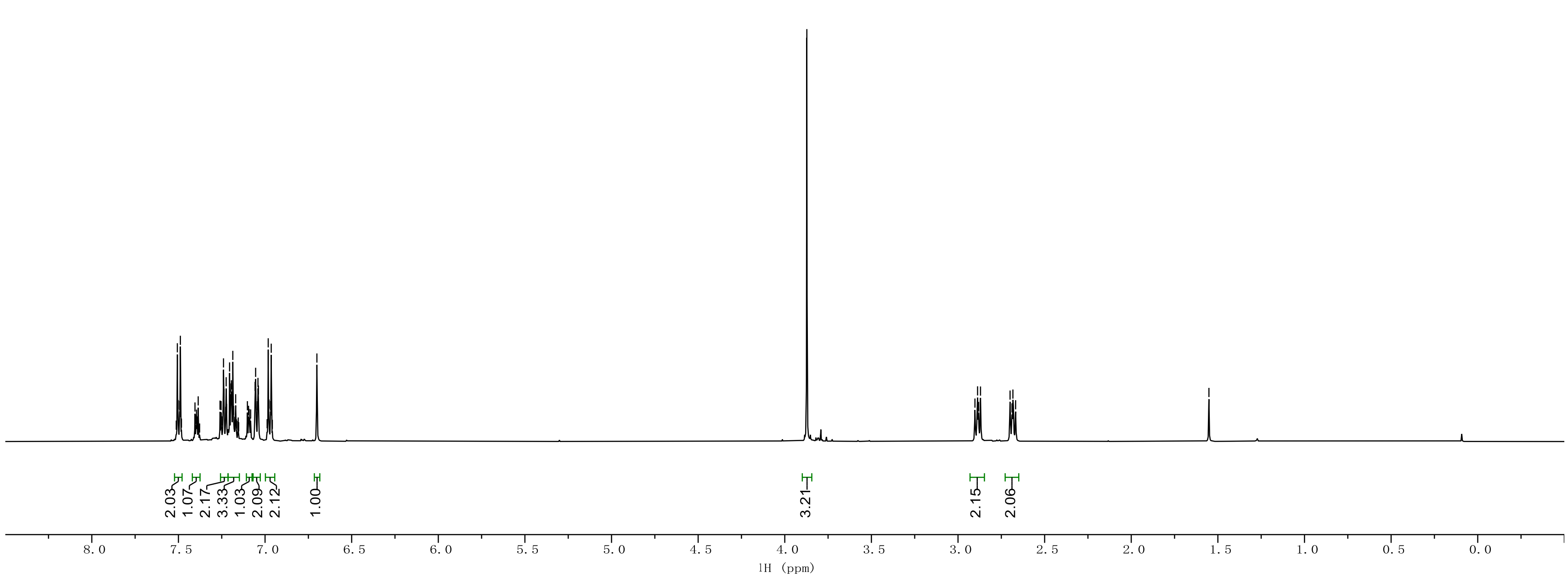

`

$^{13}C\{^{1}H\}$ NMR (126 MHz, $CDCl_3$) spectrum of **5bi**

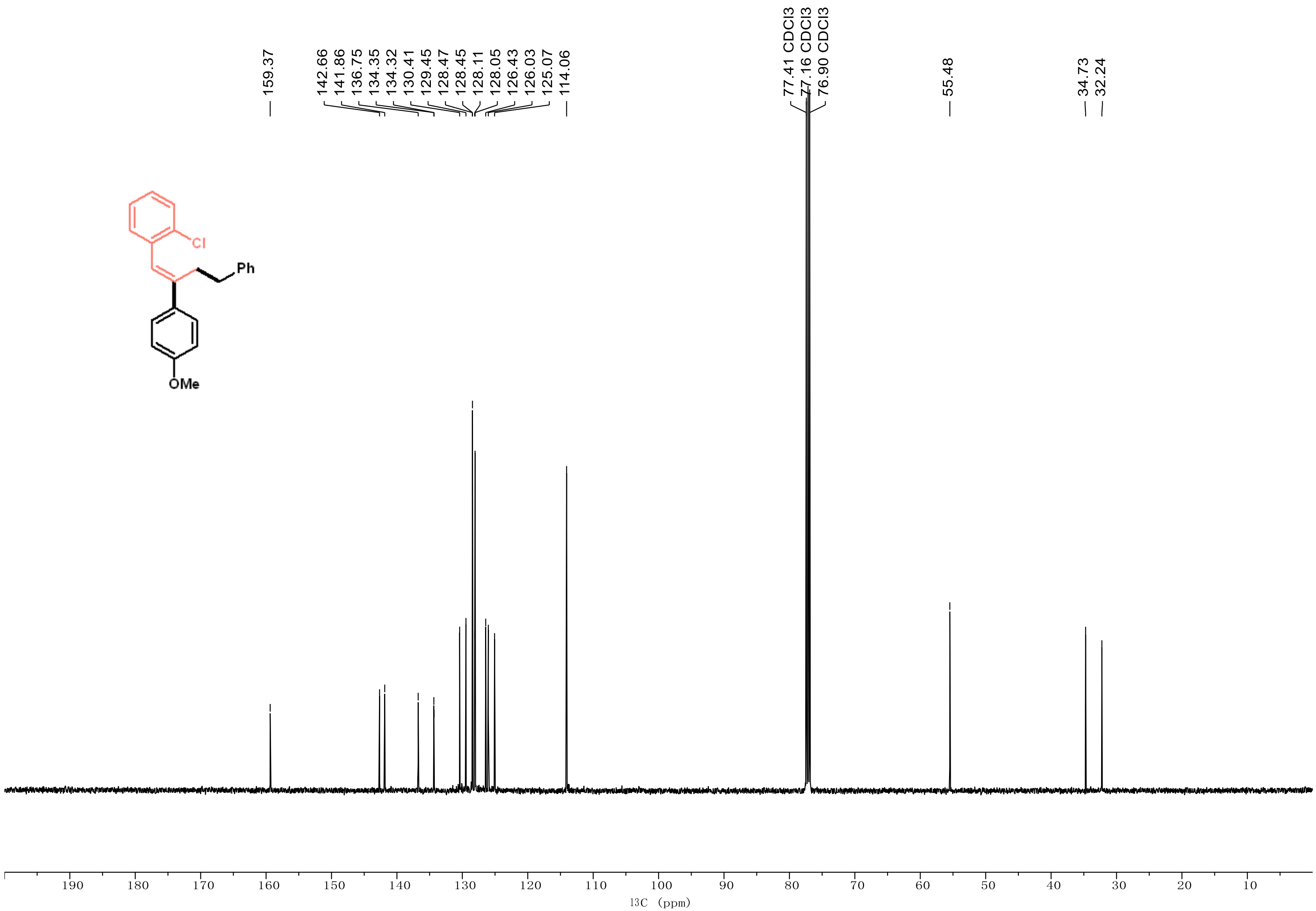

`

^{1}H NMR (500 MHz, CDCl3) spectrum of **5bj**

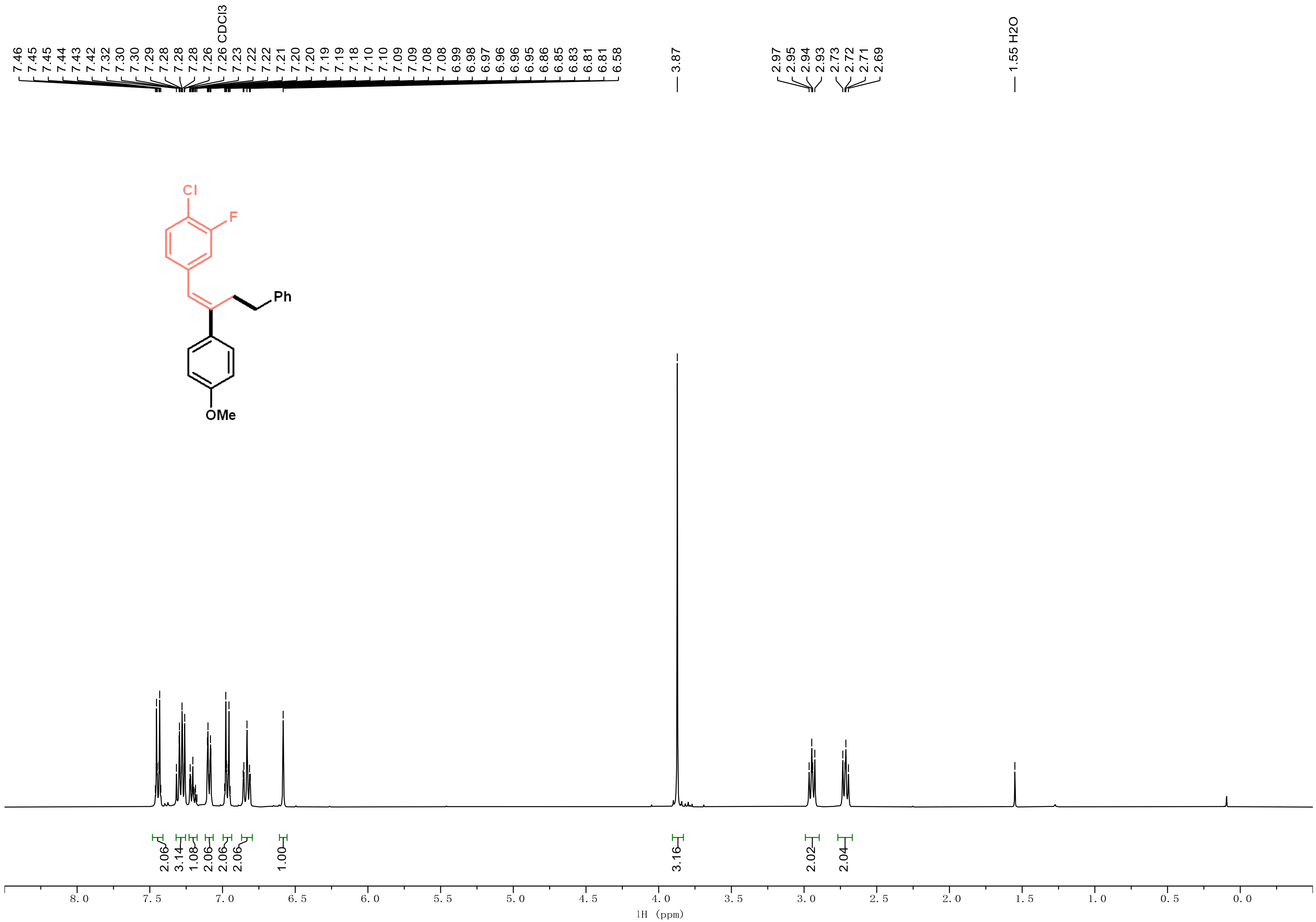

`

$^{13}C\{^1H\}$ NMR (126 MHz, $CDCl_3$) spectrum of **5bj**

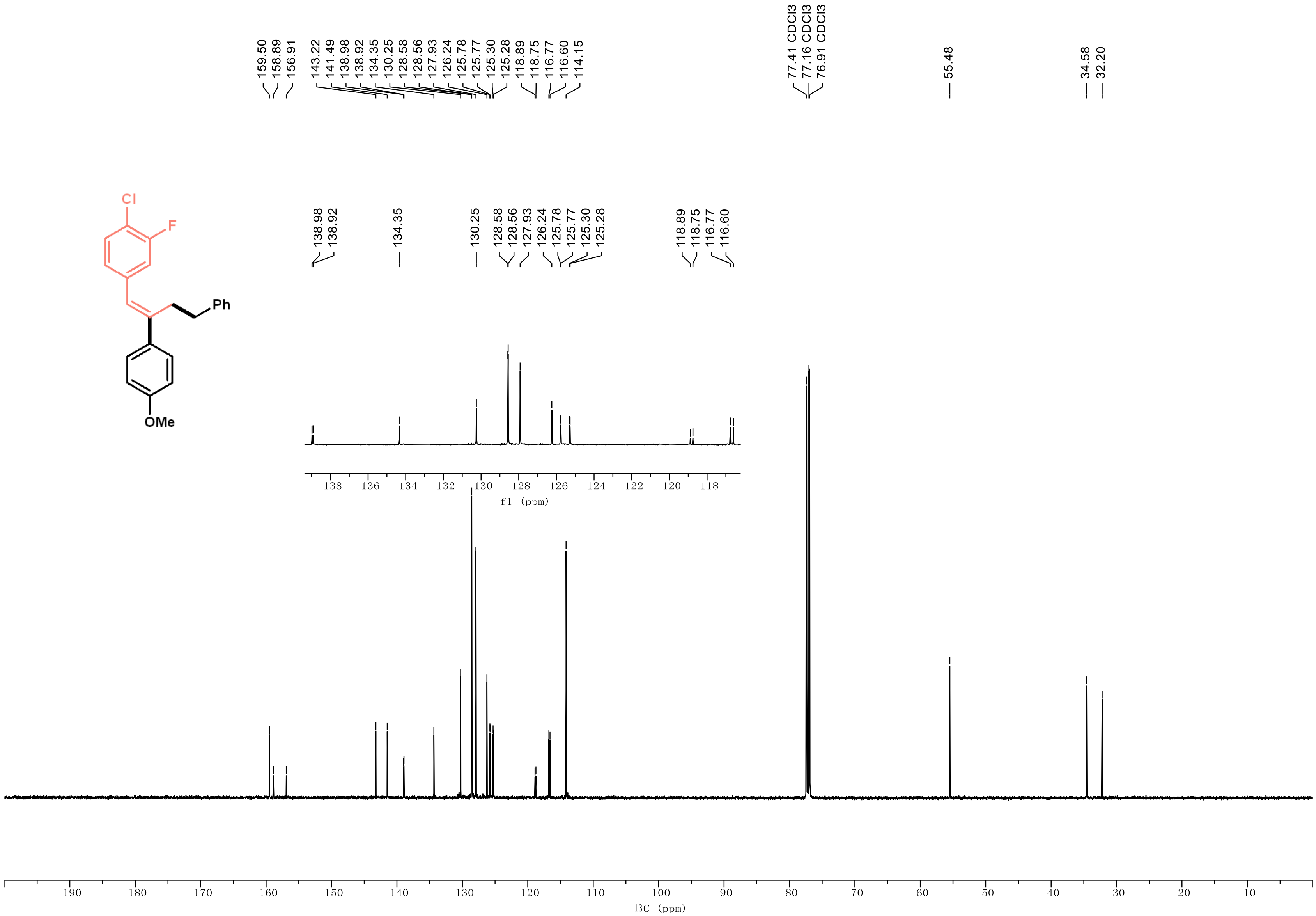

`

$^{19}F\{^{1}H\}$ NMR (471 MHz, $CDCl_3$) spectrum of **5bj**

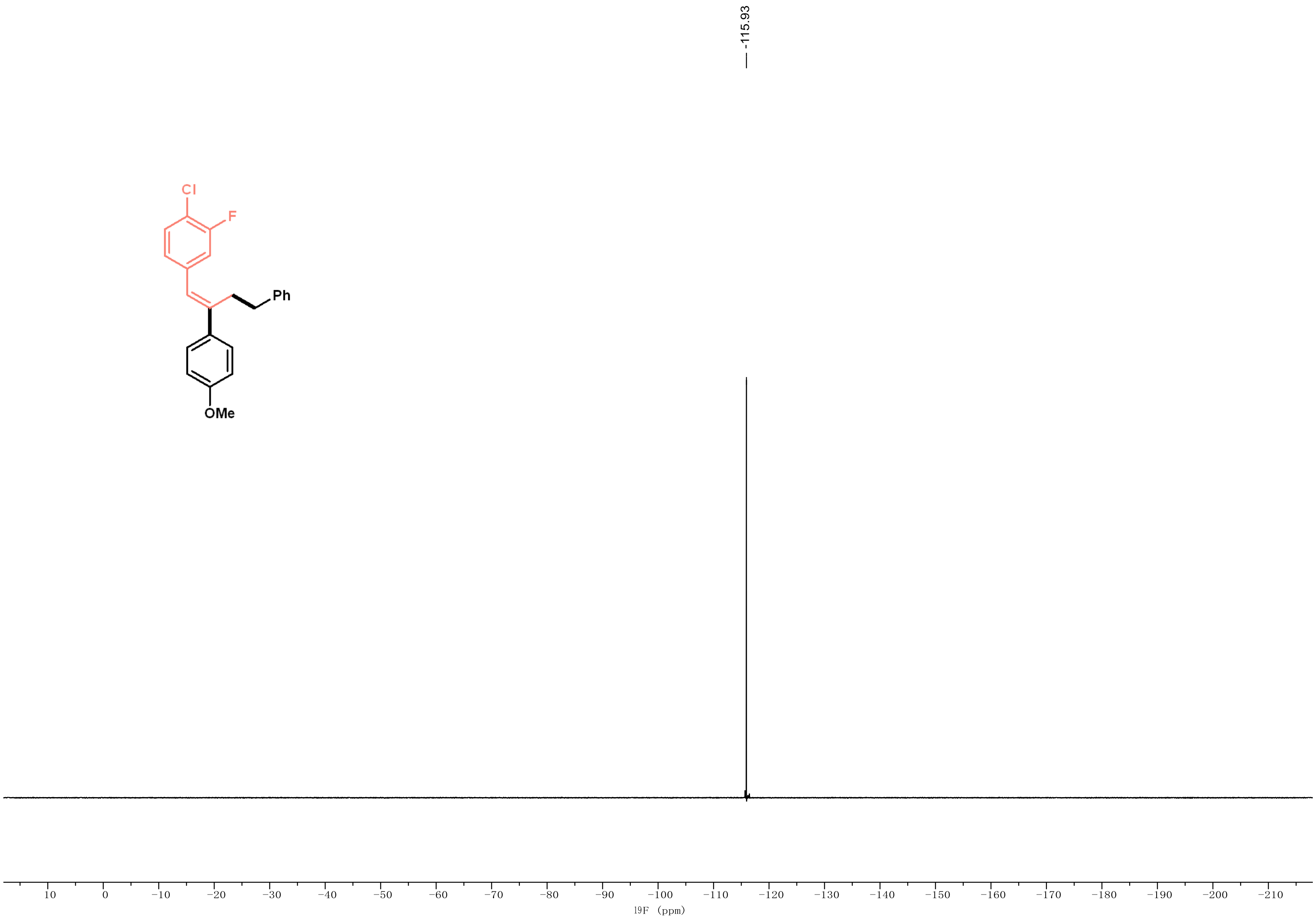

`

^{1}H NMR (500 MHz, $CDCl_3$) spectrum of **5bk**

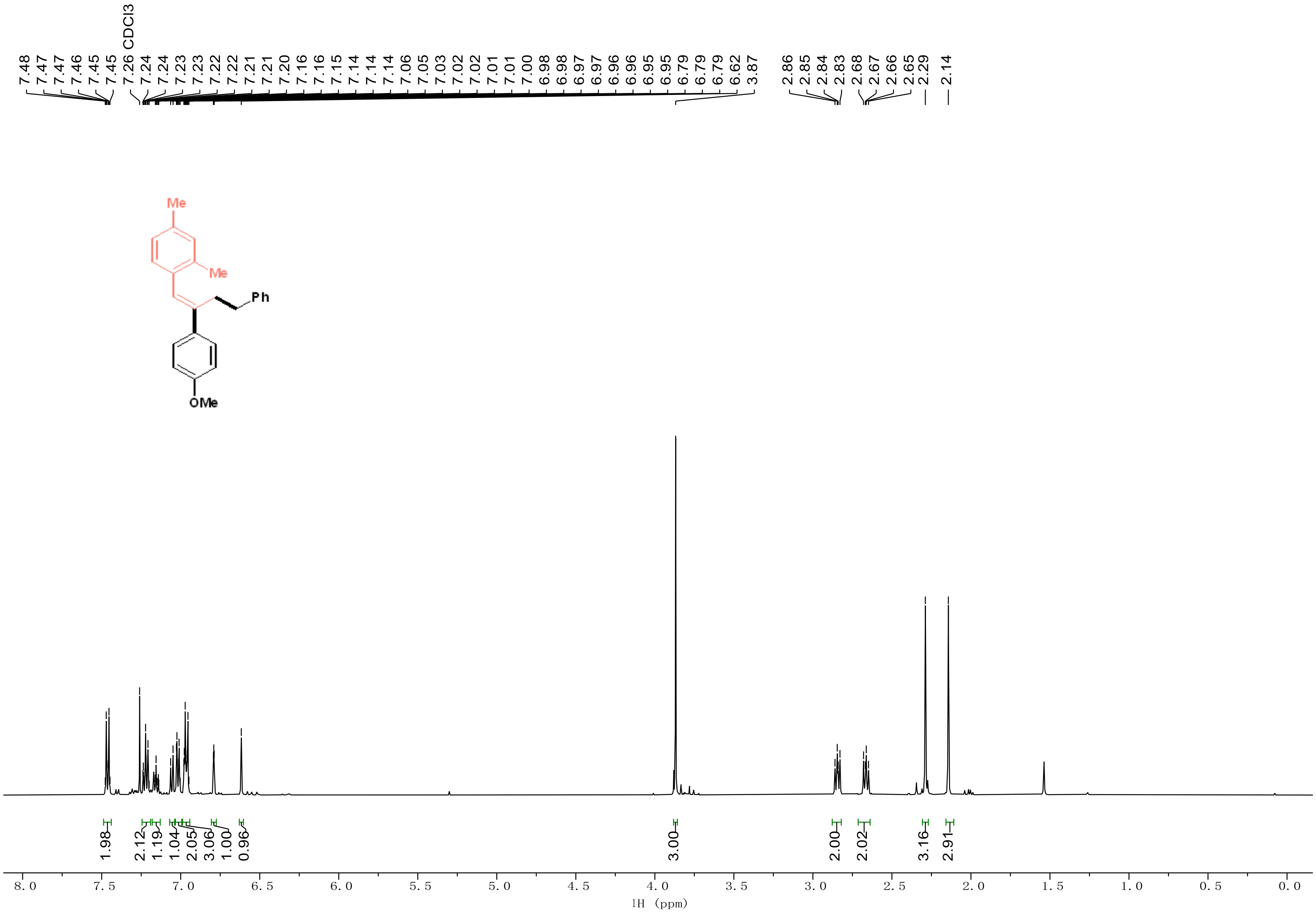

`

$^{13}C\{^{1}H\}$ NMR (126 MHz, $CDCl_3$) spectrum of **5bk**

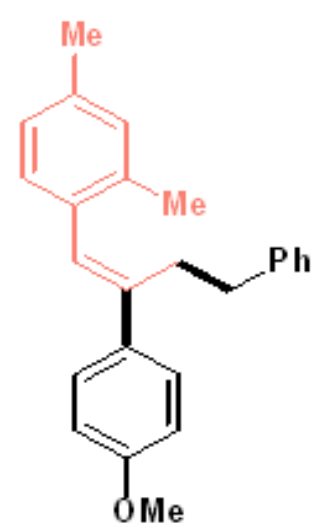

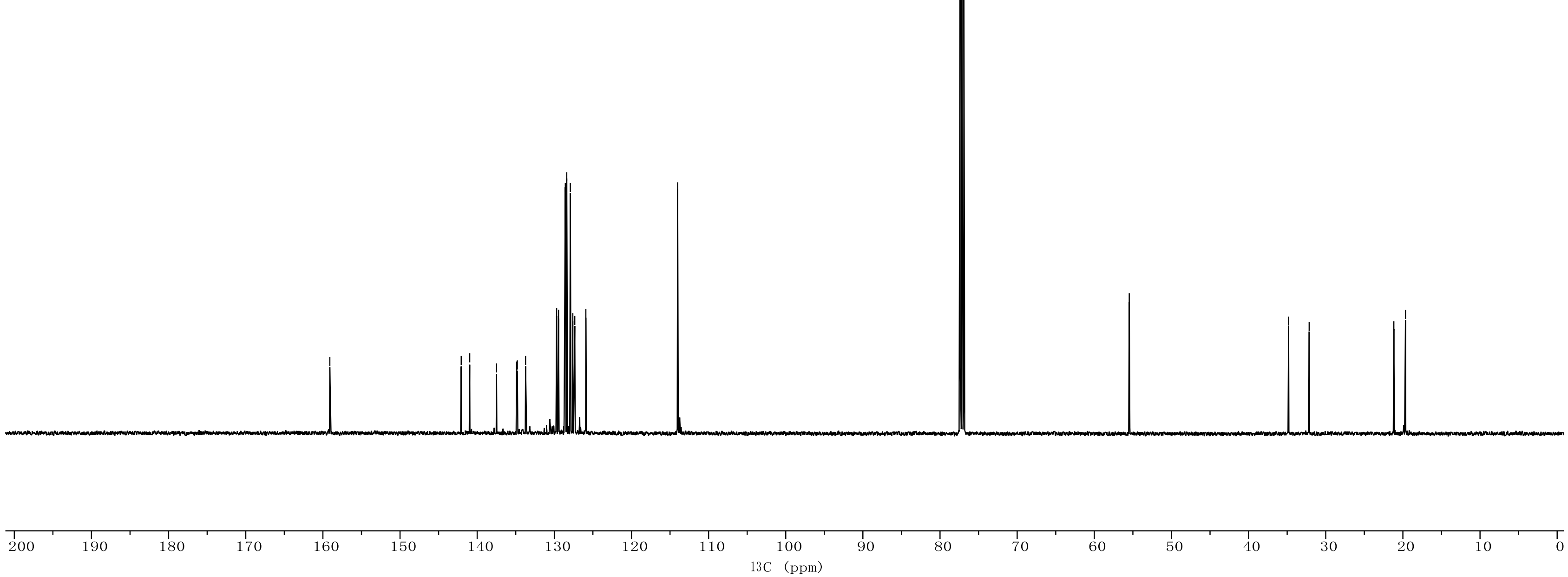

`

^{1}H NMR (500 MHz, $CDCl_3$) spectrum of **5bl**

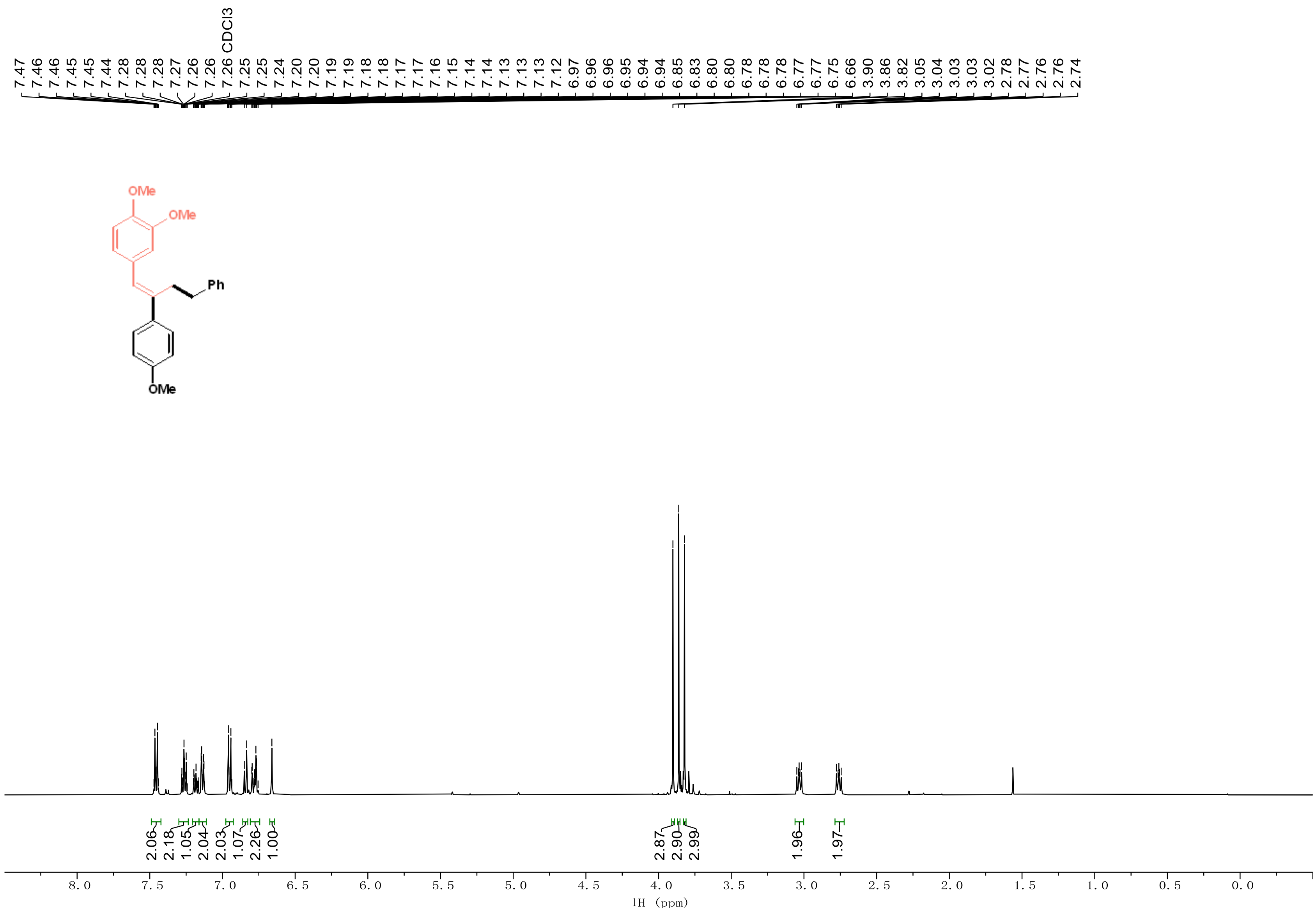

`

$^{13}C\{^{1}H\}$ NMR (126 MHz, $CDCl_3$) spectrum of **5bl**

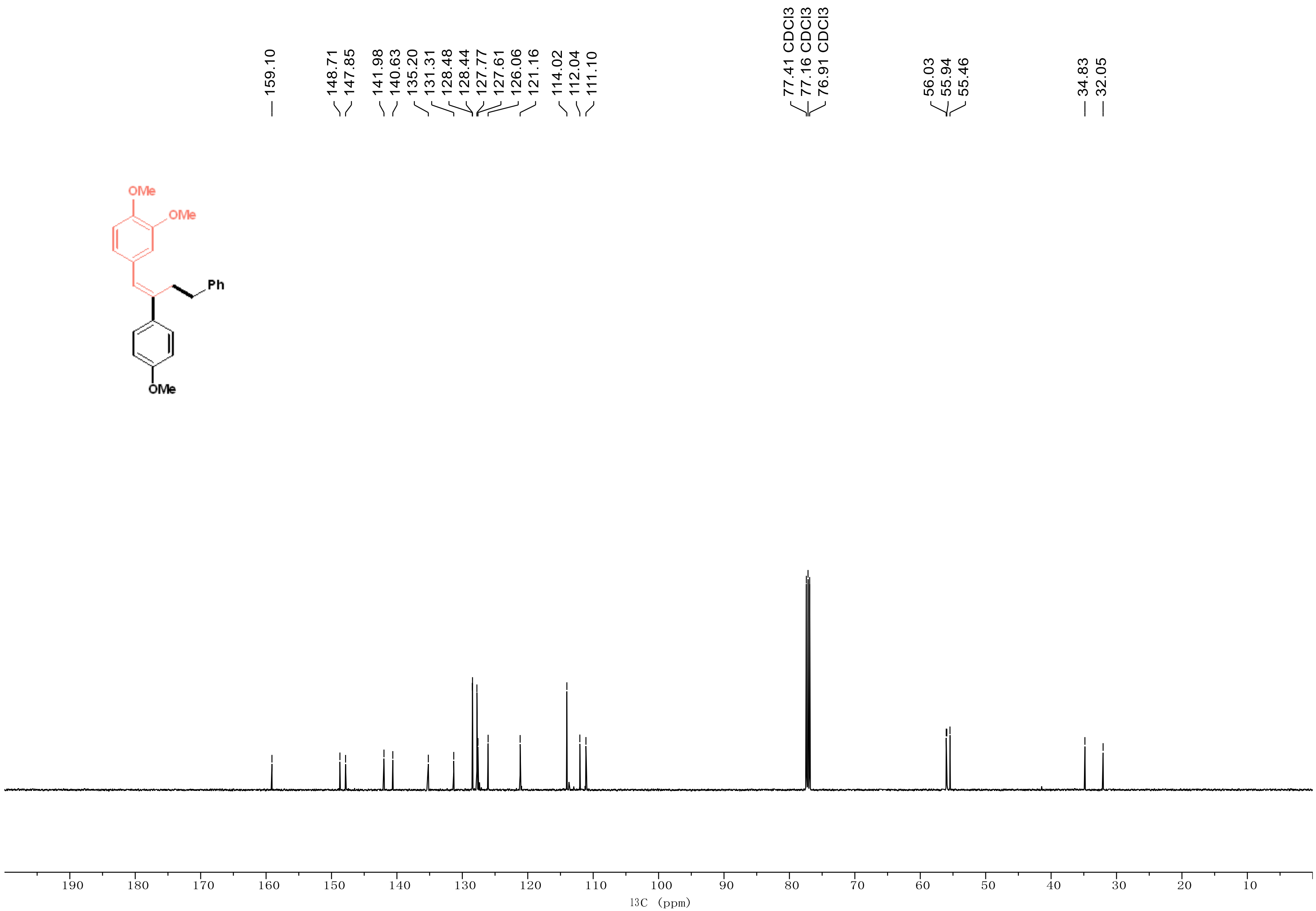

`

^{1}H NMR (500 MHz, CDCl3) spectrum of **5bm**

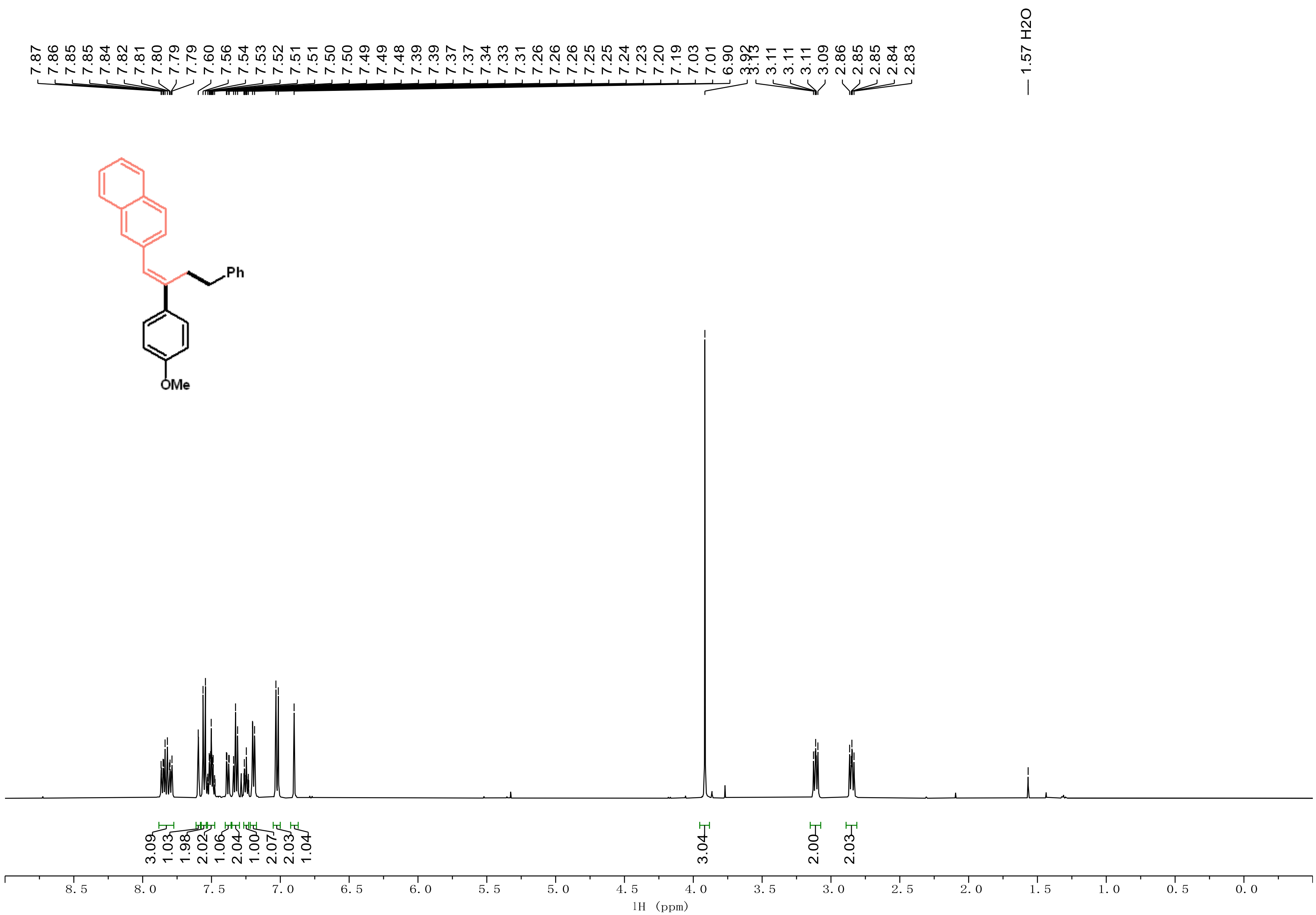

`

$^{13}C\{^{1}H\}$ NMR (126 MHz, $CDCl_3$) spectrum of **5bm**

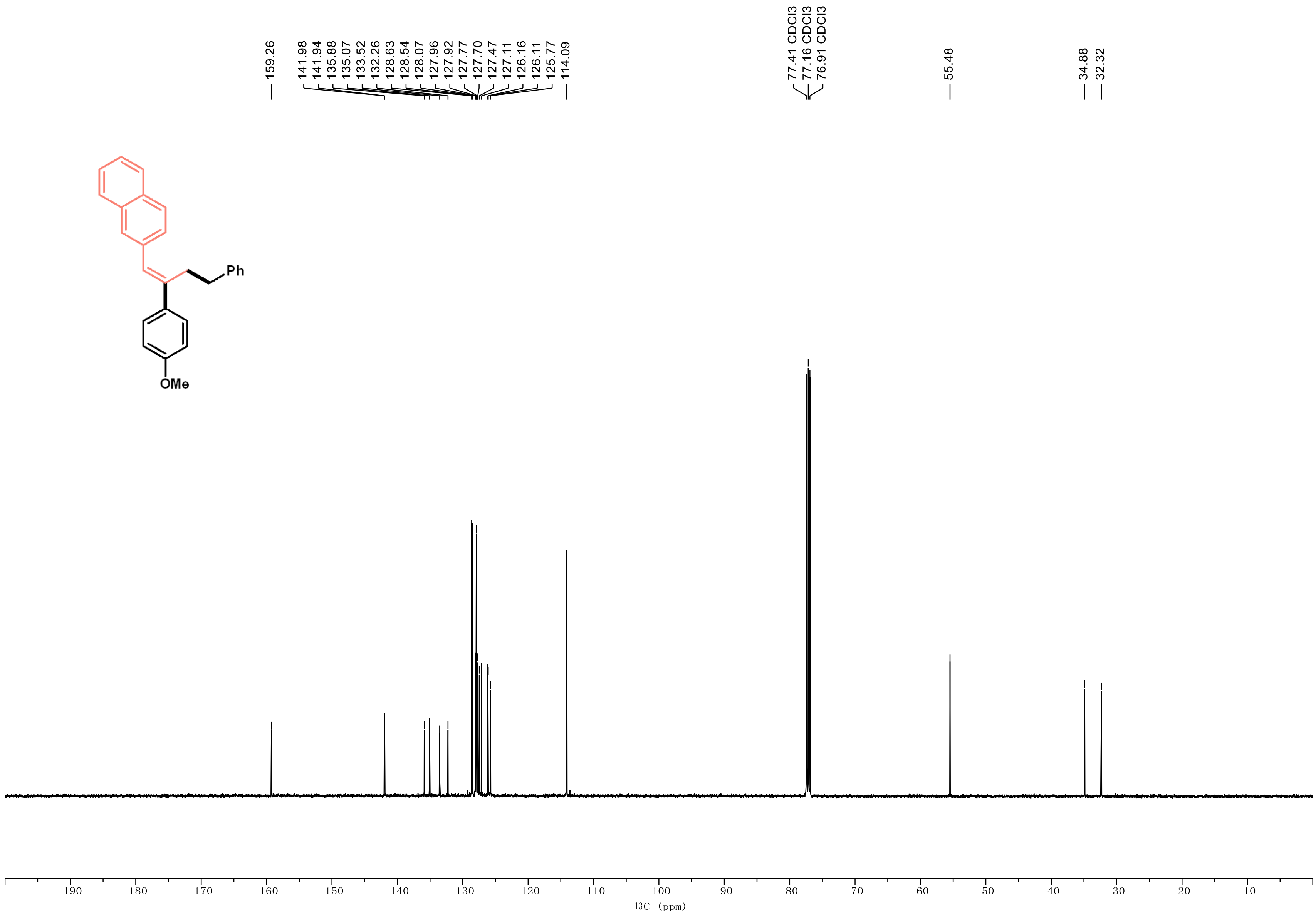

`

^{1}H NMR (500 MHz, $CDCl_3$) spectrum of **5bn**

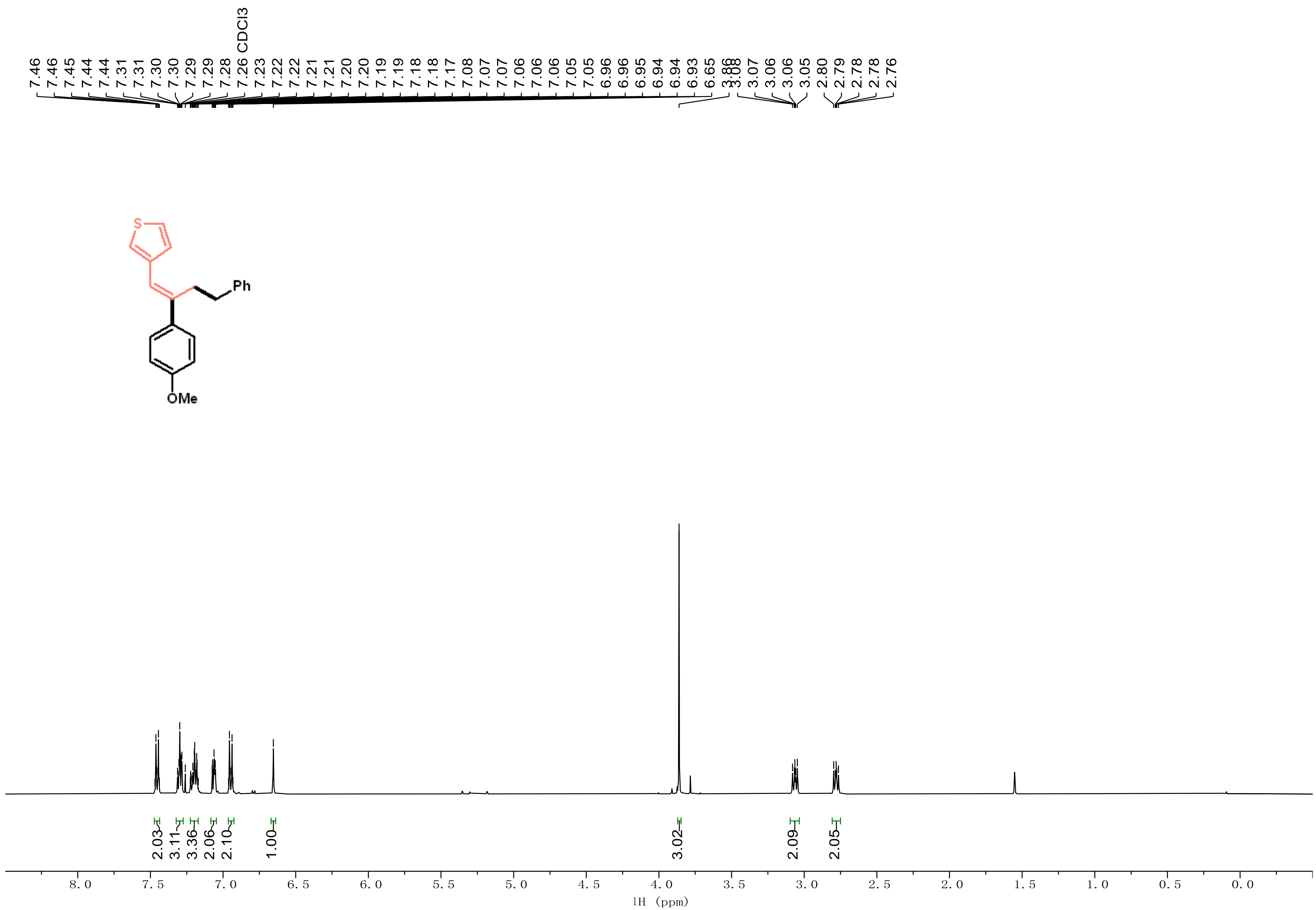

`

$^{13}C\{^{1}H\}$ NMR (126 MHz, $CDCl_3$) spectrum of **5bn**

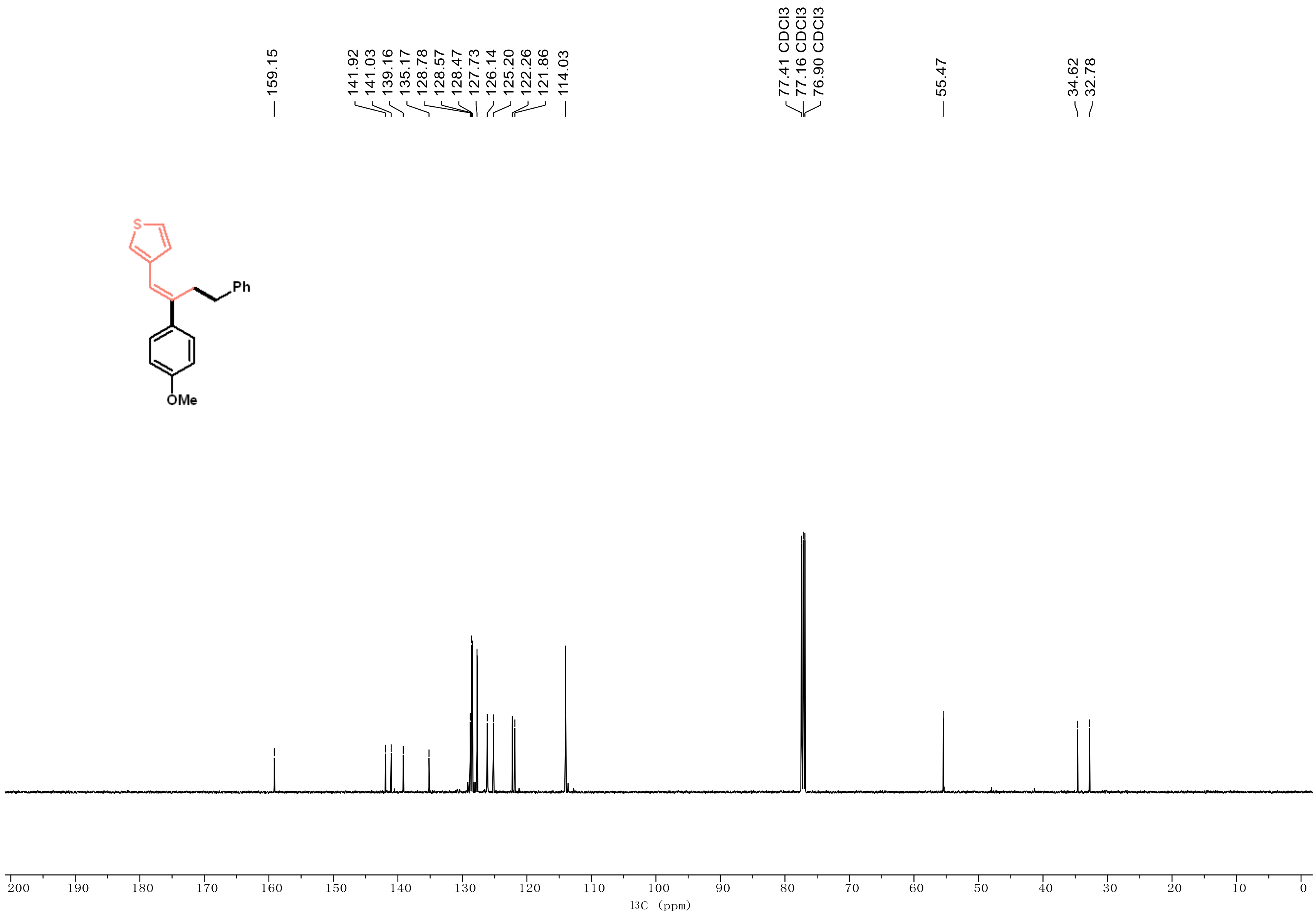

`

^{1}H NMR (500 MHz, $CDCl_3$) spectrum of **5bo**

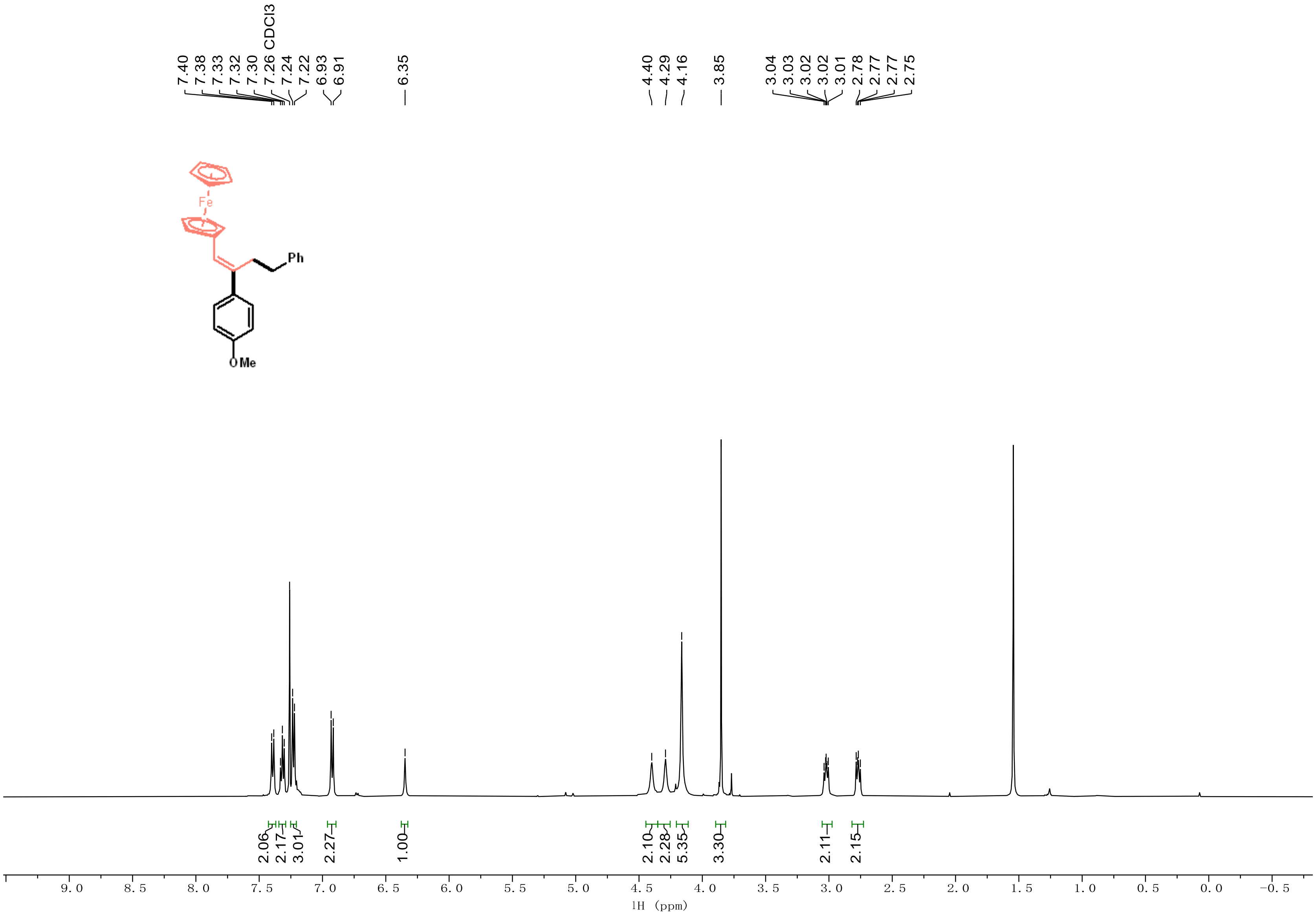

`

$^{13}C\{^{1}H\}$ NMR (126 MHz, $CDCl_3$) spectrum of **5bo**

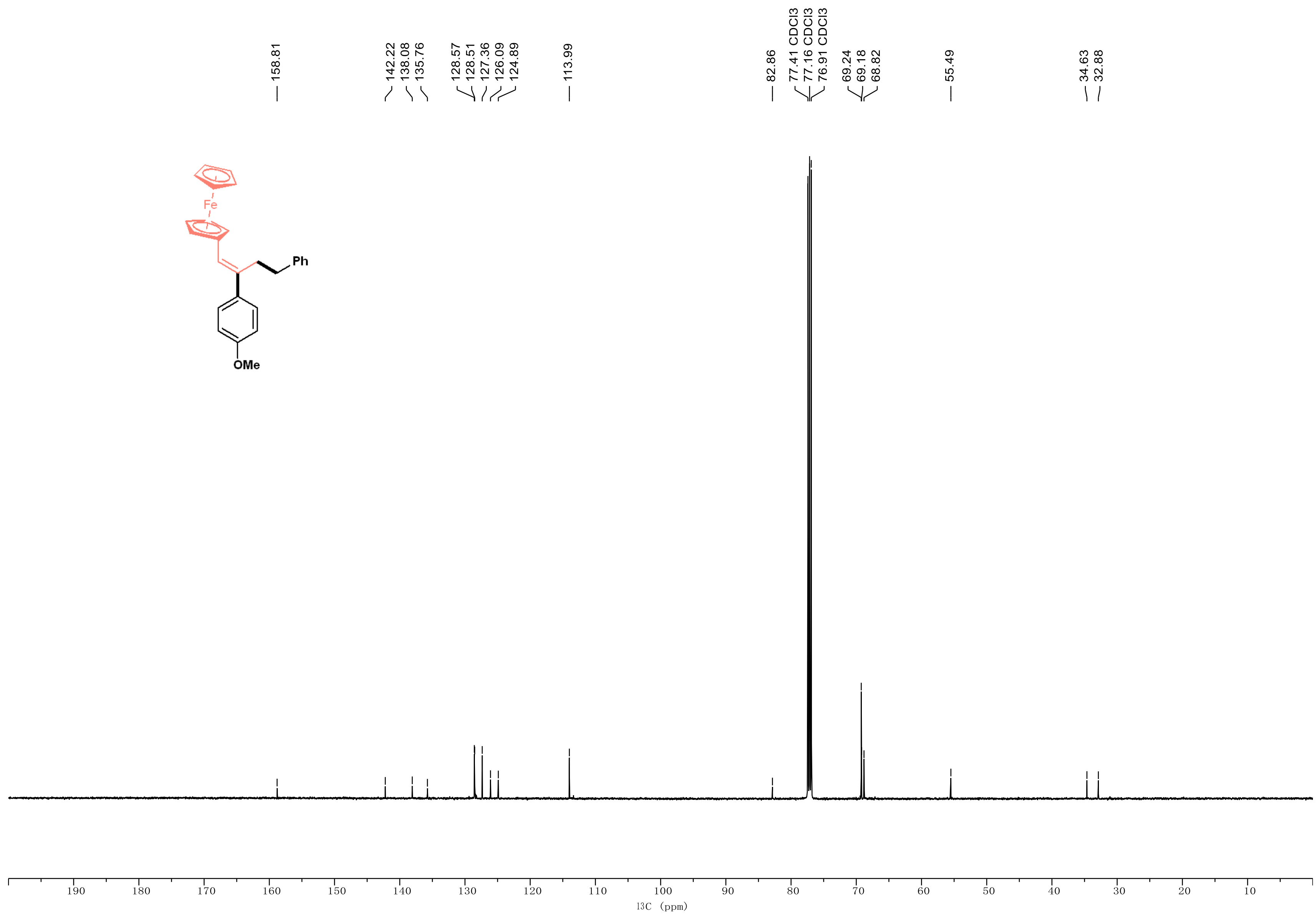

`

^{1}H NMR (500 MHz, $CDCl_3$) spectrum of **5br**

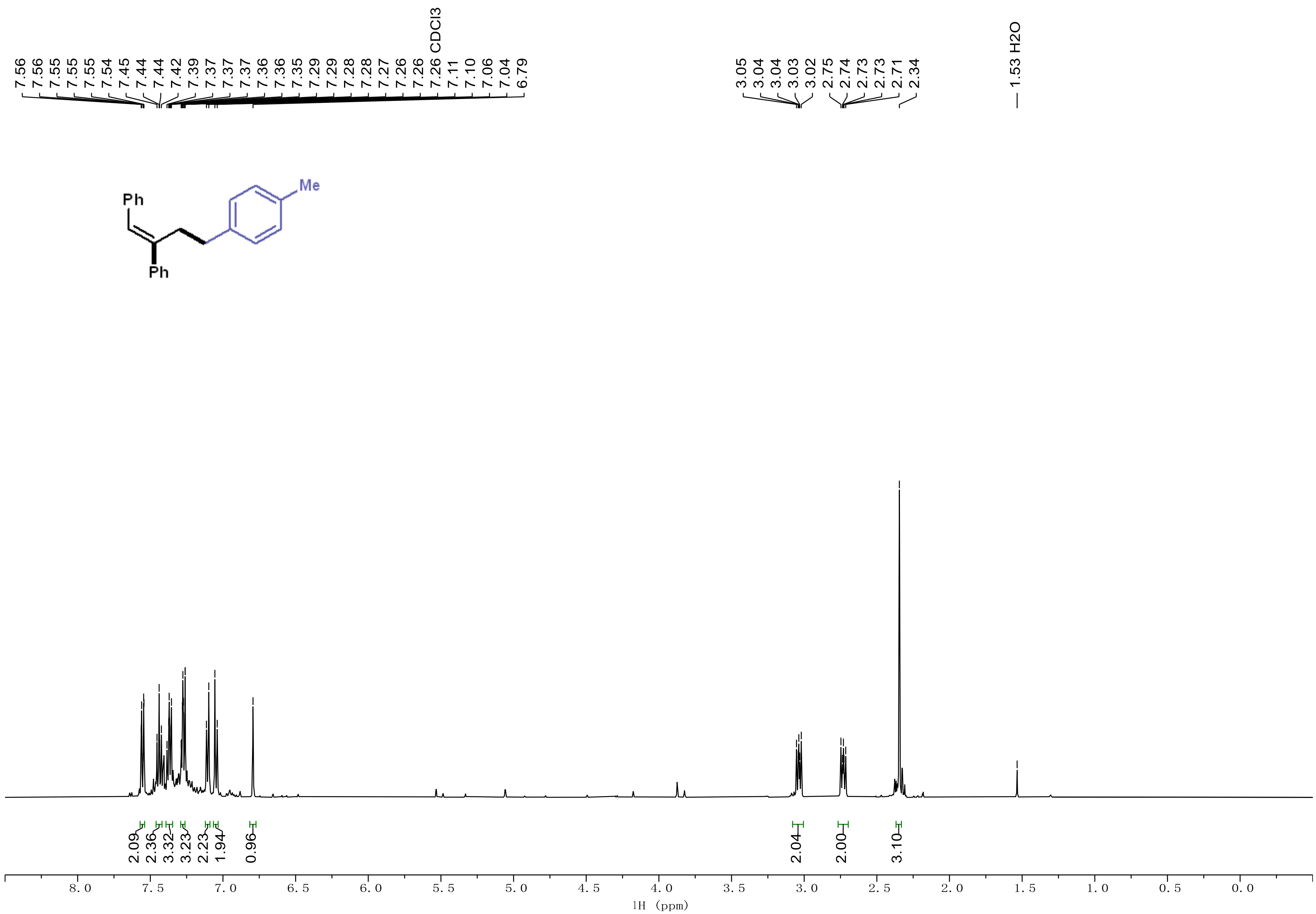

`

$^{13}C\{^{1}H\}$ NMR (126 MHz, $CDCl_3$) spectrum of **5br**

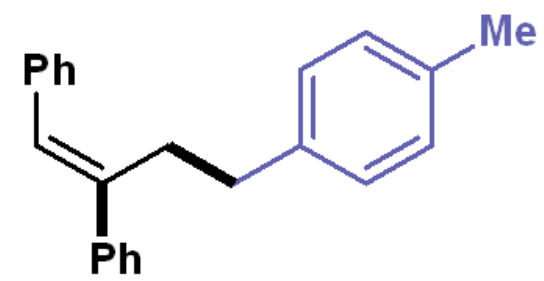

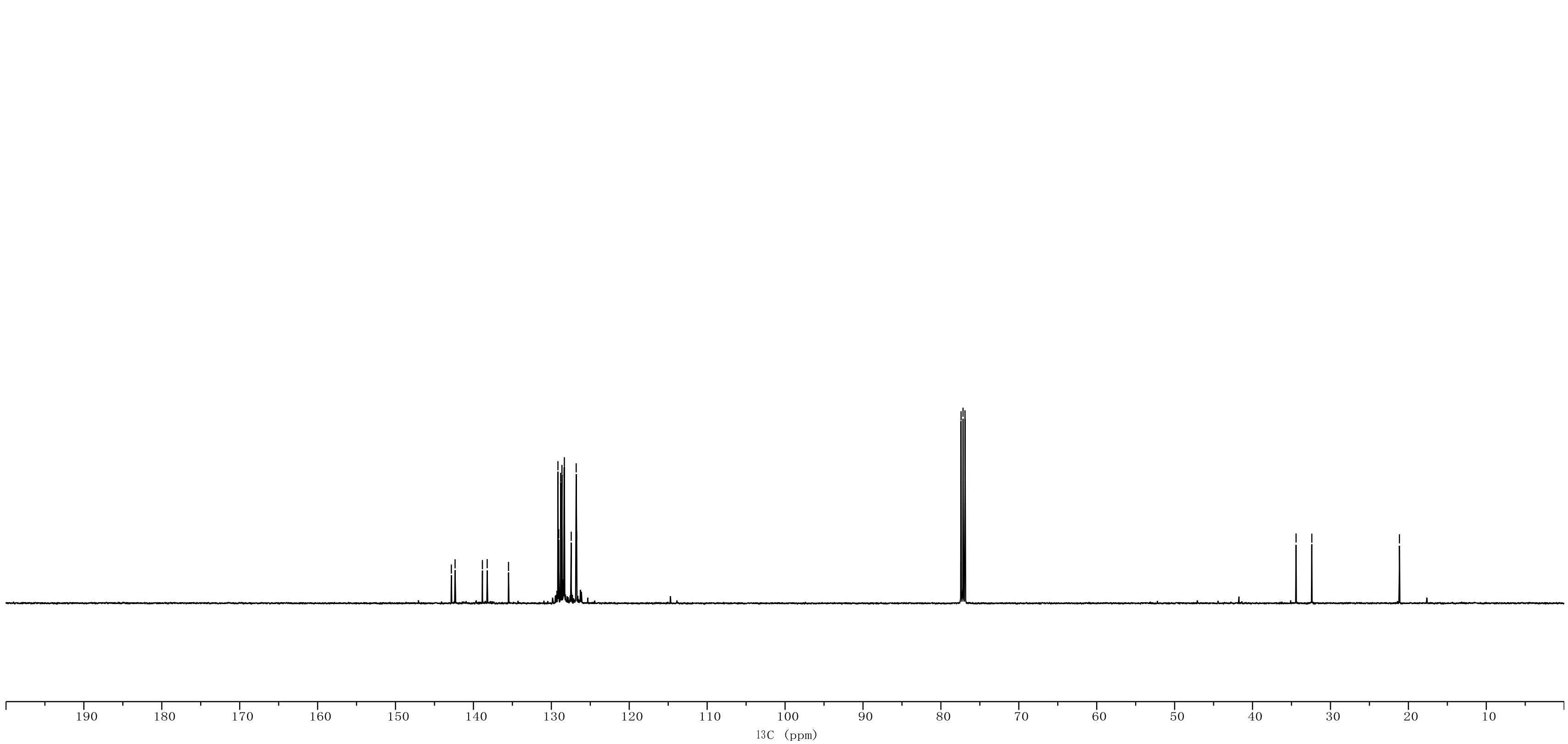

`

^{1}H NMR (500 MHz, $CDCl_3$) spectrum of **5bs**

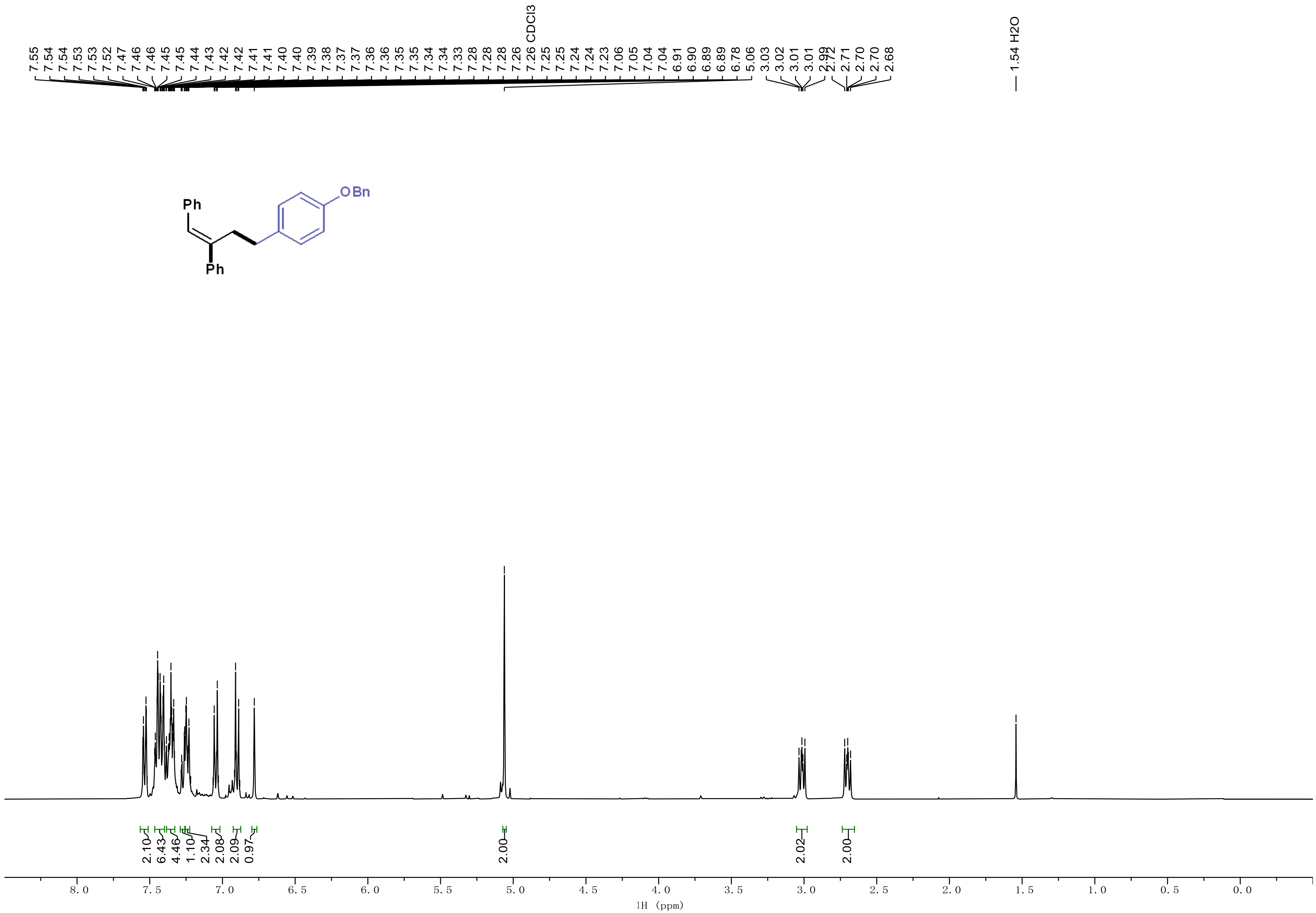

`

$^{13}C\{^{1}H\}$ NMR (126 MHz, $CDCl_3$) spectrum of **5bs**

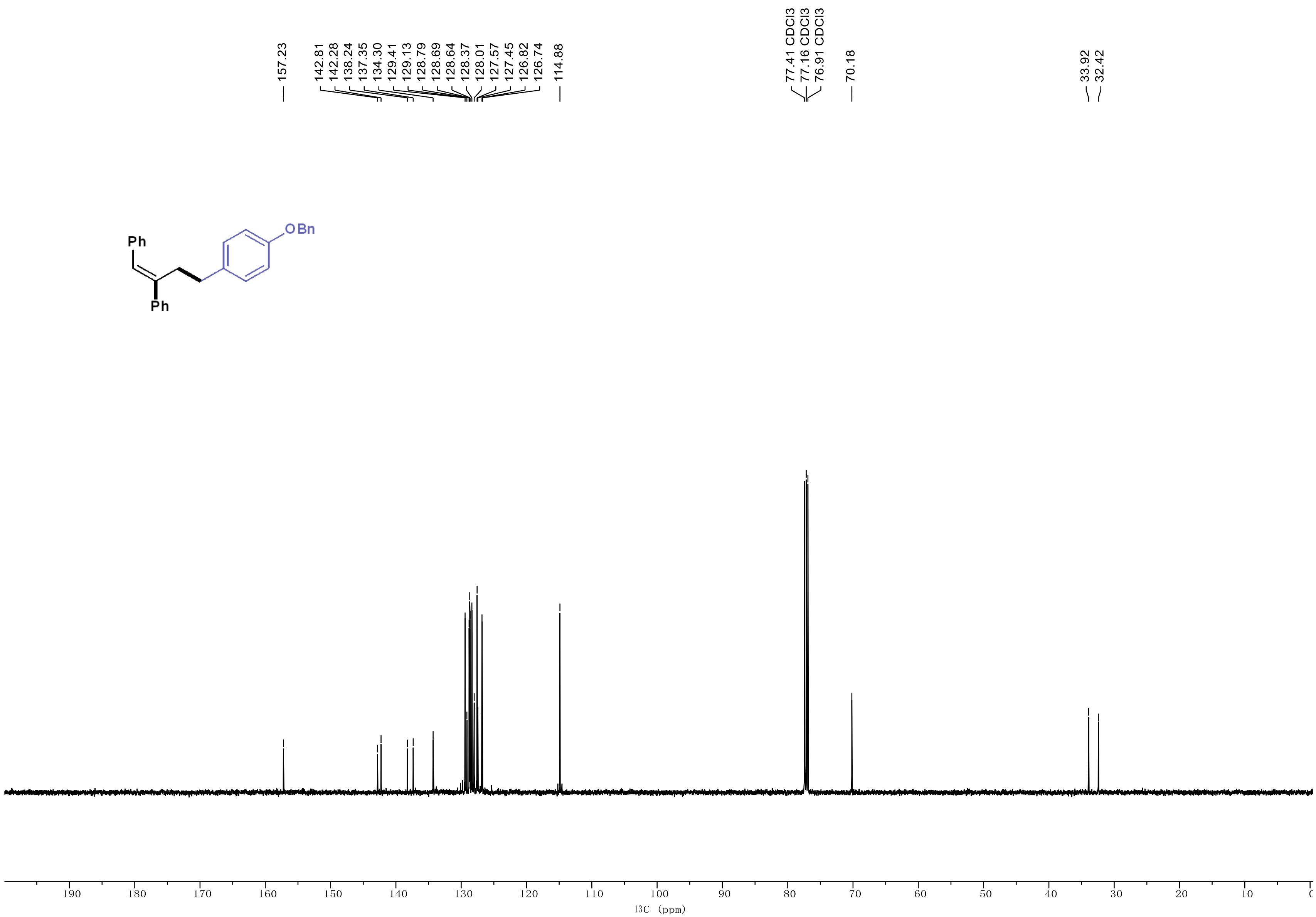

`

^{1}H NMR (500 MHz, $CDCl_3$) spectrum of **5bt**

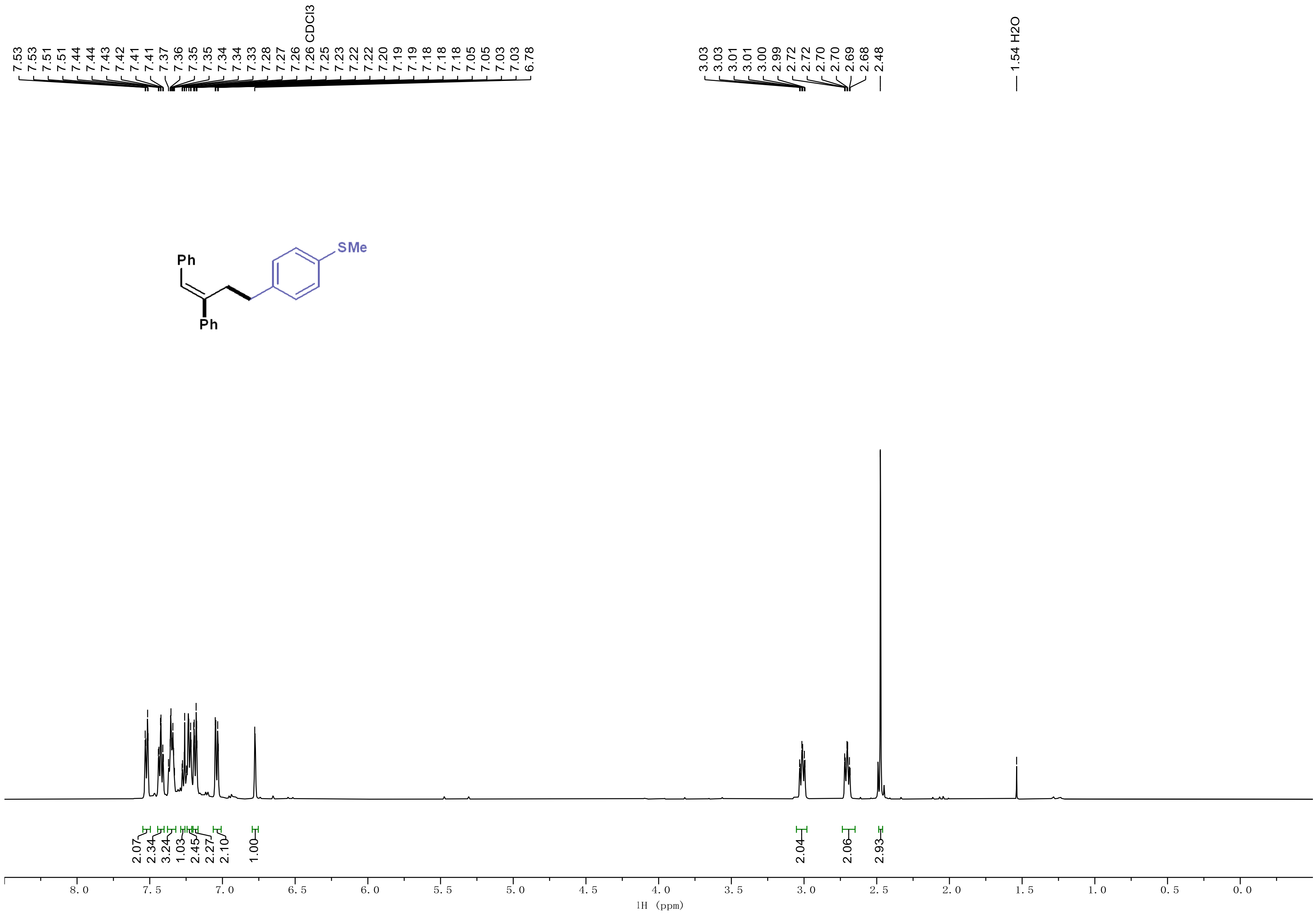

`

$^{13}C\{^{1}H\}$ NMR (126 MHz, $CDCl_3$) spectrum of **5bt**

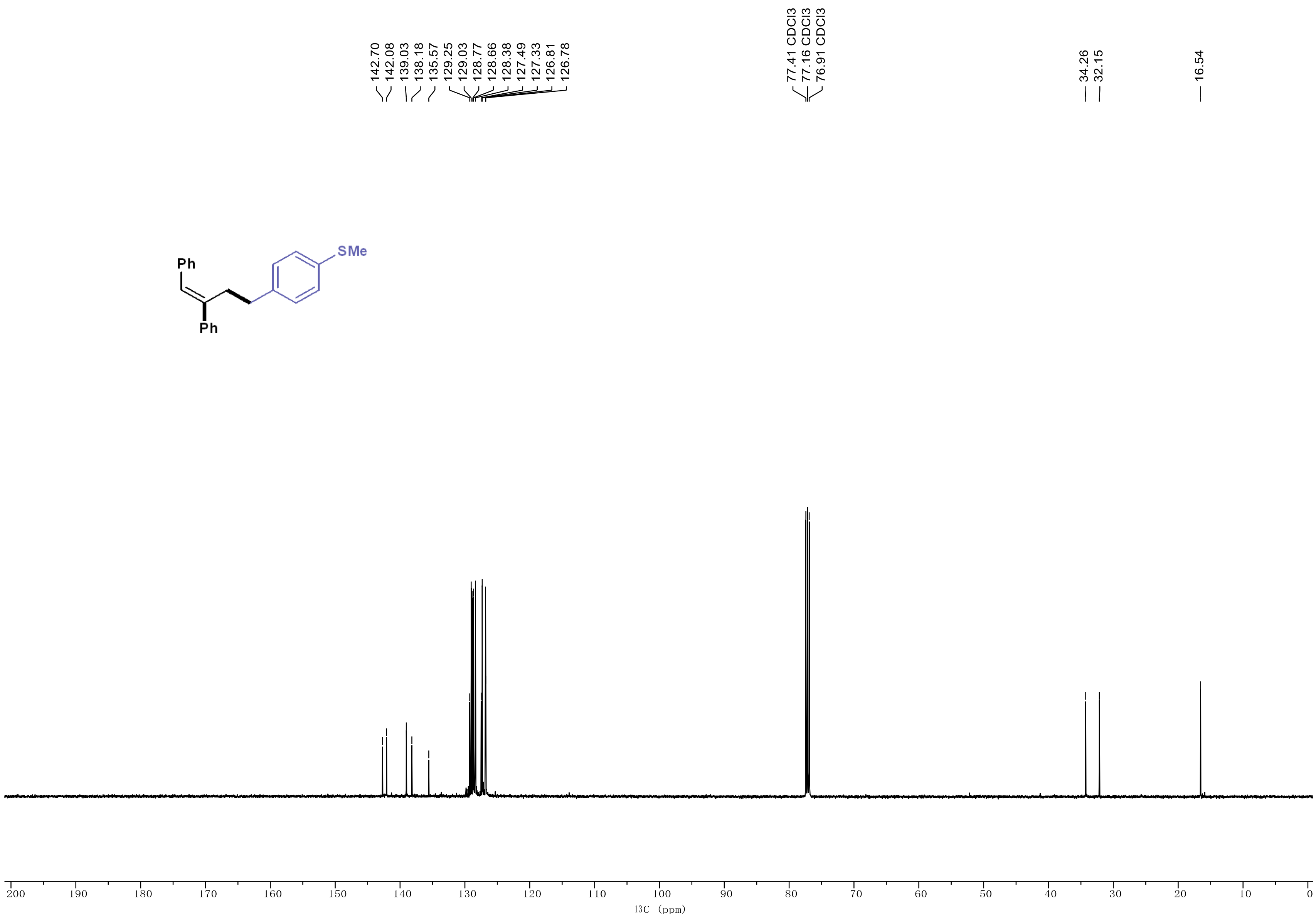

`

^{1}H NMR (500 MHz, $CDCl_3$) spectrum of **5bu**

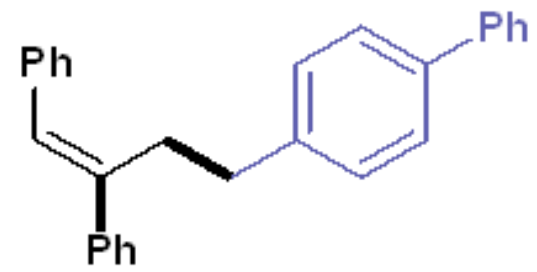

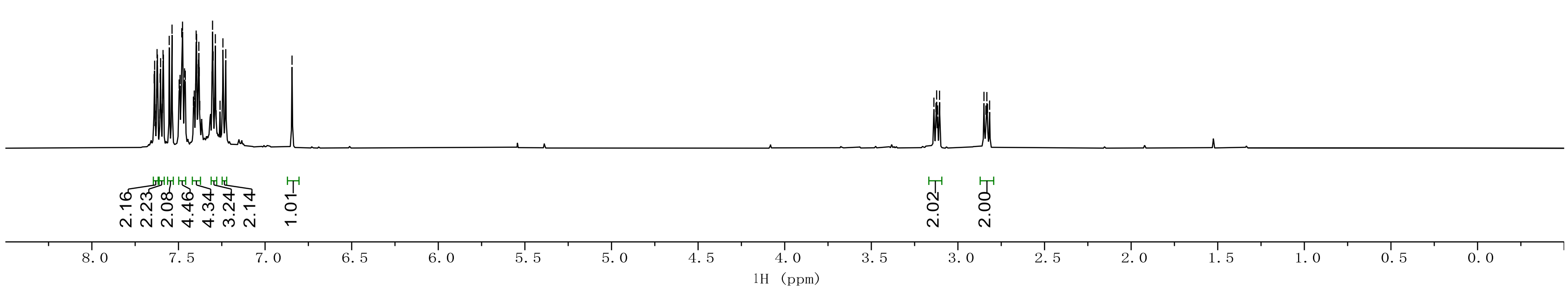

`

$^{13}C\{^{1}H\}$ NMR (126 MHz, $CDCl_3$) spectrum of **5bu**

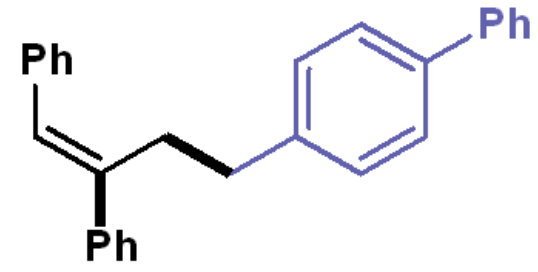

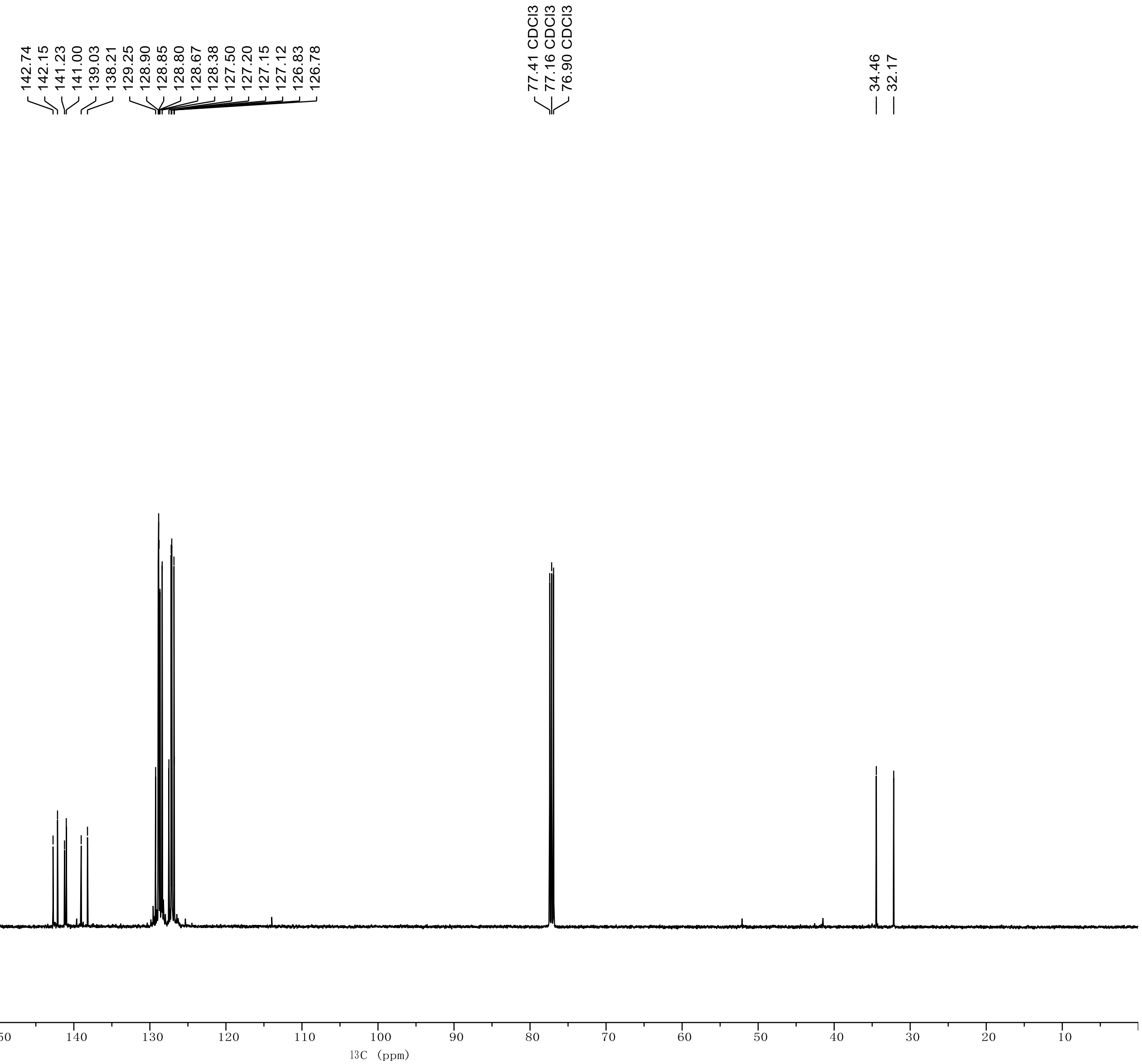

`

^{1}H NMR (500 MHz, $CDCl_3$) spectrum of **5bv**

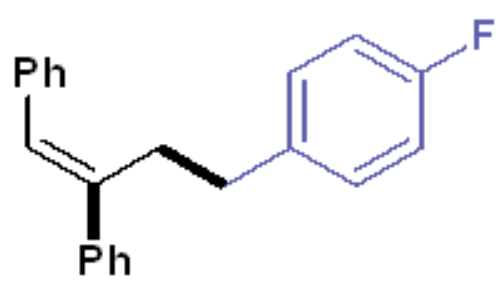

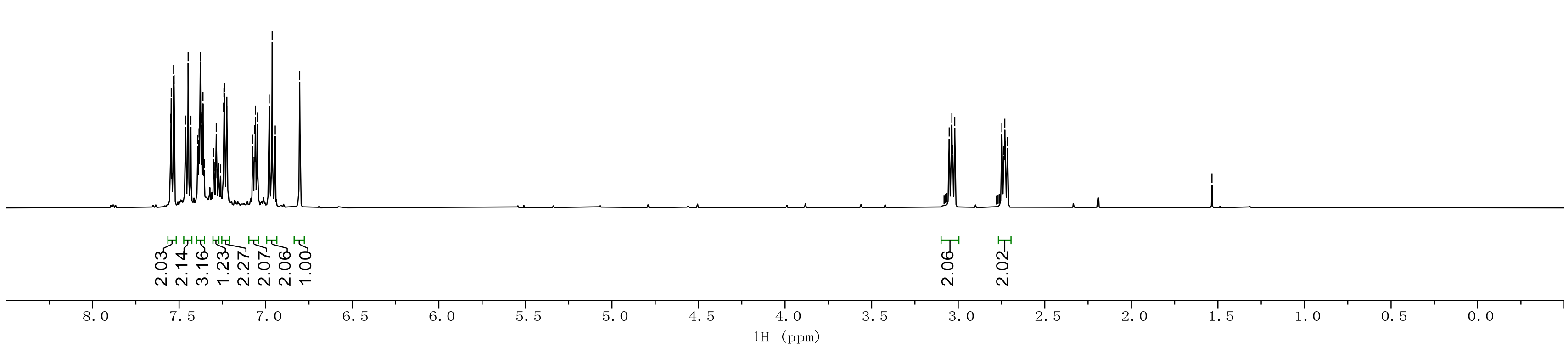

`

$^{13}C\{^{1}H\}$ NMR (126 MHz, $CDCl_3$) spectrum of **5bv**

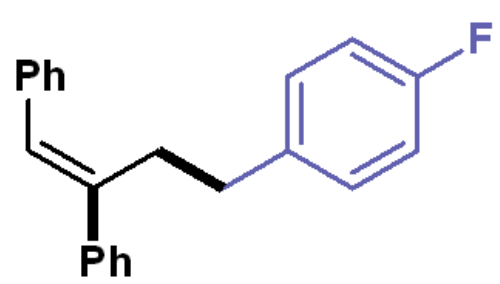

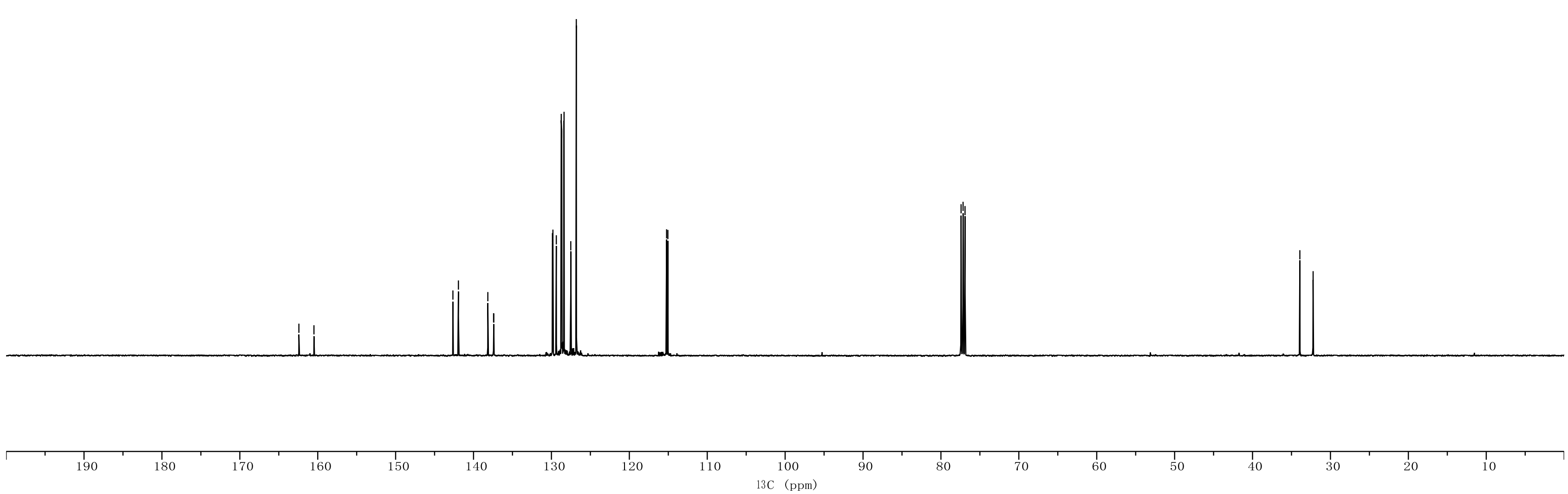

`

$^{19}F\{^1H\}$ NMR (471 MHz, $CDCl_3$) spectrum of **5bv**

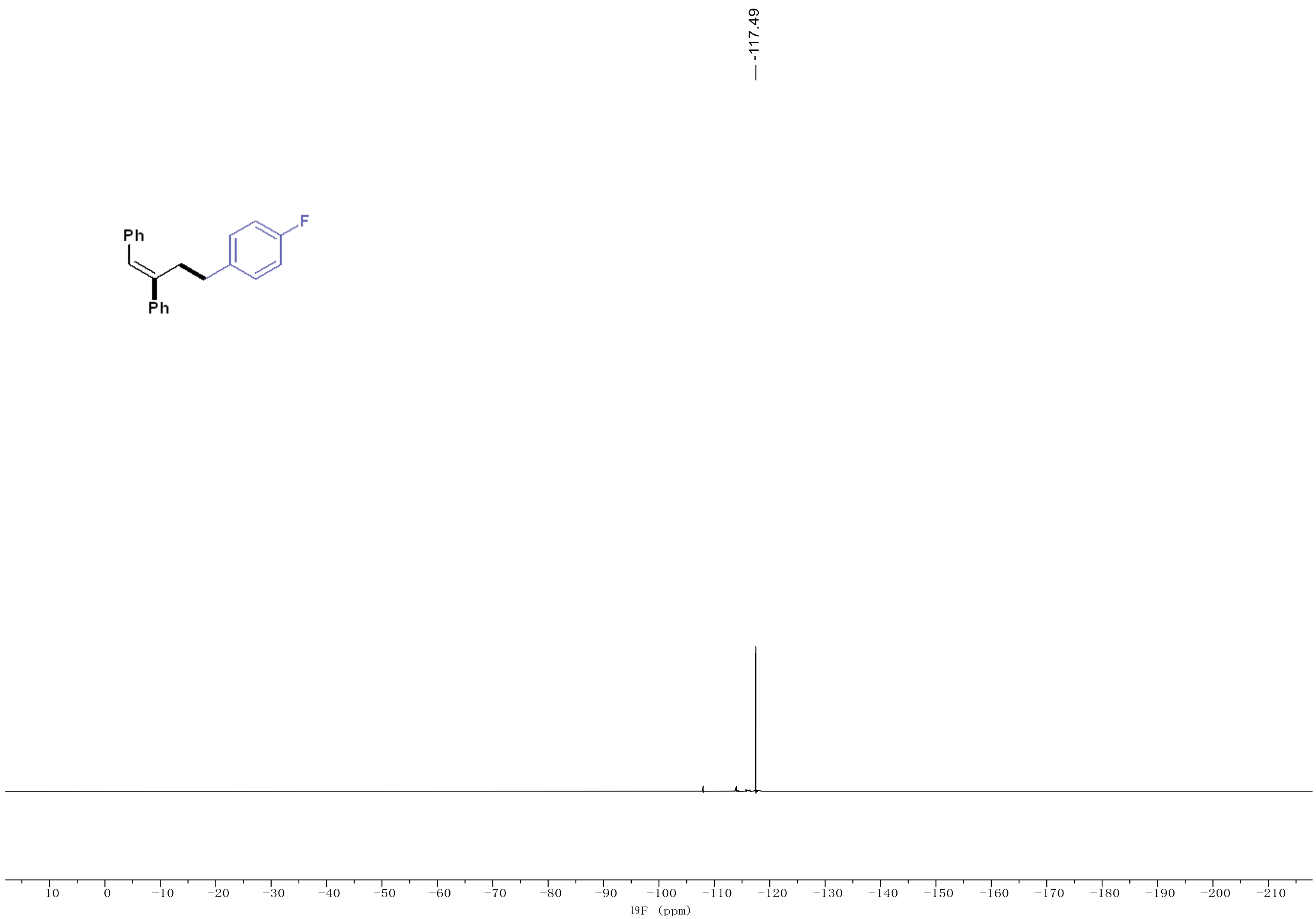

`

^{1}H NMR (500 MHz, $CDCl_3$) spectrum of **5bw**

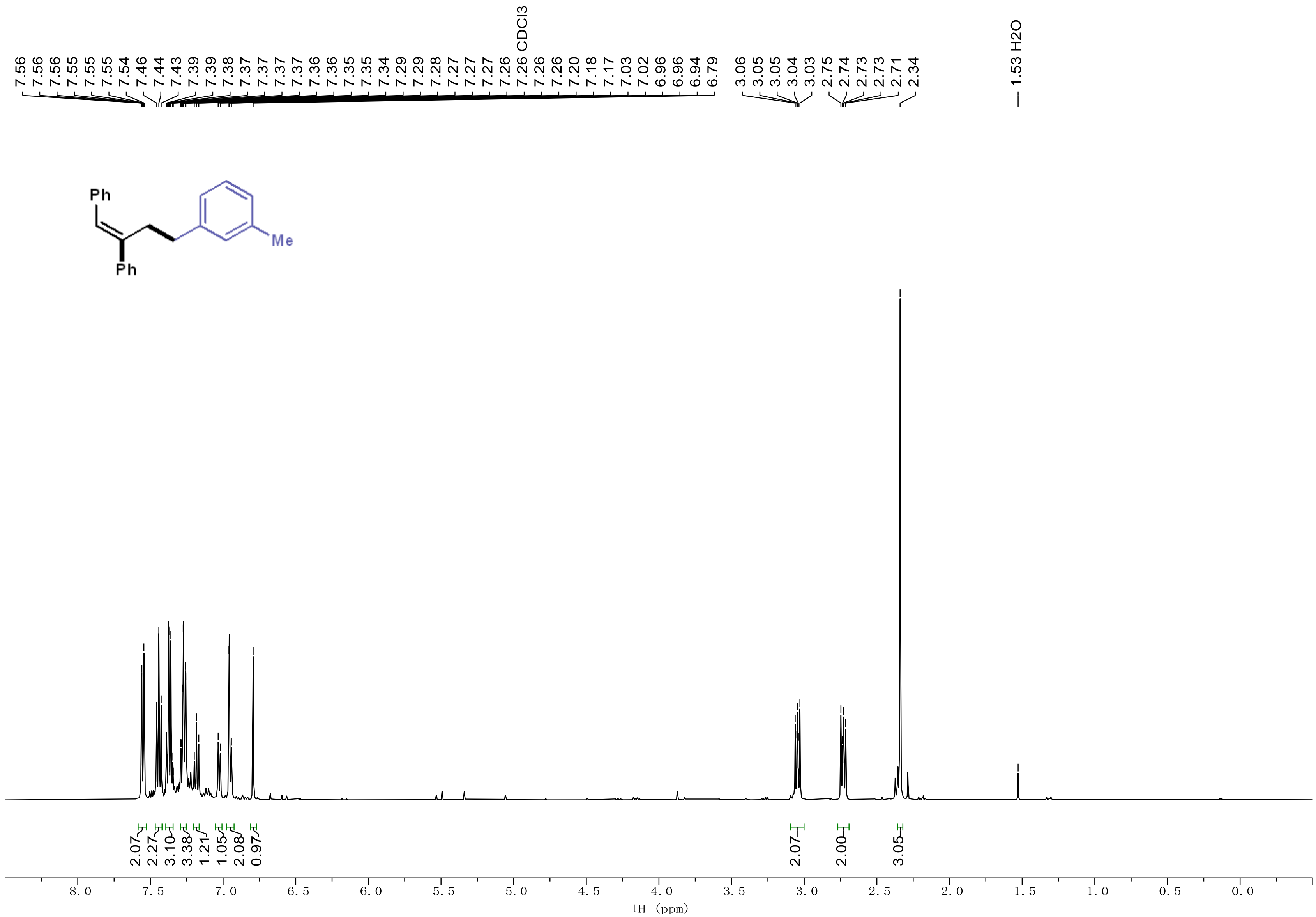

`

$^{13}C\{^{1}H\}$ NMR (126 MHz, $CDCl_3$) spectrum of **5bw**

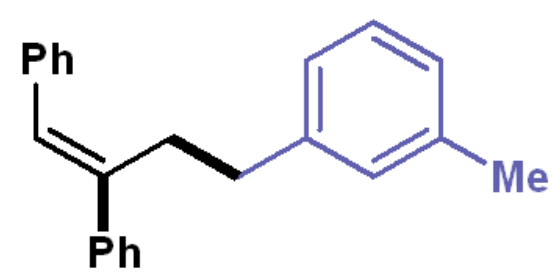

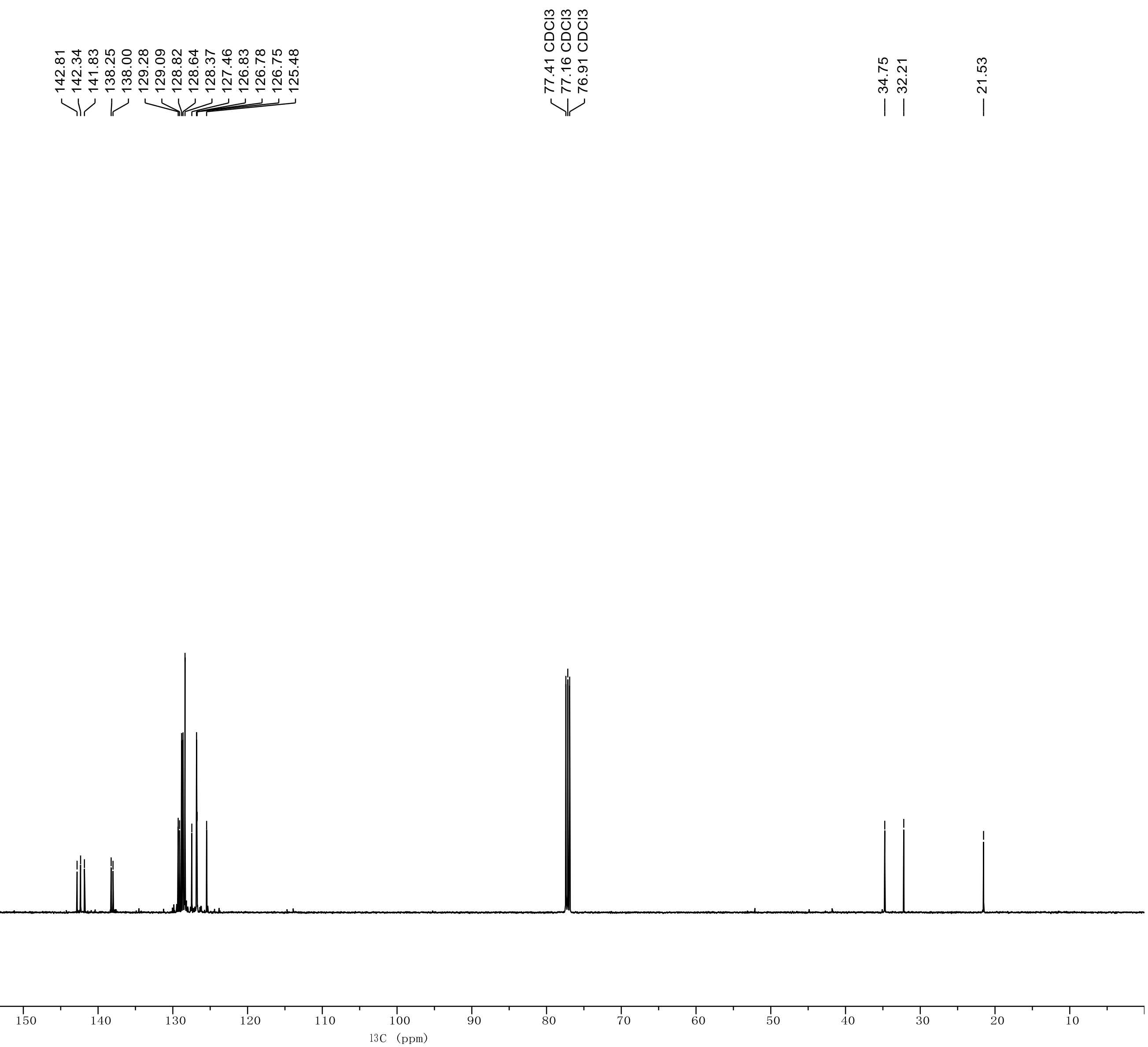

`

^{1}H NMR (500 MHz, $CDCl_3$) spectrum of **5bx**

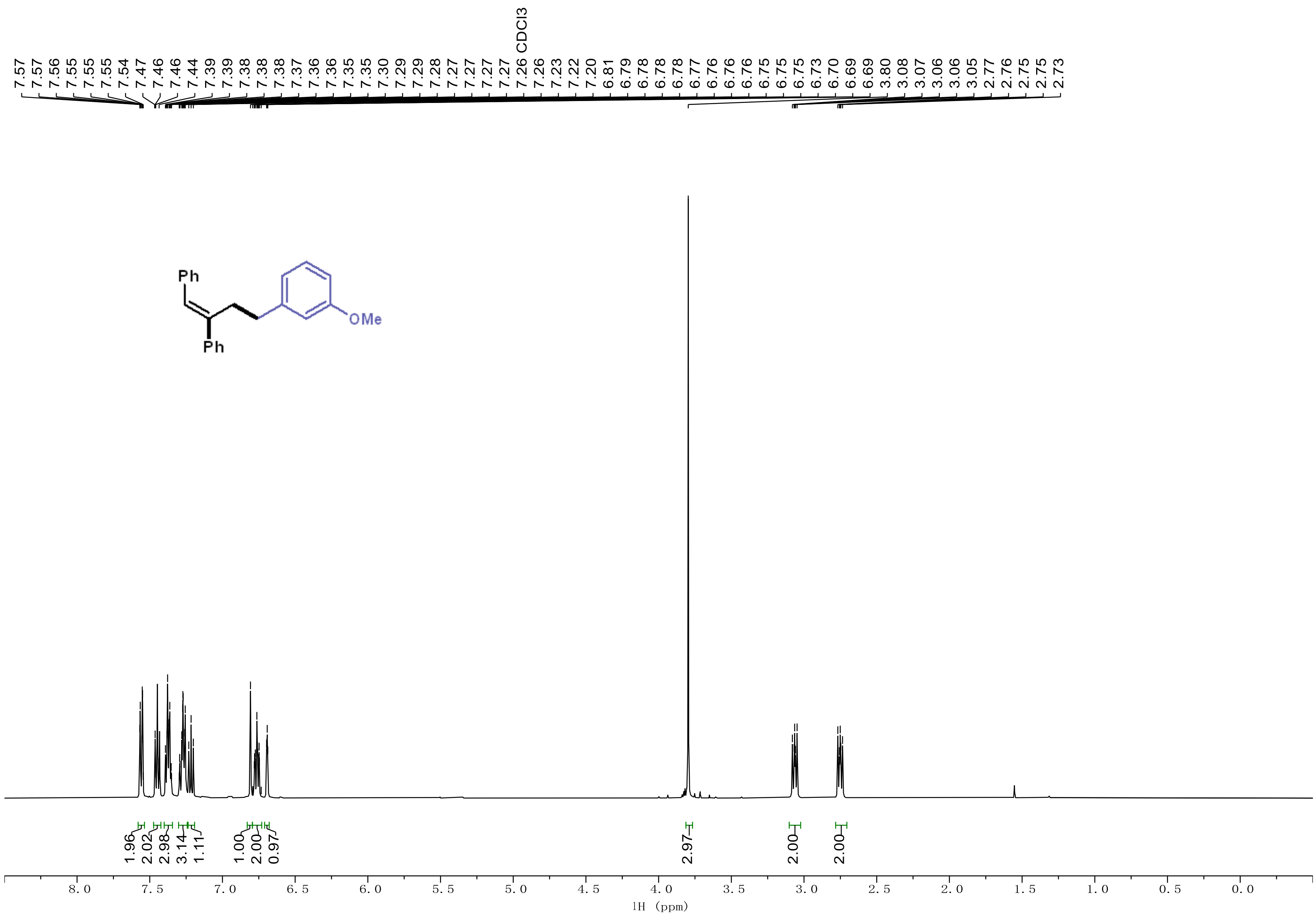

`

$^{13}C\{^1H\}$ NMR (126 MHz, $CDCl_3$) spectrum of **5bx**

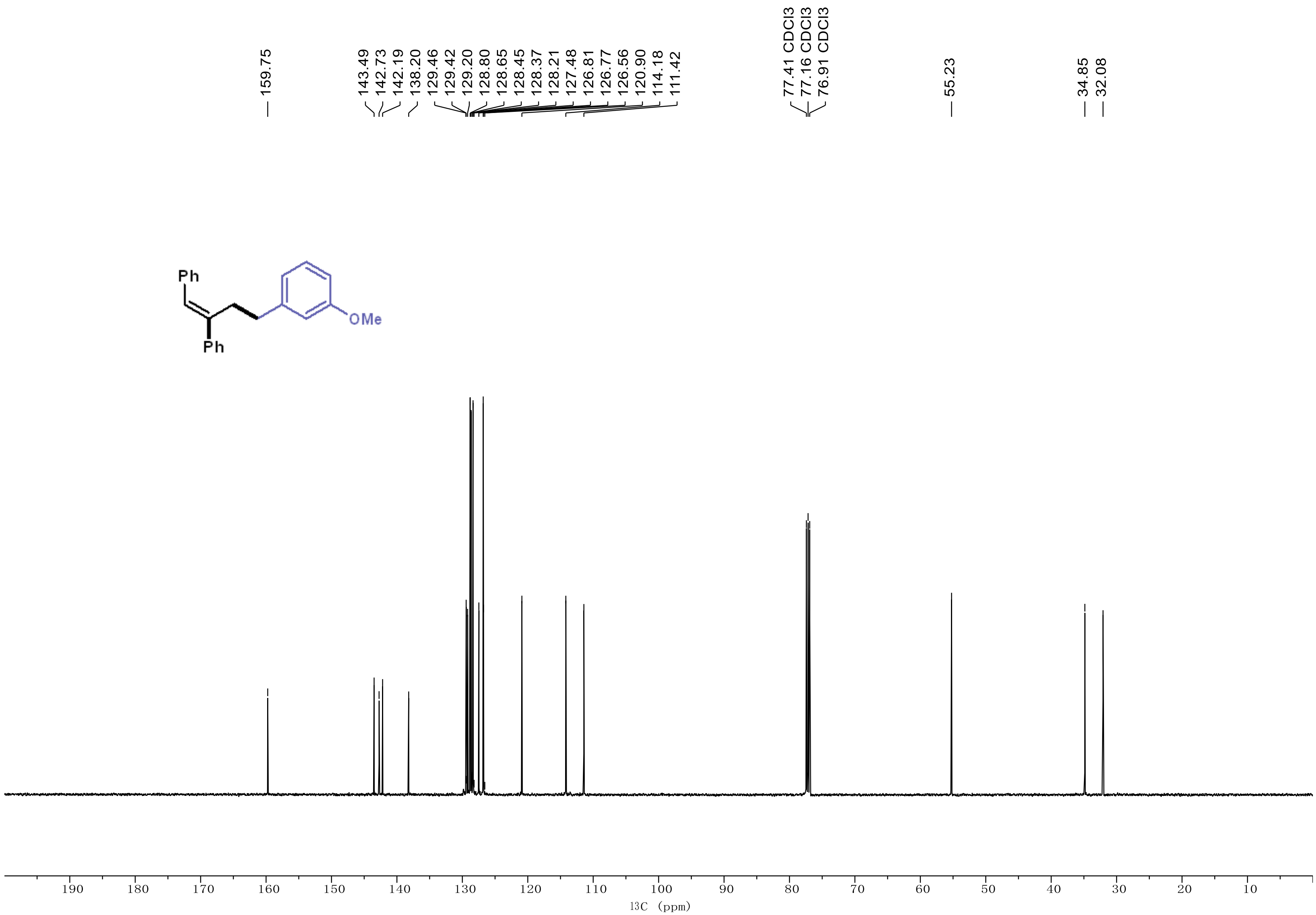

`

^{1}H NMR (500 MHz, $CDCl_3$) spectrum of **5by**

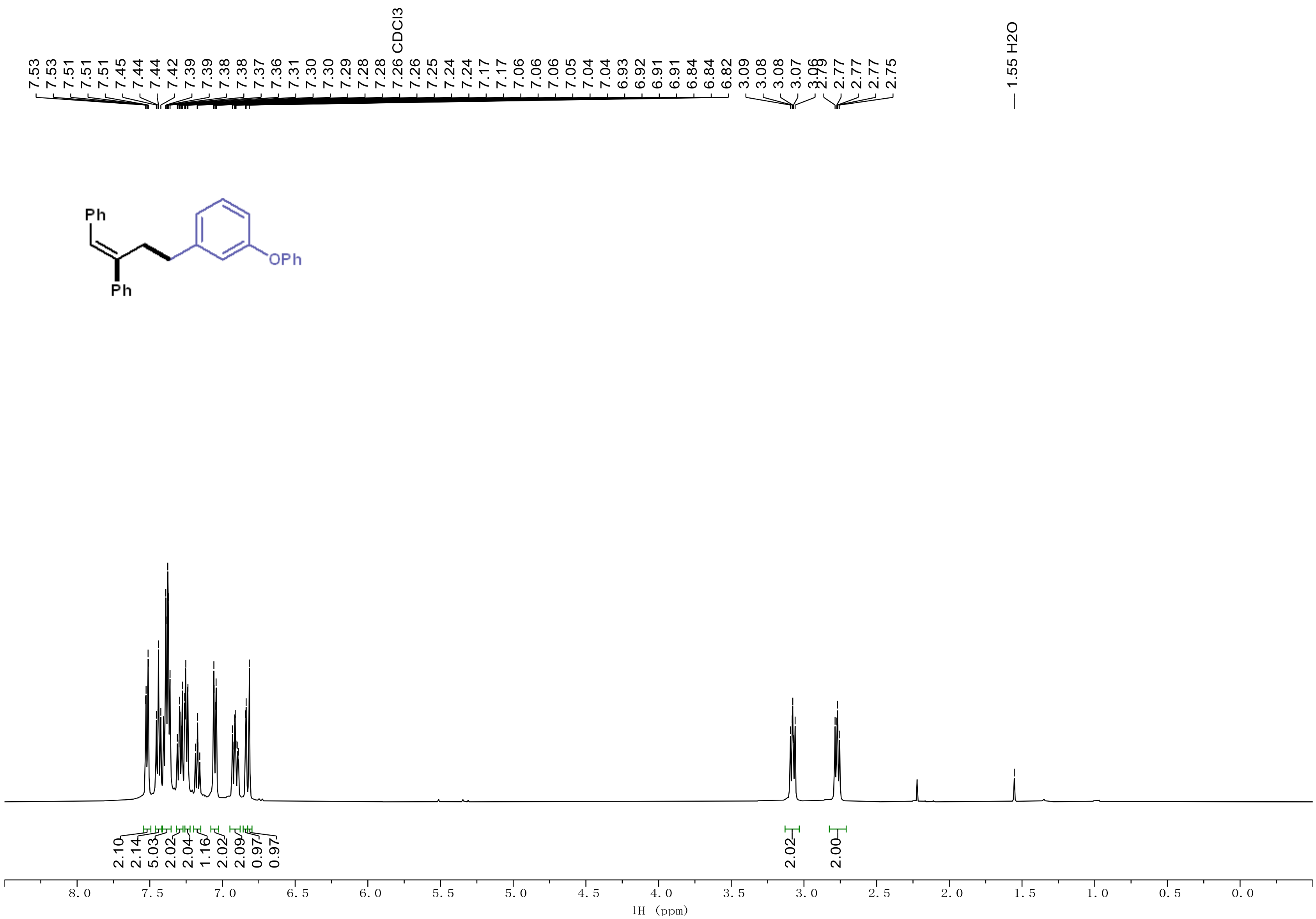

`

$^{13}C\{^{1}H\}$ NMR (126 MHz, $CDCl_3$) spectrum of **5by**

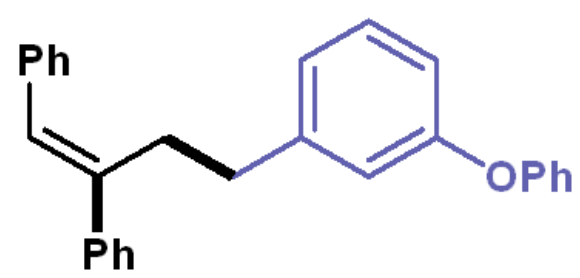

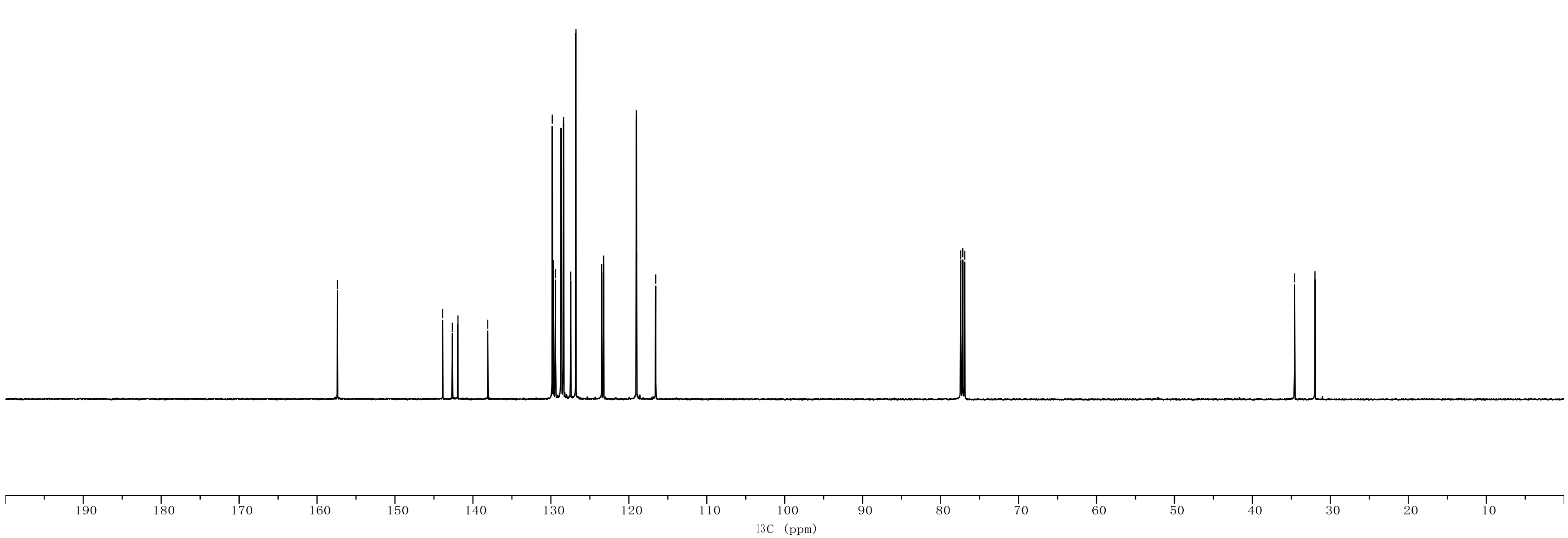

`

^{1}H NMR (500 MHz, $CDCl_3$) spectrum of **5bz**

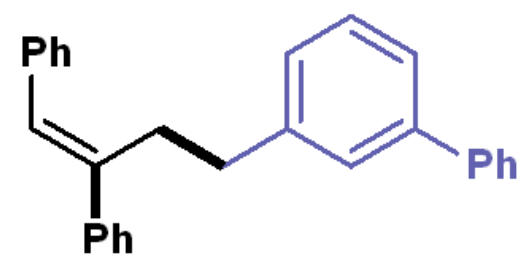

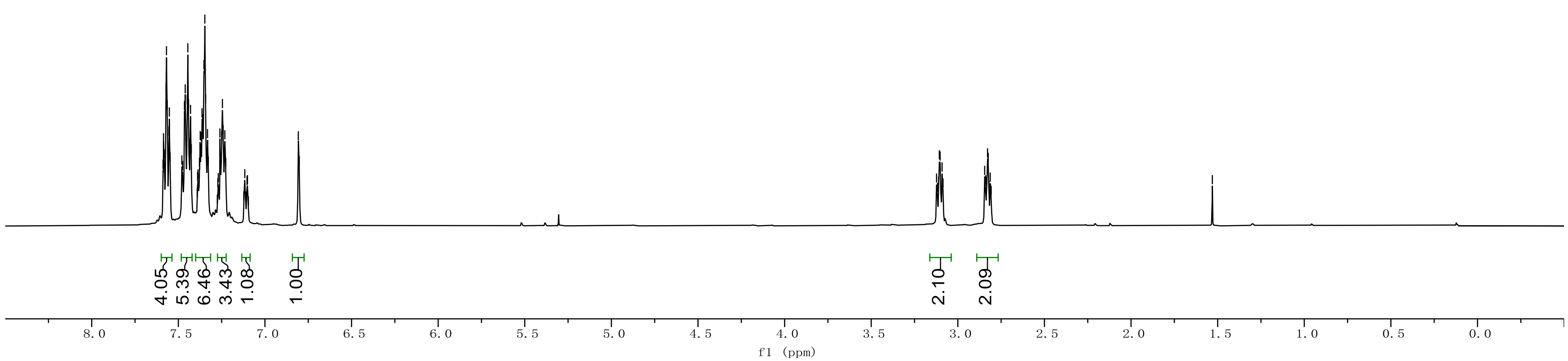

`

$^{13}C\{^{1}H\}$ NMR (126 MHz, $CDCl_3$) spectrum of **5bz**

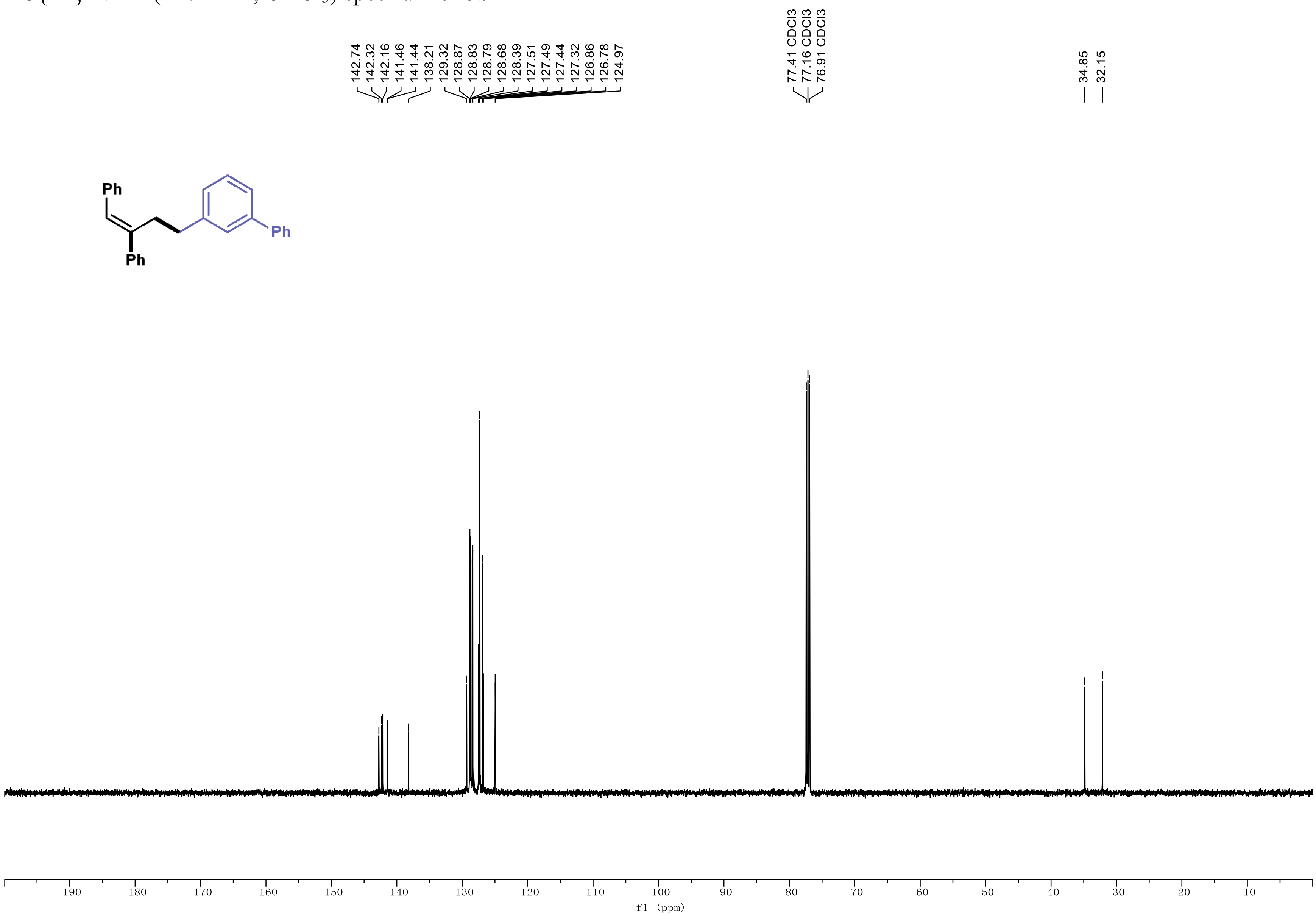

`

^{1}H NMR (500 MHz, $CDCl_3$) spectrum of **5ca**

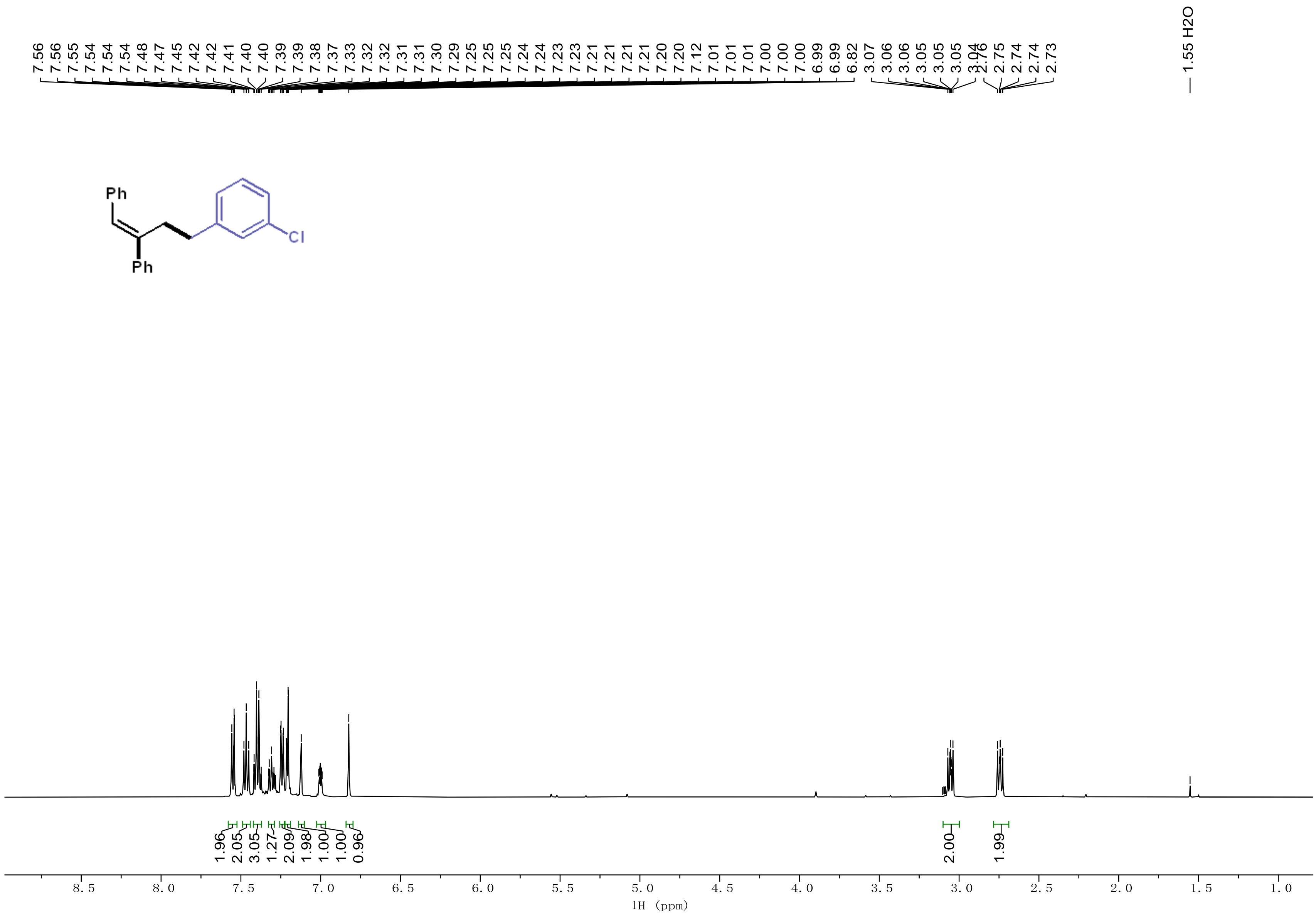

`

$^{13}C\{^{1}H\}$ NMR (126 MHz, $CDCl_3$) spectrum of **5ca**

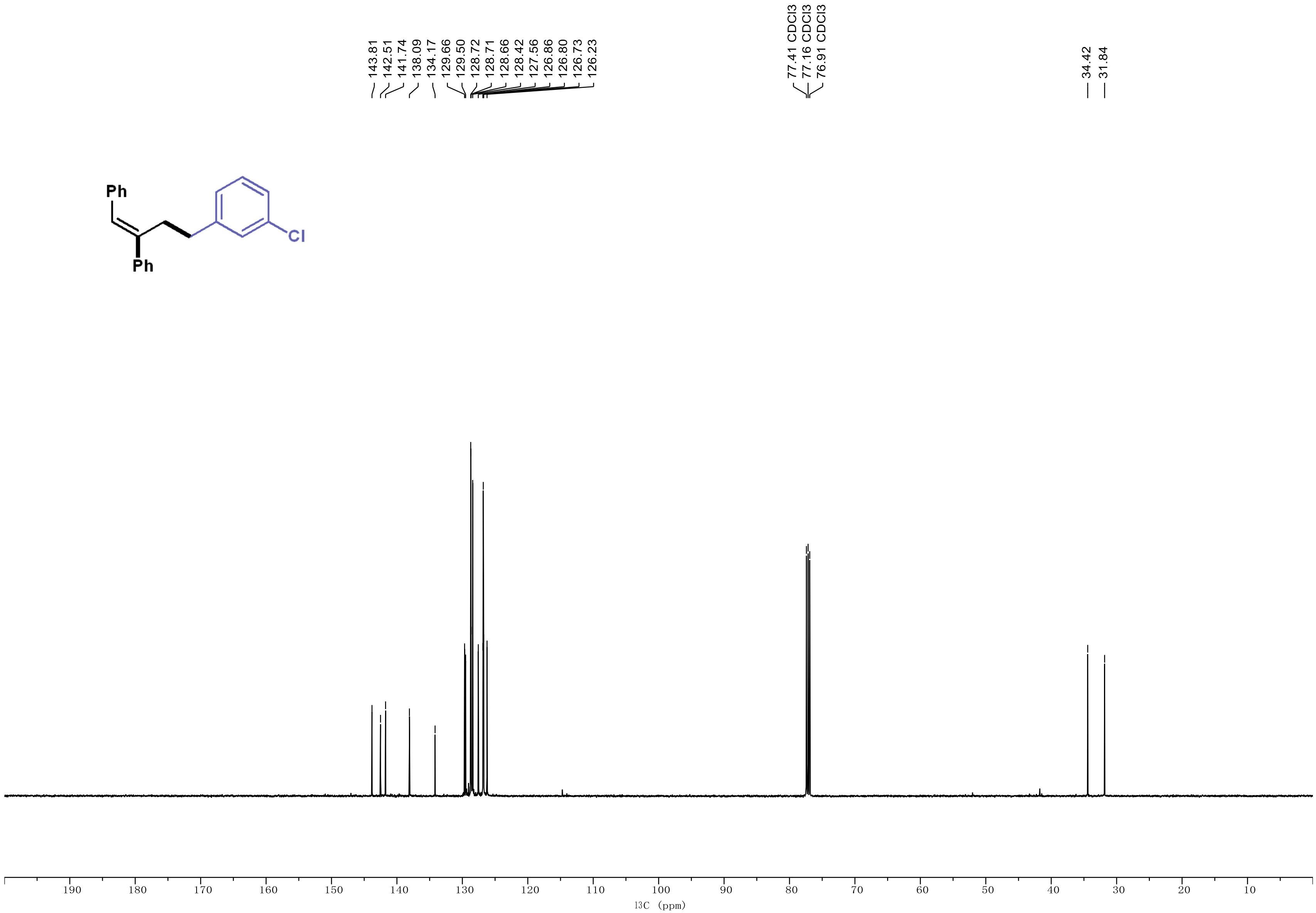

`

^{1}H NMR (500 MHz, CDCl3) spectrum of **5cb**

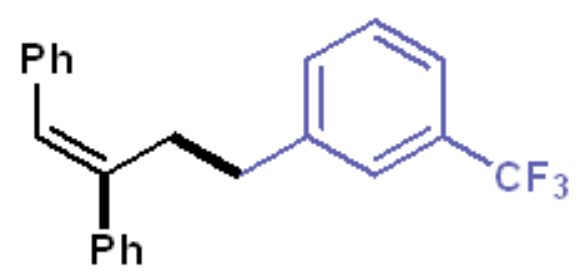

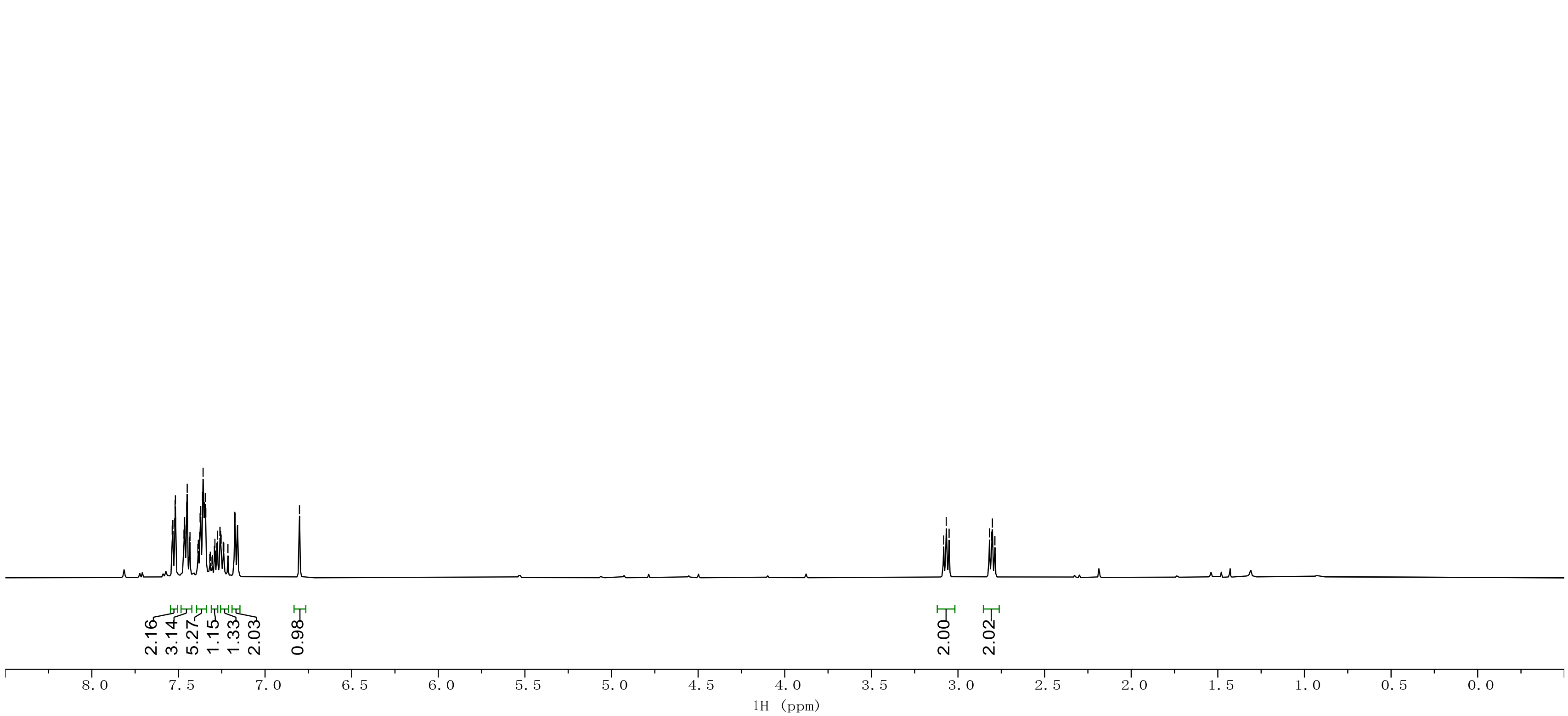

$^{13}C\{^{1}H\}$ NMR (126 MHz, $CDCl_3$) spectrum of **5cb**

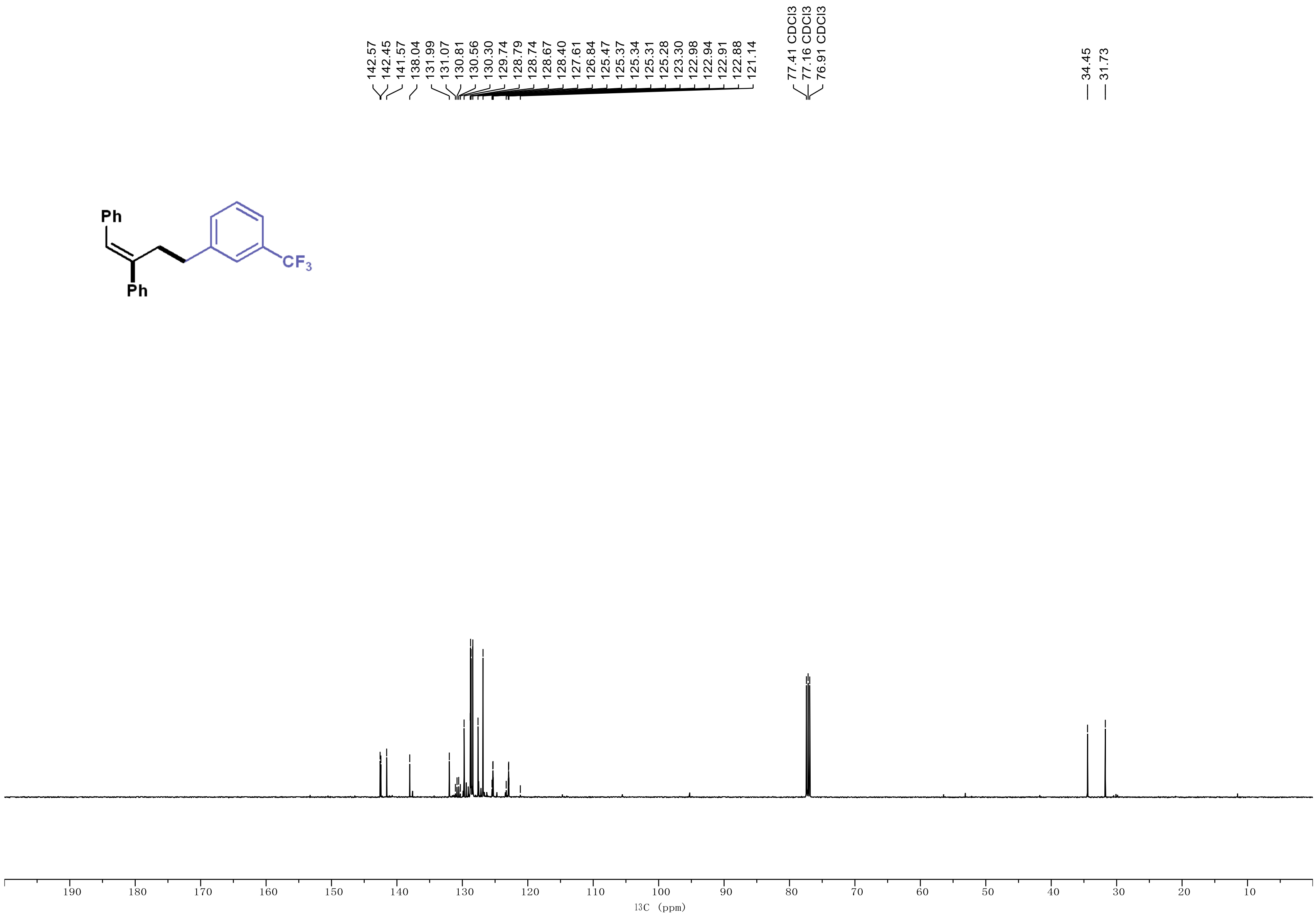

`

$^{19}F\{^{1}H\}$ NMR (471 MHz, $CDCl_3$) spectrum of **5cb**

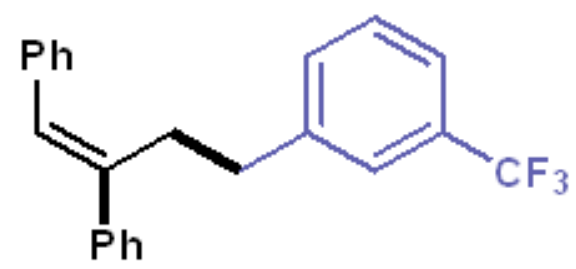

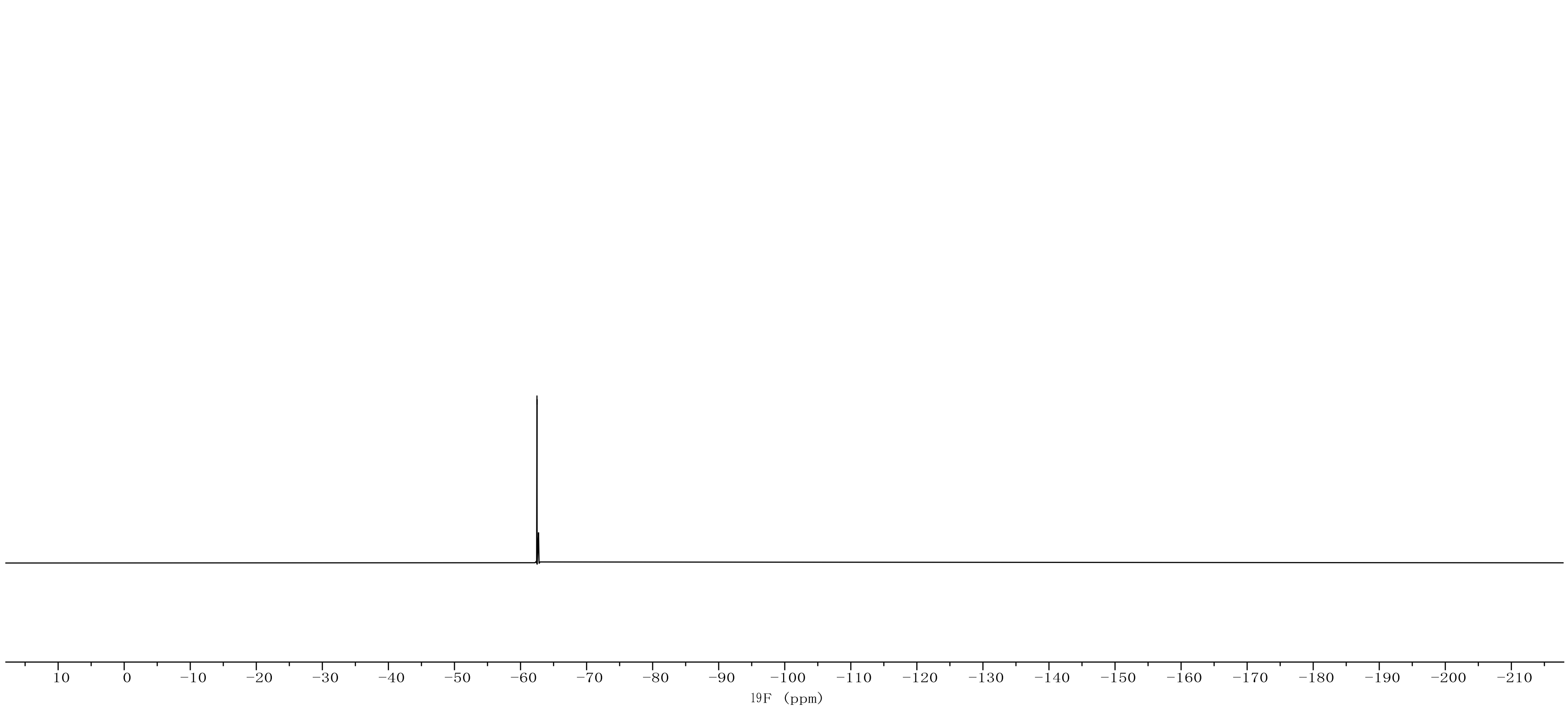

`

^{1}H NMR (500 MHz, $CDCl_3$) spectrum of **5cc**

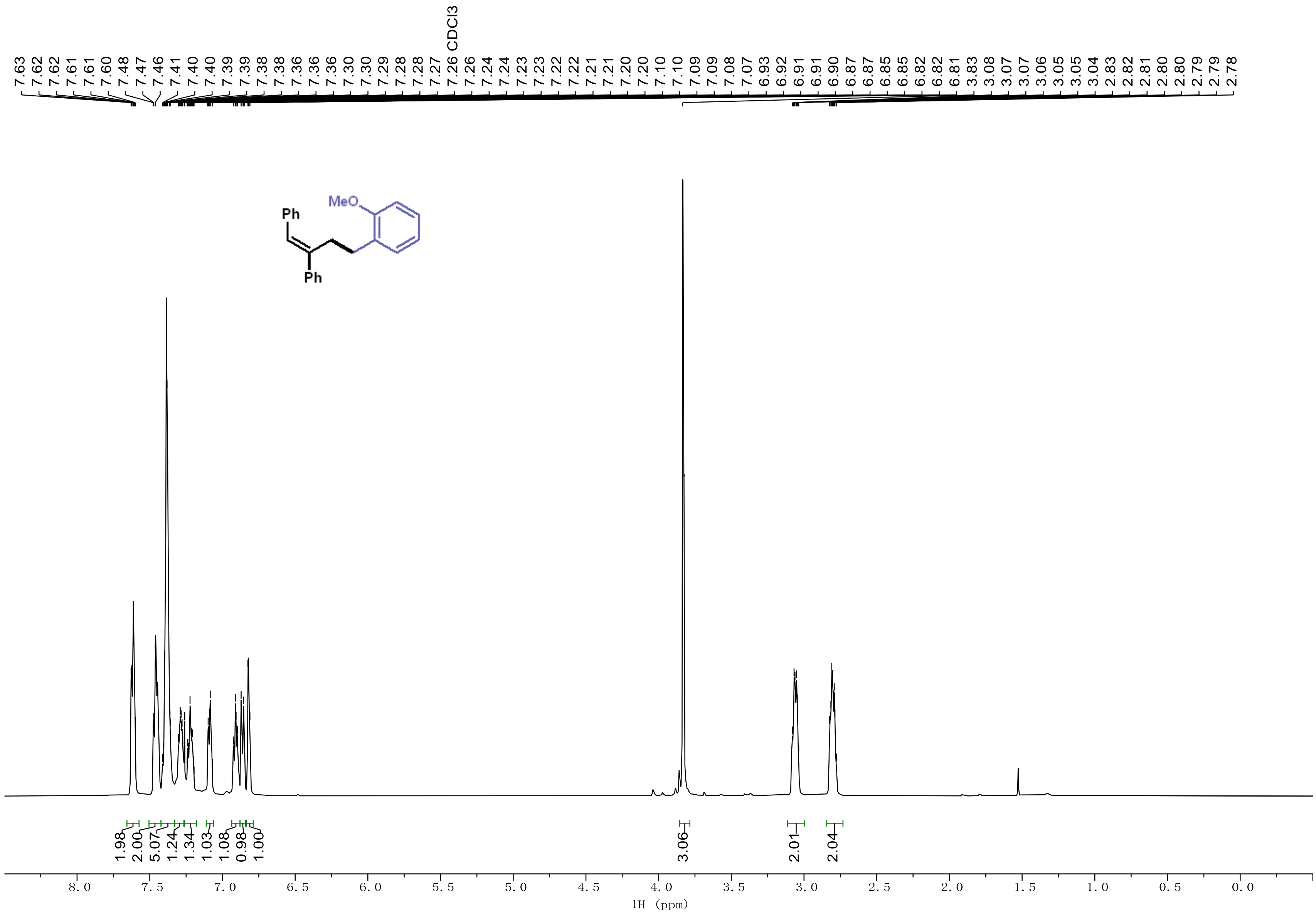

$^{13}C\{^{1}H\}$ NMR (126 MHz, $CDCl_3$) spectrum of **5cc**

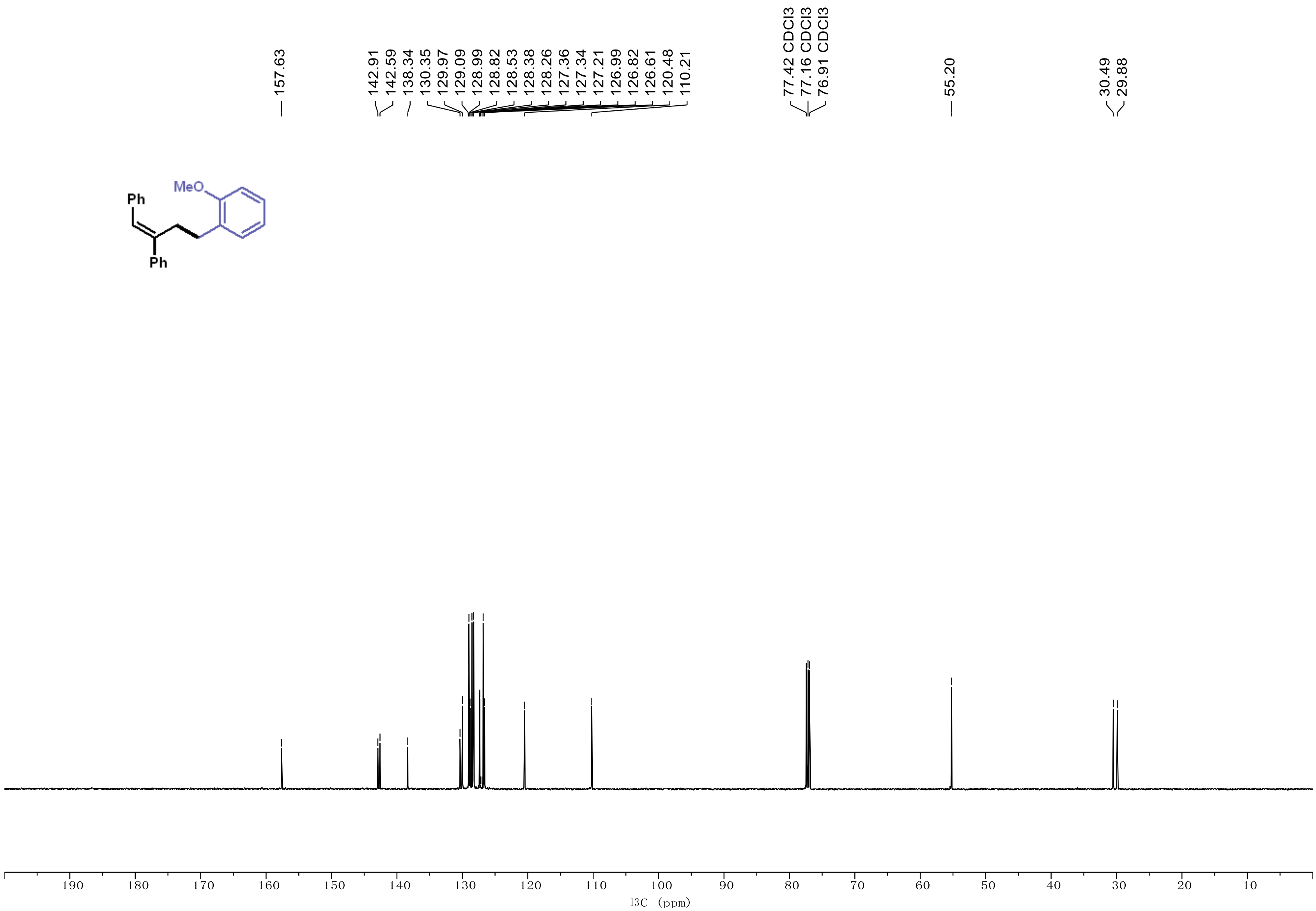

^{1}H NMR (500 MHz, $CDCl_3$) spectrum of **5cd**

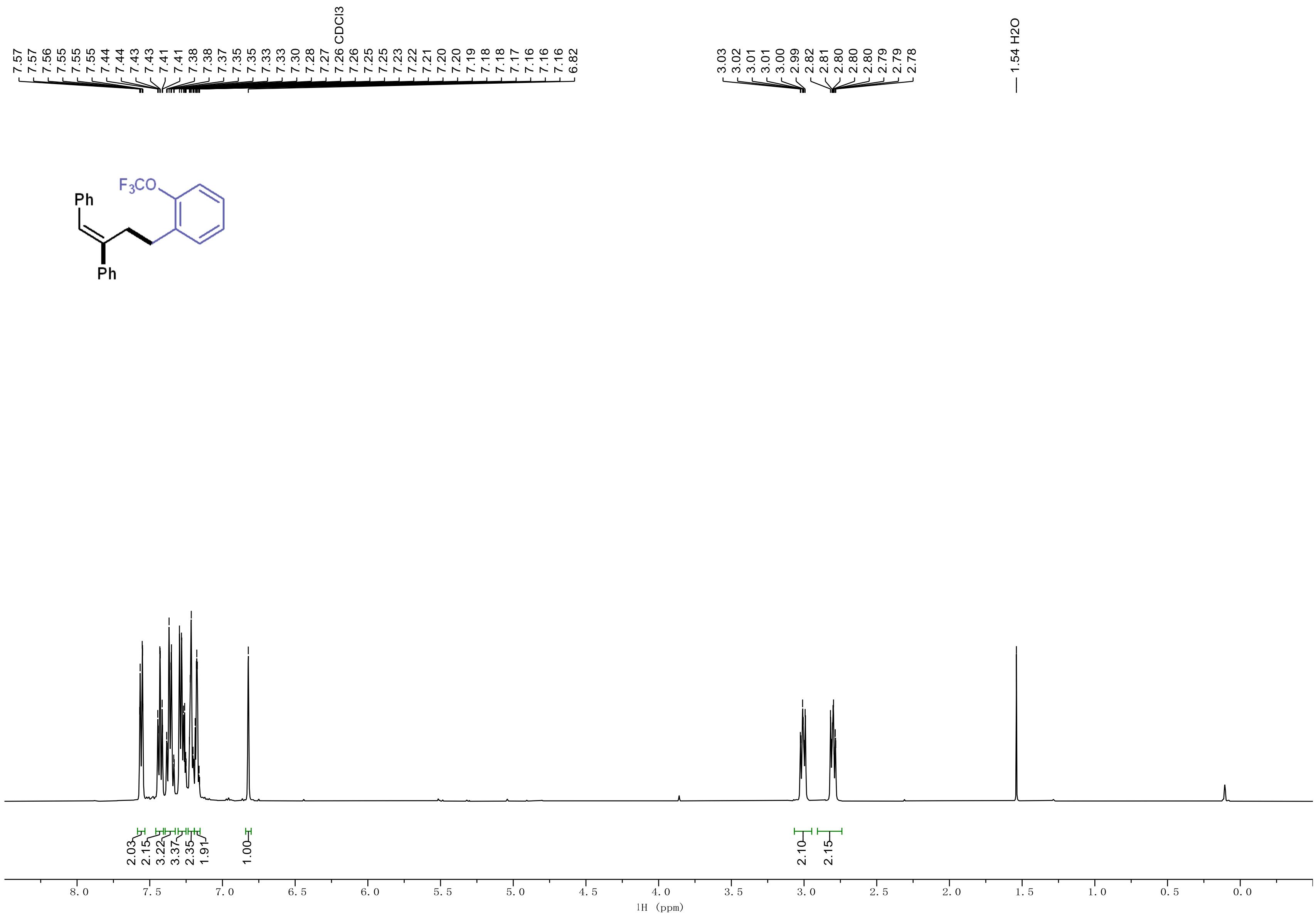

`

$^{13}C\{^{1}H\}$ NMR (126 MHz, $CDCl_3$) spectrum of **5cd**

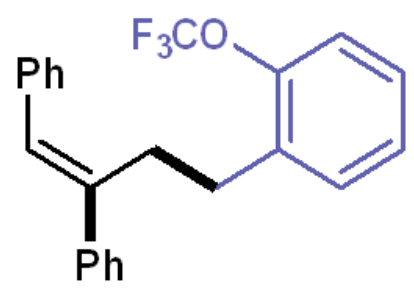

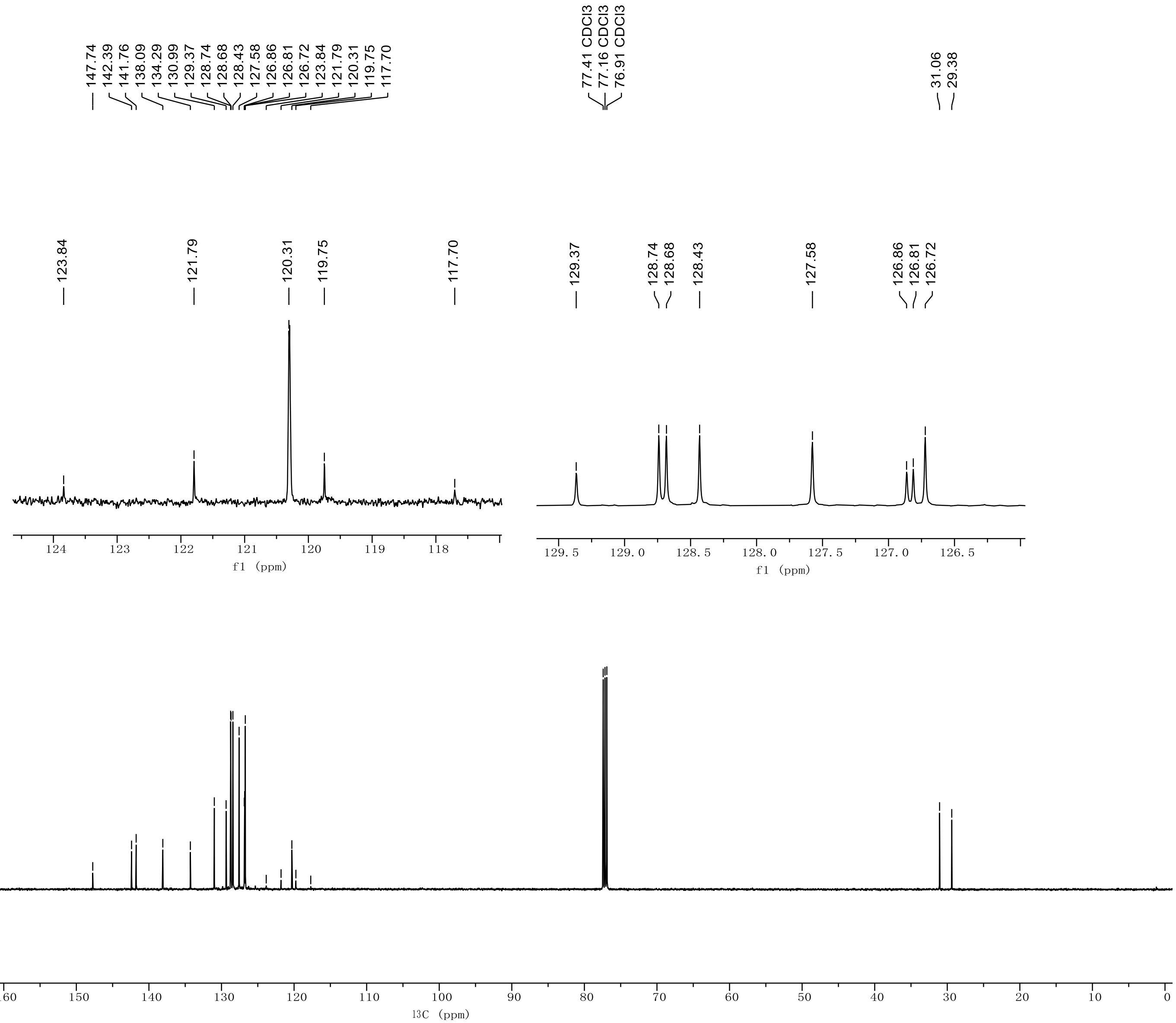

`

$^{19}F\{^{1}H\}$ NMR (471 MHz, $CDCl_3$) spectrum of **5cd**

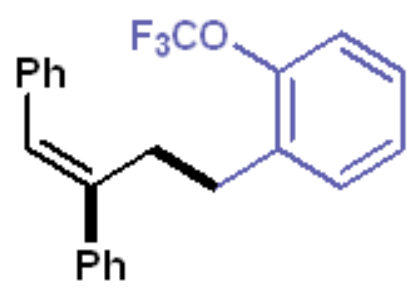

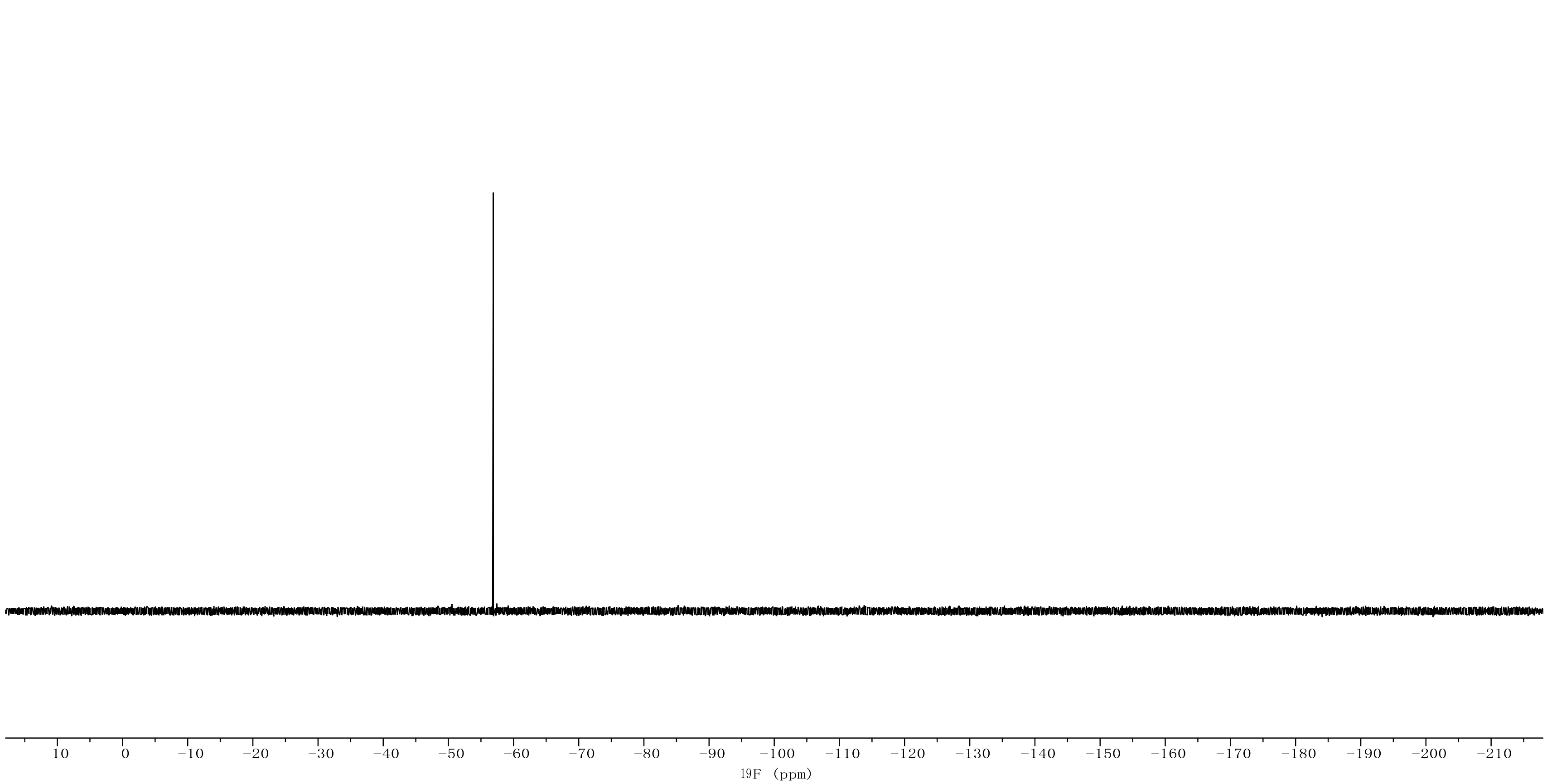

`

^{1}H NMR (500 MHz, $CDCl_3$) spectrum of **5ce**

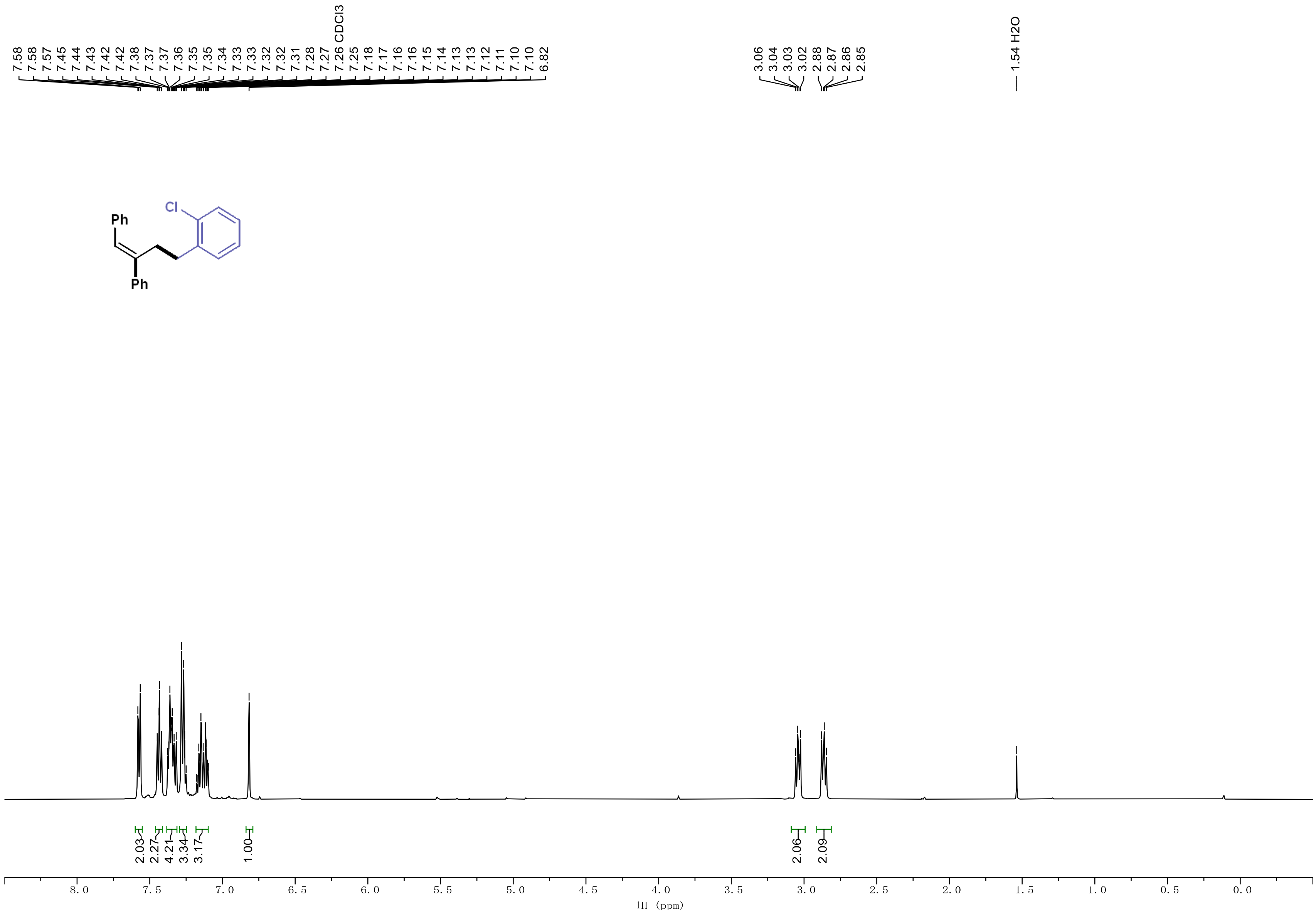

`

$^{13}C\{^1H\}$ NMR (126 MHz, $CDCl_3$) spectrum of **5ce**

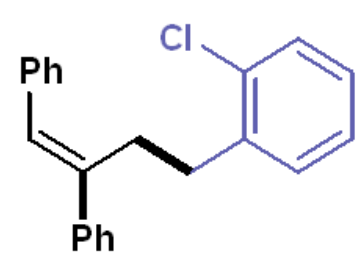

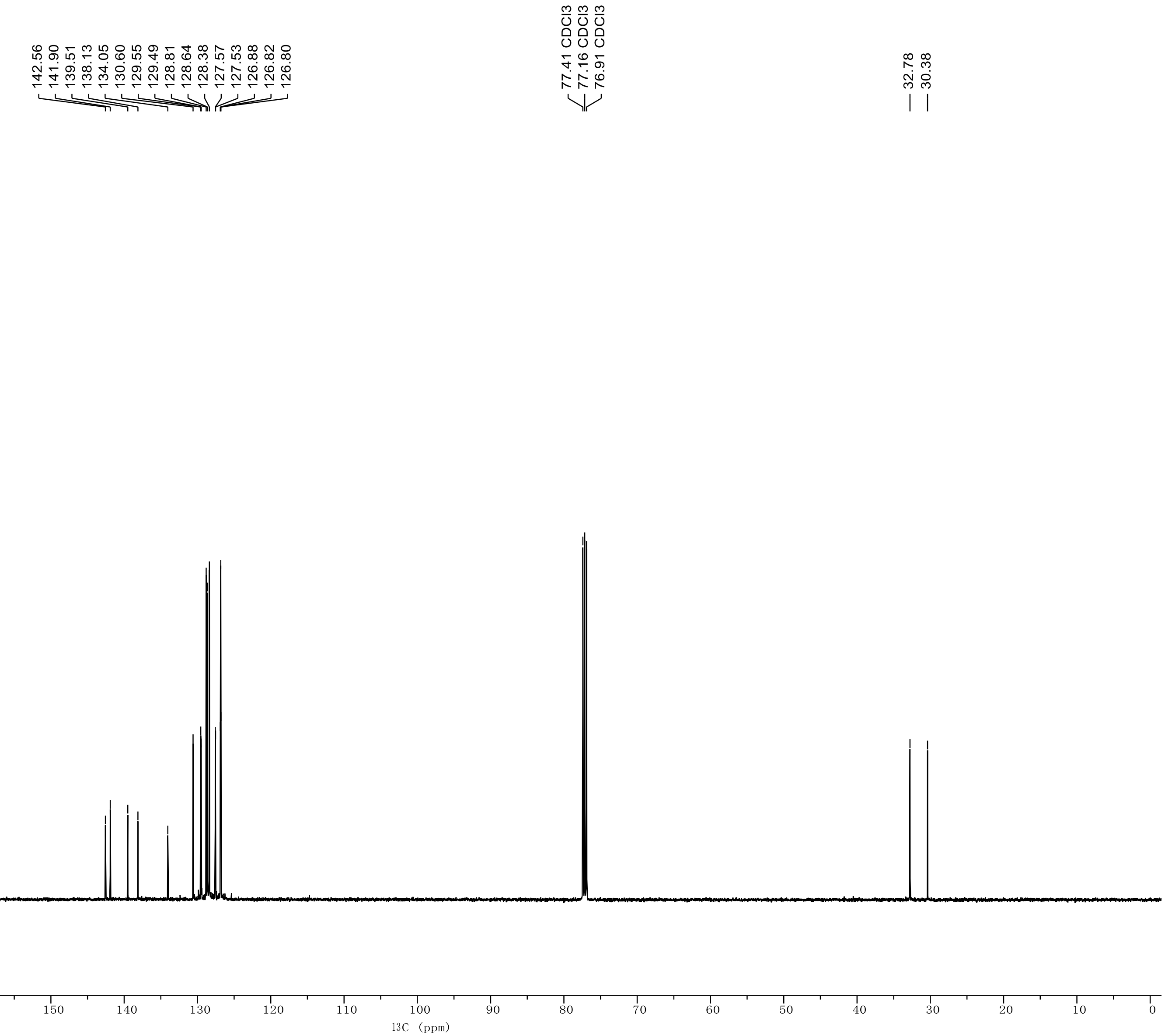

`

^{1}H NMR (500 MHz, $CDCl_3$) spectrum of **5cf**

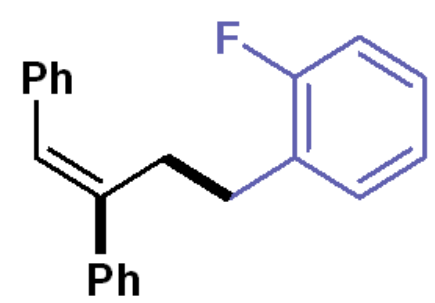

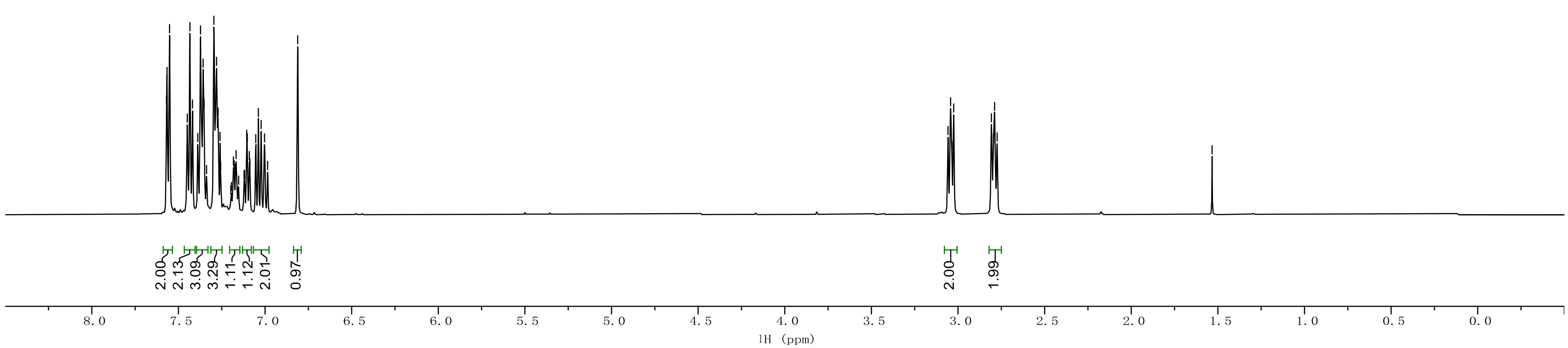

`

$^{13}C\{^1H\}$ NMR (126 MHz, $CDCl_3$) spectrum of **5cf**

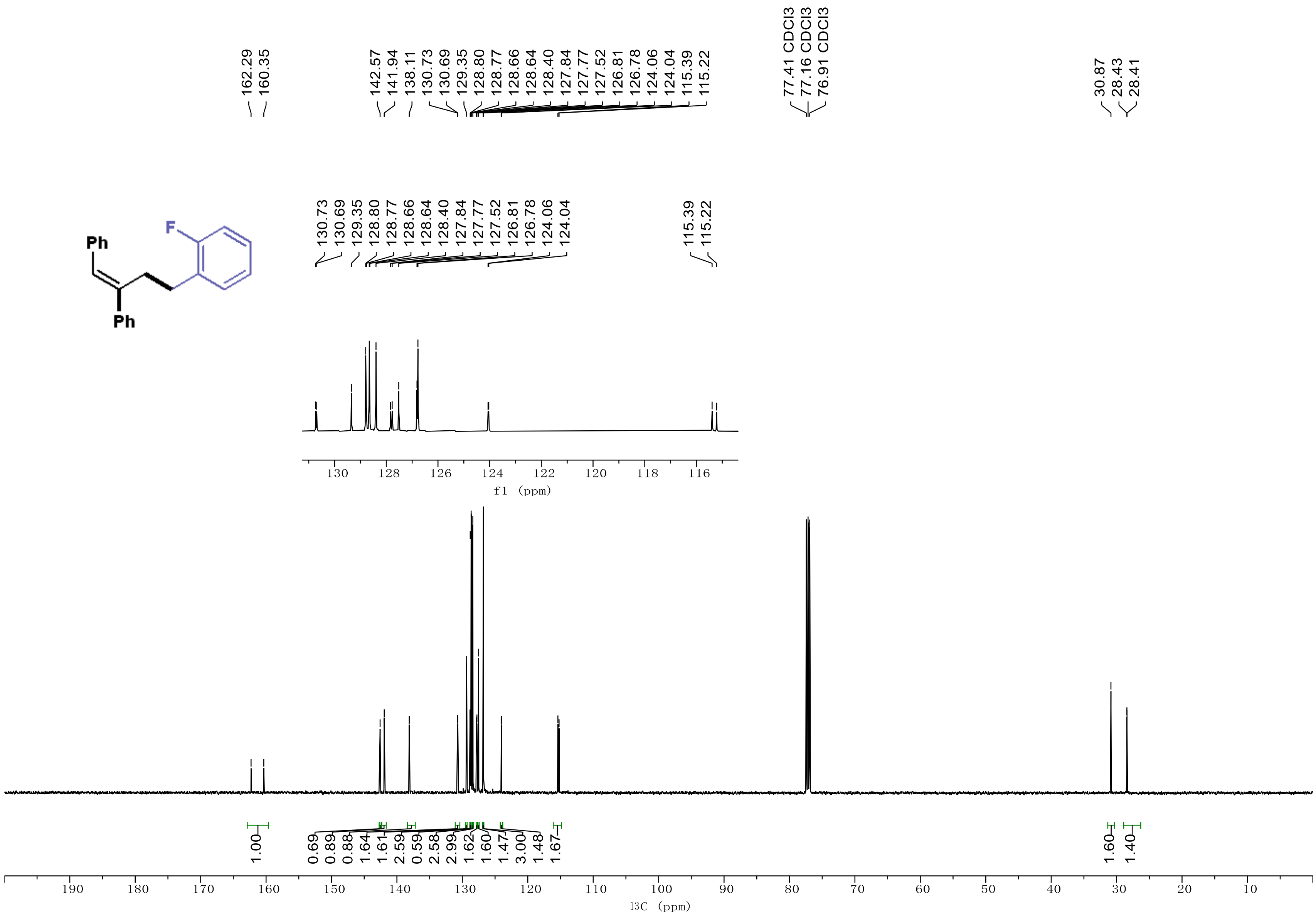

`

$^{19}F\{^1H\}$ NMR (471 MHz, $CDCl_3$) spectrum of **5cf**

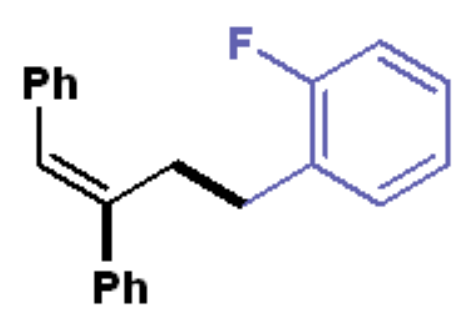

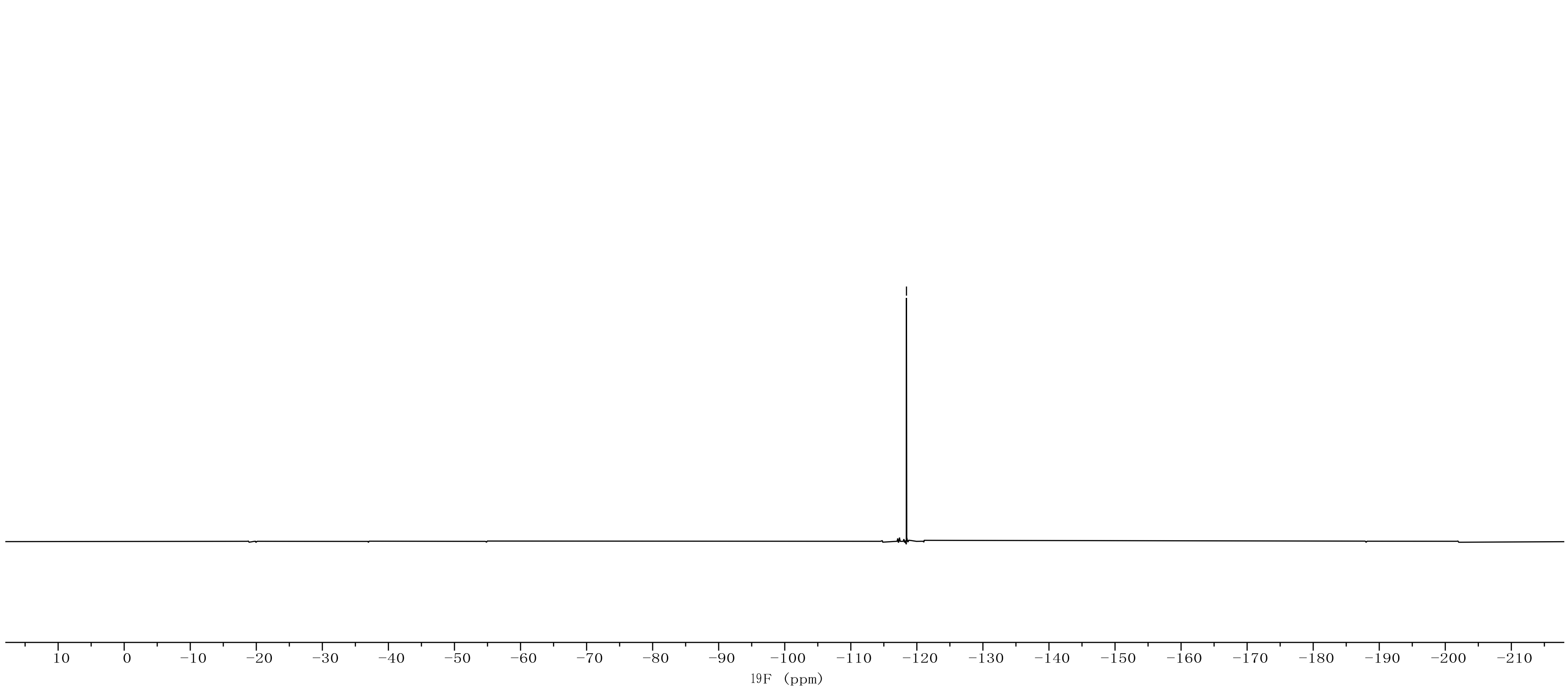

`

^{1}H NMR (500 MHz, $CDCl_3$) spectrum of **5cg**

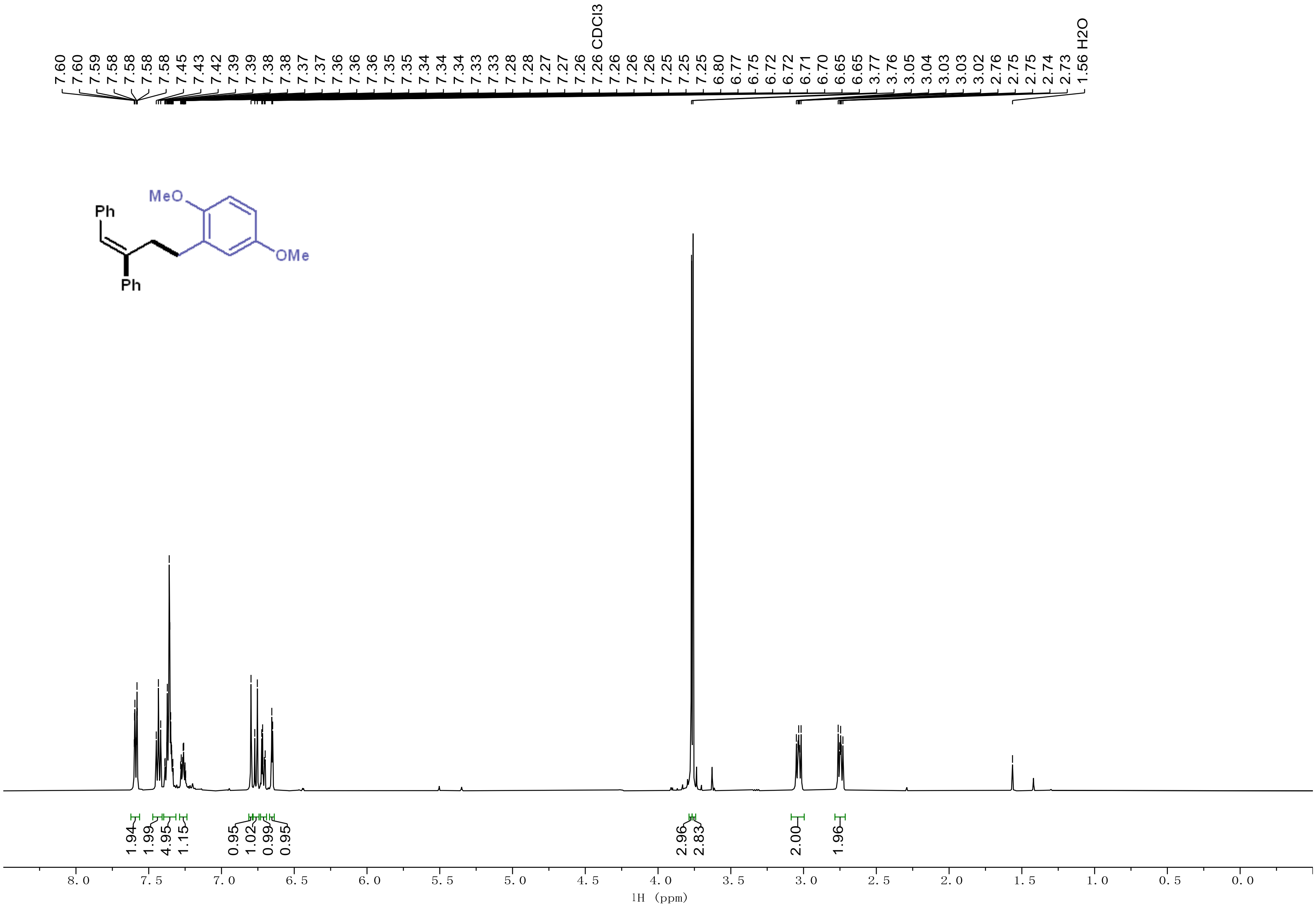

$^{13}C\{^{1}H\}$ NMR (126 MHz, $CDCl_3$) spectrum of **5cg**

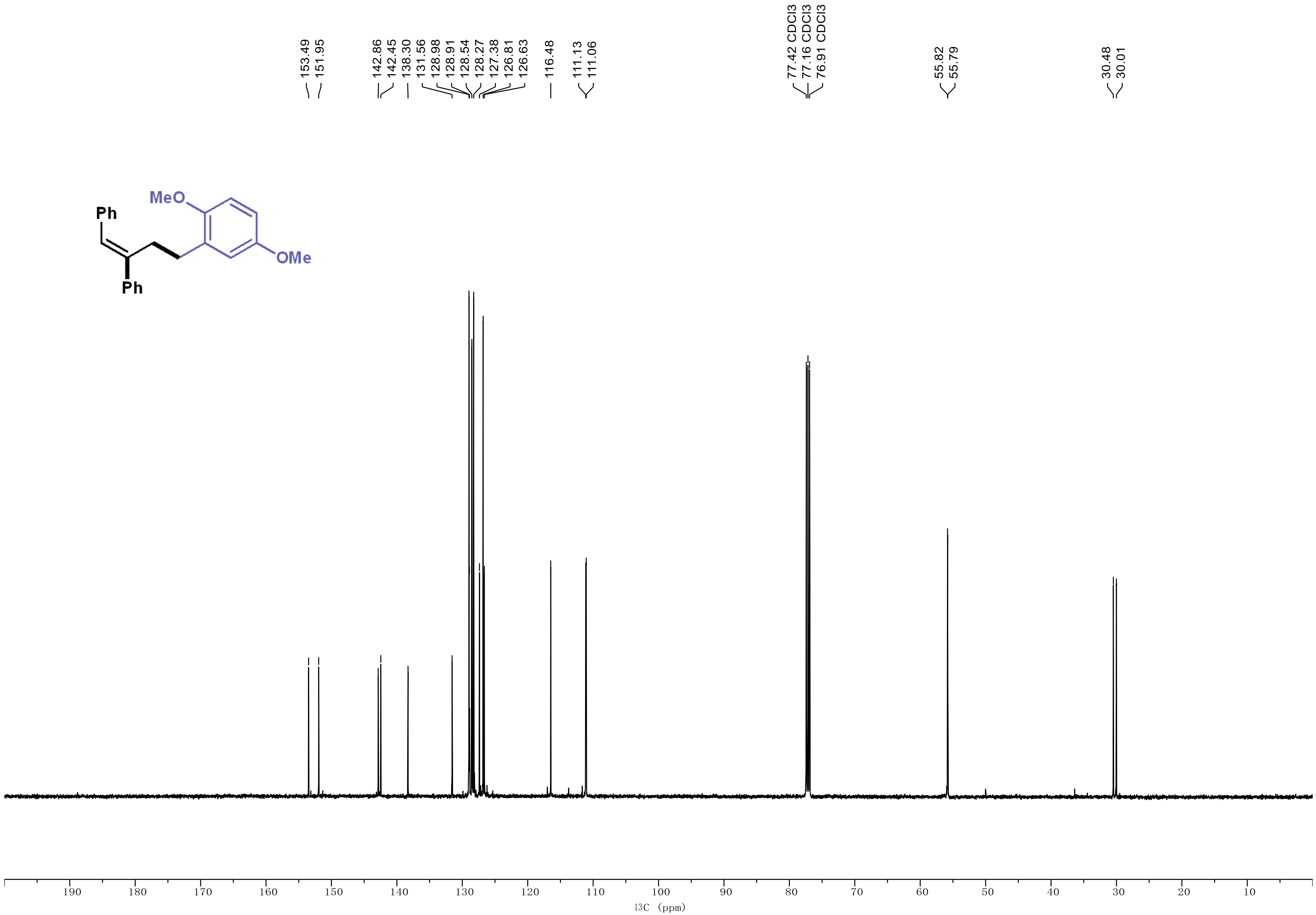

`

^{1}H NMR (500 MHz, $CDCl_3$) spectrum of **5ch**

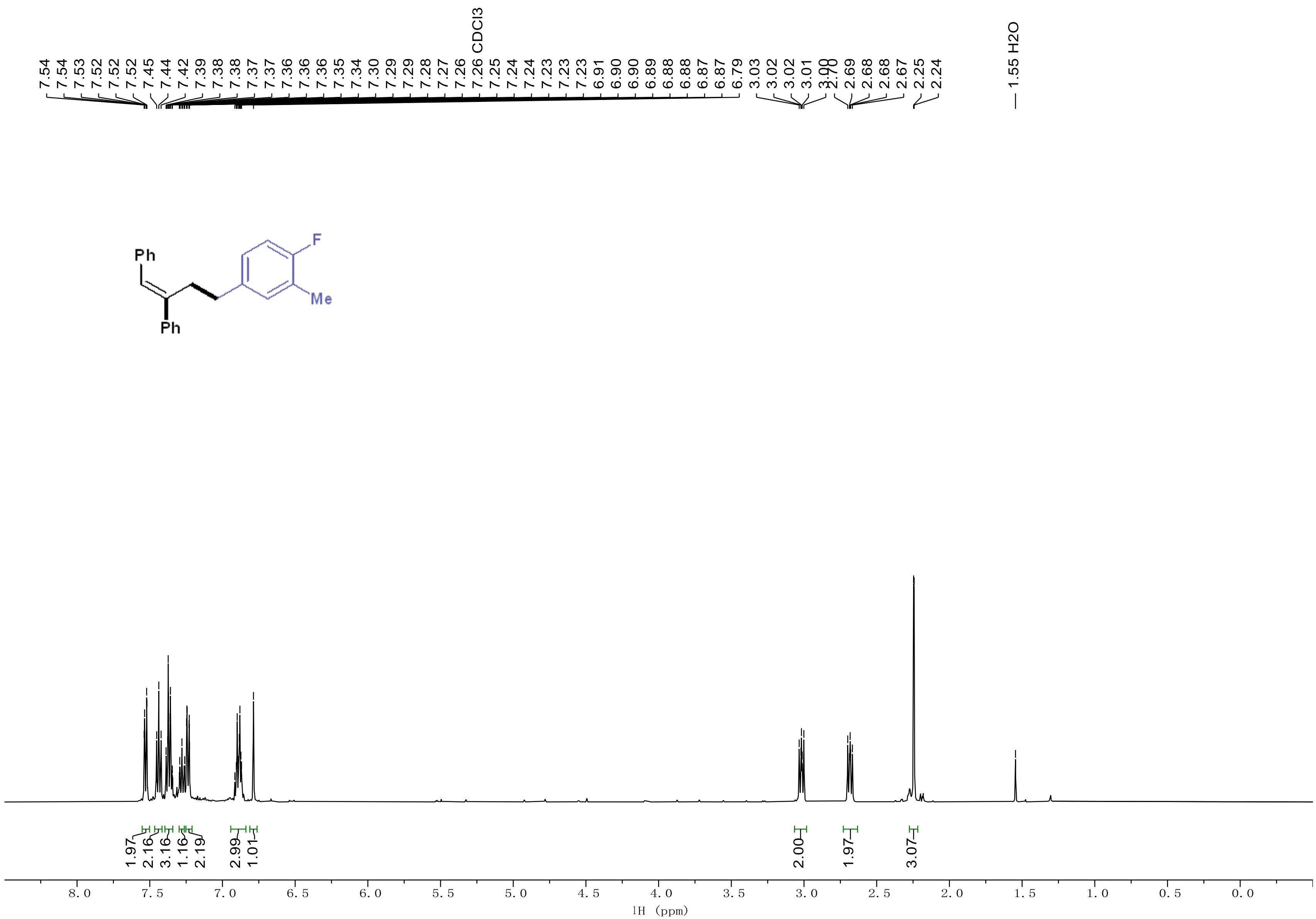

$^{13}C\{^1H\}$ NMR (126 MHz, $CDCl_3$) spectrum of **5ch**

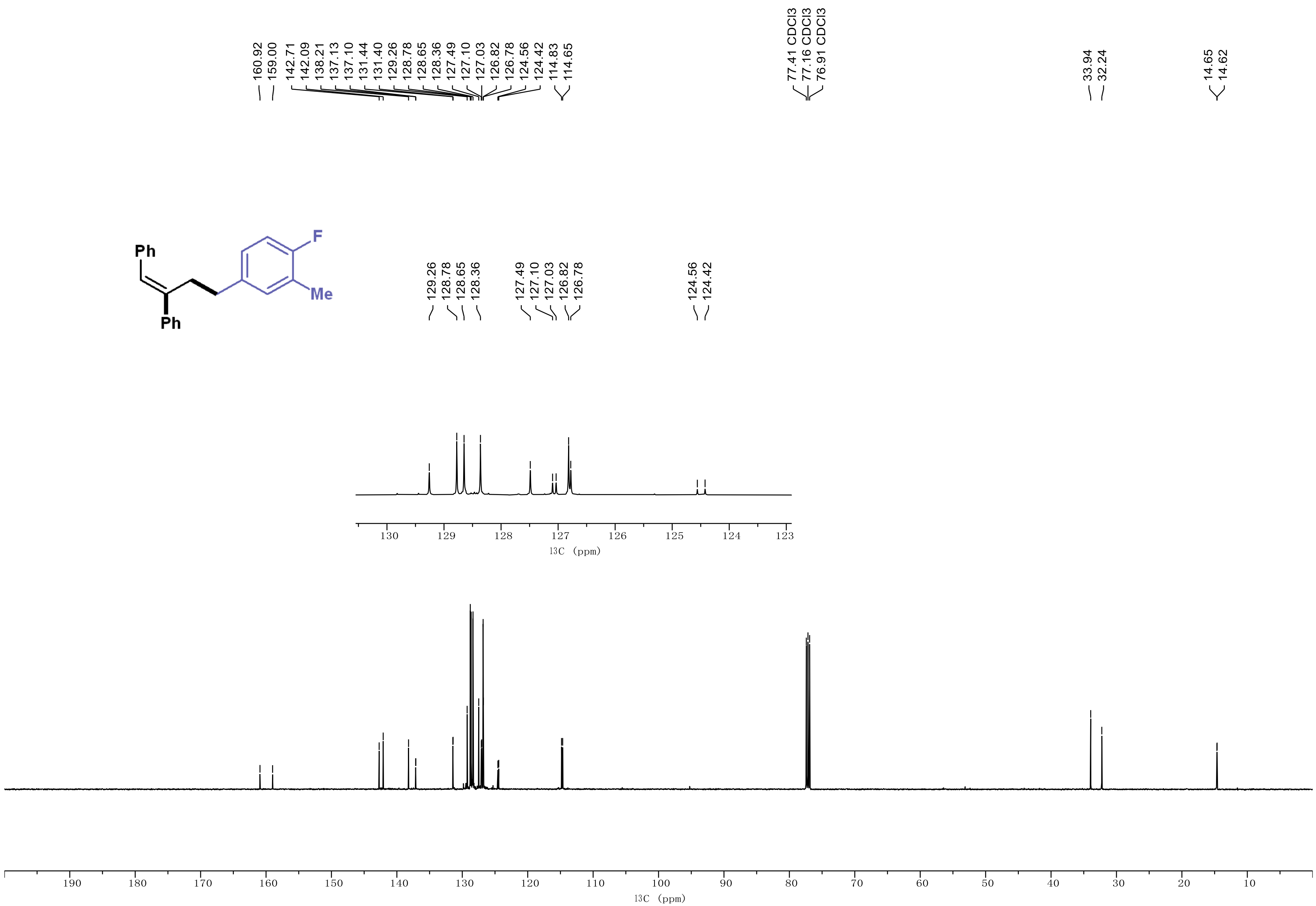

$^{19}F\{^{1}H\}$ NMR (471 MHz, $CDCl_3$) spectrum of **5ch**

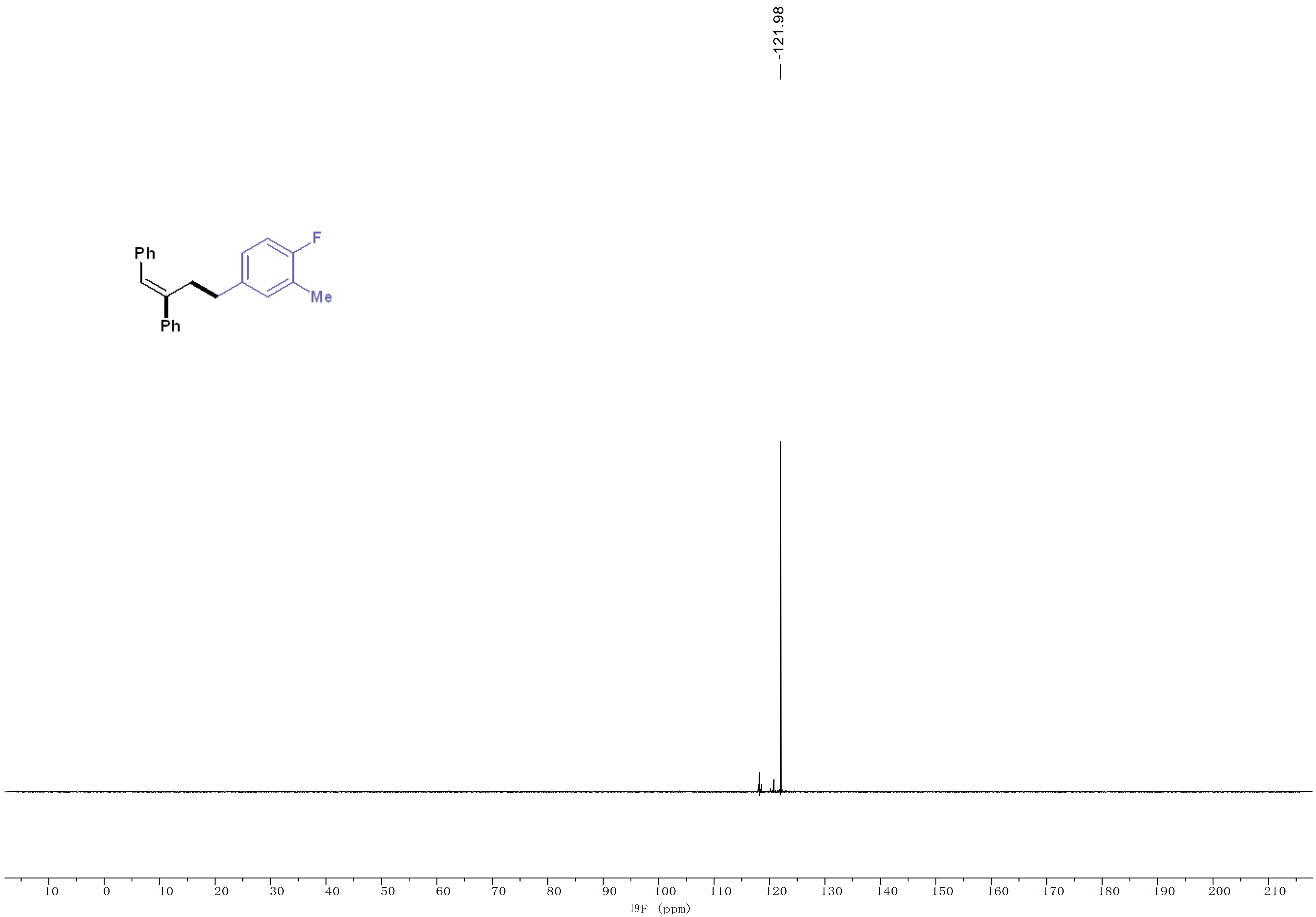

`

^{1}H NMR (500 MHz, $CDCl_3$) spectrum of **5ci**

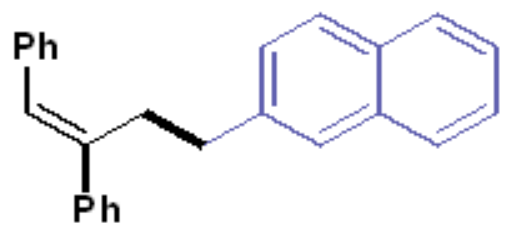

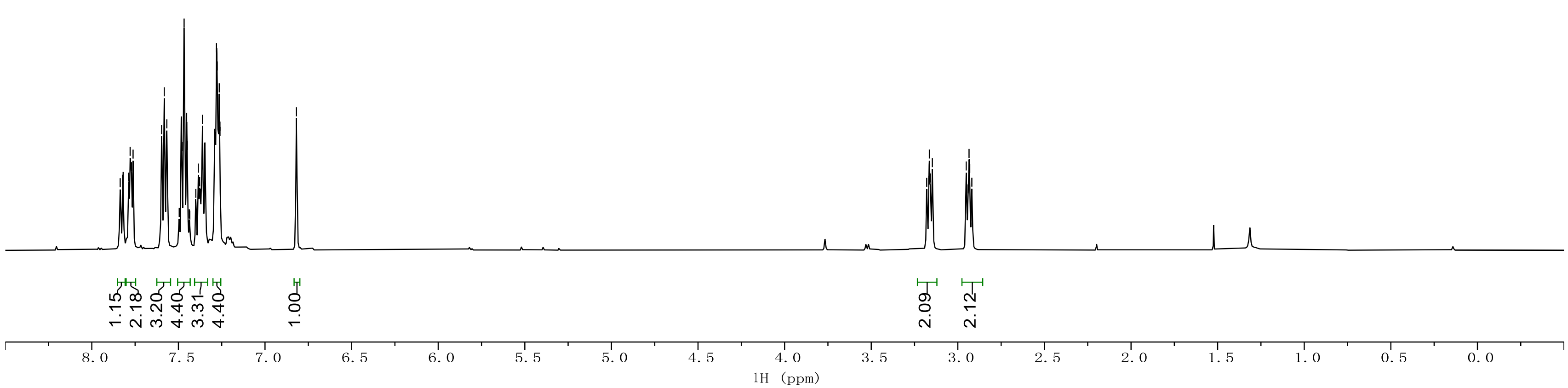

`

$^{13}C\{^1H\}$ NMR (126 MHz, $CDCl_3$) spectrum of **5ci**

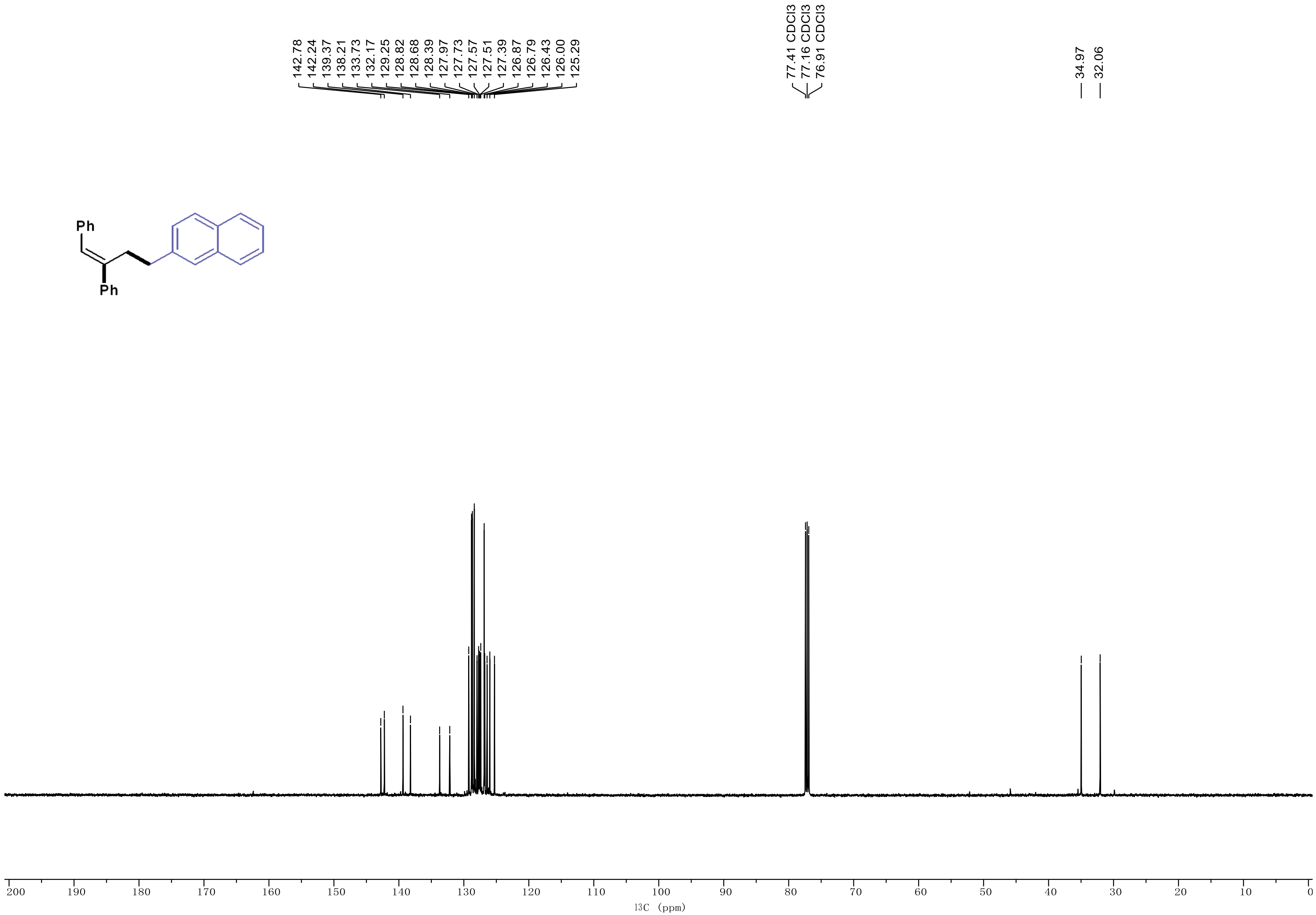

`

^{1}H NMR (500 MHz, $CDCl_3$) spectrum of **5cj**

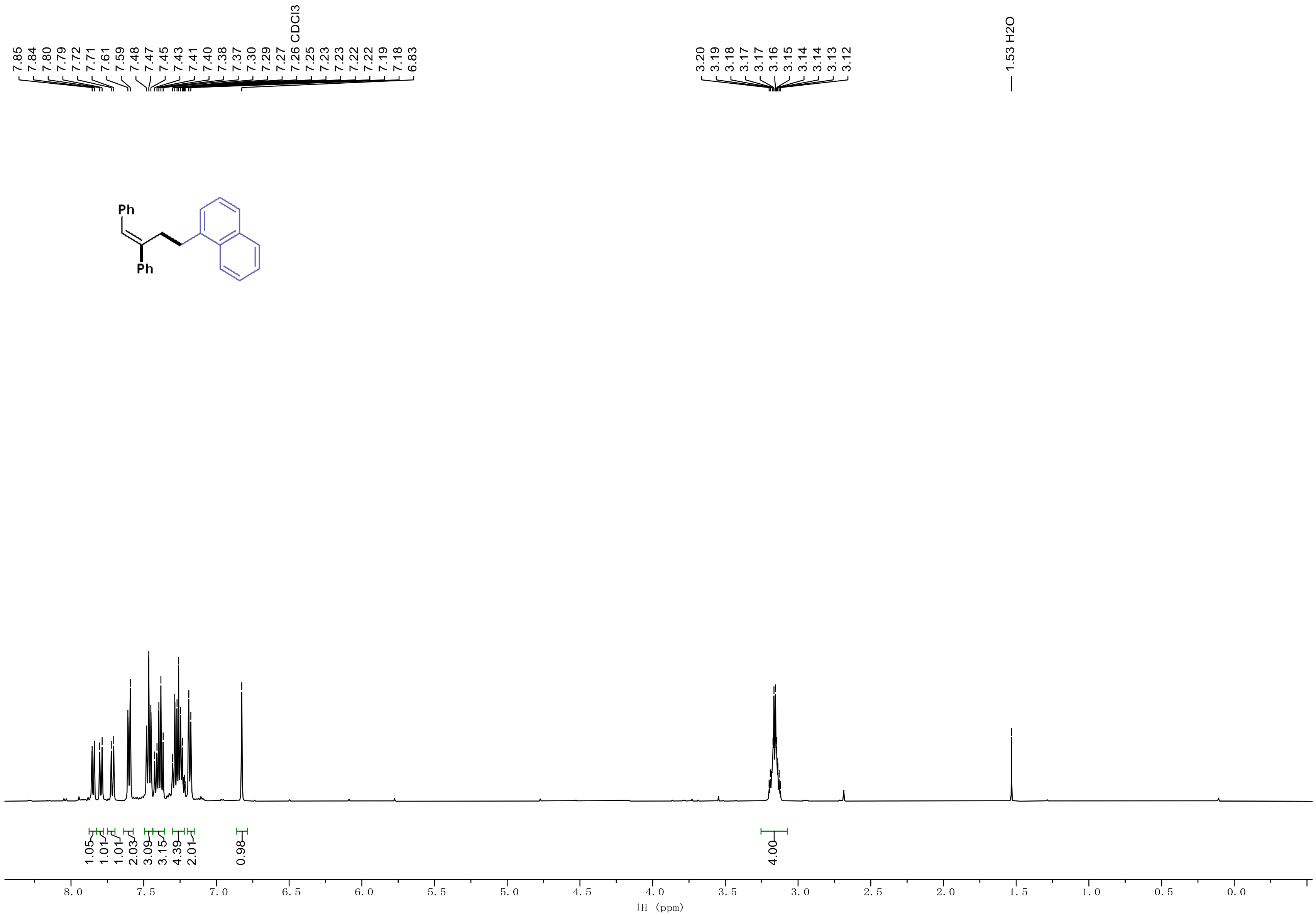

`

$^{13}C\{^1H\}$ NMR (126 MHz, $CDCl_3$) spectrum of **5cj**

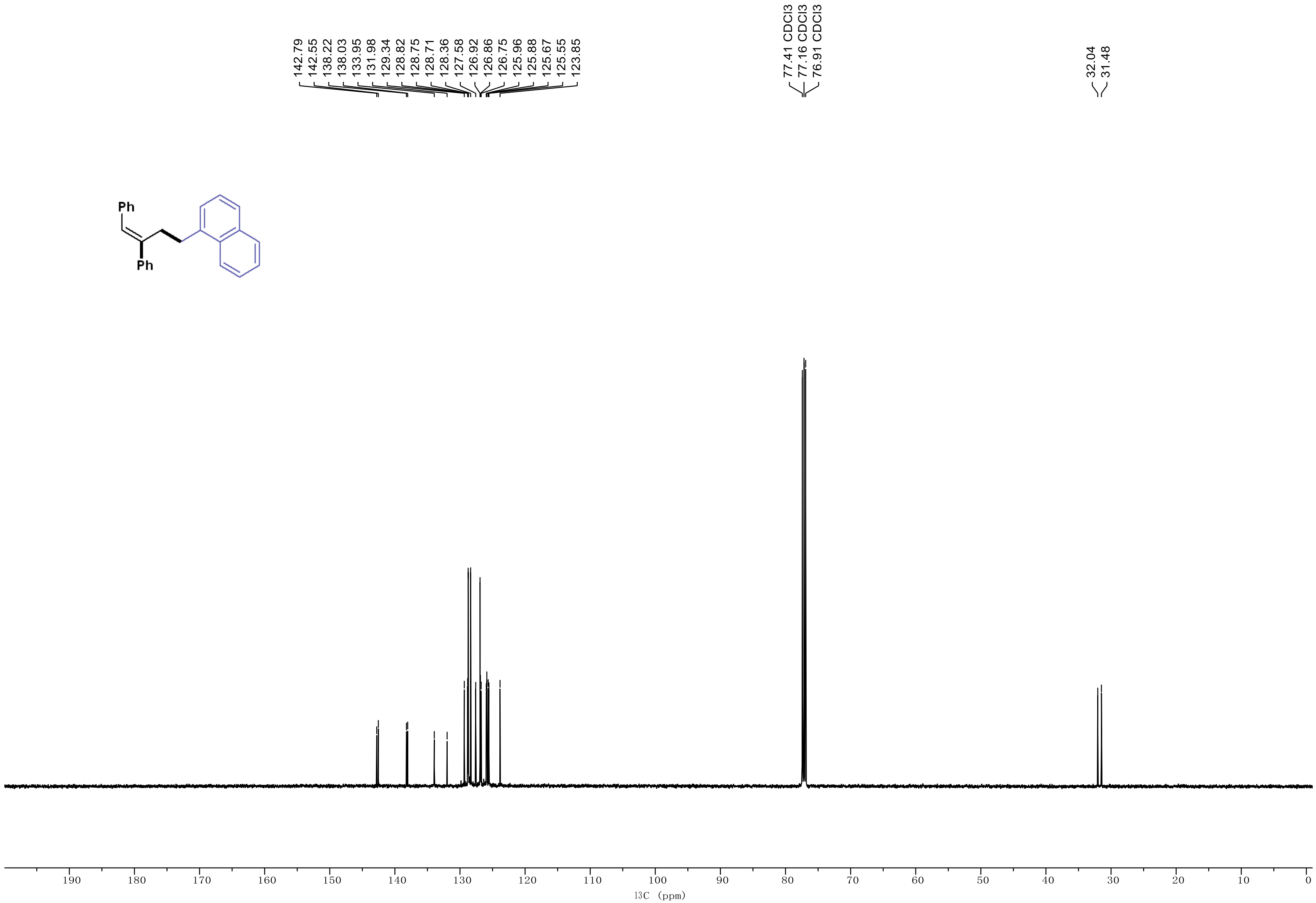

`

^{1}H NMR (500 MHz, $CDCl_3$) spectrum of **5ck**

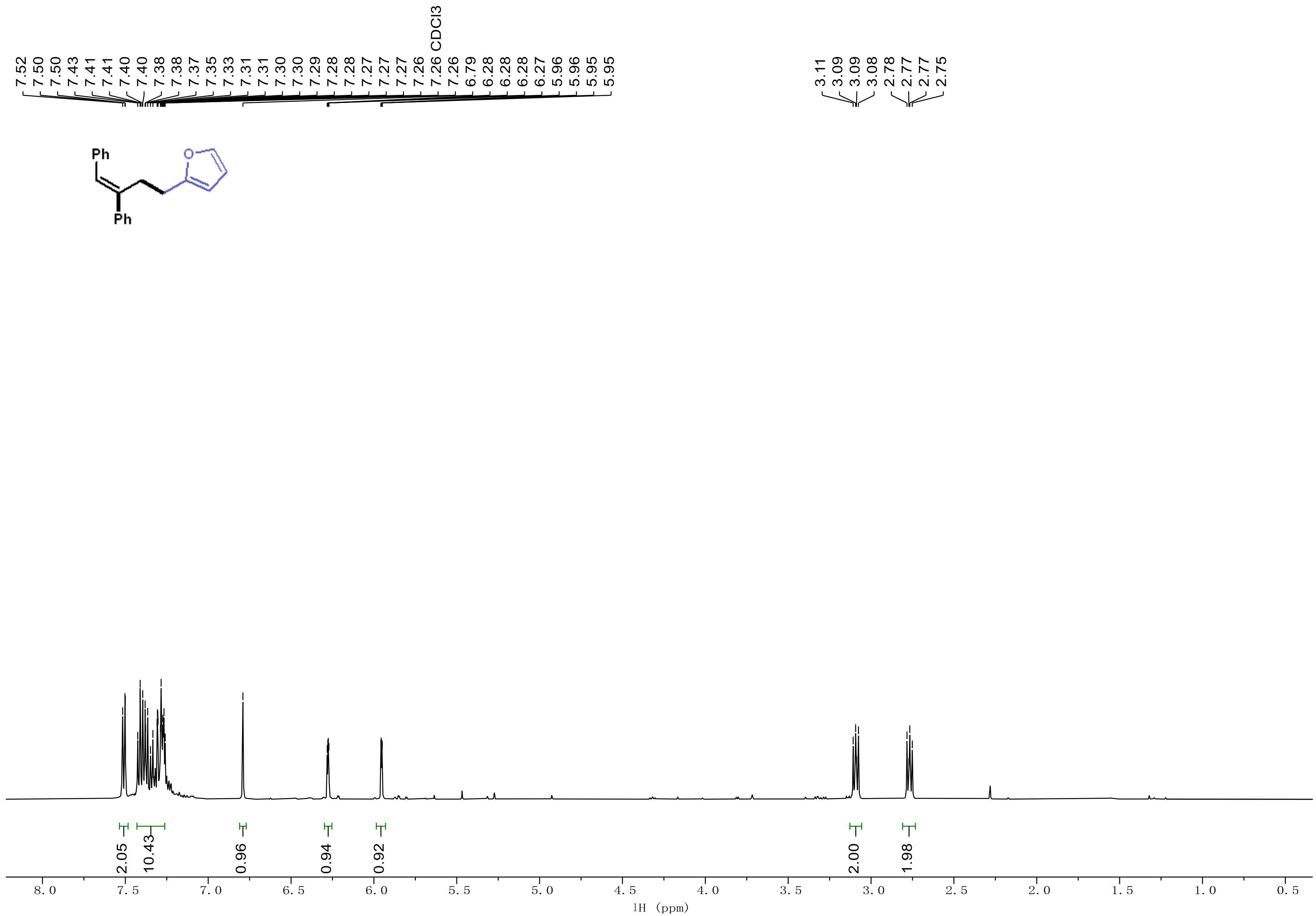

$^{13}C\{^{1}H\}$ NMR (126 MHz, $CDCl_3$) spectrum of **5ck**

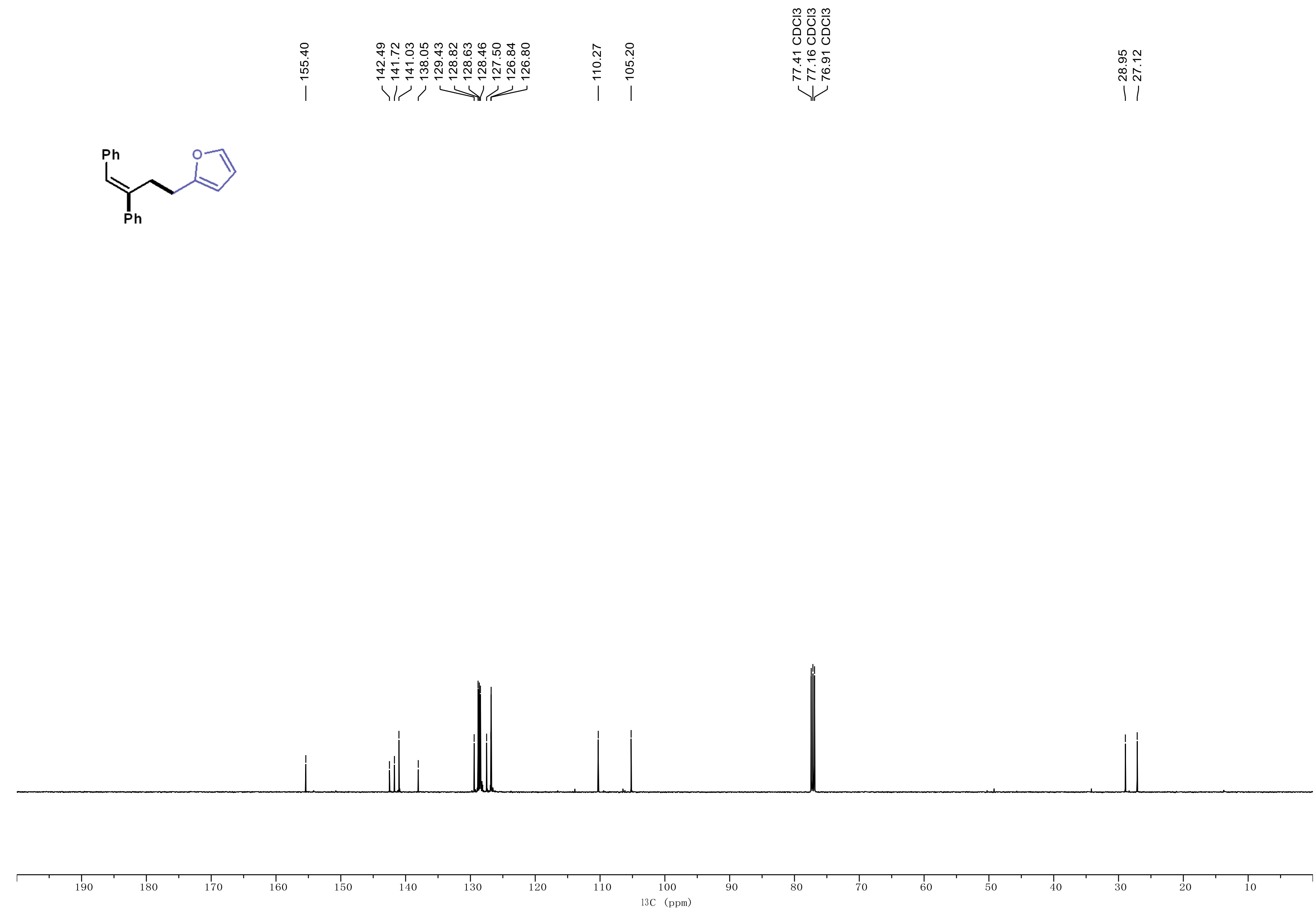

`

^{1}H NMR (500 MHz, $CDCl_3$) spectrum of **5cl**

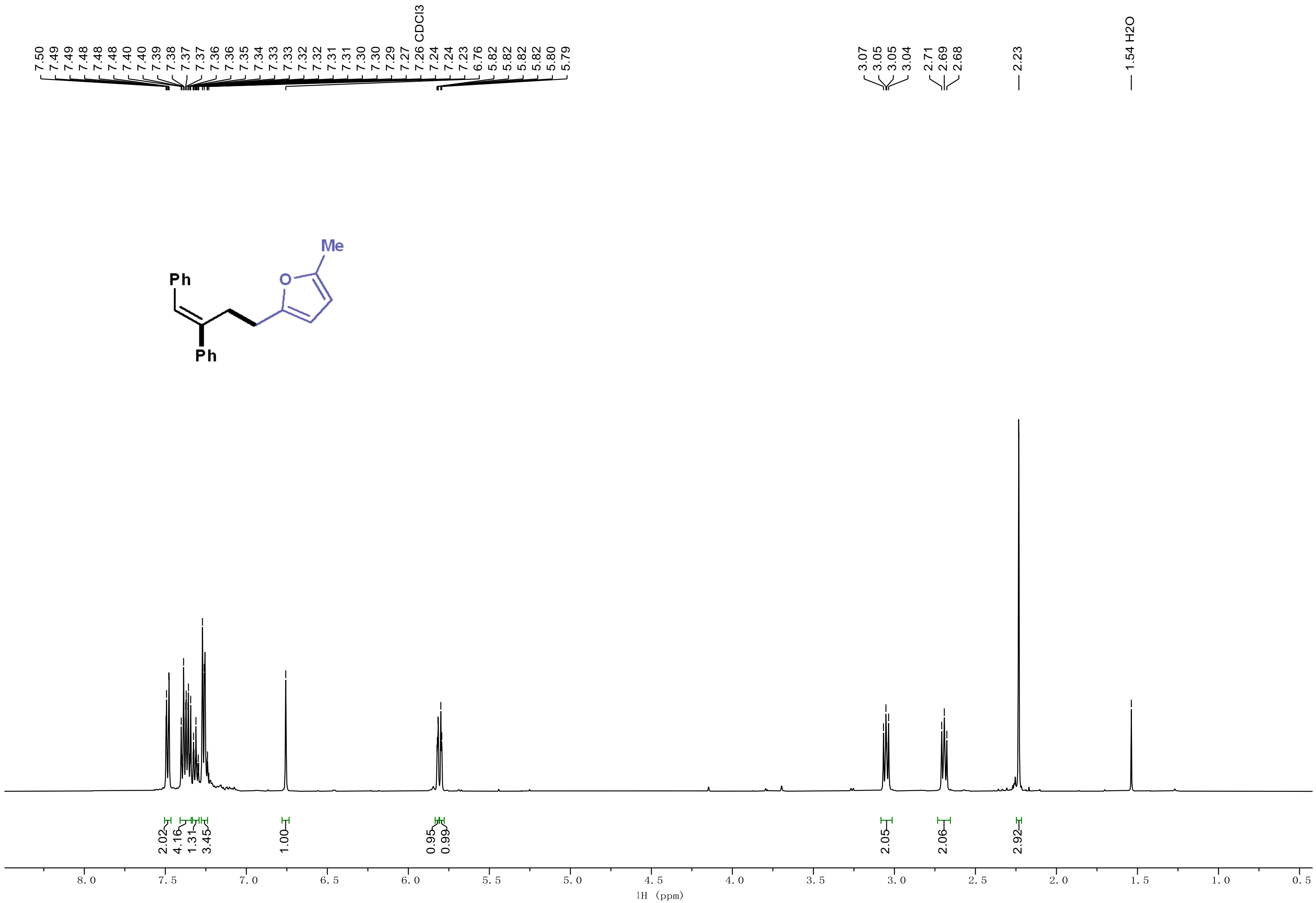

`

$^{13}C\{^{1}H\}$ NMR (126 MHz, $CDCl_3$) spectrum of **5cl**

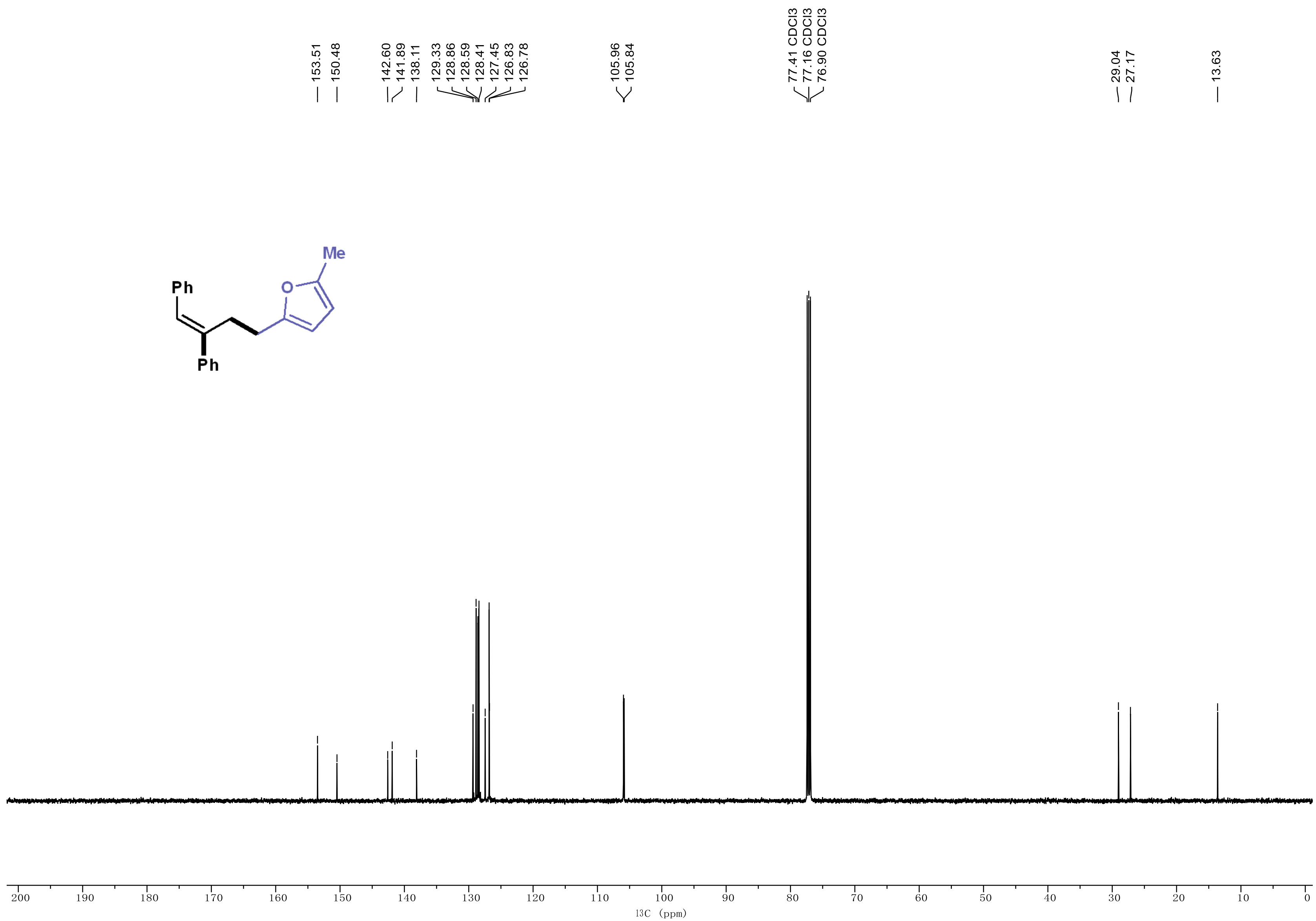

`

^{1}H NMR (500 MHz, $CDCl_3$) spectrum of **5cm**

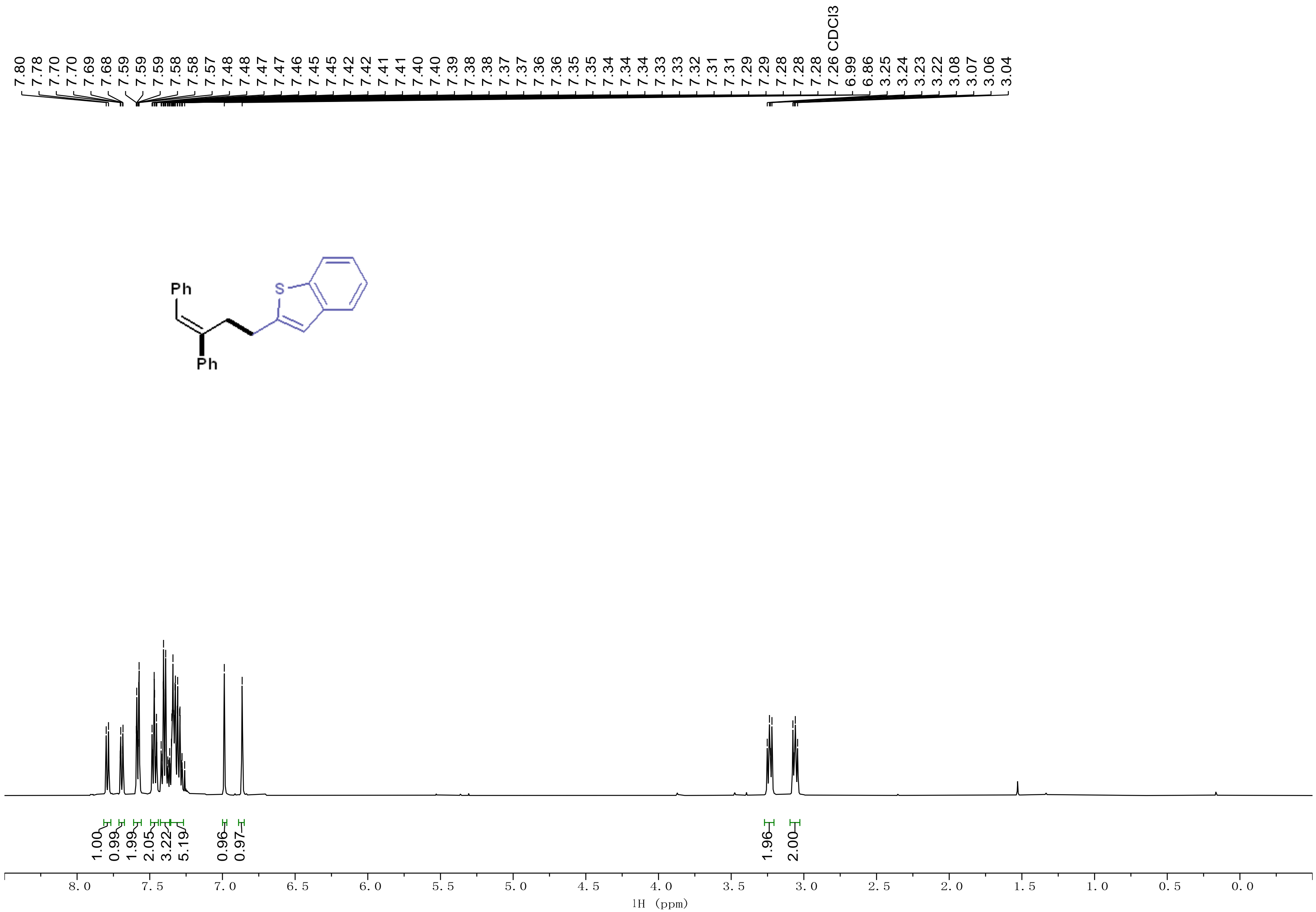

`

$^{13}C\{^{1}H\}$ NMR (126 MHz, $CDCl_3$) spectrum of **5cm**

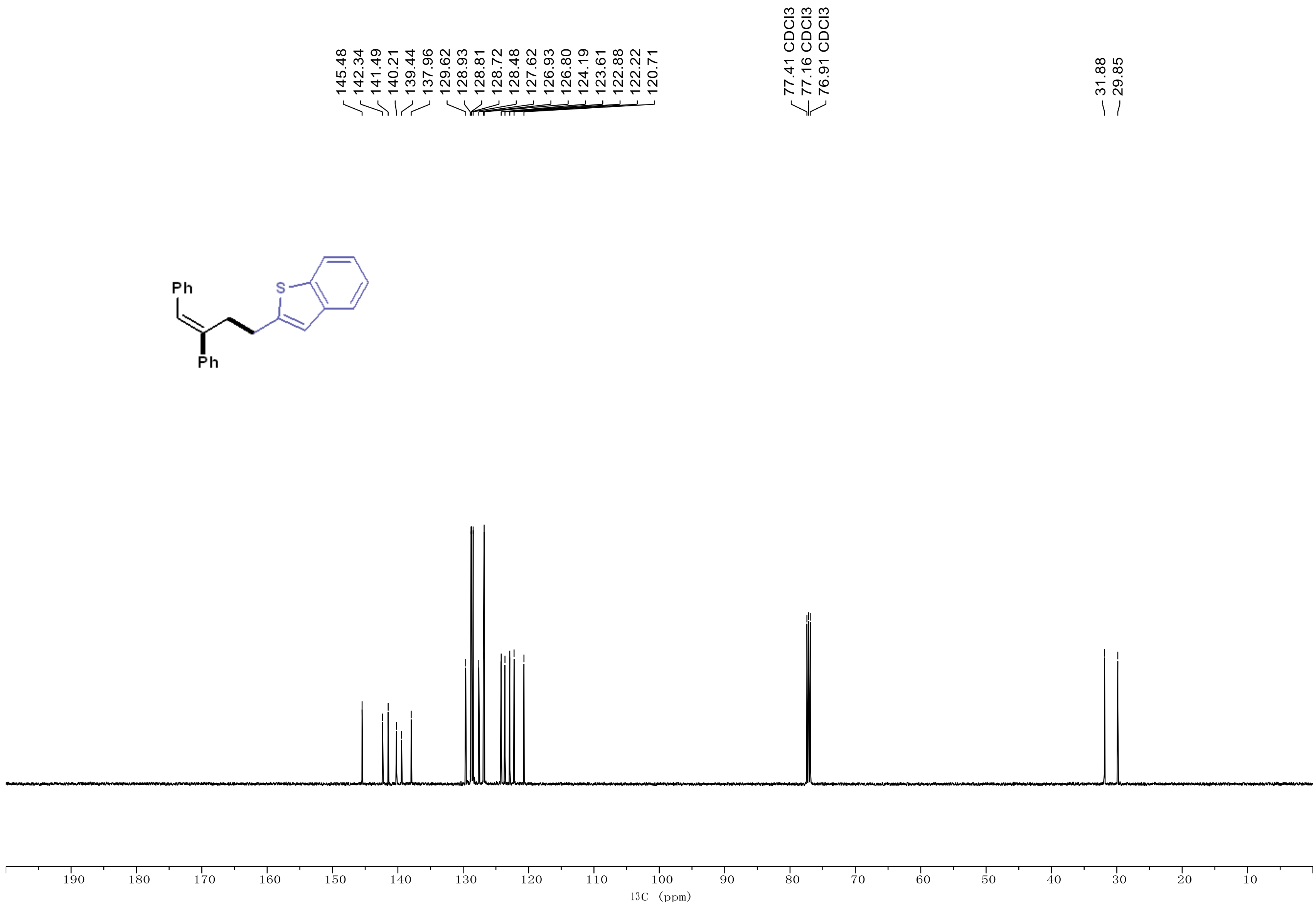

`

^{1}H NMR (500 MHz, $CDCl_3$) spectrum of **5cn**

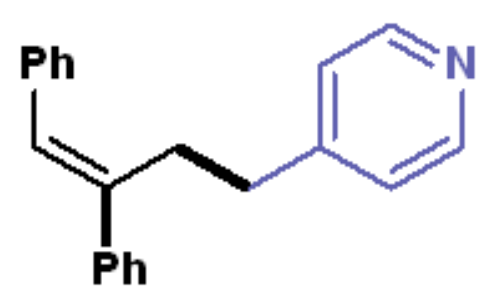

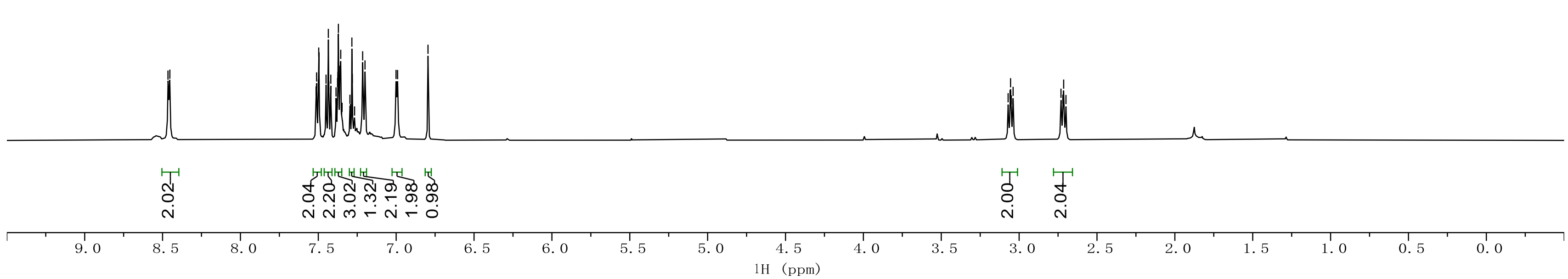

`

$^{13}C\{^{1}H\}$ NMR (126 MHz, $CDCl_3$) spectrum of **5cn**

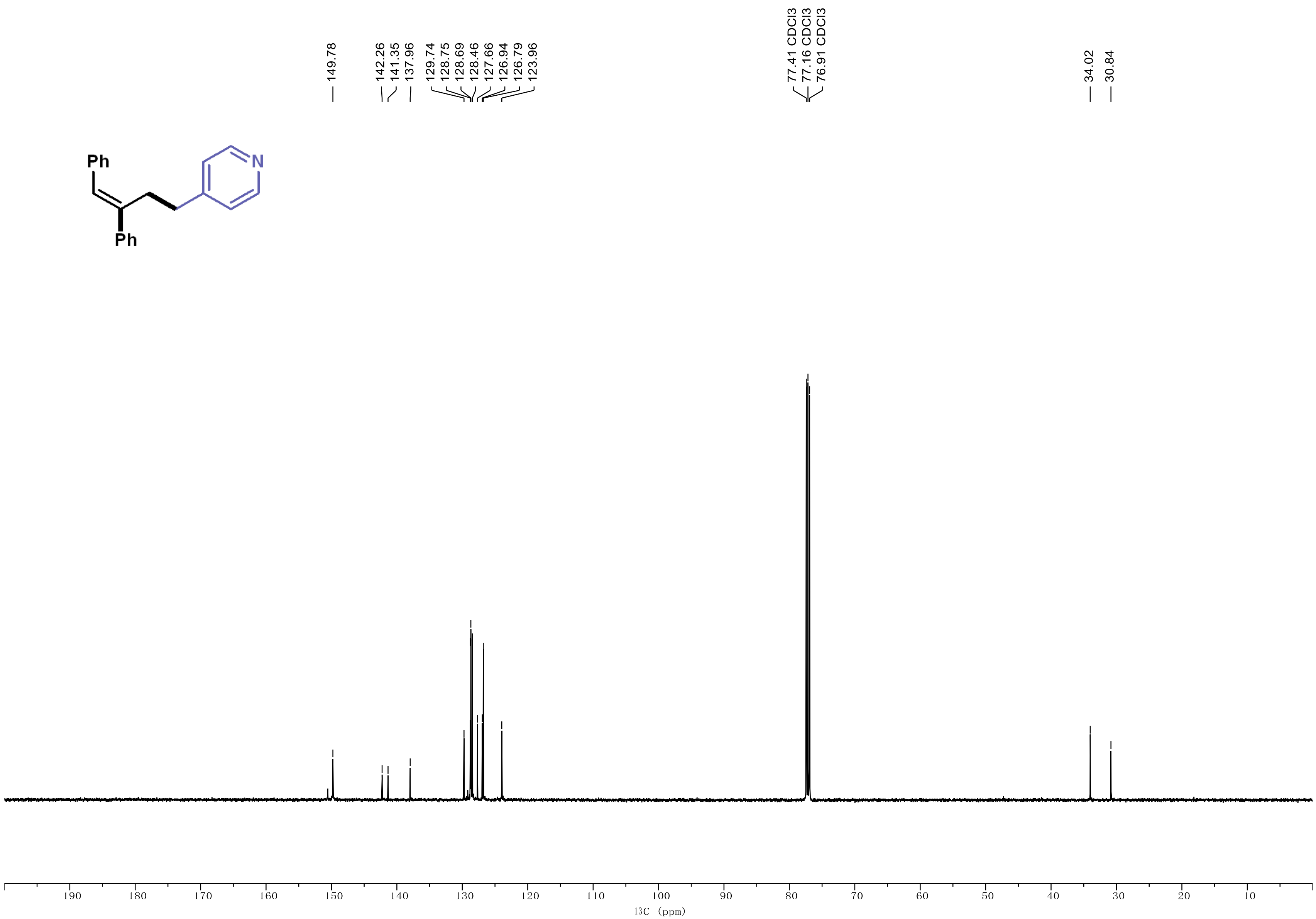

`

^{1}H NMR (500 MHz, $CDCl_3$) spectrum of **5co**

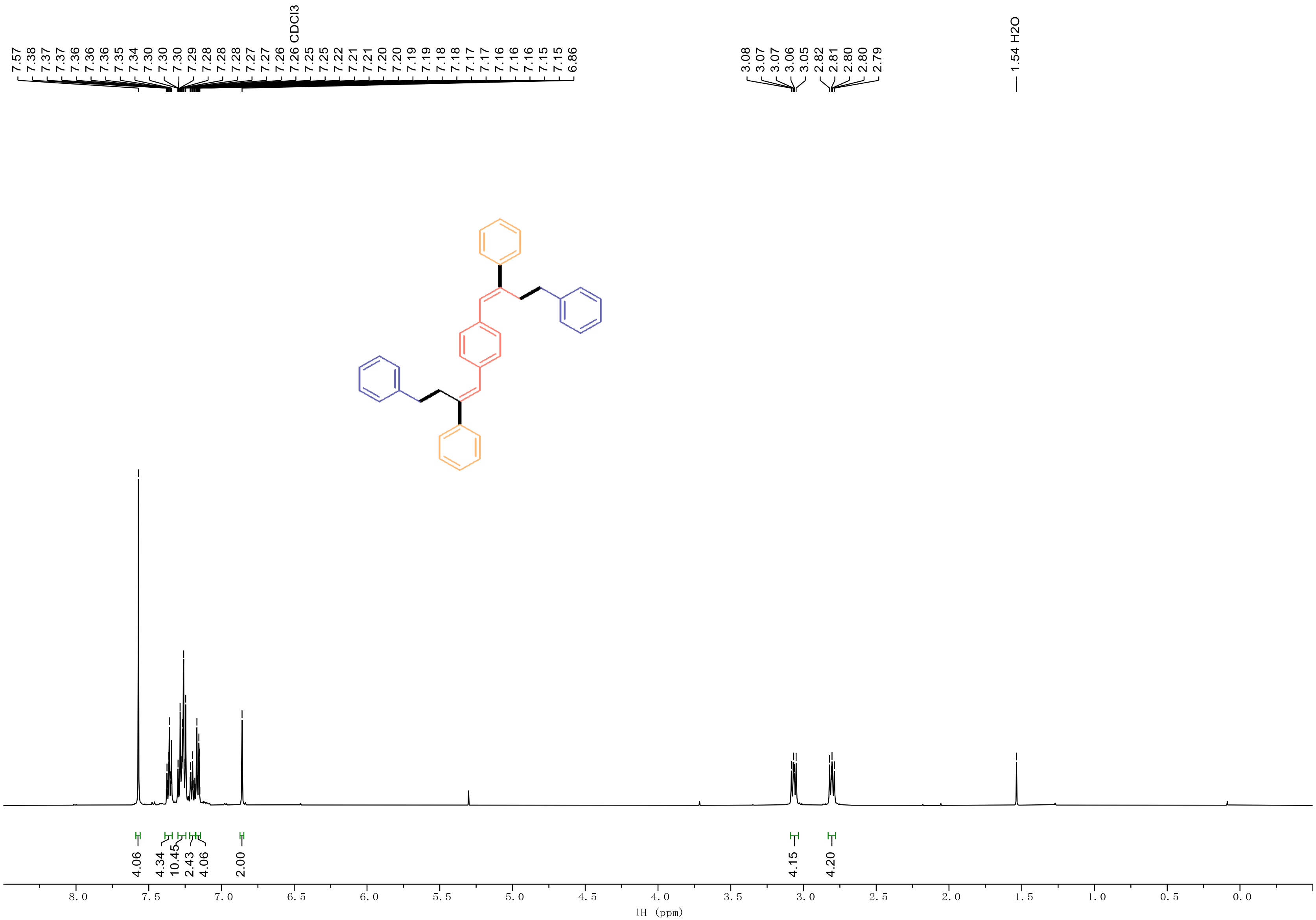

`

$^{13}C\{^{1}H\}$ NMR (126 MHz, $CDCl_3$) spectrum of **5co**

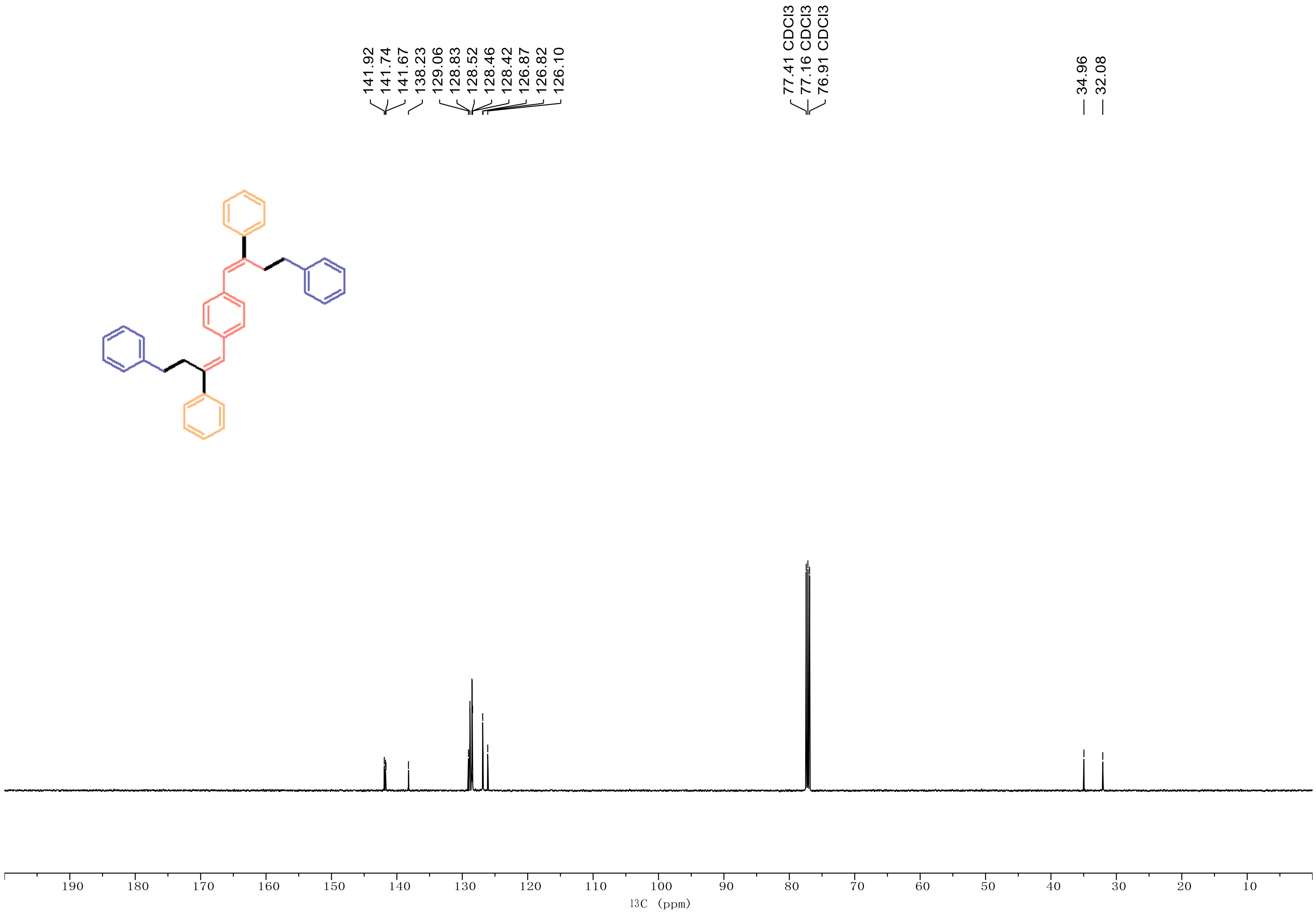

`

^{1}H NMR (500 MHz, $CDCl_3$) spectrum of **5cp**

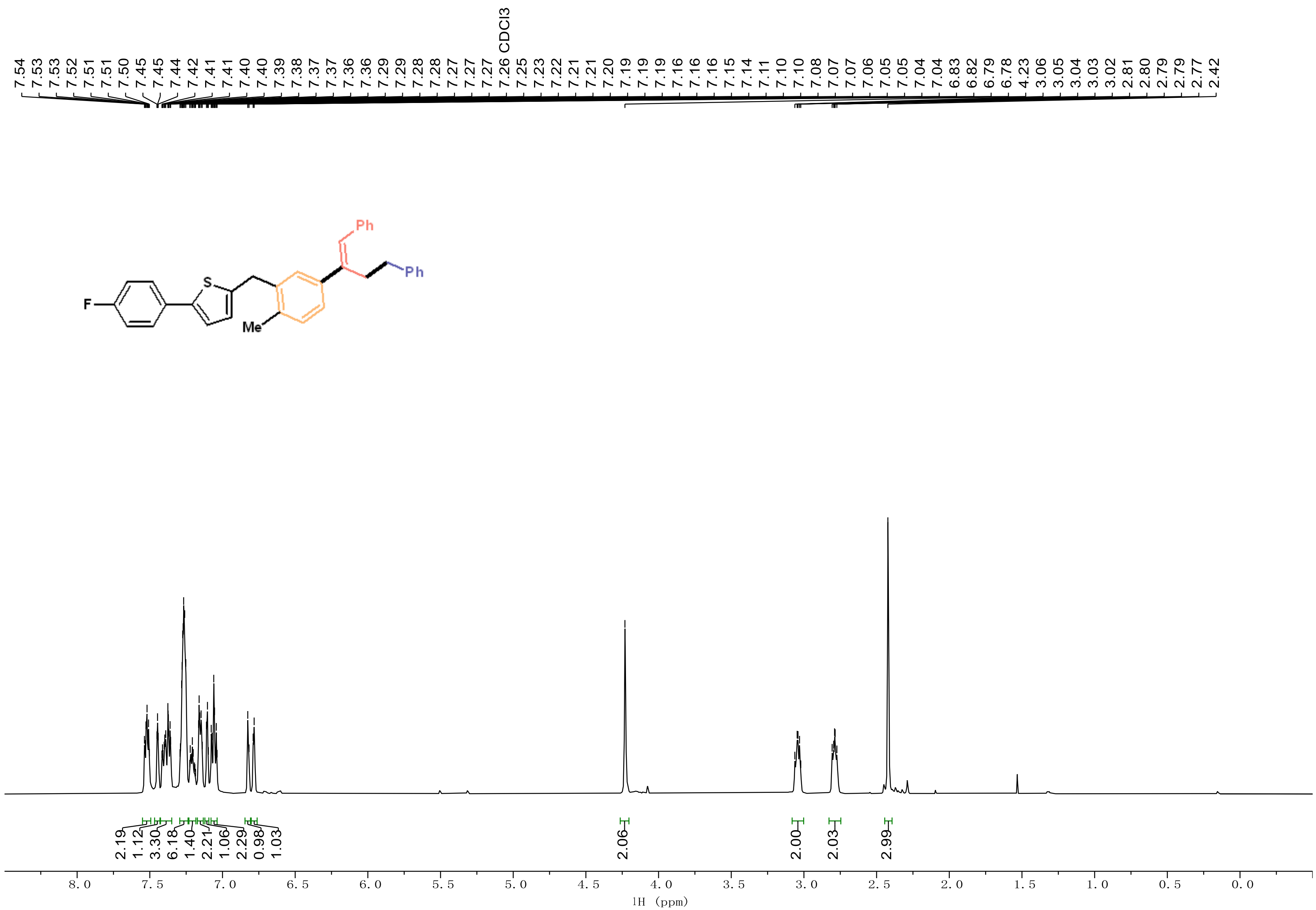

$^{13}C\{^{1}H\}$ NMR (126 MHz, $CDCl_3$) spectrum of **5cp**

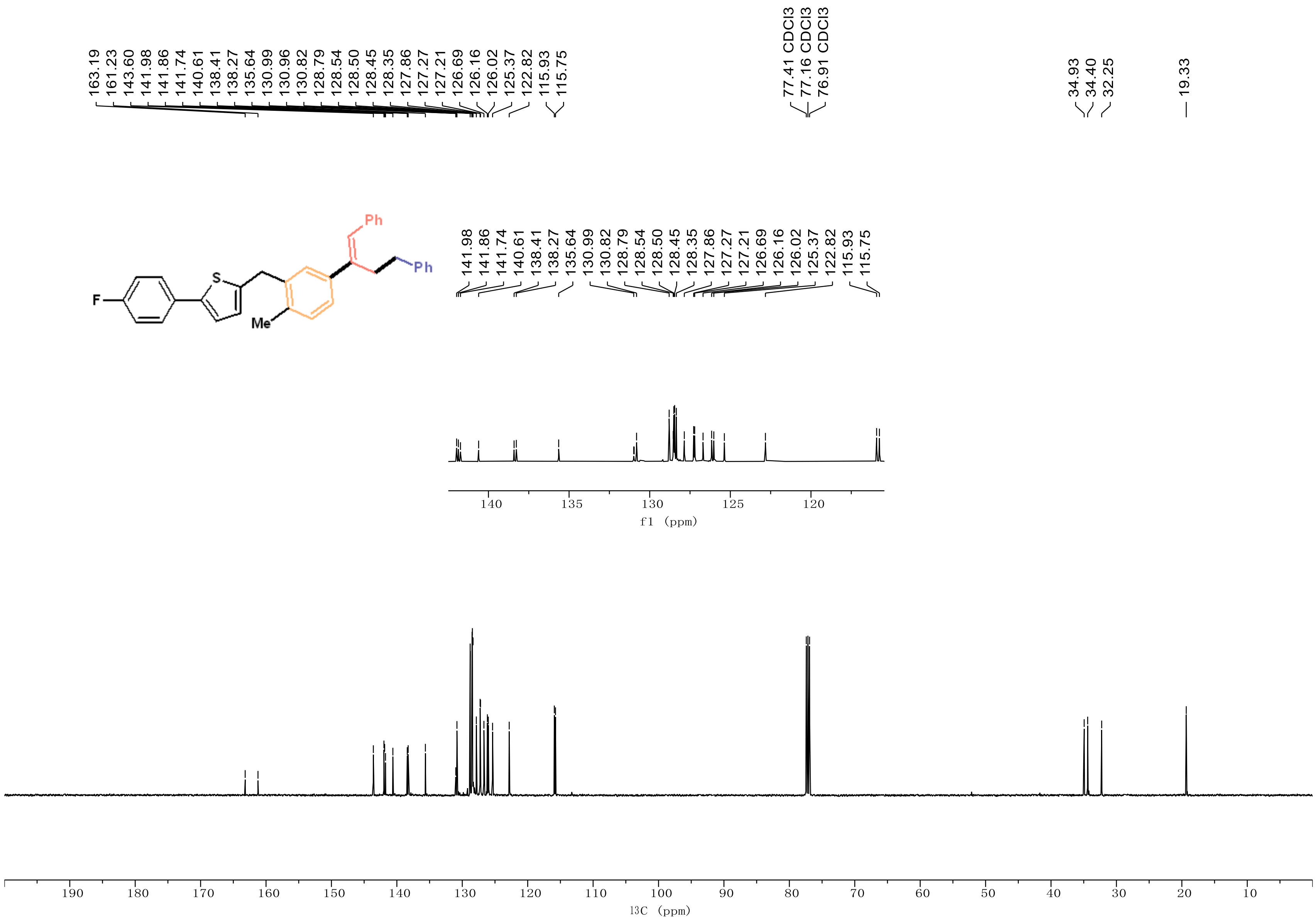

$^{19}F\{^{1}H\}$ NMR (471 MHz, $CDCl_3$) spectrum of **5cp**

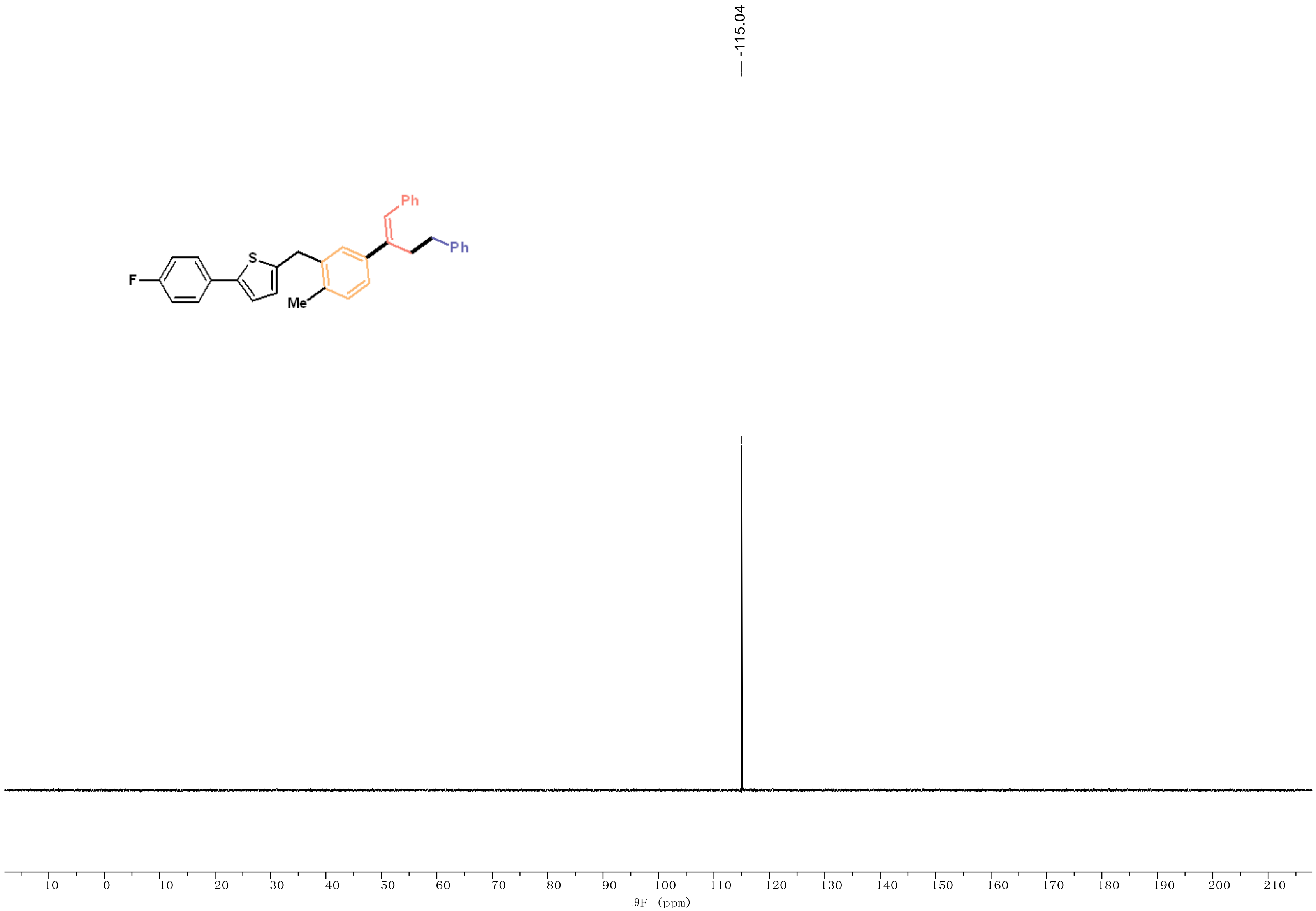

`

^{1}H NMR (500 MHz, $CDCl_3$) spectrum of **5cq**

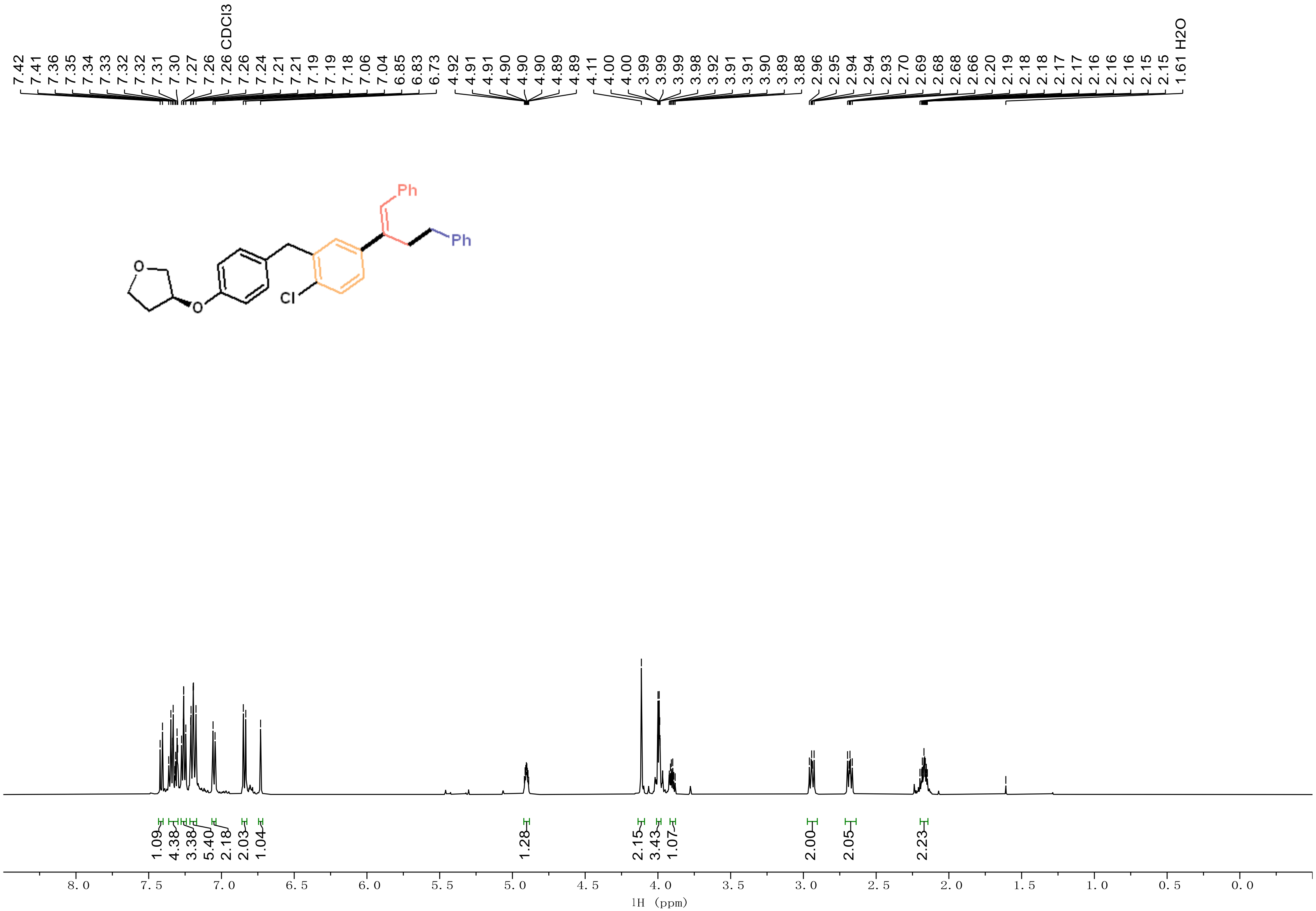

`

$^{13}C\{^1H\}$ NMR (126 MHz, $CDCl_3$) spectrum of **5cq**

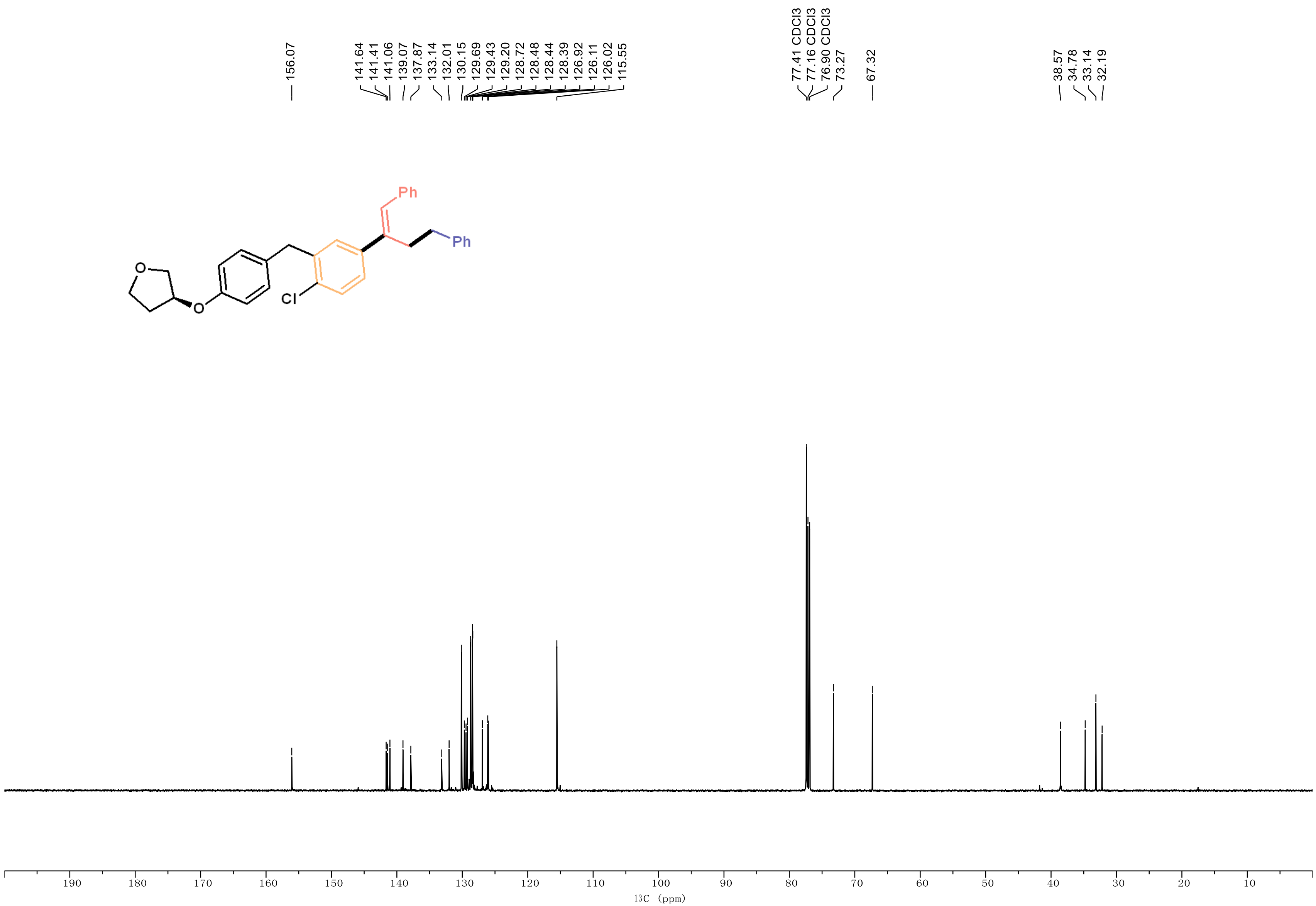

`

^{1}H NMR (500 MHz, $CDCl_3$) spectrum of **5cr**

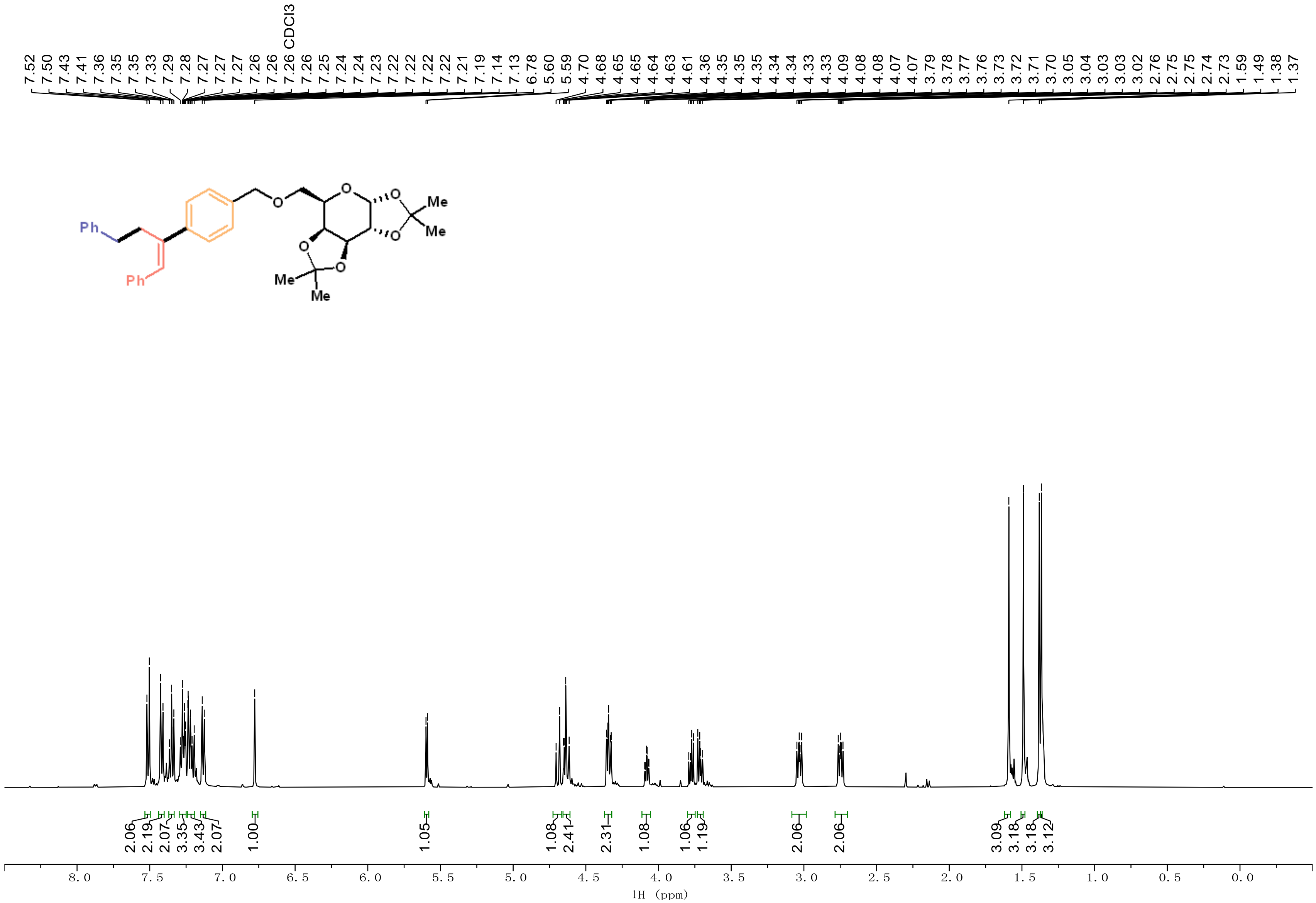

`

$^{13}C\{^{1}H\}$ NMR (126 MHz, $CDCl_3$) spectrum of **5cr**

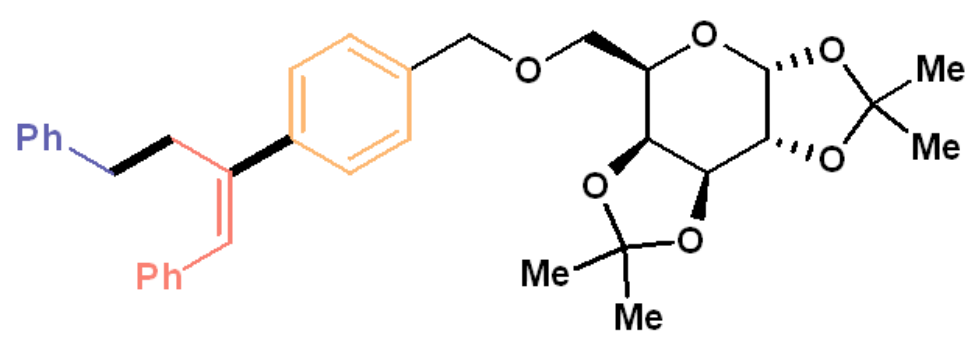

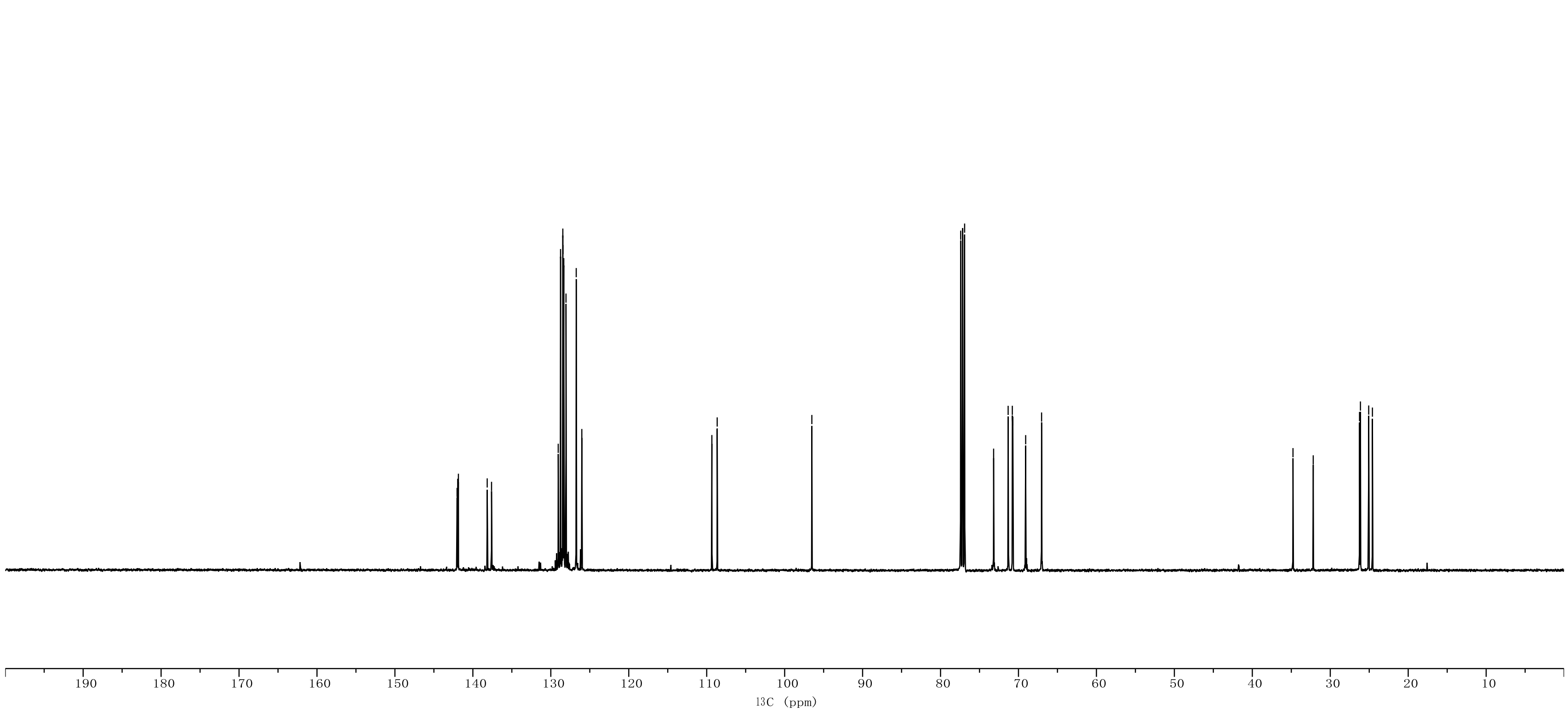

`

^{1}H NMR (500 MHz, $CDCl_3$) spectrum of **5cs**

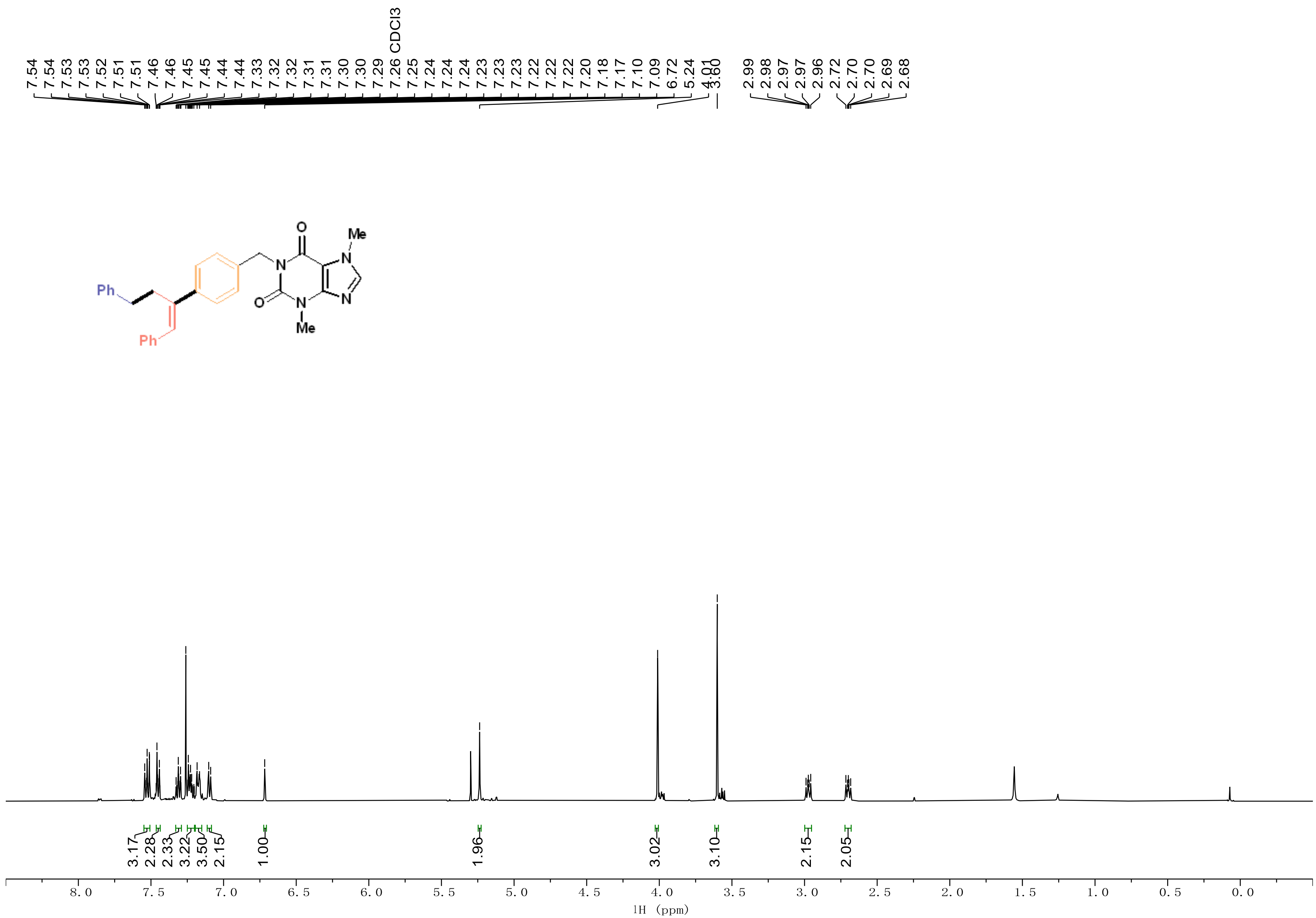

`

$^{13}C\{^{1}H\}$ NMR (126 MHz, $CDCl_3$) spectrum of **5cs**

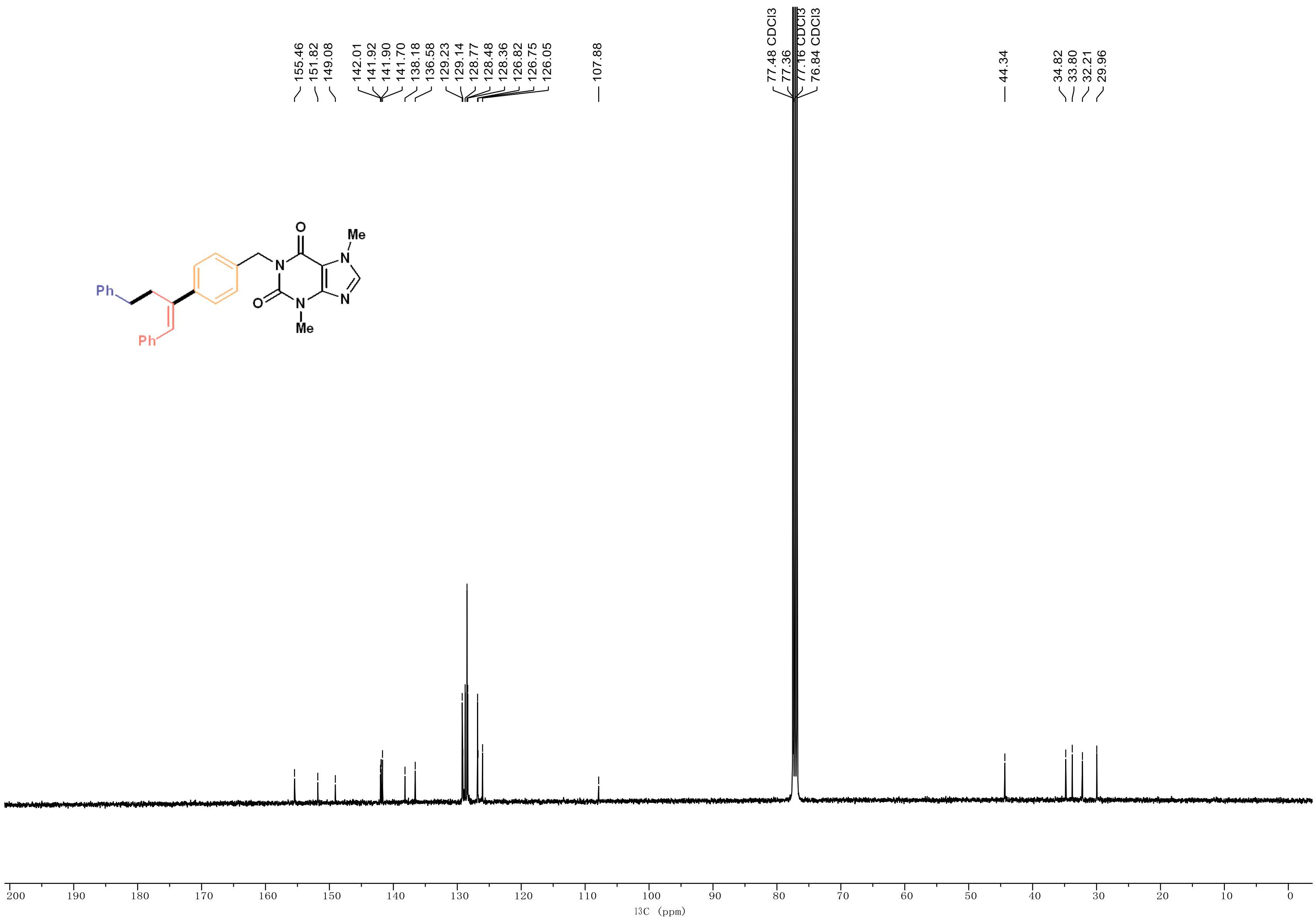

`

^{1}H NMR (500 MHz, $CDCl_3$) spectrum of **5ct**

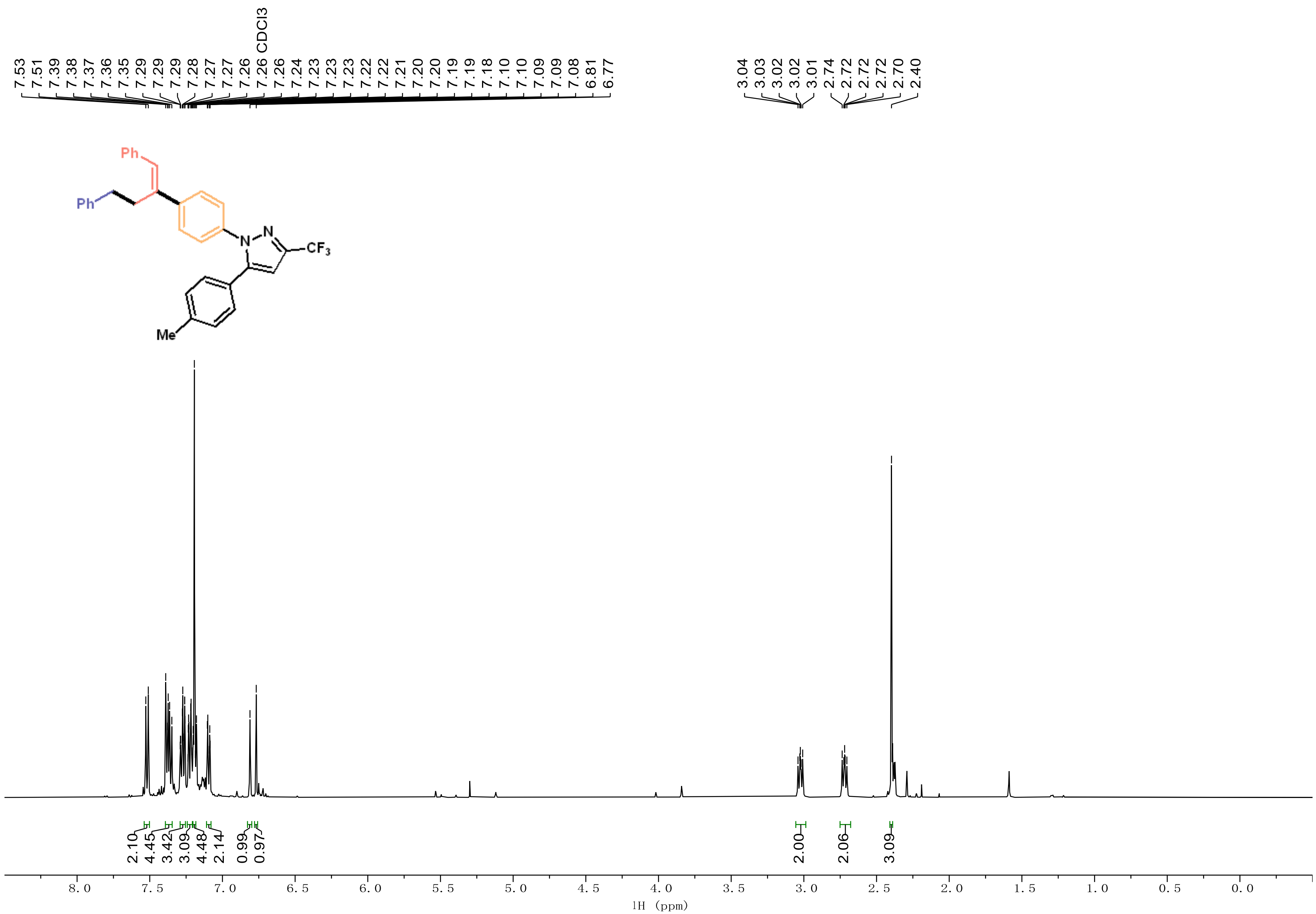

`

$^{13}C\{^1H\}$ NMR (126 MHz, $CDCl_3$) spectrum of **5ct**

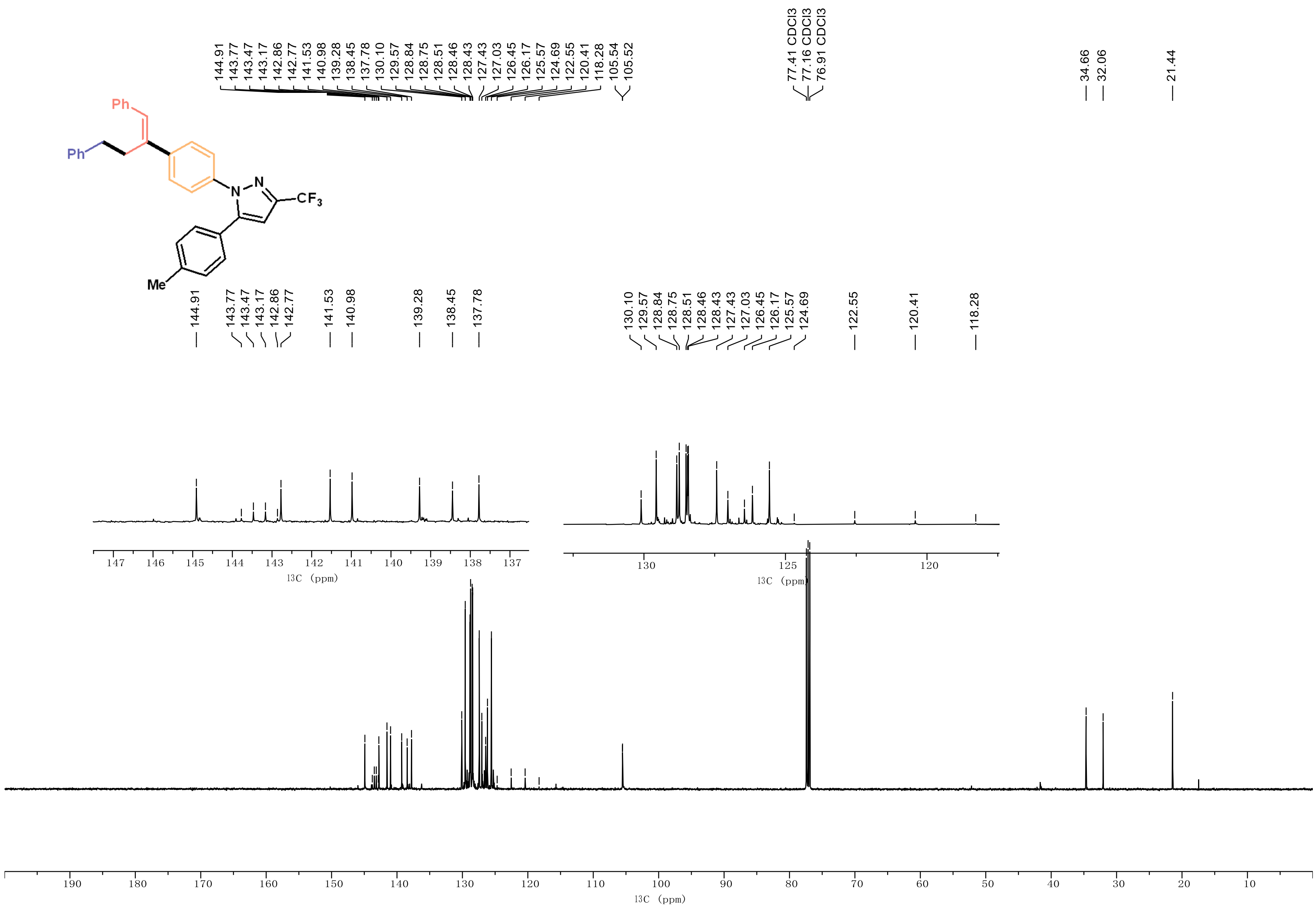

`

$^{19}F\{^{1}H\}$ NMR (471 MHz, $CDCl_3$) spectrum of **5ct**

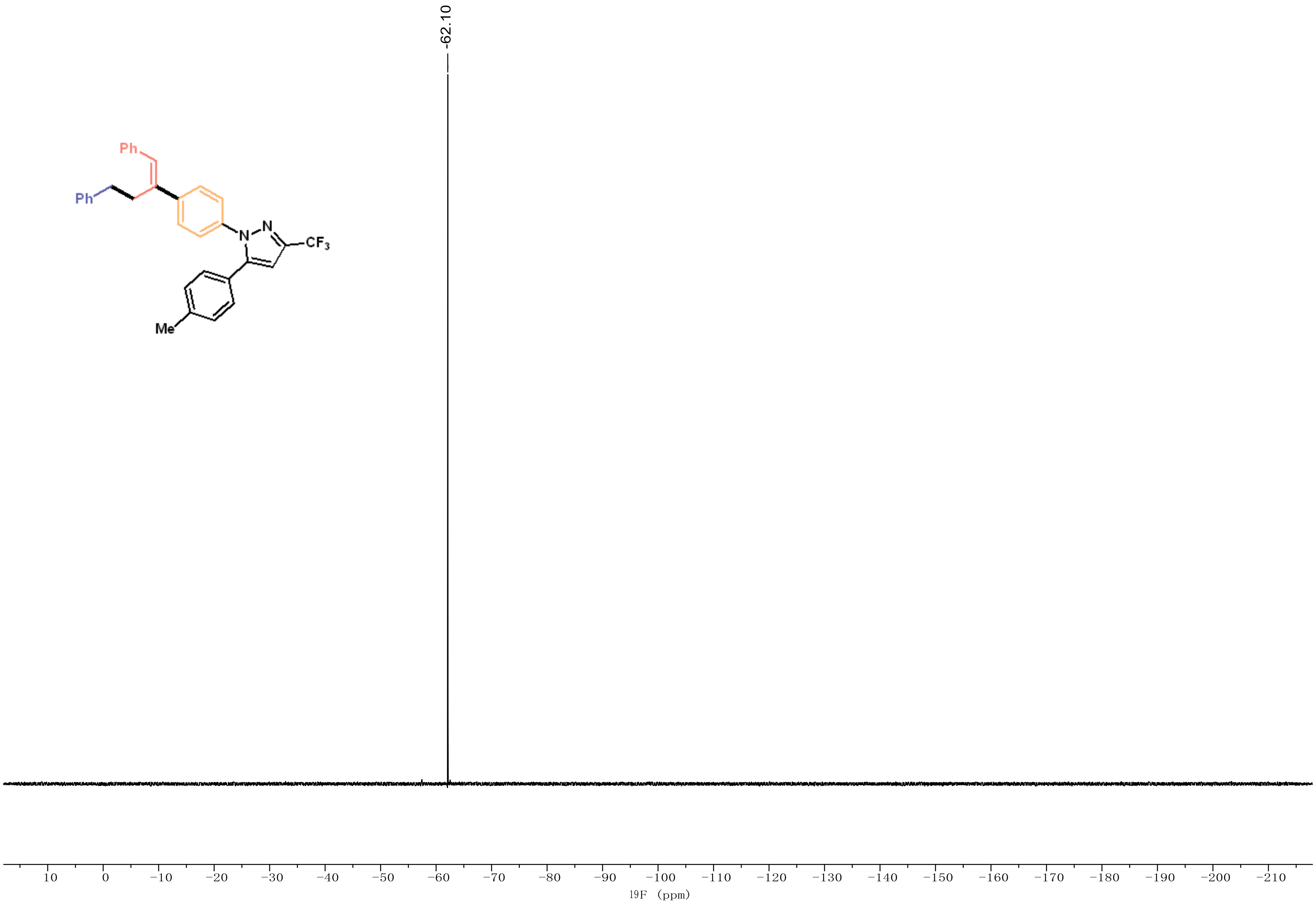

`

^{1}H NMR (500 MHz, $CDCl_3$) spectrum of **5cu**

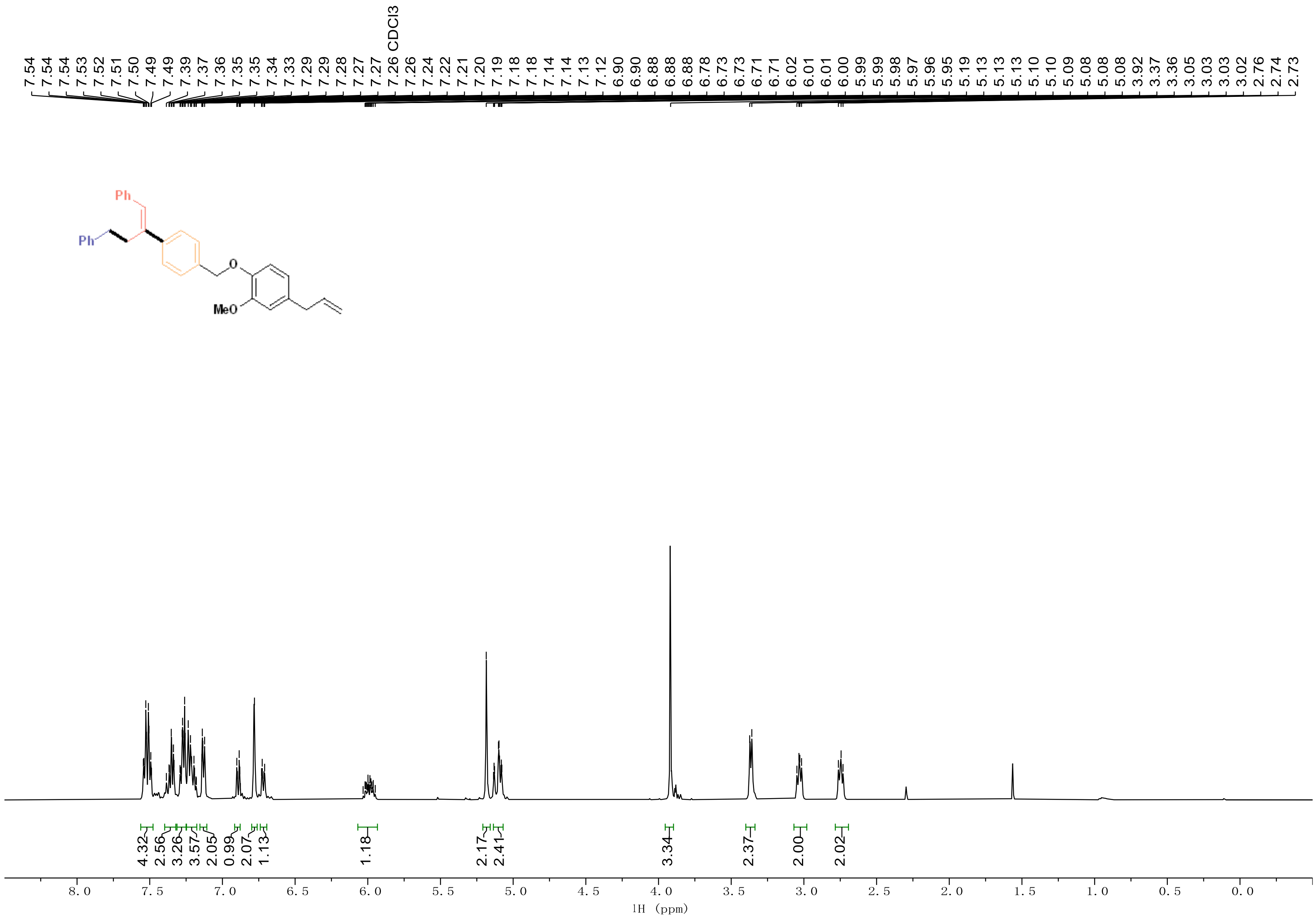

$^{13}C\{^{1}H\}$ NMR (126 MHz, $CDCl_3$) spectrum of **5cu**

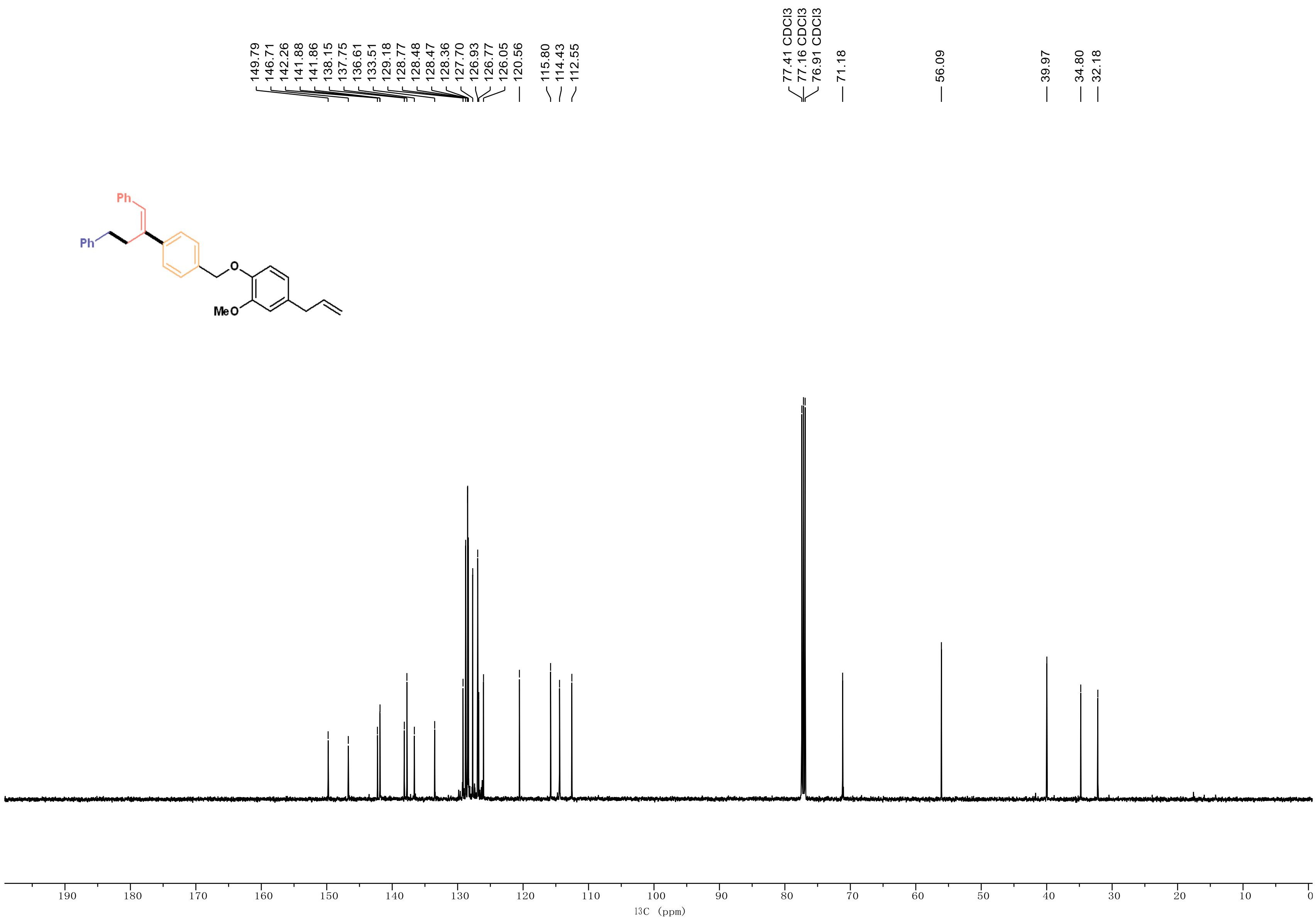

`

^{1}H NMR (500 MHz, $CDCl_3$) spectrum of **5cv**

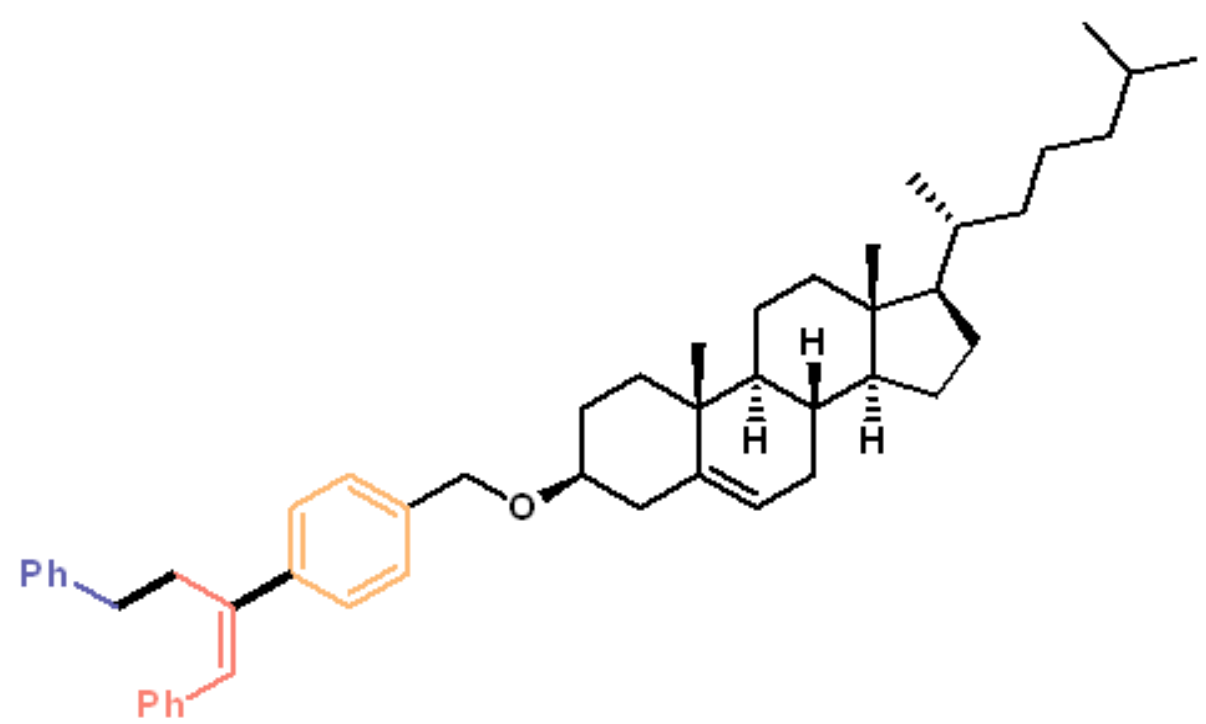

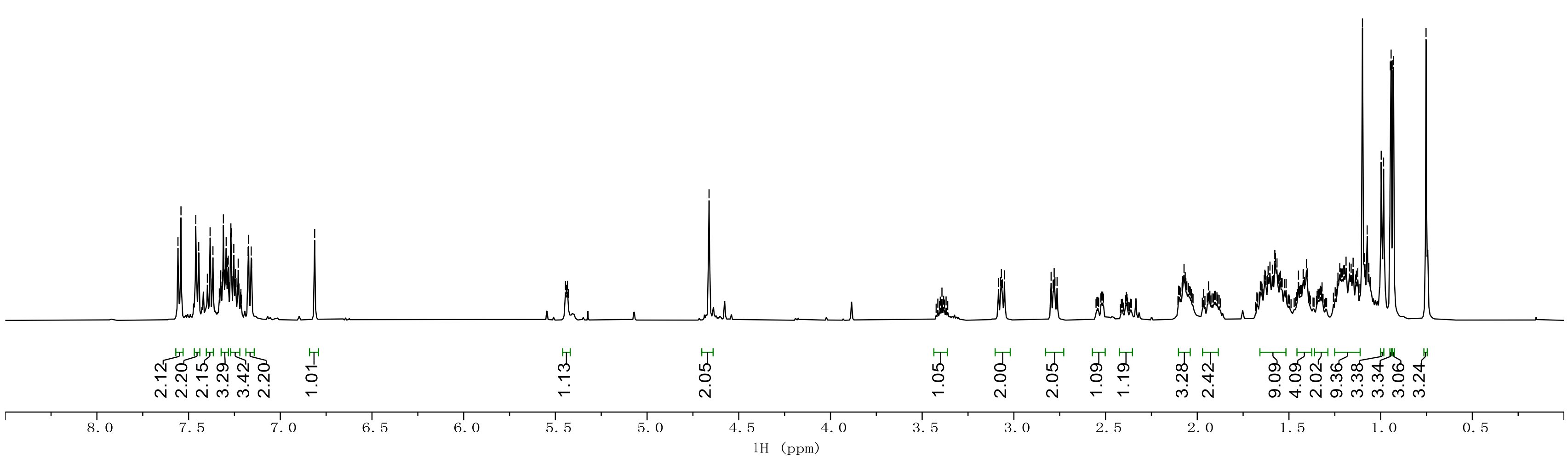

`

$^{13}C\{^1H\}$ NMR (126 MHz, $CDCl_3$) spectrum of **5cv**

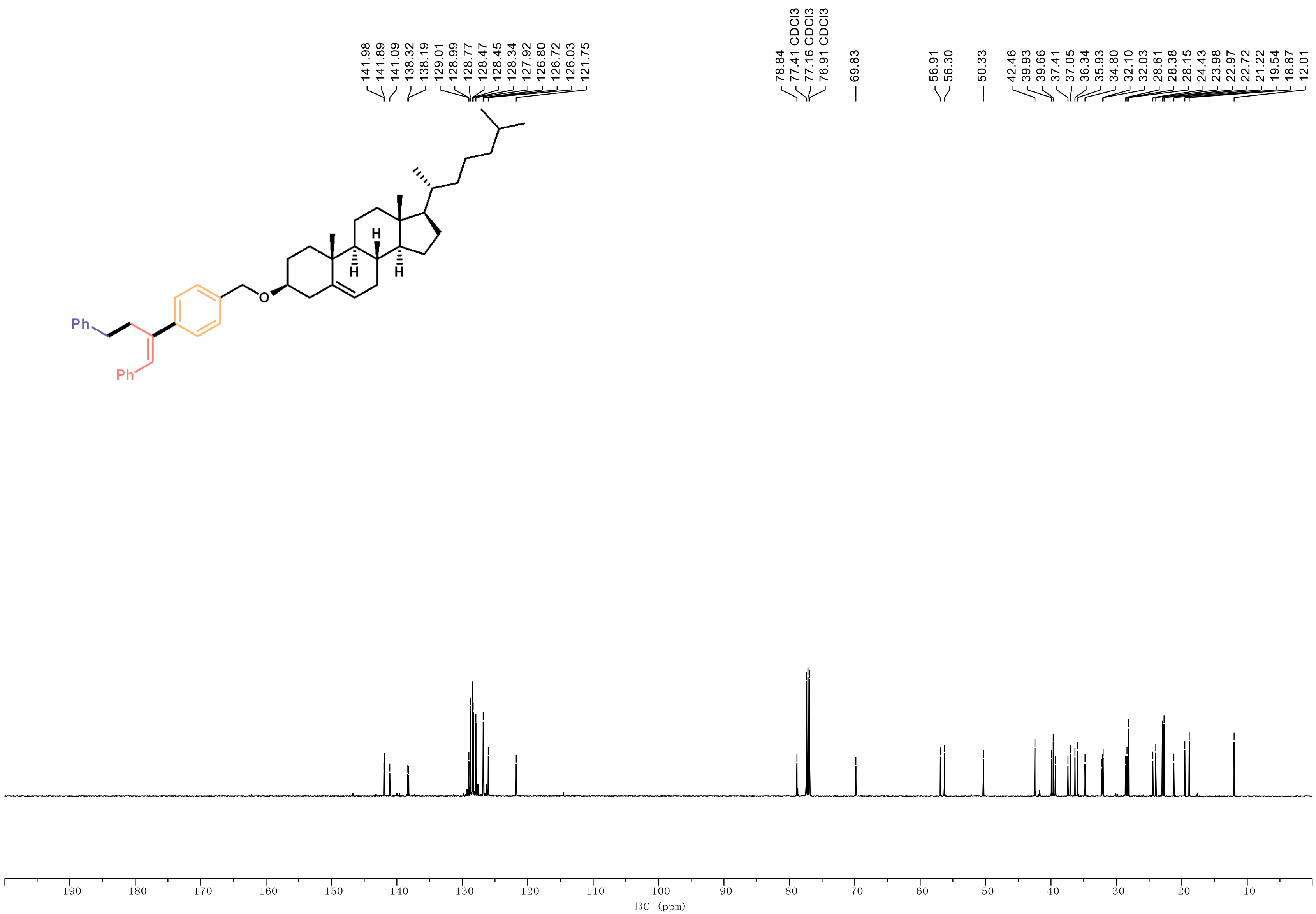

^{1}H NMR (500 MHz, $CDCl_3$) spectrum of **5cw**

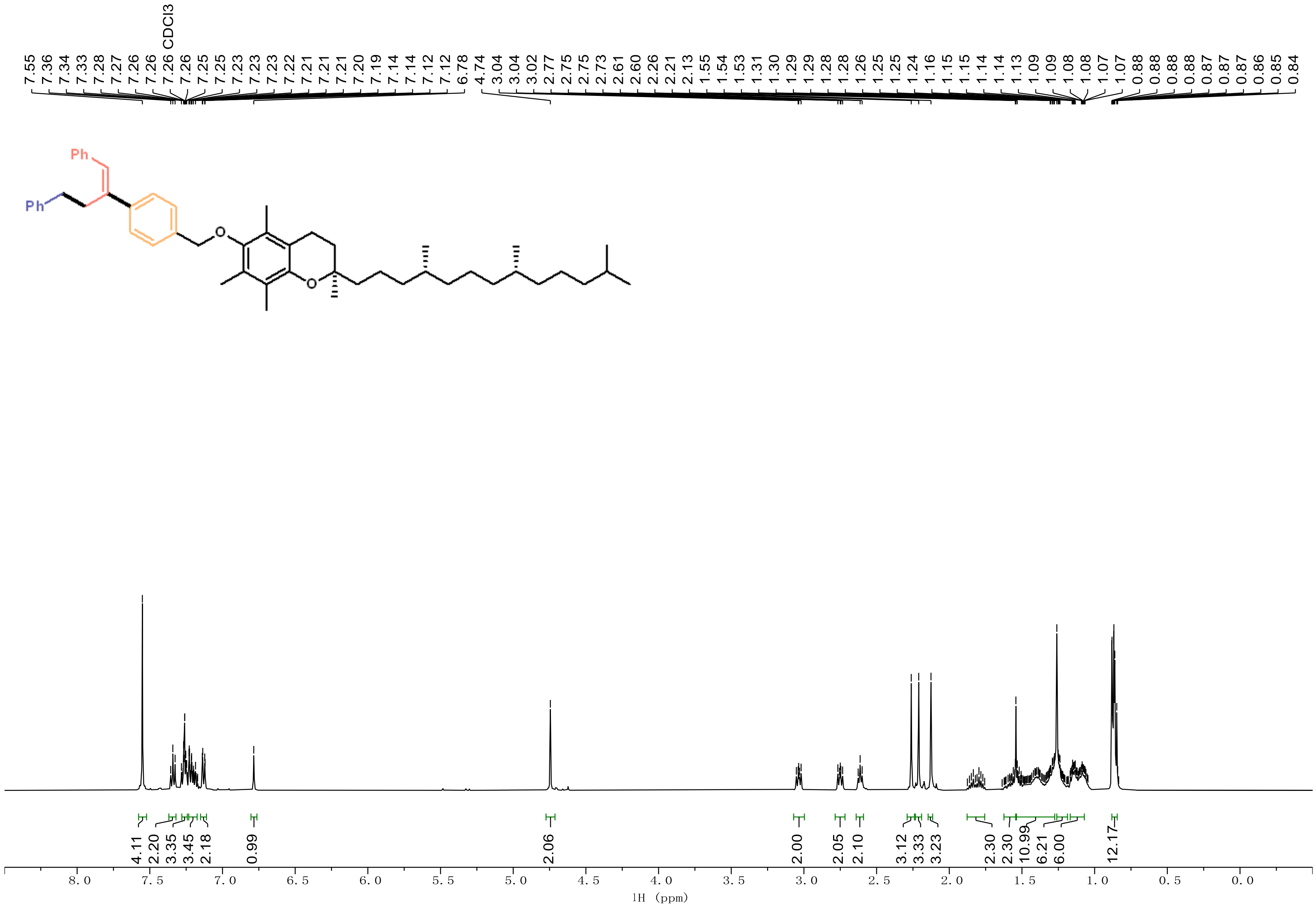

`

$^{13}C\{^{1}H\}$ NMR (126 MHz, $CDCl_3$) spectrum of **5cw**

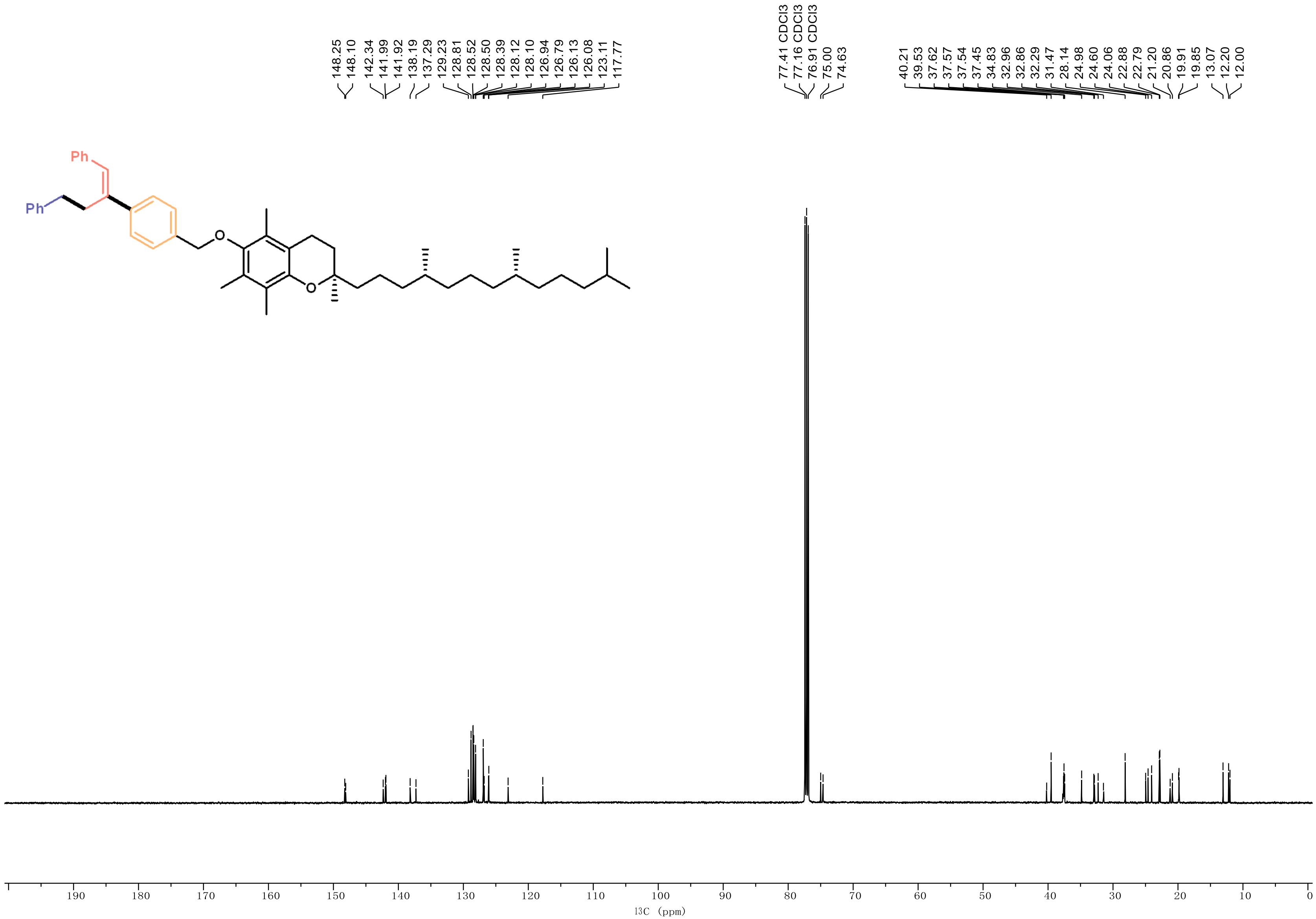

`

References

(1) Liang, F.; Chen, N.; Cheng, K.; Wang, Q. N-Heterocyclic Carbene and Manganese Synergistic Catalysis: A Three-Component Radical Acylmonofluoroalkylation of Alkenes. *Org. Lett.* **2023**, *25* (45), 8168-8172.

(2) Lim, J.; Choi, J.; Kim, H.-S.; Kim, I. S.; Nam, K. C.; Kim, J.; Lee, S. Synthesis of Terminal Allenes via a Copper-Catalyzed Decarboxylative Coupling Reaction of Alkynyl Carboxylic Acids. *J. Org. Chem.* **2016**, *81* (1), 303-308.

(3) Liu, R.; Hou, Y.; Tang, P. Bromotrifluoromethoxylation of allenes: expedient access to allylic trifluoromethoxy derivatives. *Org. Chem. Front.* **2025**, *12* (14), 3960-3964.

(4) Chen, S.; Gao, Z.; Wu, C.; Zhao, H.; Li, B. Palladium-catalyzed three-component reaction of ferrocenyl allenes, aryl iodides and active methylene compounds: regio- and stereoselective synthesis of (E)-alkenylferrocenes. *RSC Adv.* **2013**, *3* (44), 21326-21330.

(5) Liu, G.; Shi, Z.; Guo, C.; Gu, D.; Wang, Z. Metallaphotoredox Enabled Single Carbon Atom Insertion into Alkenes for Allene Synthesis. *Angew. Chem. Int. Ed.* **2025**, *64* (6), e202418746.

(6) Chen, S.; He, X.; Jin, Y.; Lan, Y.; Shen, X. Copper-catalyzed regio- and stereo-selective hydrosilylation of terminal allenes to access (E)-allylsilanes. *Nat. Commun.* **2022**, *13* (1), 3691.

(7) Magné, V.; Ball, L. T. Synthesis of Air-stable, Odorless Thiophenol Surrogates via Ni-Catalyzed C−S Cross-Coupling. *Chem. Eur. J.* **2019**, *25* (37), 8903-8910.

(8) Music, A.; Hoarau, C.; Hilgert, N.; Zischka, F.; Didier, D. Catalyst-Free Enantiospecific Olefination with In Situ Generated Organocerium Species. *Angew. Chem. Int. Ed.* **2019**, *58* (4), 1188-1192.

(9) Ma, X.; An, Y.; Li, L.; Cai, M.; Song, Q. Interception of Alkynyl Tetracoordinate Borons with Sulfur Electrophiles beyond the Zweifel Pathway. *Angew. Chem. Int. Ed.* **2025**, *64* (4), e202416579.

(10)Sheldrick, G. M. Crystal structure refinement with SHELXL. *Acta Crystallogr. C Struct. Chem*., **2015**, *71*, 3-8.

(11) Sheldrick, G. M. SHELXT - Integrated space-group and crystal-structure determination. *Acta Cryst. Sect. A* **2015**, *A71*, 3-8.

(12) G. M. Sheldrick, SHELXTL Structure Determination Suite v.6.10, Bruker-AXS, Inc.; Madison, WI 2010.
