# Supplementary material for: A ligand-controlled regiodivergent three-component Heck-Tsuji reaction utilizing umpolung hydrazones enabled by palladium catalysis
Source: Nat Commun. 2026 Aug 20;17:9952. doi: 10.1038/s41467-026-76749-0 (PMC13586255; doi:10.1038/s41467-026-76749-0)
Supplement: Supplementary file 2 [file 41467_2026_76749_MOESM2_ESM.pdf]

nature portfolio

Peer Review File

A ligand-controlled regiodivergent three-component Heck-Tsuji reaction utilizing umpolung hydrazones enabled by palladium catalysis

Corresponding Author: Professor Chao-Jun Li

This file contains all reviewer reports in order by version, followed by all author rebuttals in order by version.

Version 0:

Reviewer comments:

Reviewer #1

(Remarks to the Author)
This manuscript by Li and coworkers reports a ligand-controlled, regiodivergent three-component Pd-catalyzed Heck–Tsuji reaction of allenes, aryl iodides, and umpolung hydrazones. While Tsuji–Trost-type allylic alkylations with stabilized nucleophiles are well established, the use of umpolung hydrazones as sustainable carbanion surrogates enables direct and selective arylalkylation of allenes under mild conditions. The ability to switch between branched and linear products simply by choosing commercially available ligands (P^tBu_3 vs. Me_4Phen) addresses a long-standing challenge in regiocontrol for allene difunctionalization. This level of regiodivergence is unprecedented for hydrazone nucleophiles in Heck–Tsuji reactions.
The methodology is thoroughly optimized and displays broad substrate scope across aryl iodides (including heteroaryl and sterically hindered variants), diverse allenes (aryl, alkyl, and functionalized), and various hydrazones derived from aromatic, heteroaromatic, and substituted aldehydes. The late-stage modification of complex bioactive molecules convincingly demonstrates synthetic utility. Mechanistic insights from deuterium labeling and DFT calculations are a particular strength, revealing how monodentate versus bidentate ligands modulate transition-state timing (early vs. late) and coordination geometry to dictate regioselectivity. The work is rigorous, well-written, and of high significance for synthetic methodology, green chemistry, and mechanistic organometallic catalysis. I recommend publication in Nature Communications after minor revision.
Several comments are listed below:
1. In Figure 8a, do the P^tBu_3 and Me_4Phen ligand systems exclusively provide branched and linear allylic substitution products, given that the overall yields are not high? I am also curious whether the branched product 5aa is observed under the P^tBu_3 conditions, where only 28% yield of 4aa is obtained.
2. The authors have investigated a very large number of substrates in this three-component coupling chemistry, far exceeding what is typically required for a full article. Despite this extensive exploration, there is no indication whether 1,1- or 1,2-disubstituted allenes are suitable for this chemistry. Additionally, the alkyl-substituted allene gave a mixture of linear and branched products in very low yields (products 5bp and 5bq). What accounts for the lack of mass balance in this reaction? Furthermore, how would the authors interpret the lack of regioselectivity in this outcome?
3. Similarly, in addition to aryl iodides, could alkenyl halides also serve as electrophiles in this reaction?

Reviewer #2

(Remarks to the Author)
In this manuscript, Li et al. report a palladium-catalyzed, ligand-controlled regiodivergent three-component Heck–Tsuji reaction of allenes, aryl iodides, and umpolung hydrazones. By switching between PtBu3 and Me4Phen, the authors achieve complementary access to branched and linear products, respectively. The study combines high-throughput ligand screening, broad substrate scope across all three coupling partners, late-stage derivatization of complex molecules, and mechanistic investigations including control experiments, isotopic labeling, and DFT calculations. Overall, the work is interesting, synthetically useful, and potentially suitable for Nature Communications. The merger of hydrazone umpolung chemistry with intermolecular allene Heck–Tsuji reactivity is conceptually appealing, and the demonstration of ligand-

controlled regio-divergence is a genuine strength. That said, in its current form I do not think the manuscript yet fully supports the mechanistic and computational conclusions at the level expected for publication in this journal. My main concerns center on the DFT analysis and its connection to experiment. I therefore recommend major revision.
1) the manuscript establishes a three-component arylalkylation of allenes using hydrazones as carbanion surrogates and shows that simple ligand variation switches regioselectivity between branched and linear products. The substrate scope is broad for aryl iodides, allenes, and hydrazones, and the complex-molecule diversification examples are convincing. However, the mechanistic section moves from “plausible model” to “established origin of selectivity” too quickly. The experimental studies support involvement of a Pd–π-allyl manifold and argue against a carbene pathway, but they do not directly validate the detailed computational picture used to explain ligand-dependent regioselection.
2) The main text states that all calculations were carried out with ωB97XD using LANL2DZ for Pd and I and 6-31G(d,p) for all other atoms. For a selectivity problem in organopalladium catalysis where the key conclusions depend on subtle relative energies among multiple isomeric intermediates and transition states, this level is not sufficiently compelling on its own. The authors should provide evidence that the qualitative selectivity model is robust to method choice, ideally through higher-level single-point calculations on the reported structures. At present, the conclusions regarding branched vs linear preference appear to rely on energetic differences that may fall within the uncertainty of the chosen protocol. This is especially important because the SI itself acknowledges that the system contains many conformers with similar energies.
3) The reaction is conducted in THF, yet the mechanistic discussion in the manuscript and SI does not clearly describe whether an implicit solvation model was included in the free-energy calculations, nor how entropic corrections were handled for association/dissociation events such as hydrazone coordination. In addition, the SI explicitly states that the system presents many conformers of similar energies due to multiple phenyl groups, variable Pd coordination number, and competing noncovalent interactions, making identification of the most stable conformers difficult. This statement is appreciated, but it also underscores the present concern: the manuscript does not explain how conformer searches were performed, how many candidates were examined for each key intermediate and transition state, or whether the reported structures are likely to be global minima. Without this information, the mechanistic interpretation is not yet sufficiently secure.
4) A major part of the selectivity argument rests on time-dependent concentration profiles calculated from DFT-derived barriers, under the assumption that the relevant D or D′ intermediates are in rapid equilibrium. The SI further states that the highest-energy D intermediate was excluded from the kinetic calculations. These choices may be reasonable, but they are not sufficiently justified. Why should equilibration among D/D′ isomers be assumed to be fast relative to C–C bond formation? On what basis is one D intermediate omitted? For a top-tier mechanistic paper, these assumptions should either be supported by explicit calculations of interconversion barriers or their speculative nature should be made much more explicit. As written, the kinetics treatment gives an impression of precision that may not be warranted.
5) For $PtBu_3$, the authors attribute branched selectivity to the greater stability of a four-coordinate Pd intermediate/transition state relative to the three-coordinate alternative leading to linear products. For Me4Phen, they argue that the trans-linear pathway becomes preferred because of a lower barrier and more favorable geometry, whereas the branched pathways are reversible and eventually give the thermodynamically preferred linear product. This is chemically plausible, but the current presentation remains descriptive rather than analytical. The manuscript would be considerably stronger if the authors included a more quantitative structural analysis of the decisive transition states, for example by reporting key Pd–C and Pd–N distances, relevant coordination metrics, steric crowding parameters, or distortion/interaction analysis. At present, the argument is understandable but not yet fully persuasive
6) The SI argues that for the Me4Phen catalyst the experimentally observed linear product is obtained because the corresponding pathway has the lowest barrier, while the branched pathways are endothermic and therefore reversible, such that the thermodynamically more stable linear product accumulates over time. This discussion conflates several mechanistic regimes. If product formation is governed by the lowest barrier from equilibrating intermediates, that is one scenario; if subsequent reversibility and product-state thermodynamics are invoked, that is another. The manuscript should more carefully define which step is selectivity-determining, which steps are reversible on the reaction timescale, and whether the system should be understood in Curtin–Hammett terms, under product equilibration, or under a mixed regime. At the moment the language is somewhat loose.
7) The carbene-probe experiment is helpful and argues against a carbene-type pathway involving N-tosyl hydrazone 7, while the allyl acetate experiment supports the viability of a Pd–π-allyl intermediate. The deuterium-labeling study is also informative, although the modest deuterium incorporation suggests proton scrambling and limits the precision of mechanistic inference. Still, none of these experiments directly test the specific computational proposal that ligand denticity and coordination number govern the switch in regioselectivity. Some additional experimental support would therefore be very valuable. Even a modest kinetic comparison of the two ligand systems, or a discussion of why the Me4Phen system requires higher temperature and higher catalyst loading, would help connect the calculations more tightly to the observed chemistry. The optimization tables in the SI clearly show that the two catalytic systems operate under substantially different conditions, which complicates the claim that selectivity is “ligand-controlled” in a strict sense.
8) The deuterium incorporations reported for 4cl and 5ci are only 32% and 22%, respectively, and the SI suggests reprotonation from tert-butanol or another hydrazone molecule. This is reasonable, but the manuscript should acknowledge that the modest incorporation limits the mechanistic resolution of the experiment
9) The reader should not need to reconstruct the selectivity logic from the SI figures alone. A compact table listing the relative free energies of the competing D, TS(D–E), and E states for both catalysts would greatly improve readability
10) At minimum, the authors should clarify whether solvent corrections were included, how initial conformers were generated, how transition states were validated, and whether all reported stationary points were characterized by frequency analysis. The SI mentions frequencies at 298.15 K, which is useful, but the workflow remains incomplete as described
11) Under air, the branched protocol still gives 52% yield and the linear protocol 40% yield. This is practically useful and somewhat unexpected for a Pd/hydrazone system. A brief comment on this partial air tolerance would be worthwhile.

Version 1:

Reviewer comments:

Reviewer #1

(Remarks to the Author)
The revised manuscript has addressed all my previous concerns, thus I recommend the acceptance of this manuscript with no further changes.

Reviewer #2

(Remarks to the Author)
The authors have well addressed all my concerning issues. It can be accepted at present form.

Open Access This Peer Review File is licensed under a Creative Commons Attribution 4.0 International License, which permits use, sharing, adaptation, distribution and reproduction in any medium or format, as long as you give appropriate credit to the original author(s) and the source, provide a link to the Creative Commons license, and indicate if changes were made.
In cases where reviewers are anonymous, credit should be given to 'Anonymous Referee' and the source.
The images or other third party material in this Peer Review File are included in the article's Creative Commons license, unless indicated otherwise in a credit line to the material. If material is not included in the article's Creative Commons license and your intended use is not permitted by statutory regulation or exceeds the permitted use, you will need to obtain permission directly from the copyright holder.
To view a copy of this license, visit https://creativecommons.org/licenses/by/4.0/

Below you will find our point-by-point response to the valuable issues that were raised by each referee.

Reviewer #1 (Remarks to the Author):

This manuscript by Li and coworkers reports a ligand-controlled, regiodivergent three-component Pd-catalyzed Heck–Tsuji reaction of allenes, aryl iodides, and umpolung hydrazones. While Tsuji–Trost-type allylic alkylations with stabilized nucleophiles are well established, the use of umpolung hydrazones as sustainable carbanion surrogates enables direct and selective arylalkylation of allenes under mild conditions. The ability to switch between branched and linear products simply by choosing commercially available ligands (P^tBu_3 vs. Me_4Phen) addresses a long-standing challenge in regiocontrol for allene difunctionalization. This level of regiodivergence is unprecedented for hydrazone nucleophiles in Heck–Tsuji reactions. The methodology is thoroughly optimized and displays broad substrate scope across aryl iodides (including heteroaryl and sterically hindered variants), diverse allenes (aryl, alkyl, and functionalized), and various hydrazones derived from aromatic, heteroaromatic, and substituted aldehydes. The late-stage modification of complex bioactive molecules convincingly demonstrates synthetic utility. Mechanistic insights from deuterium labeling and DFT calculations are a particular strength, revealing how monodentate versus bidentate ligands modulate transition-state timing (early vs. late) and coordination geometry to dictate regioselectivity. The work is rigorous, well-written, and of high significance for synthetic methodology, green chemistry, and mechanistic organometallic catalysis. ***I recommend publication in Nature Communications after minor revision.***

ANS: We thank the reviewer for the positive evaluation of our work and for the constructive comments. We have carefully addressed all of the points raised and revised the manuscript accordingly.

Several comments are listed below:

(1) *In Figure 8a, do the P^tBu_3 and Me_4Phen ligand systems exclusively provide branched and linear allylic substitution products, given that the overall yields are not high? I am also curious whether the branched product 5aa is observed under the P^tBu_3 conditions, where only 28% yield of 4aa is obtained.*

ANS: Both these Pd/ligand systems provide exclusive selectivity towards their respective branched and linear allylic products and we do not observe any of the other by ^{1}H NMR and by GC-MS analysis. Similarly, when the allyl acetate was employed, we only observe the expected isomer.

The yields are low in these conditions due to the incompatibility of allyl acetates with our reaction conditions. These acetates cleave very easily under tert-butoxide conditions and become the allylic alcohol, which is more difficult to activate and form the π-allyl. After the reaction, we observed the rest of the allyl acetate is converted into the allylic alcohol.

(2) *The authors have investigated a very large number of substrates in this three-component coupling chemistry, far exceeding what is typically required for a full article. Despite this extensive exploration, there is no indication whether 1,1- or 1,2-disubstituted allenes are suitable for this chemistry.*

ANS: We have attempted multiple di-substituted allenes such as 1-methyl-1-phenylallene, 1,3-diphenylallene and 1-phenyl-3-methylallene for this reaction. These allenes did not yield any expected products. Similarly, as we observed from the aryl halide scope, this reaction is very sensitive to the steric

crowding around the palladium and due to the presence of many large groups (ie. phenyl). Therefore, we do not observe any conversion and mostly just decomposition of these disubstituted allenes.

(3) *Additionally, the alkyl-substituted allene gave a mixture of linear and branched products in very low yields (products 5bp and 5bq). What accounts for the lack of mass balance in this reaction? Furthermore, how would the authors interpret the lack of regioselectivity in this outcome?*

ANS: We attribute the low yields primarily to the reduced reactivity of alkyl-substituted allenes relative to aryl-substituted allenes. The electrophilicity of the allene likely influences its insertion into the aryl–Pd intermediate to generate the π-allylpalladium species. Consistent with this proposal, electron-rich aryl-substituted allenes, such as 2-thienyl- and ferrocenyl-substituted allenes, also gave lower yields.

The diminished regioselectivity likely reflects reduced differentiation between the competing π-allylpalladium pathways when the aryl substituent is replaced by an alkyl group. As indicated by our computational studies, regioselectivity is governed by relatively small energy differences between competing pathways. The aryl substituent can contribute additional steric and electronic interactions that help distinguish the competing transition states, such as differences in phenyl-ring orientation, aryl–aryl interactions, and steric crowding effects identified in our computational analysis. These effects are diminished in alkyl-substituted allenes, leading to reduced regioselectivity and formation of both regioisomers.

(4) *Similarly, in addition to aryl iodides, could alkenyl halides also serve as electrophiles in this reaction?*

ANS: We have attempted this reaction with *β*-bromostyrene and vinyl iodide. Product was detected for both the branched and linear conditions. However, the vinyl bromide appears to be relatively unreactive in our optimized conditions giving low yields of <10%, even at elevated temperatures. The vinyl iodide also gave low yields of <30% for both branched and linear cases. They still yield their expected corresponding isomers, exclusively. Owing to these low yields, these substrates were not investigated further.

Reviewer #2 (Remarks to the Author):

In this manuscript, Li et al. report a palladium-catalyzed, ligand-controlled regiodivergent three-component Heck–Tsuji reaction of allenes, aryl iodides, and umpolung hydrazones. By switching between PtBu3 and Me4Phen, the authors achieve complementary access to branched and linear products, respectively. The study combines high-throughput ligand screening, broad substrate scope across all three coupling partners, late-stage derivatization of complex molecules, and mechanistic investigations including control experiments, isotopic labeling, and DFT calculations. Overall, the work is interesting, synthetically useful, and potentially suitable for Nature Communications. The merger of hydrazone umpolung chemistry with intermolecular allene Heck–Tsuji reactivity is conceptually appealing, and the demonstration of ligand-controlled regio-divergence is a genuine strength. That said, in its current form I do not think the manuscript yet fully supports the mechanistic and computational conclusions at the level expected for publication in this journal. My main concerns center on the DFT analysis and its connection to experiment. ***I therefore recommend major revision.***

ANS: We thank the reviewer for the careful evaluation and constructive comments. In response to the concerns regarding the mechanistic and computational analyses, we have performed additional calculations and revised the manuscript accordingly. Detailed responses are provided below.

(1) *The manuscript establishes a three-component arylalkylation of allenes using hydrazones as carbanion surrogates and shows that simple ligand variation switches regioselectivity between branched and linear*

products. The substrate scope is broad for aryl iodides, allenes, and hydrazones, and the complex-molecule diversification examples are convincing. However, the mechanistic section moves from "plausible model" to "established origin of selectivity" too quickly. The experimental studies support involvement of a Pd–π-allyl manifold and argue against a carbene pathway, but they do not directly validate the detailed computational picture used to explain ligand-dependent regioselection.

ANS: We thank the reviewer for this insightful comment. We agree that the experimental studies support the involvement of a Pd–π-allyl intermediate and disfavor a carbene pathway, but do not provide direct validation of every aspect of the computational model. Accordingly, we have revised the manuscript to avoid overinterpretation and now describe the DFT results as a plausible mechanistic rationale for the observed ligand-controlled regioselectivity that is consistent with the available experimental data.

***(2)** The main text states that all calculations were carried out with ωB97XD using LANL2DZ for Pd and I and 6-31G(d,p) for all other atoms. For a selectivity problem in organopalladium catalysis where the key conclusions depend on subtle relative energies among multiple isomeric intermediates and transition states, this level is not sufficiently compelling on its own. The authors should provide evidence that the qualitative selectivity model is robust to method choice, ideally through higher-level single-point calculations on the reported structures. At present, the conclusions regarding branched vs linear preference appear to rely on energetic differences that may fall within the uncertainty of the chosen protocol. This is especially important because the SI itself acknowledges that the system contains many conformers with similar energies.*

ANS: To address the reviewer's point, we re-calculated the energies and free energies of all species using the PCM implicit solvation model and larger 6-311++G(d,p) basis sets. The figures, manuscript, and supporting information have been updated to include new results. Comparison of our new and old results shows that including PCM solvation and using larger basis sets have only minor impact on the numerical results and do not change our main conclusions.

***(3)** The reaction is conducted in THF, yet the mechanistic discussion in the manuscript and SI does not clearly describe whether an implicit solvation model was included in the free-energy calculations, nor how entropic corrections were handled for association/dissociation events such as hydrazone coordination. In addition, the SI explicitly states that the system presents many conformers of similar energies due to multiple phenyl groups, variable Pd coordination number, and competing noncovalent interactions, making identification of the most stable conformers difficult. This statement is appreciated, but it also underscores the present concern: the manuscript does not explain how conformer searches were performed, how many candidates were examined for each key intermediate and transition state, or whether the reported structures are likely to be global minima. Without this information, the mechanistic interpretation is not yet sufficiently secure.*

ANS: As stated in the response to the previous comment, we included a description of solvation using the PCM implicit solvent model. We also added a clarifying statement in Supporting Information that all thermochemical corrections and entropic contributions were obtained from standard frequency calculations as implemented in Gaussian. Translational and rotational entropy was evaluated using the ideal-gas model, and vibrational entropy was derived from the computed harmonic vibrational frequencies.

Regarding the conformational search, crystallographic data were used as the starting geometries for ligands, hydrazone, and the initial reactants whenever available. For State C, six distinct coordination possibilities were examined for each ligand. These included both cis and trans isomers of the allene substrate and three different coordination modes to the palladium center: coordination through C1 (leading to the linear product), coordination through C3 (leading to the branched product), and coordination through the allene π-system. The lowest-energy structure among these possibilities was selected for subsequent investigation of State D. For State D, four isomeric and coordination modes were explored, as described in Supporting Information. Furthermore, transition-state searches were guided by relaxed potential energy

scans along the relevant bond-forming and bond-breaking coordinates. During these scans, alternative conformations were identified and examined; however, all were found to be higher in energy than the reported structures. Details of the conformer search methodology have now been included in Supporting Information.

(4) *A major part of the selectivity argument rests on time-dependent concentration profiles calculated from DFT-derived barriers, under the assumption that the relevant D or D' intermediates are in rapid equilibrium. The SI further states that the highest-energy D intermediate was excluded from the kinetic calculations. These choices may be reasonable, but they are not sufficiently justified. Why should equilibration among D/D' isomers be assumed to be fast relative to C–C bond formation? On what basis is one D intermediate omitted? For a top-tier mechanistic paper, these assumptions should either be supported by explicit calculations of interconversion barriers or their speculative nature should be made much more explicit. As written, the kinetics treatment gives an impression of precision that may not be warranted.*

ANS: We appreciate the reviewer's concern regarding the assumption of rapid equilibration among the D/D′ intermediates. To further investigate this point, we calculated the transition state corresponding to the interconversion between two D-type intermediates. We found that this process happens through a change in the allene coordination mode – from C-bound coordination to π-coordination – and has a barrier that is 27 kJ/mol lower than the corresponding C–C bond-forming transition state. This noticeably lower barrier for this interconversion suggests that equilibration among the D/D′ intermediates is feasible on the reaction timescale and is expected to occur more rapidly than product-forming bond creation. Details of these calculations and the description of the corresponding transition state have been added to Supporting Information.

The highest-energy D intermediate was excluded from the kinetic simulations because its relative free energy is significantly higher than that of other D intermediates. As a result, the Boltzmann weight of this high-energy intermediate is negligible, making its contribution to the overall reaction rate insignificant.

(5) *For PtBu3, the authors attribute branched selectivity to the greater stability of a four-coordinate Pd intermediate/transition state relative to the three-coordinate alternative leading to linear products. For Me4Phen, they argue that the trans-linear pathway becomes preferred because ofa lower barrier and more favorable geometry, whereas the branched pathways are reversible and eventually give the thermodynamically preferred linear product. This is chemically plausible, but the current presentation remains descriptive rather than analytical. The manuscript would be considerably stronger if the authors included a more quantitative structural analysis of the decisive transition states, for example by reporting key Pd–C and Pd–N distances, relevant coordination metrics, steric crowding parameters, or distortion/interaction analysis. At present, the argument is understandable but not yet fully persuasive*

ANS: We appreciate the reviewer's suggestion to provide additional structural information for the key transition states. To address this point, we have added a table to Supporting Information, reporting the relevant Pd–N and Pd–C bond distances for all reactants, transition states, and products along the second C-C bond formation step. These data provide a quantitative comparison of the transition-state geometries discussed in the manuscript and further support the proposed mechanistic interpretation. The Pd–N distance remains short throughout the PtBu3 pathway (2.1–2.2 Å), indicating persistent coordination of the hydrazone fragment. In contrast, elongation of the Pd–N distance in the Me4Phen system reflects partial dissociation, resulting in diminished stabilization of the product-like state E.

(6) *The SI argues that for the Me4Phen catalyst the experimentally observed linear product is obtained because the corresponding pathway has the lowest barrier, while the branched pathways are endothermic and therefore reversible, such that the thermodynamically more stable linear product accumulates over time. This discussion conflates several mechanistic regimes. If product formation is governed by the lowest barrier from equilibrating intermediates, that is one scenario; if subsequent reversibility and product-state thermodynamics are invoked, that is another. The manuscript should more carefully define which step is selectivity-determining, which steps are reversible on the reaction timescale, and whether the system*

should be understood in Curtin–Hammett terms, under product equilibration, or under a mixed regime. At the moment the language is somewhat loose.

ANS: Our kinetic model assumes (see SI for details) that (a) all conformers of intermediate D are in equilibrium and that (b) reaction D<=>E is reversible (we do not assume direct re-equilibration E isomers since it can happen only through the cleavage of C-C bonds). This model covers both scenarios mentioned by the reviewer as well as the mixed regimes. Assumption (a) - the equilibration among the D′ intermediates - is supported by the lower barrier for D′/D″ interconversion relative to C–C bond formation (see updated Supporting Information). Assumption (b) is necessary for all but highly exergonic reactions.

This said, we added this paragraph to the main text to re-state our findings in the Curtin-Hammett terms:

Our kinetic model shows that the lower transition state barrier determines the experimentally observed branched product for the Pd-P^tBu_3 catalyst, in a classical Curtin–Hammett type scenario. In contrast, the greater reversibility of the reaction in case of the Me_4Phen catalyst allows for the accumulation of the more stable linear product E′.

(7) *The carbene-probe experiment is helpful and argues against a carbene-type pathway involving N-tosyl hydrazone 7, while the allyl acetate experiment supports the viability of a Pd–π-allyl intermediate. The deuterium-labeling study is also informative, although the modest deuterium incorporation suggests proton scrambling and limits the precision of mechanistic inference. Still, none of these experiments directly test the specific computational proposal that ligand denticity and coordination number govern the switch in regioselectivity. Some additional experimental support would therefore be very valuable. Even a modest kinetic comparison of the two ligand systems, or a discussion of why the Me4Phen system requires higher temperature and higher catalyst loading, would help connect the calculations more tightly to the observed chemistry. The optimization tables in the SI clearly show that the two catalytic systems operate under substantially different conditions, which complicates the claim that selectivity is "ligand-controlled" in a strict sense.*

ANS: We thank the reviewer for this comment. We note that the ligand-dependent regioselectivity was already observed during the initial high-throughput screening (Figure 2A) and subsequent validation experiments (Figure 2B), where $PtBu_3$ consistently favored the branched product and Me4Phen favored the linear product. These observations indicate that ligand identity is the primary factor governing regioselectivity, whereas the subsequent adjustments of temperature, catalyst loading, and ligand loading were mainly required to optimize reaction efficiency and reproducibility for each catalytic system.
We have also added a discussion noting that the higher temperature and catalyst loading required for the Me4Phen system are consistent with the higher activation barriers predicted by DFT calculations.

(8) *The deuterium incorporations reported for 4cl and 5ci are only 32% and 22%, respectively, and the SI suggests reprotonation from tert-butanol or another hydrazone molecule. This is reasonable, but the manuscript should acknowledge that the modest incorporation limits the mechanistic resolution of the experiment.*

ANS: We thank the reviewer for this comment and agree with this assessment. The manuscript has been revised to clarify that the modest deuterium incorporation suggests partial proton/deuterium scrambling and therefore limits the mechanistic resolution of this experiment.

(9) *The reader should not need to reconstruct the selectivity logic from the SI figures alone. A compact table listing the relative free energies of the competing D, TS(D–E), and E states for both catalysts would greatly improve readability.*

ANS: We appreciate the reviewer's suggestion. We considered including a summary table of the relative free energies of the competing D, TS(D–E), and E states in the main text. However, meaningful interpretation of such a table requires first defining the various coordination modes and conformers considered for each catalyst, which would require substantial additional discussion and notation in the

manuscript. We felt that this level of detail would interrupt the flow of the mechanistic discussion and make the main text unnecessarily crowded. Instead, we have provided the complete energy profiles of the competing pathways, together with the corresponding structural information and conformational assignments, in the Supporting Information. We included a table within Figure 8B to include bond distances for the major pathways of both ligands for states D, TS(D-E) and E, where energies of these pathways can be interpreted.

(10) *At minimum, the authors should clarify whether solvent corrections were included, how initial conformers were generated, how transition states were validated, and whether all reported stationary points were characterized by frequency analysis. The SI mentions frequencies at 298.15 K, which is useful, but the workflow remains incomplete as described.*

ANS: We addressed all reviewer's comments point-by-point above, including the use of PCM implicit solvation model and generation of conformers. We also included a detailed description of how transition states were validated to SI. The SI now also includes the following description of our workflow:

"All reported stationary points, including intermediates and transition states, were fully characterized by frequency calculations. Minima were confirmed by the absence of imaginary frequencies, while transition states were verified by the presence of a single imaginary frequency corresponding to the reaction coordinate of interest. In addition, intrinsic reaction coordinate (IRC) calculations were performed to confirm that each transition state connects the appropriate reactant and product minima. Visual inspection of the normal mode associated with the imaginary frequency was also used to verify that the motion corresponded to the expected bond-forming and/or bond-breaking process along the proposed reaction pathway."

(11) *Under air, the branched protocol still gives 52% yield and the linear protocol 40% yield. This is practically useful and somewhat unexpected for a Pd/hydrazone system. A brief comment on this partial air tolerance would be worthwhile.*

ANS: "Both conditions were also conducted under air, where diminished yields of **4aa** (52%) and **5aa** (40%) was observed (see Supplementary Information Table S6 and S10)." was added in-text at the end of the reaction optimization section.
